# Supplementary material for: Transition-metal-free silylboronate-mediated cross-couplings of organic fluorides with amines
Source: Nat Commun. 2023 Apr 3;14:1847. doi: 10.1038/s41467-023-37466-0 (PMC10070422; doi:10.1038/s41467-023-37466-0)
Supplement: Supplementary file 1 — Supplementary Information [file 41467_2023_37466_MOESM1_ESM.pdf]

Supplementary Information

Transition-metal-free silylboronate-mediated cross-couplings of organic fluorides with amines

Jun Zhou,[1] Zhengyu Zhao,[1] and Norio Shibata[1,2,*]

[1]*Department of Nanopharmaceutical Sciences, Nagoya Institute of Technology, Gokiso, Showa-ku, Nagoya 466-8555, Japan*

[2]*Department of Life Science and Applied Chemistry, Nagoya Institute of Technology, Gokiso, Showa-ku, Nagoya 466-8555, Japan*

[*]*Correspondence and requests for materials should be addressed to N.S. (email: nozshiba@nitech.ac.jp)*

Table of Contents

1. Supplementary Methods 3
2. Supplementary Tables 4
   2.1 Supplementary Table 1. Screening proper secondary amine for the defluoroamination of aryl fluoride 4
   2.2 Supplementary Table 2. Screening solvent for the defluoroamination of aryl fluoride 5
   2.3 Supplementary Table 3. Reaction time optimization for the defluoroamination of aryl fluoride 6
   2.4 Supplementary Table 4. Evaluation of silylboronates for the defluoroamination of aryl fluoride 7
   2.5 Supplementary Table 5. Variations from the optimal reaction conditions 8
3. Supplementary Notes 9
   3.1 Synthesis of substituted aryl fluorides **1** 9
   3.2 Synthesis of substituted alkyl fluorides **2**, allyl fluoride, and cinnamyl fluoride 26
   3.3 General procedure for the synthesis of *N*-alkyl anilines **3** 30
   3.4 Synthesis of silylboronates 38
   3.5 General procedure for the optimization of defluoroamination reaction 40
   3.6 General procedure for the defluoroamination reaction 40
   3.7 General procedure for the scale-up reaction 82
4. Supplementary Discussion 83
   4.1 The NMR spectroscopic studies 83
   4.2 Reaction with radical scavenger TEMPO 85
   4.3 Chemoselectivites of organic halides 87
   4.4 Mechanism control experiments 89
   4.5 Radical clock experiments 91
   4.6 Copy of NMR spectra 94
5. Supplementary References 214

1. Supplementary Methods

All reactions were performed in oven-dried glassware under a positive pressure of nitrogen or argon. Solvents were transferred via syringe and were introduced into the reaction vessels through a rubber septum. All solvents were dried by standard method. All the reactions were monitored by thin-layer chromatography (TLC) carried out on 0.25 mm Merck silica gel (60-F254). The TLC plates were visualized with UV light. All the reaction products were purified by column chromatography and were carried out on a column packed with silica gel 60N spherical neutral size 50-63 mm. The ^{1}H NMR (300 MHz), ^{13}C NMR (75 MHz) and ^{19}F NMR (282 MHz) spectra as for solution in $CDCl_3$ or D_2O were recorded on a Varian Mercury 300. The ^{1}H NMR (500 MHz) and ^{13}C NMR (126 MHz) spectra as for solution in $CDCl_3$ were recorded on a BRUKER 500 Ultra Shield TR. The ^{1}H NMR (700 MHz) and ^{13}C NMR (176 MHz) spectra as for solution in $CDCl_3$ were recorded on a JEOL RESONANCE ECZ700R. The ^{11}B NMR (225 MHz) spectra as for solution in THF-d^8 were also recorded on a JEOL RESONANCE ECZ700R. The chemical shifts (δ) are expressed in ppm downfield from internal TMS (δ = 0.00) and coupling constants (*J*) are reported in hertz (Hz). The hexafluorobenzene (C_6F_6) [δ = −162.2 ($CDCl_3$)] was used as internal standard for ^{19}F NMR. The following abbreviations were used to explain the multiplicities: s = singlet, d = doublet, t = triplet, q = quartet, m = multiplet, br = broad. Mass spectra were recorded on a JEOL JMS-Q1050GC (EI-MS) and SHIMADZU LCMS-2020 (ESI-MS). High resolution mass spectrometry (HRMS) was carried out on an electron impact ionization mass spectrometer with a micro-TOF analyzer and recorded on a Waters, GCT Premier (EI-MS) with a TOF analyzer. Infrared spectra were recorded on a JASCO FT/IR-4100 spectrometer. Melting points were recorded on a BUCHI M-565.

Commercially available chemicals were obtained from Aldrich Chemical Co., Alfa Aesar, TCI and used as received unless otherwise noted. Solvents acetonitrile, tetrahydrofuran (THF), dioxane, 1,2-dimethoxyethane (DME), diethylene glycol dimethyl ether (diglyme), and cyclopentyl methyl ether (CPME) were dried and distilled before use.

2. Supplementary Tables

2.1 Supplementary Table 1. Screening proper secondary amine for the defluoroamination of aryl fluoride[a]

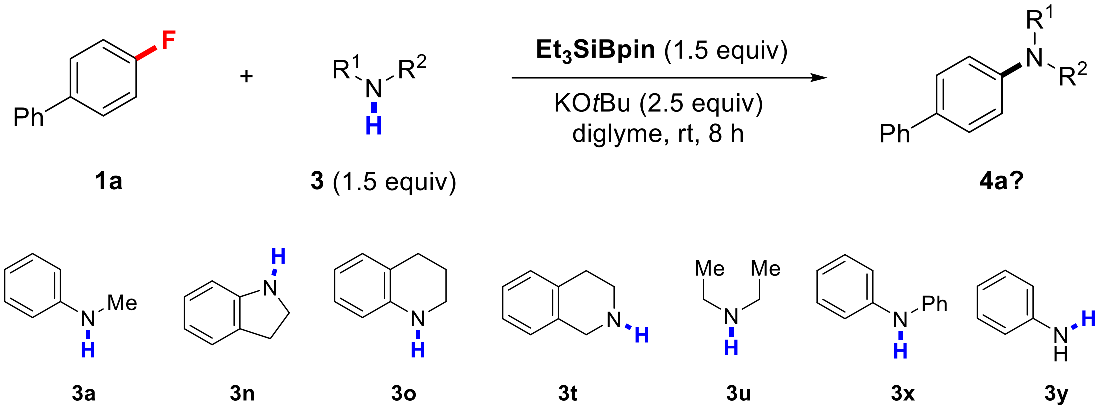

Entry	Amine	4a? (%)[b]
**1**	**3a**	**41**
2	**3n**	16
3	**3o**	14
4	**3t**	9
5	**3u**	trace
6	**3x**	N.R.
7	**3y**	N.R.
8[c]	**3a**	44

[a] Reactions were carried out with **1a** (17.2 mg, 0.1 mmol), **3** (0.15 mmol), $Et_3SiBpin$ (36.3 mg, 0.15 mmol), and KO*t*Bu (28.0 mg, 0.25 mmol) in diglyme (0.5 mL) at room temperature for 8 h.

[b] Yields were determined by ^1H NMR and ^{19}F NMR analysis of the crude reaction mixture using 3-fluoropyridine as an internal standard.

[c] $Et_3SiBpin$ (48.4 mg, 0.2 mmol), and KO*t*Bu (44.8 mg, 0.4 mmol) were used.

2.2 Supplementary Table 2. Screening solvent for the defluoroamination of aryl fluoride[a]

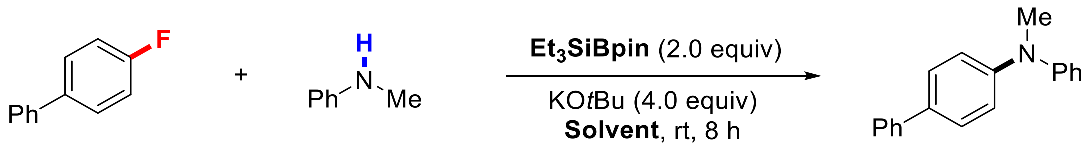

Entry	**Solvent**	Conversion (%)[b]	Yield of **4aa** (%)[b]
1	diglyme	62	58
**2**	**triglyme**	**85**	**81**
3	tetraglyme	81	74
4	dioxane	49	39
5	DME	34	31
6	CPME	--	N.R.
7	THF	29	5
8	**18-crown-6** (4.0 equiv) in THF	78	75
9	cyclohexane	--	N.R.
10	toluene	--	N.R.

[a] Unless otherwise noted, reactions were conducted with **1a** (17.2 mg, 0.1 mmol), **3a** (32 μL, 0.3 mmol), $Et_3SiBpin$ (48.4 mg, 0.2 mmol), KO*t*Bu (44.8 mg, 0.4 mmol) and solvent (0.5 mL) at room temperature for 8 h.

[b] Yields were determined by ^{1}H NMR and ^{19}F NMR analysis of the crude reaction mixture using 3-fluoropyridine as an internal standard.

Triethylene glycol dimethyl ether: triglyme; tetraethylene glycol dimethyl ether: tetraglyme.

2.3 Supplementary Table 3. Reaction time optimization for the defluoroamination of aryl fluoride[a]

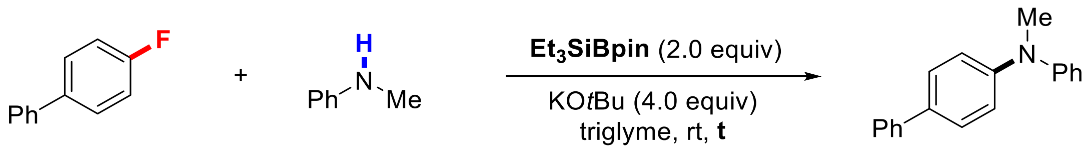

1a **3a** (3.0 equiv) **4aa**

Entry	**t** (h)	Conversion (%)[b]	Yield of **4aa** (%)[b]
1	8 h	85	81
2	2 h	49	47
3	12 h	88	85
**4**[c]	**24 h**	**93**	**91**(88)

[a] Unless otherwise noted, reactions were conducted with **1a** (17.2 mg, 0.1 mmol), **3a** (32 μL, 0.3 mmol), $Et_3SiBpin$ (48.4 mg, 0.2 mmol), KO*t*Bu (44.8 mg, 0.4 mmol) and triglyme (0.5 mL) at room temperature for indicated hours.

[b] Determined by ^{19}F NMR and ^{1}H NMR spectroscopy using 3-fluoropyridine as an internal standard.

[c] The isolated yield was shown in the parenthesis.

2.4 Supplementary Table 4. Evaluation of silylboronates for the defluoroamination of aryl fluoride[a]

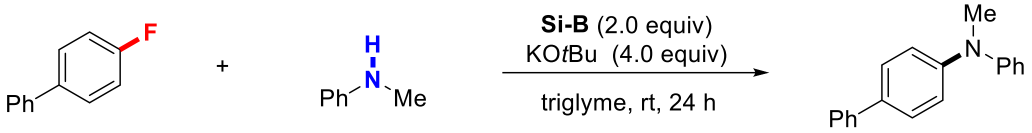

1a **3a** (3.0 equiv) **4aa**

Entry	Si-B	Conversion (%)	4aa (%)[b]
1	$Et_3SiBpin$	93	91
2	$^nPr_3SiBpin$	100	95
3	$^tBuMe_2SiBpin$	100	70
4	$PhMe_2SiBpin$	50	44
5	$TMS_3SiBpin$	67	13

[a] Unless otherwise noted, reactions were conducted with **1a** (17.2 mg, 0.1 mmol), **3a** (32 μL, 0.3 mmol), silylboronates **(Si-B**, 0.2 mmol), KO*t*Bu (44.8 mg, 0.4 mmol) and triglyme (0.5 mL) at room temperature for 24 h.

[b] Determined by ^{19}F NMR and ^1H NMR spectroscopy using 3-fluoropyridine as an internal standard.

2.5 Supplementary Table 5. Variations from the optimal reaction conditions[a]

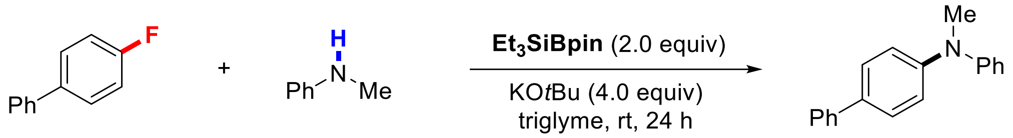

1a **3a** (3.0 equiv) "***Standard Condition***" **4aa**

Entry	Variations from "***Standard Condition***"	**4aa** (%)[b]
**1**[c]	none	91(88)
**2**	Without $Et_3SiBpin$	0
**3**	Without KO*t*Bu	0
**4**	KOMe instead of KO*t*Bu	42
**5**	NaO*t*Bu, LiO*t*Bu or KHMDS instead of KO*t*Bu	<3
**6**	2.0 equiv of KO*t*Bu	7
**7**	5.0 equiv of KO*t*Bu	88
**8**	1.5 equiv of $Et_3SiBpin$ instead of 2.0 equiv	80
**9**	1.5 equiv of **3a** instead of 3.0 equiv	53
**10**	2.0 equiv of **3a** instead of 3.0 equiv	64
**11**[c]	0.2 mmol **1a** was used	93(89)

[a] Unless otherwise noted, reactions were conducted with **1a** (17.2 mg, 0.1 mmol) and triglyme (0.5 mL), indicated amount of **3a**, $Et_3SiBpin$, KO*t*Bu were used and react at room temperature for 24 h.

[b] Determined by ^{19}F NMR and ^{1}H NMR spectroscopy using 3-fluoropyridine as an internal standard.

[c] The isolated yield was shown in the parenthesis.

3. Supplementary Notes

3.1 Synthesis of substituted aryl fluorides 1

Fluoroarenes **1a, 1c, 1d**, **1e**, **1f**, **1g**, **1h**, **1i**, and **1j** were purchased from TCI or Sigma Aldrich. **1b**, **1k**, **1l**, **1m**, **1n**, **1o**, **1p**, **1q**, **1r, 1s**, **1t**, **1u**, **1v**, **1w**, **1x**, **1y**, and **1ac** were prepared according to the known methods, and typically preparation experimental procedures were described below.

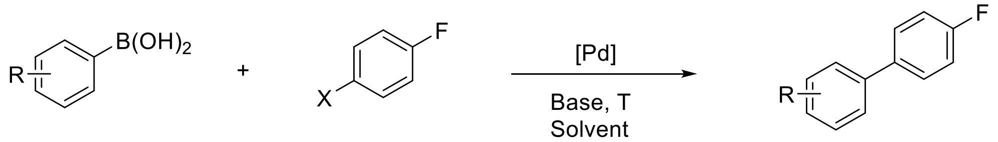

S1 **S2** **1**

3-Fluoro-1,1'-biphenyl (1b)

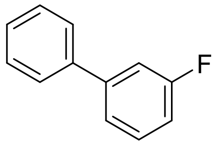

A dried flask was charged with phenylboronic acid (**S1a**, 670 mg, 5.5 mmol), tetrakis(triphenylphosphine)palladium(0) ($Pd(PPh_3)_4$, 288 mg, 0.25 mmol) and 1,4-dioxane (30 mL). The mixture was stirred at room temperature for 20 mins, then 1-fluoro-3-iodobenzene (**S2a**, 1.11 g, 5.0 mmol), saturated aqueous sodium bicarbonate ($NaHCO_3$, 7 mL, 1.4 mL/mmol) was added successively. Then the mixture was heated at 110 ℃ and stirring for 12 h. The mixture was cooled to room temperature. To this was added water (10 mL), and then extracted with ethyl acetate (EtOAc). The combined organic phase was washed with brine and dried over Na_2SO_4. After filtration, the filtrate was concentrated under reduced pressure. The residue was purified by column chromatography on silica gel (*n*-hexane) to give title compound **1b** as a white solid (857.0 mg, quantitative).

^{1}H NMR (300 MHz, $CDCl_3$) δ 7.59 – 7.55 (m, 2H), 7.47 – 7.41 (m, 2H), 7.40 – 7.34 (m, 3H), 7.31 – 7.24 (m, 1H), 7.07 – 7.01 (m, 1H).

^{19}F NMR (282 MHz, $CDCl_3$) δ -113.68 (m, 1F).

MS(EI): *m/z* 172 $[M]^+$. The chemical shifts were consistent with those reported in the literature.[1]

1-(4-Fluorophenyl)naphthalene (1k)

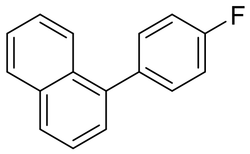

A dried flask was charged with 1-naphthaleneboronic acid (**S1b**, 2.84 g, 16.5 mmol), $Pd(PPh_3)_4$ (173 mg, 0.15 mmol) and 1,4-dioxane (60 mL). The mixture was allowed to stir at room temperature for 20 min, then 4-bromofluorobenzene (**S2b**, 1.6 mL, 15.0 mmol) and saturated $NaHCO_3$ solution (21 mL, 1.4 mL/mmol) were added subsequently. The mixture was stirred at 105 ^{o}C and keep stirring overnight before the mixture was cooled to room temperature. Evaporate to remove volatile solvent under reduced pressure, then to this mixture was added water (20 mL), and then extracted with EtOAc. The combined organic phases were washed with brine and dried over Na_2SO_4. After filtration, the filtrate was concentrated under reduced pressure. The residue was purified by column chromatography on silica gel (*n*-hexane) to give the title compound **1k** as a white solid (2.80 g, 84% yield).

^{1}H NMR (300 MHz, $CDCl_3$) δ 7.98 – 7.80 (m, 3H), 7.59 – 7.35 (m, 6H), 7.26 – 7.13 (m, 2H).

^{19}F NMR (282 MHz, $CDCl_3$) δ -116.00 (tt, J = 9.2, 5.4 Hz, 1F).

MS(EI): *m/z* 222 $[M]^+$. The chemical shifts were consistent with those reported in the literature.[2]

4-Fluoro-4'-methyl-1,1'-biphenyl (1l)

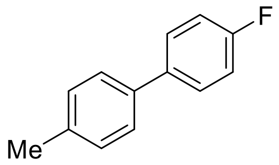

A dried flask was charged with 4-bromofluorobenzene **S2b** (0.55 mL, 5.0 mmol), 4-methylphenylboronic acid (**S1c**, 0.82 g, 6.0 mmol), $Pd(PPh_3)_4$ (174 mg, 0.15 mmol), and aqueous sodium carbonate (Na_2CO_3, 10 mL, 2 N) in toluene/EtOH (7.5 mL, *v*/*v* = 1/1). The mixture was stirred at 80 ^{o}C overnight, and the reaction progress was monitored by TLC. To this mixture was added water (10 mL), and then extracted with EtOAc. The combined organic phases were washed with brine and dried over Na_2SO_4. After filtration, the filtrate was concentrated under reduced pressure. The residue was purified by column chromatography on silica gel (*n*-hexane) to give title compound **1l** as a white solid (1.04 g, 99% yield).

^{1}H NMR (300 MHz, $CDCl_3$) δ 7.61 – 7.46 (m, 2H), 7.49 – 7.38 (m, 2H), 7.24 (d, *J* = 7.8 Hz, 2H), 7.10 (t, *J* = 8.7 Hz, 2H), 2.39 (s, 3H).

^{19}F NMR (282 MHz, $CDCl_3$) δ -116.81 (tt, *J* = 9.0, 5.3 Hz, 1F).

MS(EI): *m/z* 186 $[M]^+$. The chemical shifts were consistent with those reported in the literature.[1]

4-Fluoro-4'-methoxy-1,1'-biphenyl (1m)

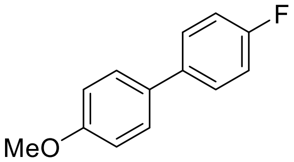

A dried flask was charged with 4-bromofluorobenzene **S2b** (0.63 mL, 5.0 mmol), 4-methoxyphenylboronic acid (**S1d**, 1.05 g, 7.5 mmol), palladium(II) dichloride ($PdCl_2$, 4.4 mg, 0.025 mmol) and potassium carbonate (K_2CO_3, 1.38 g, 10.0 mmol) in EtOH/H_2O (40 mL, *v*/*v* = 1/1). The mixture was allowed to stir at room temperature until the reaction fully completed. To this mixture was added water (10 mL), and then extracted with EtOAc. The combined organic phases were washed with brine and dried over Na_2SO_4. After filtration, the filtrate was concentrated under reduced pressure. The residue was purified by column chromatography on silica gel (*n*-hexane/EtOAc: 20/1) to give the title compound **1m** as a white solid (0.61 g, 60% yield).

^{1}H NMR (300 MHz, $CDCl_3$) δ 7.59 – 7.41 (m, 4H), 7.19 – 7.05 (m, 2H), 7.05 – 6.92 (m, 2H), 3.86 (s, 3H).

^{19}F NMR (282 MHz, $CDCl_3$) δ -117.23 (tt, *J* = 8.8, 4.9 Hz, 1F).

MS(EI): *m/z* 202 $[M]^+$. The chemical shifts were consistent with those reported in the literature.[1]

4-(Benzyloxy)-4'-fluoro-1,1'-biphenyl (1n)

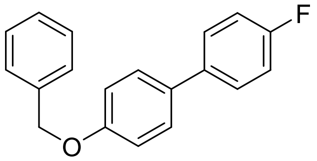

A dried flask was charged with 4-fluoroiodobenzene (**S2c**, 0.55 mL, 5.0 mmol), 4-benzyloxyphenylboronic acid (**S1e**, 1.37 g, 6.0 mmol), bis(diphenylphosphino)ferrocene]palladium(II) dichloride (Pd(dppf)Cl_2, 81.7 mg, 0.1 mmol) and

N,N-diisopropylethylamine (2.55 mL, 15.0 mmol) in *i*PrOH/H_2O (30 mL, *v*/*v* = 2/1). The mixture was stirred at 100 °C and the reaction progress was monitored by TLC. After stirring for 20 h, the mixture was cooled to room temperature. To this mixture was added water (5 mL), and then extracted with EtOAc. The combined organic phases were washed with brine and dried over Na_2SO_4. After filtration, the filtrate was concentrated under reduced pressure. The residue was purified by column chromatography on silica gel (*n*-hexane) to give title compound **1n** as a white solid (1.02 g, 73% yield).

^{1}H NMR (300 MHz, $CDCl_3$) δ 7.63 – 7.30 (m, 9H), 7.17 – 6.92 (m, 4H), 5.11 (s, 2H).

^{19}F NMR (282 MHz, $CDCl_3$) δ -117.18 (tq, *J* = 8.7, 4.3, 3.5 Hz, 1F).

MS(EI): *m/z* 278 $[M]^+$. The chemical shifts were consistent with those reported in the literature.[3]

4-Chloro-4'-fluoro-1,1'-biphenyl (1o)

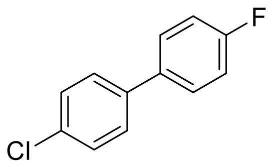

A dried flask was charged with 4-fluoroiodobenzene **S2c** (1.20 mL, 10.0 mmol), 4-chlorophenylboronic acid (**S1f**, 1.70 g, 11.0 mmol), $Pd(OAc)_2$ (23 mg, 0.1 mmol), and tripotassium phosphate (K_3PO_4, 4.25 g, 20.0 mmol) in THF/H_2O (45 mL, *v*/*v* = 2/1). The mixture was stirred at room temperature for 2 h, and the reaction progress was monitored by TLC. To this mixture was added water (10 mL), and then extracted with EtOAc. The combined organic phases were washed with brine and dried over Na_2SO_4. After filtration, the filtrate was concentrated under reduced pressure. The residue was purified by column chromatography on silica gel (*n*-hexane) to give title compound **1o** as a colorless oil (1.47 g, 71% yield).

^{1}H NMR (300 MHz, $CDCl_3$) δ 7.57 – 7.28 (m, 6H), 7.24 – 6.96 (m, 2H).

^{19}F NMR (282 MHz, $CDCl_3$) δ -115.62 (tq, *J* = 14.4, 8.8, 7.1 Hz, 1F).

MS(EI): *m/z* 206 $[M]^+$. The chemical shifts were consistent with those reported in the literature.[3]

4-Bromo-4'-fluoro-1,1'-biphenyl (1p)

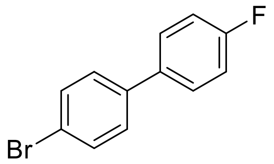

A dried flask was charged with 4-fluoroiodobenzene **S2c** (0.58 mL, 5.0 mmol), 4-bromophenylboronic acid (**S1g**, 1.30 g, 6.5 mmol), bis(triphenylphosphine)palladium(II) dichloride ($PdCl_2(PPh_3)_2$, 175 mg, 0.25 mmol) and cesium carbonate (3.26 g, 10.0 mmol) in *N*,*N*-dimethylformamide (DMF, 15 mL). The mixture was stirred at 60 °C and keep stirring overnight before the mixture was cooled to room temperature. To this mixture was added water (5 mL), and then extracted with EtOAc. The combined organic phases were washed with brine and dried over Na_2SO_4. After filtration, the filtrate was concentrated under reduced pressure. The residue was purified by column chromatography on silica gel (*n*-hexane) to afford a mixture, then purified by recrystallization form methanol to give the title compound **1p** as a white solid (0.89 g, 71% yield).

^{1}H NMR (300 MHz, $CDCl_3$) δ 7.59 – 7.46 (m, 4H), 7.44 – 7.36 (m, 2H), 7.18 – 7.08 (m, 2H).

^{19}F NMR (282 MHz, $CDCl_3$) δ -115.52 (tt, *J* = 9.1, 4.8 Hz, 1F).

MS(EI): *m/z* 251 $[M]^+$. The chemical shifts were consistent with those reported in the literature.[4]

4'-Fluoro-[1,1'-biphenyl]-4-carbonitrile (1q)

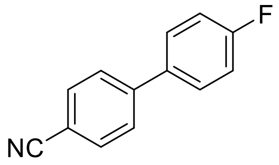

A dried flask was charged with 4-bromofluorobenzene **S2b** (0.77 mL, 7.0 mmol), (4-cyanophenyl)boronic acid (**S1h**, 1.23 g, 8.4 mmol), palladium(II) acetate ($Pd(OAc)_2$, 41.0 mg, 0.182 mmol) and triphenylphosphine (Ph_3P, 184.0 mg, 0.7 mmol) in THF/H_2O (30 mL, *v*/*v* = 5/1). The mixture was stirred at 60 °C and the reaction progress was monitored by TLC. After stirring for 16 h, the mixture was cooled to room temperature. To this mixture was added water (10 mL), and then extracted with EtOAc. The combined organic phases were washed with brine and dried over Na_2SO_4. After filtration, the filtrate was concentrated under reduced pressure. The residue was purified by

column chromatography on silica gel (*n*-hexane) to give the title compound **1q** as a white solid (0.86 g, 62% yield).

^{1}H NMR (300 MHz, $CDCl_3$) δ 7.73 (d, *J* = 8.3 Hz, 2H), 7.64 (d, *J* = 8.2 Hz, 2H), 7.60 – 7.52 (m, 2H), 7.18 (t, *J* = 8.5 Hz, 2H).

^{19}F NMR (282 MHz, $CDCl_3$) δ -113.71 (dt, *J* = 9.9, 4.2 Hz, 1F).

MS(EI): *m/z* 197 $[M]^+$. The chemical shifts were consistent with those reported in the literature.[5]

4-Fluoro-4'-(trifluoromethyl)-1,1'-biphenyl (1r)

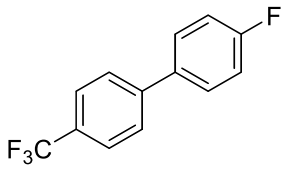

A dried flask was charged with 4-bromofluorobenzene **S2b** (1.10 mL, 10.0 mmol), 4-(trifluoromethyl)phenylboronic acid (**S1i**, 2.29 g, 12.0 mmol), $Pd(OAc)_2$ (59 mg, 0.26 mmol), Ph_3P (263 mg, 1.0 mmol) and KOH (1.12 g, 20.0 mmol) in THF/H_2O (50 mL, *v*/*v* = 5/1). The mixture was stirred at 60 °C and the reaction progress was monitored by TLC. After stirring for 16 h, the mixture was cooled to room temperature. To this mixture was added water (10 mL), and then extracted with EtOAc. The combined organic phases were washed with brine and dried over Na_2SO_4. After filtration, the filtrate was concentrated under reduced pressure. The residue was purified by column chromatography on silica gel (*n*-hexane) to give title compound **1r** as a white solid (2.19 g, 91% yield).

^{1}H NMR (300 MHz, $CDCl_3$) δ 7.72 – 7.61 (m, 4H), 7.60 – 7.52 (m, 2H), 7.22 – 7.10 (m, 2H).

^{19}F NMR (282 MHz, $CDCl_3$) δ -62.92 (s, 3F), -114.66 (dt, *J* = 8.8, 3.5 Hz, 1F).

MS(EI): *m/z* 240 $[M]^+$. The chemical shifts were consistent with those reported in the literature.[3]

4'-Fluoro-3-(trifluoromethyl)-1,1'-biphenyl (1s)

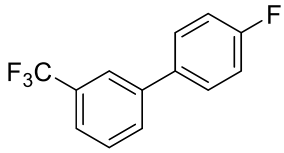

A dried flask was charged with 4-bromofluorobenzene **S2b** (1.10 mL, 10.0 mmol), 3-(trifluoromethyl)phenylboronic acid (**S1j**, 2.29 g, 12.0 mmol), $Pd(OAc)_2$ (59 mg, 0.26 mmol), Ph_3P

(263 mg, 1.0 mmol) and KOH (1.12 g, 20.0 mmol) in THF/H_2O (50 mL, *v*/*v* = 5/1). The mixture was stirred at 60 °C and the reaction progress was monitored by TLC. After stirring for 16 h, the mixture was cooled to room temperature. To this mixture was added water (10 mL), and then extracted with EtOAc. The combined organic phases were washed with brine and dried over Na_2SO_4. After filtration, the filtrate was concentrated under reduced pressure. The residue was purified by column chromatography on silica gel (*n*-hexane) to give title compound **1s** as a white solid (1.93 g, 80% yield).

^{1}H NMR (300 MHz, $CDCl_3$) δ 7.76 – 7.60 (m, 4H), 7.62 – 7.51 (m, 2H), 7.17 (t, *J* = 8.7 Hz, 2H).

^{19}F NMR (282 MHz, $CDCl_3$) δ -63.11 (s, 3F), -114.46 – -115.60 (m, 1F).

MS(EI): *m/z* 240 $[M]^+$. The chemical shifts were consistent with those reported in the literature).[3]

4'-Fluoro-3,5-bis(trifluoromethyl)-1,1'-biphenyl (1t)

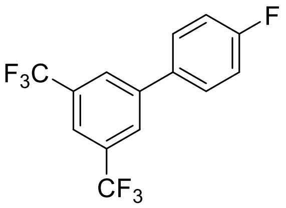

A dried flask was charged with 4-bromofluorobenzene **S2b** (0.55 mL, 5.0 mmol), 3,5-bis(trifluoromethyl)phenylboronic acid (**S1k**, 1.60 g, 6.0 mmol), $PdCl_2(PPh_3)_2$ (70.0 mg, 0.1 mmol), and K_2CO_3 (2.80 g, 20.0 mmol) in DMF/H_2O (20 mL, *v*/*v* = 4/1). The mixture was stirred overnight at 80 °C and the reaction progress was monitored by TLC. After the mixture was cooled to room temperature, to this mixture was added water (10 mL), and then extracted with EtOAc. The combined organic phases were washed with brine and dried over Na_2SO_4. After filtration, the filtrate was concentrated under reduced pressure. The residue was purified by column chromatography on silica gel (*n*-hexane) to give the title compound **1t** as a white solid (1.19 g, 77 % yield).

^{1}H NMR (300 MHz, $CDCl_3$) δ 7.97 (s, 2H), 7.86 (s, 1H), 7.59 (dd, *J* = 8.1, 5.3 Hz, 2H), 7.20 (t, *J* = 8.5 Hz, 2H).

^{19}F NMR (282 MHz, $CDCl_3$) δ -62.92 (s, 6F), -112.85 (tt, *J* = 8.5, 5.2 Hz, 1F).

MS(EI): *m/z* 308 $[M]^+$. The chemical shifts were consistent with those reported in the literature.[6]

5-(4-Fluorophenyl)benzo[*d*][1,3]dioxole (1u)

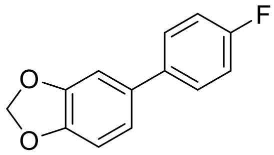

A dried flask was charged with 3,4-(methylenedioxy)phenylboronic acid (**S1l**, 1.66 g, 10.0 mmol), $Pd(PPh_3)_4$ (116 mg, 0.1 mmol) and 1,4-dioxane (56 mL). The mixture was allowed to stir at room temperature for 20 min, then 4-bromofluorobenzene **S2b** (1.2 mL, 11.0 mmol) and saturated $NaHCO_3$ (14 mL, 1.4 mL/mmol) were added subsequently. The mixture was stirred at 105 ^{o}C and keep stirring overnight before the mixture was cooled to room temperature. Evaporate to remove volatiles under reduced pressure, then to this mixture was added water (20 mL), and then extracted with EtOAc. The combined organic phases were washed with brine and dried over Na_2SO_4. After filtration, the filtrate was concentrated under reduced pressure. The residue was purified by column chromatography on silica gel (*n*-hexane/EtOAc: 20/1) to give the title compound **1u** as a white solid (1.86 g, 86% yield).

^{1}H NMR (300 MHz, $CDCl_3$) δ 7.57 – 7.36 (m, 2H), 7.15 – 7.04 (m, 2H), 7.03 – 6.96 (m, 2H), 6.87 (dd, *J* = 7.6, 0.9 Hz, 1H), 6.00 (s, 2H).

^{19}F NMR (282 MHz, $CDCl_3$) δ -116.77 (tt, *J* = 8.9, 5.3 Hz, 1F).

MS(EI): *m/z* 216 $[M]^+$. The chemical shifts were consistent with those reported in the literature.[7]

5-Fluoro-2-phenylpyridine (1v)

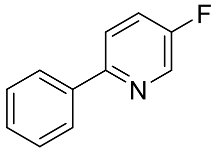

A dried flask was charged with 2-bromo-5-fluoropyridine (**S2d**, 0.88 g, 5.0 mmol), phenylboronic acid **S1a** (0.92 g, 7.5 mmol), $Pd(OAc)_2$ (56 mg, 0.25 mmol), and K_2CO_3 (1.38 g, 10.0 mmol) in $EtOH/H_2O$ (20 mL, *v*/*v* = 3/1). The mixture was stirred at 80 ^{o}C overnight, and the reaction progress was monitored by TLC. To this mixture was added water (10 mL), and then extracted with EtOAc. The combined organic phases were washed with brine and dried over Na_2SO_4. After filtration, the filtrate was concentrated under reduced pressure. The residue was purified by column chromatography on silica gel (*n*-hexane/EtOAc: 10/1) to give title compound **1v** as a white solid (0.80 g, 93% yield).

^{1}**H NMR** (300 MHz, $CDCl_3$) δ 8.55 (s, 1H), 7.94 (d, *J* = 7.4 Hz, 3H), 7.79 – 7.57 (m, 2H), 7.58 – 7.27 (m, 5H).

^{19}**F NMR** (282 MHz, $CDCl_3$) δ -130.26 (t, *J* = 5.8 Hz, 1F).

MS(EI): *m/z* 173 $[M]^+$. The chemical shifts were consistent with those reported in the literature.[3]

4-(4-Fluorophenyl)pyridine (1w)[2]

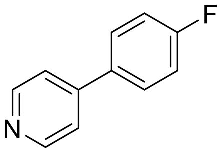

A dried flask was charged with 4-iodopyridine (**S2e**, 410 mg, 2.0 mmol), 4-fluorophenylboronic acid (**S1m**, 0.36 g, 2.6 mmol), $Pd(PPh_3)_4$ (70 mg, 0.06 mmol), and Na_2CO_3 (1.59 g, 15.0 mmol) in toluene/H_2O/EtOH (15.5 mL, *v/v/v* = 7/7/1.5). The mixture was stirred at 70 ^{o}C for 6 h, and the reaction progress was monitored by TLC. To this mixture was added water (10 mL), and then extracted with EtOAc. The combined organic phases were washed with brine and dried over Na_2SO_4. After filtration, the filtrate was concentrated under reduced pressure. The residue was purified by column chromatography on silica gel (*n*-hexane/EtOAc: 1/1) to give title compound **1w** as a white solid (0.72 g, 65% yield).

^{1}**H NMR** (300 MHz, $CDCl_3$) δ 8.64 (d, *J* = 5.3 Hz, 1H), 7.60 (dd, *J* = 8.5, 5.2 Hz, 1H), 7.45 (d, *J* = 5.4 Hz, 1H), 7.16 (t, *J* = 8.4 Hz, 1H).

^{19}**F NMR** (282 MHz, $CDCl_3$) δ -113.07 (dq, *J* = 13.4, 5.8 Hz, 1F).

MS(EI): *m/z* 173 $[M]^+$. The chemical shifts were consistent with those reported in the literature.[3]

4-Fluoro-2-phenylpyridine (1x)

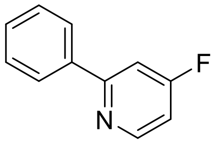

A dried flask was charged with phenylboronic acid **S1a** (670 mg, 5.5 mmol), $Pd(PPh_3)_4$ (288 mg, 0.25 mmol) and 1,4-dioxane (30 mL). The mixture was stirred at room temperature for 20 mins, then 2-bromo-4-fluoropyridine (**S2f**, 522 μL, 5.0 mmol), saturated aqueous $NaHCO_3$ (7 mL, 1.4 mL/mmol) was added successively. Then the mixture was heated at 110 °C, After stirring for 12 h, the mixture was cooled to room temperature. To this mixture was added water (10 mL), and then

extracted with EtOAc. The combined organic phase was washed with brine and dried over Na_2SO_4. After filtration, the filtrate was concentrated under reduced pressure. The residue was purified by column chromatography on silica gel (*n*-hexane/EtOAc: 20/1) to give title compound **1x** as a white solid (843.0 mg, 97% yield).

^{1}H NMR (300 MHz, $CDCl_3$) δ 8.65 (dd, *J* = 8.9, 5.6 Hz, 1H), 8.03 – 7.86 (m, 2H), 7.57 – 7.34 (m, 4H), 7.05 – 6.85 (m, 1H).

^{19}F NMR (282 MHz, $CDCl_3$) δ -103.01 (m, 1F).

MS(EI): *m/z* 173 $[M]^+$. The chemical shifts were consistent with those reported in the literature.[1]

2-Fluoro-5-phenylpyridine (1y)

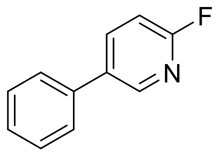

A dried flask was charged with phenylboronic acid **S1a** (670 mg, 5.5 mmol), $Pd(PPh_3)_4$ (288 mg, 0.25 mmol) and 1,4-dioxane (30 mL). The mixture was stirred at room temperature for 20 mins, then 5-bromo-2-fluoropyridine (**S2g**, 522 μL, 5.0 mmol), saturated aqueous $NaHCO_3$ (7 mL, 1.4 mL/mmol) was added successively. Then the mixture was heated at 110 °C, After stirring for 12 h, the mixture was cooled to room temperature. To this was added water (10 mL), and then extracted with EtOAc. The combined organic phase was washed with brine (20 mL) and dried over Na_2SO_4. After filtration, the filtrate was concentrated under reduced pressure. The residue was purified by column chromatography on silica gel (*n*-hexane/EtOAc: 20/1) to give title compound **1y** as a colorless oil (835.0 mg, 96% yield).

^{1}H NMR (300 MHz, $CDCl_3$) δ 8.42 (d, *J* = 3.6 Hz, 1H), 8.00 – 7.94 (m, 1H), 7.56 – 7.38 (m, 5H), 7.00 (dd, *J* = 8.5, 3.0 Hz, 1H).

^{19}F NMR (282 MHz, $CDCl_3$) δ -71.13 (s, 1F).

MS(EI): *m/z* 173 $[M]^+$. The chemical shifts were consistent with those reported in the literature.[3]

2-(4-Fluorophenyl)benzofuran (1ac)

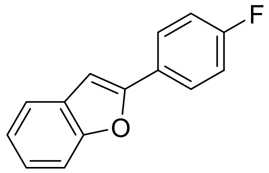

A dried flask was charged with benzofuran-2-boronic acid (**S1n**, 0.61 g, 3.75 mmol), 1-fluoro-4-iodobenzene **S2c** (0.40 mL, 3.41 mmol), $Pd(OAc)_2$ (76.6 mg, 0.34 mmol), Na_2CO_3 (0.72 g, 6.82 mmol) and acetone/H_2O (44 mL, *v*/*v* = 1/1.2). The mixture was stirred at room temperature for 12 h, the mixture was quenched with water (10 mL), then extracted with EtOAc. The combined organic phase was washed with brine and dried over Na_2SO_4. After filtration, the filtrate was concentrated under reduced pressure. The residue was purified by column chromatography on silica gel (*n*-hexane/EtOAc: 20/1) to give title compound **1ac** as a white solid (459 mg, 64% yield).

^{1}H NMR (300 MHz, $CDCl_3$) δ 8.00 – 7.71 (m, 2H), 7.73 – 7.41 (m, 2H), 7.38 – 7.20 (m, 2H), 7.20 – 7.05 (m, 2H), 6.98 (s, 1H).

^{19}F NMR (282 MHz, $CDCl_3$) δ -112.89 (q, *J* = 7.6, 6.7 Hz, 1F).

MS(EI): *m/z* 212 $[M]^+$. The chemical shifts were consistent with those reported in the literature.[8]

A typically preparation experimental procedures for **1z**, **1aa**, **1ab**, **1ad**, **1ae**, **1af**, and **1ag** were described below.

1-(4-Fluorophenyl)-1*H*-pyrrole (1z)

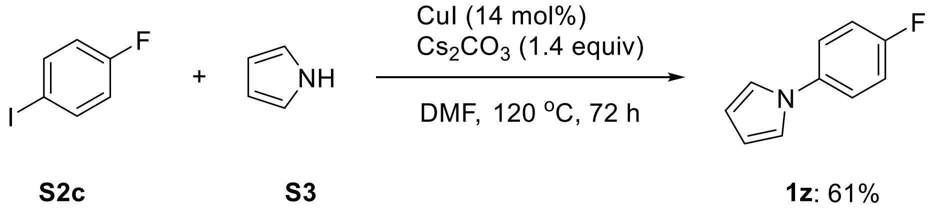

A dried flask was charged with copper(I) iodide (213.3 mg, 1.12 mmol), cesium carbonate (3.65 g, 11.2 mmol), then anhydrous DMF (10 mL) was added to the flask which protected by nitrogen atmosphere. Then 4-fluoroiodobenzene **S2c** (1.30 mL, 11.2 mmol), pyrrole (**S3**, 0.56 mL, 8.0 mmol) was added to the resulting mixture, and stirred at 120 °C for 72 h in an oil bath. After check TLC, the reaction was filtered through a short of celite®, washed with EtOAc and extracted with EtOAc

and water. The combined organic extract was washed with brine and dried over Na_2SO_4. After filtration, the filtrate was concentrated under reduced pressure to give the residue. Which was purified by column chromatography on silica gel (*n*-hexane) to give title compound **1z** as a white solid (789 mg, 61% yield).

^{1}H NMR (300 MHz, $CDCl_3$) δ 7.43 – 7.30 (m, 2H), 7.19 – 7.06 (m, 2H), 7.07 – 6.98 (m, 2H), 6.36 (q, J = 2.1 Hz, 2H).

^{19}F NMR (282 MHz, $CDCl_3$) δ -117.65 (td, J = 7.7, 4.1 Hz, 1F).

MS(EI): *m/z* 161 $[M]^+$. The chemical shifts were consistent with those reported in the literature.[3]

6-Fluoro-1-methyl-1*H*-indole (1aa)

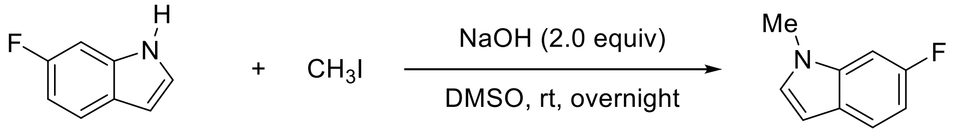

S4 **S5** **1aa**: 84%

To a solution of 6-fluoroindole (**S4**, 0.675 g, 5.0 mmol) in DMSO (20 mL) was added NaOH (0.40 g, 10.0 mmol) at room temperature. After stirring for 30 min, then iodomethane (**S5**, 0.62 mL, 10.0 mmol) was added, and the reaction was then stirred at room temperature overnight. After check TLC, the reaction was quenched with saturated NH_4Cl (10 mL) and extracted with Et_2O. The combined organic extract was washed with brine and dried over Na_2SO_4. After filtration, the filtrate was concentrated under reduced pressure. The crude reaction mixture was purified by column chromatography on silica gel (*n*-hexane/DCM: 50/1) to give title compound **1aa** as a colorless oil (0.63 g, 84% yield).

^{1}H NMR (300 MHz, $CDCl_3$) δ 7.79 – 7.44 (m, 1H), 7.16 – 6.82 (m, 3H), 6.66 – 6.38 (m, 1H), 3.74 (s, 3H).

^{19}F NMR (282 MHz, $CDCl_3$) δ -121.59 (td, J = 9.8, 5.3 Hz, 1F).

MS(EI): *m/z* 149 $[M]^+$. The chemical shifts were consistent with those reported in the literature.[3]

2-(4-Fluorophenyl)-1-methyl-1*H*-indole (1ab)

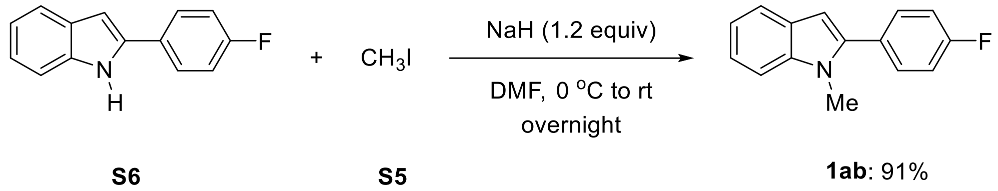

To a solution of 2-(4-fluorophenyl)-1*H*-indole (**S6**, 1.05 g, 5.0 mmol) in DMF (10 mL) was added NaH (0.24 g, 6.0 mmol, 60% in mineral oil) at 0 °C. After stirring for 30 min at 0 °C, then iodomethane **S5** (374 μL, 6.0 mmol) was added and the reaction was then stirred at room temperature overnight. After check TLC, the reaction was quenched with saturated NH_4Cl (10 mL) and extracted with Et_2O. The combined organic extract was washed with brine and dried over Na_2SO_4. After filtration, the filtrate was concentrated under reduced pressure. The crude reaction mixture was purified by recrystallization from Et_2O/*n*-hexane to give title compound **1ab** as a light-yellow solid (1.03 g, 91% yield).

^{1}H NMR (300 MHz, $CDCl_3$) δ 7.63 (d, *J* = 7.8 Hz, 1H), 7.50 – 7.45 (m, 2H), 7.36 (d, *J* = 8.2 Hz, 1H), 7.28 – 7.23 (m, 1H), 7.19 – 7.12 (m, 3H), 6.53 (s, 1H), 3.72 (s, 3H).

^{19}F NMR (282 MHz, $CDCl_3$) δ -114.38 (m, 1F).

MS(EI): *m/z* 225 $[M]^+$. The chemical shifts were consistent with those reported in the literature.[9]

(*R*)-6-((4-Fluorobenzyl)oxy)-2,5,7,8-tetramethyl-2-((4*R*,8*R*)-4,8,12-trimethyltridecyl)chromane (1ad)

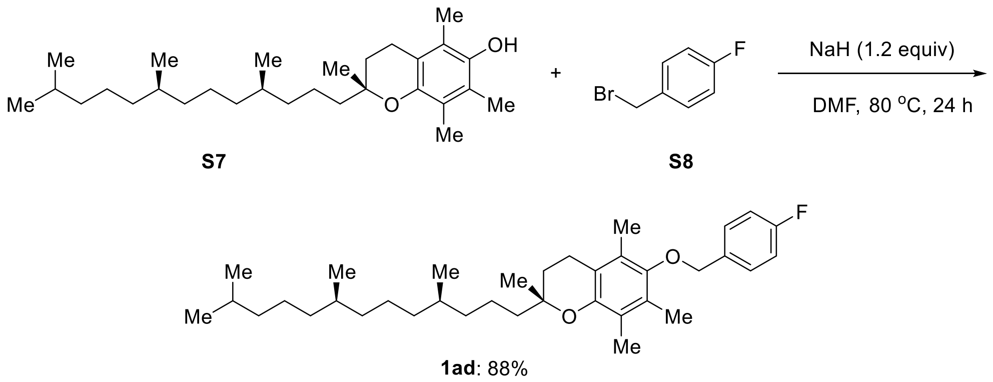

To a solution of (±)-α-Tocopherol (**S7**, 2.74 g, 6.36 mmol) in DMF (20 mL) was added NaH (0.31 g, 7.63 mmol, 60% in mineral oil) at room temperature. After stirring for 30 min, then 4-fluorobenzyl bromide (**S8**, 1.32 g, 7.0 mmol) was added, and the reaction was then stirred at 80 °C for 24h. After check TLC, the reaction was quenched with saturated NH_4Cl (5 mL) and extracted

with EtOAc. The combined organic extract was washed with brine and dried over Na_2SO_4. After filtration, the filtrate was concentrated under reduced pressure. The crude reaction mixture was purified by column chromatography on silica gel (*n*-hexane/EtOAc: 100/1) to give title compound **1ad** as a colorless oil (3.02 g, 88% yield).

^{1}H NMR (300 MHz, $CDCl_3$) δ 7.56 – 7.36 (m, 2H), 7.17 – 6.93 (m, 2H), 4.67 (s, 2H), 2.61 (t, *J* = 7.0 Hz, 2H), 2.32 – 1.99 (m, 9H), 1.97 – 1.66 (m, 2H), 1.65 – 1.49 (m, 3H), 1.49 – 1.34 (m, 4H), 1.34 – 1.22 (m, 12H), 1.23 – 1.02 (m, 7H), 0.95 – 0.82 (m, 12H).

^{19}F NMR (282 MHz, $CDCl_3$) δ -115.09 (t, *J* = 7.6 Hz, 1F).

MS(ESI): *m/z* 561 $[M+Na]^+$. The chemical shifts were consistent with those reported in the literature.[3]

(8*R*,9*S*,13*S*,14*S*)-3-Fluoro-17-methoxy-13-methyl-7,8,9,11,12,13,14,15,16,17-decahydro-6*H*-cyclopenta[*a*]phenanthrene (1ae)

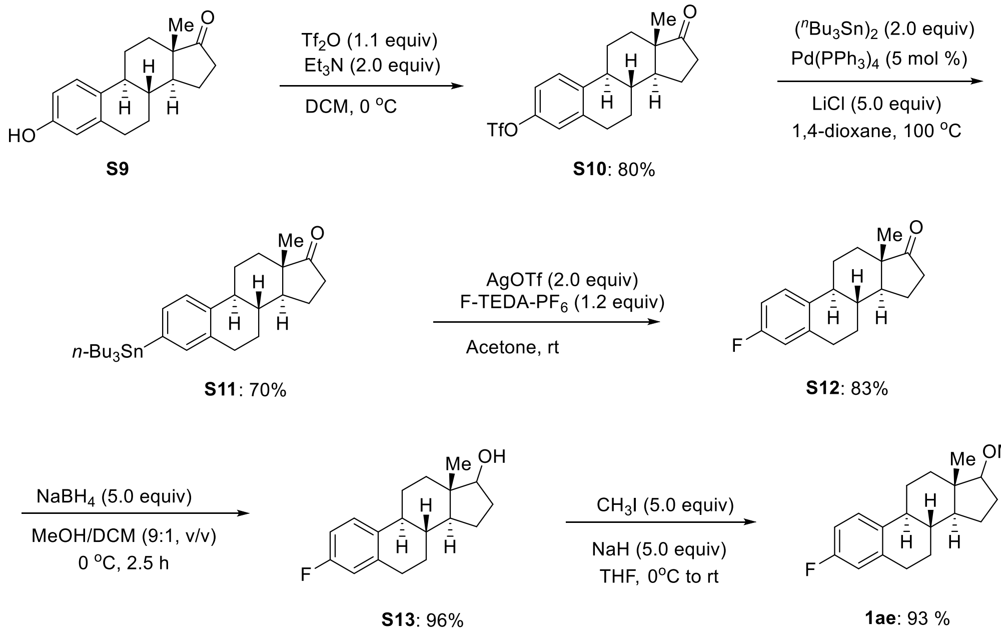

Synthetic intermediates were prepared according to procedures previously described.[10]

In a 200 mL dried flask, a solution of estrone (**S9**, 2.70 g, 10.0 mmol) in anhydrous DCM (50 mL) was added triethylamine (Et_3N, 2.8 mL, 20.0 mmol) at 0 ºC under a N_2 atmosphere, then trifluoromethanesulfonic anhydride (1.85 mL, 11.0 mmol) was added dropwise. The reaction mixture was stirred at 0 ºC for 30 min and then quenched by the adding saturated aqueous $NaHCO_3$ (50 mL). The reaction mixture was extracted with DCM, and then washed with brine and dried over

Na_2SO_4. After filtration, the filtrate was concentrated in vacuo and then the residue was purified by chromatography on silica gel (*n*-hexane/EtOAc: 5/1) to afford triflate intermediate **S10** as a colorless oil (3.21 g, 80% yield).

In a N_2-filled glovebox, to a solution of previously obtained triflate intermediate (**S10**, 2.01 g, 5.0 mmol) in dioxane (50 mL) was added lithium chloride (1.06 g, 25.0 mmol), $Pd(PPh_3)_4$ (290 mg, 0.25 mmol) and bis(tri-*n*-butyltin) (5.0 mL, 10.0 mmol) subsequently. After sealed and moved out from glovebox, the reaction mixture was allowed to stir under 100 ºC in an oil bath for 14 h. The reaction mixture was cooled down to room temperature once the reaction was finished and concentrated in vacuo to remove the volatiles. The residue was purified by chromatography on silica gel (*n*-hexane/EtOAc: 25/1) to afford organotin intermediate **S11** as a colorless oil (1.90 g, 70% yield).

In a N_2-filled glovebox, to a solution of organotin intermediate (**S11**, 1.88 g, 3.46 mmol) in anhydrous acetone (65 mL) was added silver triflate (1.80 g, 6.92 mmol) and 1-chloromethyl-4-fluoro-1,4-diazoniabicyclo[2.2.2]octanebis(trifluoroborate) (F-TEDA-PF_6, 1.96 g, 4.16 mmol). The reaction mixture was stirred for 30 min at room temperature and then moved out from glovebox. Then filtered through a short pad of silica, the filtrate was concentrated in vacuo to afford the residue. Which was purified by column chromatography on silica gel (*n*-hexane) to give fluorinated estrone **S12** as a white solid (0.78 g, 83% yield).

To a solution of fluorinated estrone (**S12**, 0.74 g, 2.72 mmol) in MeOH/DCM (10 mL, *v*/*v* = 9/1) under 0 ºC was added sodium borohydride ($NaBH_4$, 0.52 g, 13.6 mmol) portion-wise. The flask keeps stirring for 5 h under same temperature and equipped with a N_2 balloon to release the pressure during the reaction progress. After the reaction completed, slowly quenched with water (5 mL) and extracted with Et_2O. The combined organic phases were washed with brine, dried over Na_2SO_4. After filtered and concentrated in vacuo to afford the residue. Which was purified by column chromatography on silica gel (*n*-hexane/EtOAc: 10/1) to give alcohol intermediate **S13** as a white solid (0.72 g, 96% yield).

To a solution of alcohol intermediate (**S13**, 720 mg, 2.63 mmol) in dry THF (10 mL) under 0 ºC was added NaH (320 mg, 13.2 mmol, 60% in mineral oil). After stirring 30 min, iodomethane **S5** (0.82 mL, 13.2 mmol) was then added slowly. The mixture was stirred at room temperature for 2 h, and the reaction progress was monitored by TLC. To this mixture was added water (10 mL), and

then extracted with EtOAc. The combined organic phases were washed with brine and dried over Na_2SO_4. After filtration, the filtrate was concentrated under reduced pressure. The residue was purified by column chromatography on silica gel (*n*-hexane) to give title compound **1ae** as a white solid (706 mg, 93% yield).

^{1}H NMR (300 MHz, $CDCl_3$) δ 7.28 – 7.15 (m, 1H), 6.90 – 6.67 (m, 2H), 3.38 (s, 3H), 3.31 (t, *J* = 8.3 Hz, 1H), 2.84 (dd, *J* = 7.5, 3.3 Hz, 2H), 2.33 – 2.24 (m, 1H), 2.18 (dd, *J* = 19.5, 9.1 Hz, 1H), 2.12 – 2.00 (m, 2H), 1.93 – 1.83 (m, 1H), 1.76 – 1.63 (m, 1H), 1.56 – 1.30 (m, 6H), 1.26 – 1.14 (m, 1H), 0.79 (s, 3H).

^{19}F NMR (282 MHz, $CDCl_3$) δ -119.10 (q, *J* = 8.4 Hz, 1F).

MS(EI): *m/z* 288 $[M]^+$. The chemical shifts were consistent with those reported in the literature.[3]

1-Fluoro-4-((((2*S*,5*R*)-2-isopropyl-5-methylcyclohexyl)oxy)methyl)benzene (1af)

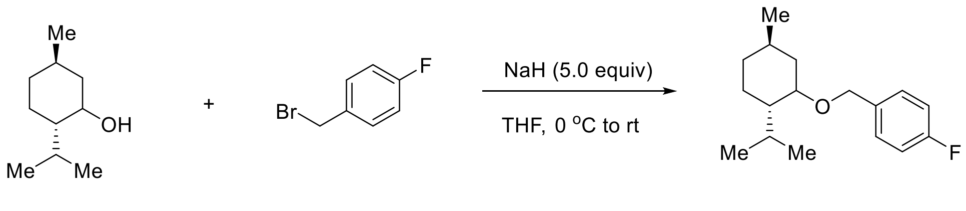

To a solution of menthol (**S14**, 0.78 g, 5.0 mmol) in THF (20 mL) was added NaH (1.0 g, 25.0 mmol, 60% in mineral oil) at 0 °C. After stirring for 30 min, then 4-fluorobenzyl bromide **S8** (1.23 mL, 10.0 mmol) was added at 0 °C and the reaction was then stirred at room temperature overnight. After check TLC, the reaction was quenched with saturated NH_4Cl (5 mL) and extracted with Et_2O. The combined organic extract was washed with brine and dried over Na_2SO_4. After filtration, the filtrate was concentrated under reduced pressure. The crude reaction mixture was purified by column chromatography on silica gel (*n*-hexane/DCM: 100/1) to give title compound **1af** as colorless oil (1.07 g, 81% yield).

^{1}H NMR (300 MHz, $CDCl_3$) δ 7.37 – 7.25 (m, 2H), 7.11 – 6.85 (m, 2H), 4.62 (d, *J* = 11.3 Hz, 1H), 4.35 (d, *J* = 11.3 Hz, 1H), 3.16 (td, *J* = 10.5, 4.1 Hz, 1H), 2.41 – 2.08 (m, 2H), 1.75 – 1.56 (m, 2H), 1.42 – 1.20 (m, 2H), 1.02 – 0.80 (m, 9H), 0.70 (d, *J* = 7.0 Hz, 3H).

^{19}F NMR (282 MHz, $CDCl_3$) δ -115.84 (tt, *J* = 9.3, 5.4 Hz, 1F).

MS (EI): *m/z* 264 $[M]^+$. The chemical shifts were consistent with those reported in the literature.[3]

4-Fluorophenyl (*R*)-2-(6-methoxynaphthalen-2-yl)propanoate (1ag)

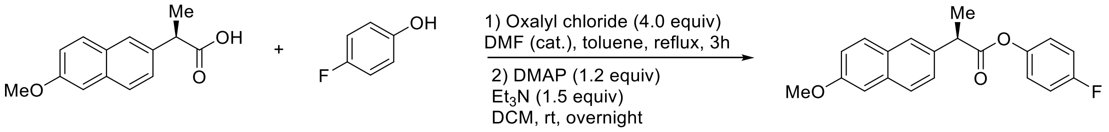

S15 **S16** **1ag**: 91%

To a solution of (*S*)-(+)-2-(6-methoxy-2-naphthyl)propionic acid (**S15**, 1.20 g, 5.0 mmol) in 15 mL of anhydrous toluene was added oxalyl chloride (1.7 mL, 20.0 mmol) and 3 drops of DMF under room temperature. The reaction mixture was stirred and reflux overnight under N_2. The solvent was removed under reduced pressure to afford the corresponding acid chloride. To a dried flask was charged with 4-fluorophenol (**S16**, 0.45 g, 4.0 mmol), DMAP (49.0 mg, 0.4 mmol), then a solution of previous acid chloride in DCM was injected and followed by Et_3N (0.84 mL, 6.0 mmol), the resulted solution was stirred overnight at room temperature. After the reaction finished, to this mixture was added water (40 mL), and then extracted with DCM. The combined organic phases were washed with brine and dried over Na_2SO_4. After filtration, the filtrate was concentrated under reduced pressure. The residue was purified by column chromatography on silica gel (*n*-hexane/EtOAc: 10/1) to give title compound **1ag** as a white solid (1.18 g, 91% yield), m.p. = 79.3 – 80.1 ^{o}C.

^{1}H NMR (300 MHz, $CDCl_3$) δ 7.81 – 7.67 (m, 3H), 7.50 (d, *J* = 8.4 Hz, 1H), 7.23 – 7.10 (m, 2H), 7.07 – 6.88 (m, 4H), 4.09 (q, *J* = 7.1 Hz, 1H), 3.93 (s, 3H), 1.69 (d, *J* = 7.2 Hz, 3H).

^{13}C NMR (75 MHz, $CDCl_3$) δ 173.4, 160.3 (d, *J* = 244.2 Hz), 157.9, 146.8, 135.1, 134.0, 129.5, 129.1, 127.6, 126.2, 122.9, 119.3, 116.2, 115.9, 105.7, 55.5, 45.6, 18.6.

^{19}F NMR (282 MHz, $CDCl_3$) δ -117.58 (tt, *J* = 8.2, 4.5 Hz, 1F).

IR (KBr): 3079, 3018, 2986, 2959, 2939, 1747, 1631, 1603, 1502, 1457, 1393, 1378, 1332, 1266, 1216, 1118, 1093, 1081, 1027, 894, 820, 767, 661 cm^{-1}.

HRMS (ESI) [$C_{20}H_{17}FO_3Na$] $[M+Na]^+$ calculated: 347.1059, found: 347.1057.

3.2 Synthesis of substituted alkyl fluorides 2, allyl fluoride, and cinnamyl fluoride

Alkyl fluorides **2a**, **2b**, **2c**, and **2e** were prepared according to the known procedures.[1]

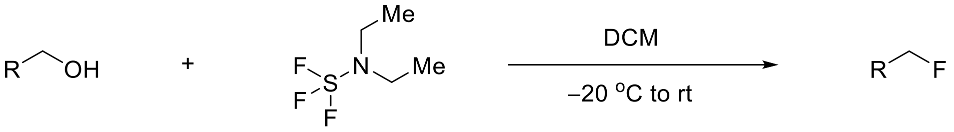

S17 DAST **2**

General procedure A: To the solution of alcohol (**S17**, 1.0 equiv) in the DCM (0.4 M) was added *N*,*N*-diethylaminosulfur trifluoride (DAST, 1.1 equiv) at –20 °C dropwise. Then the mixture was moved to room temperature and stirred for 6 h. After that, the reaction mixture was poured into cooled water, quenched with saturated $NaHCO_3$ solution, and extracted with DCM. The combined organic phase was dried over Na_2SO_4, filtered, and concentrated under reduced pressure. The crude was purified by column chromatography on silica gel (*n*-hexane) to give alkyl fluorides **2**.

4-(Fluoromethyl)biphenyl (2a)

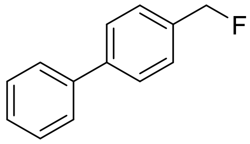

Compound **2a** was synthesized from DAST (1.45 mL, 11.0 mmol) and biphenyl-4-methanol (**S17a**, 1.84 g, 10.0 mmol) by using **General procedure A**. And title compound was obtained as a white solid (1.63 g, 88% yield), which packed into a few sample bottles and stored in refrigerator (-20 °C).

^{1}H NMR (300 MHz, $CDCl_3$) δ 7.69 – 7.55 (m, 4H), 7.52 – 7.40 (m, 4H), 7.41 – 7.33 (m, 1H), 5.43 (d, *J* = 47.9 Hz, 2H).

^{19}F NMR (282 MHz, $CDCl_3$) δ -206.63 (t, *J* = 47.8 Hz, 1F).

MS(EI): *m/z* 186 $[M]^+$. The chemical shifts were consistent with those reported in the literature.[1]

1-(Fluoromethyl)-4-(trifluoromethoxy)benzene (2b)

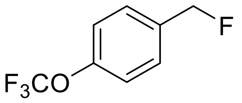

Compound **2b** was synthesized from DAST (0.73 mL, 5.5 mmol) and (4-(trifluoromethyl)phenyl)methanol (**S17b**, 0.88 g, 5.0 mmol) by using **General procedure A**. And title compound was obtained as a colorless oil (0.65 g, 67% yield), which stored in refrigerator (-20

°C).

^{1}H NMR (300 MHz, $CDCl_3$) δ 7.46 – 7.34 (m, 2H), 7.29 – 7.15 (m, 2H), 5.38 (d, J = 47.5 Hz, 2H).

^{19}F NMR (282 MHz, $CDCl_3$) δ -58.33 (s, 3F), -208.18 (t, J = 47.6 Hz, 1F).

MS(EI): m/z 194 $[M]^+$. The chemical shifts were consistent with those reported in the literature.[1]

1-(Fluoromethyl)naphthalene (2c)

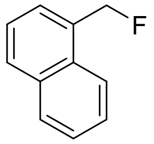

Compound **2c** was synthesized from DAST (0.73 mL, 5.5 mmol) and 1-naphthalenemethanol (**S17c**, 0.79 g, 5.0 mmol) by using **General procedure A**. And title compound was obtained as a colorless oil (0.60 g, 75% yield), which stored in refrigerator (-20 °C).

^{1}H NMR (300 MHz, $CDCl_3$) δ 8.17 – 8.01 (m, 1H), 8.02 – 7.80 (m, 2H), 7.68 – 7.37 (m, 4H), 5.87 (d, J = 47.9 Hz, 2H).

^{19}F NMR (282 MHz, $CDCl_3$) δ -206.61 (t, J = 47.9 Hz, 1F).

MS(EI): m/z 160 $[M]^+$. The chemical shifts were consistent with those reported in the literature.[1]

1-Fluorodecane (2e)

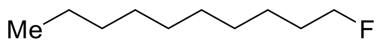

Compound **2e** was synthesized from DAST (0.73 mL, 5.5 mmol) and 1-decanol (**S17d**, 0.79 g, 5.0 mmol) by using **General procedure A**. And title compound was obtained as a colorless oil (0.64 g, 80% yield).

^{1}H NMR (300 MHz, $CDCl_3$) δ 4.43 (dt, J = 47.4, 6.2 Hz, 2H), 1.82 – 1.57 (m, 2H), 1.44 – 1.16 (m, 14H), 0.87 (t, J = 6.4 Hz, 3H).

^{19}F NMR (282 MHz, $CDCl_3$) δ -218.45 (tt, J = 47.6, 24.8 Hz, 1F).

MS(EI): m/z 160 $[M]^+$. The chemical shifts were consistent with those reported in the literature.[3]

A typical experimental procedure for the preparation of **2d**, (*E*)-(3-fluoroprop-1-en-1-yl)benzene, and (*E*)-1-fluorooct-2-ene were described below.

2-(1-Fluoroethyl)naphthalene (2d)

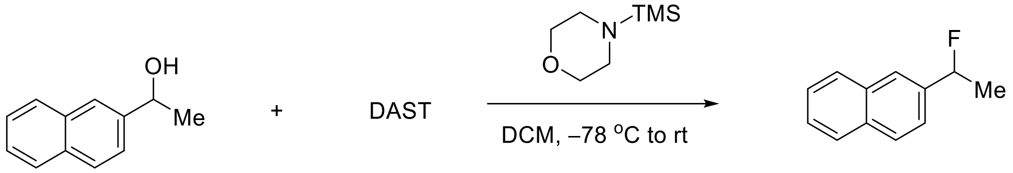

S17e **2d**: 78%

Synthetic procedure was followed to a previously reported method.[11] To a solution of diethylaminosulfur trifluoride (DAST, 1.3 mL, 9.0 mmol) in DCM (10 mL) was added trimethylsilylmorpholine (1.7 mL, 9.75 mmol) at –78 °C, and then the mixture was stirred at room temperature for 2.5 h. The mixture was returned to cooled at –78 °C before a solution of 1-(2-naphthyl)ethanol (**S17e**, 0.52 g, 3.0 mmol) in DCM (10 mL) was added dropwise. Thereafter, the reaction mixture was allowed to warm to room temperature and then stirred for 16 h. The reaction was quenched by the dropwise addition of 1.0 mL of methanol followed by saturated $NaHCO_3$, and then extracted with DCM. The combined organic phase was washed with brine, then dried over Na_2SO_4. After filtered and evaporated under reduced pressure to give the reaction crude. Which was purified by column chromatography on silica gel (*n*-pentane) to give the title compound **2d** as a white solid (0.41 g, 78% yield) and stored in a plastic tube.

^{1}H NMR (300 MHz, $CDCl_3$) δ 8.01 – 7.73 (m, 4H), 7.60 – 7.43 (m, 3H), 5.81 (dq, *J* = 47.6, 6.2 Hz, 1H), 1.85 – 1.61 (m, 3H).

^{19}F NMR (282 MHz, $CDCl_3$) δ -167.47 (dq, *J* = 47.7, 23.9 Hz, 1F).

MS(EI): *m/z* 174 $[M]^+$. The chemical shifts were consistent with those reported in the literature.[11]

(*E*)-(3-Fluoroprop-1-en-1-yl)benzene (S19)

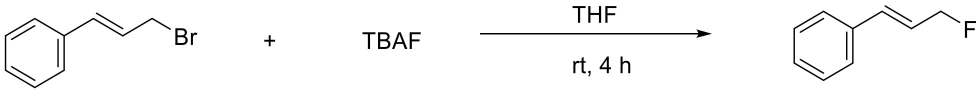

S18 **S19**: 57%

Synthetic procedure was followed to a previously reported method.[12] To a solution of (*E*)-(3-bromoprop-1-en-1-yl)benzene (**S18**, 1.0 g, 5.0 mmol) in THF (5 mL) was added tetrabutylammonium fluoride dropwise (TBAF, 10.0 mL, 10.0 mmol, 1.0 M in THF) at room

temperature, and then stirred at for 4 h. The reaction was quenched by poured into a buffer (pH = 7.0). Extracted with dimethyl ether (Et_2O) and the combined organic phase was washed with brine, then dried over Na_2SO_4. After filtered and evaporated under reduced pressure to give the reaction crude. Which was purified by column chromatography on silica gel (*n*-pentane) to give the title compound **S19** as a colorless oil (0.39 g, 57% yield).

^{1}H NMR (300 MHz, $CDCl_3$) δ 7.50 – 7.11 (m, 5H), 6.68 (dt, *J* = 15.9, 4.5 Hz, 1H), 6.48 – 6.22 (m, 1H), 5.01 (ddt, *J* = 47.0, 6.1, 2.2 Hz, 2H).

^{19}F NMR (282 MHz, $CDCl_3$) δ -210.51 (tdd, *J* = 47.0, 12.1, 5.0 Hz, 1F).

MS(EI): *m/z* 136 $[M]^+$. The chemical shifts were consistent with those reported in the literature.[12]

(*E*)-1-Fluorooct-2-ene (S23)

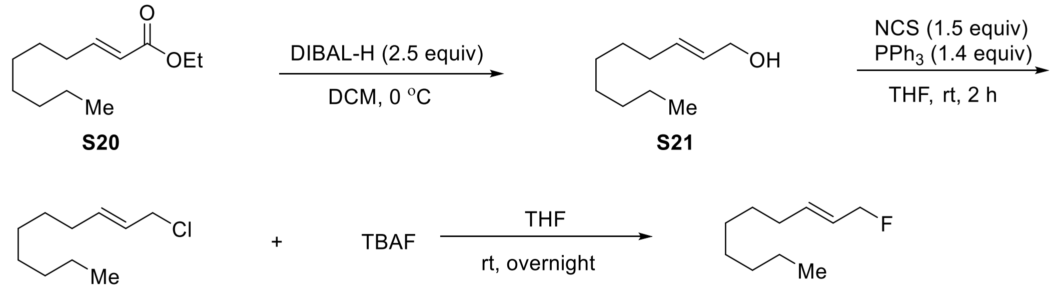

S20 **S21** **S22** **S23**: 68%

To a stirring solution of ethyl (*E*)-dec-2-enoate (**S20**, 1.0 g, 5.0 mmol) in DCM (10.0 mL) at 0 °C was added a solution of diisobutylaluminium hydride (DIBAL-H, 12.5 mL, 12.5 mmol, 1.0 M in *n*-hexane) dropwise. The reaction was allowed to warm up and stirred at room temperature for 2 hours. The reaction was then cooled to 0 °C and quenched by addition of H_2O, $NaOH/H_2O$ (15%, *w/w*), and keep stirring at room temperature until two layers formed. Then extracted with DCM and the combined organic phase was dried over Na_2SO_4. After filtered and evaporated under reduced pressure. The crude was purified by column chromatography on silica gel (*n*-hexane/EtOAc: 4/1) to give allyl alcohol **S21** as a colorless oil (0.62 g, 79% yield).

To a solution of (*E*)-dec-2-ene-1-ol (**S21**, 0.47 g, 3.0 mmol) in THF (10 mL) at room temperature, triphenylphosphine (PPh_3, 1.10 g, 4.2 mmol) was added, followed by *N*-chlorosuccinimide (NCS, 0.60 g, 7.50 mmol). The reaction mixture was allowed to stir at room temperature for 2 h. Then *n*-hexane was then added to the mixture (until precipitate formed), and the suspension was filtered through a short silica pad. The filtrate was concentrated under reduced pressure and purified by

column chromatography on silica gel (*n*-hexane) to give allyl chloride **S22** as a colorless oil (0.43 g, 82% yield).

To a solution of allyl chloride (**S22**, 174.0 mg, 1.0 mmol) in THF (5 mL) was added tetrabutylammonium fluoride dropwise (TBAF, 2.0 mL, 2.0 mmol, 1.0 M in THF) at room temperature, and then stirred overnight. The reaction was quenched by pouring into a buffer (pH = 7.0). Extracted with Et_2O and the combined organic phase was washed with brine, dried over Na_2SO_4. After filtered and evaporated under reduced pressure to give the reaction crude. Which was purified by column chromatography on silica gel (*n*-hexane) to give the title compound **S23** as a colorless oil (107 mg, 68% yield).

^{1}H NMR (300 MHz, $CDCl_3$) δ 5.75 (ddt, *J* = 44.1, 15.8, 7.5 Hz, 2H), 4.79 (dd, *J* = 47.5, 6.2 Hz, 2H), 2.07 (t, *J* = 6.8 Hz, 2H), 1.26 (s, 12H), 0.88 (t, *J* = 6.4 Hz, 3H).

^{19}F NMR (282 MHz, $CDCl_3$) δ -207.65 (t, *J* = 47.4 Hz, 1F).

MS(EI): *m/z* 158 $[M]^+$. The chemical shifts were consistent with those reported in the literature.[13]

3.3 General procedure for the synthesis of *N*-alkyl anilines 3

N-methyl anilines **3a**, **3l**, **3n**, **3o**, **3p**, **3q**, **3r**, **3s**, **3t** and **3u** were purchased from TCI or Sigma Aldrich. *N*-methyl anilines **3b**, **3c**, **3d**, **3e**, **3f**, **3g**, **3h**, **3i**, **3j**, **3k** and **3m** were prepared according to known methods. A typical experimental procedure for the preparation of *N*-methyl anilines were described below.

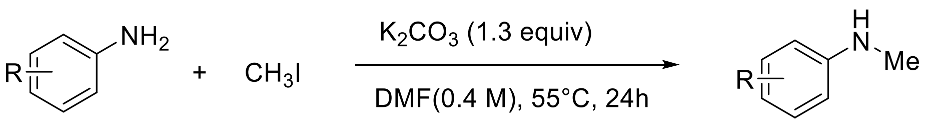

S24 **S5** **3**

General procedure B: Synthetic intermediates were prepared according to the procedures previously described.[14] In a flame-dried flask was charged with anilines (**S24**, 10.0 mmol), iodomethane **S5** (0.75 mL, 12.0 mmol), potassium carbonate (1.80 g, 13.0 mmol), and DMF (25 mL). The flask was then sealed and heated to 55 °C for 24 h. After reaction cooling, water was added into the mixture. The organic phase was separated, and the aqueous layer was extracted with

EtOAc. The combined organic phase was washed with brine and dried over Na_2SO_4. After filtered and evaporated under reduced pressure to give the reaction crude. Which was purified by column chromatography on silica gel using *n*-hexane/EtOAc as the eluent to give *N*-methyl anilines **3**. Compounds **3c**, **3e**, **3f**, **3g**, **3h**, **3j** were synthesized by using **General procedure B**.

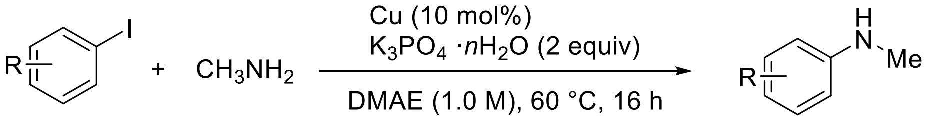

S25 **S26** **3**

General procedure C: Synthetic intermediates were prepared according to the procedures previously described.[15] In a flame-dried flask was charged with aryl iodide (**S25**, 10.0 mmol), methylamine (**S26**, 15.0 mmol, 33 wt% in absolute ethanol), Cu powder (64.0 mg, 10 mol%), $K_3PO_4 \cdot nH_2O$ (5.34 g, 20.0 mmol) in 2-(dimethylamino)ethanol (DMAE, 10 mL) was heated at 60 °C for 16 h. The reaction mixture was poured into water and extracted with EtOAc. The combined organic phase was washed with brine and dried over Na_2SO_4. After filtered and evaporated under reduced pressure to give the reaction crude. Which was purified by column chromatography on silica gel using *n*-hexane/EtOAc as the eluent to give *N*-methyl anilines **3**. Compounds **3b**, **3d**, **3i**, and **3m** were synthesized by using **General procedure C**.

N,4-Dimethylaniline (3b)

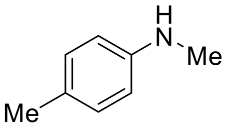

Compound **3b** was obtained from 4-iodotoluene (**S25a**) by using **General procedure C** as a light-yellow oil (0.92 g, 77% yield).

^{1}H NMR (300 MHz, $CDCl_3$) δ 7.01 (d, *J* = 8.0 Hz, 2H), 6.55 (d, *J* = 8.4 Hz, 2H), 3.56 (bs, 1H), 2.82 (s, 3H), 2.25 (s, 3H).

MS(EI): *m/z* 121 $[M]^+$. The chemical shifts were consistent with those reported in the literature.[16]

***N*,3,5-Trimethylaniline (3c)**

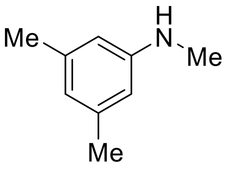

Compound **3c** was obtained from 3,5-dimethylaniline (**S24a**) by using **General procedure B** as a light-yellow oil (0.43 g, 32% yield).

^{1}H NMR (300 MHz, $CDCl_3$) δ 6.42 (s, 1H), 6.29 (s, 2H), 3.57 (brs, 1H), 2.84 (s, 3H), 2.29 (s, 6H).

MS(EI): *m/z* 135 $[M]^+$. The chemical shifts were consistent with those reported in the literature.[16]

***N*,2-Dimethylaniline (3d)**

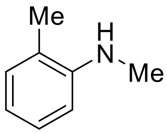

Compound **3d** was obtained from 2-iodotoluene (**S25b**) by using **General procedure C** as a light-yellow oil (0.22 g, 18% yield).

^{1}H NMR (300 MHz, $CDCl_3$) δ 7.17 (t, J = 7.7 Hz, 1H), 7.06 (d, J = 7.5 Hz, 1H), 6.67 (t, J = 8.0 Hz, 1H), 6.61 (d, J = 8.0 Hz, 1H), 3.56 (brs, 1H), 2.90 (s, 3H), 2.14 (s, 3H).

MS(EI): *m/z* 121 $[M]^+$. The chemical shifts were consistent with those reported in the literature.[16]

***N*,2,4,6-Tetramethylaniline (3e)**

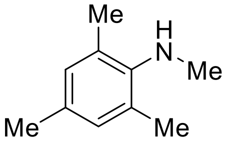

Compound **3e** was obtained from 2,4,6-trimethylaniline (**S24b**) by using **General procedure B** as a light-yellow oil (0.36 g, 24% yield).

^{1}H NMR (300 MHz, $CDCl_3$) δ 6.82 (s, 2H), 2.96 (brs, 1H), 2.74 (s, 3H), 2.26 (s, 6H), 2.23 (s, 3H).

MS(EI): *m/z* 149 $[M]^+$. The chemical shifts were consistent with those reported in the literature.[17]

4-Methoxy-*N*-methylaniline (3f)

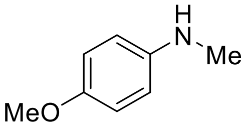

Compound **3b** was obtained from *p*-anisidine (**S24c**) by using **General procedure B** as a light-yellow oil (0.38 g, 28% yield).

^{1}H NMR (300 MHz, $CDCl_3$) δ 6.81 (d, *J* = 8.9 Hz, 2H), 6.59 (d, *J* = 8.9 Hz, 2H), 3.76 (s, 3H), 3.43 (brs, 1H), 2.81 (s, 3H).

MS(EI): *m/z* 137 $[M]^+$. The chemical shifts were consistent with those reported in the literature.[16]

***N*-Methylbenzo[*d*][1,3]dioxol-5-amine (3g)**

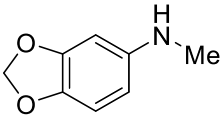

Compound **3g** was obtained from 3,4-methylenedioxyaniline (**S24d**) by using **General procedure B** as a light-yellow oil (0.36 g, 24% yield).

^{1}H NMR (300 MHz, $CDCl_3$) δ 6.67 (d, *J* = 8.3 Hz, 1H), 6.25 (d, *J* = 2.3 Hz, 1H), 6.04 (dd, *J* = 8.3, 2.4 Hz, 1H), 5.85 (s, 2H), 2.78 (s, 3H).

MS(EI): *m/z* 151 $[M]^+$. The chemical shifts were consistent with those reported in the literature.[16]

***N*-Methyl-4-(trifluoromethoxy)aniline (3h)**

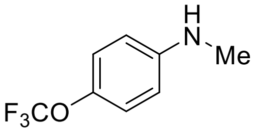

Compound **3h** was obtained from 4-(trifluoromethoxy)aniline (**S24e**) by using **General procedure B** as a light-yellow oil (0.69 g, 47% yield).

^{1}H NMR (300 MHz, $CDCl_3$) δ 7.05 (d, *J* = 8.5 Hz, 2H), 6.55 (d, *J* = 8.7 Hz, 2H), 3.77 (s, 1H), 2.83 (s, 3H).

^{19}F NMR (282 MHz, $CDCl_3$) δ -58.54 (s, 3F).

MS(EI): *m/z* 191 $[M]^+$. The chemical shifts were consistent with those reported in the literature.[18]

4-Chloro-*N*-methylaniline (3i)

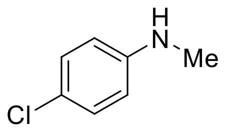

Compound **3i** was obtained from 1-chloro-4-iodobenzene (**S25c**) by using **General procedure C** as a light-yellow oil (1.10 g, 79% yield).

^{1}H NMR (300 MHz, $CDCl_3$) δ 7.13 (d, *J* = 8.8 Hz, 2H), 6.53 (d, *J* = 8.8 Hz, 2H), 3.71 (brs, 1H), 2.81 (s, 3H).

MS(EI): *m/z* 141 $[M]^+$. The chemical shifts were consistent with those reported in the literature.[17]

3-Chloro-*N*-methylaniline (3j)

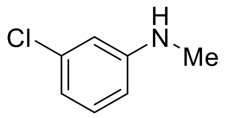

Compound **3j** was obtained from 3-chloroaniline (**S24f**) by using **General procedure B** as a light-yellow oil (0.52 g, 37% yield).

^{1}H NMR (300 MHz, $CDCl_3$) δ 7.08 (t, *J* = 8.0 Hz, 1H), 6.68 – 6.65 (m, 1H), 6.58 – 6.57 (m, 1H), 6.50 – 6.44 (m, 1H), 3.79 (brs, 1H), 2.82 (s, 3H).

MS(EI): *m/z* 141 $[M]^+$. The chemical shifts were consistent with those reported in the literature.[18]

4-Bromo-*N*-methylaniline (3k)

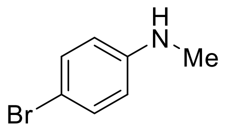

Compound **3k** was obtained from 4-bromoaniline (**S24g**) by using **General procedure B** as a light-yellow oil (1.21 g, 65% yield).

^{1}H NMR (300 MHz, $CDCl_3$) δ 7.24 (d, *J* = 8.6 Hz, 2H), 6.46 (d, *J* = 8.8 Hz, 2H), 3.71 (s, 1H), 2.78 (s, 3H).

MS(EI): *m/z* 186 $[M]^+$. The chemical shifts were consistent with those reported in the literature.[18]

***N*-Butylaniline (3m)**

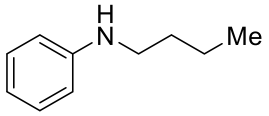

Compound **3m** was obtained from iodobenzene (**S25d**) and *n*-$BuNH_2$ by using **General procedure C** as a light-yellow oil (0.74 g, 50% yield).

^{1}H NMR (300 MHz, $CDCl_3$) δ 7.19 (d, *J* = 8.6 Hz, 2H), 6.73 – 6.65 (m, 1H), 6.61 (d, *J* = 7.6 Hz, 2H), 3.59 (brs, 1H), 3.11 (t, *J* = 7.1 Hz, 2H), 1.68 – 1.51 (m, 2H), 1.50 – 1.35 (m, 2H), 0.96 (t, *J* = 7.3 Hz, 3H).

MS(EI): *m/z* 149 $[M]^+$. The chemical shifts were consistent with those reported in the literature.[15]

(1*R*,2*S*,5*R*)-2-Isopropyl-5-methylcyclohexyl 4-(methylamino)benzoate (3v)

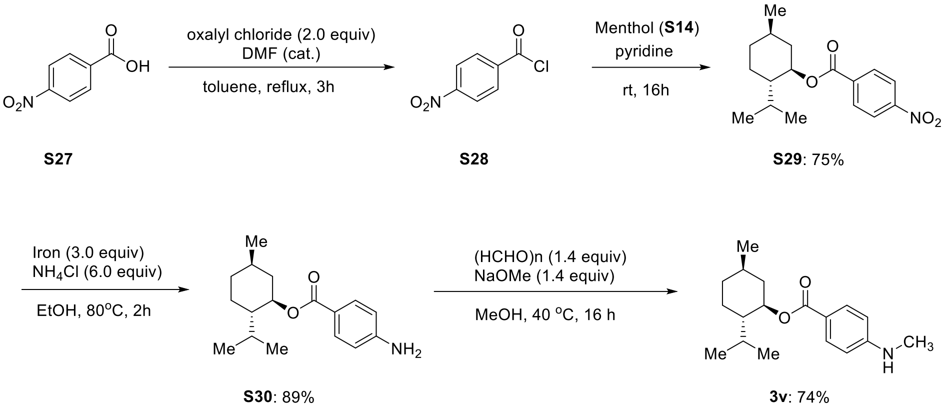

To a solution of *p*-nitrobenzoic acid (**S27**, 1.67 g, 10.0 mmol) in anhydrous toluene (20 mL) were added oxalyl chloride (1.72 mL, 20.0 mmol) and 3 drops of DMF at 0 °C. The reaction mixture was stirred for 8 h at 0 °C under N_2. The solvent was removed under reduced pressure to afford the *p*-nitrobenzoyl chloride **S28** as a light-yellow solid. To the former flask was charged with (-)-menthol **S14** (1.56 g, 10.0 mmol) and followed by anhydrous pyridine (20 mL), the resulted solution was stirred for 16 h at room temperature. After the reaction finished, removal of pyridine under reduced pressure, the residue was dissolved in DCM (30 mL) and washed with 1 N HCl, saturated $NaHCO_3$ aqueous solution and brine, the separated organic layer was dried over Na_2SO_4 and concentrated under reduced pressure, the residue was purified by column chromatography (*n*-hexane/EtOAc: 10/1) to afford the ester **S29** as a light-yellow solid (2.29 g, 75% yield).

In a 100 mL round bottom flask, previous obtained ester (**S29**, 2.20 g, 7.2 mmol), Iron powder (1.20 g, 21.6 mmol) and NH_4Cl (2.30 g, 43.2 mmol) was added subsequently into H_2O/EtOH (50 mL, *v*/*v*=1:5). The mixture was stirred at 80° C for 2 h. Completion of the reaction was monitored by

TLC. Filtered through a pad of celite to remove the solid, and then extracted with DCM, dried, and concentrated under reduced pressure. Purification by chromatography on a short silica gel column (*n*-hexane/EtOAc: 5/1) to afford corresponding aniline **S30** as a light-yellow oil (1.80 g, 89% yield). Synthetic procedure was followed previously reported method.[19] To a solution of previous obtained aniline (**S30**, 0.55 g, 2.0 mmol), paraformaldehyde (84.0 mg, 2.8 mmol) in MeOH (10 mL, 0.2 M) was added sodium methoxide solution (0.56 mL, 2.8 mmol, 5 M in methanol) and stirred at 50 °C for 5 hours. Then sodium borohydride (0.23 g, 6.0 mmol) was added under nitrogen and the reaction was stirred at room temperature. The reaction mixture was monitored by TLC until aniline was fully consumed, the solvent was removed under reduced pressure, then washed with saturated NH_4Cl and extracted with EtOAc. The combined organic layer was dried over Na_2SO_4, filtered, and concentrated. The crude material was purified by column chromatography (*n*-hexane/EtOAc: 10/1) to afford corresponding *N*-methylaniline **3v** as a light-yellow solid (0.42 g, 74% yield), m.p. = 106.5 – 109.0 °C.

^{1}H NMR (700 MHz, $CDCl_3$) δ 7.96 – 7.82 (m, 2H), 6.71 – 6.41 (m, 2H), 4.87 (td, *J* = 10.9, 4.4 Hz, 1H), 4.19 (br, 1H), 2.88 (s, 3H), 2.14 – 2.07 (m, 1H), 2.00 – 1.93 (m, 1H), 1.74 – 1.68 (m, 2H), 1.58 – 1.48 (m, 2H), 1.15 – 1.09 (m, 1H), 1.06 (td, *J* = 12.2, 10.9 Hz, 1H), 0.91 (t, *J* = 7.1 Hz, 7H), 0.78 (d, *J* = 6.9 Hz, 3H).

^{13}C NMR (176 MHz, $CDCl_3$) δ 166.5, 152.9, 131.6, 119.1, 111.2, 74.0, 47.5, 41.3, 34.5, 31.6, 30.3, 26.6, 23.8, 22.2, 20.9, 16.7.

IR (KBr): 3387, 2961, 2919, 2893, 2860, 1681, 1601, 1535, 1500, 1455, 1346, 1287, 1174, 1119, 1102, 981, 964, 832, 769, 700 cm^{-1}.

HRMS (ESI) [$C_{19}H_{17}NNa$] $[M+Na]^+$ calculated: 312.1939, found: 312.1943.

***N*-Cyclopropylaniline (3w)**

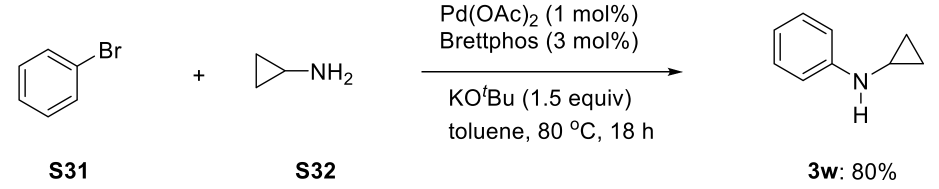

Synthetic procedure was followed previously reported method with modification.[20] In a glovebox, a flame-dried flask was charged with $Pd(OAc)_2$ (22.4 mg, 0.1 mmol) and BrettPhos (160 mg, 0.3 mmol), bromobenzene (**S31**, 1.57 g, 10.0 mmol), cyclopropylamine (**S32**, 0.68 g, 12.0 mmol),

KO^{t}Bu (1.68 g, 15.0 mmol), and degassed toluene (50 mL), then sealed and removed from the glovebox, the mixture stirred at 80 ºC in an oil bath for 18 h. After completion, the reaction mixture was cooled to room temperature, diluted with EtOAc, filtered over a short pad of silica gel, and concentrated in vacuo. The crude was purified by column chromatography (*n*-hexane/EtOAc: 20/1) to afford desired product **3w** as a light-yellow oil (1.07g, 80% yield).

^{1}H NMR (300 MHz, $CDCl_3$) δ 7.38 – 7.05 (m, 2H), 6.95 – 6.59 (m, 3H), 4.18 (s, 1H), 2.54 – 2.36 (m, 1H), 0.83 – 0.64 (m, 2H), 0.64 – 0.39 (m, 2H).

MS(EI): *m/z* 133 $[M]^+$. The chemical shifts were consistent with those reported in the literature.[21]

***N*-(Methyl-*d*^{3})aniline (*d*^{3}-3a)**

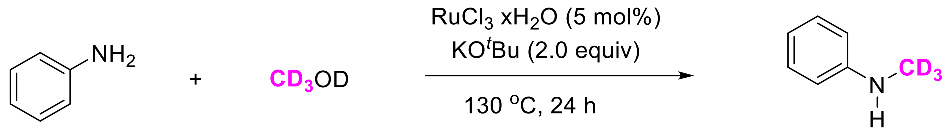

S24h *d*^{3}-**3a**: 68%

Synthetic procedure was followed previously reported method with modification.[22] In a nitrogen glovebox, to a 20 mL ACE® pressure tube was charged with $RuCl_3$•nH_2O (20.6 mg, 0.1 mmol), KO*t*Bu (0.45 g, 4.0 mmol), aniline (**S24h**, 200 μL, 2.0 mmol), and MeOH-*d*^{3} (5 mL). The pressure tube was then sealed and allowed to stir at 130 ºC in an oil bath for 24 h. After completion of the reaction, the pressure tube was cooled to room temperature, and then filtered through a short silica pad and washed with EtOAc to remove the solid catalyst and base. After concentrate the solvent, the crude mixture was purified by column chromatography (*n*-hexane/EtOAc: 20/1) to afford desired product ***d*^{3}-3a** as a light-yellow oil (150 mg, 68% yield).

^{1}H NMR (300 MHz, $CDCl_3$) δ 7.23 (t, J = 7.8 Hz, 2H), 6.74 (t, J = 7.3 Hz, 1H), 6.64 (d, J = 7.9 Hz, 2H), 3.68 (br, 1H), 2.82 (s, 0.2H).

MS(EI): *m/z* 110 $[M]^+$. The chemical shifts were consistent with those reported in the literature.[23]

3.4 Synthesis of silylboronates

Dimethyl(phenyl)(4,4,5,5-tetramethyl-1,3,2-dioxaborolan-2-yl)silane ($PhMe_2SiBpin$) was purchased from TCI, $Et_3SiBpin$, *n*-$Pr_3SiBpin$, $^tBuMe_2SiBpin$ were obtained by repeat the previous procedures, $TMS_3SiBpin$ was prepared according to procedures previously reported work with modification.

Triethyl(4,4,5,5-tetramethyl-1,3,2-dioxaborolan-2-yl)silane ($Et_3SiBpin$)

Silylboronate $Et_3SiBpin$ was prepared according to the procedures previously reported.[24] An oven-dried vial was charged with $[Ir(COD)OMe]_2$ (66.3 mg, 0.1 mmol), dtbpy (54.0 mg, 0.2 mmol), B_2pin_2 (5.10 g, 20.0 mmol), cyclohexane (10 mL), and triethylsilane (12.8 mL, 80.0 mmol) inside a nitrogen-filled glovebox. The resulting dark brown solution was heated at 80 °C overnight outside the glovebox. After being cooled to room temperature, the crude reaction mixture was concentrated in vacuo, and the residue was purified by flash column chromatography (*n*-hexane) to afford the $Et_3SiBpin$ as a colorless oil (3.74 g, 77% yield).

^{1}H NMR (300 MHz, $CDCl_3$) δ 1.22 (s, 12H), 1.05 – 0.87 (m, 9H), 0.71 – 0.48 (m, 6H).

MS(EI): *m/z* 242 $[M]^+$. The chemical shifts were consistent with those reported in the literature.[3]

Tripropyl(4,4,5,5-tetramethyl-1,3,2-dioxaborolan-2-yl)silane ($^nPr_3SiBpin$)

Silylboronate $^nPr_3SiBpin$ was prepared according to the procedures previously reported.[24] An oven-dried vial was charged with $[Ir(COD)OMe]_2$ (16.5 mg, 0.025 mmol), dtbpy (13.5 mg, 0.05 mmol), B_2pin_2 (1.30 g, 5.0 mmol), cyclohexane (2 mL), and tripropylsilane (4.2 mL, 20.0 mmol) inside a nitrogen-filled glovebox. The resulting dark brown solution was heated at 80 °C overnight outside the glovebox. After being cooled to room temperature, the crude reaction mixture was concentrated in vacuo, and the residue was purified by flash column chromatography (*n*-hexane) to afford the $^nPr_3SiBpin$ as a colorless oil (0.70 g, 49% yield).

^{1}H NMR (300 MHz, $CDCl_3$) δ 1.48 – 1.29 (m, 6H), 1.22 (s, 12H), 0.94 (t, J = 6.0 Hz, 9H), 0.66 – 0.51 (m, 6H).

MS(EI): *m/z* 269 $[M–CH_3]^+$. The chemical shifts were consistent with those reported in the literature.[3]

tert-Butyldimethyl(4,4,5,5-tetramethyl-1,3,2-dioxaborolan-2-yl)silane (^{t}BuMe$_2$SiBpin)

Silylboronate ^{t}BuMe$_2$SiBpin was prepared according to the procedures previously reported.[24] An oven-dried vial was charged with $[Ir(COD)OMe]_2$ (16.5 mg, 0.025 mmol), dtbpy (13.5 mg, 0.05 mmol), B$_2$pin$_2$ (1.30 g, 5.0 mmol), cyclohexane (2 mL), and *tert*-butyldimethylsilane (3.3 mL, 20.0 mmol) inside a nitrogen-filled glovebox. The resulting dark brown solution was heated at 80 °C overnight outside the glovebox. After being cooled to room temperature, the crude reaction mixture was concentrated in vacuo, and the residue was purified by flash column chromatography (*n*-hexane) to afford the ^{t}BuMe$_2$SiBpin as a white solid (0.69 g, 57% yield).

^{1}H NMR (300 MHz, CDCl$_3$) δ 1.23 (s, 12H), 0.91 (s, 9H), 0.01 (s, 6H).

MS(EI): *m/z* 185 [M–^{t}Bu]$^+$. The chemical shifts were consistent with those reported in the literature.[3]

1,1,1,3,3,3-Hexamethyl-2-(4,4,5,5-tetramethyl-1,3,2-dioxaborolan-2-yl)-2-(trimethylsilyl)trisilane (TMS$_3$SiBpin)

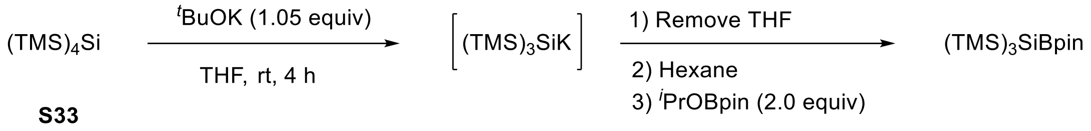

In a nitrogen-filled glovebox, an oven-dried flask were charged with tetrakis(trimethylsilyl)silane (**S33**, 1.65 g, 5.2 mmol), anhydrous THF (5 mL), then KO^{t}Bu (0.61 g, 5.4 mmol). After the resultant solution was stirred for 4 h at room temperature, the solvent was removed under reduced pressure, and *n*-hexane (10 mL) was added to the flask. Then, 2-isopropoxy-4,4,5,5-tetramethyl-1,3,2-dioxaborolane (^{i}PrOBpin, 1.92 g, 10.3 mmol) was added in one portion to the mixture. After the resultant solution was stirred for 4 h at rt, then the mixture was directly filtered through a short silica-gel column with *n*-hexane as the eluent. After removal of the solvents under reduced pressure, the residue was purified by flash column chromatography (*n*-hexane) to afford TMS$_3$SiBpin as a white solid (0.89 g, 46% yield).

^{1}H NMR (300 MHz, CDCl$_3$) δ 1.19 (s, 12H), 0.18 (s, 27H).

MS(EI): *m/z* 374 [M]$^+$. The chemical shifts were consistent with those reported in the literature.[25]

3.5 General procedure for the optimization of defluoroamination reaction

General procedure D: In a nitrogen-filled glovebox, to a flame-dried screw-capped test tube was added 4-fluorobiphenyl **1a** (17.2 mg, 0.10 mmol), *N*-methylaniline **3a**, base, solvent (0.5 mL), and silyl boronate, sequentially. The tube then was sealed and moved out from the glovebox. The solution was stirred at room temperature for indicated hours. The reaction tube was diluted with Et_2O (5 mL), then extracted with Et_2O and water, washed with brine, dried over Na_2SO_4. After filtered and concentrated under vacuum, the obtained reaction crude was followed by adding 3-fluoropyridine (8.6 µl, 0.1 mmol) as an internal standard. Corresponding yields were copied from the ^1H NMR and ^{19}F NMR analysis.

3.6 General procedure for the defluoroamination reaction

General procedure E: In a nitrogen-filled glovebox, to a flame-dried screw-capped test tube was added organic fluorides **1** or **2** (0.2 mmol), KO*t*Bu (89.6 mg, 0.8 mmol), dry triglyme (1.0 mL), and secondary amines **3** (0.6 mmol), sequentially. After stirring for 5 min, silyl boronate (96.8 mg, 0.4 mmol) was added to the mixture, and the tube then was sealed and moved out from the glovebox. The solution was stirred at room temperature for 24 h. The reaction tube was diluted with Et_2O (5 mL), then extracted with Et_2O and water, washed with brine, dried over Na_2SO_4. After filtered and concentrated under vacuum, the obtained reaction crude was followed by adding 3-fluoropyridine (8.6 µL, 0.1 mmol) as an internal standard. After NMR analysis was conducted, the mixture was then concentrated again to give the crude, which was purified by column chromatography on silica gel to give the corresponding amines **4** or **5**.

N-Methyl-N-phenyl-[1,1'-biphenyl]-4-amine (4aa)

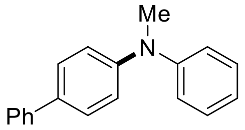

Compound **4aa** was prepared according to the **General procedure E** from **1a** (34.4 mg, 0.2 mmol) and **3a** (64.0 µL, 0.6 mmol), and purified by silica gel column chromatography (*n*-hexane/DCM: 10/1) as a white solid (46.1 mg, 89% yield).

^{1}H NMR (300 MHz, $CDCl_3$) δ 7.63 – 7.57 (m, 2H), 7.56 – 7.49 (m, 2H), 7.44 (t, *J* = 7.5 Hz, 2H), 7.38 – 7.28 (m, 3H), 7.19 – 6.99 (m, 5H), 3.38 (s, 3H).

MS(EI): *m/z* 259 $[M]^+$. The chemical shifts were consistent with those reported in the literature.[26]

N-Methyl-N-phenyl-[1,1'-biphenyl]-3-amine (4ba)

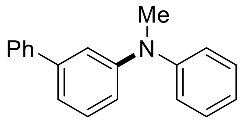

Compound **4ba** was prepared according to the **General procedure E** from **1b** (34.4 mg, 0.2 mmol) and **3a** (64.0 µL, 0.6 mmol), and purified by silica gel column chromatography (*n*-hexane/DCM: 10/1) as a colorless oil (44.2 mg, 85% yield)

^{1}H NMR (500 MHz, $CDCl_3$) δ 7.65 – 7.59 (m, 2H), 7.47 (t, *J* = 7.6 Hz, 2H), 7.42 – 7.29 (m, 5H), 7.27 – 7.21 (m, 1H), 7.18 – 7.12 (m, 2H), 7.09 – 7.01 (m, 2H), 3.42 (s, 3H).

^{13}C NMR (126 MHz, $CDCl_3$) δ 149.5, 149.0, 142.5, 141.5, 129.7, 129.4, 128.8, 127.4, 127.3, 121.6, 120.9, 120.2, 119.2, 119.1, 40.5.

IR (KBr): 3058, 3032, 2941, 2879, 2812, 1591, 1495, 1450, 1418, 1345, 1232, 1131, 990, 895, 755, 698 cm^{-1}.

HRMS (EI) [$C_{19}H_{17}N$] $[M]^+$ calculated: 259.1361, found: 259.1373.

***N*-Methyl-*N*-phenyl-[1,1'-biphenyl]-2-amine (4ca)**

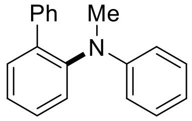

Compound **4ca** was prepared according to the **General procedure E** from **1c** (34.4 mg, 0.2 mmol) and **3a** (64.0 µL, 0.6 mmol), and purified by silica gel column chromatography (*n*-hexane/DCM: 10/1) as a white solid (41.5 mg, 80% yield).

^{1}H NMR (300 MHz, $CDCl_3$) δ 7.54 – 7.47 (m, 1H), 7.44 – 7.29 (m, 8H), 7.23 (dd, *J* = 8.7, 7.3 Hz, 2H), 6.81 – 6.70 (m, 3H), 2.85 (s, 3H).

MS(EI): *m/z* 259 $[M]^+$. The chemical shifts were consistent with those reported in the literature.[27]

***N*-Methyl-*N*-phenylnaphthalen-1-amine (4da)**

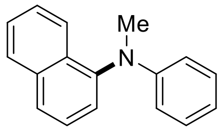

Compound **4da** was prepared according to the **General procedure E** from **1d** (26.0 µL, 0.2 mmol) and **3a** (64.0 µL, 0.6 mmol), and purified by silica gel column chromatography (*n*-hexane/DCM: 10/1) as a white solid (38.1 mg, 82% yield).

^{1}H NMR (300 MHz, $CDCl_3$) δ 7.94 (t, *J* = 6.8 Hz, 2H), 7.83 (d, *J* = 8.3 Hz, 1H), 7.59 – 7.36 (m, 4H), 7.20 (dd, *J* = 8.8, 7.3 Hz, 2H), 6.78 (t, *J* = 7.3 Hz, 1H), 6.67 (dd, *J* = 8.8, 1.1 Hz, 2H), 3.43 (s, 3H).

MS(EI): *m/z* 233 $[M]^+$. The chemical shifts were consistent with those reported in the literature.[26]

***N*-Methyl-*N*-phenylaniline (4ea)**

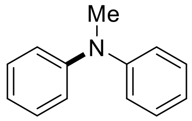

Compound **4ea** was prepared according to the **General procedure E** from **1e** (38.0 µL, 0.4 mmol) and **3a** (128.0 µL, 1.2 mmol), and purified by silica gel column chromatography (*n*-hexane/DCM: 20/1) as a light-yellow oil (57.9 mg, 79% yield).

^{1}H NMR (300 MHz, $CDCl_3$) δ 7.34 – 7.26 (m, 4H), 7.10 – 7.02 (m, 4H), 6.98 (t, *J* = 7.3 Hz, 2H), 3.34 (s, 3H).

MS(EI): *m/z* 183 $[M]^+$. The chemical shifts were consistent with those reported in the literature.[26]

N,4-Dimethyl-N-phenylaniline (4fa)

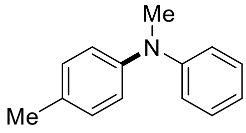

Compound **4fa** was prepared according to the **General procedure E** from **1f** (44.0 µL, 0.4 mmol) and **3a** (128.0 µL, 1.2 mmol), and purified by silica gel column chromatography (*n*-hexane/DCM: 10/1) as a light-yellow oil (58 mg, 74% yield).

^{1}H NMR (300 MHz, $CDCl_3$) δ 7.22 (t, J = 7.9 Hz, 2H), 7.11 (d, J = 8.1 Hz, 2H), 6.99 (d, J = 8.3 Hz, 2H), 6.96 – 6.79 (m, 3H), 3.28 (s, 3H), 2.32 (s, 3H).

MS(EI): *m/z* 197 $[M]^+$. The chemical shifts were consistent with those reported in the literature.[26]

4-Methoxy-N-methyl-N-phenylaniline (4ga)

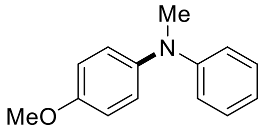

Compound **4ga** was prepared according to the **General procedure E** from **1g** (45.0 µL, 0.4 mmol) and **3a** (128.0 µL, 1.2 mmol), and purified by silica gel column chromatography (*n*-hexane/DCM: 5/1) as a light-yellow oil (56 mg, 66% yield).

^{1}H NMR (300 MHz, $CDCl_3$) δ 7.19 (t, J = 8.0 Hz, 2H), 7.12 – 7.05 (m, 2H), 6.94 – 6.85 (m, 2H), 6.79 (d, J = 6.9 Hz, 3H), 3.80 (s, 3H), 3.25 (s, 3H).

MS(EI): *m/z* 213 $[M]^+$. The chemical shifts were consistent with those reported in the literature.[26]

N-Methyl-N-phenyl-4-(trifluoromethyl)aniline (4ha)

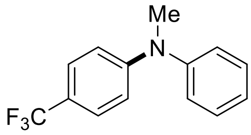

Compound **4ha** was prepared according to the **General procedure E** from **1h** (51.0 µL, 0.4 mmol) and **3a** (128.0 µL, 1.2 mmol), and purified by silica gel column chromatography (*n*-hexane/DCM: 10/1) as a colorless oil (82.0 mg, 82% yield).

^{1}H NMR (300 MHz, $CDCl_3$) δ 7.59 – 7.34 (m, 4H), 7.21 (dd, J = 8.0, 2.6 Hz, 3H), 6.87 (d, J = 8.5 Hz, 2H), 3.36 (s, 3H).

^{19}F NMR (282 MHz, $CDCl_3$) δ -61.63 (s, 3F).

MS(EI): *m/z* 251 $[M]^+$. The chemical shifts were consistent with those reported in the literature.[28]

4-Chloro-*N*-methyl-*N*-phenylaniline (4ia)

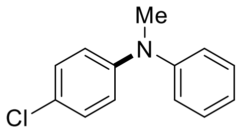

Compound **4ia** was prepared according to the **General procedure E** from **1i** (52.0 mg, 0.4 mmol) and **3a** (128.0 μL, 1.2 mmol), and purified by silica gel column chromatography (*n*-hexane/DCM: 10/1) as a light-yellow oil (44.1 mg, 51% yield).

^{1}H NMR (300 MHz, $CDCl_3$) δ 7.36 – 7.27 (m, 2H), 7.24 – 7.17 (m, 2H), 7.10 – 6.98 (m, 3H), 6.95 – 6.87 (m, 2H), 3.30 (s, 3H).

^{13}C NMR (75 MHz, $CDCl_3$) δ 148.8, 147.8, 129.5, 129.2, 125.7, 122.3, 121.6, 120.7, 40.5.

MS(EI): *m/z* 217 $[M]^+$. The chemical shifts were consistent with those reported in the literature.[29]

3-Bromo-*N*-methyl-*N*-phenylaniline (4ja)

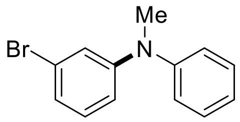

Compound **4ja** was prepared according to the **General procedure E** from **1j** (70.0 mg, 0.4 mmol) and **3a** (128.0 μL, 1.2 mmol), and purified by silica gel column chromatography (*n*-hexane/DCM: 15/1) as a light-yellow oil (41.1 mg, 39% yield).

^{1}H NMR (300 MHz, $CDCl_3$) δ 7.42 – 7.29 (m, 2H), 7.18 – 7.07 (m, 3H), 7.12 – 7.04 (m, 2H), 7.02 – 6.96 (m, 1H), 6.88 – 6.80 (m, 1H), 3.31 (s, 3H).

MS(EI): *m/z* 262 $[M]^+$. The chemical shifts were consistent with those reported in the literature.[30]

***N*-Methyl-4-(naphthalen-1-yl)-*N*-phenylaniline (4ka)**

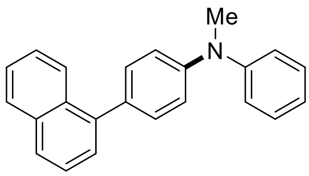

Compound **4ka** was prepared according to the **General procedure E** from **1k** (44.4 mg, 0.2 mmol) and **3a** (64.0 μL, 0.6 mmol), and purified by silica gel column chromatography (*n*-hexane/DCM: 10/1) as a white solid (47.5 mg, 77% yield), m.p. = 124.5 – 125.3 ^{o}C.

^{1}H NMR (300 MHz, $CDCl_3$) δ 8.08 (d, *J* = 8.4 Hz, 1H), 7.95 (d, *J* = 7.5 Hz, 1H), 7.88 (d, *J* = 8.2

Hz, 1H), 7.59 – 7.48 (m, 4H), 7.48 – 7.44 (m, 2H), 7.39 (dd, J = 8.6, 7.4 Hz, 2H), 7.23 (dd, J = 8.6, 1.0 Hz, 2H), 7.18 – 7.14 (m, 2H), 7.09 (t, J = 7.3 Hz, 1H), 3.45 (s, 3H).

^{13}C NMR (75 MHz, $CDCl_3$) δ 148.9, 148.3, 140.2, 134.0, 133.0, 131.9, 130.9, 129.5, 128.4, 127.3, 127.0, 126.3, 126.0, 125.8, 125.6, 122.2, 121.7, 119.1, 40.4.

IR (KBr): 3059, 3032, 2929, 2902, 2818, 1590, 1497, 1392, 1345, 1252, 1185, 1131, 849, 801, 780, 759, 694 cm^{-1}.

HRMS (EI) [$C_{23}H_{19}N$] $[M]^+$ calculated: 309.1517, found: 309.1532.

***N*,*4'*-Dimethyl-*N*-phenyl-[1,1'-biphenyl]-4-amine (4la)**

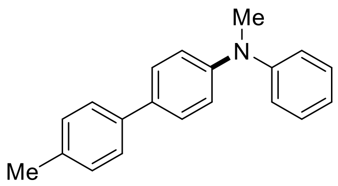

Compound **4la** was prepared according to the **General procedure E** from **1l** (37.2 mg, 0.2 mmol) and **3a** (64.0 µL, 0.6 mmol), and purified by silica gel column chromatography (*n*-hexane/DCM: 10/1) as a white solid (50.0 mg, 86% yield), m.p. = 113.8 – 114.6 °C.

^{1}H NMR (500 MHz, $CDCl_3$) δ 7.51 (dd, J = 10.4, 8.4 Hz, 4H), 7.33 (dd, J = 8.6, 7.4 Hz, 2H), 7.26 (d, J = 7.9 Hz, 2H), 7.12 (dd, J = 8.6, 1.0 Hz, 2H), 7.09 (d, J = 8.7 Hz, 2H), 7.02 (t, J = 7.3 Hz, 1H), 3.38 (s, 3H), 2.42 (s, 3H).

^{13}C NMR (126 MHz, $CDCl_3$) δ 149.0, 148.2, 138.1, 136.4, 133.8, 129.6, 129.4, 127.7, 126.6, 121.8, 121.2, 120.1, 40.4, 21.2.

IR (KBr): 3059, 3026, 2917, 2884, 2819, 1590, 1496, 1342, 1255, 1128, 868, 808, 758, 699 cm^{-1}.

HRMS (EI) [$C_{20}H_{19}N$] $[M]^+$ calculated: 273.1517, found: 273.1505.

***4'*-Methoxy-*N*-methyl-*N*-phenyl-[1,1'-biphenyl]-4-amine (4ma)**

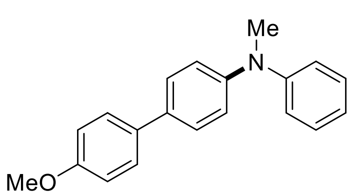

Compound **4ma** was prepared according to the **General procedure E** from **1m** (40.4 mg, 0.2 mmol) and **3a** (64.0 µL, 0.6 mmol), and purified by silica gel column chromatography (*n*-hexane/DCM: 5/1) as a white solid (48.3 mg, 83% yield), m.p. = 144.1 – 144.9 °C.

^{1}H NMR (300 MHz, $CDCl_3$) δ 7.53 (d, *J* = 8.8 Hz, 2H), 7.49 (d, *J* = 8.7 Hz, 2H), 7.32 (dd, *J* = 8.6, 7.4 Hz, 2H), 7.12 – 7.06 (m, 4H), 7.03 – 6.96 (m, 3H), 3.87 (s, 3H), 3.38 (s, 3H).

^{13}C NMR (126 MHz, $CDCl_3$) δ 158.8, 149.0, 147.9, 133.7, 133.6, 129.4, 127.7, 127.5, 121.6, 120.9, 120.5, 114.3, 55.5, 40.4.

IR (KBr): 3033, 3003, 2953, 2835, 1594, 1496, 1349, 1281, 1250, 1181, 1036, 824, 766, 704 cm^{-1}.

HRMS (EI) [$C_{20}H_{19}NO$] $[M]^+$ calculated: 289.1467, found: 289.1480.

4'-(Benzyloxy)-*N*-methyl-*N*-phenyl-[1,1'-biphenyl]-4-amine (4na)

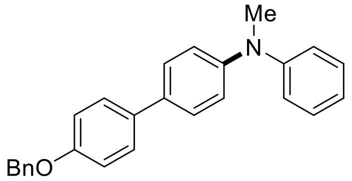

Compound **4na** was prepared according to the **General procedure E** from **1n** (56.0 mg, 0.2 mmol) and **3a** (64.0 µL, 0.6 mmol), and purified by silica gel column chromatography (*n*-hexane/DCM: 5/1) as a white solid (44.5 mg, 61% yield), m.p. = 163.1 – 163.5 °C.

^{1}H NMR (500 MHz, $CDCl_3$) δ 7.55 – 7.52 (m, 2H), 7.51 – 7.47 (m, 4H), 7.43 (t, *J* = 7.4 Hz, 2H), 7.37 (t, *J* = 7.3 Hz, 1H), 7.35 – 7.31 (m, 2H), 7.11 (dd, *J* = 9.0, 1.4 Hz, 3H), 7.09 – 7.05 (m, 3H), 7.04 – 6.99 (m, 1H), 5.13 (s, 2H), 3.38 (s, 3H).

^{13}C NMR (126 MHz, $CDCl_3$) δ 158.0, 149.0, 147.9, 137.2, 133.9, 133.6, 129.4, 128.7, 128.1, 127.7, 127.6, 127.5, 121.6, 120.9, 120.4, 115.2, 70.2, 40.4.

IR (KBr): 3061, 3037, 2910, 2864, 2835, 1594, 1503, 1378, 1348, 1279, 1248, 1179, 1085, 1004, 860, 824, 739, 700 cm^{-1}.

HRMS (EI) [$C_{26}H_{23}NO$] $[M]^+$ calculated: 365.1780, found: 365.1772.

4'-Chloro-*N*-methyl-*N*-phenyl-[1,1'-biphenyl]-4-amine (4oa)

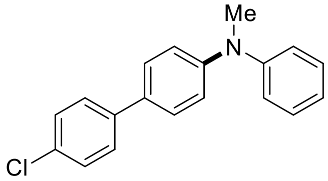

Compound **4oa** was prepared according to the **General procedure E** from **1o** (41.2 mg, 0.2 mmol) and **3a** (64.0 µL, 0.6 mmol), and purified by silica gel column chromatography (*n*-hexane/DCM: 10/1) as a white solid (46.0 mg, 77% yield), m.p. = 145.3 – 146.0 °C.

^{1}H NMR (500 MHz, $CDCl_3$) δ 7.53 – 7.49 (m, 2H), 7.49 – 7.44 (m, 2H), 7.39 (d, *J* = 8.6 Hz, 2H), 7.35 (dd, *J* = 8.6, 7.3 Hz, 2H), 7.15 (dd, *J* = 8.7, 1.1 Hz, 2H), 7.09 – 7.02 (m, 3H), 3.38 (s, 3H).

^{13}C NMR (126 MHz, $CDCl_3$) δ 148.7, 139.4, 132.6, 131.8, 129.5, 129.0, 127.8, 127.7, 122.6, 122.2, 119.2, 40.4.

IR (KBr): 3094, 3060, 3028, 2926, 2899, 2828, 1593, 1487, 1396, 1417, 1340, 1255, 1090, 821, 760, 695 cm^{-1}.

HRMS (EI) [$C_{19}H_{16}ClN$] $[M]^+$ calculated: 293.0971, found: 293.0972.

4'-Bromo-*N*-methyl-*N*-phenyl-[1,1'-biphenyl]-4-amine (4pa)

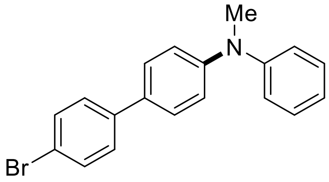

Compound **4pa** was prepared according to the **General procedure E** from **1p** (51.0 mg, 0.2 mmol) and **3a** (64.0 µL, 0.6 mmol), and purified by silica gel column chromatography (*n*-hexane/DCM: 10/1) as a white solid (40.2 mg, 59% yield), m.p. = 98.6 – 99.3 °C.

^{1}H NMR (300 MHz, $CDCl_3$) δ 7.63 – 7.53 (m, 2H), 7.56 – 7.47 (m, 2H), 7.48 – 7.36 (m, 2H), 7.36 – 7.28 (m, 2H), 7.17 – 6.96 (m, 5H), 3.37 (s, 3H).

^{13}C NMR (75 MHz, $CDCl_3$) δ 148.9, 148.5, 141.0, 133.5, 129.4, 128.8, 127.9, 126.71, 126.69, 122.1, 121.6, 119.8, 40.4.

IR (KBr): 3055, 3033, 2939, 2884, 2817, 1590, 1523, 1488, 1344, 1256, 1131, 870, 817, 753, 692 cm^{-1}.

HRMS (ESI) [$C_{19}H_{17}BrN$] $[M+H]^+$ calculated: 338.0544, found: 338.0552.

4'-(Methyl(phenyl)amino)-[1,1'-biphenyl]-4-carbonitrile (4qa)

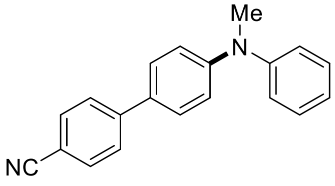

Compound **4qa** was prepared according to the **General procedure E** from **1q** (39.4 mg, 0.2 mmol) and **3a** (64.0 µL, 0.6 mmol), and purified by silica gel column chromatography (*n*-hexane/DCM: 5/1) as a white solid (29.0 mg, 51% yield), m.p. = 139.2 – 140.0 °C.

^{1}H NMR (500 MHz, $CDCl_3$) δ 7.70 – 7.61 (m, 4H), 7.51 – 7.47 (m, 2H), 7.37 (dd, *J* = 8.4, 7.3 Hz, 2H), 7.19 (dd, *J* = 8.6, 1.1 Hz, 2H), 7.13 (t, *J* = 7.4 Hz, 1H), 7.02 – 6.96 (m, 2H), 3.38 (s, 3H).

^{13}C NMR (75 MHz, $CDCl_3$) δ 149.6, 148.3, 145.4, 132.7, 129.7, 129.6, 127.9, 126.8, 123.8, 123.7, 119.4, 117.5, 109.7, 40.3.

IR (KBr): 3055, 2997, 2901, 2218, 1591, 1525, 1491, 1345, 1291, 1178, 1117, 818, 773, 700 cm^{-1}.

HRMS (EI) [$C_{20}H_{16}N_2$] $[M]^+$ calculated: 284.1313, found: 284.1317.

***N*-Methyl-*N*-phenyl-4'-(trifluoromethyl)-[1,1'-biphenyl]-4-amine (4ra)**

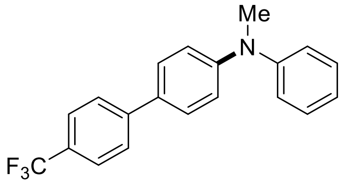

Compound **4ra** was prepared according to the **General procedure E** from **1r** (48.0 mg, 0.2 mmol) and **3a** (64.0 µL, 0.6 mmol), and purified by silica gel column chromatography (*n*-hexane/DCM: 10/1) as a white solid (55.1 mg, 84% yield), m.p. = 121.6 – 122.4 °C.

^{1}H NMR (500 MHz, $CDCl_3$) δ 7.66 (s, 4H), 7.51 (d, *J* = 8.6 Hz, 2H), 7.36 (t, *J* = 7.3 Hz, 2H), 7.17 (d, *J* = 8.3 Hz, 2H), 7.09 (t, *J* = 6.9 Hz, 1H), 7.04 (d, *J* = 7.5 Hz, 2H), 3.38 (s, 3H).

^{13}C NMR (126 MHz, $CDCl_3$) δ 149.3, 148.6, 144.5, 130.9, 129.6, 128.5 (q, *J* = 32.7 Hz), 128.0, 126.7, 125.8, 124.6 (q, *J* = 270.4 Hz), 123.2, 123.1, 118.4, 40.4.

^{19}F NMR (282 MHz, $CDCl_3$) δ -62.73 (s, 3F).

IR (KBr): 3035, 2953, 2889, 2826, 1602, 1532, 1498, 1333, 1250, 1171, 1115, 1073, 1011, 829, 814, 759, 701 cm^{-1}.

HRMS (EI) [$C_{20}H_{16}F_3N$] $[M]^+$ calculated: 327.1235, found: 327.1230.

***N*-Methyl-*N*-phenyl-3'-(trifluoromethyl)-[1,1'-biphenyl]-4-amine (4sa)**

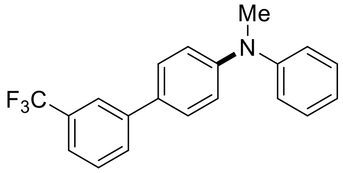

Compound **4sa** was prepared according to the **General procedure E** from **1s** (48.0 mg, 0.2 mmol) and **3a** (64.0 µL, 0.6 mmol), and purified by silica gel column chromatography (*n*-hexane/DCM: 10/1) as a white solid (53.6 mg, 82% yield), m.p. = 80.0 – 80.5 °C.

^{1}H NMR (500 MHz, $CDCl_3$) δ 7.84 (s, 1H), 7.76 (d, *J* = 7.3 Hz, 1H), 7.59 – 7.50 (m, 4H), 7.37 (dd, *J* = 8.5, 7.4 Hz, 2H), 7.18 (dd, *J* = 8.6, 1.0 Hz, 2H), 7.10 (t, *J* = 7.4 Hz, 1H), 7.08 – 7.05 (m, 2H), 3.40 (s, 3H).

^{13}C NMR (126 MHz, $CDCl_3$) δ 149.1, 148.6, 141.8, 131.22, 131.19 (d, *J* = 32.1 Hz), 129.8, 129.6, 129.3, 127.9, 124.4 (q, *J* = 272.9 Hz), 123.3, 123.2, 123.0, 122.7, 118.7, 40.4.

^{19}F NMR (282 MHz, $CDCl_3$) δ -63.06 (s, 3F).

IR (KBr): 3068, 3027, 2940, 2884, 2815, 1591, 1521, 1496, 1440, 1336, 1122, 1072, 1036, 802, 771, 701 cm^{-1}.

HRMS (EI) [$C_{20}H_{16}F_3N$] $[M]^+$ calculated: 327.1235, found: 327.1242.

***N*-Methyl-*N*-phenyl-3',5'-bis(trifluoromethyl)-[1,1'-biphenyl]-4-amine (4ta)**

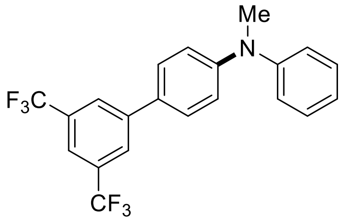

Compound **4ta** was prepared according to the **General procedure E** from **1t** (62.0 mg, 0.2 mmol) and **3a** (64.0 μL, 0.6 mmol), and purified by silica gel column chromatography (*n*-hexane/DCM: 10/1) as a white solid (42.7 mg, 54% yield), m.p. = 41.6 – 42.3 °C.

^{1}H NMR (500 MHz, $CDCl_3$) δ 7.98 (s, 2H), 7.78 (s, 1H), 7.53 – 7.47 (m, 2H), 7.38 (dd, *J* = 8.5, 7.4 Hz, 2H), 7.20 (dd, *J* = 8.6, 1.1 Hz, 2H), 7.14 (t, *J* = 7.4 Hz, 1H), 7.03 (d, *J* = 8.8 Hz, 2H), 3.40 (s, 3H).

^{13}C NMR (126 MHz, $CDCl_3$) δ 149.8, 148.3, 143.1, 132.1 (q, *J* = 33.0 Hz), 129.7, 128.8, 128.0, 126.4, 123.9, 123.7 (q, *J* = 272.7 Hz), 119.9, 117.7, 40.4.

^{19}F NMR (282 MHz, $CDCl_3$) δ -63.32 (s, 6F).

IR (KBr): 3064, 3037, 2956, 2933, 2897, 1594, 1520, 1496, 1467, 1383, 1273, 1168, 1126, 1052, 836, 701 cm^{-1}.

HRMS (EI) [$C_{21}H_{15}F_6N$] $[M]^+$ calculated: 395.1109, found: 395.1096.

4-(Benzo[*d*][1,3]dioxol-5-yl)-*N*-methyl-*N*-phenylaniline (4ua)

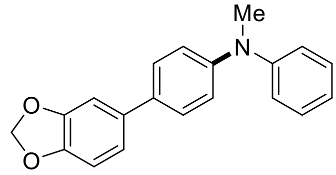

Compound **4ua** was prepared according to the **General procedure E** from **1u** (43.3 mg, 0.2 mmol) and **3a** (64.0 μL, 0.6 mmol), and purified by silica gel column chromatography (*n*-hexane/DCM: 5/1) as a white solid (49.4 mg, 81% yield), m.p. = 98.3 – 99.1 °C.

^{1}H NMR (500 MHz, $CDCl_3$) δ 7.46 (d, *J* = 8.7 Hz, 2H), 7.34 (dd, *J* = 8.6, 7.3 Hz, 2H), 7.13 (dd, *J* = 8.7, 1.1 Hz, 2H), 7.10 – 7.05 (m, 4H), 7.04 (t, *J* = 7.3 Hz, 1H), 6.90 (d, *J* = 8.0 Hz, 1H), 6.01 (s, 2H), 3.38 (s, 3H).

^{13}C NMR (75 MHz, $CDCl_3$) δ 148.9, 148.2, 148.1, 146.6, 135.4, 133.5, 129.4, 127.6, 121.9, 121.2, 120.03, 120.00, 108.6, 107.3, 101.1, 40.4.

IR (KBr): 3033, 2954, 2917, 2823, 1595, 1503, 1437, 1349, 1304, 1251, 1233, 1180, 1106, 1044, 928, 804, 755, 699 cm^{-1}.

HRMS (EI) [$C_{20}H_{17}NO_2$] [M]$^+$ calculated: 303.1259, found: 303.1267.

***N*-Methyl-*N*,*6*-diphenylpyridin-3-amine (4va)**

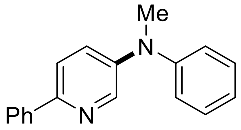

Compound **4va** was prepared according to the **General procedure E** from **1v** (35.0 mg, 0.2 mmol) and **3a** (64.0 μL, 0.6 mmol), and purified by silica gel column chromatography (*n*-hexane/DCM: 1/1) as a white solid (48.4 mg, 93% yield), m.p. = 85.7 – 86.3 °C.

^{1}H NMR (500 MHz, $CDCl_3$) δ 8.44 (dd, *J* = 2.9, 0.7 Hz, 1H), 7.99 (dd, *J* = 8.3, 1.3 Hz, 2H), 7.62 (dd, *J* = 8.6, 0.7 Hz, 1H), 7.47 (t, *J* = 7.7 Hz, 2H), 7.41 – 7.33 (m, 3H), 7.31 (dd, *J* = 8.7, 2.9 Hz, 1H), 7.17 (dd, *J* = 8.7, 1.1 Hz, 2H), 7.11 (t, *J* = 7.4 Hz, 1H), 3.39 (s, 3H).

^{13}C NMR (126 MHz, $CDCl_3$) δ 148.7, 147.8, 143.9, 140.0, 139.3, 129.6, 128.7, 128.0, 126.1, 125.4, 123.3, 122.5, 120.3, 40.1.

IR (KBr): 3065, 3025, 2960, 2884, 2814, 1577, 1556, 1477, 1348, 1228, 1133, 1070, 1007, 907, 830, 734, 699 cm^{-1}.

HRMS (EI) [$C_{18}H_{16}N_2$] [M]$^+$ calculated: 260.1313, found: 260.1325.

***N*-Methyl-*N*-phenyl-4-(pyridin-4-yl)aniline (4wa)**

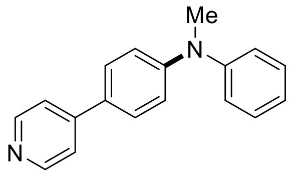

Compound **4wa** was prepared according to the **General procedure E** from **1w** (35.0 mg, 0.2 mmol) and **3a** (64.0 µL, 0.6 mmol), and purified by silica gel column chromatography (*n*-hexane/EtOAc: 4/1) as a light-yellow solid (47.6 mg, 91% yield), m.p. = 109.8 – 110.6 °C.

^{1}H NMR (500 MHz, $CDCl_3$) δ 8.59 (d, *J* = 6.2 Hz, 2H), 7.54 (d, *J* = 8.8 Hz, 2H), 7.49 – 7.44 (m, 2H), 7.37 (dd, *J* = 8.5, 7.4 Hz, 2H), 7.19 (dd, *J* = 8.6, 1.2 Hz, 2H), 7.13 (t, *J* = 7.4 Hz, 1H), 6.99 (d, *J* = 8.8 Hz, 2H), 3.37 (s, 3H).

^{13}C NMR (126 MHz, $CDCl_3$) δ 150.2, 149.9, 148.2, 147.9, 129.6, 128.3, 127.6, 123.9, 123.8, 120.7, 117.3, 40.3.

IR (KBr): 3032, 3003, 2952, 2885, 2821, 1588, 1486, 1345, 1227, 1200, 1124, 990, 811, 766, 703 cm^{-1}.

HRMS (EI) [$C_{18}H_{16}N_2$] [M]$^+$ calculated: 260.1313, found: 260.1320.

***N*-Methyl-*N*,2-diphenylpyridin-4-amine (4xa)**

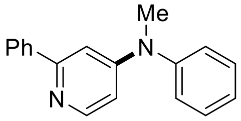

Compound **4xa** was prepared according to the **General procedure E** from **1x** (35.0 mg, 0.2 mmol) and **3a** (64.0 µL, 0.6 mmol), and purified by silica gel column chromatography (*n*-hexane/EtOAc: 4/1) as a colorless oil (48.0 mg, 94% yield), m.p. = 70.2 – 70.7 °C.

^{1}H NMR (500 MHz, $CDCl_3$) δ 8.31 (d, *J* = 5.8 Hz, 1H), 7.86 (dd, *J* = 8.3, 1.4 Hz, 2H), 7.46 – 7.38 (m, 4H), 7.35 (t, *J* = 7.3 Hz, 1H), 7.29 – 7.21 (m, 3H), 6.98 (d, *J* = 2.4 Hz, 1H), 6.53 (dd, *J* = 5.9, 2.5 Hz, 1H), 3.36 (s, 3H).

^{13}C NMR (126 MHz, $CDCl_3$) δ 158.0, 154.6, 149.8, 146.4, 140.4, 130.0, 128.6, 127.0, 126.7, 126.4, 107.2, 105.5, 39.7.

IR (KBr): 3033, 2920, 2854, 1577, 1539, 1493, 1413, 1360, 1248, 1139, 1081, 913, 825, 769, 731, 693 cm^{-1}.

HRMS (EI) [$C_{18}H_{16}N_2$] $[M]^+$ calculated: 260.1313, found: 260.1324.

***N*-Methyl-*N*,5-diphenylpyridin-2-amine (4ya)**

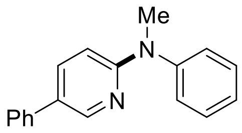

Compound **4ya** was prepared according to the **General procedure E** from **1y** (35.0 mg, 0.2 mmol) and **3a** (64.0 μL, 0.6 mmol), and purified by silica gel column chromatography (*n*-hexane/DCM: 5/1) as a colorless oil (49.1 mg, 94% yield).

^{1}H NMR (300 MHz, $CDCl_3$) δ 8.50 (d, *J* = 2.5 Hz, 1H), 7.59 – 7.46 (m, 3H), 7.39 (d, *J* = 7.0 Hz, 4H), 7.28 (d, *J* = 7.4 Hz, 3H), 7.25 – 7.17 (m, 1H), 6.61 (d, *J* = 8.8 Hz, 1H), 3.52 (s, 3H).

^{13}C NMR (75 MHz, $CDCl_3$) δ 158.1, 146.8, 146.0, 138.5, 135.4, 129.8, 129.0, 126.8, 126.4, 126.2, 125.6, 109.1, 38.6.

IR (KBr): 3060, 3021, 2899, 1607, 1550, 1496, 1381, 1296, 1127, 1079, 1025, 767, 746, 699 cm^{-1}.

HRMS (EI) [$C_{18}H_{16}N_2$] $[M]^+$ calculated: 260.1313, found: 260.1320.

***N*-Methyl-*N*-phenyl-4-(1*H*-pyrrol-1-yl)aniline (4za)**

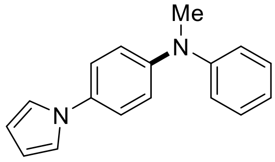

Compound **4za** was prepared according to the **General procedure E** from **1z** (32.2 mg, 0.2 mmol) and **3a** (64.0 μL, 0.6 mmol), and purified by silica gel column chromatography (*n*-hexane/DCM: 10/1) as a white solid (48.6 mg, 97% yield).

^{1}H NMR (300 MHz, $CDCl_3$) δ 7.32 (dd, *J* = 8.3, 6.9 Hz, 4H), 7.12 – 7.03 (m, 6H), 7.00 (td, *J* = 7.3, 1.0 Hz, 1H), 6.39 – 6.31 (m, 2H), 3.35 (s, 3H).

MS(EI): *m/z* 248 $[M]^+$. The chemical shifts were consistent with those reported in the literature.[31]

N,1-Dimethyl-N-phenyl-1H-indol-6-amine (4aaa)

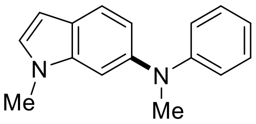

Compound **4aaa** was prepared according to the **General procedure E** from **1aa** (30.0 mg, 0.2 mmol) and **3a** (64.0 μL, 0.6 mmol), and purified by silica gel column chromatography (*n*-hexane/DCM: 4/1) as a light brown solid (40.0 mg, 85% yield), m.p. = 62.4 – 63.1 ^{o}C.

^{1}H NMR (300 MHz, $CDCl_3$) δ 7.56 (d, J = 8.4 Hz, 1H), 7.18 (t, J = 7.8 Hz, 2H), 7.11 (s, 1H), 6.99 (d, J = 3.1 Hz, 1H), 6.94 (dd, J = 8.4, 1.9 Hz, 1H), 6.83 (d, J = 8.2 Hz, 2H), 6.77 (t, J = 7.4 Hz, 1H), 6.45 (d, J = 3.1 Hz, 1H), 3.69 (s, 3H), 3.34 (s, 3H).

^{13}C NMR (176 MHz, $CDCl_3$) δ 150.3, 143.9, 137.5, 129.03, 128.96, 125.6, 121.7, 118.2, 115.9, 105.9, 101.0, 41.0, 32.9.

IR (KBr): 3057, 3020, 2996, 2938, 2874, 1597, 1498, 1473, 1331, 1309, 1254, 1100, 751, 713, 694 cm^{-1}.

HRMS (EI) [$C_{16}H_{16}N_2$] $[M]^+$ calculated: 236.1313, found: 236.1305.

N-Methyl-4-(1-methyl-1H-indol-2-yl)-N-phenylaniline (4aba)

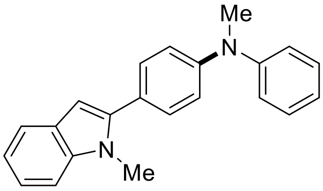

Compound **4aba** was prepared according to the **General procedure E** from **1ab** (45.0 mg, 0.2 mmol) and **3a** (64.0 μL, 0.6 mmol), and purified by silica gel column chromatography (*n*-hexane/DCM: 5/1) as a white solid (61.0 mg, 96% yield), m.p. = 119.9 – 120.7 ^{o}C.

^{1}H NMR (500 MHz, $CDCl_3$) δ 7.69 (dt, J = 7.8, 1.0 Hz, 1H), 7.46 – 7.43 (m, 2H), 7.41 (dd, J = 8.6, 7.4 Hz, 3H), 7.33 – 7.26 (m, 1H), 7.24 (dd, J = 8.6, 1.1 Hz, 2H), 7.23 – 7.16 (m, 1H), 7.14 (tt, J = 7.3, 1.2 Hz, 1H), 7.12 – 7.06 (m, 2H), 6.58 (d, J = 0.9 Hz, 1H), 3.81 (s, 3H), 3.44 (s, 3H).

^{13}C NMR (126 MHz, $CDCl_3$) δ 148.8, 148.6, 141.9, 138.3, 130.2, 129.6, 128.2, 124.2, 123.1, 122.9, 121.4, 120.3, 119.8, 117.9, 109.6, 100.9, 40.3, 31.3.

IR (KBr): 3056, 3036, 2938, 2904, 2882, 1611, 1591, 1561, 1497, 1430, 1343, 1253, 1113, 1067, 821, 782, 735, 694 cm^{-1}.

HRMS (EI) [$C_{22}H_{20}N_2$] $[M]^+$ calculated: 312.1626, found: 312.1632.

4-(Benzofuran-2-yl)-*N*-methyl-*N*-phenylaniline (4aca)

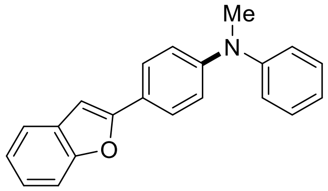

Compound **4aca** was prepared according to the **General procedure E** from **1ac** (42.4 mg, 0.2 mmol) and **3a** (64.0 μL, 0.6 mmol), and purified by silica gel column chromatography (*n*-hexane/DCM: 10/1) as a white solid (53.9 mg, 90% yield), m.p. = 128.5 – 129.1 °C.

^{1}H NMR (500 MHz, $CDCl_3$) δ 7.73 – 7.69 (m, 2H), 7.53 – 7.50 (m, 1H), 7.49 – 7.45 (m, 1H), 7.33 (dd, *J* = 8.5, 7.3 Hz, 2H), 7.24 – 7.17 (m, 2H), 7.14 (dd, *J* = 8.6, 1.1 Hz, 2H), 7.07 (t, *J* = 7.3 Hz, 1H), 6.96 (d, *J* = 8.9 Hz, 2H), 6.82 (d, *J* = 1.0 Hz, 1H), 3.33 (s, 3H).

^{13}C NMR (126 MHz, $CDCl_3$) δ 156.6, 154.8, 149.3, 148.4, 129.7, 129.6, 126.1, 123.6, 123.4, 122.9, 121.9, 120.5, 117.7, 111.0, 99.3, 40.3.

IR (KBr): 3114, 3062, 3034, 2924, 2899, 2830, 1608, 1593, 1492, 1451, 1346, 1256, 1135, 1032, 917, 802, 745, 700 cm^{-1}.

HRMS (EI) [$C_{21}H_{17}NO$] [M]$^+$ calculated: 299.1310, found: 299.1307.

***N*-Methyl-*N*-(*p*-tolyl)-[1,1'-biphenyl]-4-amine (4ab)**

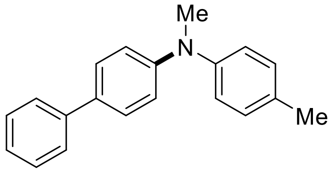

Compound **4ab** was prepared according to the **General procedure E** from **1a** (34.4 mg, 0.2 mmol) and **3b** (75.6 μL, 0.6 mmol), and purified by silica gel column chromatography (*n*-hexane/DCM: 10/1) as a white solid (47.3 mg, 87% yield), m.p. = 132.8 – 133.4 °C.

^{1}H NMR (500 MHz, $CDCl_3$) δ 7.59 (dd, *J* = 8.3, 1.3 Hz, 2H), 7.53 – 7.48 (m, 2H), 7.43 (t, *J* = 7.7 Hz, 2H), 7.31 (t, *J* = 7.4 Hz, 1H), 7.18 (d, *J* = 7.8 Hz, 2H), 7.12 – 7.08 (m, 2H), 7.02 – 6.97 (m, 2H), 3.36 (s, 3H), 2.37 (s, 3H).

^{13}C NMR (126 MHz, $CDCl_3$) δ 148.8, 146.4, 141.1, 132.9, 132.1, 130.2, 128.8, 127.7, 126.6, 126.5, 123.5, 117.6, 40.5, 22.0.

IR (KBr): 3060, 3029, 2916, 2819, 1600, 1510, 1487, 1340, 1255, 1120, 1073, 819, 761, 691 cm^{-1}.

HRMS (EI) [$C_{20}H_{19}N$] [M]$^+$ calculated: 273.1517, found: 273.1519.

N-(3,5-Dimethylphenyl)-N-methyl-[1,1'-biphenyl]-4-amine (4ac)

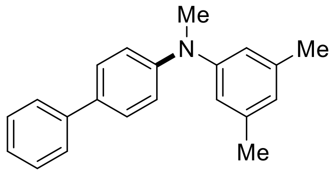

Compound **4ac** was prepared according to the **General procedure E** from **1a** (34.4 mg, 0.2 mmol) and **3c** (85.0 µL, 0.6 mmol), and purified by silica gel column chromatography (*n*-hexane/DCM: 10/1) as a white solid (47.0 mg, 82% yield), m.p. = 92.4 – 93.0 °C.

^{1}H NMR (500 MHz, $CDCl_3$) δ 7.61 – 7.56 (m, 2H), 7.51 (d, *J* = 8.8 Hz, 2H), 7.42 (t, *J* = 7.8 Hz, 2H), 7.30 (t, *J* = 6.8 Hz, 1H), 7.03 (d, *J* = 8.7 Hz, 2H), 6.77 (s, 2H), 6.71 (s, 1H), 3.35 (d, *J* = 1.4 Hz, 3H), 2.38 – 2.16 (m, 6H).

^{13}C NMR (126 MHz, $CDCl_3$) δ 148.9, 148.7, 141.1, 139.1, 132.9, 128.8, 127.8, 126.7, 126.6, 124.4, 120.0, 119.1, 40.5, 21.6.

IR (KBr): 3061, 3026, 2919, 2816, 1593, 1521, 1486, 1348, 1318, 1201, 1091, 841, 759, 722, 690 cm^{-1}.

HRMS (EI) [$C_{21}H_{21}N$] $[M]^+$ calculated: 287.1674, found: 287.1683.

N-Methyl-N-(o-tolyl)-[1,1'-biphenyl]-4-amine (4ad)

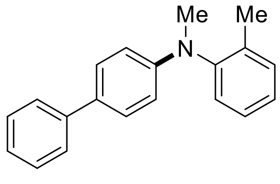

Compound **4ad** was prepared according to the **General procedure E** from **1a** (34.4 mg, 0.2 mmol) and **3d** (72.6 mg, 0.6 mmol), and purified by silica gel column chromatography (*n*-hexane/DCM: 10/1) as a white solid (16.7 mg, 31% yield), m.p. = 99.5 – 101.6 °C.

^{1}H NMR (500 MHz, $CDCl_3$) δ 7.56 – 7.49 (m, 2H), 7.46 – 7.33 (m, 4H), 7.32 – 7.11 (m, 5H), 6.75 – 6.46 (m, 2H), 3.25 (s, 3H), 2.17 (s, 3H).

^{13}C NMR (126 MHz, $CDCl_3$) δ 148.6, 146.7, 141.3, 136.9, 131.5, 129.6, 128.8, 128.5, 127.8, 127.7, 126.7, 126.4, 126.1, 113.1, 39.3, 18.0.

IR (KBr): 3058, 3028, 2921, 2812, 1612, 1578, 1520, 1487, 1345, 1253, 1197, 1118, 1074, 824, 762, 728, 697 cm^{-1}.

HRMS (EI) [$C_{20}H_{19}N$] $[M]^+$ calculated: 273.1517, found: 273.1527.

***N*-Mesityl-*N*-methyl-[1,1'-biphenyl]-4-amine (4ae)**

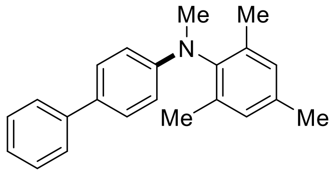

Compound **4ae** was prepared according to the **General procedure E** from **1a** (34.4 mg, 0.2 mmol) and **3e** (94.0 µL, 0.6 mmol), and purified by silica gel column chromatography (*n*-hexane/DCM: 10/1) as a white solid (19.4 mg, 32% yield), m.p. = 119.3 – 119.9 °C.

^{1}H NMR (300 MHz, $CDCl_3$) δ 7.59 – 7.50 (m, 2H), 7.47 – 7.32 (m, 4H), 7.26 – 7.18 (m, 1H), 7.02 – 6.93 (m, 2H), 6.48 (s, 2H), 3.22 (s, 3H), 2.34 (s, 3H), 2.09 (s, 6H).

^{13}C NMR (126 MHz, $CDCl_3$) δ 147.8, 141.5, 141.4, 137.5, 136.7, 129.7, 128.7, 128.66, 127.9, 126.3, 125.9, 37.4, 21.1, 18.0.

IR (KBr): 3072, 3022, 2917, 2809, 1613, 1520, 1486, 1345, 1318, 1250, 1191, 1119, 1073, 816, 761, 695 cm^{-1}.

HRMS (EI) [$C_{22}H_{23}N$] [M]$^+$ calculated:301.1830, found: 301.1823.

***N*-(4-Methoxyphenyl)-*N*-methyl-[1,1'-biphenyl]-4-amine (4af)**

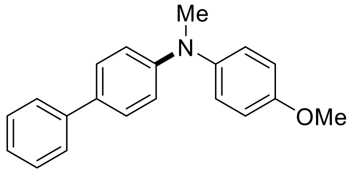

Compound **4af** was prepared according to the **General procedure E** from **1a** (34.4 mg, 0.2 mmol) and **3f** (80.0 µL, 0.6 mmol), and purified by silica gel column chromatography (*n*-hexane/DCM: 5/1) as a white solid (35.1 mg, 61% yield), m.p. = 132.8 – 133.4 °C.

^{1}H NMR (300 MHz, $CDCl_3$) δ 7.56 (d, *J* = 7.5 Hz, 2H), 7.50 – 7.36 (m, 4H), 7.33 – 7.23 (m, 1H), 7.20 – 7.10 (m, 2H), 6.98 – 6.90 (m, 2H), 6.89 – 6.81 (m, 2H), 3.84 (s, 3H), 3.32 (s, 3H).

^{13}C NMR (176 MHz, $CDCl_3$) δ 156.7, 149.2, 142.0, 141.2, 131.0, 128.8, 127.7, 126.8, 126.5, 126.3, 115.6, 115.0, 55.6, 40.7.

IR (KBr): 3035, 3002, 2948, 2932, 2899, 2830, 1607, 1509, 1487, 1454, 1341, 1246, 1109, 1031, 831, 767, 695 cm^{-1}.

HRMS (EI) [$C_{20}H_{19}NO$] [M]$^+$ calculated: 289.1467, found: 289.1461.

N-([1,1'-Biphenyl]-4-yl)-N-methylbenzo[d][1,3]dioxol-5-amine (4ag)

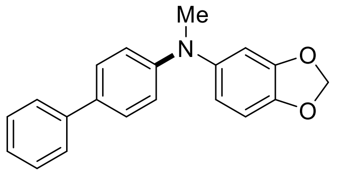

Compound **4ag** was prepared according to the **General procedure E** from **1a** (34.4 mg, 0.2 mmol) and **3g** (72.0 μL, 0.6 mmol), and purified by silica gel column chromatography (*n*-hexane/DCM: 4/1) as a white solid (40.6 mg, 82% yield), m.p. = 131.7 – 132.1 °C.

^{1}H NMR (500 MHz, $CDCl_3$) δ 7.56 (dd, *J* = 8.3, 1.2 Hz, 2H), 7.49 – 7.45 (m, 2H), 7.43 – 7.38 (m, 2H), 7.28 (t, *J* = 7.4 Hz, 1H), 6.88 (d, *J* = 8.8 Hz, 2H), 6.82 (d, *J* = 8.2 Hz, 1H), 6.72 (d, *J* = 2.1 Hz, 1H), 6.66 (dd, *J* = 8.2, 2.2 Hz, 1H), 5.98 (s, 2H), 3.29 (s, 3H).

^{13}C NMR (126 MHz, $CDCl_3$) δ 149.1, 148.5, 144.5, 143.5, 141.2, 131.5, 128.8, 127.7, 126.6, 126.4, 118.1, 116.3, 108.8, 106.8, 101.4, 40.8.

IR (KBr): 3070, 3019, 2926, 2898, 2814, 1604, 1519, 1480, 1446, 1364, 1326, 1220, 1112, 1038, 922, 764, 719, 692 cm^{-1}.

HRMS (EI) [$C_{20}H_{17}NO_2$] $[M]^+$ calculated: 303.1259, found: 303.1273.

N-methyl-N-(4-(trifluoromethoxy)phenyl)-[1,1'-biphenyl]-4-amine (4ah)

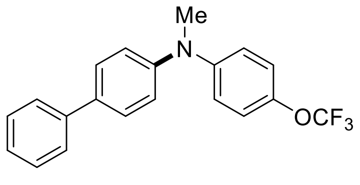

Compound **4ah** was prepared according to the **General procedure E** from **1a** (34.4 mg, 0.2 mmol) and **3h** (90.0 μL, 0.6 mmol), and purified by silica gel column chromatography (*n*-hexane/DCM: 10/1) as a white solid (33.7 mg, 49% yield), m.p. = 123.7– 124.1 °C.

^{1}H NMR (300 MHz, $CDCl_3$) δ 7.64 – 7.49 (m, 4H), 7.43 (t, *J* = 7.5 Hz, 2H), 7.37 – 7.28 (m, 1H), 7.17 – 7.07 (m, 4H), 7.07 – 7.00 (m, 2H), 3.36 (s, 3H).

^{13}C NMR (176 MHz, $CDCl_3$) δ 148.0, 147.7, 143.2, 140.8, 134.9, 128.9, 128.1, 127.0, 126.8, 122.2, 121.3, 120.9, 120.76 (q, *J* = 256.3 Hz), 40.6.

^{19}F NMR (282 MHz, $CDCl_3$) δ -58.67 (s, 3F).

IR (KBr): 3078, 3029, 2940, 2887, 1601, 1507, 1489, 1345, 1267, 1254, 1223, 1198, 1146, 839, 764, 693 cm^{-1}.

HRMS (EI) [$C_{20}H_{16}F_3NO$] $[M]^+$ calculated: 343.1184, found: 343.1198.

N-(4-Chlorophenyl)-*N*-methyl-[1,1'-biphenyl]-4-amine (4ai)

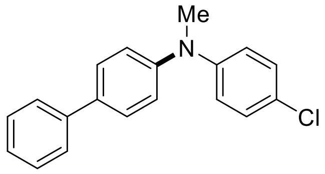

Compound **4ai** was prepared according to the **General procedure E** from **1a** (34.4 mg, 0.2 mmol) and **3i** (72.6 µL, 0.6 mmol), and purified by silica gel column chromatography (*n*-hexane/DCM: 15/1) as a white solid (35.4 mg, 60% yield), m.p. = 134.2 – 135.0 °C.

^{1}H NMR (500 MHz, $CDCl_3$) δ 7.57 (d, *J* = 7.2 Hz, 2H), 7.52 (d, *J* = 6.9 Hz, 2H), 7.42 (t, *J* = 7.0 Hz, 2H), 7.31 (t, *J* = 7.3 Hz, 1H), 7.23 (d, *J* = 7.1 Hz, 2H), 7.07 (d, *J* = 7.8 Hz, 2H), 6.99 (d, *J* = 8.5 Hz, 2H), 3.33 (s, 3H).

^{13}C NMR (126 MHz, $CDCl_3$) δ 148.0, 147.6, 140.8, 134.6, 129.3, 128.9, 128.1, 126.9, 126.8, 126.4, 121.8, 120.9, 40.5.

IR (KBr): 3033, 2886, 1606, 1586, 1518, 1488, 1335, 1252, 1100, 1070, 822, 761, 699 cm^{-1}.

HRMS (EI) [$C_{19}H_{16}ClN$] $[M]^+$ calculated: 293.0971, found: 293.0970.

N-(3-Chlorophenyl)-*N*-methyl-[1,1'-biphenyl]-4-amine (4aj)

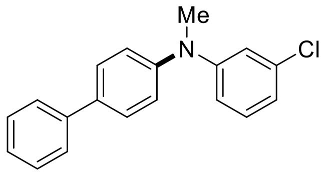

Compound **4aj** was prepared according to the **General procedure E** from **1a** (34.4 mg, 0.2 mmol) and **3j** (72.6 µL, 0.6 mmol), and purified by silica gel column chromatography (*n*-hexane/DCM: 10/1) as a white solid (31.6 mg, 54% yield), m.p. = 97.6 – 98.3 °C.

^{1}H NMR (500 MHz, $CDCl_3$) δ 7.59 (dd, *J* = 8.3, 1.2 Hz, 2H), 7.58 – 7.53 (m, 2H), 7.44 (t, *J* = 7.7 Hz, 2H), 7.33 (t, *J* = 7.4 Hz, 1H), 7.19 – 7.14 (m, 3H), 6.98 (t, *J* = 2.1 Hz, 1H), 6.89 – 6.85 (m, 2H), 3.35 (s, 3H).

^{13}C NMR (126 MHz, $CDCl_3$) δ 150.2, 147.6, 140.7, 135.9, 135.0, 130.2, 128.9, 128.3, 127.1, 126.9, 122.9, 120.3, 118.5, 116.8, 40.4.

IR (KBr): 3052, 3027, 2925, 2905, 2887, 1587, 1554, 1483, 1333, 1247, 1104, 1079, 901, 839, 762,

689 cm^{-1}.

HRMS (EI) [$C_{19}H_{16}ClN$] $[M]^+$ calculated: 293.0971, found: 293.0957.

***N*-(4-Bromophenyl)-*N*-methyl-[1,1'-biphenyl]-4-amine (4ak)**

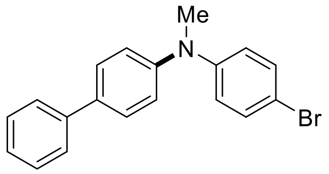

Compound **4ak** was prepared according to the **General procedure E** from **1a** (34.4 mg, 0.2 mmol) and **3k** (112.0 mg, 0.6 mmol), and purified by silica gel column chromatography (*n*-hexane/DCM: 20/1) as a white solid (22.1 mg, 33% yield), m.p. = 131.0 – 132.1 °C.

^{1}H NMR (300 MHz, $CDCl_3$) δ 7.63 – 7.49 (m, 4H), 7.49 – 7.28 (m, 5H), 7.11 (d, *J* = 8.1 Hz, 2H), 6.93 (d, *J* = 8.3 Hz, 2H), 3.34 (s, 3H).

^{13}C NMR (75 MHz, $CDCl_3$) δ 148.0, 147.9, 140.8, 134.9, 132.2, 128.9, 128.1, 127.0, 126.8, 121.7, 121.4, 113.5, 40.4.

IR (KBr): 3059, 3031, 2935, 2886, 2820, 1604, 1583, 1522, 1488, 1341, 1256, 1205, 1123, 1006, 824, 764, 693 cm^{-1}.

HRMS (ESI) [$C_{19}H_{17}BrN$] $[M+H]^+$ calculated: 338.0544, found: 338.0529.

***N*-(3,5-Dimethylphenyl)-*N*-methyl-[1,1'-biphenyl]-3-amine (4bc)**

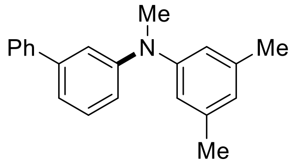

Compound **4bc** was prepared according to the **General procedure E** from **1b** (34.4 mg, 0.2 mmol) and **3c** (85.0 µL, 0.6 mmol), and purified by silica gel column chromatography (*n*-hexane/DCM: 10/1) as a light-yellow oil (43.5 mg, 76% yield).

^{1}H NMR (700 MHz, $CDCl_3$) δ 7.59 – 7.55 (m, 2H), 7.45 – 7.40 (m, 2H), 7.37 – 7.31 (m, 2H), 7.22 (t, *J* = 2.1 Hz, 1H), 7.18 – 7.14 (m, 1H), 7.00 – 6.96 (m, 1H), 6.75 (s, 2H), 6.68 (s, 1H), 3.35 (s, 3H), 2.29 (s, 3H).

^{13}C NMR (176 MHz, $CDCl_3$) δ 149.7, 149.1, 142.4, 141.6, 139.0, 129.5, 128.8, 127.4, 127.3, 123.9, 119.7, 119.3, 118.7, 118.5, 40.6, 21.6.

IR (KBr): 3031, 2916, 2812, 1588, 1568, 1482, 1417, 1346, 1203, 1130, 991, 848, 756, 698 cm^{-1}.

HRMS (ESI) [$C_{21}H_{22}N$] $[M+H]^+$ calculated: 288.1752, found: 288.1747.

***N*-Methyl-*N*-(*o*-tolyl)-[1,1'-biphenyl]-2-amine (4cd)**

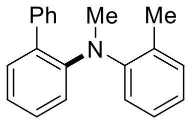

Compound **4cd** was prepared according to the **General procedure E** from **1c** (34.4 mg, 0.2 mmol) and **3d** (72.6 mg, 0.6 mmol), and purified by silica gel column chromatography (*n*-hexane/DCM: 15/1) as a white solid (12.1 mg, 22% yield), m.p. = 93.7 – 94.2 °C.

^{1}H NMR (500 MHz, $CDCl_3$) δ 7.38 (dd, *J* = 8.2, 1.3 Hz, 2H), 7.26 – 7.20 (m, 4H), 7.20 – 7.16 (m, 1H), 7.06 (qd, *J* = 7.3, 1.2 Hz, 2H), 6.96 (dd, *J* = 8.0, 1.0 Hz, 2H), 6.90 – 6.85 (m, 2H), 2.93 (s, 3H), 2.02 (s, 3H).

^{13}C NMR (126 MHz, $CDCl_3$) δ 149.4, 149.0, 141.5, 135.1, 132.5, 132.1, 131.4, 128.8, 128.2, 128.0, 126.6, 126.5, 123.2, 123.0, 122.4, 121.2, 41.2, 19.4.

IR (KBr): 3059, 3014, 2966, 2929, 2871, 1593, 1477, 1434, 1278, 1127, 1053, 775, 760, 699 cm^{-1}.

HRMS (EI) [$C_{20}H_{19}N$] $[M]^+$ calculated: 273.1517, found: 273.1521.

***N*-Methyl-*N*-(4-(trifluoromethoxy)phenyl)naphthalen-1-amine (4dh)**

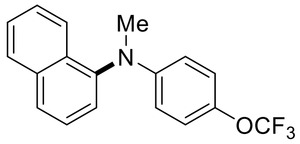

Compound **4dh** was prepared according to the **General procedure E** from **1d** (26.0 μL, 0.2 mmol) and **3h** (90.0 μL, 0.6 mmol), and purified by silica gel column chromatography (*n*-hexane/DCM: 10/1) as a colorless oil (34.2 mg, 54% yield).

^{1}H NMR (500 MHz, $CDCl_3$) δ 7.94 (d, *J* = 8.1 Hz, 1H), 7.88 – 7.80 (m, 2H), 7.56 – 7.50 (m, 2H), 7.47 (ddd, *J* = 8.2, 6.8, 1.4 Hz, 1H), 7.37 (dd, *J* = 7.3, 1.2 Hz, 1H), 7.02 (dd, *J* = 9.3, 0.9 Hz, 2H), 6.59 – 6.53 (m, 2H), 3.40 (s, 3H).

^{13}C NMR (126 MHz, $CDCl_3$) δ 149.0, 145.0, 140.5, 135.3, 131.2, 128.7, 127.2, 126.7, 126.6, 126.5, 125.5, 123.7, 122.1, 120.9 (q, *J* = 255.4 Hz), 113.8, 40.5.

^{19}F NMR (282 MHz, $CDCl_3$) δ -58.91 (s, 3F).

IR (KBr): 3051, 3027, 2951, 1611, 1595, 1505, 1474, 1394, 1336, 1268, 1204, 1160, 1115, 1016,

832, 775 cm^{-1}.

HRMS (EI) [$C_{18}H_{14}F_3NO$] [M]$^+$ calculated: 317.1027, found: 317.1035.

***N*-(4-methoxyphenyl)-*N*,3,5-trimethylaniline (4gc)**

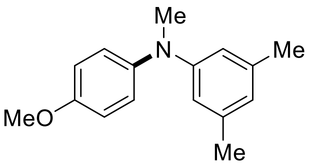

Compound **4gc** was prepared according to the **General procedure E** from **1g** (45.0 μL, 0.4 mmol) and **3c** (170.0 μL, 1.2 mmol), and purified by silica gel column chromatography (*n*-hexane/DCM: 5/1) as a light-yellow solid (65.7 mg, 68% yield), m.p. = 50.0 – 51.2 °C.

^{1}H NMR (700 MHz, $CDCl_3$) δ 7.13 – 6.99 (m, 2H), 6.91 – 6.86 (m, 2H), 6.47 (s, 1H), 6.44 (s, 2H), 3.83 (s, 3H), 3.24 (s, 3H), 2.24 (s, 6H).

^{13}C NMR (176 MHz, $CDCl_3$) δ 156.2, 150.0, 142.6, 138.7, 126.1, 120.6, 114.8, 114.0, 55.6, 40.7, 21.7.

IR (KBr): 3006, 2950, 2912, 2831, 1595, 1508, 1476, 1353, 1298, 1243, 1205, 1185, 1106, 1098, 1035, 839, 820, 693 cm^{-1}.

HRMS (ESI) [$C_{16}H_{20}NO$] [M+H]$^+$ calculated: 242.1545, found: 242.1544.

***N*-Methyl-4-(trifluoromethoxy)-*N*-(4-(trifluoromethyl)phenyl)aniline (4hh)**

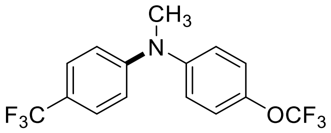

Compound **4hh** was prepared according to the **General procedure E** from **1h** (51.0 μL, 0.4 mmol) and **3h** (180.0 μL, 1.2 mmol), and purified by silica gel column chromatography (*n*-hexane/DCM: 10/1) as a light-yellow oil (60.4 mg, 45% yield).

^{1}H NMR (700 MHz, $CDCl_3$) δ 7.54 – 7.39 (m, 2H), 7.27 – 7.12 (m, 4H), 6.93 – 6.83 (m, 2H), 3.35 (s, 3H).

^{13}C NMR (176 MHz, $CDCl_3$) δ 151.3, 146.6, 145.7, 126.5 (q, J = 3.9 Hz), 125.8, 124.8 (q, J = 270.7 Hz), 122.6, 121.1 (q, J = 32.8 Hz), 119.9 (q, J = 256.9 Hz), 116.0, 40.4.

^{19}F NMR (282 MHz, $CDCl_3$) δ -58.46 (s, 3F), -61.80 (s, 3F).

IR (KBr): 3052, 2954, 2893, 2825, 1620, 1505, 1333, 1270, 1208, 1120, 1078, 1062, 828 cm^{-1}.

HRMS (ESI) [$C_{15}H_{12}F_6NO$] $[M+H]^+$ calculated: 336.0825, found: 336.0829.

***N*-(4-Chlorophenyl)-*N*,4'-dimethyl-[1,1'-biphenyl]-4-amine (4li)**

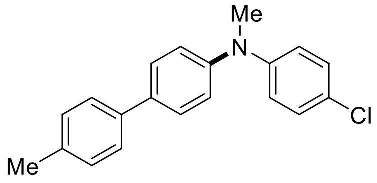

Compound **4li** was prepared according to the **General procedure E** from **1l** (37.2 mg, 0.2 mmol) and **3i** (72.6 μL, 0.6 mmol), and purified by silica gel column chromatography (*n*-hexane/DCM: 10/1) as a white solid (42.6 mg, 69% yield), m.p. = 136.3 – 136.9 °C.

^{1}H NMR (500 MHz, $CDCl_3$) δ 7.54 – 7.50 (m, 2H), 7.48 (d, J = 8.1 Hz, 2H), 7.25 – 7.21 (m, 4H), 7.12 – 7.05 (m, 2H), 7.03 – 6.92 (m, 2H), 3.33 (s, 3H), 2.40 (s, 3H).

^{13}C NMR (126 MHz, $CDCl_3$) δ 147.8, 147.6, 137.9, 136.7, 134.8, 129.6, 129.3, 127.9, 126.6, 126.1, 121.3, 121.2, 40.5, 21.2.

IR (KBr): 3031, 2917, 2822, 1605, 1590, 1485, 1341, 1253, 1181, 1131, 1104, 1004, 803, 753, 686 cm^{-1}.

HRMS (EI) [$C_{20}H_{18}ClN$] $[M]^+$ calculated: 307.1128, found: 307.1139.

***N*,4'-Dimethyl-*N*-(4-(trifluoromethoxy)phenyl)-[1,1'-biphenyl]-4-amine (4lh)**

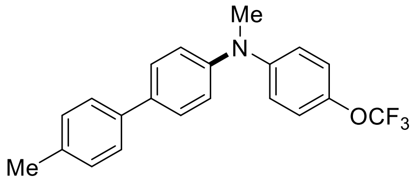

Compound **4lh** was prepared according to the **General procedure E** from **1l** (37.2 mg, 0.2 mmol) and **3h** (90.0 μL, 0.6 mmol), and purified by silica gel column chromatography (*n*-hexane/DCM: 10/1) as a white solid (38.8 mg, 54% yield), m.p. = 127.4 – 128.1 °C.

^{1}H NMR (500 MHz, $CDCl_3$) δ 7.57 – 7.51 (m, 2H), 7.49 (d, J = 8.2 Hz, 2H), 7.26 (d, J = 7.8 Hz, 2H), 7.17 – 7.09 (m, 4H), 7.06 – 6.97 (m, 2H), 3.36 (s, 3H), 2.41 (s, 3H).

^{13}C NMR (126 MHz, $CDCl_3$) δ 147.8, 143.0, 137.9, 136.7, 135.1, 129.6, 128.0, 126.7, 122.2, 121.8, 121.7, 120.8 (q, J = 256.0 Hz), 120.4, 40.6, 21.2.

^{19}F NMR (282 MHz, $CDCl_3$) δ -58.72 (s, 3F).

IR (KBr): 3024, 2925, 2883, 2819, 1603, 1497, 1343, 1288, 1196, 1155, 1107, 844, 810, 509 cm^{-1}.

HRMS (EI) [$C_{21}H_{18}F_3NO$] [M]$^+$ calculated: 357.1340, found: 357.1355.

4'-Methoxy-*N*-(4-methoxyphenyl)-*N*-methyl-[1,1'-biphenyl]-4-amine (4mf)

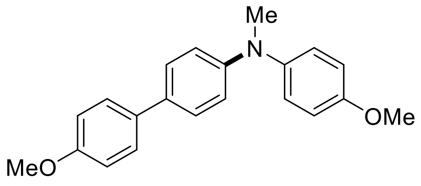

Compound **4mf** was prepared according to the **General procedure E** from **1m** (40.4 mg, 0.2 mmol) and **3f** (82.0 mg, 0.6 mmol), and purified by silica gel column chromatography (*n*-hexane/DCM: 3/1) as a white solid (42.0 mg, 66% yield), m.p. = 170.0 – 170.5 °C.

^{1}H NMR (500 MHz, $CDCl_3$) δ 7.50 – 7.46 (m, 2H), 7.43 – 7.39 (m, 2H), 7.16 – 7.11 (m, 2H), 6.97 – 6.94 (m, 2H), 6.93 – 6.90 (m, 2H), 6.86 – 6.83 (m, 2H), 3.84 (s, 3H), 3.83 (s, 3H), 3.30 (s, 3H).

^{13}C NMR (126 MHz, $CDCl_3$) δ 158.5, 156.5, 148.8, 142.2, 133.9, 130.9, 127.5, 127.3, 126.5, 115.9, 114.9, 114.2, 55.6, 55.5, 40.7.

IR (KBr): 3035, 3012, 2951, 2912, 2836, 1606, 1505, 1441, 1344, 1246, 1182, 1110, 1030, 831, 787 cm^{-1}.

HRMS (EI) [$C_{21}H_{21}NO_2$] [M]$^+$ calculated: 319.1572, found: 319.1586.

4'-Methoxy-*N*-methyl-*N*-(4-(trifluoromethoxy)phenyl)-[1,1'-biphenyl]-4-amine (4mh)

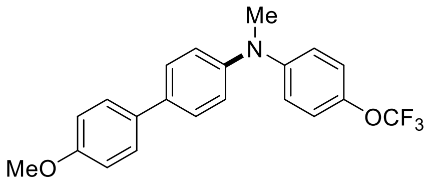

Compound **4mh** was prepared according to the **General procedure E** from **1m** (40.4 mg, 0.2 mmol) and **3h** (90.0 μL, 0.6 mmol), and purified by silica gel column chromatography (*n*-hexane/DCM: 5/1) as a white solid (35.0 mg, 47% yield), m.p. = 127.4 – 128.1 °C.

^{1}H NMR (500 MHz, $CDCl_3$) δ 7.51 (td, *J* = 6.6, 2.1 Hz, 4H), 7.15 – 7.09 (m, 4H), 7.02 – 6.95 (m, 4H), 3.86 (s, 3H), 3.35 (s, 3H).

^{13}C NMR (126 MHz, $CDCl_3$) δ 159.0, 147.8, 147.5, 142.9, 135.0, 133.4, 127.9, 127.7, 122.2, 122.0, 120.8 (q, *J* = 256.1 Hz), 120.1, 114.3, 55.5, 40.6.

^{19}F NMR (282 MHz, $CDCl_3$) δ -58.70 (s, 3F).

IR (KBr): 3063, 3037, 2952, 2908, 2834, 1607, 1579, 1509, 1353, 1181, 1041, 1015, 820 cm^{-1}.

HRMS (EI) [$C_{21}H_{18}F_3NO_2$] [M]$^+$ calculated: 373.1290, found: 373.1298.

4'-Chloro-*N*-(4-chlorophenyl)-*N*-methyl-[1,1'-biphenyl]-4-amine (4oi)

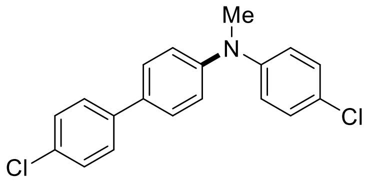

Compound **4oi** was prepared according to the **General procedure E** from **1o** (41.2 mg, 0.2 mmol) and **3i** (72.6 μL, 0.6 mmol), and purified by silica gel column chromatography (*n*-hexane/DCM: 10/1) as a white solid (60.6 mg, 92% yield), m.p. = 170.8 – 171.3 °C.

^{1}H NMR (500 MHz, $CDCl_3$) δ 7.52 – 7.45 (m, 4H), 7.41 – 7.36 (m, 2H), 7.28 – 7.23 (m, 2H), 7.09 – 7.03 (m, 2H), 7.04 – 6.99 (m, 2H), 3.34 (s, 3H).

^{13}C NMR (126 MHz, $CDCl_3$) δ 148.3, 147.4, 139.3, 132.9, 132.8, 129.4, 129.0, 127.9, 127.8, 126.9, 122.4, 120.3, 40.5.

IR (KBr): 3082, 3037, 2937, 2891, 1606, 1586, 1520, 1485, 1342, 1254, 1124, 1093, 1007, 824, 730 cm^{-1}.

HRMS (EI) [$C_{19}H_{15}Cl_2N$] [M]$^+$ calculated: 327.0582, found: 327.0582.

4'-((4-Chlorophenyl)(methyl)amino)-[1,1'-biphenyl]-4-carbonitrile (4qi)

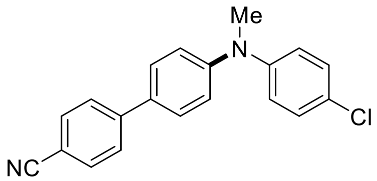

Compound **4qi** was prepared according to the **General procedure E** from **1q** (39.4 mg, 0.2 mmol) and **3i** (72.6 μL, 0.6 mmol), and purified by silica gel column chromatography (*n*-hexane/DCM: 5/1) as a white solid (55.4 mg, 87% yield), m.p. = 139.5 – 140.3 °C.

^{1}H NMR (500 MHz, $CDCl_3$) δ 7.70 – 7.62 (m, 4H), 7.54 – 7.48 (m, 2H), 7.32 – 7.28 (m, 2H), 7.10 – 7.06 (m, 2H), 7.04 – 7.00 (m, 2H), 3.36 (s, 3H).

^{13}C NMR (126 MHz, $CDCl_3$) δ 149.2, 146.9, 145.2, 132.7, 130.6, 129.6, 128.2, 128.0, 126.9, 124.1, 119.3, 118.6, 109.9, 40.3.

IR (KBr): 3035, 2935, 2915, 2814, 1586, 1522, 1485, 1397, 1336, 1258, 1184, 1123, 1111, 1002, 837, 803, 748, 718, 677 cm^{-1}.

HRMS (EI) [$C_{20}H_{15}ClN_2$] $[M]^+$ calculated: 318.0924, found: 318.0915.

***N*-(4-Methoxyphenyl)-*N*-methyl-4'-(trifluoromethyl)-[1,1'-biphenyl]-4-amine (4rf)**

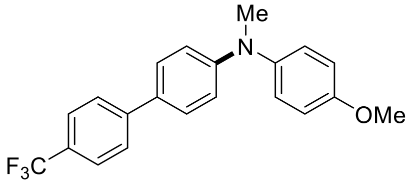

Compound **4rf** was prepared according to the **General procedure E** from **1r** (48.0 mg, 0.2 mmol) and **3f** (82.0 mg, 0.6 mmol), and purified by silica gel column chromatography (*n*-hexane/DCM: 5/1) as a white solid (63.5 mg, 89% yield), m.p. = 139.9 – 140.5 °C.

^{1}H NMR (500 MHz, $CDCl_3$) δ 7.65 (s, 4H), 7.51 – 7.46 (m, 2H), 7.21 – 7.14 (m, 2H), 6.98 – 6.94 (m, 2H), 6.89 – 6.81 (m, 2H), 3.86 (s, 3H), 3.33 (s, 3H).

^{13}C NMR (126 MHz, $CDCl_3$) δ 157.1, 149.9, 144.6, 141.5, 128.8, 128.1 (d, *J* = 32.5 Hz), 128.0, 127.8, 127.4, 126.4, 125.7, 124.6 (d, *J* = 271.8 Hz), 115.1, 55.6, 40.6.

^{19}F NMR (282 MHz, $CDCl_3$) δ -62.70 (s, 3F).

IR (KBr): 3099, 3026, 2955, 2898, 1602, 1513, 1332, 1282, 1253, 1210, 1124, 1074, 1032, 835, 816, 729, 598 cm^{-1}.

HRMS (EI) [$C_{21}H_{18}F_3NO$] $[M]^+$ calculated: 357.1340, found: 357.1346.

***N*-Methyl-*N*-(4-(trifluoromethoxy)phenyl)-4'-(trifluoromethyl)-[1,1'-biphenyl]-4-amine (4rh)**

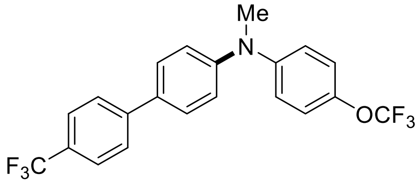

Compound **4rh** was prepared according to the **General procedure E** from **1r** (48.0 mg, 0.2 mmol) and **3h** (90.0 µL, 0.6 mmol), and purified by silica gel column chromatography (*n*-hexane/DCM: 10/1) as a white solid (74.5 mg, 91% yield), m.p. = 111.3 – 112.0 °C.

^{1}H NMR (500 MHz, $CDCl_3$) δ 7.68 (s, 4H), 7.57 – 7.53 (m, 2H), 7.19 (d, *J* = 8.0 Hz, 2H), 7.15 – 7.07 (m, 4H), 3.38 (s, 3H).

^{13}C NMR (126 MHz, $CDCl_3$) δ 148.9, 147.4, 144.3, 144.1, 132.3, 128.8 (q, *J* = 32.7 Hz), 128.2, 126.8, 125.9, 124.54 (q, *J* = 271.7 Hz), 122.6, 122.4, 119.9, 119.7 (q, *J* = 256.1 Hz), 40.5.

^{19}F NMR (282 MHz, $CDCl_3$) δ -58.50 (s, 3F), -62.61 (s, 3F).

IR (KBr): 3053, 3033, 2951, 2890, 1602, 1531, 1508, 1350, 1328, 1273, 1163, 1130, 1012, 854, 820 cm^{-1}.

HRMS (EI) [$C_{21}H_{15}F_6NO$] $[M]^+$ calculated: 411.1058, found: 411.1047.

***N*-(4-Chlorophenyl)-*N*-methyl-3'-(trifluoromethyl)-[1,1'-biphenyl]-4-amine (4si)**

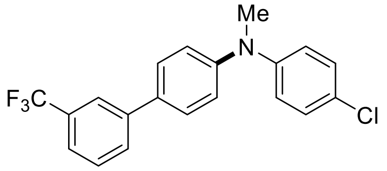

Compound **4si** was prepared according to the **General procedure E** from **1s** (48.0 mg, 0.2 mmol) and **3i** (72.6 μL, 0.6 mmol), and purified by silica gel column chromatography (*n*-hexane/DCM: 10/1) as a white solid (62.4 mg, 86% yield), m.p. = 106.8 – 107.4 °C.

^{1}H NMR (500 MHz, $CDCl_3$) δ 7.82 (s, 1H), 7.75 (d, *J* = 7.3 Hz, 1H), 7.60 – 7.49 (m, 4H), 7.31 – 7.26 (m, 2H), 7.11 – 7.01 (m, 4H), 3.36 (s, 3H).

^{13}C NMR (126 MHz, $CDCl_3$) δ 148.7, 147.3, 141.6, 132.3, 131.2 (q, *J* = 32.0 Hz), 129.9, 129.5, 129.3, 128.1, 127.3, 123.4, 123.3 (q, *J* = 271.8 Hz), 122.9, 119.9, 40.4.

^{19}F NMR (282 MHz, $CDCl_3$) δ -63.07 (s, 3F).

IR (KBr): 3036, 2893, 1604, 1586, 1521, 1492, 1444, 1335, 1253, 1166, 1125, 1073, 1034, 833, 801, 694 cm^{-1}.

HRMS (EI) [$C_{20}H_{15}ClF_3N$] $[M]^+$ calculated: 361.0845, found: 361.0840.

***N*-(3-Chlorophenyl)-*N*-methyl-3',5'-bis(trifluoromethyl)-[1,1'-biphenyl]-4-amine (4tj)**

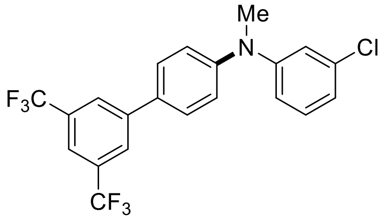

Compound **4tj** was prepared according to the **General procedure E** from **1t** (62.0 mg, 0.2 mmol) and **3j** (72.6 μL, 0.6 mmol), and purified by silica gel column chromatography (*n*-hexane/DCM: 10/1) as a white solid (72.8 mg, 84% yield), m.p. = 68.1 – 68.9 °C.

^{1}H NMR (700 MHz, $CDCl_3$) δ 8.01 – 7.98 (m, 2H), 7.84 – 7.80 (m, 1H), 7.58 – 7.53 (m, 2H), 7.24 (t, *J* = 8.1 Hz, 1H), 7.16 – 7.12 (m, 2H), 7.09 (t, *J* = 2.1 Hz, 1H), 7.02 – 6.96 (m, 2H), 3.38 (s, 3H).

^{13}C NMR (176 MHz, $CDCl_3$) δ 149.7, 149.1, 142.9, 135.1, 132.2 (q, *J* = 33.0 Hz), 131.1, 130.4,

128.3, 126.6, 123.6 (q, J = 272.7 Hz), 122.2, 121.1, 120.7, 119.3, 100.0, 40.4.

^{19}F NMR (282 MHz, $CDCl_3$) δ -63.32 (s, 6F).

IR (KBr): 3071, 3043, 2960, 2882, 2818, 1590, 1521, 1485, 1381, 1278, 1260, 1168, 1134, 1051, 897, 838, 770, 683 cm^{-1}.

HRMS (EI) [$C_{20}H_{14}ClF_6N$] [M]$^+$ calculated: 429.0719, found: 429.0722.

_N_-(4-Chlorophenyl)-_N_-methyl-6-phenylpyridin-3-amine (4vi)

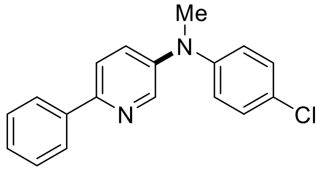

Compound **4vi** was prepared according to the **General procedure E** from **1v** (35.0 mg, 0.2 mmol) and **3i** (72.6 μL, 0.6 mmol), and purified by silica gel column chromatography (*n*-hexane/DCM: 5/1) as a white solid (57.4 mg, 97% yield), m.p. = 88.5 – 89.2 ^{o}C.

^{1}H NMR (500 MHz, $CDCl_3$) δ 8.42 (dd, J = 2.9, 0.7 Hz, 1H), 7.96 (dd, J = 8.4, 1.3 Hz, 2H), 7.63 (dd, J = 8.7, 0.8 Hz, 1H), 7.46 (t, J = 7.6 Hz, 2H), 7.37 (t, J = 7.4 Hz, 1H), 7.32 (dd, J = 8.7, 2.9 Hz, 1H), 7.30 – 7.26 (m, 2H), 7.07 – 7.01 (m, 2H), 3.35 (s, 3H).

^{13}C NMR (126 MHz, $CDCl_3$) δ 149.8, 146.6, 143.6, 141.1, 139.1, 129.6, 128.8, 128.3, 127.8, 126.6, 126.3, 122.8, 120.5, 40.2.

IR (KBr): 3054, 3029, 2952, 2894, 1580, 1556, 1481, 1445, 1340, 1088, 1010, 835, 777, 736, 694 cm^{-1}.

HRMS (EI) [$C_{18}H_{15}ClN_2$] [M]$^+$ calculated: 294.0924, found: 294.0927.

3-Chloro-_N_-methyl-_N_-(4-(pyridin-4-yl)phenyl)aniline (4wj)

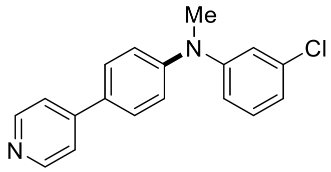

Compound **4wj** was prepared according to the **General procedure E** from **1w** (35.0 mg, 0.2 mmol) and **3j** (72.6 μL, 0.6 mmol), and purified by silica gel column chromatography (*n*-hexane/EtOAc: 4/1) as a light-yellow oil (56.6 mg, 96% yield).

^{1}H NMR (700 MHz, $CDCl_3$) δ 8.62 (d, J = 5.9 Hz, 2H), 7.61 – 7.55 (m, 2H), 7.52 – 7.43 (m, 2H),

7.24 – 7.17 (m, 1H), 7.14 – 7.03 (m, 3H), 7.01 – 6.91 (m, 2H), 3.36 (s, 3H).

^{13}C NMR (176 MHz, $CDCl_3$) δ 150.3, 149.6, 149.2, 147.7, 135.1, 130.7, 130.4, 128.0, 122.2, 121.2, 121.0, 120.4, 119.4, 40.3.

IR (KBr): 3033, 3022, 2906, 2886, 1579, 1523, 1484, 1404, 1343, 1248, 1229, 1137, 1118, 1084, 988, 905, 809, 776, 713 cm^{-1}.

HRMS (ESI) [$C_{18}H_{16}ClN_2$] $[M+H]^+$ calculated: 295.1002, found: 295.1006.

***N*-(4-(1*H*-Pyrrol-1-yl)phenyl)-*N*-methylbenzo[*d*][1,3]dioxol-5-amine (4zg)**

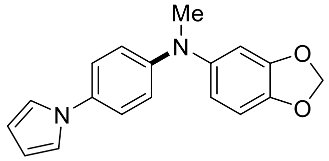

Compound **4zg** was prepared according to the **General procedure E** from **1z** (32.2 mg, 0.2 mmol) and **3g** (72.0 μL, 0.6 mmol), and purified by silica gel column chromatography (*n*-hexane/DCM: 4/1) as a white solid (37.9 mg, 65% yield), m.p. = 119.7 – 120.5 °C.

^{1}H NMR (500 MHz, $CDCl_3$) δ 7.27 – 7.22 (m, 2H), 7.01 (t, *J* = 2.2 Hz, 2H), 6.89 – 6.84 (m, 2H), 6.81 (d, *J* = 8.2 Hz, 1H), 6.68 (d, *J* = 2.2 Hz, 1H), 6.63 (dd, *J* = 8.2, 2.2 Hz, 1H), 6.32 (t, *J* = 2.2 Hz, 2H), 5.98 (s, 2H), 3.27 (s, 3H).

^{13}C NMR (126 MHz, $CDCl_3$) δ 148.5, 147.9, 144.3, 143.6, 133.1, 122.0, 119.8, 117.5, 117.2, 109.7, 108.8, 106.3, 101.4, 41.0.

IR (KBr): 3040, 2951, 2897, 1609, 1522, 1486, 1322, 1260, 1217, 1126, 1038, 923, 829, 720, 630 cm^{-1}.

HRMS (EI) [$C_{18}H_{16}N_2O_2$] $[M]^+$ calculated: 292.1212, found: 292.1222.

***N*-Methyl-*N*-(4-(1-methyl-1*H*-indol-2-yl)phenyl)benzo[*d*][1,3]dioxol-5-amine (4abg)**

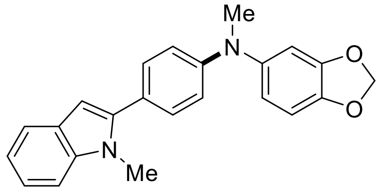

Compound **4abg** was prepared according to the **General procedure E** from **1ab** (45.0 mg, 0.2 mmol) and **3g** (72.0 μL, 0.6 mmol), and purified by silica gel column chromatography (*n*-hexane/DCM: 5/1) as a white solid (70.0 mg, 98% yield), m.p. = 127.1 – 128.2 °C.

^{1}H NMR (700 MHz, $CDCl_3$) δ 7.61 (d, *J* = 7.8 Hz, 1H), 7.34 (t, *J* = 8.0 Hz, 3H), 7.24 – 7.19 (m,

1H), 7.13 (t, J = 7.4 Hz, 1H), 6.86 (d, J = 8.4 Hz, 2H), 6.83 (d, J = 8.2 Hz, 1H), 6.74 (d, J = 1.6 Hz, 1H), 6.69 (dd, J = 8.2, 1.8 Hz, 1H), 6.49 (s, 1H), 5.97 (s, 2H), 3.73 (s, 3H), 3.30 (s, 3H).

^{13}C NMR (176 MHz, $CDCl_3$) δ 149.3, 148.5, 144.9, 143.0, 142.1, 138.2, 130.1, 128.2, 122.5, 121.2, 120.2, 119.8, 118.8, 115.0, 109.5, 108.9, 107.3, 101.5, 100.6, 40.7, 31.2.

IR (KBr): 3056, 2955, 2887, 1607, 1478, 1435, 1361, 1338, 1303, 1246, 1214, 1188, 1110, 1037, 937, 821, 778, 755 cm^{-1}.

HRMS (ESI) [$C_{23}H_{21}N_2O_2$] $[M+H]^+$ calculated: 357.1603, found: 357.1611.

N-Ethyl-*N*-phenyl-[1,1'-biphenyl]-4-amine (4al)

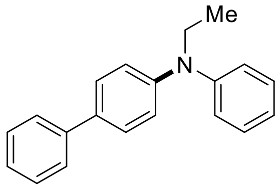

Compound **4al** was prepared according to the **General procedure E** from **1a** (34.4 mg, 0.2 mmol) and **3l** (76.0 μL, 0.6 mmol), and purified by silica gel column chromatography (*n*-hexane/DCM: 10/1) as a white solid (15.0 mg, 27% yield), m.p. = 64.3 – 64.8 °C.

^{1}H NMR (500 MHz, $CDCl_3$) δ 7.63 – 7.56 (m, 2H), 7.52 (dd, J = 8.5, 1.4 Hz, 2H), 7.47 – 7.40 (m, 2H), 7.37 – 7.28 (m, 3H), 7.12 (d, J = 7.9 Hz, 2H), 7.08 – 7.00 (m, 3H), 3.85 (q, J = 7.0 Hz, 2H), 1.28 (td, J = 7.1 Hz, 3H).

^{13}C NMR (126 MHz, $CDCl_3$) δ 147.6, 147.2, 141.0, 133.2, 129.5, 128.8, 127.9, 126.6, 122.3, 122.1, 119.9, 46.6, 12.9.

IR (KBr): 3050, 3032, 2966, 2925, 2870, 1589, 1522, 1486, 1372, 1353, 1244, 1125, 1097, 832, 762, 745, 689 cm^{-1}.

HRMS (EI) [$C_{20}H_{19}N$] $[M]^+$ calculated: 273.1517, found: 273.1529.

N-Butyl-*N*-phenyl-[1,1'-biphenyl]-4-amine (4am)

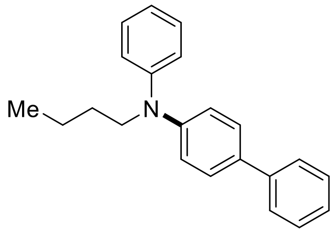

Compound **4am** was prepared according to the **General procedure E** from **1a** (34.4 mg, 0.2 mmol) and **3m** (96.0 μL, 0.6 mmol), and purified by silica gel column chromatography (*n*-hexane/DCM:

20/1) as a white solid (16.1 mg, 27% yield), m.p. = 80.8 – 81.3 °C.

^{1}H NMR (500 MHz, $CDCl_3$) δ 7.58 (d, J = 8.1 Hz, 2H), 7.49 (d, J = 8.1 Hz, 2H), 7.42 (t, J = 7.5 Hz, 2H), 7.30 (q, J = 7.4 Hz, 3H), 7.09 (d, J = 8.5 Hz, 2H), 7.02 (d, J = 7.7 Hz, 3H), 3.74 (t, J = 7.4 Hz, 2H), 1.70 (p, J = 7.8 Hz, 2H), 1.40 (h, J = 7.4 Hz, 2H), 0.95 (t, J = 7.4 Hz, 3H).

^{13}C NMR (126 MHz, $CDCl_3$) δ 148.0, 147.7, 141.0, 133.1, 129.5, 128.8, 127.9, 126.7, 122.3, 122.0, 119.9, 52.3, 29.8, 20.5, 14.1.

IR (KBr): 3057, 3033, 2960, 2862, 1587, 1457, 1361, 1318, 1284, 1132, 1070, 816, 764, 698, 597 cm^{-1}.

HRMS (EI) [$C_{22}H_{23}N$] $[M]^+$ calculated: 301.1830, found: 301.1844.

1-([1,1'-Biphenyl]-4-yl)indoline (4an)

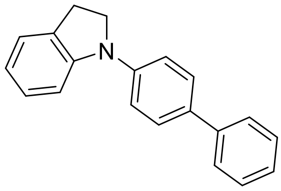

Compound **4an** was prepared according to the **General procedure E** from **1a** (34.4 mg, 0.2 mmol) and **3n** (67.0 µL, 0.6 mmol), and purified by silica gel column chromatography (*n*-hexane/DCM: 10/1) as a white solid (25.7 mg, 47% yield).

^{1}H NMR (300 MHz, $CDCl_3$) δ 7.65 – 7.56 (m, 4H), 7.45 (t, J = 7.8 Hz, 2H), 7.36 – 7.29 (m, 3H), 7.27 – 7.18 (m, 2H), 7.16 – 7.08 (m, 1H), 6.80 (t, J = 7.3 Hz, 1H), 4.01 (t, J = 8.5 Hz, 2H), 3.17 (t, J = 8.4 Hz, 2H).

MS(EI): *m/z* 271 $[M]^+$. The chemical shifts were consistent with those reported in the literature.[32]

1-([1,1'-Biphenyl]-4-yl)-2-methylindoline (4ao)

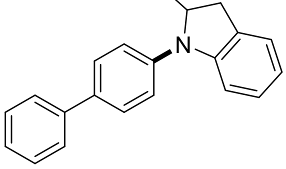

Compound **4ao** was prepared according to the **General procedure E** from **1a** (34.4 mg, 0.2 mmol) and **3o** (78.0 µL, 0.6 mmol), and purified by silica gel column chromatography (*n*-hexane/DCM: 10/1) as a colorless oil (26.0 mg, 46% yield).

^{1}H NMR (500 MHz, $CDCl_3$) δ 7.57 (dd, J = 8.3, 2.6 Hz, 4H), 7.40 (t, J = 7.6 Hz, 2H), 7.29 (d, J =

8.7 Hz, 3H), 7.12 (d, J = 7.6 Hz, 1H), 7.03 (t, J = 7.7 Hz, 1H), 6.90 (d, J = 7.8 Hz, 1H), 6.80 – 6.66 (m, 1H), 4.52 – 4.28 (m, 1H), 3.32 (dd, J = 15.4, 8.8 Hz, 1H), 2.73 (dd, J = 15.4, 7.0 Hz, 1H), 1.33 (d, J = 6.2 Hz, 3H).

^{13}C NMR (126 MHz, $CDCl_3$) δ 148.2, 142.8, 140.9, 135.2, 129.9, 128.9, 128.0, 127.3, 126.9, 126.8, 125.1, 121.2, 119.0, 108.7, 59.8, 37.2, 20.3.

IR (KBr): 3254, 3033, 2970, 2897, 1598, 1522, 1487, 1461, 1380, 1280, 1026, 975, 840, 763, 697 cm^{-1}.

HRMS (EI) [$C_{21}H_{19}N$] $[M]^+$ calculated: 285.1517, found: 285.1511.

1-([1,1'-Biphenyl]-4-yl)-1,2,3,4-tetrahydroquinoline (4ap)

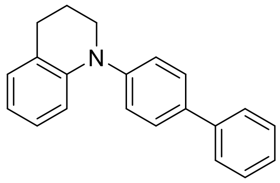

Compound **4ap** was prepared according to the **General procedure E** from **1a** (34.4 mg, 0.2 mmol) and **3p** (75.0 µL, 0.6 mmol), and purified by silica gel column chromatography (*n*-hexane/DCM: 10/1) as a white solid (33.9 mg, 59% yield).

^{1}H NMR (300 MHz, $CDCl_3$) δ 7.60 (t, J = 8.5 Hz, 4H), 7.46 (t, J = 7.5 Hz, 2H), 7.38 – 7.29 (m, 3H), 7.09 (d, J = 7.4 Hz, 1H), 6.99 (t, J = 7.6 Hz, 1H), 6.91 (d, J = 7.5 Hz, 1H), 6.76 (t, J = 7.7 Hz, 1H), 3.75 – 3.64 (t, J = 6.5 Hz, 2H), 2.88 (t, J = 6.5 Hz, 2H), 2.08 (p, J = 6.3 Hz, 2H).

MS(EI): *m/z* 285 $[M]^+$. The chemical shifts were consistent with those reported in the literature.[33]

4-([1,1'-Biphenyl]-4-yl)-3,4-dihydro-2*H*-benzo[*b*][1,4]oxazine (4aq)

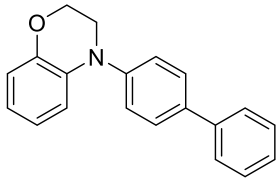

Compound **4aq** was prepared according to the **General procedure E** from **1a** (34.4 mg, 0.2 mmol) and **3q** (70.0 µL, 0.6 mmol), and purified by silica gel column chromatography (*n*-hexane/EtOAc: 10/1) as a white solid (20.0 mg, 35% yield), m.p. = 126.2 – 127.0 ^oC.

^{1}H NMR (500 MHz, $CDCl_3$) δ 7.61 (dd, J = 7.8, 6.0 Hz, 4H), 7.46 (t, J = 7.7 Hz, 2H), 7.39 – 7.32 (m, 1H), 7.32 (d, J = 8.6 Hz, 2H), 7.09 – 7.03 (m, 1H), 6.97 – 6.91 (m, 1H), 6.85 – 6.76 (m, 2H),

4.36 – 4.31 (m, 2H), 3.79 – 3.76 (m, 2H).

^{13}C NMR (126 MHz, $CDCl_3$) δ 146.4, 145.2, 140.7, 136.2, 132.0, 128.9, 128.1, 127.1, 126.9, 123.3, 121.0, 120.7, 117.4, 117.3, 64.5, 48.6.

IR (KBr): 3077, 3056, 3027, 2983, 2898, 1596, 1520, 1502, 1370, 1336, 1259, 1056, 750, 695 cm^{-1}.

HRMS (EI) [$C_{20}H_{17}NO$] $[M]^+$ calculated: 287.1310, found: 287.1321.

4-([1,1'-Biphenyl]-4-yl)morpholine (4ar)

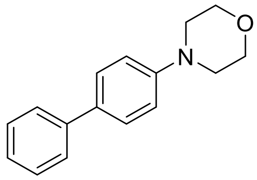

Compound **4ar** was prepared according to the **General procedure E** from **1a** (34.4 mg, 0.2 mmol) and **3r** (52.0 μL, 0.6 mmol), and purified by silica gel column chromatography (*n*-hexane/DCM: 5/1) as a white solid (10.3 mg, 21% yield).

^{1}H NMR (300 MHz, $CDCl_3$) δ 7.61 – 7.49 (m, 4H), 7.41 (t, *J* = 7.5 Hz, 2H), 7.33 – 7.24 (m, 1H), 6.99 (d, *J* = 8.9 Hz, 2H), 3.94 – 3.84 (m, 4H), 3.26 – 3.16 (m, 4H).

MS(EI): *m/z* 239 $[M]^+$. The chemical shifts were consistent with those reported in the literature.[32]

1-([1,1'-Biphenyl]-4-yl)pyrrolidine (4as)

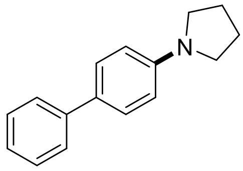

Compound **4as** was prepared according to the **General procedure E** from **1a** (34.4 mg, 0.2 mmol) and **3s** (50.0 μL, 0.6 mmol), and purified by silica gel column chromatography (*n*-hexane) as a white solid (5.0 mg, 11% yield).

^{1}H NMR (300 MHz, $CDCl_3$) δ 7.58 – 7.53 (m, 2H), 7.52 – 7.47 (m, 2H), 7.42 – 7.34 (m, 2H), 7.23 (t, *J* = 7.5 Hz, 1H), 6.64 (d, *J* = 8.7 Hz, 2H), 3.41 – 3.25 (m, 4H), 2.09 – 1.94 (m, 4H).

MS(EI): *m/z* 223 $[M]^+$. The chemical shifts were consistent with those reported in the literature.[34]

2-([1,1'-Biphenyl]-4-yl)-1,2,3,4-tetrahydroisoquinoline (4at)

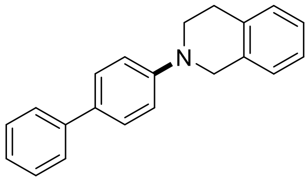

Compound **4at** was prepared according to the **General procedure E** from **1a** (34.4 mg, 0.2 mmol) and **3t** (76.0 μL, 0.6 mmol), and purified by silica gel column chromatography (*n*-hexane/DCM: 3/1) as a white solid (14.9 mg, 26% yield).

^{1}H NMR (300 MHz, $CDCl_3$) δ 7.59 (t, *J* = 7.8 Hz, 4H), 7.43 (t, *J* = 7.7 Hz, 2H), 7.30 (t, *J* = 7.3 Hz, 1H), 7.22 (s, 4H), 7.07 (d, *J* = 8.8 Hz, 2H), 4.49 (s, 2H), 3.64 (t, *J* = 5.9 Hz, 2H), 3.03 (t, *J* = 5.8 Hz, 2H).

MS(EI): *m/z* 285 $[M]^+$. The chemical shifts were consistent with those reported in the literature.[35]

***N*,*N*-Diethyl-[1,1'-biphenyl]-4-amine (4au)**

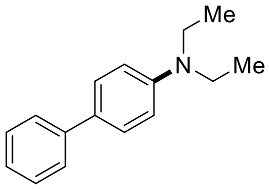

Compound **4au** was prepared according to the **General procedure E** from **1a** (34.4 mg, 0.2 mmol) and **3u** (62.0 μL, 0.6 mmol), and purified by silica gel column chromatography (*n*-hexane) as a white solid (8.3 mg, 18% yield).

^{1}H NMR (300 MHz, $CDCl_3$) δ 7.55 (d, *J* = 7.6 Hz, 2H), 7.48 (d, *J* = 8.6 Hz, 2H), 7.38 (t, *J* = 7.6 Hz, 2H), 7.23 (t, *J* = 7.4 Hz, 1H), 6.74 (d, *J* = 8.8 Hz, 2H), 3.39 (q, *J* = 7.1 Hz, 4H), 1.19 (t, *J* = 7.0 Hz, 6H).

MS(EI): *m/z* 225 $[M]^+$. The chemical shifts were consistent with those reported in the literature.[36]

***N*-(Biphenyl-4-ylmethyl)-*N*-methylaniline (5aa)**

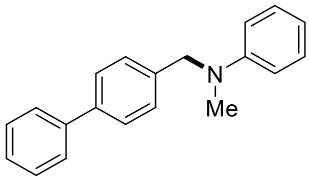

Compound **5aa** was prepared according to the **General procedure E** from **2a** (37.2 mg, 0.2 mmol) and **3a** (64.0 μL, 0.6 mmol), and purified by silica gel column chromatography (*n*-hexane/DCM:

10/1) as a white solid (44.3 mg, 81% yield).

^{1}H NMR (300 MHz, $CDCl_3$) δ 7.55 (t, *J* = 9.0 Hz, 4H), 7.41 (t, *J* = 7.5 Hz, 2H), 7.36 – 7.16 (m, 5H), 6.84 – 6.66 (m, 3H), 4.55 (s, 2H), 3.03 (s, 3H).

MS(EI): *m/z* 273 $[M]^+$. The chemical shifts were consistent with those reported in the literature.[37]

_N_-Methyl-_N_-(naphthalen-1-ylmethyl)aniline (5ba)

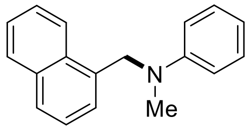

Compound **5ba** was prepared according to the **General procedure E** from **2b** (32.0 mg, 0.2 mmol) and **3a** (64.0 µL, 0.6 mmol), and purified by silica gel column chromatography (*n*-hexane/DCM: 10/1) as a colorless oil (43.1 mg, 87% yield).

^{1}H NMR (300 MHz, $CDCl_3$) δ 8.03 – 7.93 (m, 1H), 7.94 – 7.85 (m, 1H), 7.76 (d, *J* = 8.1 Hz, 1H), 7.60 – 7.46 (m, 2H), 7.44 – 7.33 (m, 1H), 7.31 (d, *J* = 6.9 Hz, 1H), 7.22 (t, *J* = 7.0 Hz, 2H), 6.83 – 6.66 (m, 3H), 4.97 (s, 2H), 3.08 (s, 3H).

MS(EI): *m/z* 247 $[M]^+$. The chemical shifts were consistent with those reported in the literature.[38]

_N_-Methyl-_N_-(4-(trifluoromethoxy)benzyl)aniline (5ca)

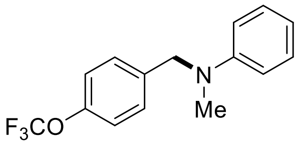

Compound **5ca** was prepared according to the **General procedure E** from **2c** (38.8 mg, 0.2 mmol) and **3a** (64.0 µL, 0.6 mmol), and purified by silica gel column chromatography (*n*-hexane/DCM: 10/1) as a colorless oil (43.0 mg, 76% yield).

^{1}H NMR (300 MHz, $CDCl_3$) δ 7.37 – 7.00 (m, 6H), 6.73 (d, *J* = 8.8 Hz, 3H), 4.52 (s, 2H), 3.01 (s, 3H).

^{13}C NMR (176 MHz, $CDCl_3$) δ 149.6, 148.2, 137.9, 129.4, 128.1, 121.3, 120.6 (q, *J* = 256.9 Hz), 117.0, 112.5, 56.2, 38.7.

^{19}F NMR (282 MHz, $CDCl_3$) δ -58.39 (s, 3F).

IR (KBr): 3062, 3035, 2898, 2821, 1600, 1505, 1347, 1270, 1215, 1161, 1118, 929, 749, 692 cm^{-1}.

HRMS (EI) $[C_{15}H_{14}F_3NO]$ $[M]^+$ calculated: 281.1027, found: 281.1022.

***N*-Methyl-*N*-(1-(naphthalen-2-yl)ethyl)aniline (5da)**

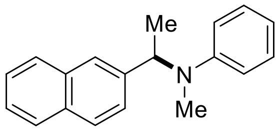

Compound **5da** was prepared according to the **General procedure E** from **2d** (34.8 mg, 0.2 mmol) and **3a** (64.0 µL, 0.6 mmol), and purified by silica gel column chromatography (*n*-hexane/DCM: 10/1) as a white solid (22.2 mg, 43% yield).

^{1}H NMR (300 MHz, $CDCl_3$) δ 7.89 – 7.68 (m, 4H), 7.52 – 7.40 (m, 3H), 7.33 – 7.19 (m, 2H), 6.90 (d, *J* = 8.1 Hz, 2H), 6.75 (t, *J* = 7.2 Hz, 1H), 5.28 (q, *J* = 6.8 Hz, 1H), 2.69 (s, 3H), 1.64 (d, *J* = 6.8 Hz, 3H).

MS(EI): *m/z* 261 $[M]^+$. The chemical shifts were consistent with those reported in the literature.[39]

***N*-Decyl-*N*-methylaniline (5ea)**

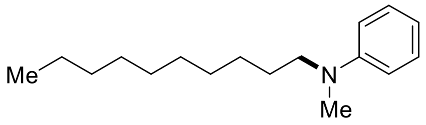

Compound **5ea** was prepared according to the **General procedure E** from **2e** (32.0 mg, 0.2 mmol) and **3a** (64.0 µL, 0.6 mmol), and purified by silica gel column chromatography (*n*-hexane/DCM: 10/1) as a colorless oil (9.7 mg, 20% yield).

^{1}H NMR (300 MHz, $CDCl_3$) δ 7.22 (t, *J* = 8.0 Hz, 2H), 6.67 (m, 3H), 3.29 (t, *J* = 7.6 Hz, 2H), 2.92 (s, 3H), 1.57 (s, 2H), 1.27 (m, 14H), 0.88 (t, *J* = 6.5 Hz, 3H).

MS(EI): *m/z* 247 $[M]^+$. The chemical shifts were consistent with those reported in the literature.[40]

***N*-Methyl-*N*-phenyl-4-((((*R*)-2,5,7,8-tetramethyl-2-((4*R*,8*R*)-4,8,12-trimethyltridecyl)chroman-6-yl)oxy)methyl)aniline (4ada)**

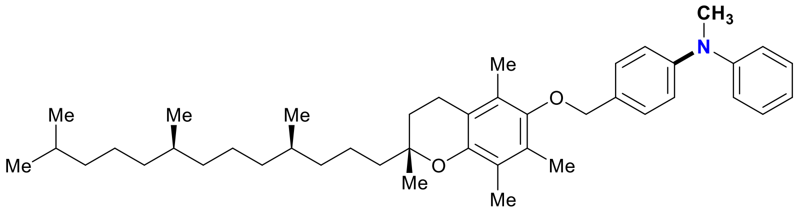

Compound **4ada** was prepared according to the **General procedure E** from **1ad** (107.8 mg, 0.2 mmol) and **3a** (64.0 µL, 0.6 mmol), and purified by silica gel column chromatography (*n*-hexane/DCM: 3/1) as a light-yellow oil (103.8 mg, 83% yield).

^{1}H NMR (700 MHz, $CDCl_3$) δ 7.48 – 7.39 (m, 2H), 7.36 – 7.27 (m, 2H), 7.12 – 7.04 (m, 4H), 7.00

(tt, J = 7.4, 1.1 Hz, 1H), 4.65 (s, 2H), 3.36 (s, 3H), 2.62 (t, J = 6.8 Hz, 2H), 2.27 (s, 3H), 2.22 (s, 3H), 2.14 (s, 3H), 1.92 – 1.75 (m, 2H), 1.65 – 1.52 (m, 3H), 1.51 – 1.37 (m, 4H), 1.35 – 1.23 (m, 12H), 1.21 – 1.05 (m, 7H), 0.94 – 0.84 (m, 12H).

^{13}C NMR (176 MHz, $CDCl_3$) δ 149.1, 148.9, 148.2, 148.0, 130.7, 129.3, 129.2, 128.1, 126.1, 123.0, 121.5, 120.7, 120.4, 117.7, 74.9, 74.8, 40.4, 40.2, 39.5, 37.6, 37.5, 37.4, 32.9, 32.8, 31.4, 28.1, 25.0, 24.6, 24.0, 22.9, 22.8, 21.2, 20.8, 19.9, 19.8, 13.1, 12.2, 12.0.

IR (KBr): 3031, 2951, 2925, 2866, 1596, 1514, 1496, 1462, 1412, 1368, 1343, 1256, 1132, 1085, 993, 697 cm^{-1}.

HRMS (**ESI**) [$C_{43}H_{63}NO_2Na$] $[M+Na]^+$ calculated: 648.4756, found: 648.4759.

(2*S*,5*R*)-2-*iso*-Propyl-5-methylcyclohexyl 4-([1,1'-biphenyl]-4-yl(methyl)amino)benzoate (4av)

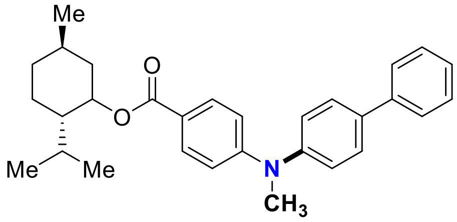

Compound **4av** was prepared according to the **General procedure E** from **1a** (34.4 mg, 0.2 mmol) and **3v** (87.0 mg, 0.6 mmol), and purified by silica gel column chromatography (*n*-hexane/EtOAc: 15/1) as a light-yellow oil (66.8 mg, 75% yield).

^{1}H NMR (700 MHz, $CDCl_3$) δ 7.97 – 7.91 (m, 2H), 7.64 – 7.60 (m, 4H), 7.49 – 7.45 (m, 2H), 7.37 (ddt, J = 8.6, 7.0, 1.2 Hz, 1H), 7.30 – 7.27 (m, 2H), 6.94 – 6.79 (m, 2H), 4.92 (td, J = 10.9, 4.4 Hz, 1H), 3.41 (s, 3H), 2.17 – 2.12 (m, 1H), 2.02 – 1.97 (m, 1H), 1.76 – 1.71 (m, 2H), 1.61 – 1.52 (m, 2H), 1.18 – 1.06 (m, 2H), 0.93 (dd, J = 9.2, 6.8 Hz, 7H), 0.82 (d, J = 7.0 Hz, 3H).

^{13}C NMR (176 MHz, $CDCl_3$) δ 166.3, 152.4, 146.9, 140.5, 137.7, 131.1, 128.9, 128.4, 127.3, 127.0, 125.5, 120.5, 114.7, 74.2, 47.4, 41.2, 40.3, 34.5, 31.6, 26.6, 23.8, 22.2, 20.9, 16.7.

IR (KBr): 3032, 2954, 2869, 1704, 1599, 1513, 1487, 1347, 1275, 1181, 1115, 768, 697 cm^{-1}.

HRMS (ESI) [$C_{30}H_{35}NO_2Na$] $[M+Na]^+$ calculated: 464.2565, found: 464.2568.

(2*S*,5*R*)-2-*iso*-Propyl-5-methylcyclohexyl 4-(((8*R*,9*S*,13*S*,14*S*)-17-methoxy-13-methyl-7,8,9,11,12,13,14,15,16,17-decahydro-6*H*-cyclopenta[*a*]phenanthren-3-yl)(methyl)amino)benzoate (4aev)

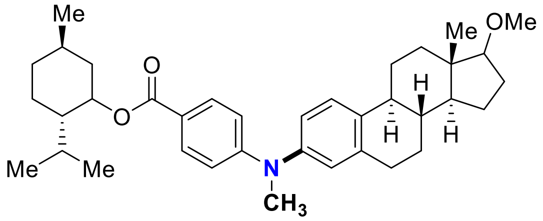

Compound **4aev** was prepared according to the **General procedure E** from **1ae** (57.6 mg, 0.2 mmol) and **3v** (87.0 mg, 0.6 mmol), and purified by silica gel column chromatography (*n*-hexane/EtOAc: 10/1) as a light-yellow oil (69.1 mg, 62% yield).

^{1}H NMR (700 MHz, $CDCl_3$) δ 7.90 – 7.81 (m, 2H), 7.29 (dd, *J* = 8.4, 1.1 Hz, 1H), 6.96 (dd, *J* = 8.3, 2.5 Hz, 1H), 6.91 (dd, *J* = 2.4, 1.1 Hz, 1H), 6.78 – 6.70 (m, 2H), 4.86 (td, *J* = 10.8, 4.4 Hz, 1H), 3.38 (s, 3H), 3.32 (s, 3H), 3.32 (t, *J* = 8.4 Hz, 1H), 2.91 – 2.76 (m, 2H), 2.36 – 2.28 (m, 1H), 2.27 – 2.12 (m, 1H), 2.15 – 2.02 (m, 3H), 1.99 – 1.92 (m, 1H), 1.92 – 1.86 (m, 1H), 1.74 – 1.66 (m, 3H), 1.58 – 1.44 (m, 4H), 1.49 – 1.38 (m, 1H), 1.36 (tt, *J* = 12.7, 6.5 Hz, 2H), 1.28 – 1.16 (m, 1H), 1.17 – 1.06 (m, 1H), 1.11 – 1.00 (m, 1H), 0.92 – 0.86 (m, 8H), 0.81 (s, 3H), 0.77 (d, *J* = 6.9 Hz, 3H).

^{13}C NMR (176 MHz, $CDCl_3$) δ 166.4, 152.7, 145.1, 138.5, 137.9, 131.0, 126.8, 126.3, 123.3, 119.4, 113.5, 90.9, 74.0, 58.0, 50.5, 47.5, 44.4, 43.3, 41.2, 40.3, 38.5, 38.1, 34.5, 31.6, 29.7, 27.9, 27.2, 26.6, 26.4, 23.8, 23.2, 22.2, 20.9, 16.7, 11.7.

IR (KBr): 2953, 2924, 2870, 1704, 1600, 1514, 1498, 1355, 1274, 1180, 1119, 918, 768, 732 cm^{-1}.

HRMS (ESI) [$C_{37}H_{52}NO_3$] $[M+H]^+$ calculated: 558.3947, found: 558.3929.

(8*R*,9*S*,13*S*,14*S*)-17-Methoxy-13-methyl-*N*-(methyl-*d*^{3})-*N*-phenyl-7,8,9,11,12,13,14,15,16,17-decahydro-6*H*-cyclopenta[*a*]phenanthren-3-amine (*d*^{3}-4aea)

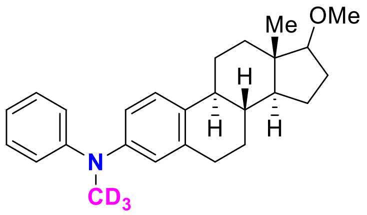

Compound ***d*^{3}-4aea** was prepared according to the **General procedure E** from **1ae** (57.6 mg, 0.2 mmol) and ***d*^{3}-3a** (70.0 µL, 0.6 mmol), and purified by silica gel column chromatography (*n*-hexane/DCM: 4/1) as a white solid (58.3 mg, 77% yield), m.p. = 110.3 – 111.2 °C.

^{1}H NMR (700 MHz, $CDCl_3$) δ 7.29 – 7.20 (m, 2H), 7.26 – 7.18 (m, 1H), 7.01 – 6.93 (m, 2H), 6.94 – 6.83 (m, 2H), 6.81 (dd, *J* = 2.4, 1.2 Hz, 1H), 3.39 (s, 3H), 3.33 (t, *J* = 8.4 Hz, 1H), 2.88 – 2.77 (m, 2H), 2.30 (dq, *J* = 11.2, 4.0, 3.5 Hz, 1H), 2.22 (td, *J* = 11.3, 4.1 Hz, 1H), 2.11 – 2.04 (m, 2H), 1.91 – 1.85 (m, 1H), 1.49 – 1.30 (m, 1H), 1.58 – 1.49 (m, 2H), 1.49 – 1.30 (m, 4H), 1.26 – 1.19 (m, 1H), 0.81 (s, 3H).

^{13}C NMR (176 MHz, $CDCl_3$) δ 149.3, 146.6, 137.8, 134.4, 129.1, 126.3, 122.1, 120.1, 119.5, 118.9, 90.9, 58.0, 50.4, 44.2, 43.4, 38.7, 38.2, 29.8, 27.9, 27.4, 26.4, 23.2, 11.7.

IR (KBr): 3058, 3031, 2931, 2867, 2193, 2060, 1610, 1592, 1494, 1429, 1337, 1298, 1264, 1193, 1133, 1103, 992, 812, 778, 702 cm^{-1}.

HRMS (ESI) [$C_{26}H_{31}D_3NO$] [M+H]$^+$ calculated: 379.2829, found: 379.2820.

4-((((1*R*,2*S*,5*R*)-2-Isopropyl-5-methylcyclohexyl)oxy)methyl)-*N*-(methyl-d^3)-*N*-phenylaniline (d^3-4afa)

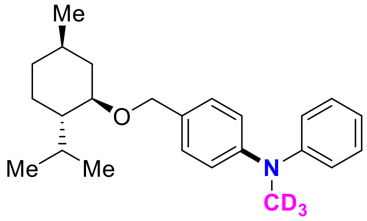

Compound ***d^3*-4afa** was prepared according to the **General procedure E** from **1af** (53.0 mg, 0.2 mmol) and ***d^3*-3a** (70.0 µL, 0.6 mmol), and purified by silica gel column chromatography (*n*-hexane/EtOAc: 20/1) as a light-yellow oil (62.5 mg, 88% yield).

^{1}H NMR (700 MHz, $CDCl_3$) δ 7.36 – 7.22 (m, 4H), 7.08 – 6.98 (m, 4H), 6.98 – 6.90 (m, 1H), 4.62 (d, J = 11.0 Hz, 1H), 4.36 (d, J = 11.0 Hz, 1H), 3.20 (td, J = 10.6, 4.2 Hz, 1H), 2.40 – 2.27 (m, 1H), 2.23 (dtd, J = 12.2, 3.6, 2.0 Hz, 1H), 1.67 (ddq, J = 23.0, 13.0, 3.2 Hz, 2H), 1.46 – 1.34 (m, 1H), 1.35 – 1.25 (m, 1H), 1.02 – 0.87 (m, 9H), 0.75 (d, J = 7.0 Hz, 3H).

^{13}C NMR (176 MHz, $CDCl_3$) δ 149.2, 148.5, 132.3, 129.3, 129.2, 121.0, 120.9, 120.0, 78.6, 70.3, 48.4, 40.4, 34.7, 31.7, 25.6, 23.3, 22.5, 21.2, 16.2.

IR (KBr): 3030, 2953, 2920, 2868, 2198, 2064, 1595, 1513, 1495, 1513, 1494, 1334, 1272, 1068, 698 cm^{-1}.

HRMS (ESI) [$C_{24}H_{30}D_3NONa$] $[M+Na]^+$ calculated: 377.2468, found: 377.2460.

4-((Methyl-*d*^{3})(phenyl)amino)phenyl (*R*)-2-(6-methoxynaphthalen-2-yl)propanoate (*d*^{3}-4aga)

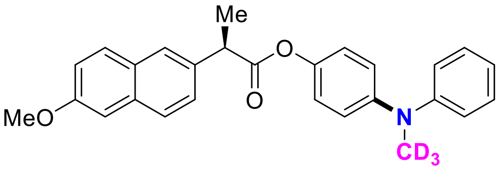

Compound ***d*^{3}-4aga** was prepared according to the **General procedure E** from **1ag** (65.0 mg, 0.2 mmol) and ***d*^{3}-3a** (70.0 µL, 0.6 mmol), and purified by silica gel column chromatography (*n*-hexane/EtOAc: 10/1) as a light-yellow oil (58.3 mg, 70% yield).

^{1}H NMR (700 MHz, $CDCl_3$) δ 7.78 (dt, *J* = 2.0, 0.6 Hz, 1H), 7.77 – 7.73 (m, 2H), 7.52 (dd, *J* = 8.4, 1.9 Hz, 1H), 7.26 – 7.23 (m, 2H), 7.17 (dd, *J* = 8.8, 2.5 Hz, 1H), 7.15 (d, *J* = 2.5 Hz, 1H), 6.99 – 6.95 (m, 4H), 6.94 – 6.89 (m, 3H), 4.10 (q, *J* = 7.1 Hz, 1H), 3.93 (s, 3H), 1.70 (, *J* = 7.2 Hz, 3H).

^{13}C NMR (176 MHz, $CDCl_3$) δ 173.6, 157.8, 149.0, 146.8, 145.3, 135.4, 133.9, 129.4, 129.3, 129.1, 127.5, 126.3, 126.2, 122.1, 121.8, 121.1, 119.8, 119.2, 105.7, 55.4, 50.9, 45.7, 18.7.

IR (KBr): 3056, 2975, 2935, 2840, 2188, 2057, 1754, 1605, 1496, 1327, 1268, 1200, 1135, 852 cm^{-1}.

HRMS (ESI) [$C_{27}H_{22}D_3NO_3Na$] [M+Na]$^+$ calculated: 437.1920, found: 437.1929.

_N_-(Methyl-_d_³)-_N_-phenyl-4-((((_R_)-2,5,7,8-tetramethyl-2-((4_R_,8_R_)-4,8,12-trimethyltridecyl)chroman-6-yl)oxy)methyl)aniline (_d_³-4ada)

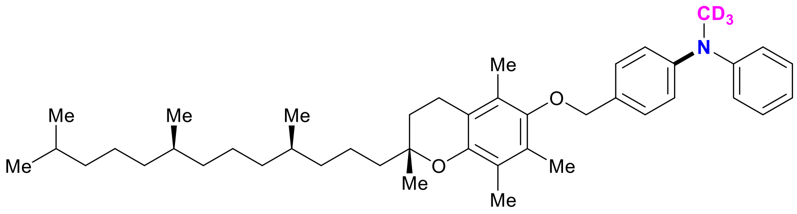

Compound ***d^3*-4ada** was prepared according to the **General procedure E** from **1ad** (110.0 mg, 0.2 mmol) and ***d^3*-3a** (70.0 µL, 0.6 mmol), and purified by silica gel column chromatography (*n*-hexane/DCM: 3/1) as a light-yellow oil (99.0 mg, 79% yield).

^{1}H NMR (700 MHz, $CDCl_3$) δ 7.51 – 7.39 (m, 2H), 7.36 – 7.27 (m, 2H), 7.12 – 7.06 (m, 4H), 7.01 (tt, *J* = 7.3, 1.2 Hz, 1H), 4.67 (s, 2H), 2.64 (t, *J* = 6.9 Hz, 2H), 2.28 (s, 3H), 2.24 (s, 3H), 2.16 (s, 3H), 1.94 – 1.76 (m, 2H), 1.66 – 1.54 (m, 3H), 1.53 – 1.39 (m, 3H), 1.37 – 1.25 (m, 11H), 1.22 – 1.08 (m, 7H), 0.96 – 0.86 (m, 12H).

^{13}C NMR (176 MHz, $CDCl_3$) δ 149.0, 148.8, 148.2, 148.0, 130.7, 129.3, 129.3, 128.1, 126.1, 123.0, 121.5, 120.7, 120.3, 117.7, 74.9, 74.8, 40.2, 39.5, 37.5, 37.4, 32.9, 32.8, 31.4, 28.1, 25.0, 24.6, 24.0, 22.9, 22.8, 21.2, 20.8, 19.9, 19.8, 19.7, 13.1, 12.2, 12.0.

IR (KBr): 3032, , 2953, 2905, 2865, 2181, 2059, 1595, 1514, 1495, 1462, 1414, 1367, 1335, 1258, 1084, 993, 748, 699 cm^{-1}.

HRMS (ESI) [$C_{43}H_{60}D_3NO_2Na$] [M+Na]$^+$ calculated: 651.4945, found: 651.4938.

1,2-Dimethyl-1,2-diphenylhydrazine (7)

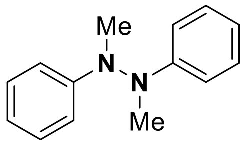

Compound **7** was prepared according to the **General procedure E** from **3a** (64.0 µL, 0.6 mmol), and purified by silica gel column chromatography (*n*-hexane/DCM: 10/1) as a light-yellow oil (17.0 mg, 27% yield).

^{1}H NMR (300 MHz, $CDCl_3$) δ 7.41 – 7.17 (m, 4H), 6.96 – 6.68 (m, 6H), 3.01 (s, 6H).

MS(EI): *m/z* 212 [M]$^+$. The chemical shifts were consistent with those reported in the literature.[41]

3.7 General procedure for the scale-up reaction

In a nitrogen-filled glovebox, to a flame-dried flask was added 4-fluorobiphenyl **1a** (0.86 g, 5.0 mmol), KO*t*Bu (2.25 g, 20.0 mmol), dry triglyme (20 mL), and *N*-methylaniline **3a** (1.62 mL, 15.0 mmol), sequentially. The flask was then sealed and moved out from the glovebox. After stirring for 5 min, a triethylsilyl boronate (2.42 g, 10.0 mmol) solution in 5.0 mL dry triglyme was injected to the flask slowly over 30 minutes. The solution was stirred at room temperature for 24 h. The reaction mixture was diluted with Et_2O (100.0 mL), then extracted with Et_2O, washed with water and brine, dried over Na_2SO_4, then concentrated under vacuum to give the crude, which was purified by column chromatography on silica gel to give the corresponding amines **4aa** (1.13 g, 87% yield).

4. Supplementary Discussion

4.1 The NMR spectroscopic studies

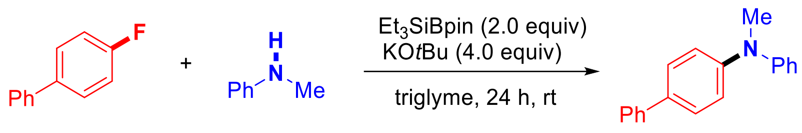

Following the **General procedure E**, charging **1a** (17.2 mg, 0.1 mmol), **3a** (32.0 μL, 0.3 mmol), silyl boronate $Et_3SiBpin$ (48.4 mg, 0.2 mmol), KO*t*Bu (44.8 mg, 0.4 mmol), and then anhydrous triglyme (0.5 mL) sequentially into a flame-dried screw-capped test tube. And then stirred in glovebox at room temperature for 24 h. The reaction mixture was then subjected to ^{11}B NMR analysis using THF-d^8 as a solvent to show the details of the reaction. After that the reaction mixture was quenched by adding D_2O (2.0 mL) while stirring for 5 min, then the ^{19}F NMR analysis of the water system was conducted to show the details of the reaction. The organic system was extracted with Et_2O (5 mL), washed with water, dried over Na_2SO_4, and concentrated under vacuum, followed by 3-fluoropyridine (8.6 μL, 0.1 mmol) as an internal standard. Then the ^{1}H NMR analysis and ^{19}F NMR analysis of the crude mixture were conducted to show the details of the model reaction.

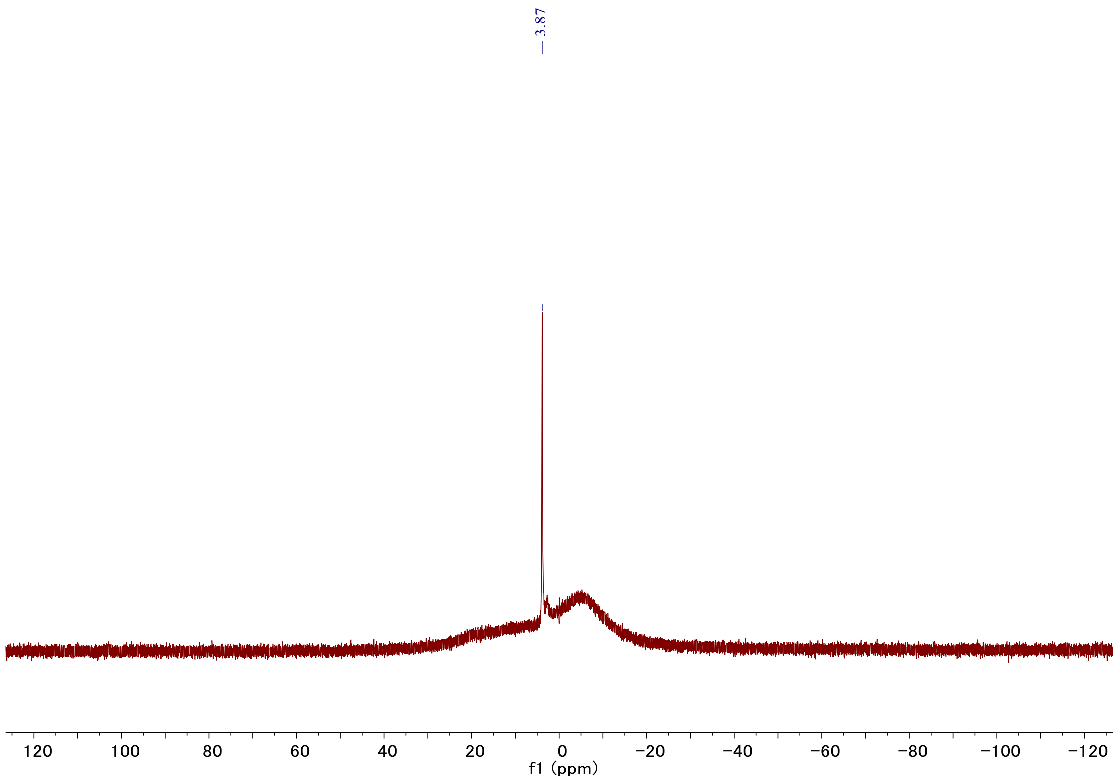

Supplementary Figure 1.^{11}B NMR (225 MHz, THF-d_8, 25 °C) observation of the model reaction.

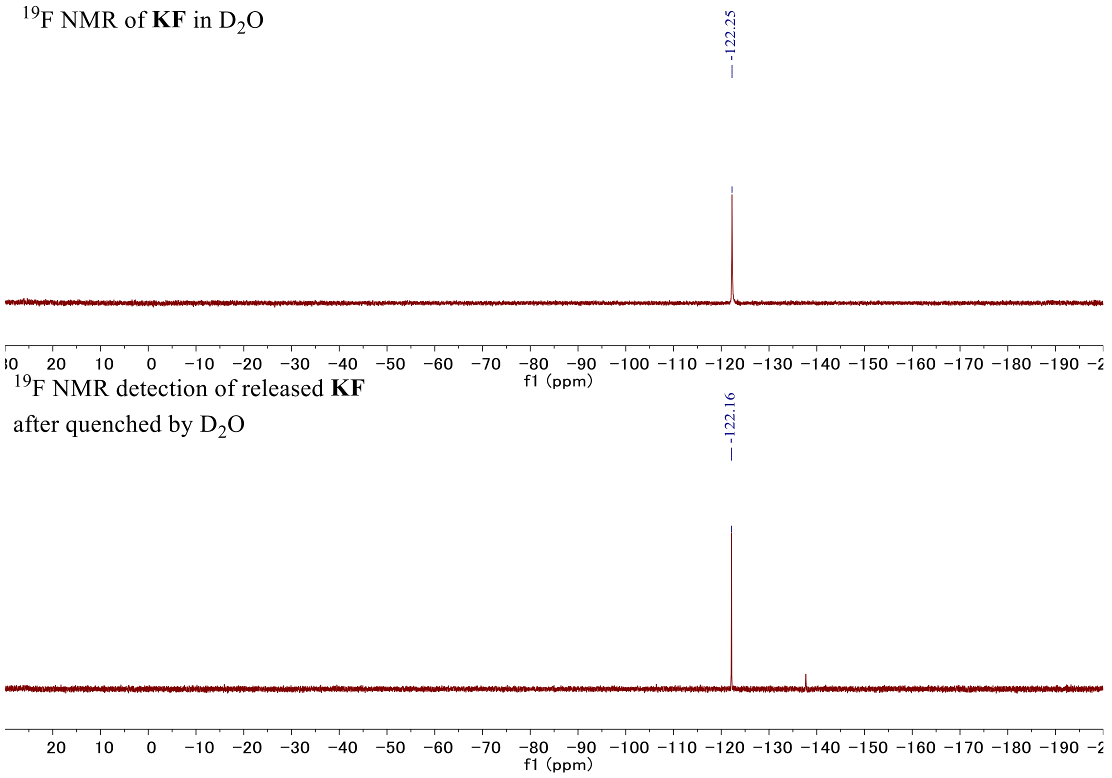

Supplementary Figure 2. ^{19}F NMR (282 MHz, D_2O, 25 °C) observation of KF in D_2O and KF released in the model reaction.

4.2 Reaction with radical scavenger TEMPO

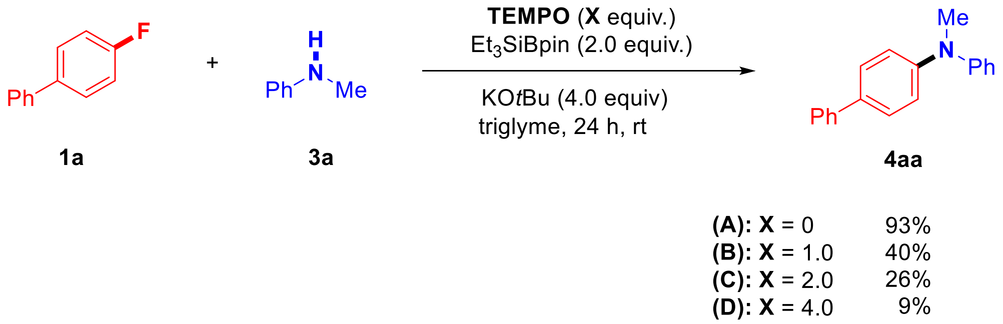

Supplementary Figure 3. Effect of TEMPO to the silylboronate-mediated coupling reaction of aryl fluoride **1a** with **3a**.

Following the **General procedure E**, charging **1a** (17.2 mg, 0.1 mmol), **3a** (32.0 µL, 0.3 mmol), silyl boronate $Et_3SiBpin$ (48.4 mg, 0.2 mmol), KO*t*Bu (44.8 mg, 0.4 mmol), TEMPO, and then anhydrous triglyme (0.5 mL) sequentially into a flame-dried screw-capped test tube. Then move out from glovebox and stirred at room temperature for 24 h. The reaction tube was diluted with Et_2O (5 mL), then extracted with Et_2O, washed with brine, dried over Na_2SO_4, then concentrated under vacuum, followed by adding 3-fluoropyridine (8.6 µL, 0.1 mmol) as an internal standard, then ^{1}H NMR analysis was taken to show the reaction details.

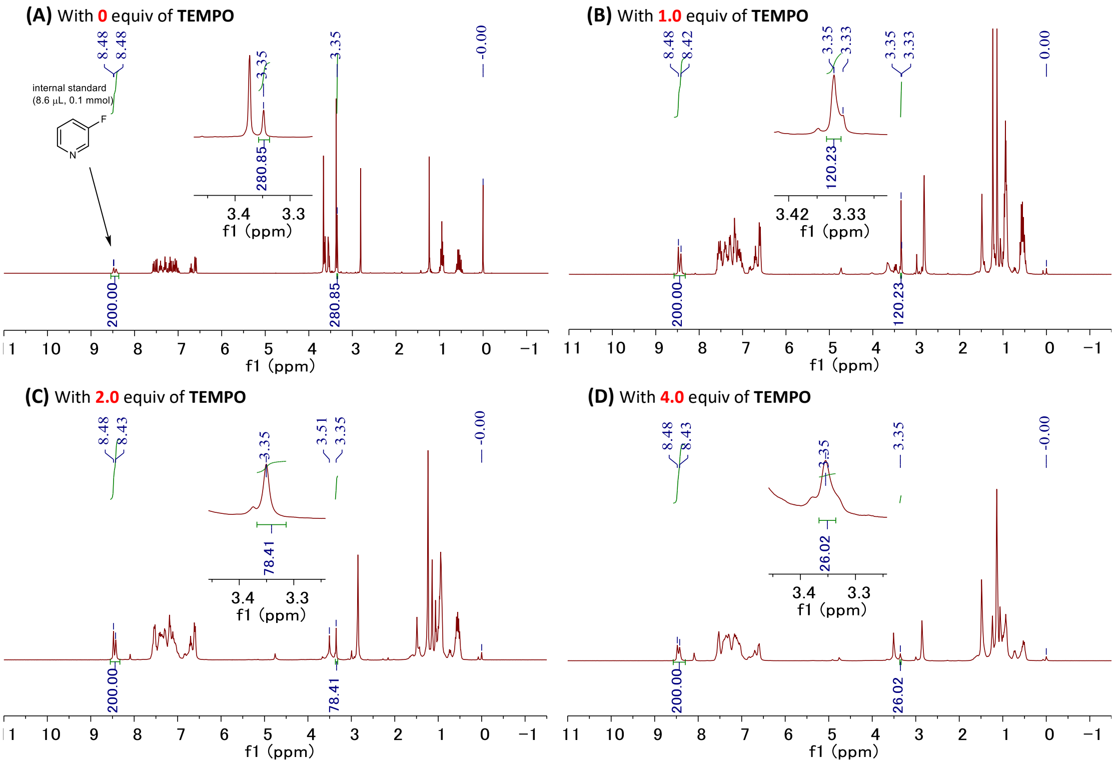

Supplementary Figure 4. ^{1}H NMR (300 MHz, $CDCl_3$, 25 °C) observation of the effect of TEMPO to the coupling reaction.

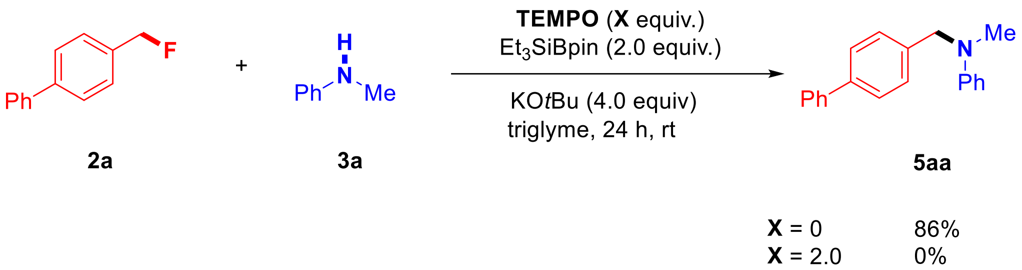

Supplementary Figure 5. Effect of TEMPO to the silylboronate-mediated coupling reaction of benzyl fluoride **2a** with **3a**.

Following the **General procedure E**, charging **2a** (18.6 mg, 0.1 mmol), **3a** (32.0 μL, 0.3 mmol), silyl boronate $Et_3SiBpin$ (48.4 mg, 0.2 mmol), KO*t*Bu (44.8 mg, 0.4 mmol), TEMPO (32.0 mg, 0.2 mmol), and then anhydrous triglyme (0.5 mL) sequentially into a flame-dried screw-capped test tube. Then move out from glovebox and stirred at room temperature for 24 h. The reaction tube was diluted with Et_2O (5 mL), then extracted with Et_2O, washed with brine, dried over Na_2SO_4, then concentrated under vacuum, followed by adding 3-fluoropyridine (8.6 μL, 0.1 mmol) as an internal standard, then ^1H NMR analysis was taken to show the reaction details.

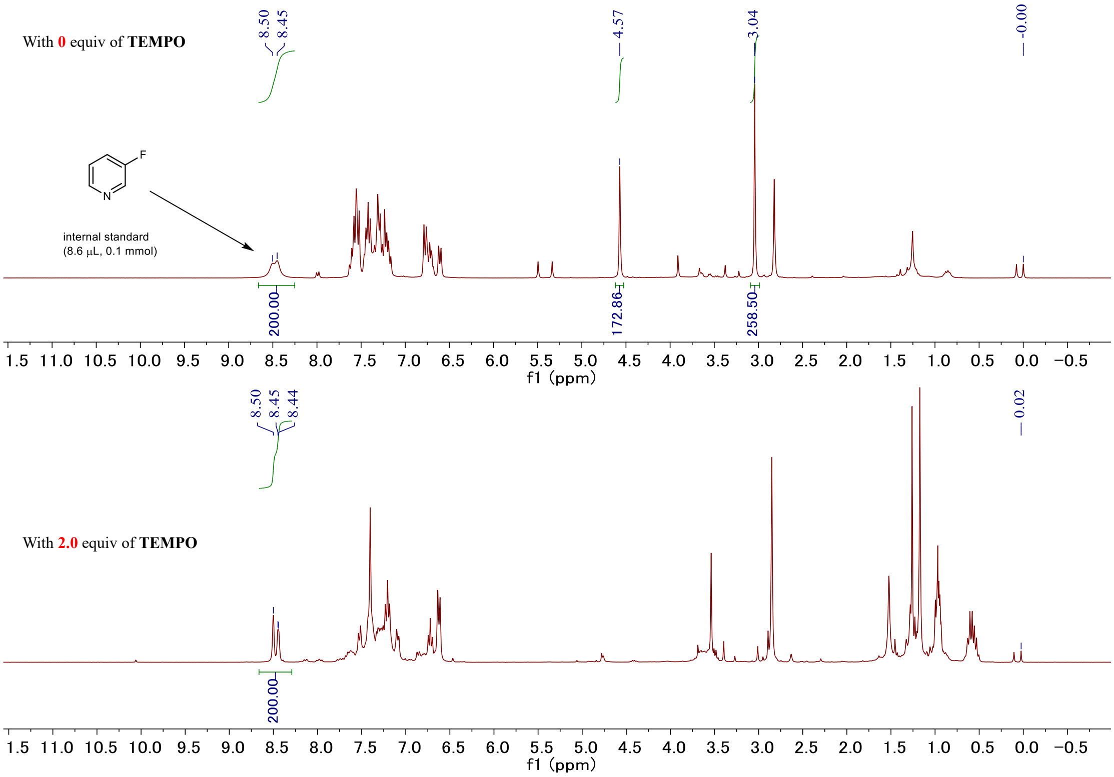

Supplementary Figure 6. ^1H NMR (300 MHz, $CDCl_3$, 25 °C) observation of the effect of TEMPO to the coupling reaction of benzyl fluoride **2a** with **3a**.

4.3 Chemoselectivites of organic halides

Evaluate aryl halides under the standard conditions:

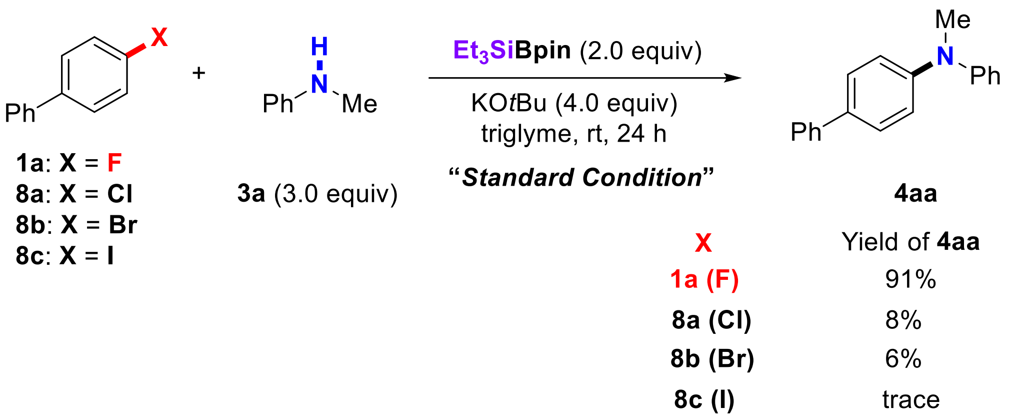

X	Yield of **4aa**
**1a (F)**	91%
**8a (Cl)**	8%
**8b (Br)**	6%
**8c (I)**	trace

Supplementary Figure 7. Effect of aryl halides to the silylboronate-mediated coupling reaction.

Following the **General procedure E**, charging **8** (0.1 mmol), **3a** (32.0 μL, 0.3 mmol), silyl boronate $Et_3SiBpin$ (48.4 mg, 0.2 mmol), KO*t*Bu (44.8 mg, 0.4 mmol), and then anhydrous triglyme (0.5 mL) sequentially into a flame-dried screw-capped test tube. Then move out from glovebox and stirred at room temperature for 24 h. The reaction tube was diluted with Et_2O (5 mL), then extracted with Et_2O, washed with brine, dried over Na_2SO_4, then concentrated under vacuum, followed by adding 3-fluoropyridine (8.6 μL, 0.1 mmol) as an internal standard, then ^1H NMR analysis was taken to show the reaction details.

Note: for the reaction using **8c**, after the reaction finished and work-up, the trace yield of **4aa** was observed by thin layer chromatography (TLC, eluent: *n*-hexane/DCM: 10/1).

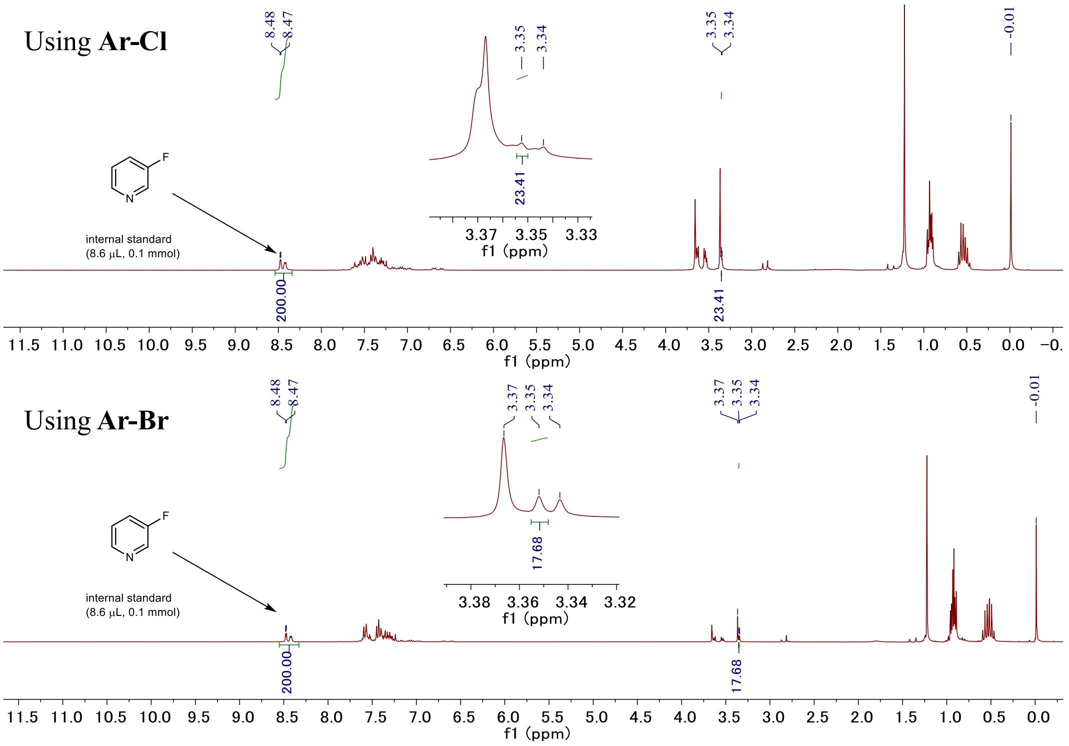

Supplementary Figure 8. ^{1}H NMR (300 MHz, $CDCl_3$, 25 °C) observation of the effect of aryl halides to the silylboronate-mediated coupling reaction.

Evaluate alkyl halides under the standard conditions with/without silylboronate:

Firstly, we evaluated the reaction of alkyl bromide with *N*-Me-aniline **3a** under standard conditions, and desired amination product was obtained in 91% yield. However, even without silylboronate, the amination product formed quantitatively. Alkyl chloride also gave the desired amination product in high yield even without silylboronate. On the other hand, alkyl fluoride **2e** gave the product **5ea** in a 26% NMR yield under standard conditions but no reaction without silylboronate. These results agree with the report.[42]

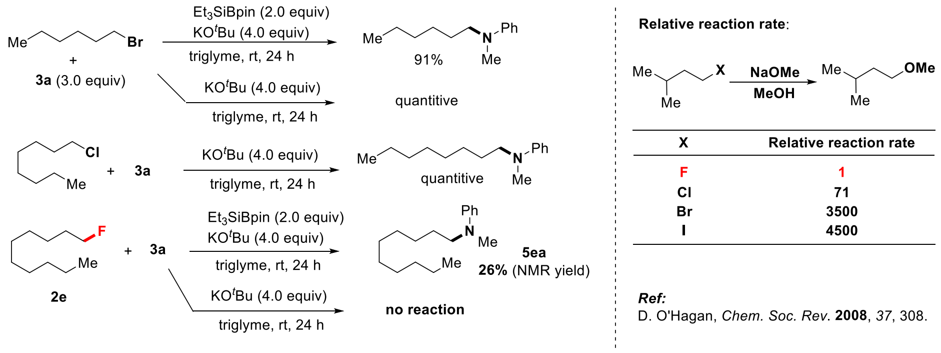

Supplementary Figure 9. Evaluate of alkyl halides for this reaction.

Evaluate allyl fluoride or cinnamyl fluoride under the standard conditions with/without silylboronate:

The reaction of allyl fluoride or cinnamyl fluoride under the standard conditions gave the complex mixtures, and we detected no desired products. Furthermore, allyl fluoride or cinnamyl fluoride also give the same result even under identical conditions but without silylboronate. This is presumably because of allyl fluoride and cinnamyl fluoride are too reactive and unstable.

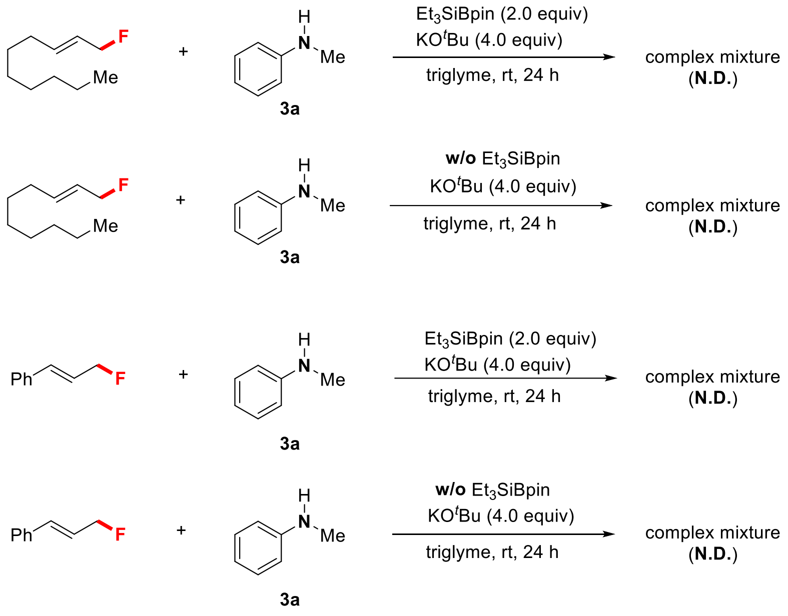

Supplementary Figure 10. Evaluate of allyl fluoride or cinnamyl fluoride for this reaction.

4.4 Mechanism control experiments

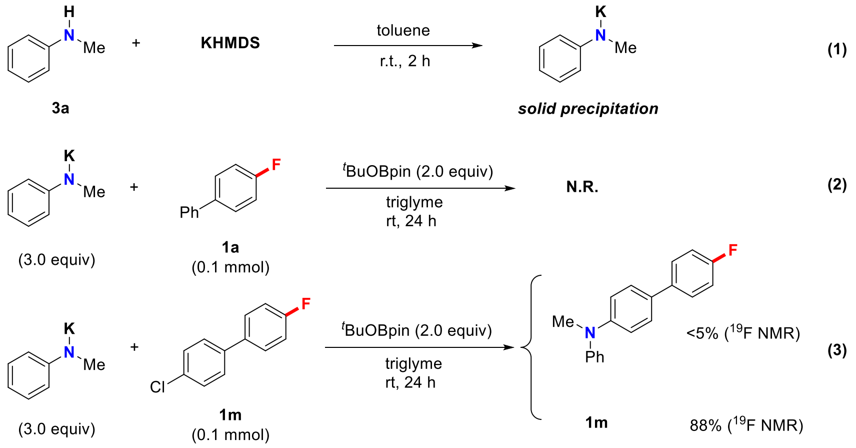

Supplementary Figure 11. Mechanism control experiments.

Reaction (**1**): Synthetic procedure was followed to a previously reported method.[43] In a nitrogen-filled glovebox, to a flask were charged potassium hexamethyldisilazide (KHMDS, 600 mg, 3.0 mmol) and dry toluene (6 mL). Stirred at room temperature until solid was fully dissolved. To this solution was added *N*-methylaniline **3a** (330 μL, 3.05 mmol) dropwise, and keep stirring while a precipitate of potassium salt (KNPhMe) was formed slowly. After stirring for 2 h, the product was filtered and washed twice with dry toluene (3 mL) and dry *n*-hexane (3 mL). The product was collected and move out of glovebox, and then dried in vacuo to yield solvent-free K-salt of **3a** as a solid (411 mg, 95% yield).

Reaction (**2**) and (**3**): In a nitrogen-filled glovebox, to a flame-dried screw-capped test tube was added sequentially aryl fluorides **1a** (17.2 mg, 0.1 mmol) or **1m** (20.6 mg, 0.1 mmol), KNPhMe (43.5 mg, 0.3 mmol), dry triglyme (0.5 mL), and ^{t}BuOBpin (40.0 mg, 0.2 mmol, freshly prepared). The tube was then sealed and moved out of glovebox and stirred at room temperature for 24 h. The reaction tube was diluted with Et_2O (5 mL), then extracted with Et_2O, washed with brine, dried over Na_2SO_4, then concentrated under vacuum, followed by adding 3-fluoropyridine (8.6 μL, 0.1 mmol) as an internal standard.

^{1}H NMR and **^{19}F NMR** analysis of reaction (**3**) are copied below:

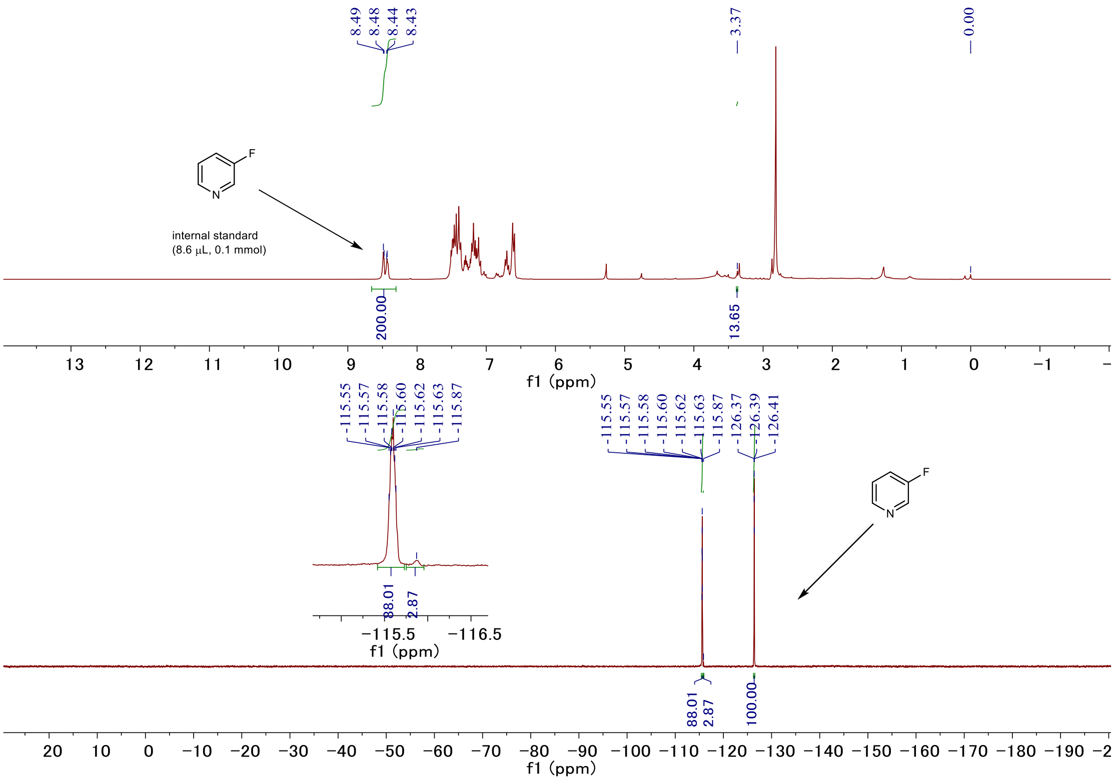

Supplementary Figure 12. ^{1}H NMR (300 MHz, $CDCl_3$, 25 °C) and ^{19}F NMR (282 MHz, $CDCl_3$, 25 °C) analysis of reaction (**3**).

Note: After the reaction of (**2**) was work-up, no reaction (N.R.) was observed by TLC (eluent: *n*-hexane/DCM: 10/1).

4.5 Radical clock experiments

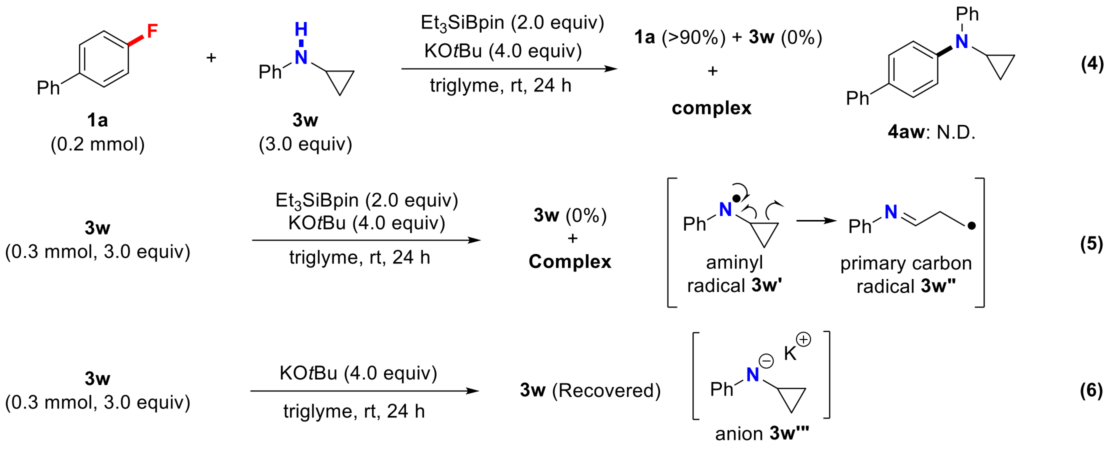

Supplementary Figure 13. Evaluate of allyl fluoride or cinnamyl fluoride for this reaction.

Reaction (**4**): Following the **General procedure E**, charging **1a** (0.2 mmol), **3w** (80.0 mg, 0.6 mmol), silyl boronate $Et_3SiBpin$ (96.8 mg, 0.4 mmol), KO*t*Bu (90.0 mg, 0.8 mmol), and then anhydrous triglyme (1.0 mL) sequentially into a flame-dried screw-capped test tube. And then move out from glovebox and stirred at room temperature for 24 h. The reaction tube was diluted with Et_2O (5 mL), then extracted with Et_2O, washed with brine, dried over Na_2SO_4, then concentrated under vacuum, followed by adding 3-fluoropyridine (8.6 μL, 0.1 mmol) as an internal standard, then ^{19}F NMR and ^{1}H NMR analysis was taken to show the reaction details.

Reaction (**5**) and (**6**): Following the **General procedure E**, charging **3w** (40.0 mg, 0.3 mmol), with or without silyl boronate $Et_3SiBpin$ (48.4 mg, 0.2 mmol), KO*t*Bu (44.8 mg, 0.8 mmol), and then anhydrous triglyme (0.5 mL) sequentially into a flame-dried screw-capped test tube. And then move out from glovebox and stirred at room temperature for 24 h. The reaction tube was diluted with Et_2O (5 mL), then extracted with Et_2O, washed with brine, dried over Na_2SO_4, then concentrated under vacuum, followed by adding 3-fluoropyridine (8.6 μL, 0.1 mmol) as an internal standard, then ^{1}H NMR analysis was taken to show the reaction details.

Note: After the reaction of **(6)** was work-up, starting material **3w** was observed by TLC (eluent: *n*-hexane/DCM: 10/1).

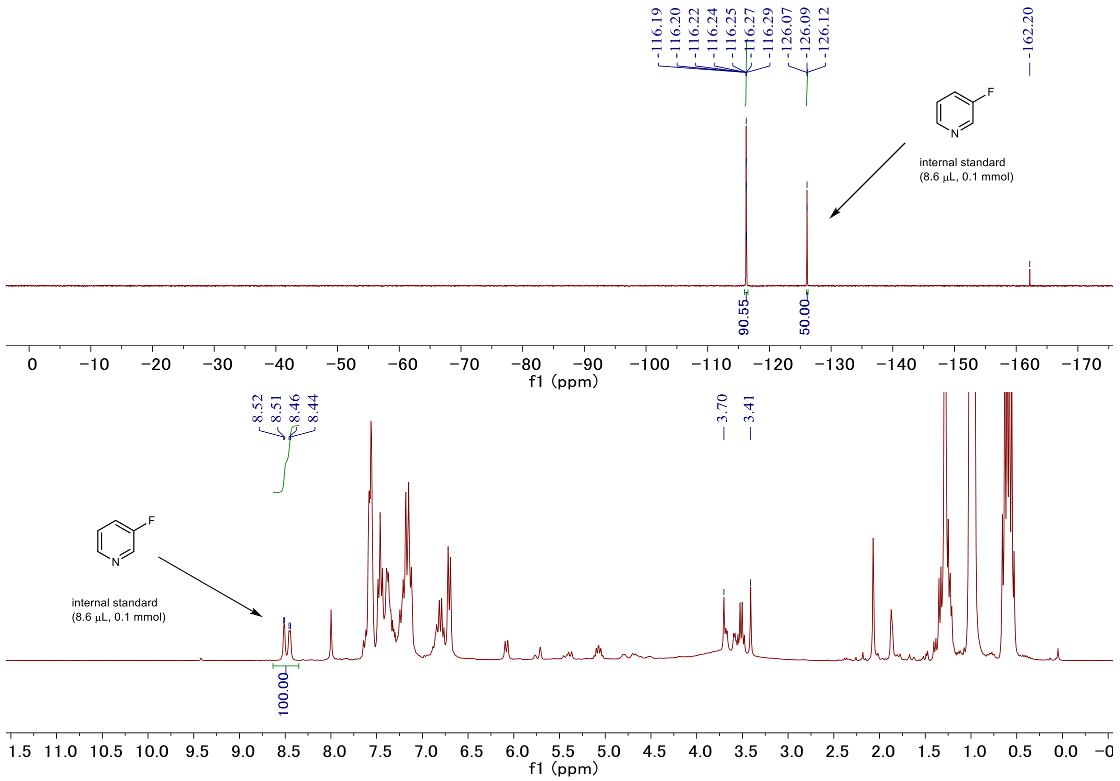

Supplementary Figure 14. ^{19}F NMR (282 MHz, $CDCl_3$, 25 °C) and ^{1}H NMR (300 MHz, $CDCl_3$, 25 °C) analysis of the reaction (**4**).

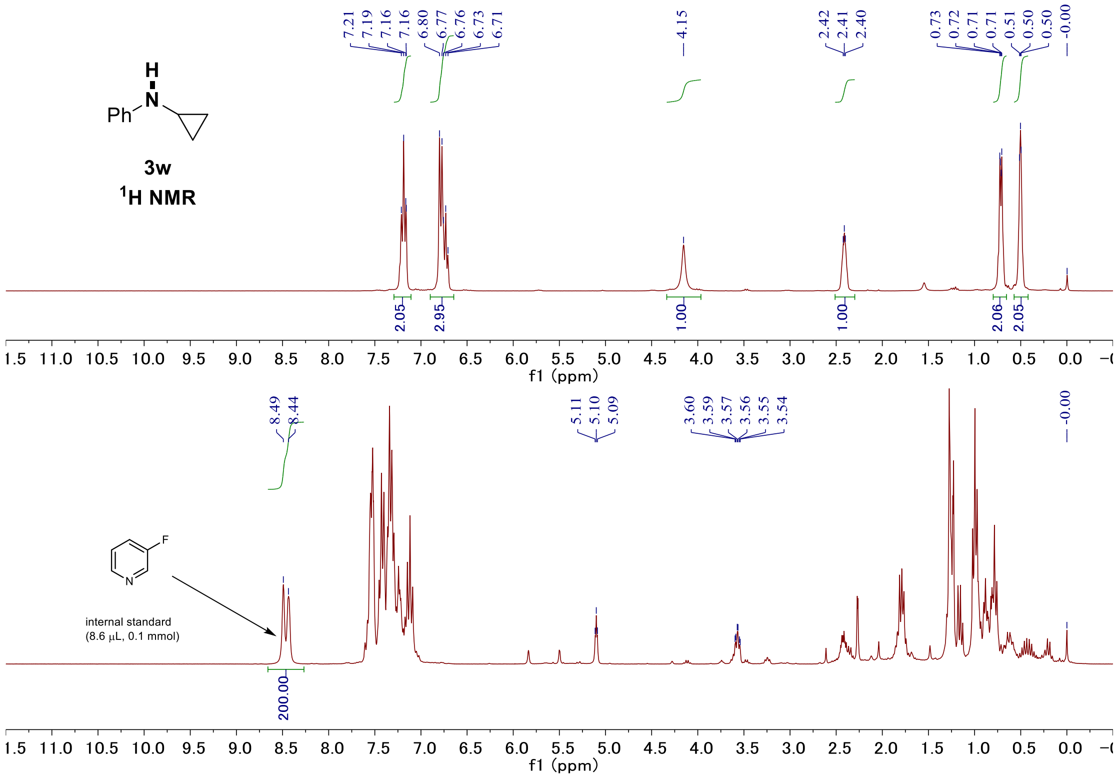

Supplementary Figure 15. ^{1}H NMR (300 MHz, $CDCl_3$, 25 °C) of **3w** and the ^{1}H NMR (300 MHz, $CDCl_3$, 25 °C) analysis details of reaction (**5**).

The reaction of **1a** with **3w** under standard conditions did not give the desired product **4aw**, and majority of **1a** were remains and recovered (around 90%, **^{19}F NMR** yield). **^{1}H NMR** spectrum shows complex mixtures, and no desired product **4aw** formed (Similar characteristic peak should be around 2.77 ppm, see *Tetrahedron*, **2006**, *62*, 4253–4261 and *Chem. Eur. J.* **2018**, *24*, 13744–13748 for the details.) However, **3w** disappeared completely. Thus, it is difficult to colorlessly assigned the products in this reaction.

When treated only **3w** under standard conditions, resulting in the complex mixture and **3w** disappearing. This fact is consistent with the literature that the generated aminyl radical **3w'** spontaneously transforms into the ring-opening primary carbon radical **3w"** (*J. Am. Chem. Soc.* **1980**, *102*, 328–331; *Chem. Soc. Rev.* **2022**, *51*, 7344–7357; and *Acc. Chem. Res.* **2016**, *49*, 1957−1968). Thus, the amine radical **3w'** does not exist anymore, and the resulting ring-opening primary radical **3w"** is unstable and decomposes into the complex mixture.

Additionally, when treated only **3w** under standard conditions without $Et_3SiBpin$ for 24 h, and as a result, **3w** was recovered after work-up process. This is reasonable since the anion **3w"'** is stable.

4.6 Copy of NMR spectra

3-Fluoro-1,1'-biphenyl (1b)

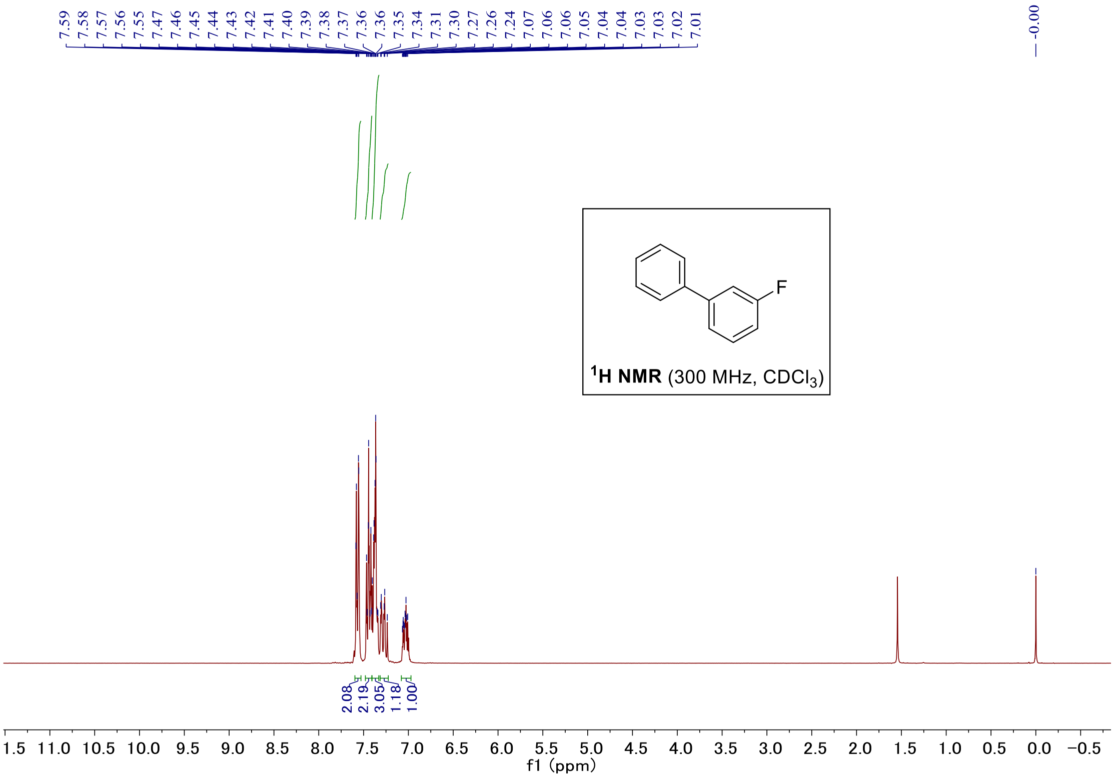

Supplementary Figure 16. ^{1}H NMR (300 MHz, $CDCl_3$, 25 °C) of compound **1b**

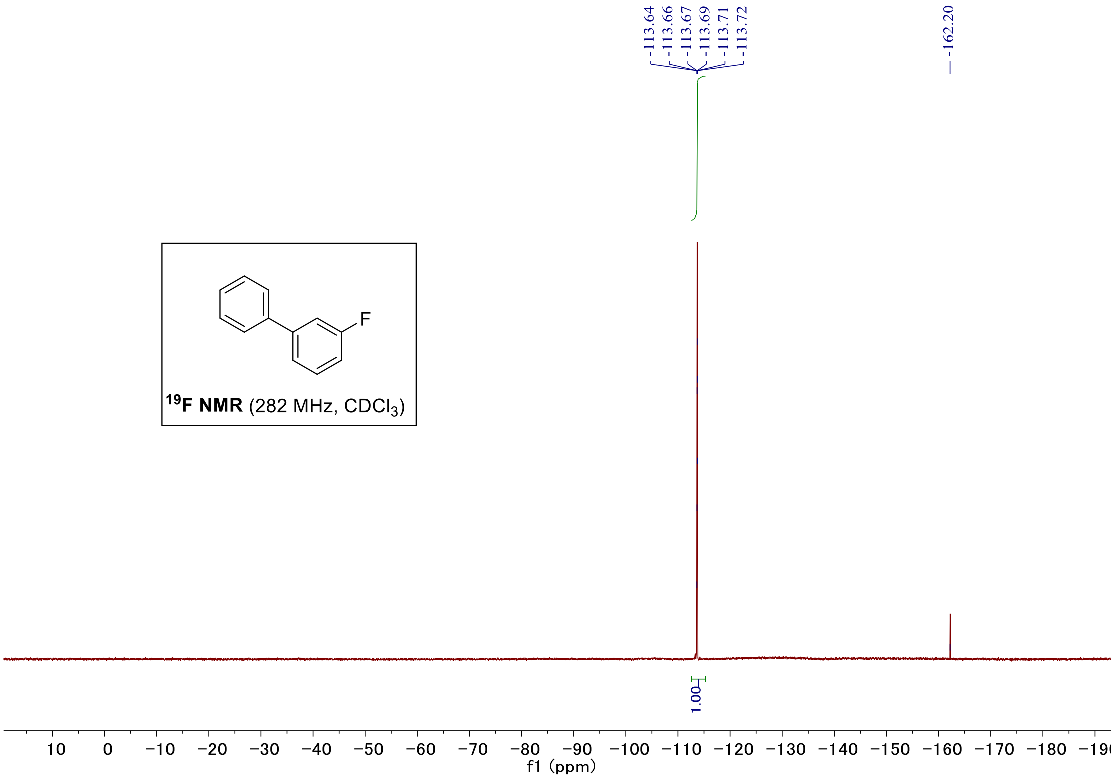

Supplementary Figure 17. ^{19}F NMR (282 MHz, $CDCl_3$, 25 °C) of compound **1b**

1-(4-Fluorophenyl)naphthalene (1k)

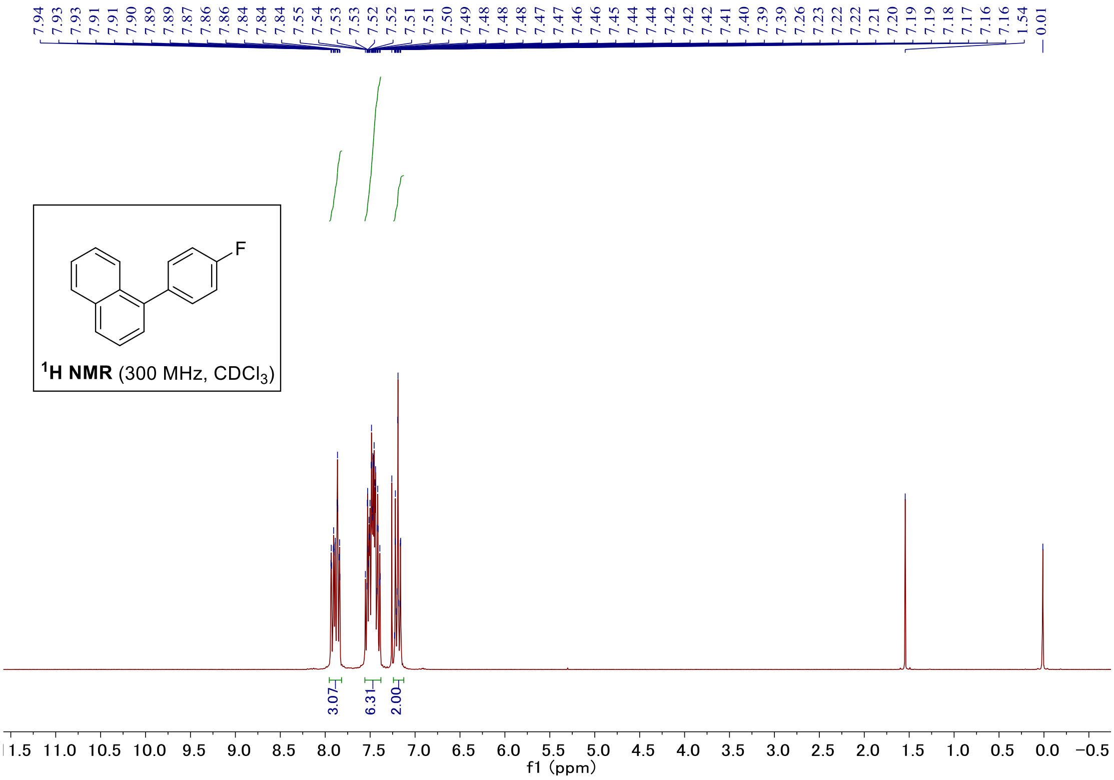

Supplementary Figure 18. ^{1}H NMR (300 MHz, $CDCl_3$, 25 °C) of compound **1k**

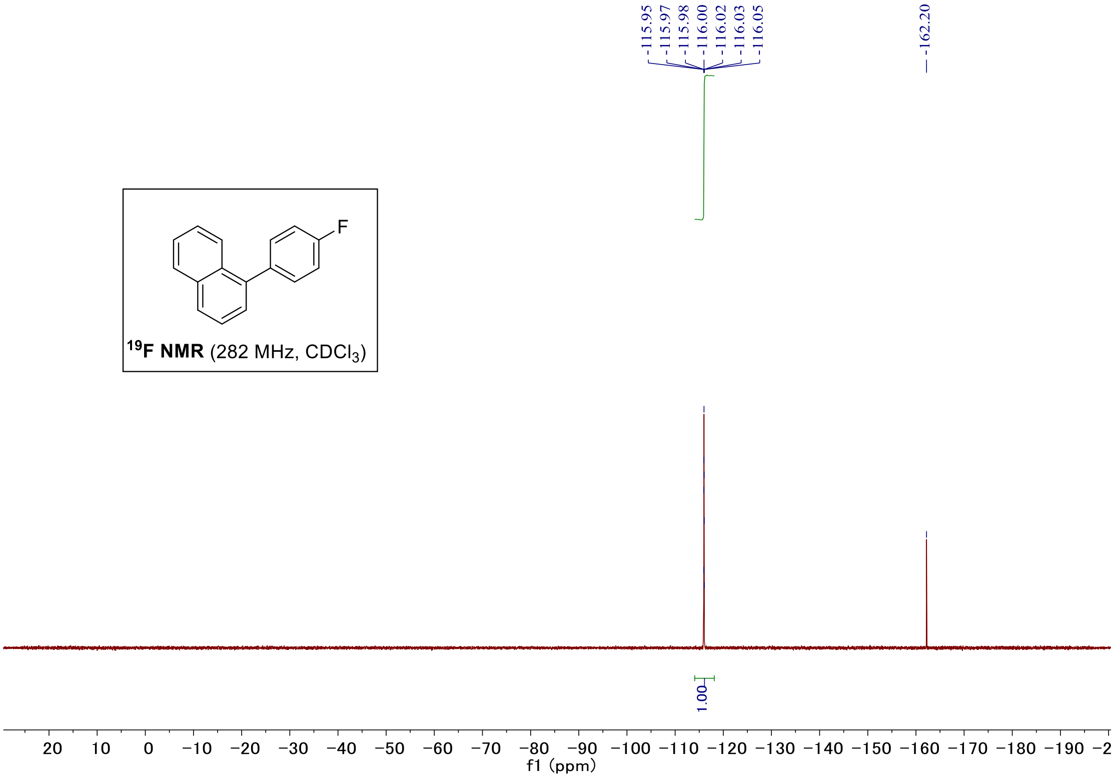

Supplementary Figure 19. ^{19}F NMR (282 MHz, $CDCl_3$, 25 °C) of compound **1k**

4-Fluoro-4'-methyl-1,1'-biphenyl (1l)

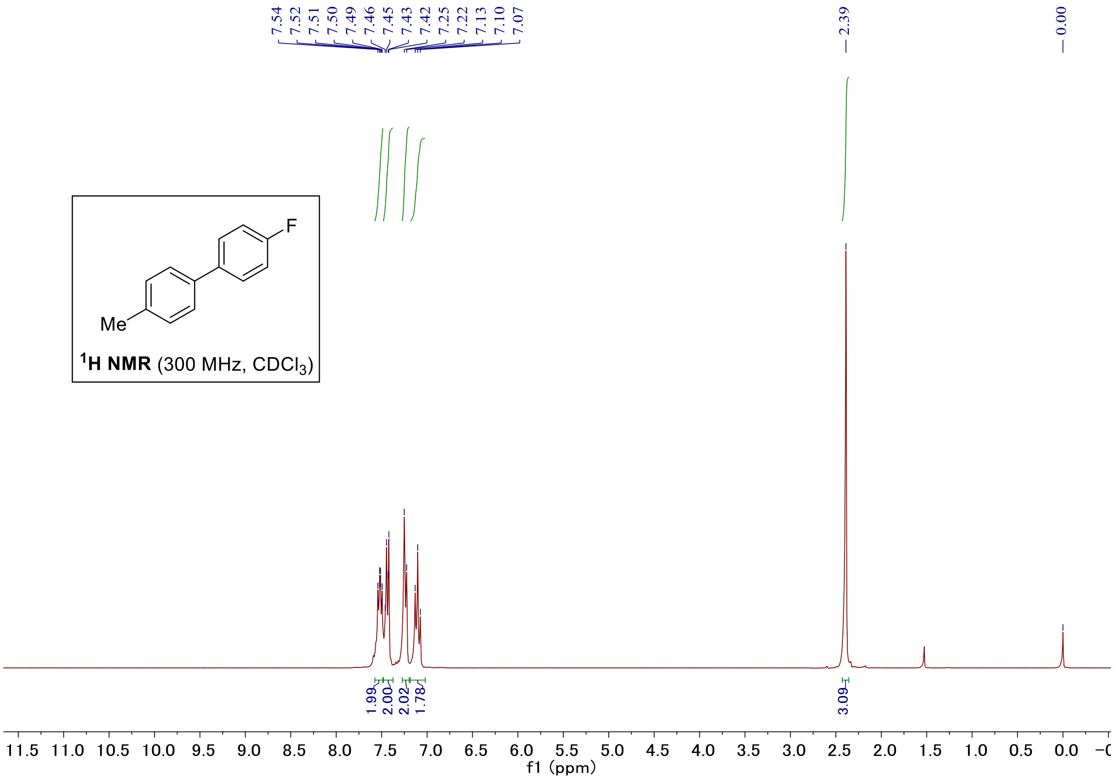

Supplementary Figure 20. ^{1}H NMR (300 MHz, $CDCl_3$, 25 °C) of compound **1l**

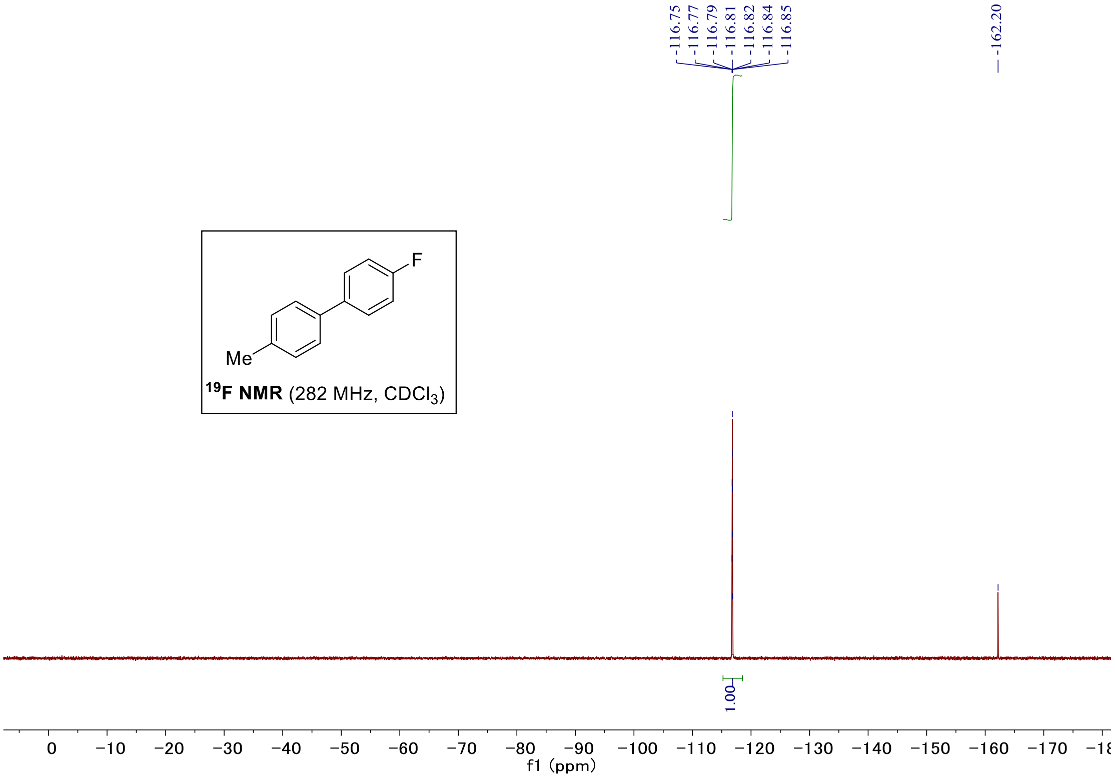

Supplementary Figure 21. ^{19}F NMR (282 MHz, $CDCl_3$, 25 °C) of compound **1l**

4-Fluoro-4'-methoxy-1,1'-biphenyl (1m)

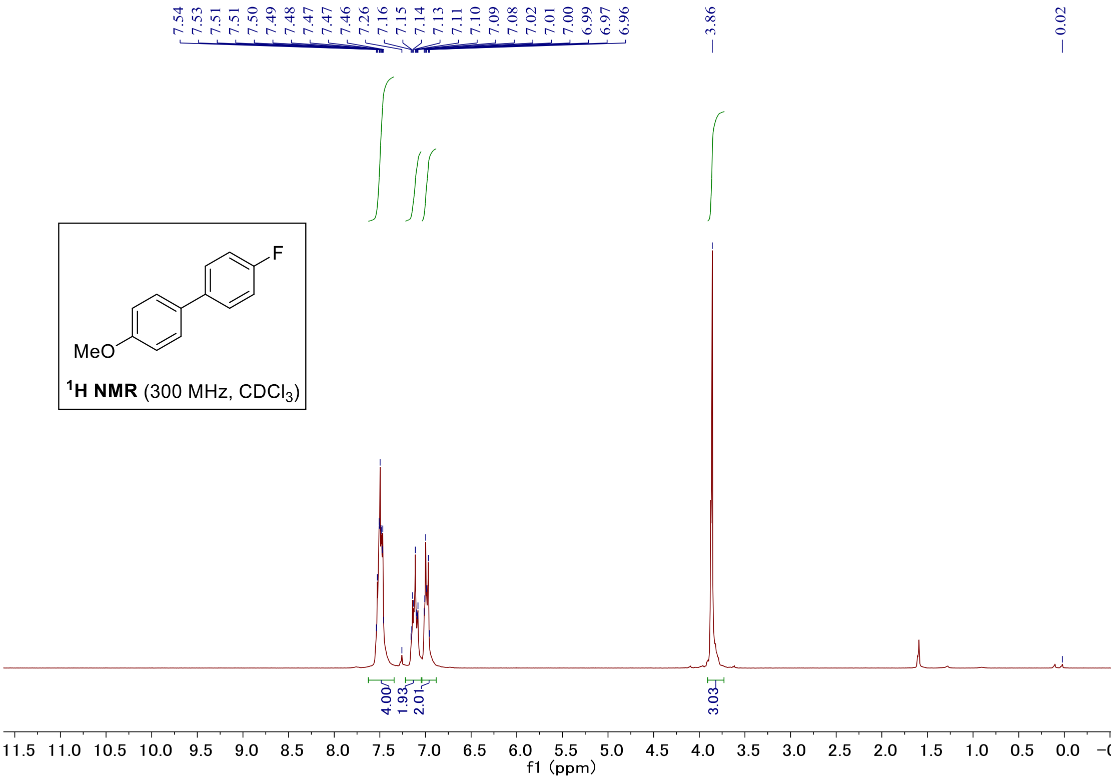

Supplementary Figure 22. ^{1}H NMR (300 MHz, $CDCl_3$, 25 °C) of compound **1m**

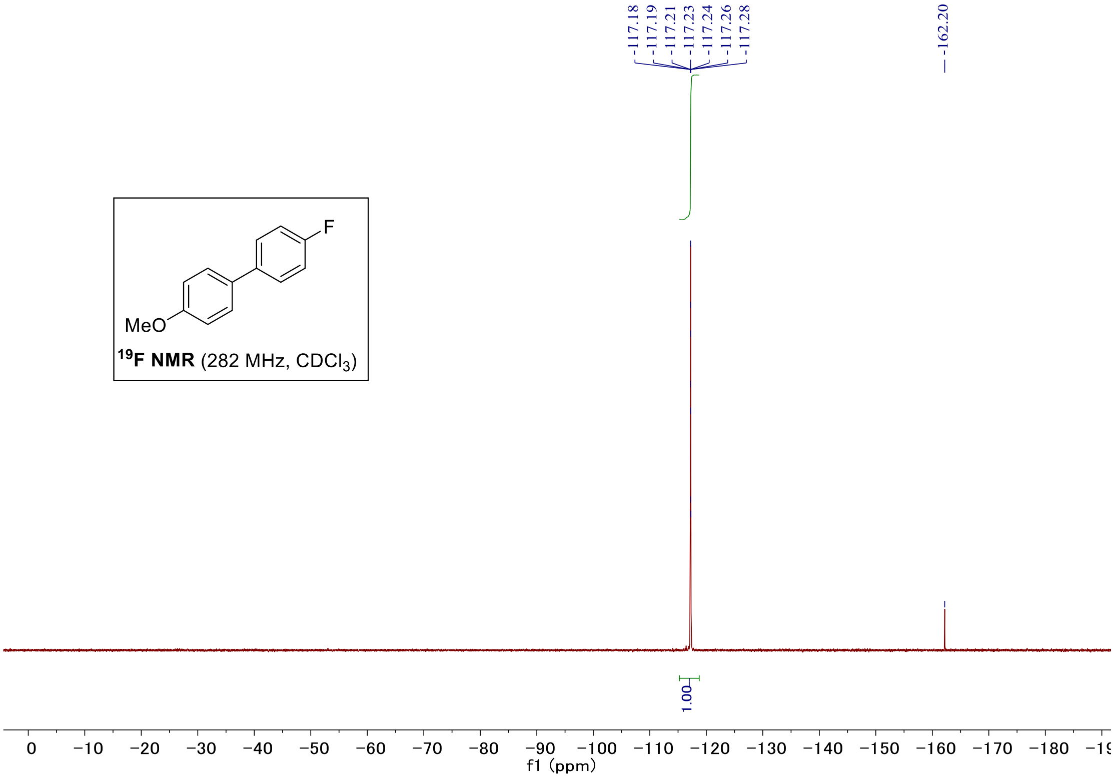

Supplementary Figure 23. ^{19}F NMR (282 MHz, $CDCl_3$, 25 °C) of compound **1m**

4-(Benzyloxy)-4'-fluoro-1,1'-biphenyl (1n)

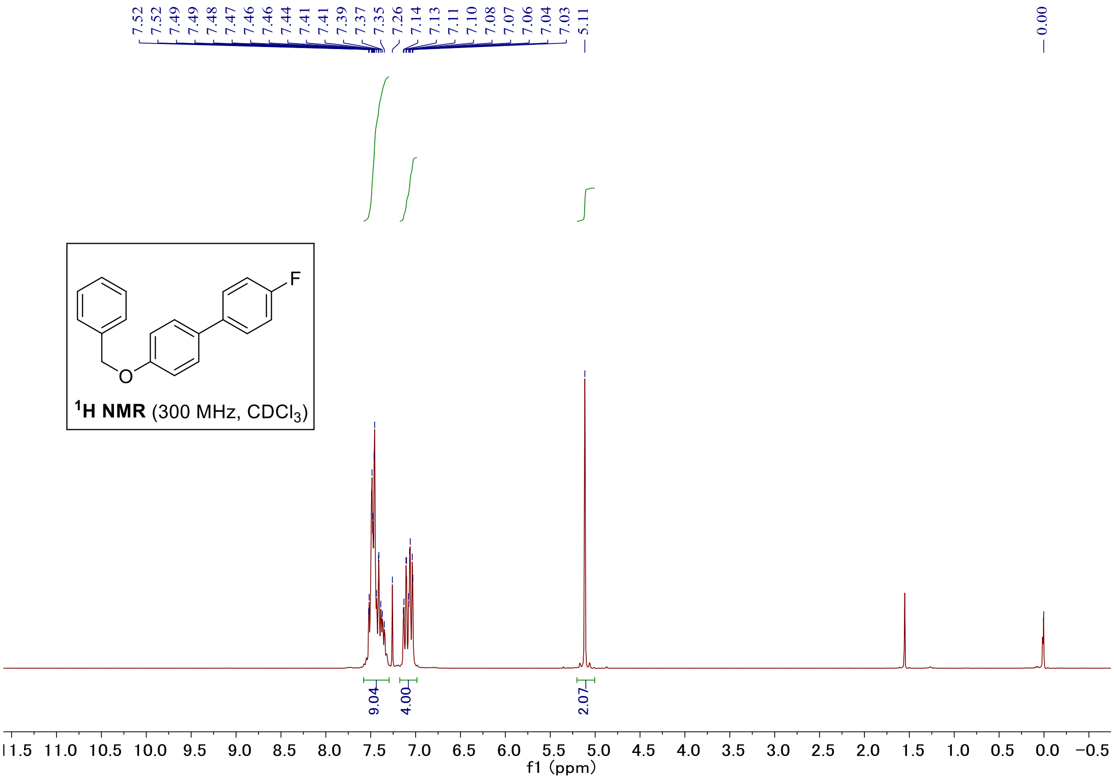

Supplementary Figure 24. ^{1}H NMR (300 MHz, $CDCl_3$, 25 °C) of compound **1n**

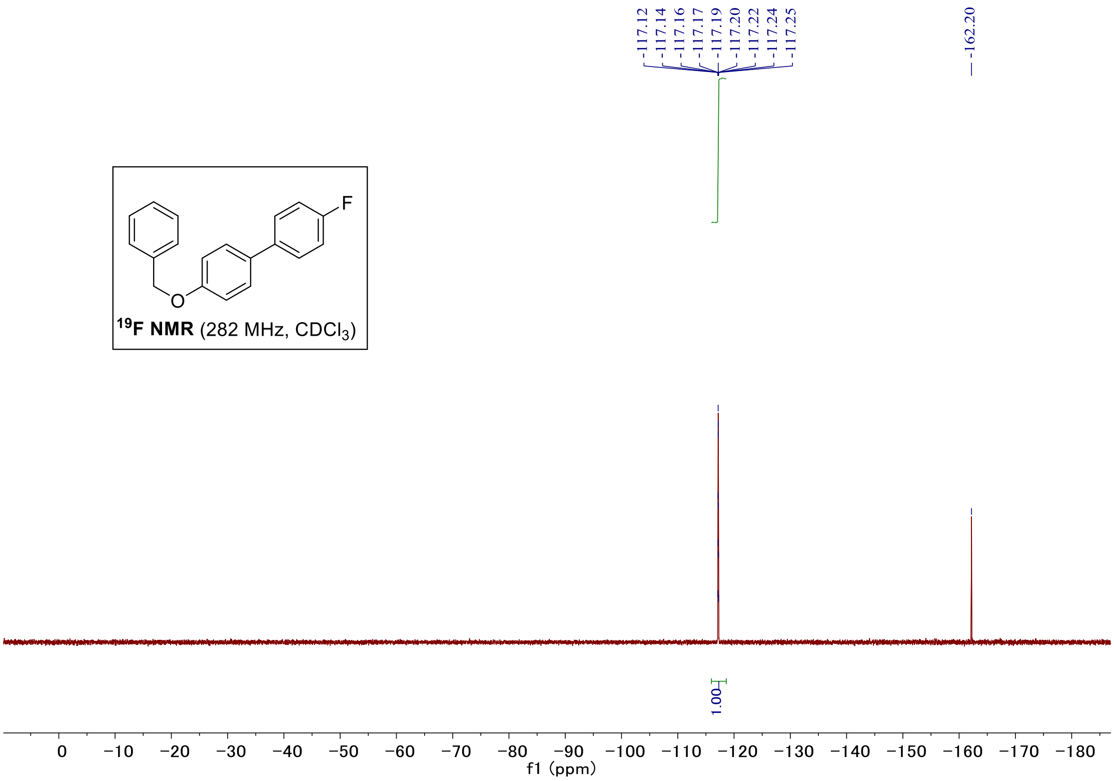

Supplementary Figure 25. ^{19}F NMR (282 MHz, $CDCl_3$, 25 °C) of compound **1n**

4-Chloro-4'-fluoro-1,1'-biphenyl (1o)

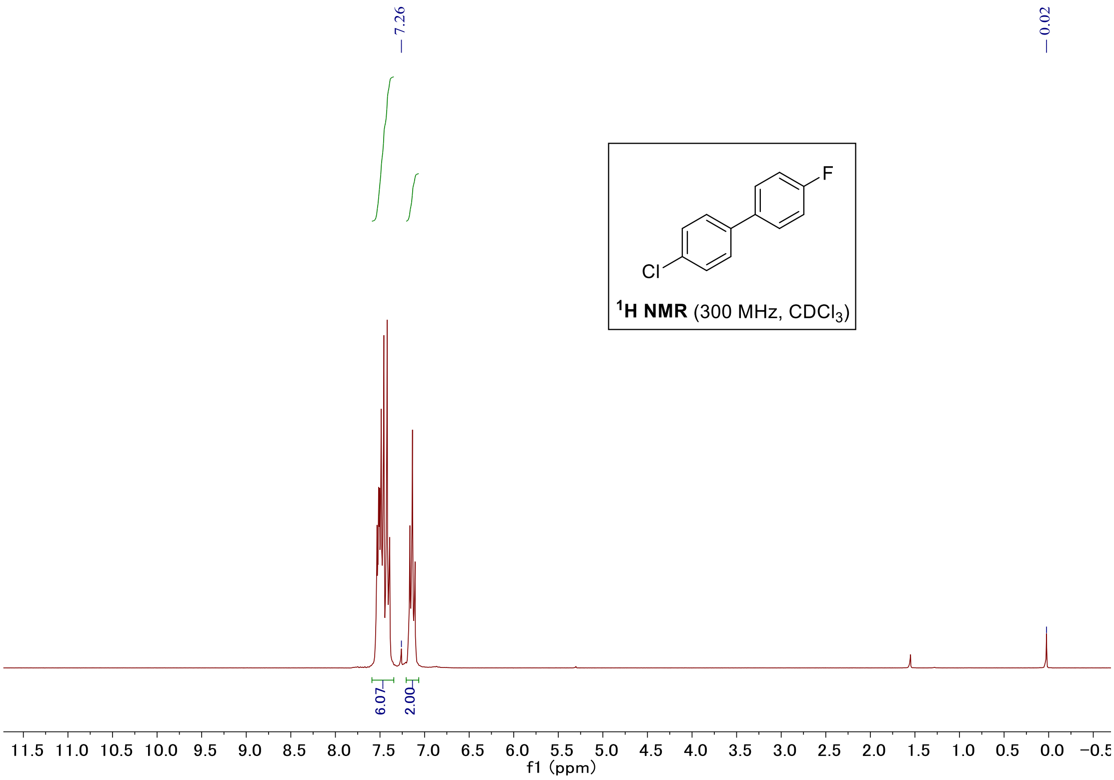

Supplementary Figure 26. ^{1}H NMR (300 MHz, $CDCl_3$, 25 °C) of compound **1o**

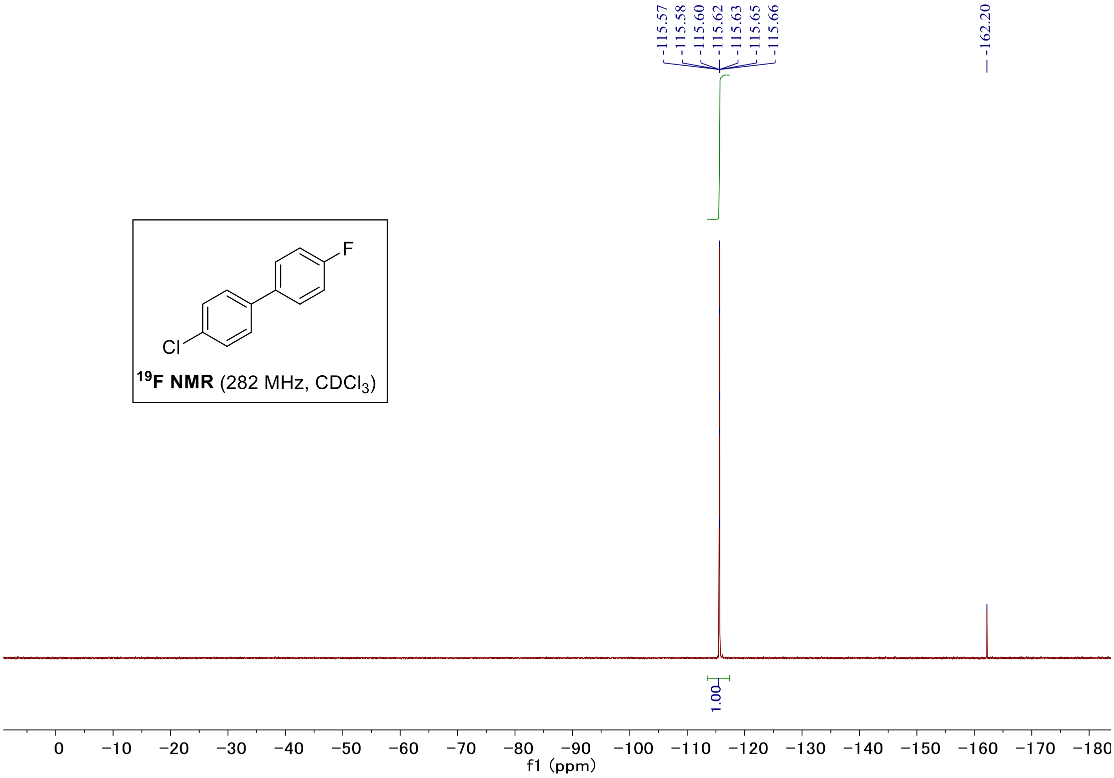

Supplementary Figure 27. ^{19}F NMR (282 MHz, $CDCl_3$, 25 °C) of compound **1o**

4-Bromo-4'-fluoro-1,1'-biphenyl (1p)

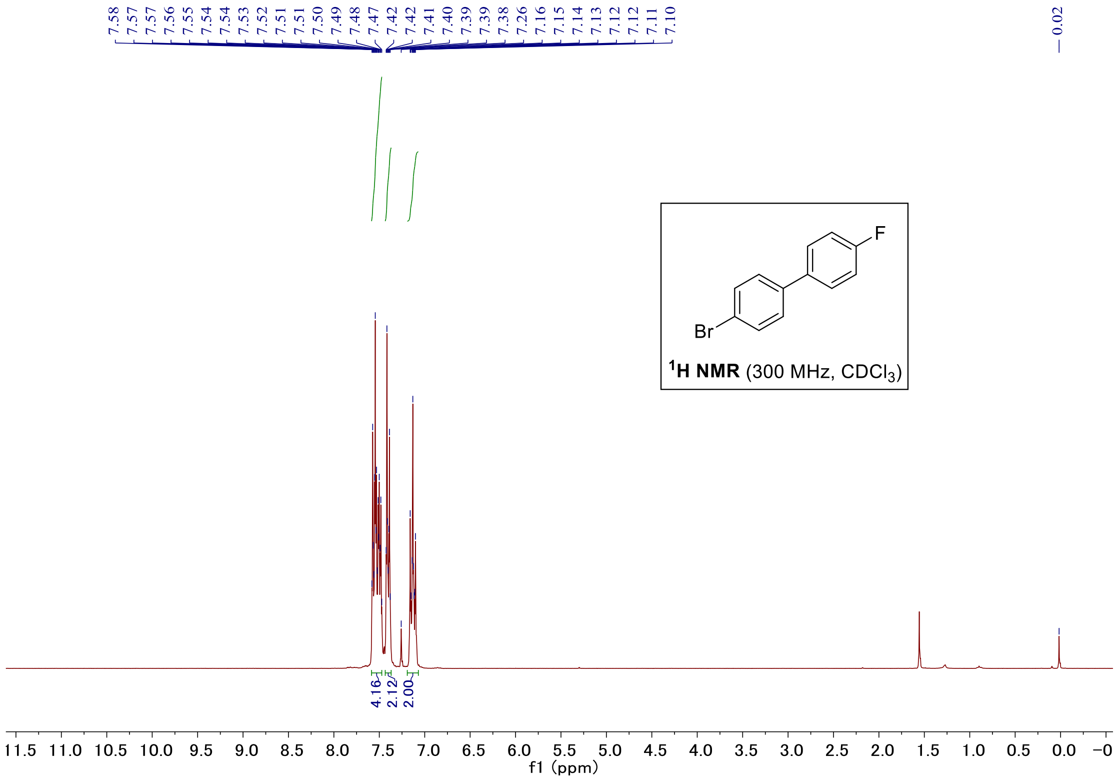

Supplementary Figure 28. ^{1}H NMR (300 MHz, $CDCl_3$, 25 °C) of compound **1p**

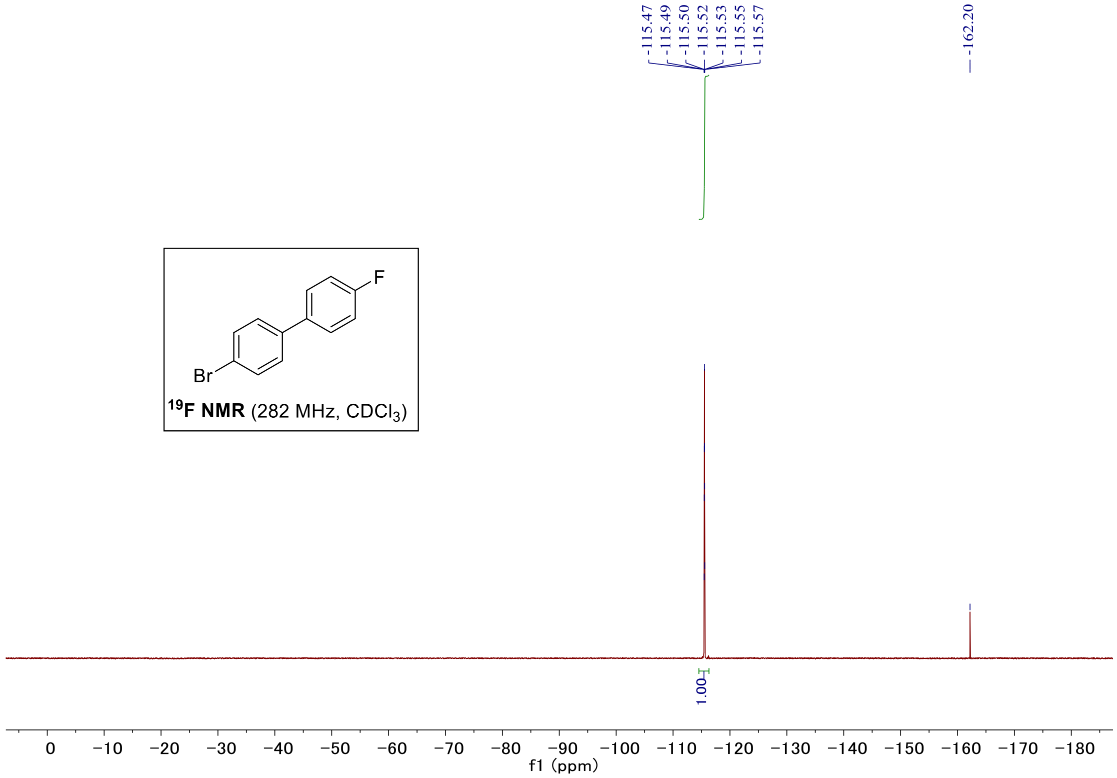

Supplementary Figure 29. ^{19}F NMR (282 MHz, $CDCl_3$, 25 °C) of compound **1p**

4'-Fluoro-[1,1'-biphenyl]-4-carbonitrile (1q)

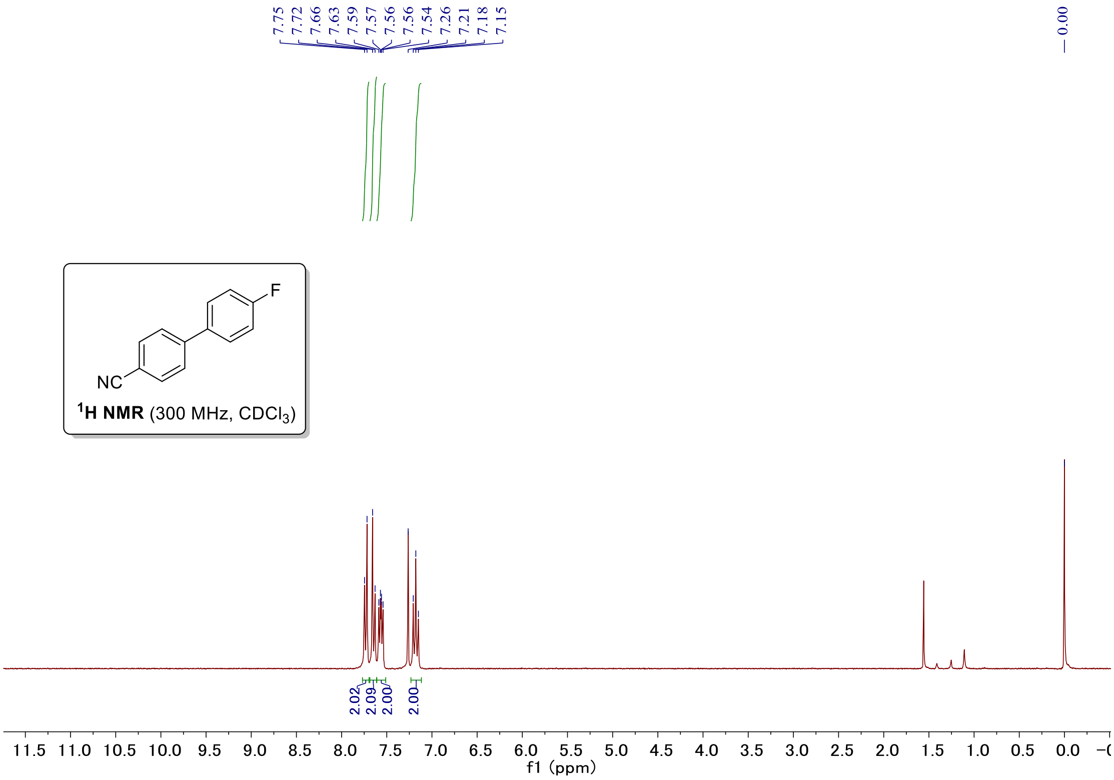

Supplementary Figure 30. ^{1}H NMR (300 MHz, $CDCl_3$, 25 °C) of compound **1q**

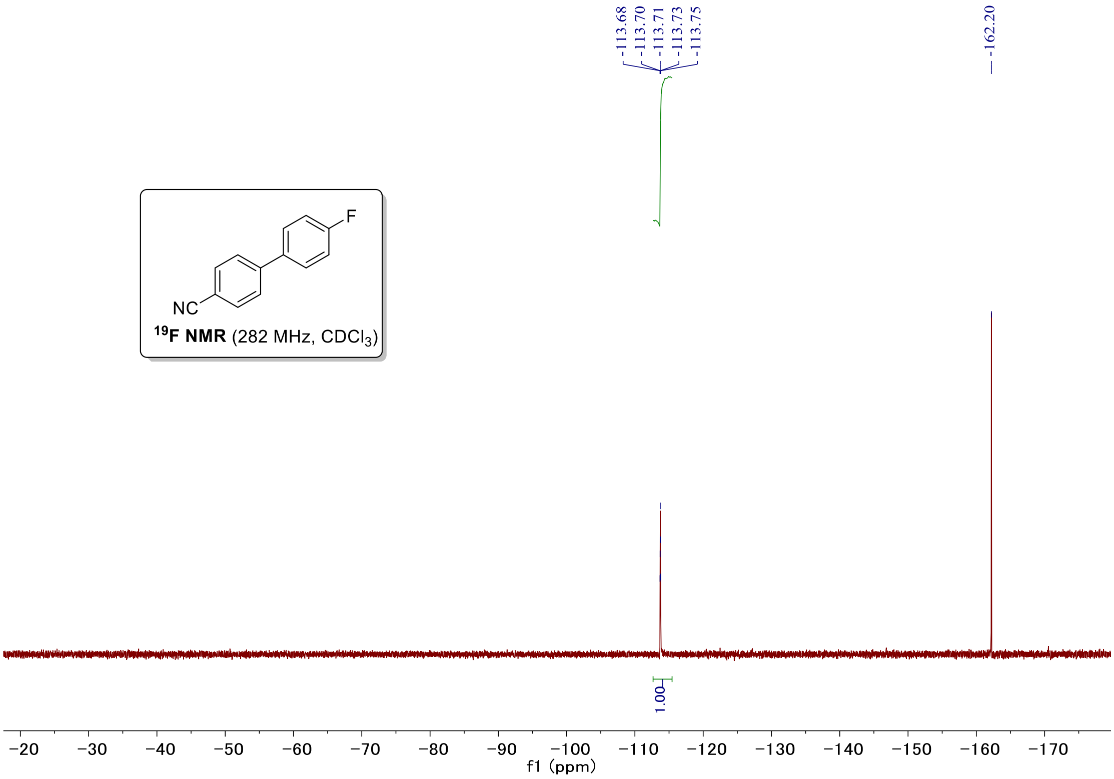

Supplementary Figure 31. ^{19}F NMR (282 MHz, $CDCl_3$, 25 °C) of compound **1q**

4-Fluoro-4'-(trifluoromethyl)-1,1'-biphenyl (1r)

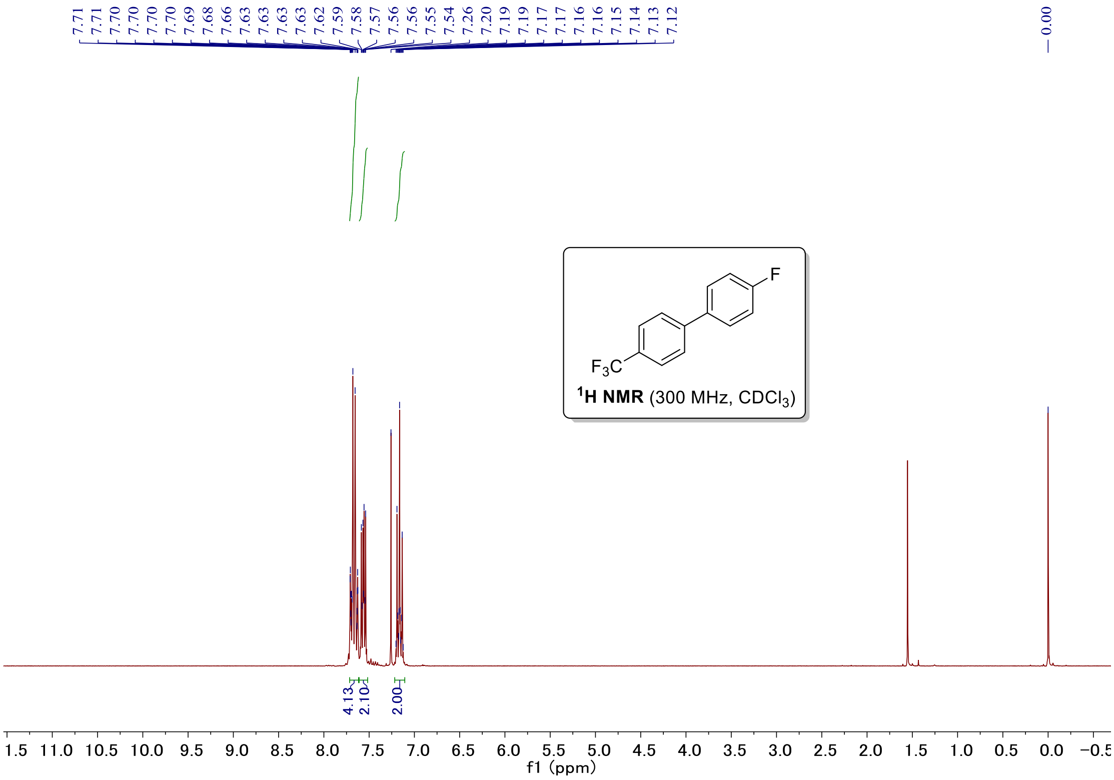

Supplementary Figure 32. ^{1}H NMR (300 MHz, $CDCl_3$, 25 °C) of compound **1r**

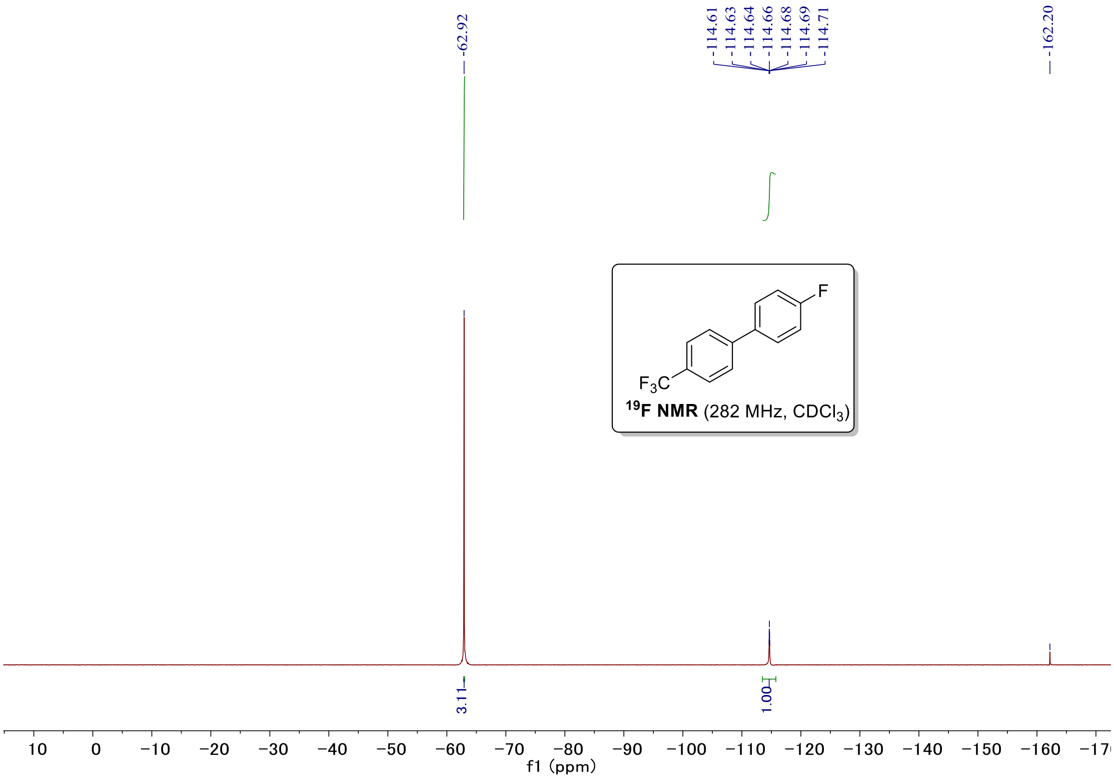

Supplementary Figure 33. ^{19}F NMR (282 MHz, $CDCl_3$, 25 °C) of compound **1r**

4-Fluoro-3'-(trifluoromethyl)-1,1'-biphenyl (1s)

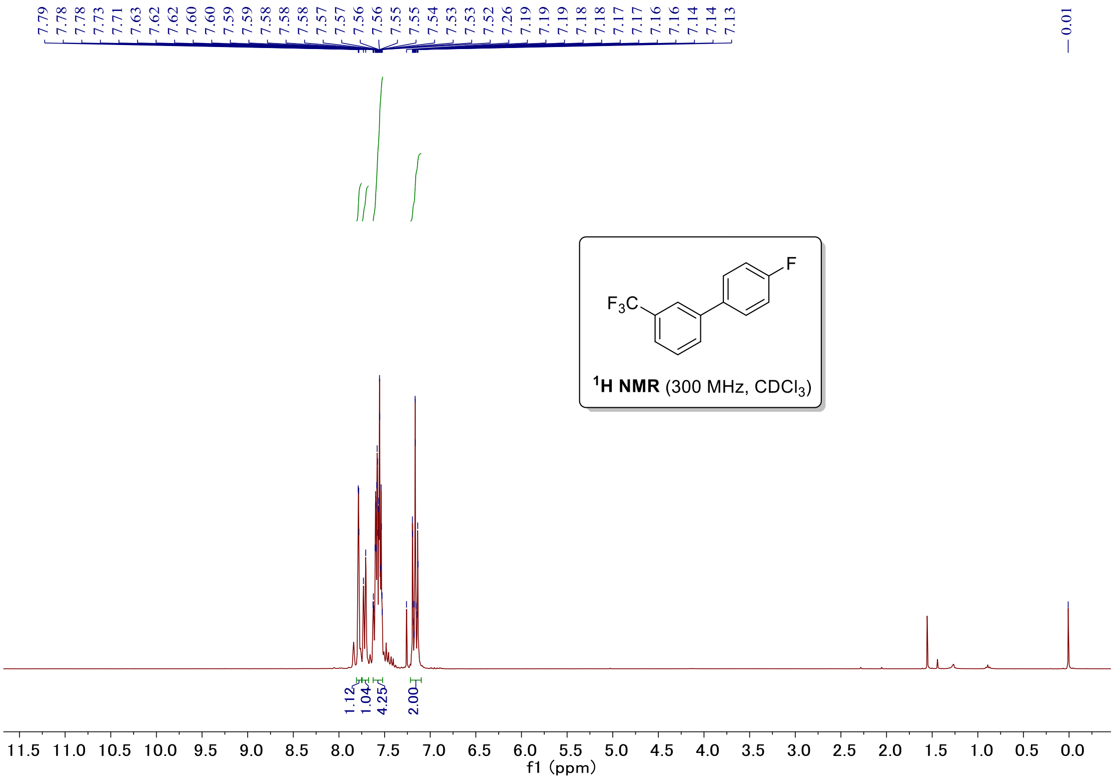

Supplementary Figure 34. ^{1}H NMR (300 MHz, $CDCl_3$, 25 °C) of compound **1s**

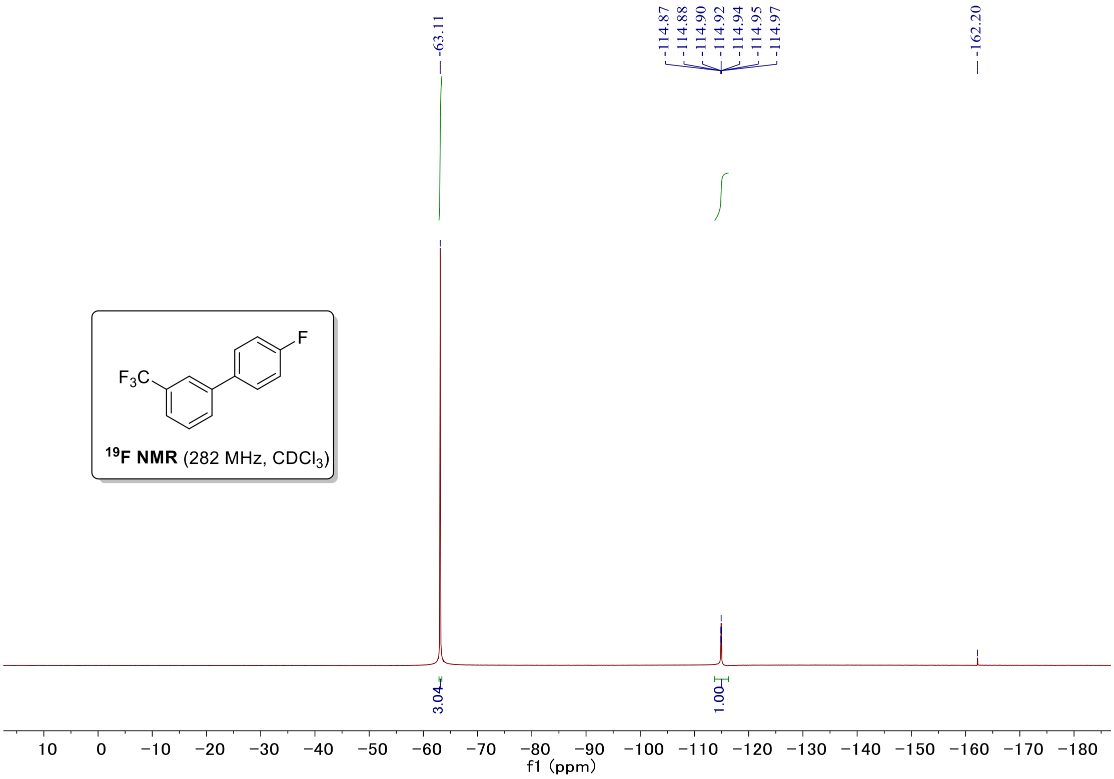

Supplementary Figure 35. ^{19}F NMR (282 MHz, $CDCl_3$, 25 °C) of compound **1s**

4'-Fluoro-3,5-bis(trifluoromethyl)-1,1'-biphenyl (1t)

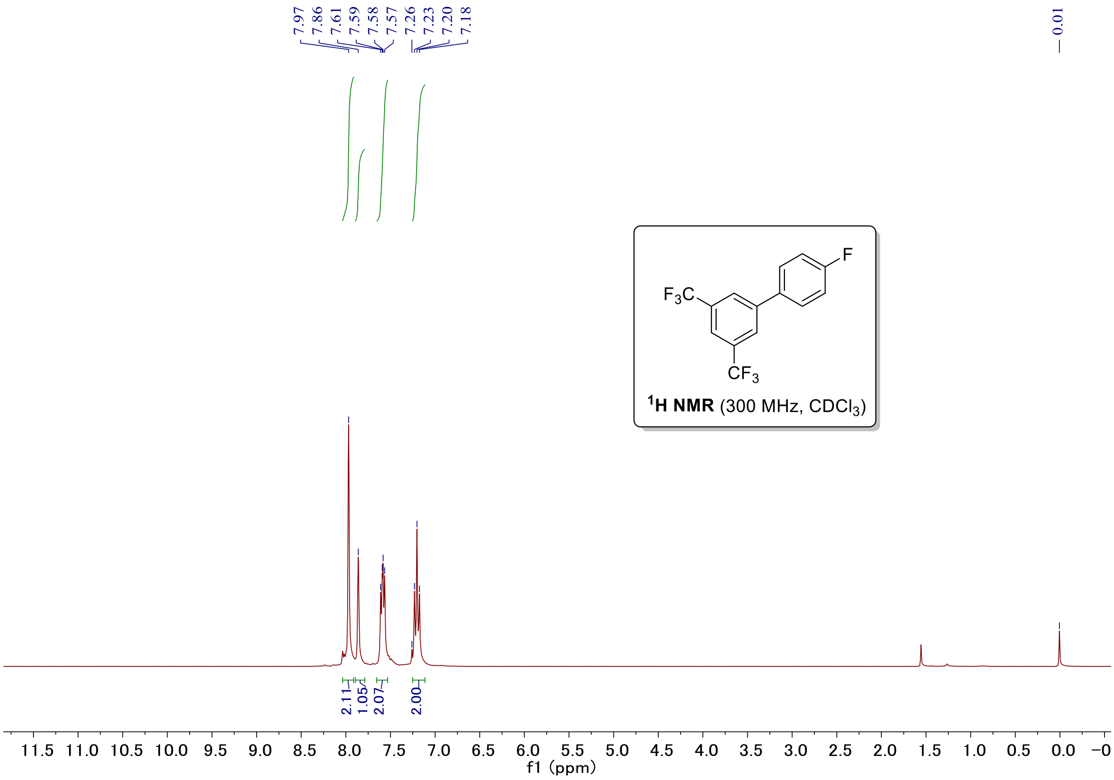

Supplementary Figure 36. ^{1}H NMR (300 MHz, $CDCl_3$, 25 °C) of compound **1t**

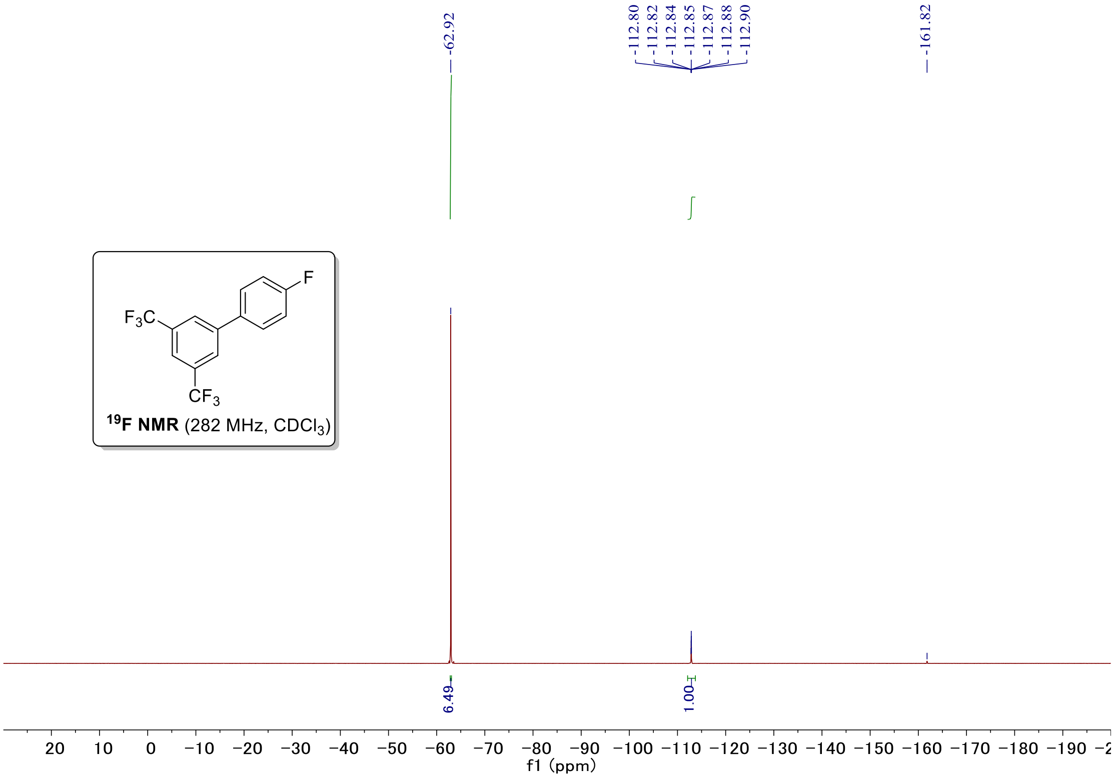

Supplementary Figure 37. ^{19}F NMR (282 MHz, $CDCl_3$, 25 °C) of compound **1t**

5-(4-Fluorophenyl)benzo[*d*][1,3]dioxole (1u)

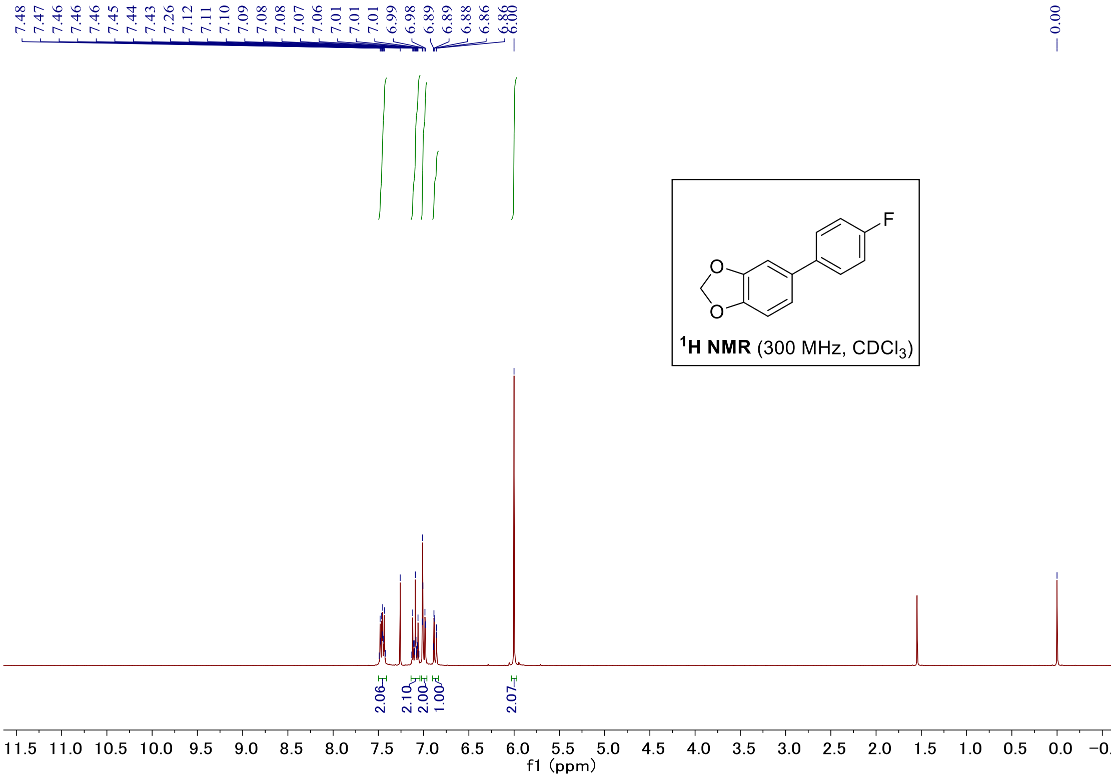

Supplementary Figure 38. ^{1}H NMR (300 MHz, $CDCl_3$, 25 °C) of compound **1u**

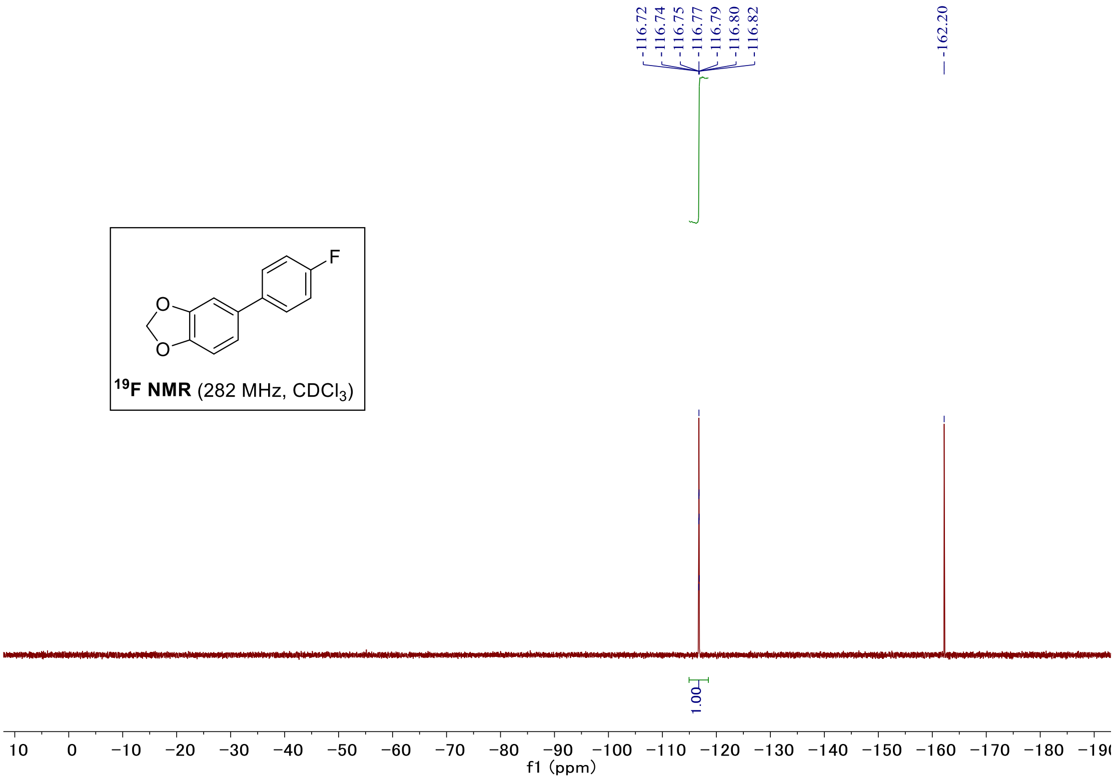

Supplementary Figure 39. ^{19}F NMR (282 MHz, $CDCl_3$, 25 °C) of compound **1u**

5-Fluoro-2-phenylpyridine (1v)

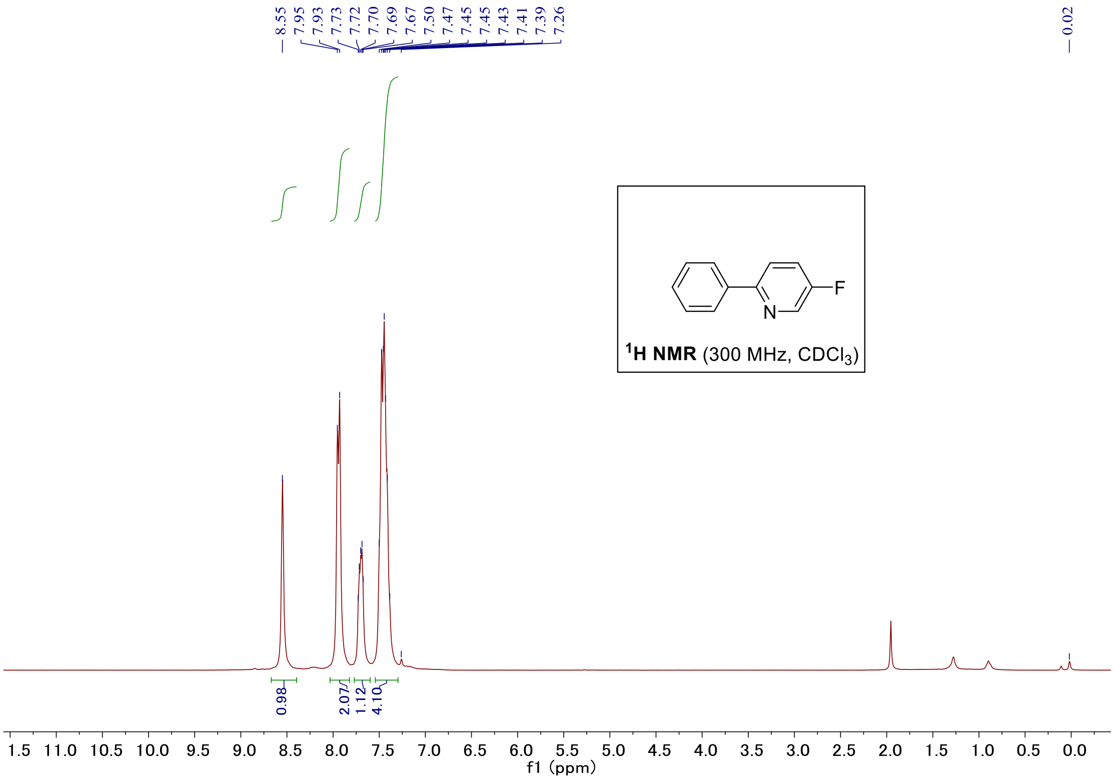

Supplementary Figure 40. ^{1}H NMR (300 MHz, $CDCl_3$, 25 °C) of compound **1v**

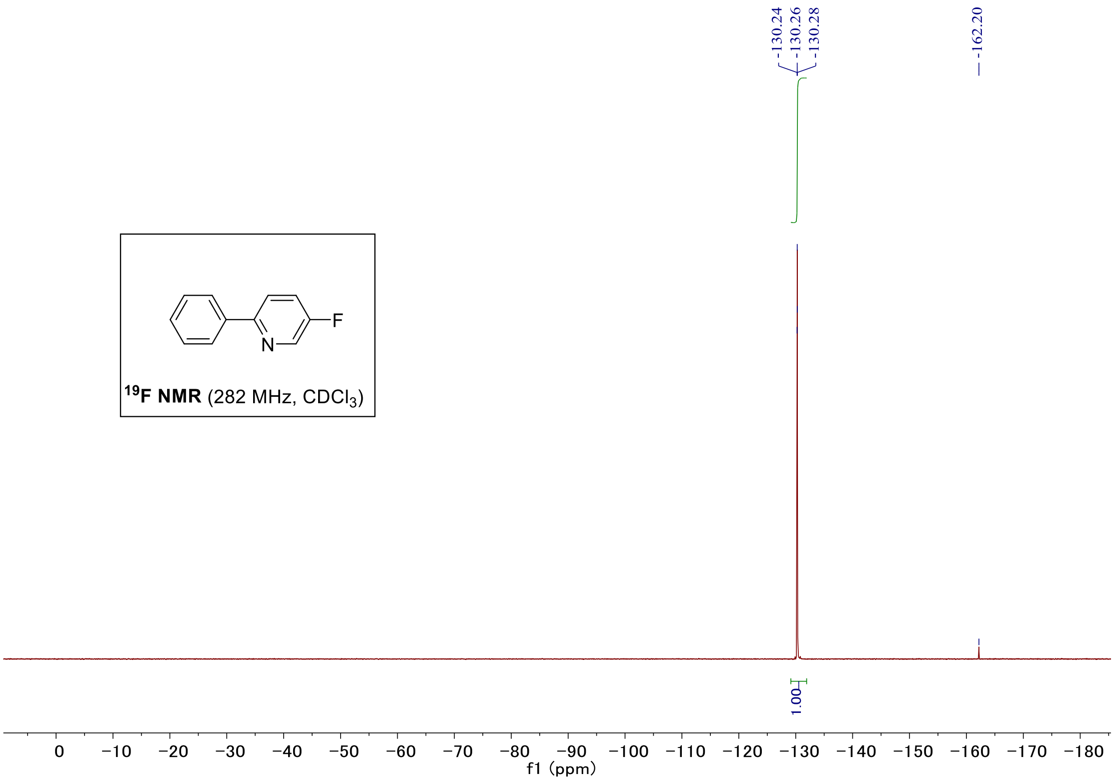

Supplementary Figure 41. ^{19}F NMR (282 MHz, $CDCl_3$, 25 °C) of compound **1v**

4-(4-Fluorophenyl)pyridine (1w)

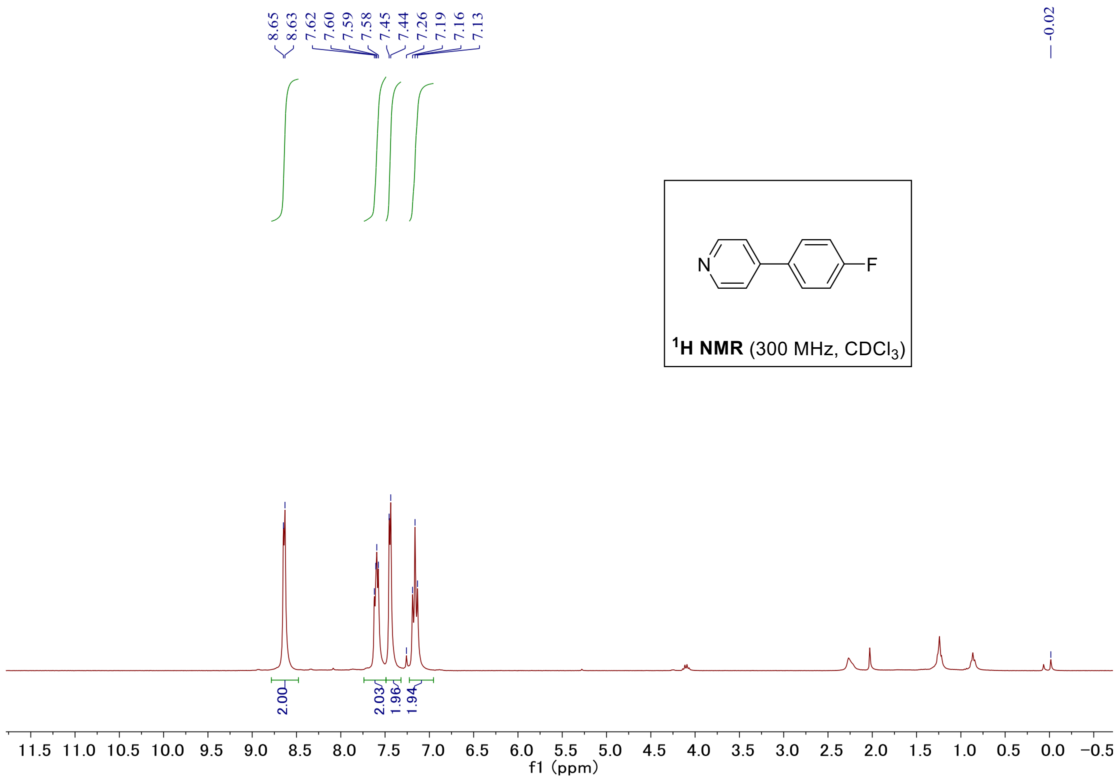

Supplementary Figure 42. ^{1}H NMR (300 MHz, $CDCl_3$, 25 °C) of compound **1w**

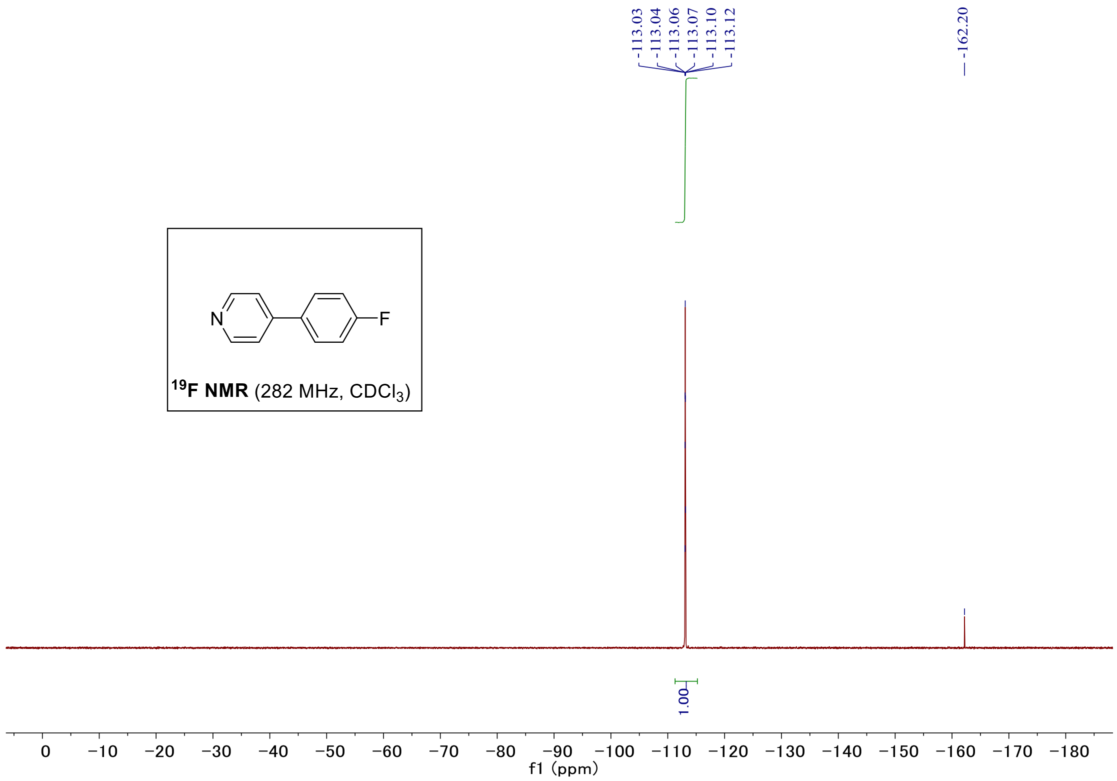

Supplementary Figure 43. ^{19}F NMR (282 MHz, $CDCl_3$, 25 °C) of compound **1w**

4-Fluoro-2-phenylpyridine (1x)

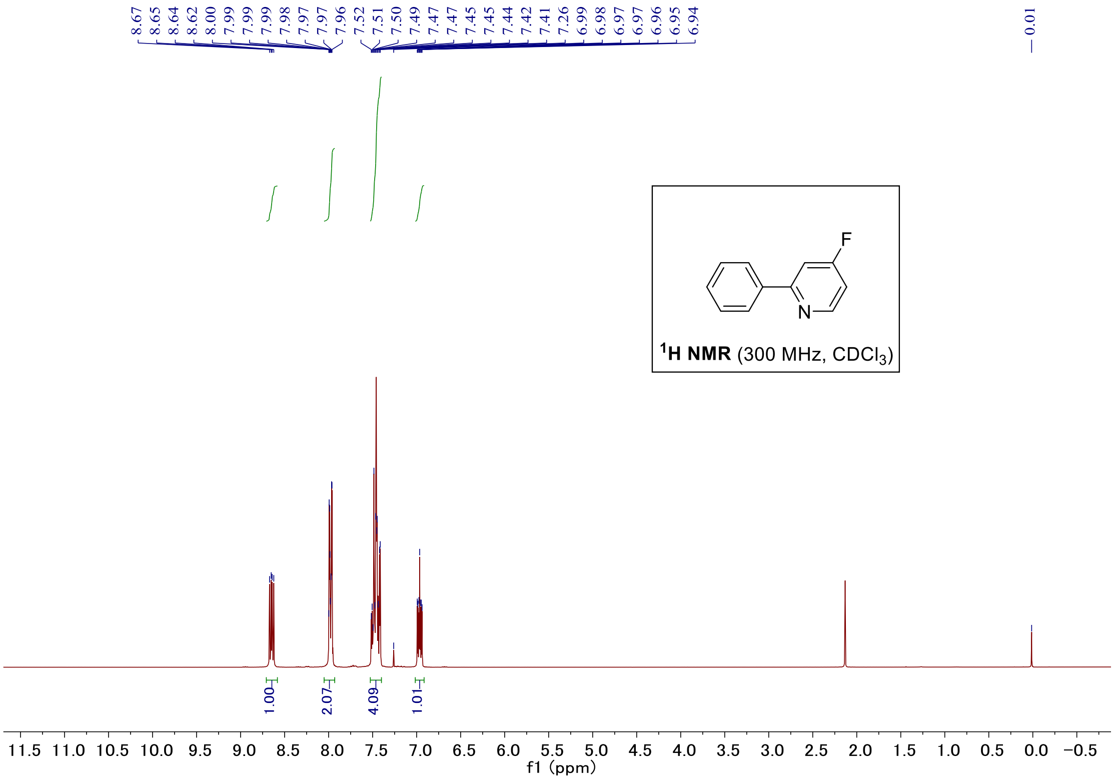

Supplementary Figure 44. ^{1}H NMR (300 MHz, $CDCl_3$, 25 °C) of compound **1x**

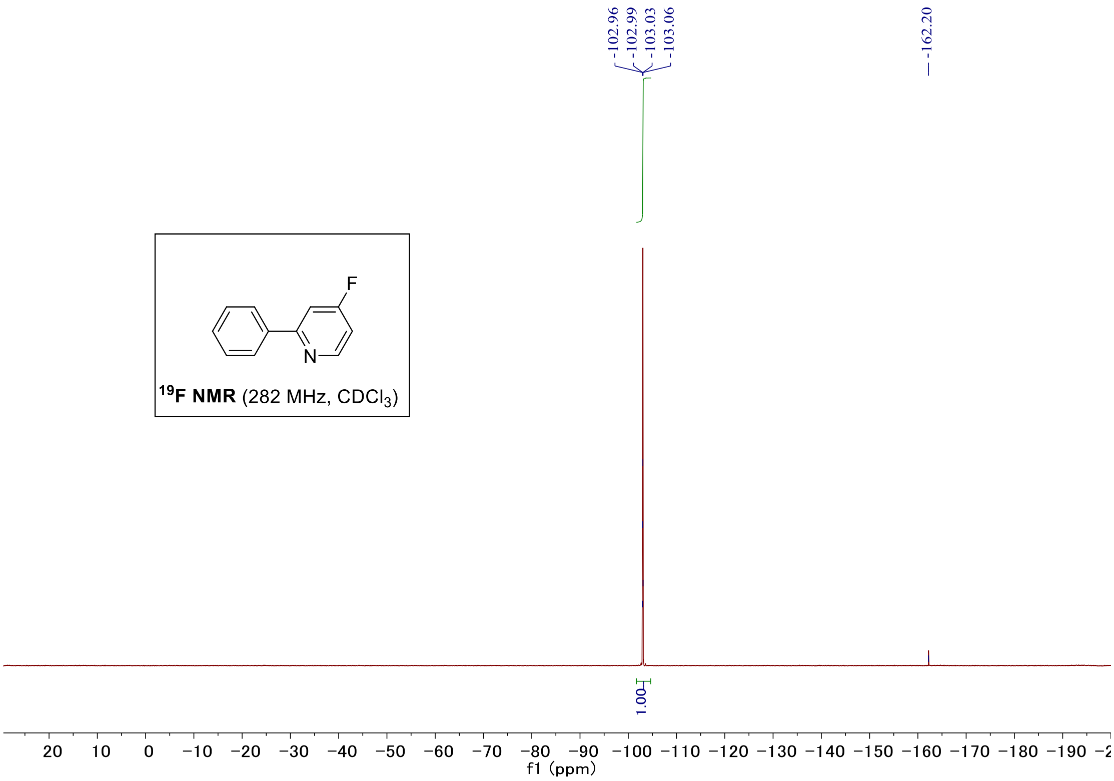

Supplementary Figure 45. ^{19}F NMR (282 MHz, $CDCl_3$, 25 °C) of compound **1x**

2-Fluoro-5-phenylpyridine (1y)

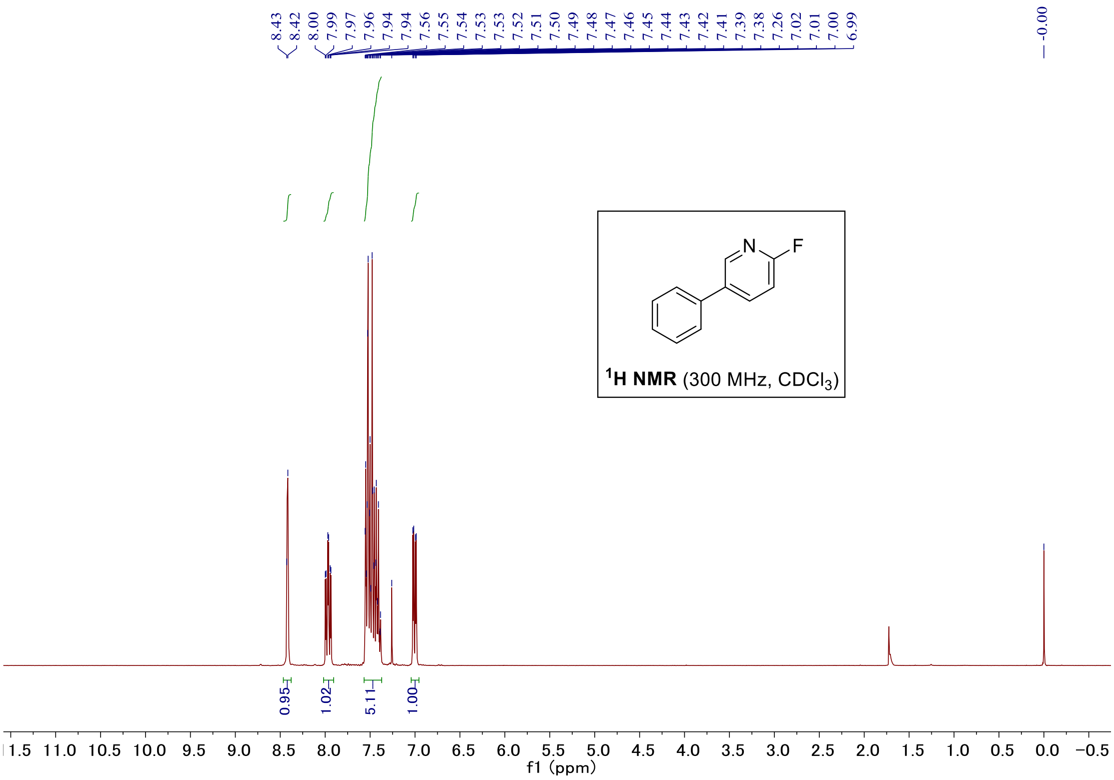

Supplementary Figure 46. ^{1}H NMR (300 MHz, $CDCl_3$, 25 °C) of compound **1y**

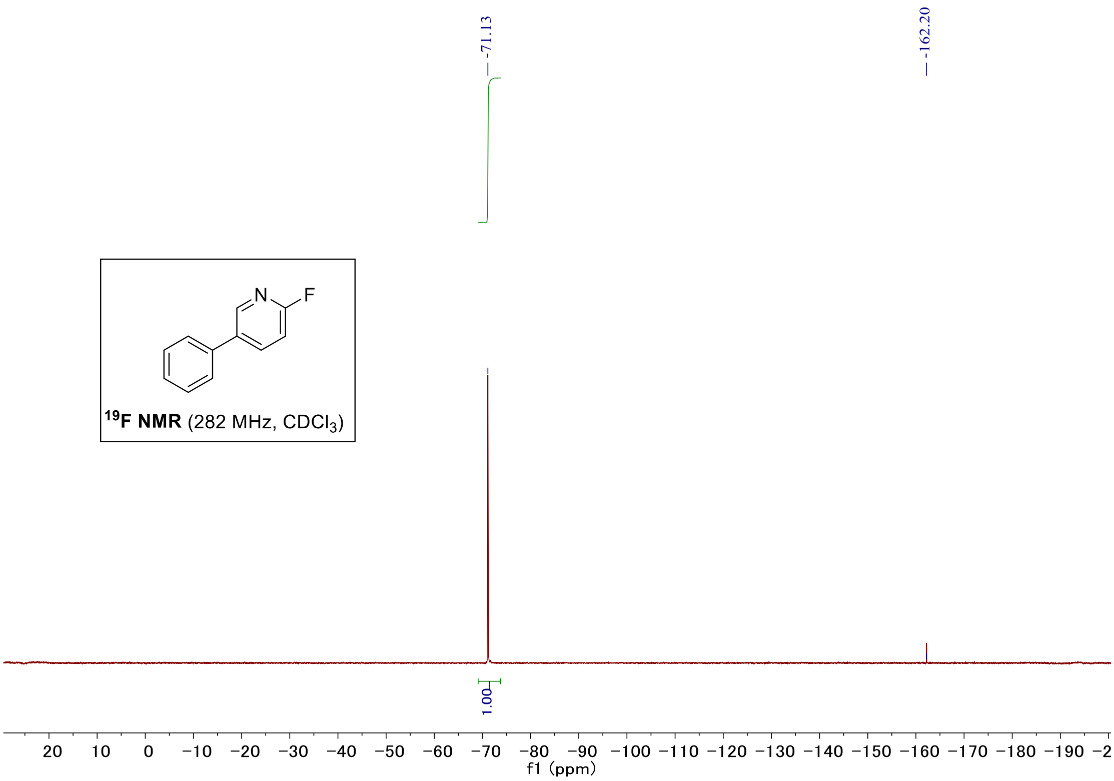

Supplementary Figure 47. ^{19}F NMR (282 MHz, $CDCl_3$, 25 °C) of compound **1y**

1-(4-Fluorophenyl)-1*H*-pyrrole (1z)

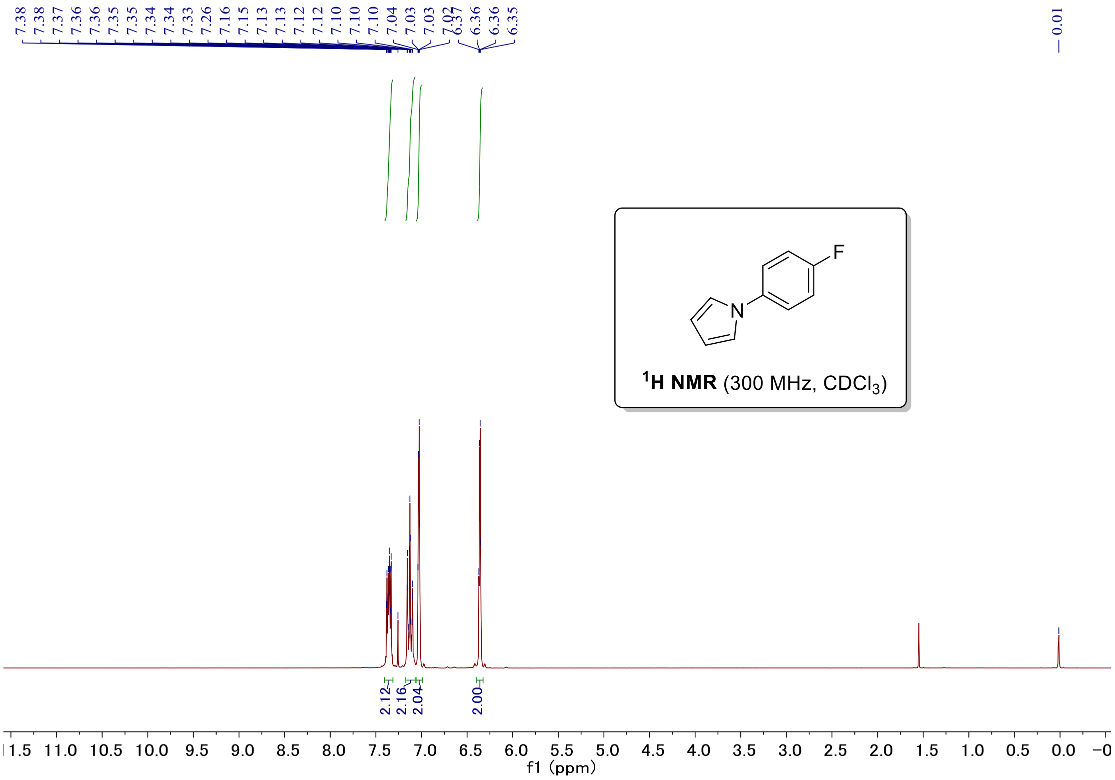

Supplementary Figure 48. ^{1}H NMR (300 MHz, $CDCl_3$, 25 °C) of compound **1z**

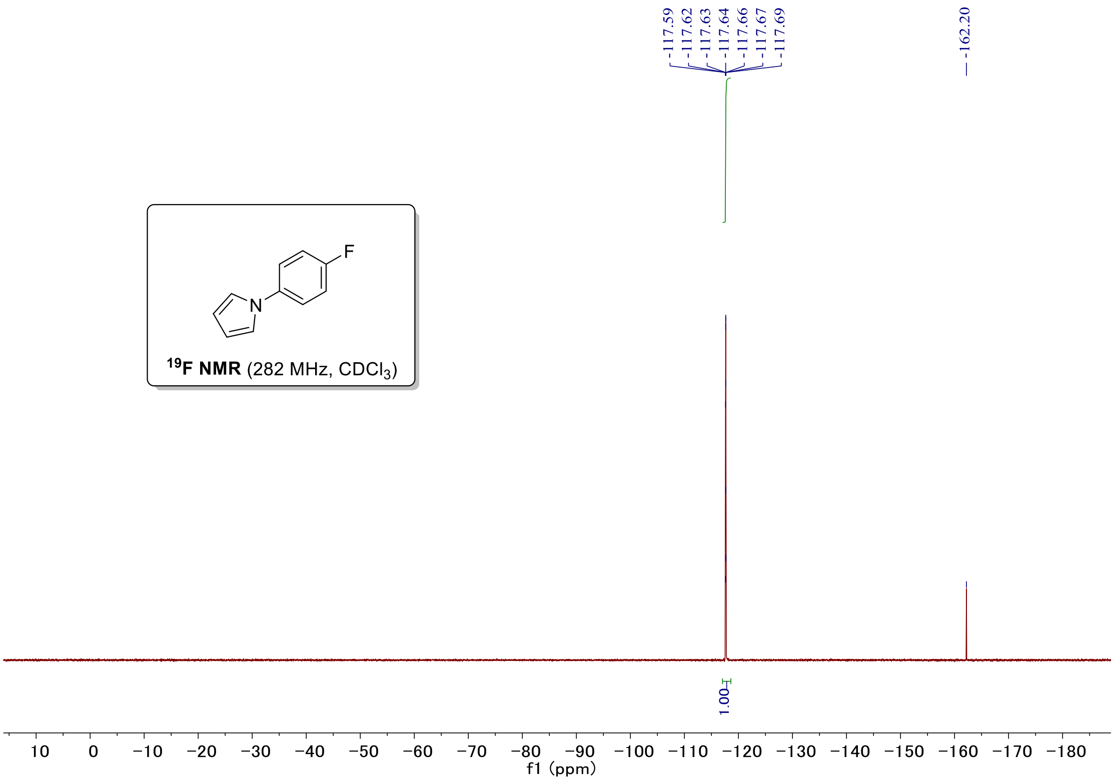

Supplementary Figure 49. ^{19}F NMR (282 MHz, $CDCl_3$, 25 °C) of compound **1z**

6-Fluoro-1-methyl-1*H*-indole (1aa)

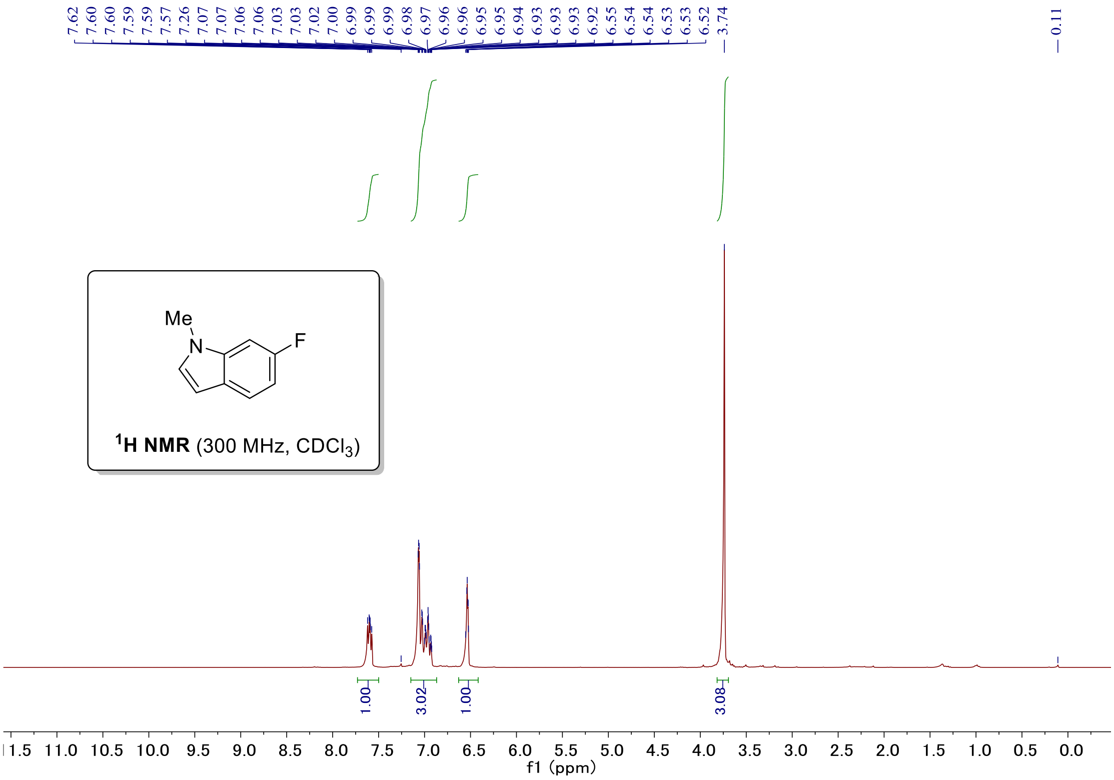

Supplementary Figure 50. ^{1}H NMR (300 MHz, $CDCl_3$, 25 ^{o}C) of compound **1aa**

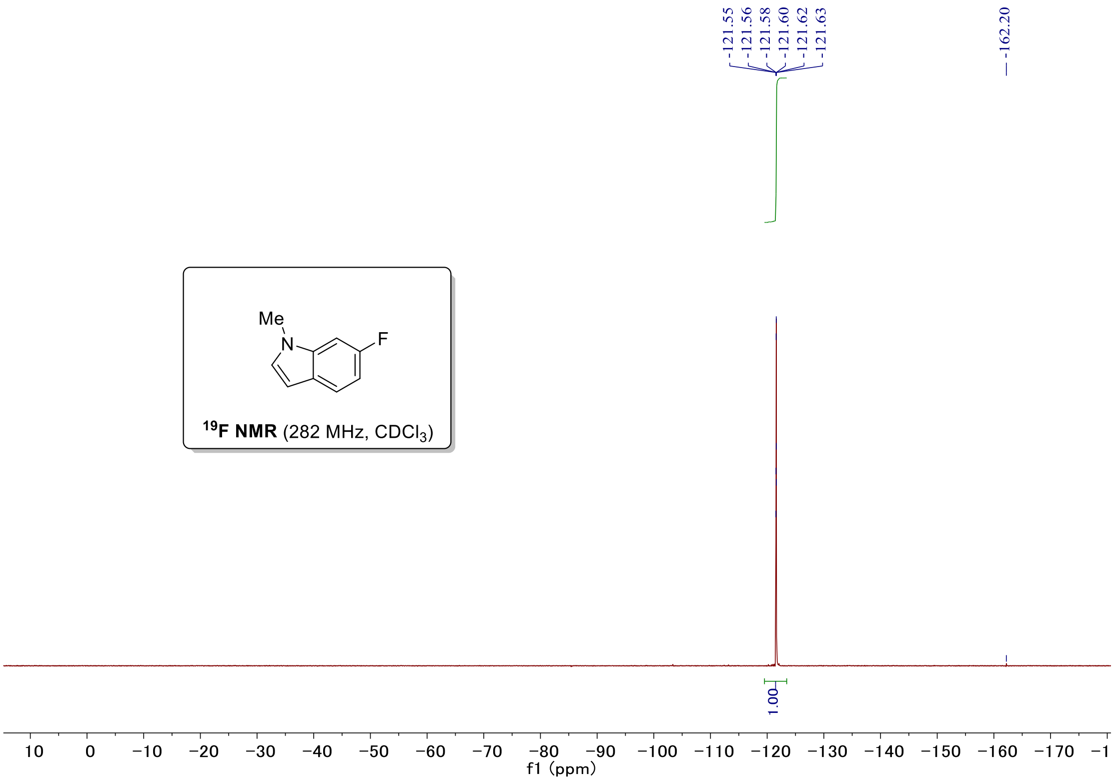

Supplementary Figure 51. ^{19}F NMR (282 MHz, $CDCl_3$, 25 ^{o}C) of compound **1aa**

2-(4-Fluorophenyl)-1-methyl-1*H*-indole (1ab)

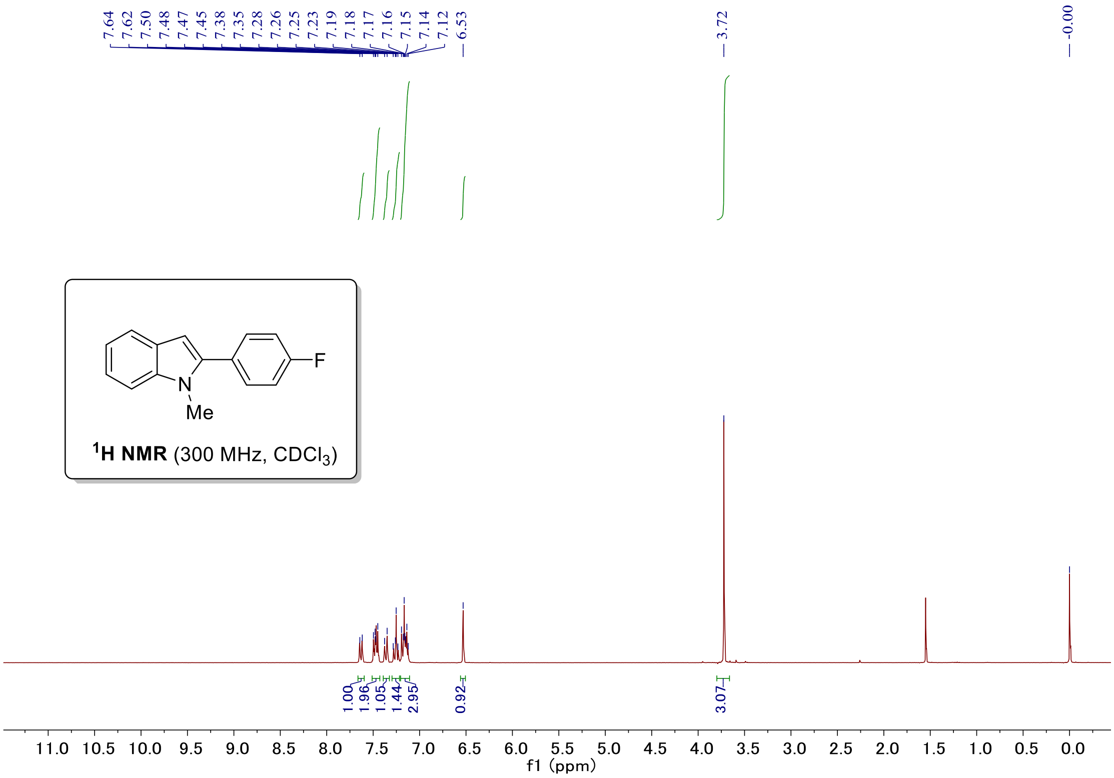

Supplementary Figure 52. ^{1}H NMR (300 MHz, $CDCl_3$, 25 °C) of compound **1ab**

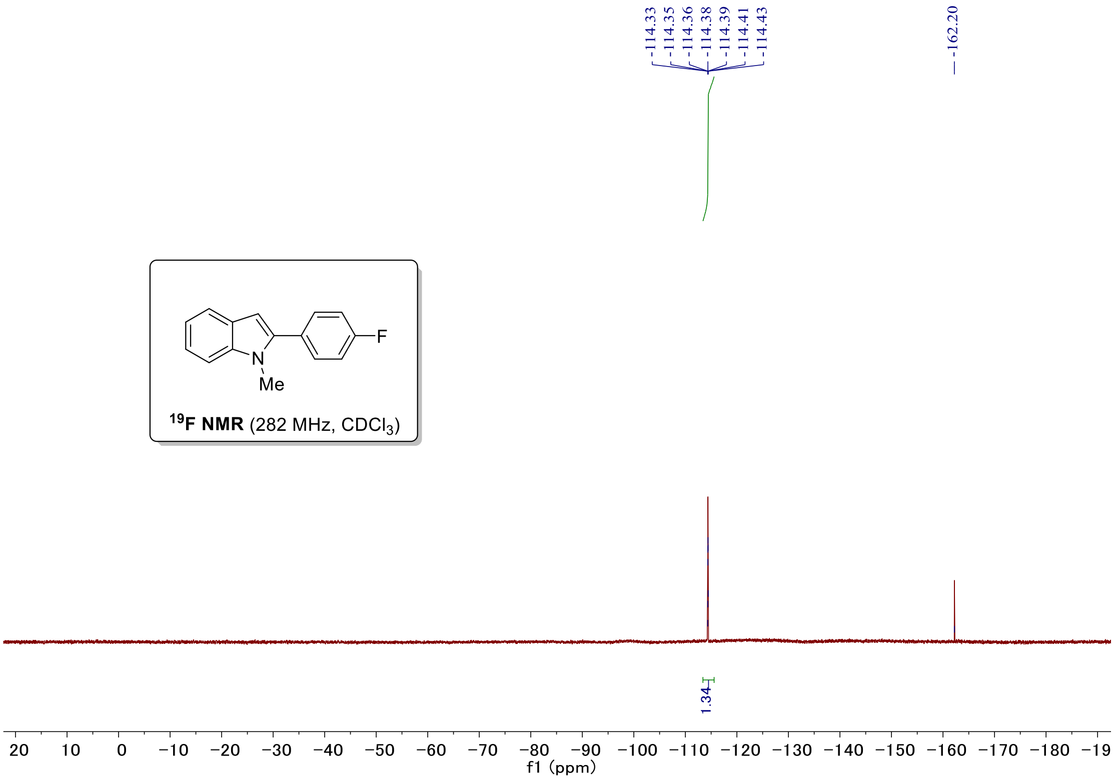

Supplementary Figure 53. ^{19}F NMR (282 MHz, $CDCl_3$, 25 °C) of compound **1ab**

2-(4-Fluorophenyl)benzofuran (1ac)

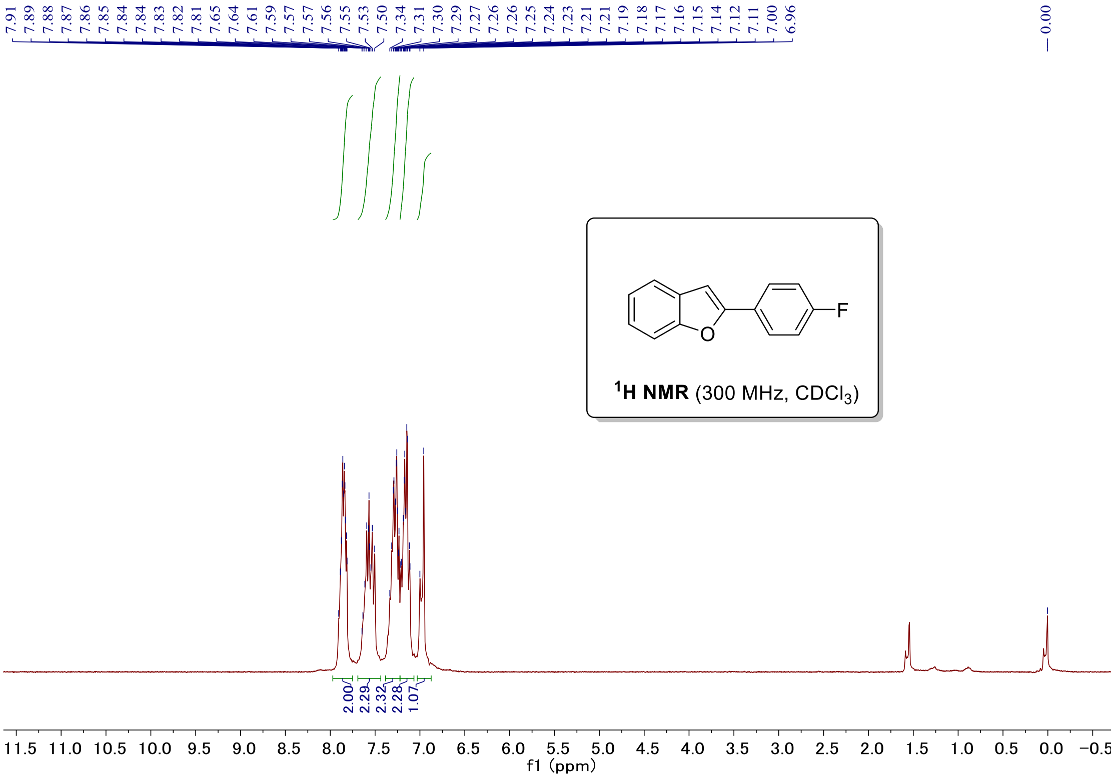

Supplementary Figure 54. ^{1}H NMR (300 MHz, $CDCl_3$, 25 °C) of compound **1ac**

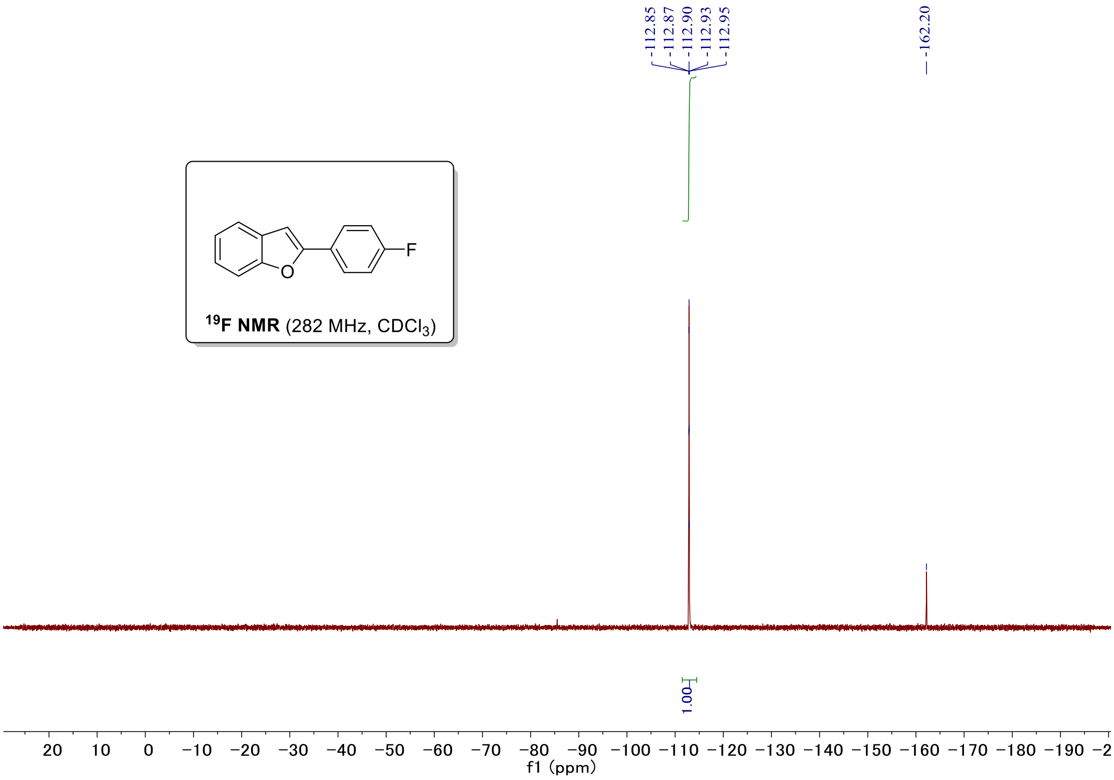

Supplementary Figure 55. ^{19}F NMR (282 MHz, $CDCl_3$, 25 °C) of compound **1ac**

(*R*)-6-((4-Fluorobenzyl)oxy)-2,5,7,8-tetramethyl-2-((4*R*,8*R*)-4,8,12-trimethyltridecyl)chromane (1ad)

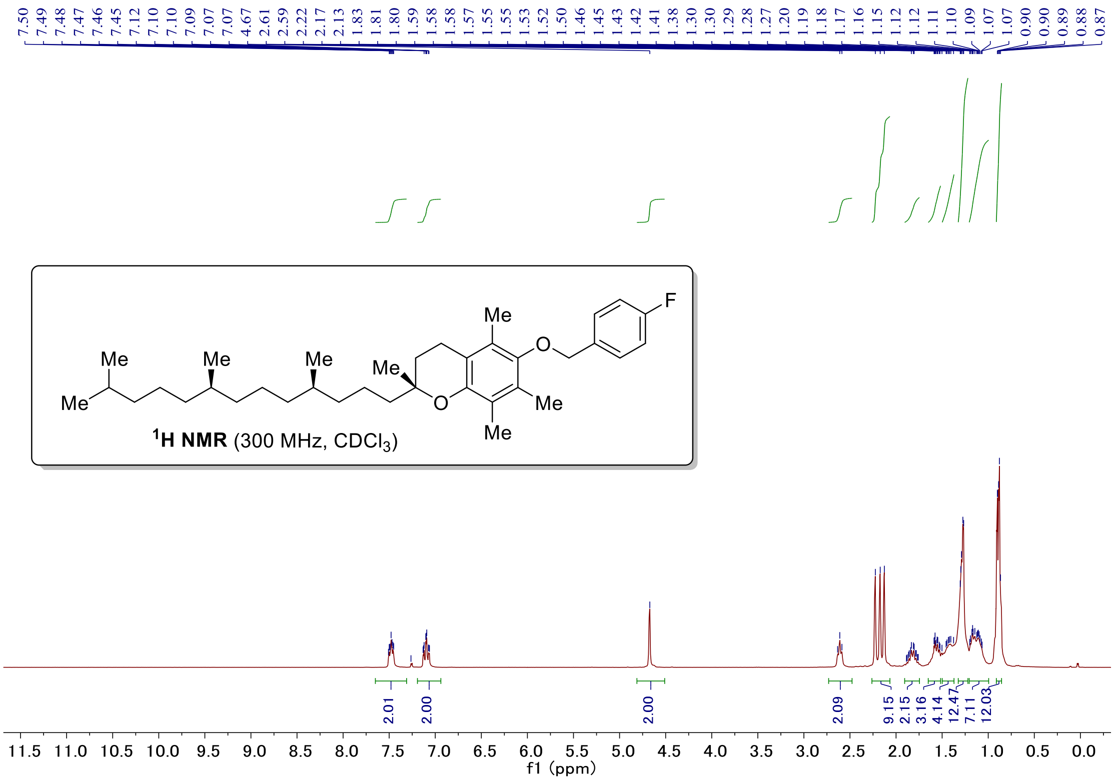

Supplementary Figure 56. ^{1}H NMR (300 MHz, $CDCl_3$, 25 °C) of compound **1ad**

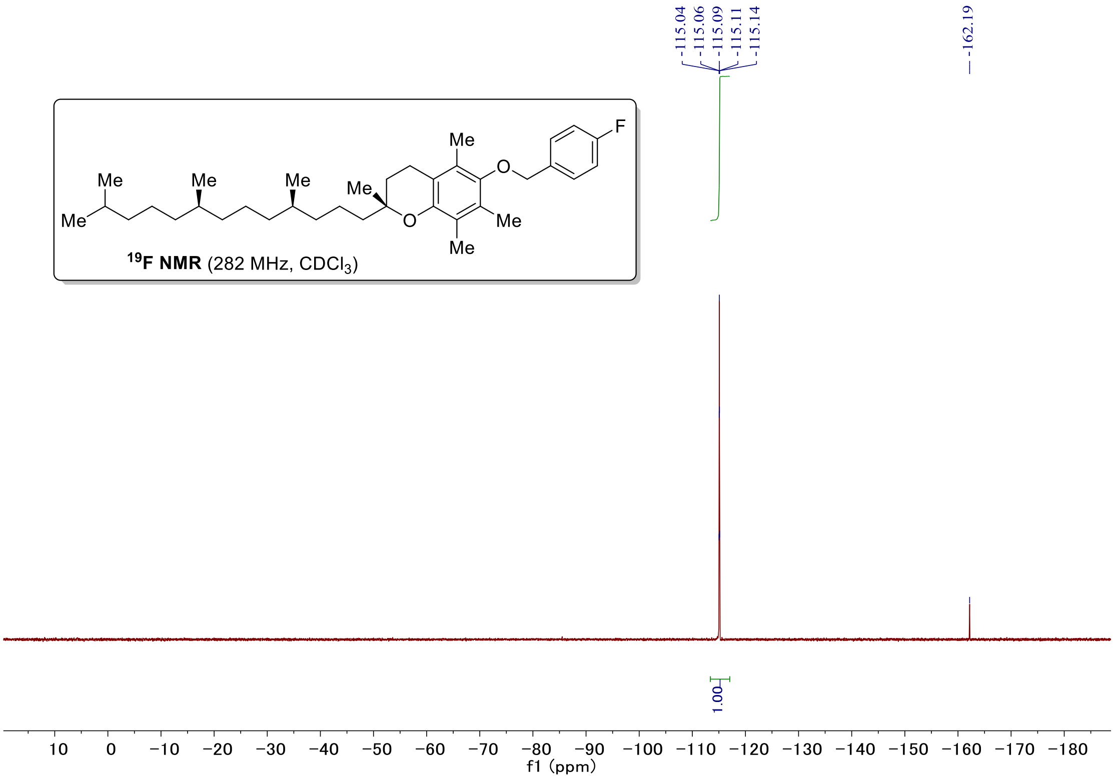

Supplementary Figure 57. ^{19}F NMR (282 MHz, $CDCl_3$, 25 °C) of compound **1ad**

(8*R*,9*S*,13*S*,14*S*)-3-Fluoro-17-methoxy-13-methyl-7,8,9,11,12,13,14,15,16,17-decahydro-6*H*-cyclopenta[*a*]phenanthrene (1ae)

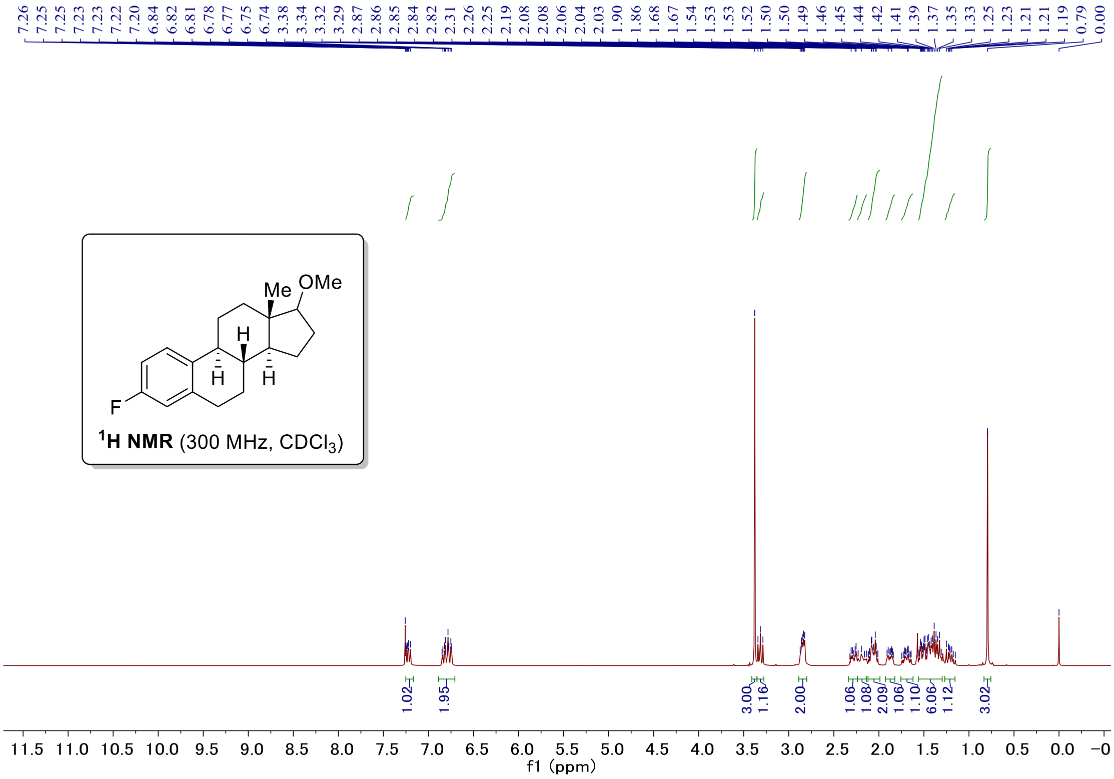

Supplementary Figure 58. ^{1}H NMR (300 MHz, $CDCl_3$, 25 °C) of compound **1ae**

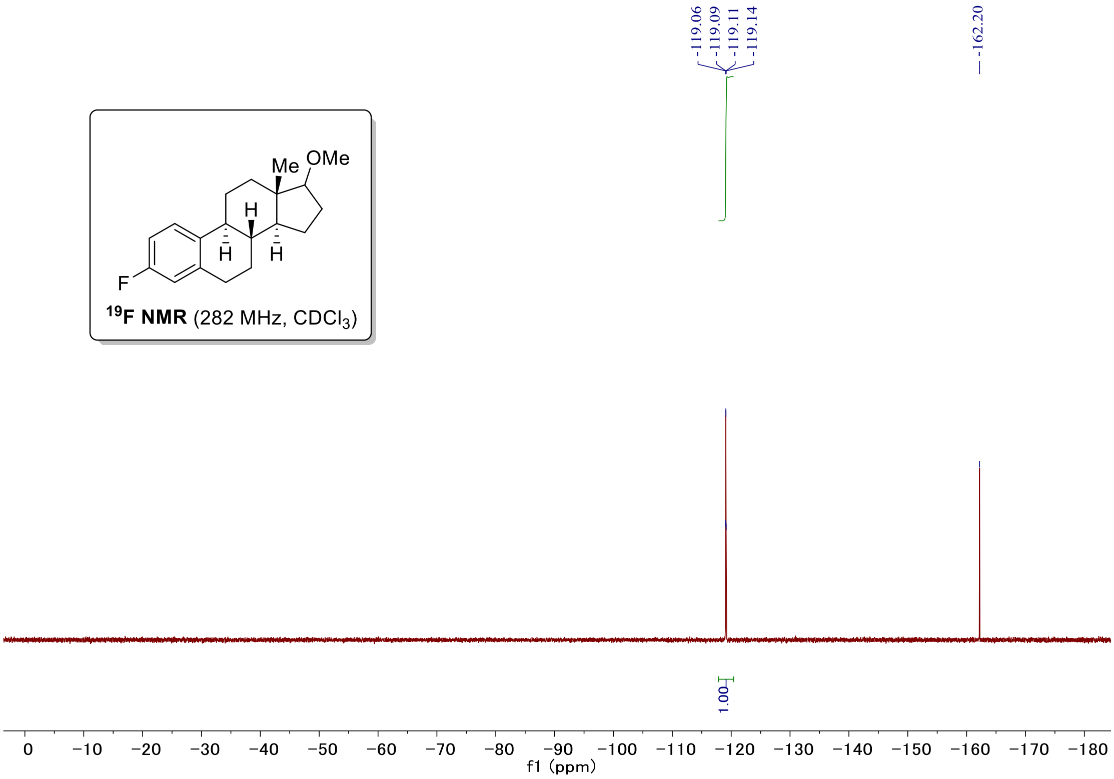

Supplementary Figure 59. ^{19}F NMR (282 MHz, $CDCl_3$, 25 °C) of compound **1ae**

1-Fluoro-4-((((2*S*,5*R*)-2-isopropyl-5-methylcyclohexyl)oxy)methyl)benzene (1af)

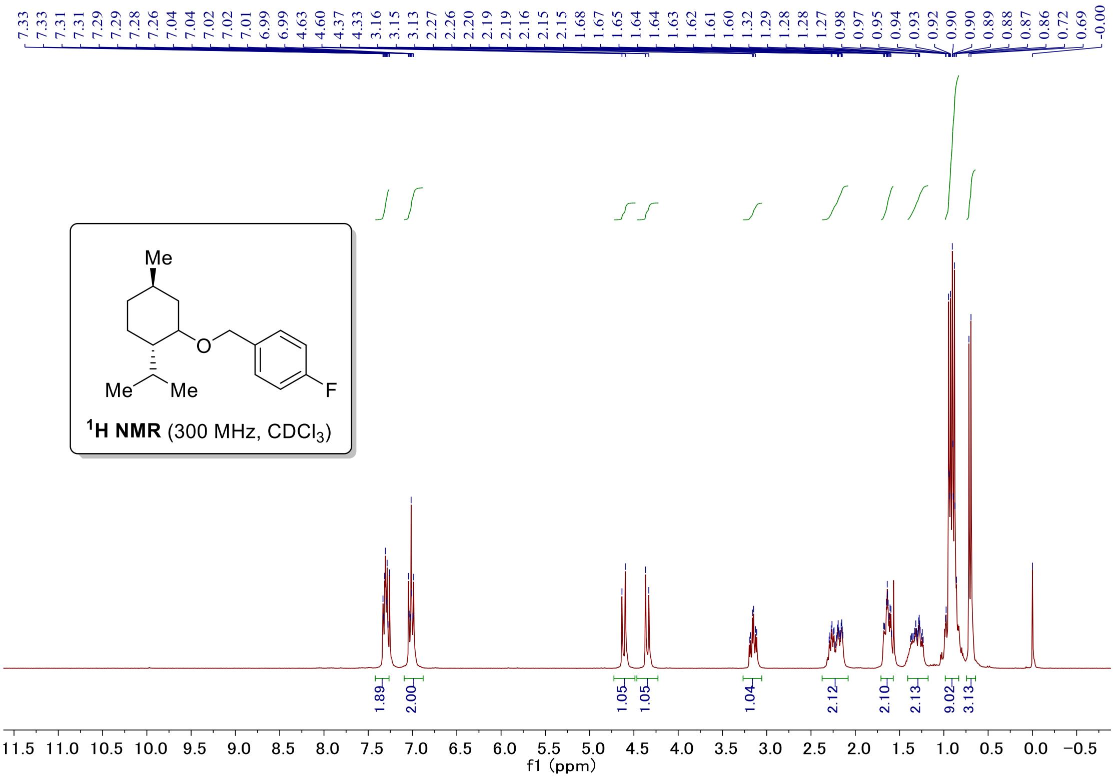

Supplementary Figure 60. ^{1}H NMR (300 MHz, $CDCl_3$, 25 °C) of compound **1af**

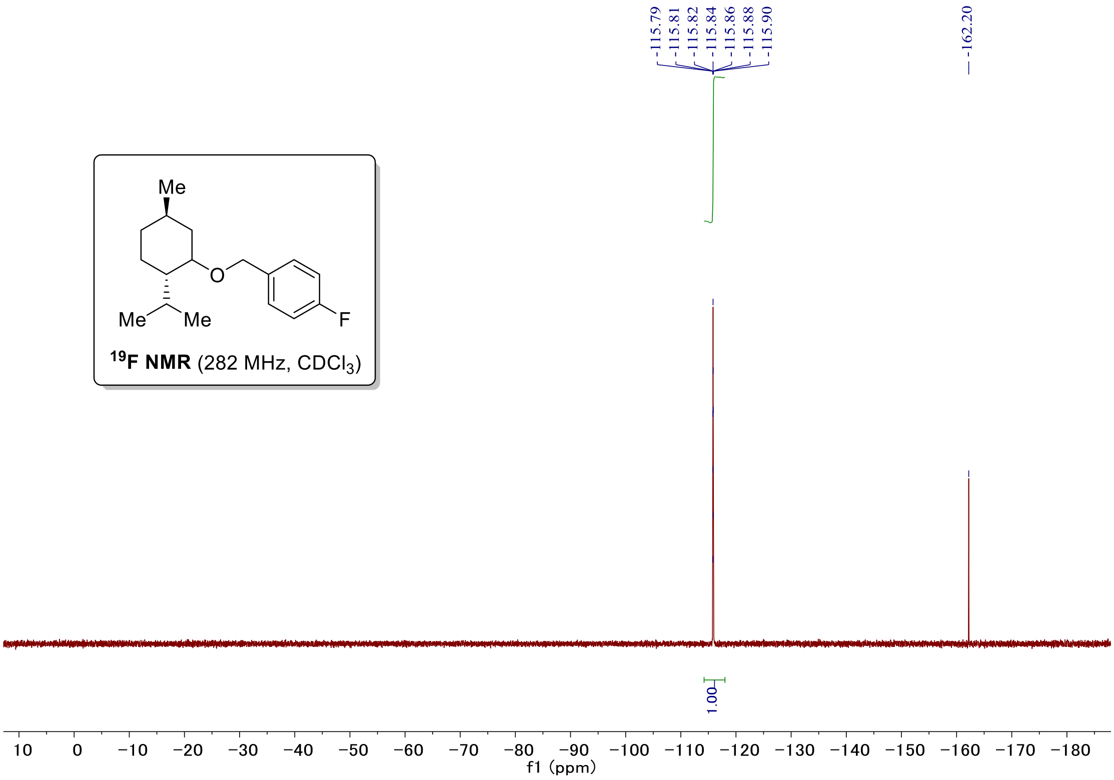

Supplementary Figure 61. ^{19}F NMR (282 MHz, $CDCl_3$, 25 °C) of compound **1af**

4-Fluorophenyl (*R*)-2-(6-methoxynaphthalen-2-yl)propanoate (1ag)

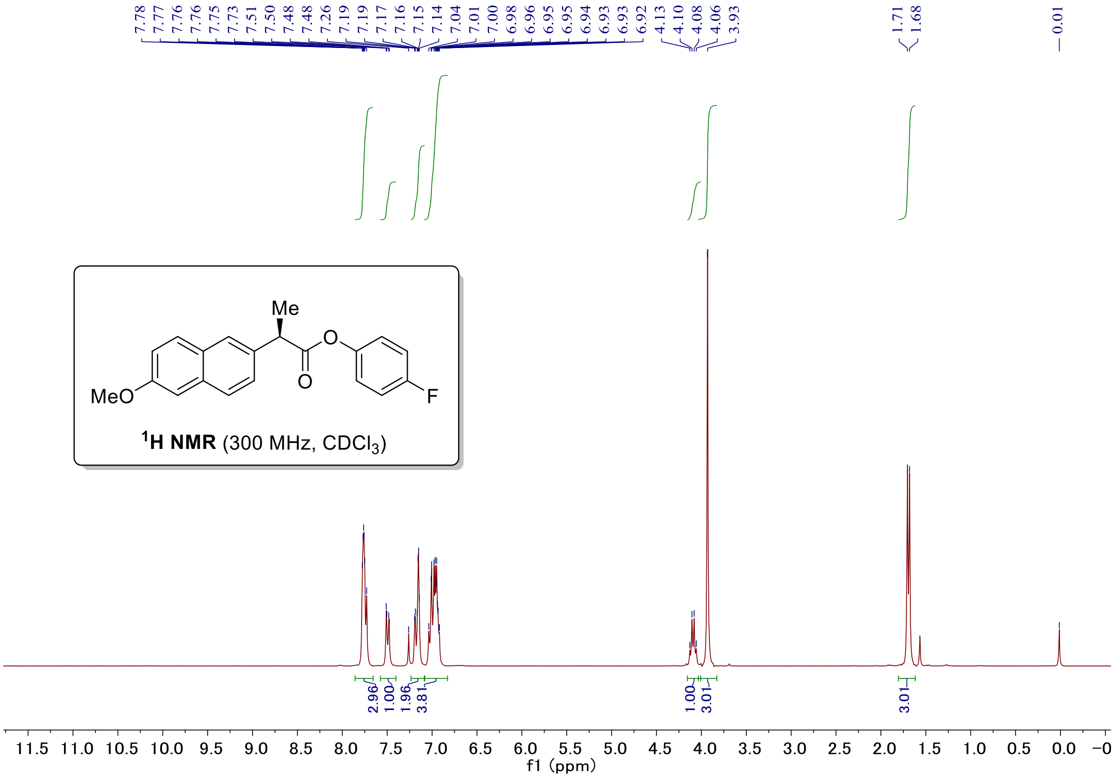

Supplementary Figure 62. ^{1}H NMR (300 MHz, $CDCl_3$, 25 °C) of compound **1ag**

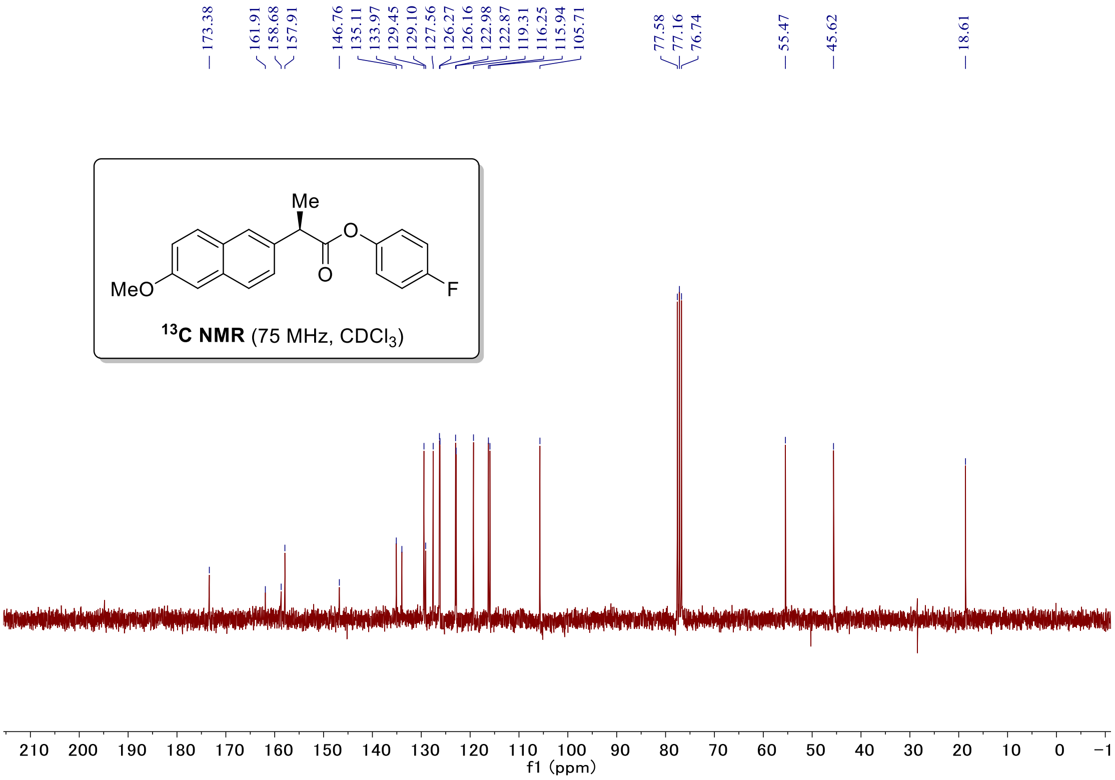

Supplementary Figure 63. ^{13}C NMR (75 MHz, $CDCl_3$, 25 °C) of compound **1ag**

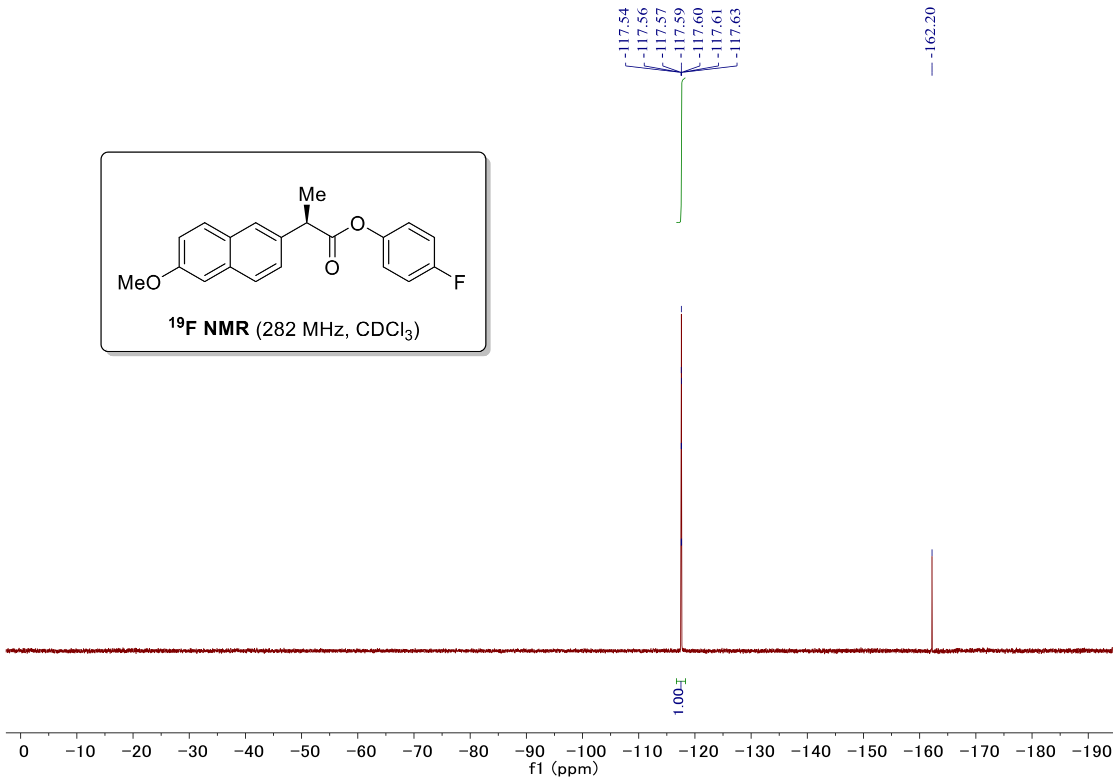

Supplementary Figure 64. ^{19}F NMR (282 MHz, $CDCl_3$, 25 °C) of compound **1ag**

4-(Fluoromethyl)biphenyl (2a)

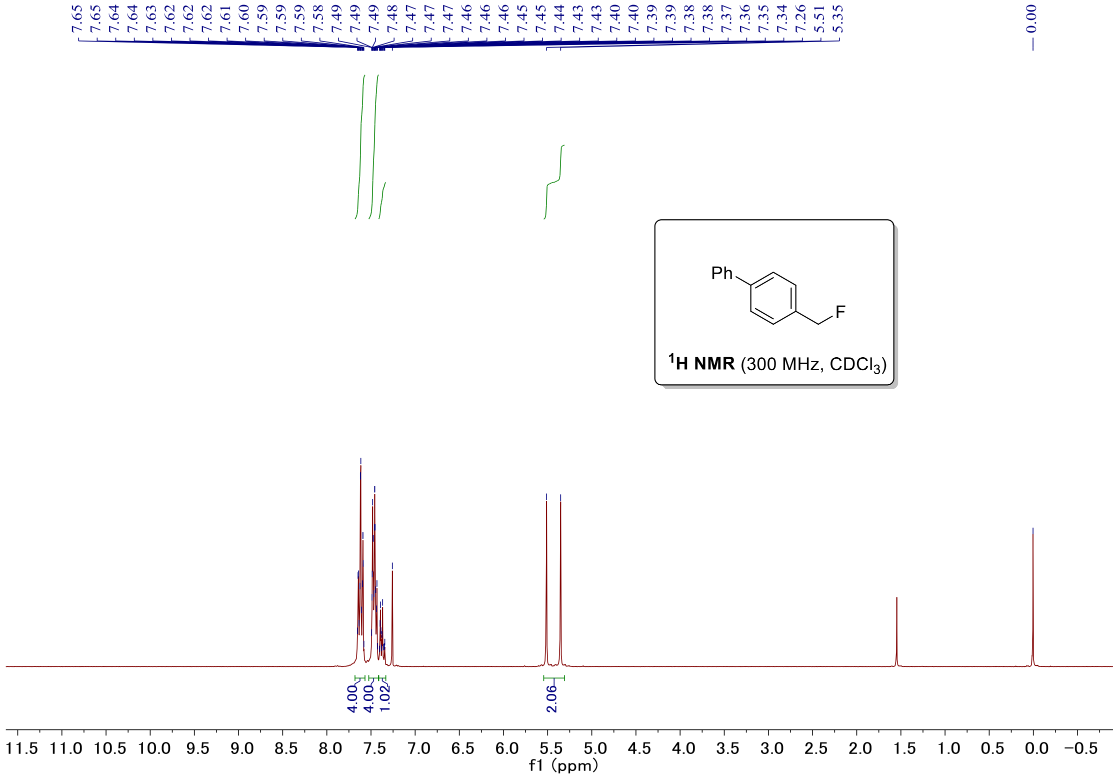

Supplementary Figure 65. ^{1}H NMR (300 MHz, $CDCl_3$, 25 °C) of compound **2a**

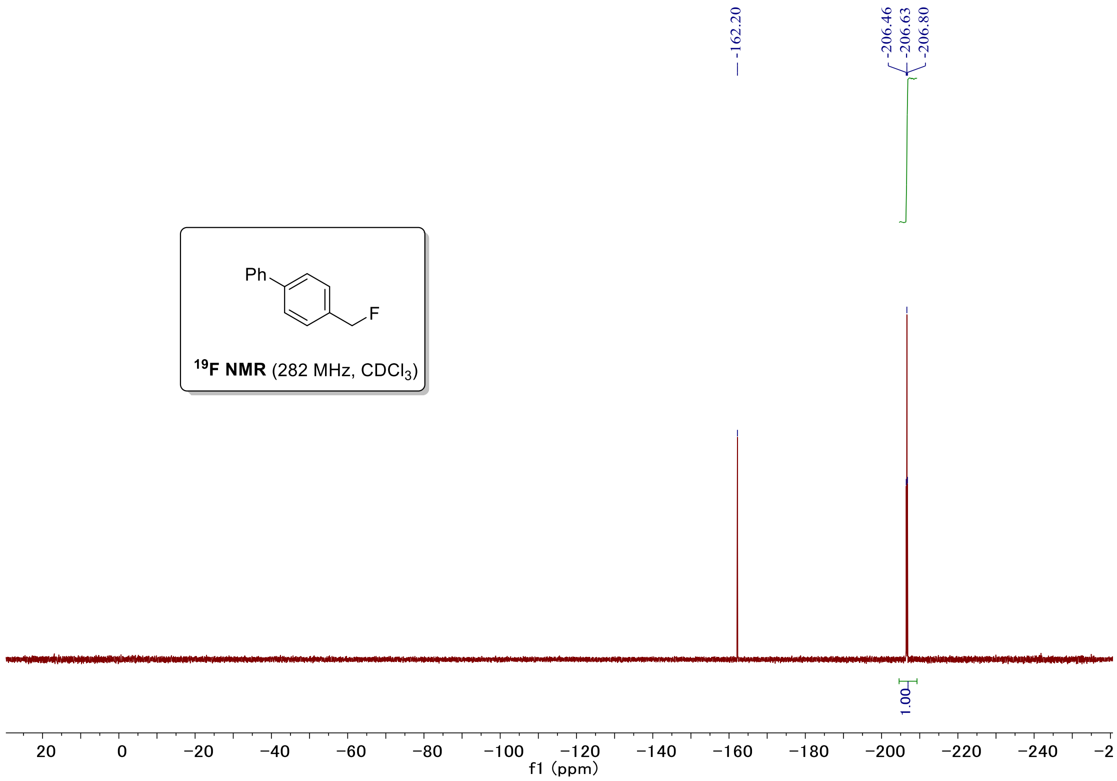

Supplementary Figure 66. ^{19}F NMR (282 MHz, $CDCl_3$, 25 °C) of compound **2a**

1-(Fluoromethyl)-4-(trifluoromethoxy)benzene (2b)

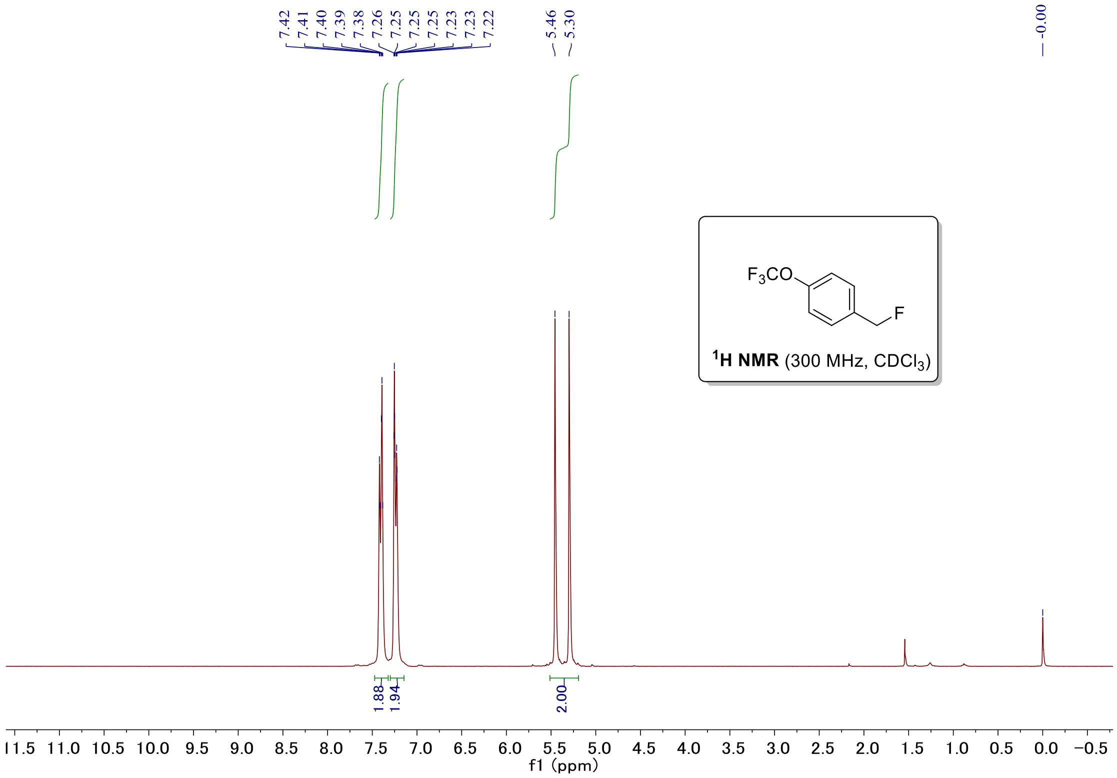

Supplementary Figure 67. ^{1}H NMR (300 MHz, $CDCl_3$, 25 °C) of compound **2b**

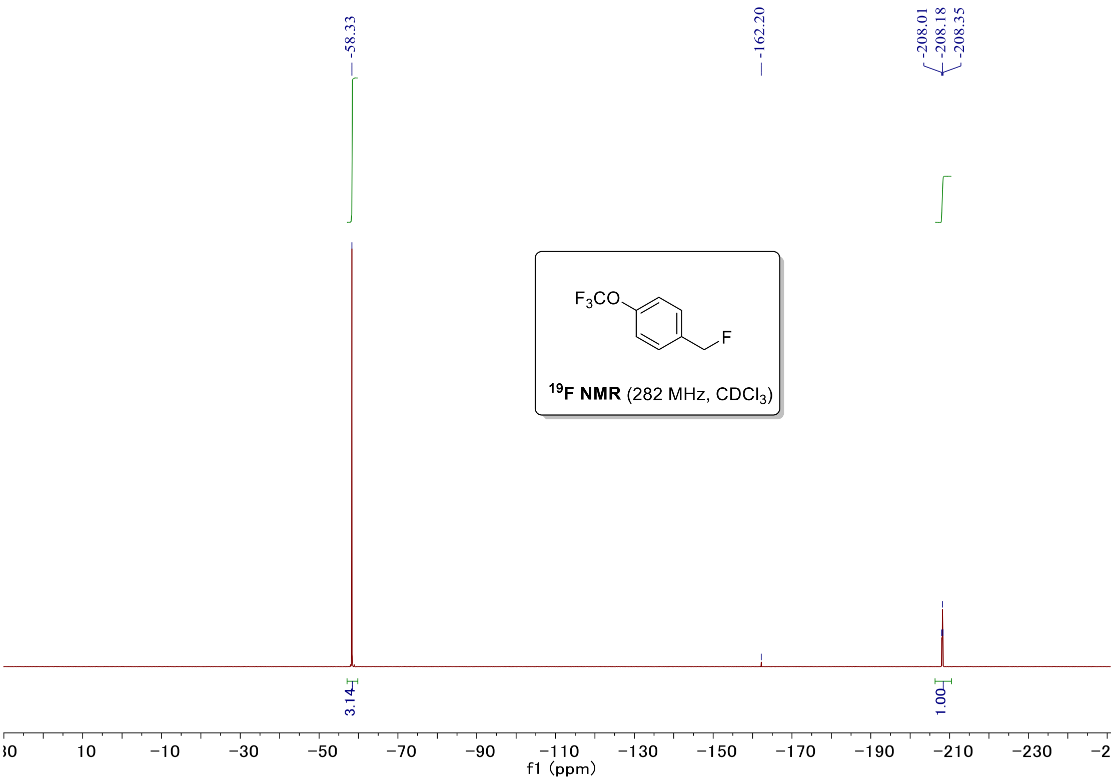

Supplementary Figure 68. ^{19}F NMR (282 MHz, $CDCl_3$, 25 °C) of compound **2b**

1-(Fluoromethyl)naphthalene (2c)

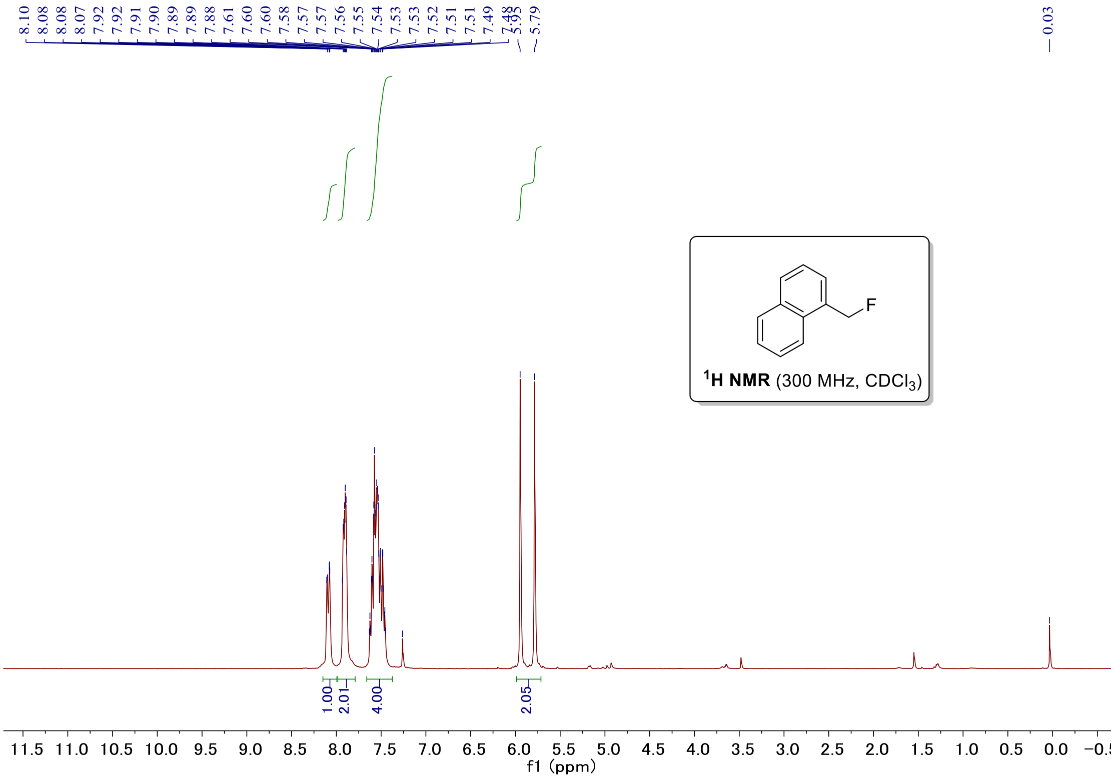

Supplementary Figure 69. ^{1}H NMR (300 MHz, $CDCl_3$, 25 °C) of compound **2c**

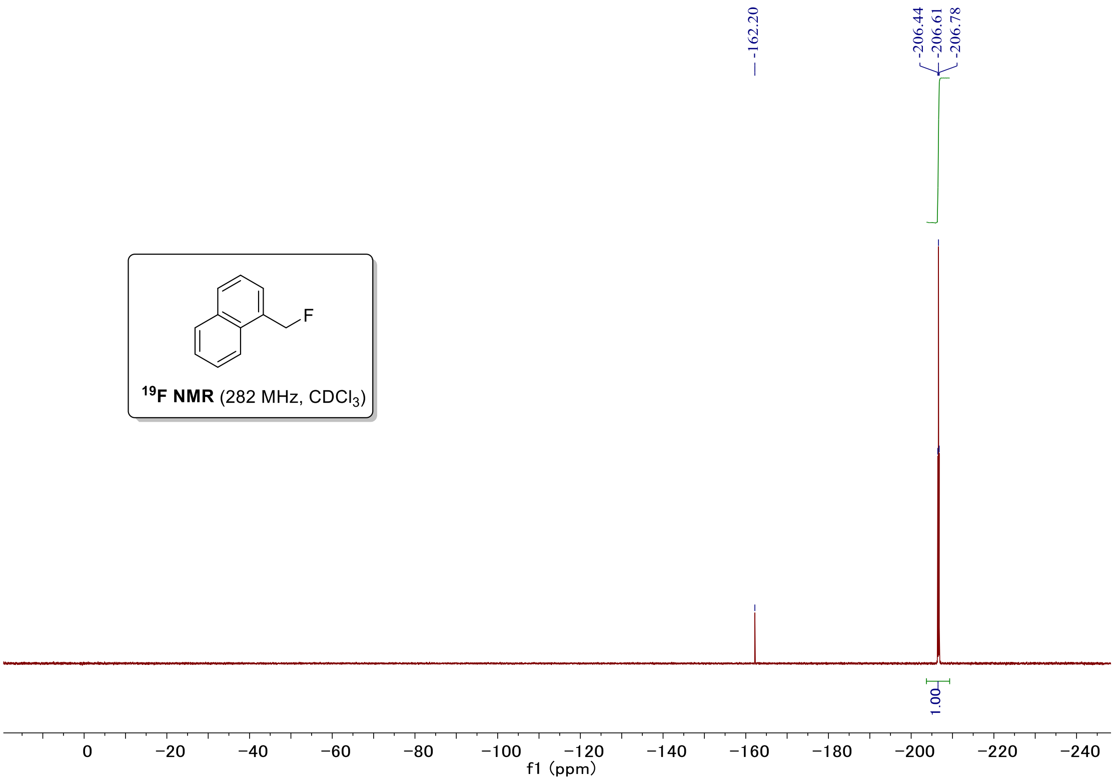

Supplementary Figure 70. ^{19}F NMR (282 MHz, $CDCl_3$, 25 °C) of compound **2c**

1-Fluorodecane (2e)

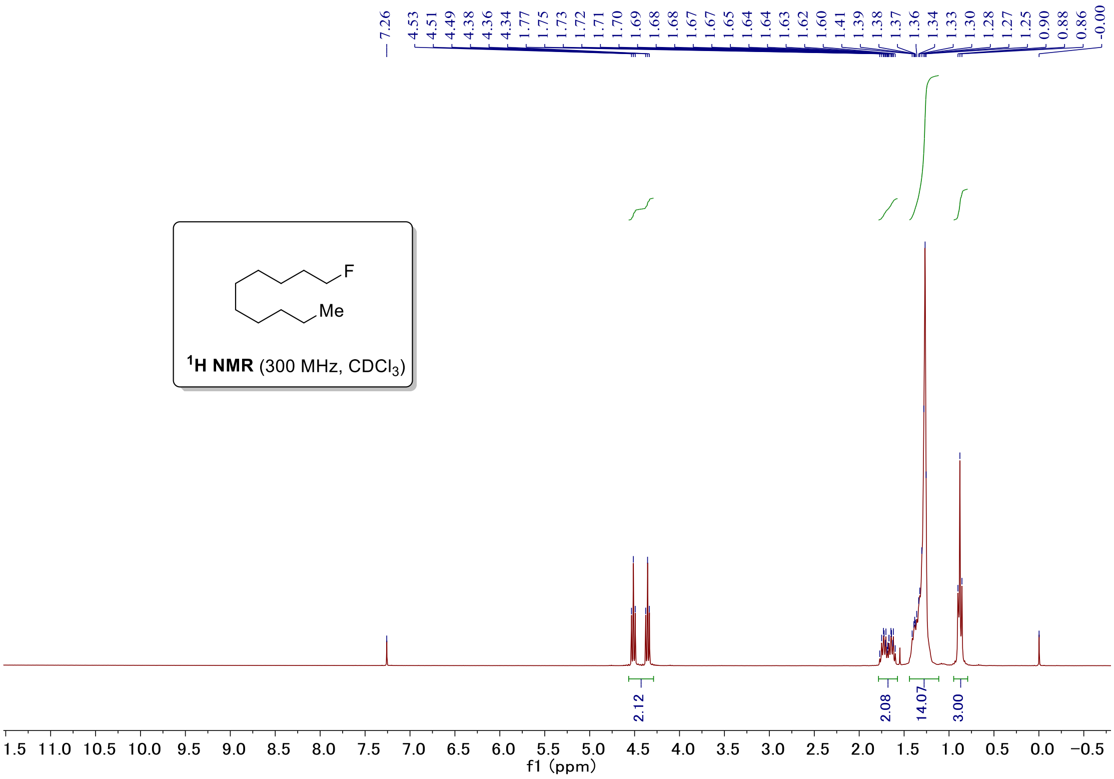

Supplementary Figure 71. ^{1}H NMR (300 MHz, $CDCl_3$, 25 °C) of compound **2e**

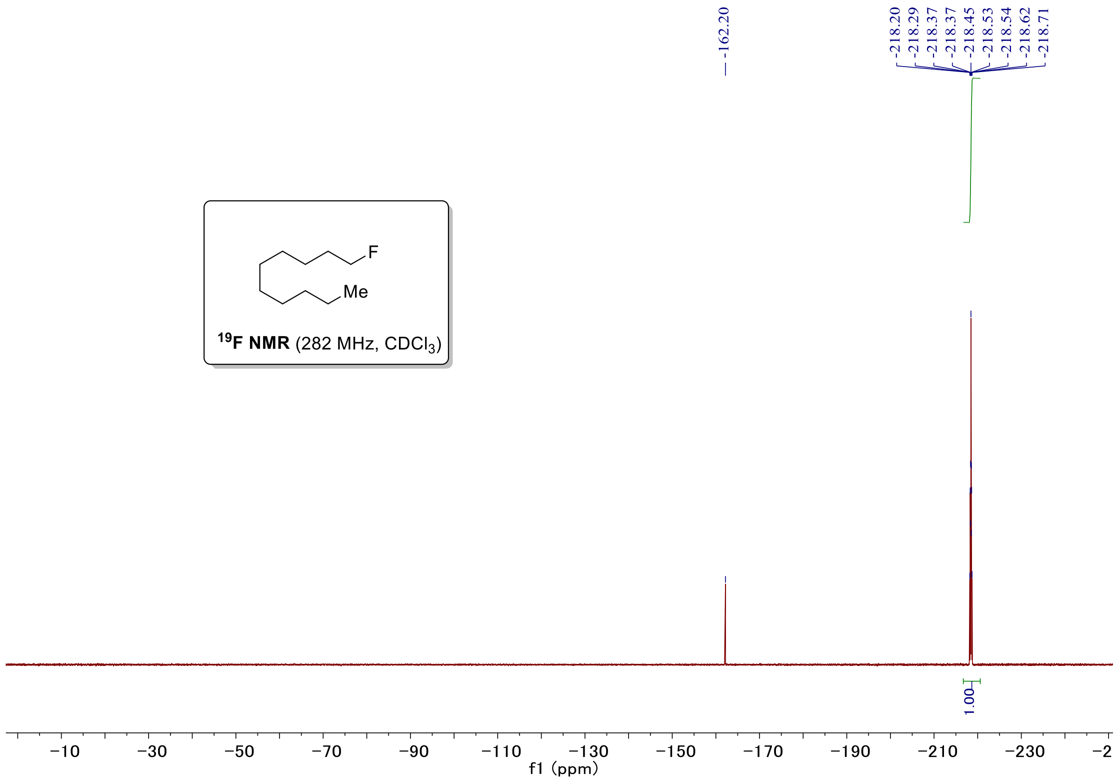

Supplementary Figure 72. ^{19}F NMR (282 MHz, $CDCl_3$, 25 °C) of compound **2e**

2-(1-Fluoroethyl)naphthalene (2d)

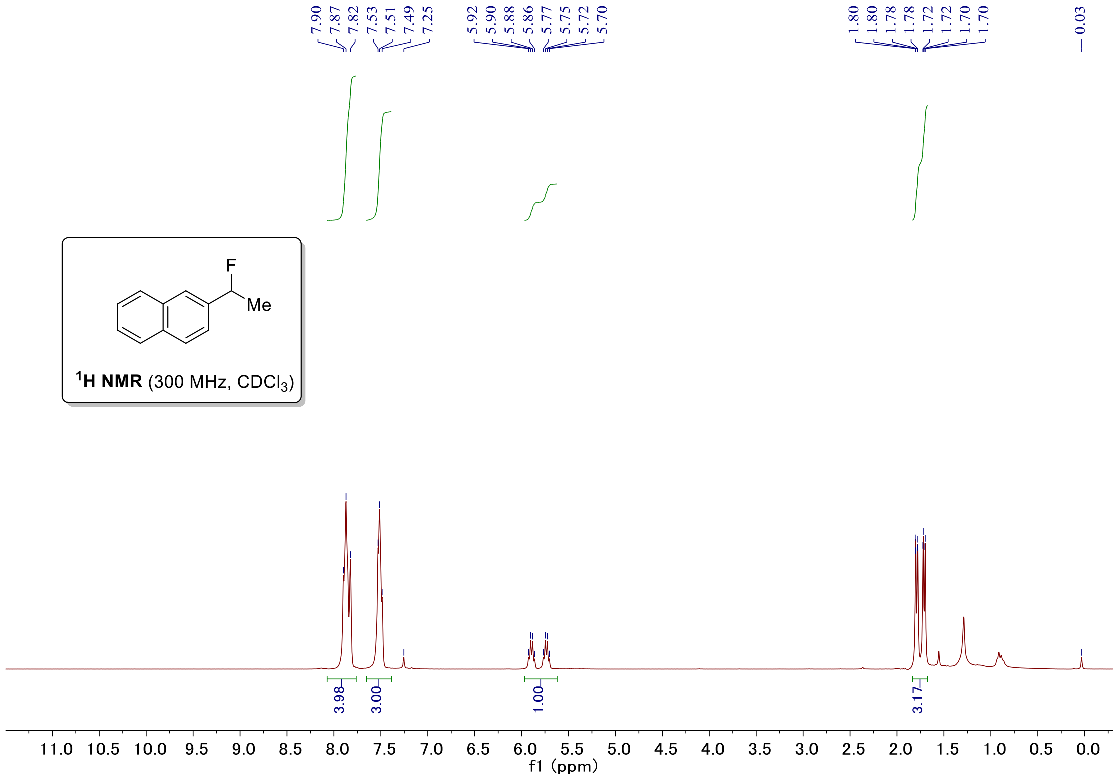

Supplementary Figure 73. ^{1}H NMR (300 MHz, $CDCl_3$, 25 °C) of compound **2d**

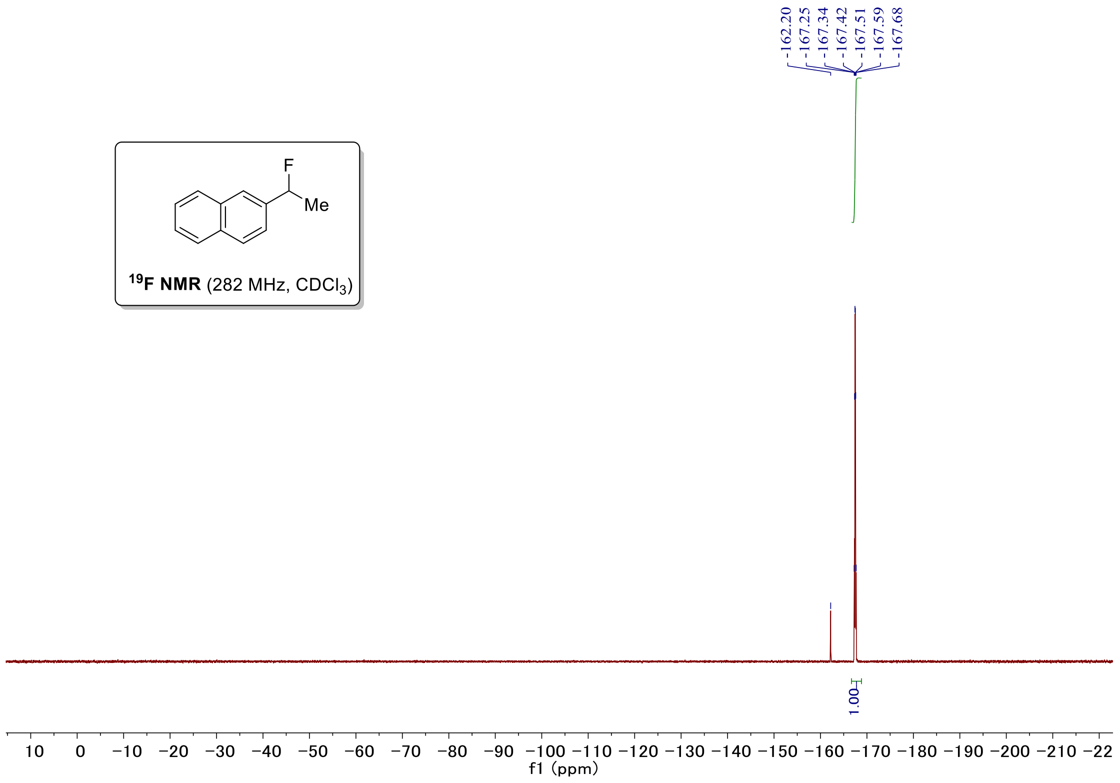

Supplementary Figure 74. ^{19}F NMR (282 MHz, $CDCl_3$, 25 °C) of compound **2d**

(*E*)-(3-Fluoroprop-1-en-1-yl)benzene

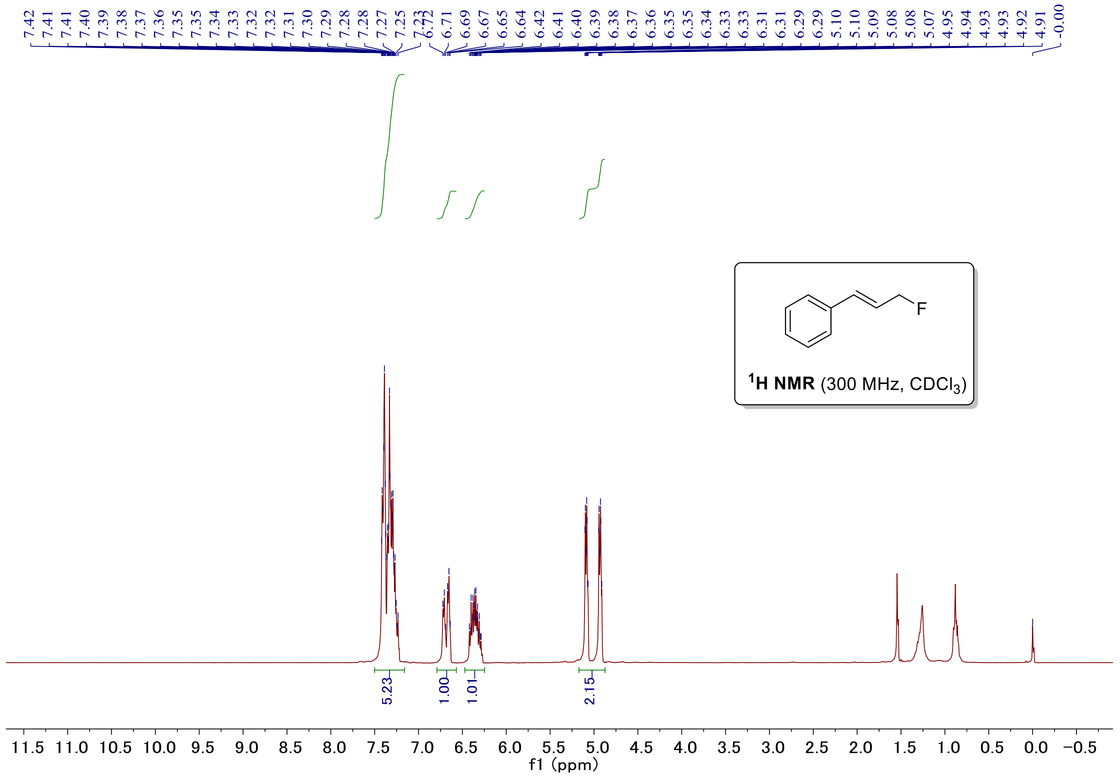

Supplementary Figure 75. ^{1}H NMR (300 MHz, $CDCl_3$, 25 °C) of compound **(*E*)-(3-Fluoroprop-1-en-1-yl)benzene**

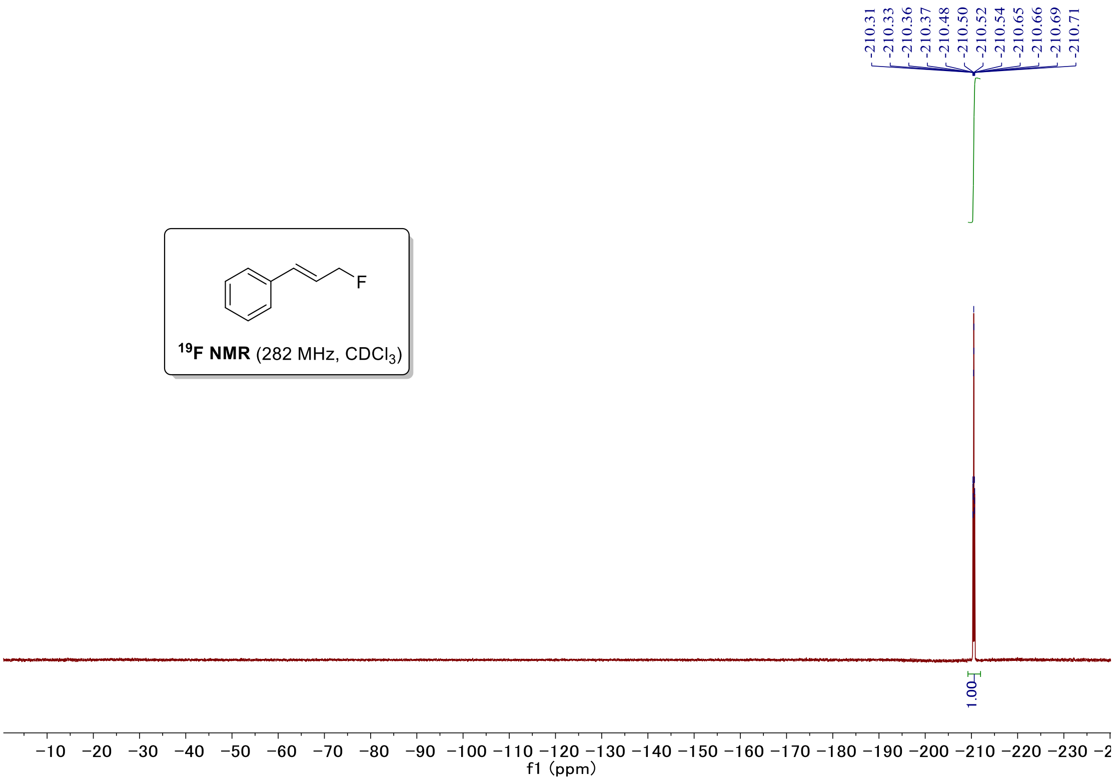

Supplementary Figure 76. ^{19}F NMR (282 MHz, $CDCl_3$, 25 ºC) of compound **(*E*)-(3-Fluoroprop-1-en-1-yl)benzene**

(*E*)-1-Fluorooct-2-ene

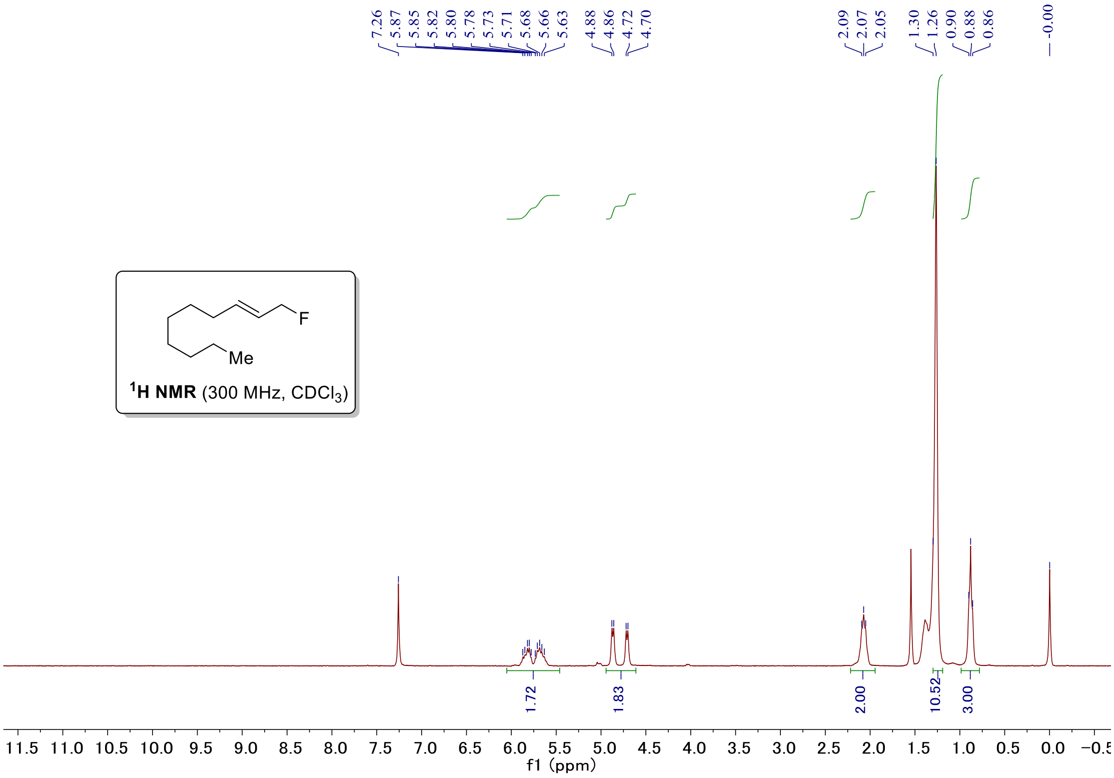

Supplementary Figure 77. ^{1}H NMR (300 MHz, $CDCl_3$, 25 ºC) of compound **(*E*)-1-Fluorooct-2-ene**

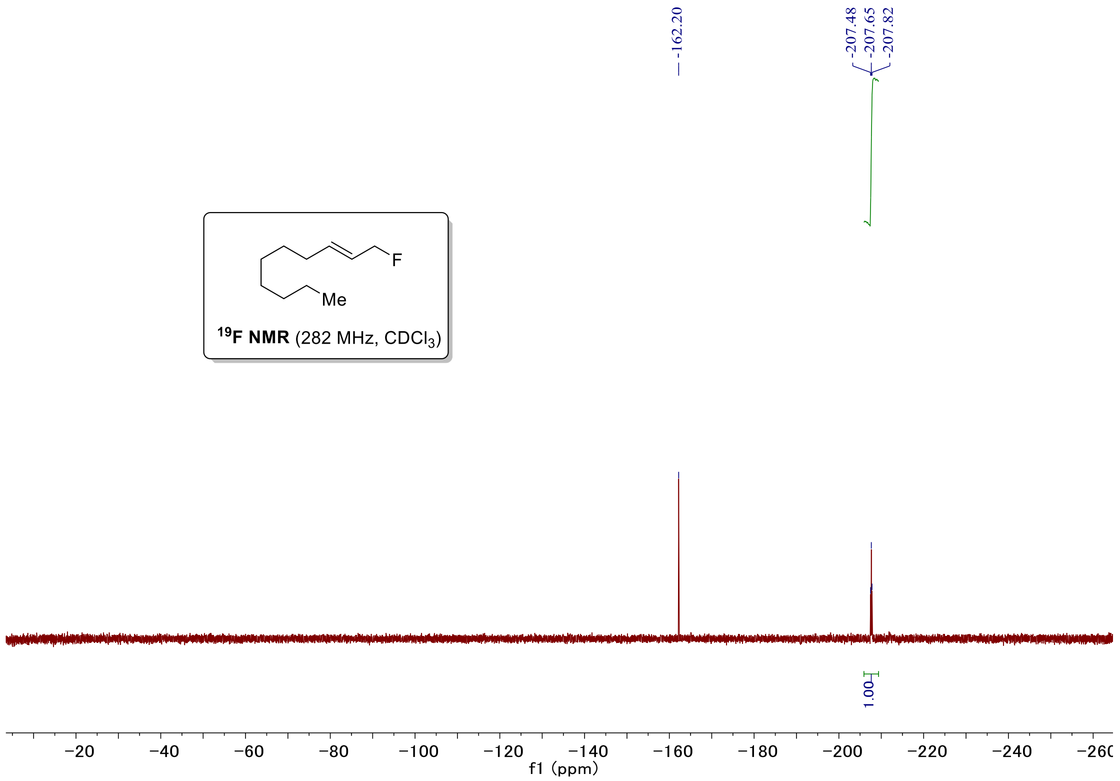

Supplementary Figure 78. ^{19}F NMR (282 MHz, $CDCl_3$, 25 ºC) of compound **(*E*)-1-Fluorooct-2-ene**

***N*,4-Dimethylaniline (3b)**

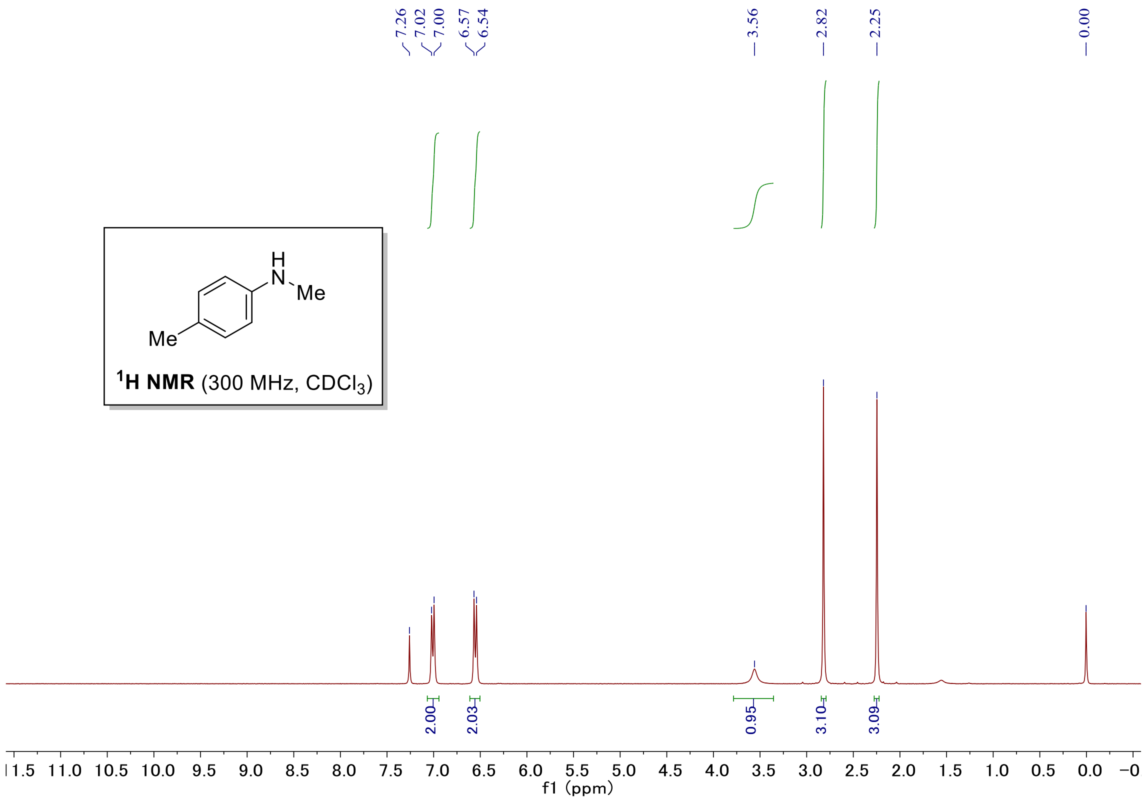

Supplementary Figure 79. ^{1}H NMR (300 MHz, $CDCl_3$, 25 ºC) of compound **3b**

N,3,5-Trimethylaniline (3c)

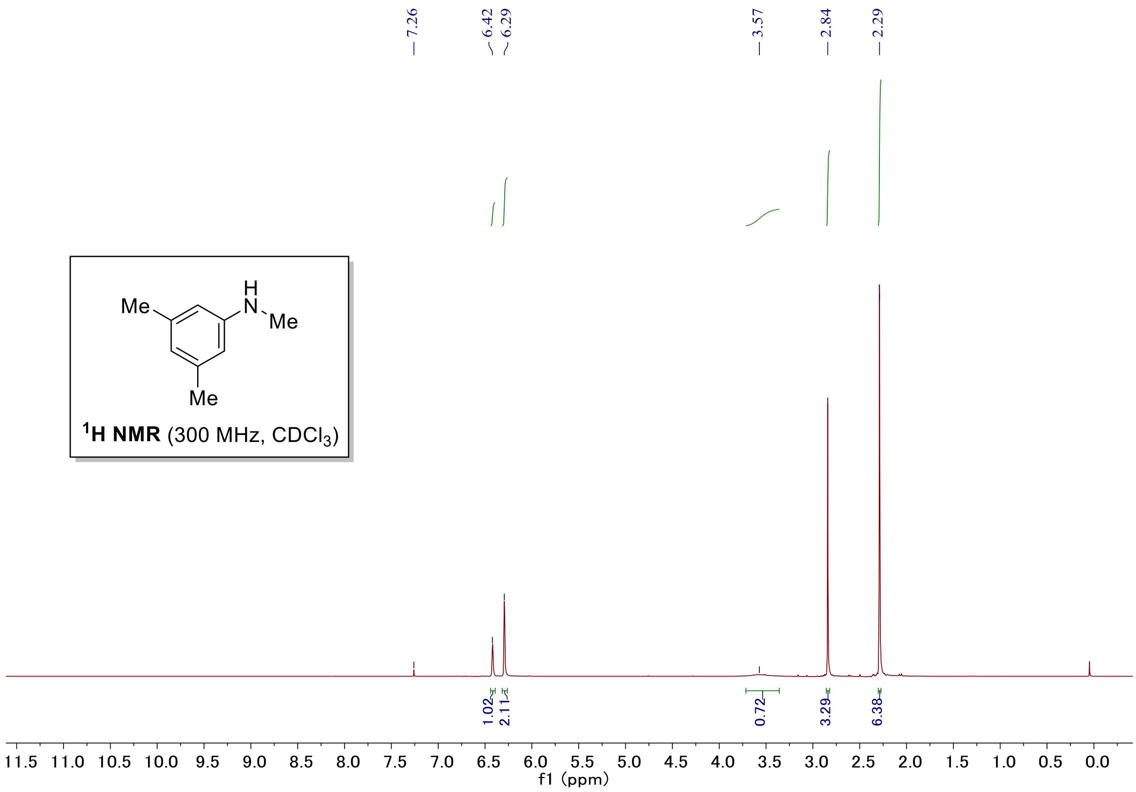

Supplementary Figure 80. ^{1}H NMR (300 MHz, $CDCl_3$, 25 °C) of compound **3c**

N,2-Dimethylaniline (3d)

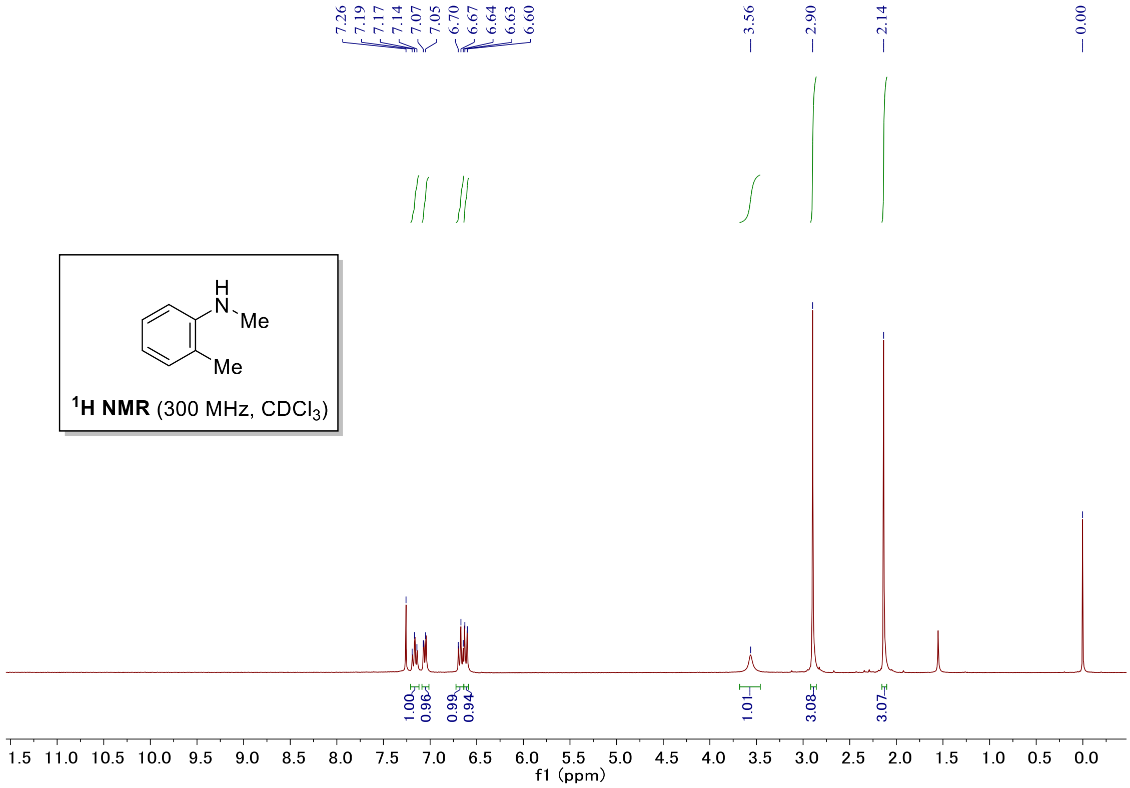

Supplementary Figure 81. ^{1}H NMR (300 MHz, $CDCl_3$, 25 °C) of compound **3d**

***N*,2,4,6-Tetramethylaniline (3e)**

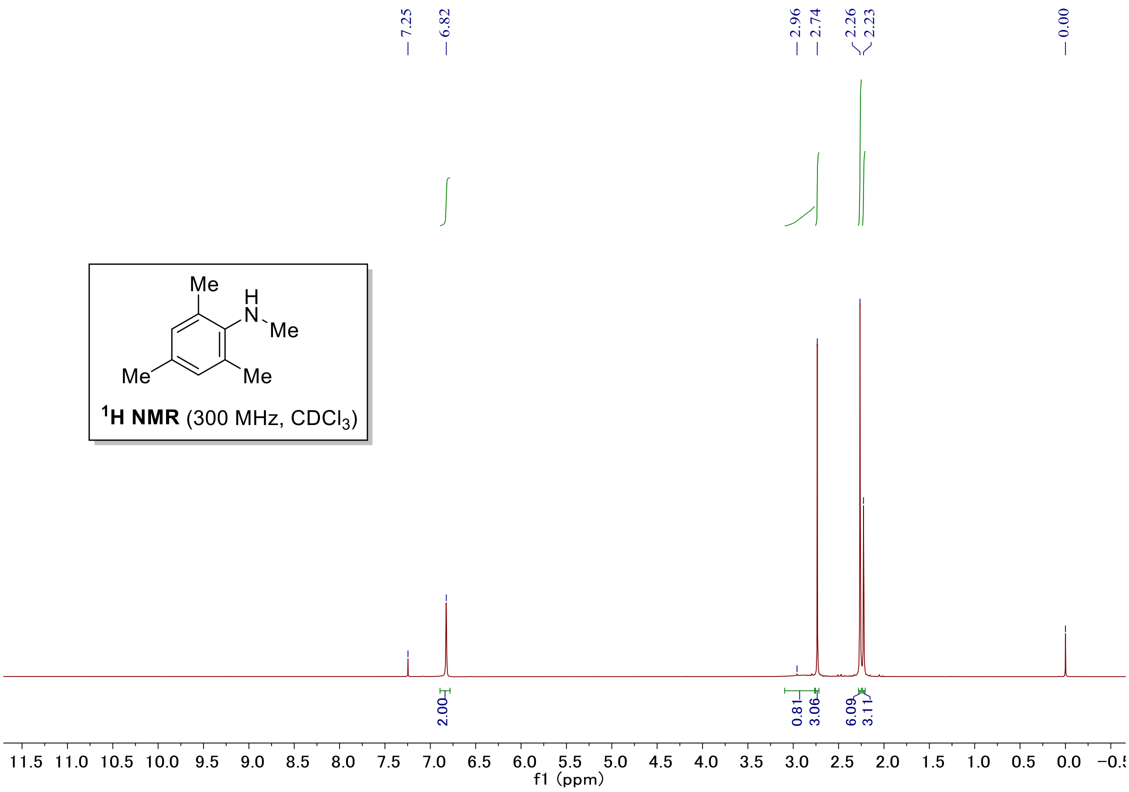

Supplementary Figure 82. ^{1}H NMR (300 MHz, $CDCl_3$, 25 °C) of compound **3e**

4-Methoxy-*N*-methylaniline (3f)

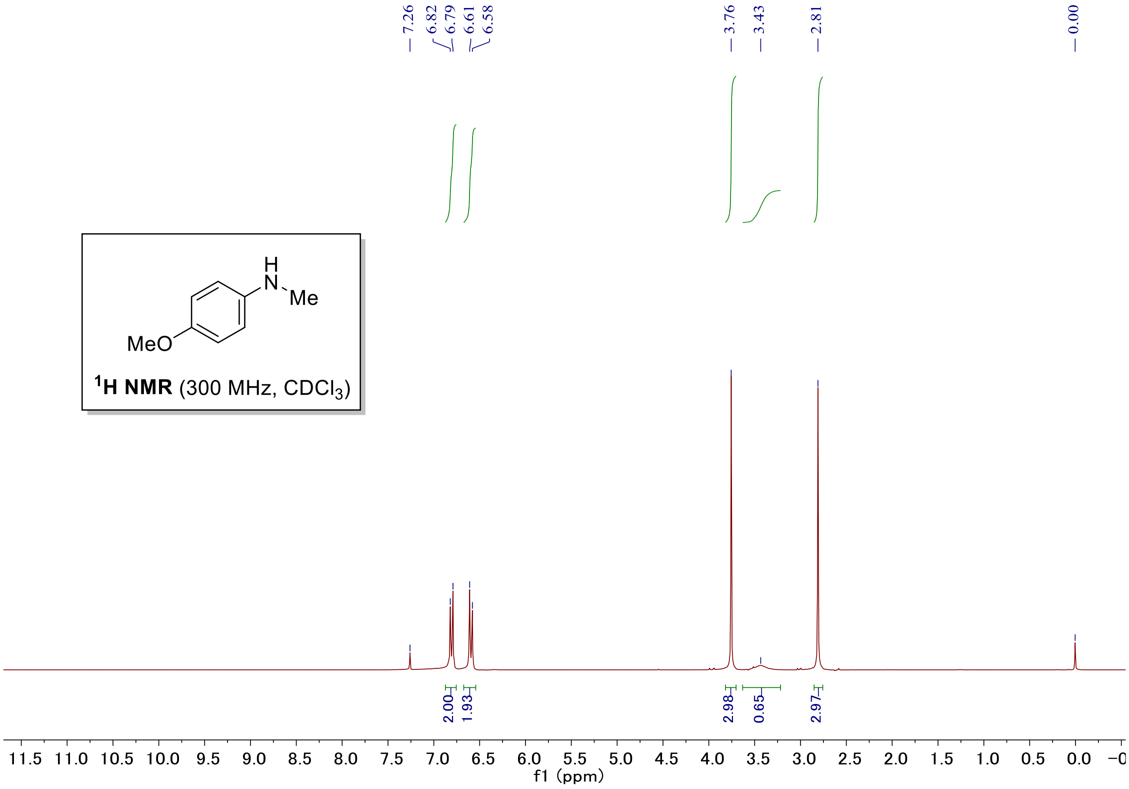

Supplementary Figure 83. ^{1}H NMR (300 MHz, $CDCl_3$, 25 °C) of compound **3f**

N-Methylbenzo[d][1,3]dioxol-5-amine (3g)

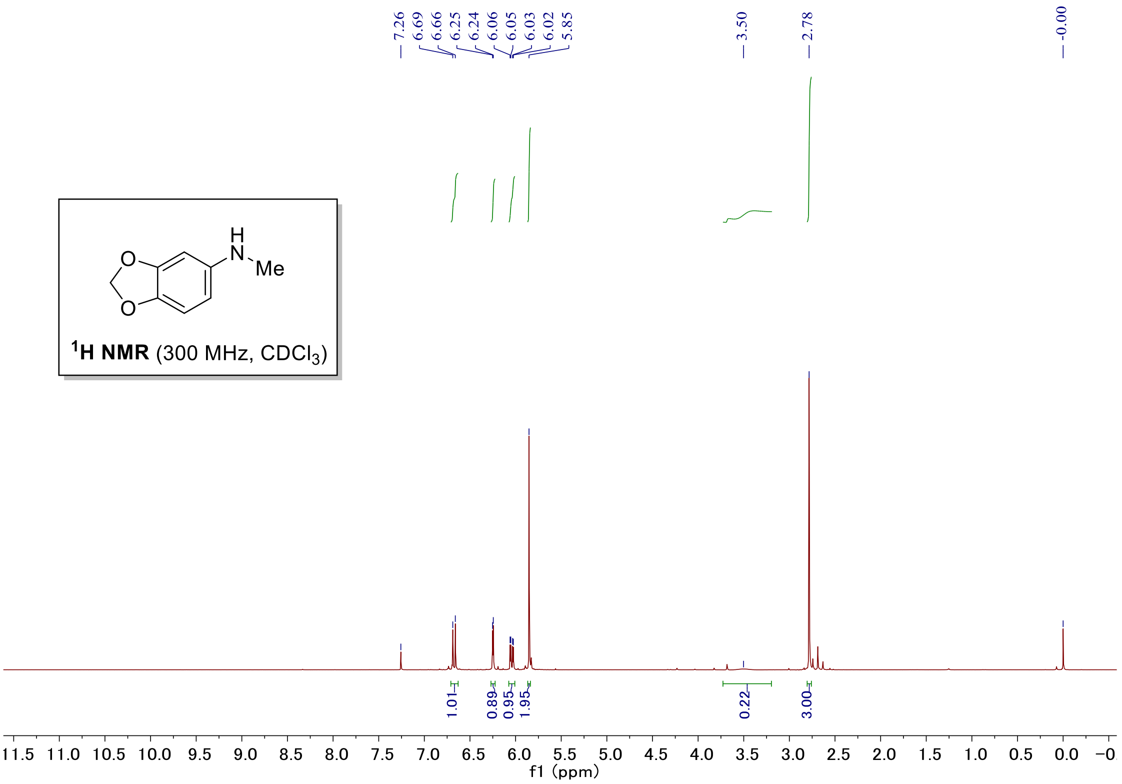

Supplementary Figure 84. ^{1}H NMR (300 MHz, $CDCl_3$, 25 °C) of compound **3g**

N-Methyl-4-(trifluoromethoxy)aniline (3h)

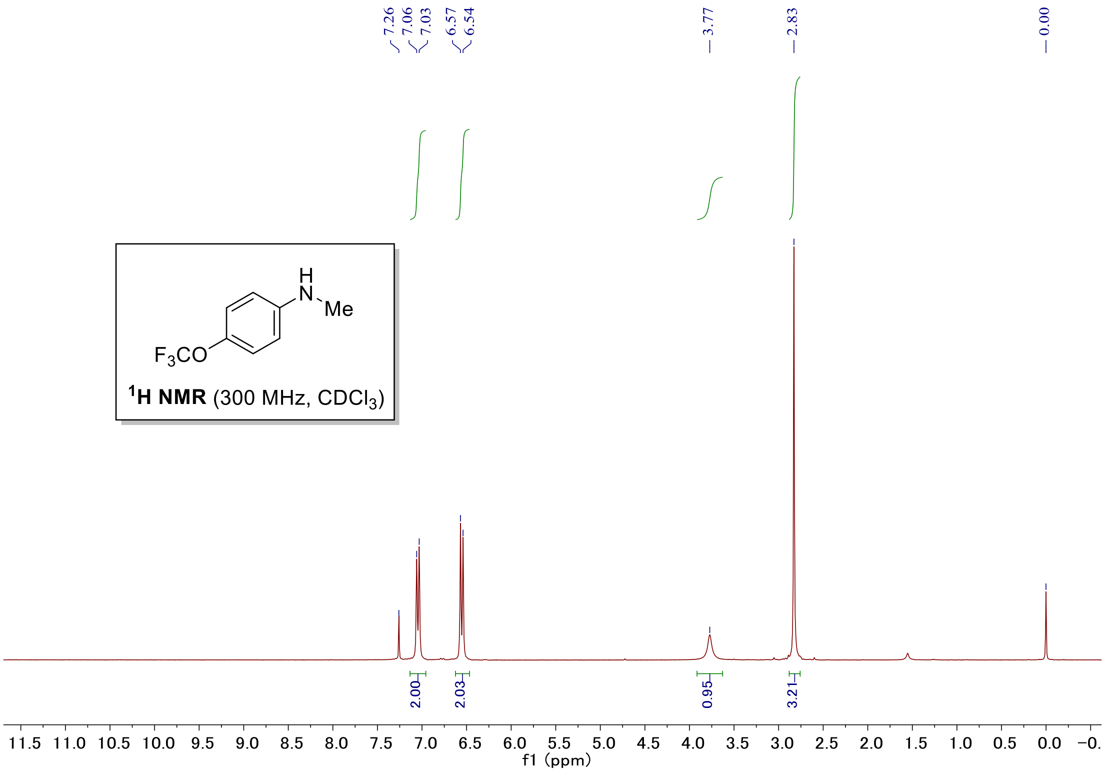

Supplementary Figure 85. ^{1}H NMR (300 MHz, $CDCl_3$, 25 °C) of compound **3h**

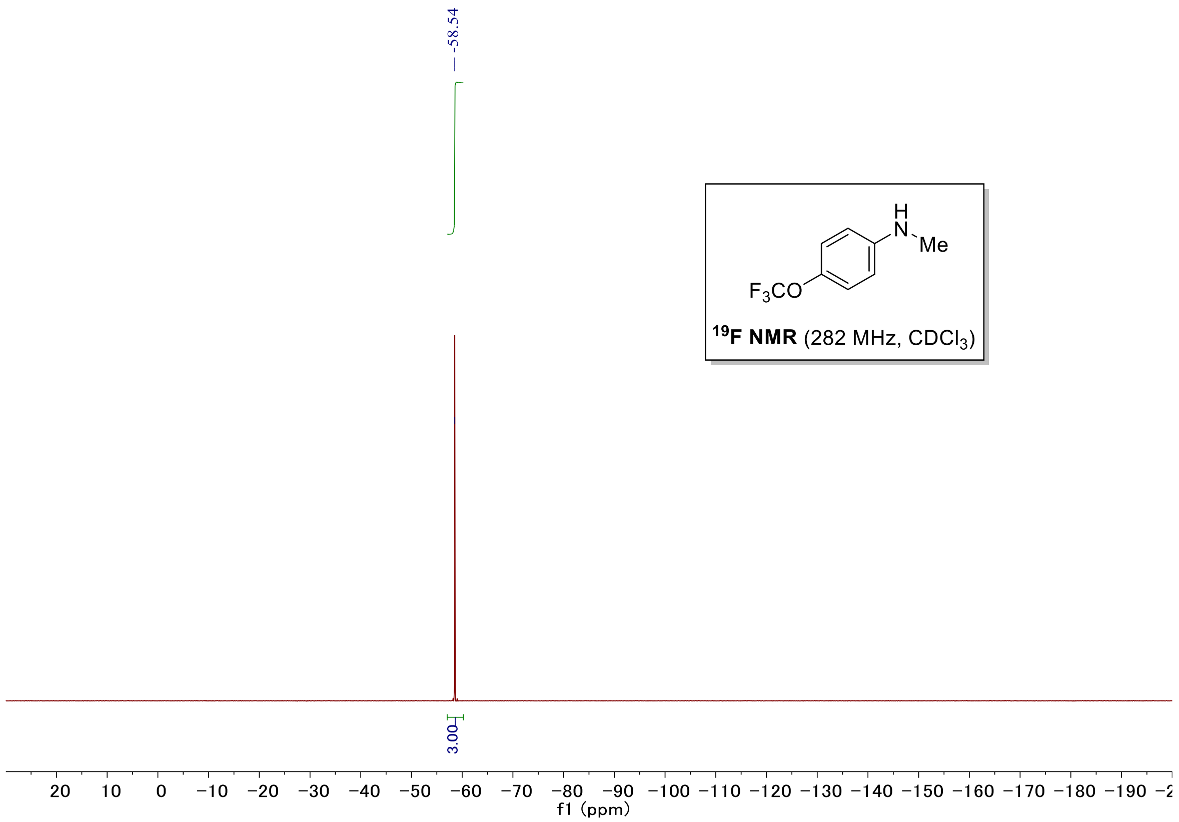

Supplementary Figure 86. ^{19}F NMR (282 MHz, $CDCl_3$, 25 °C) of compound **3h**

4-Chloro-*N*-methylaniline (3i)

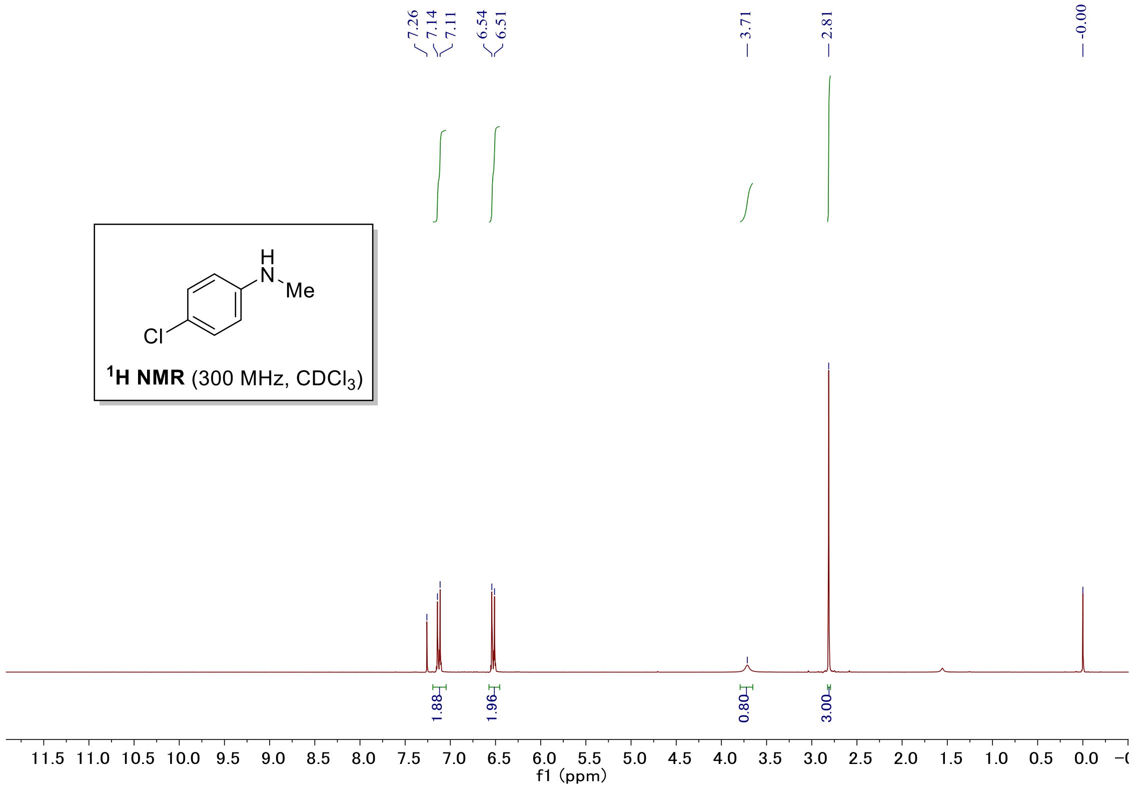

Supplementary Figure 87. ^{1}H NMR (300 MHz, $CDCl_3$, 25 °C) of compound **3i**

3-Chloro-*N*-methylaniline (3j)

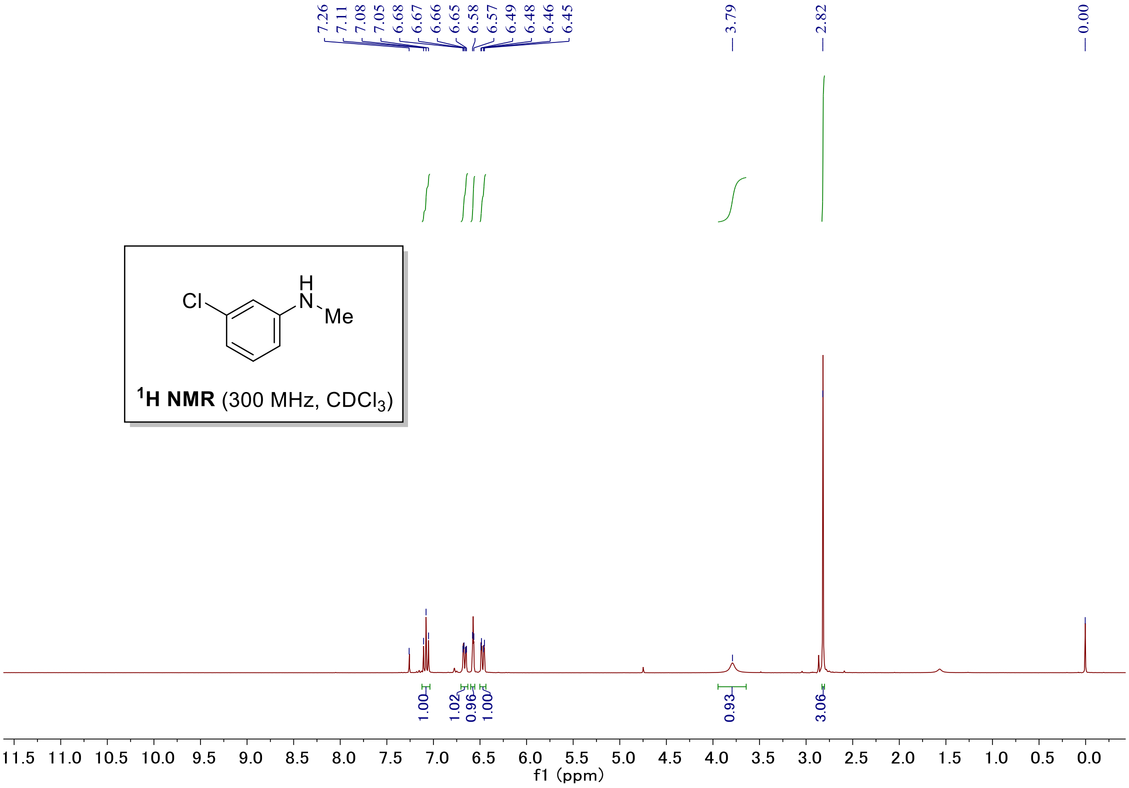

Supplementary Figure 88. ^{1}H NMR (300 MHz, $CDCl_3$, 25 ^{o}C) of compound **3j**

4-Bromo-*N*-methylaniline (3k)

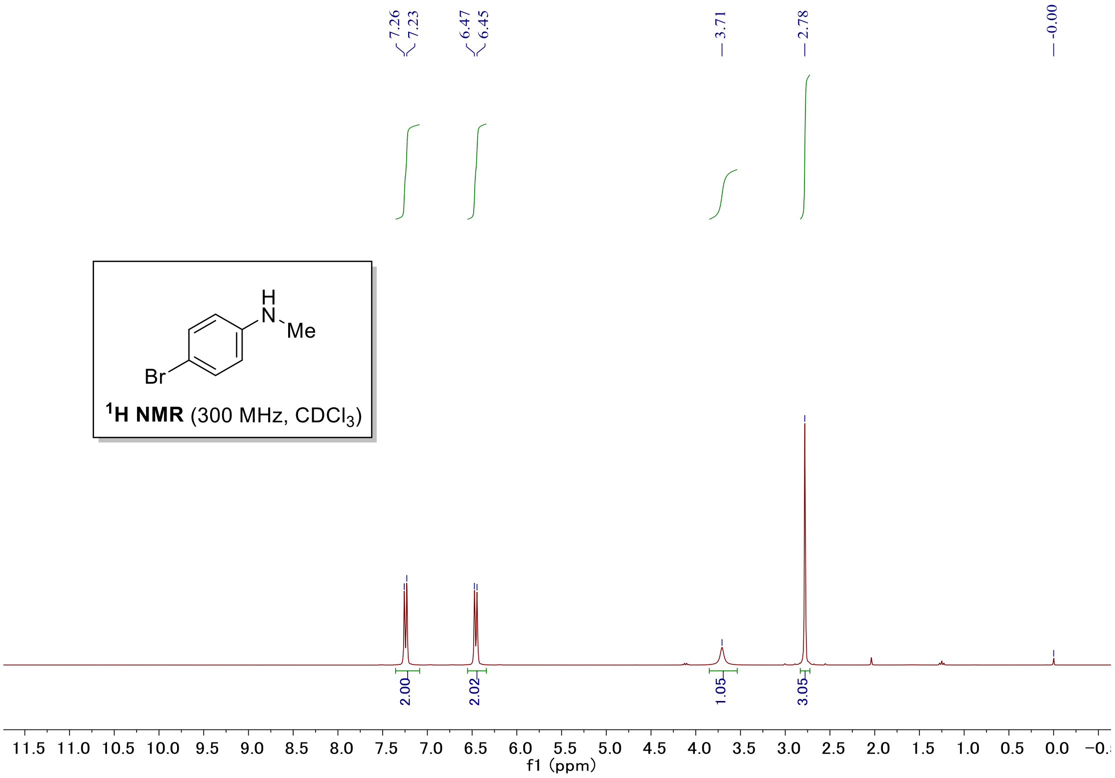

Supplementary Figure 89. ^{1}H NMR (300 MHz, $CDCl_3$, 25 ^{o}C) of compound **3k**

***N*-Butylaniline (3m)**

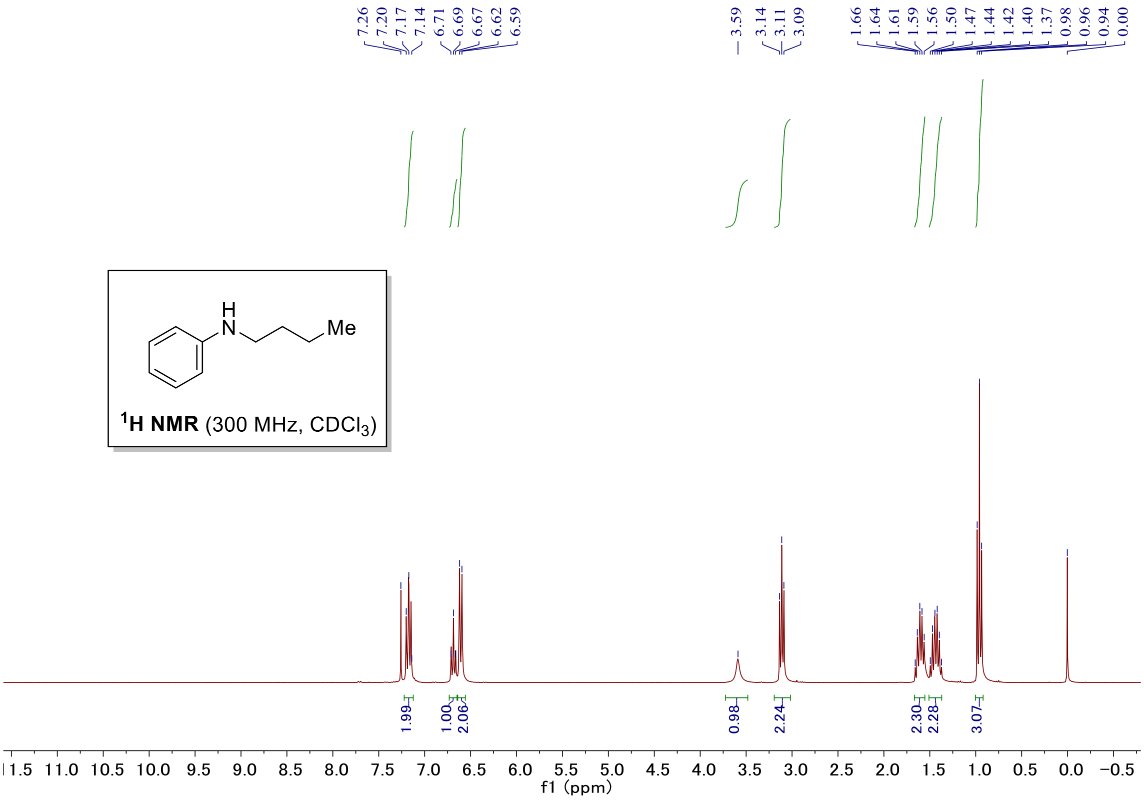

Supplementary Figure 90. ^{1}H NMR (300 MHz, $CDCl_3$, 25 °C) of compound **3m**

(*1R,2S,5R*)-2-Isopropyl-5-methylcyclohexyl 4-(methylamino)benzoate (3v)

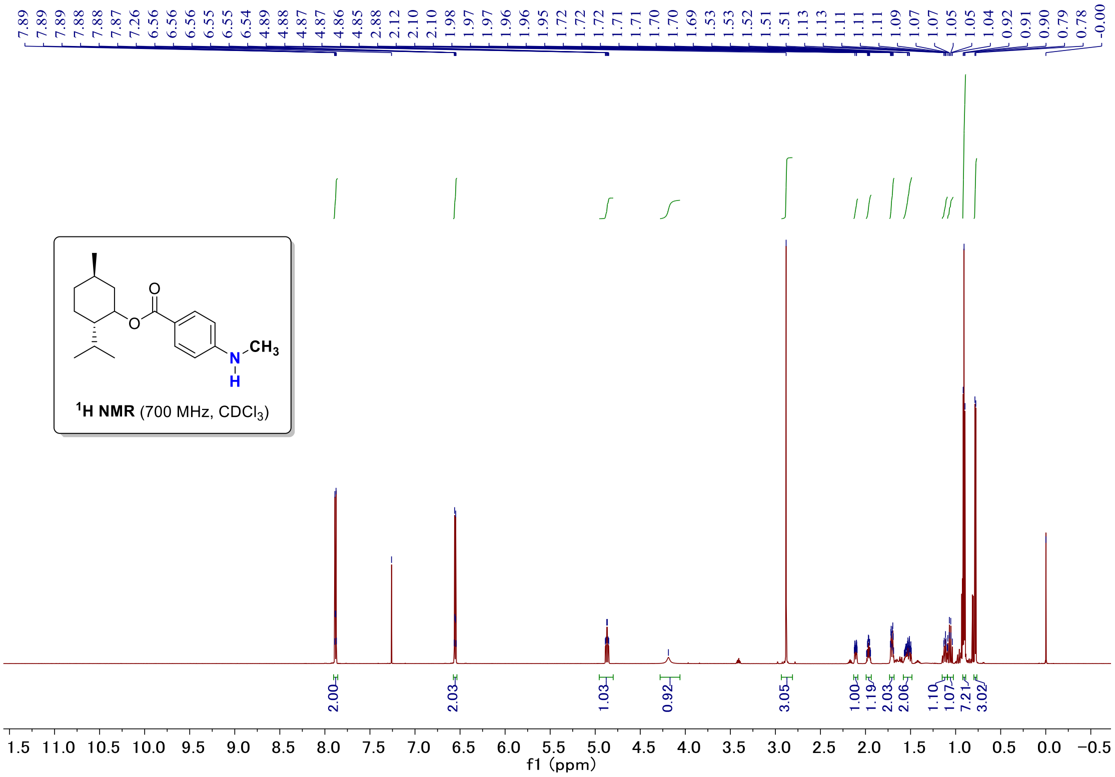

Supplementary Figure 91. ^{1}H NMR (300 MHz, $CDCl_3$, 25 °C) of compound **3v**

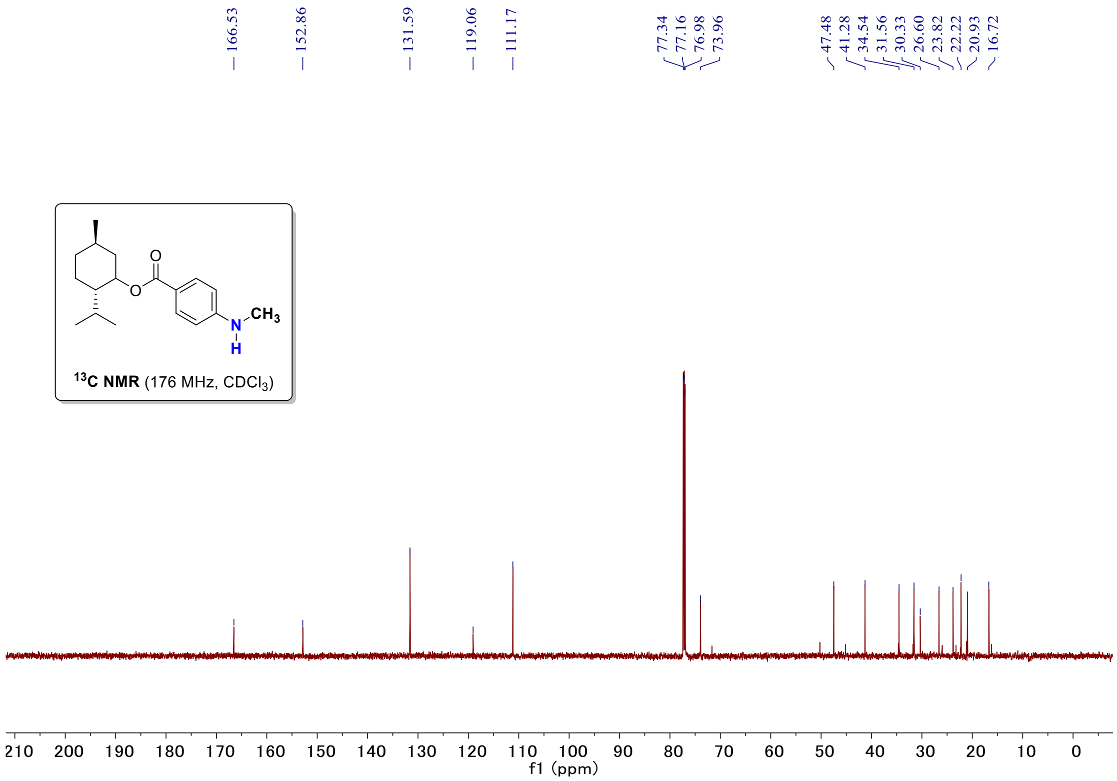

Supplementary Figure 92. ^{13}C NMR (176 MHz, $CDCl_3$, 25 °C) of compound **3v**

N-Cyclopropylaniline (3w)

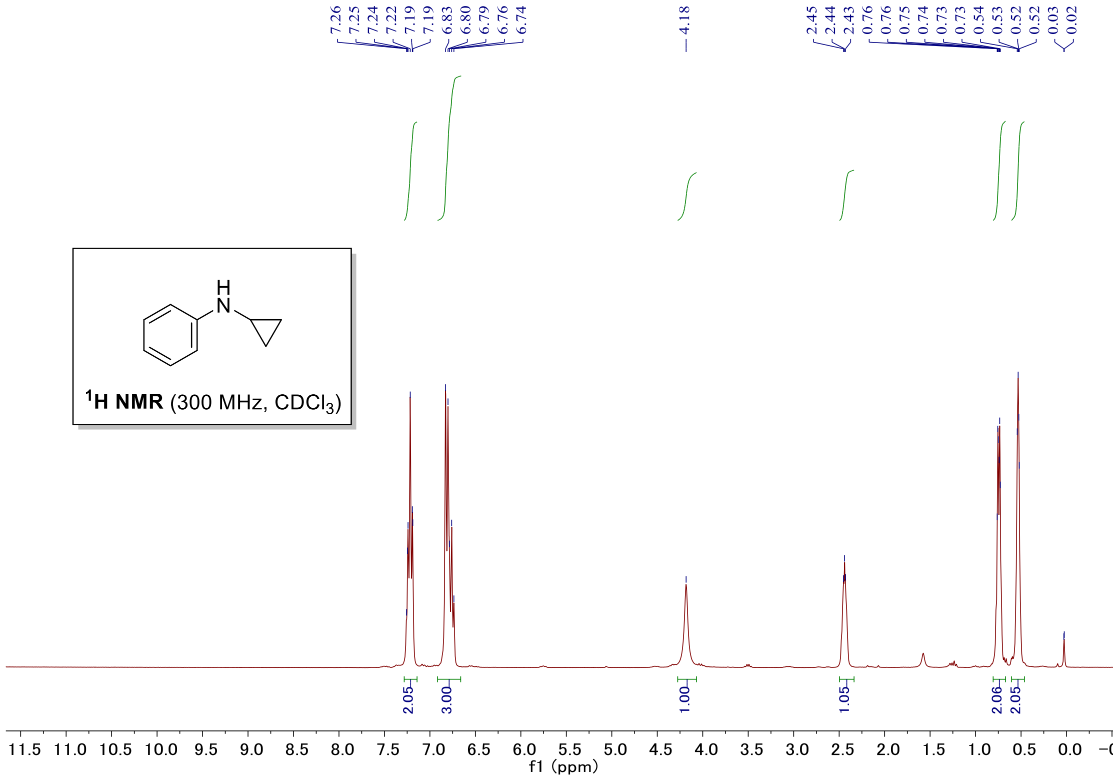

Supplementary Figure 93. ^{1}H NMR (300 MHz, $CDCl_3$, 25 °C) of compound **3w**

***N*-(Methyl-*d*^{3})aniline (*d*^{3}-3a)**

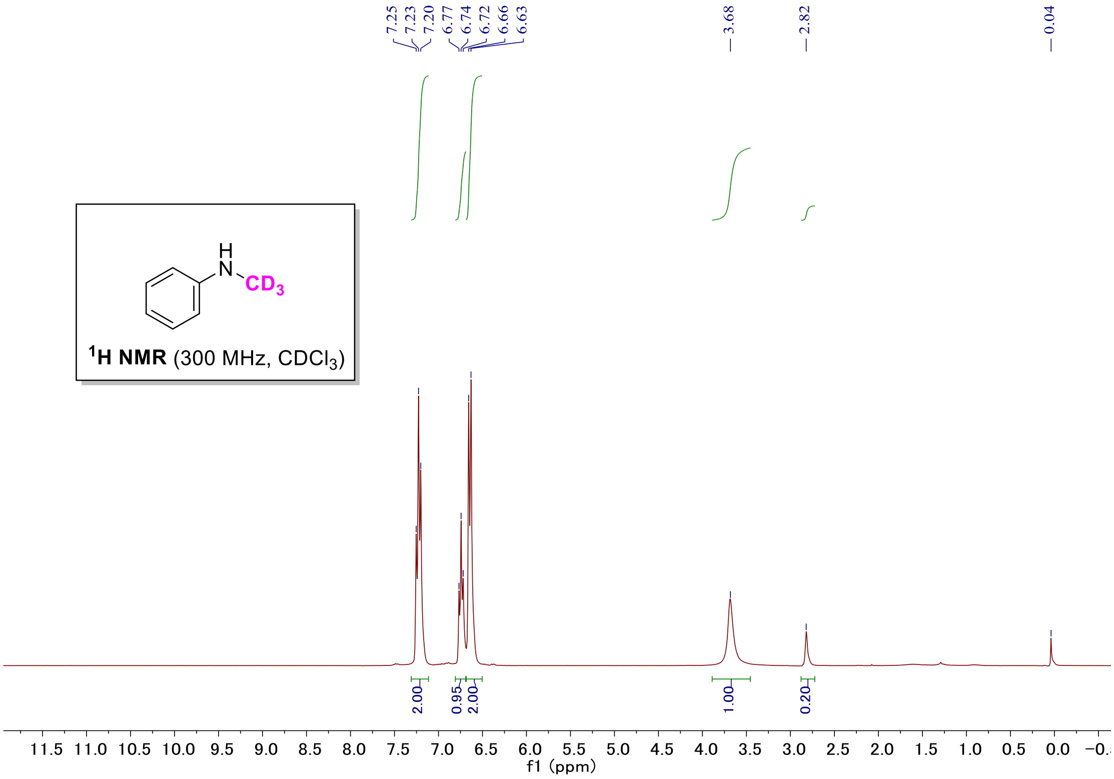

Supplementary Figure 94. ^{1}H NMR (300 MHz, $CDCl_3$, 25 ^oC) of compound ***d*^{3}-3a**

Triethyl(4,4,5,5-tetramethyl-1,3,2-dioxaborolan-2-yl)silane ($Et_3SiBpin$)

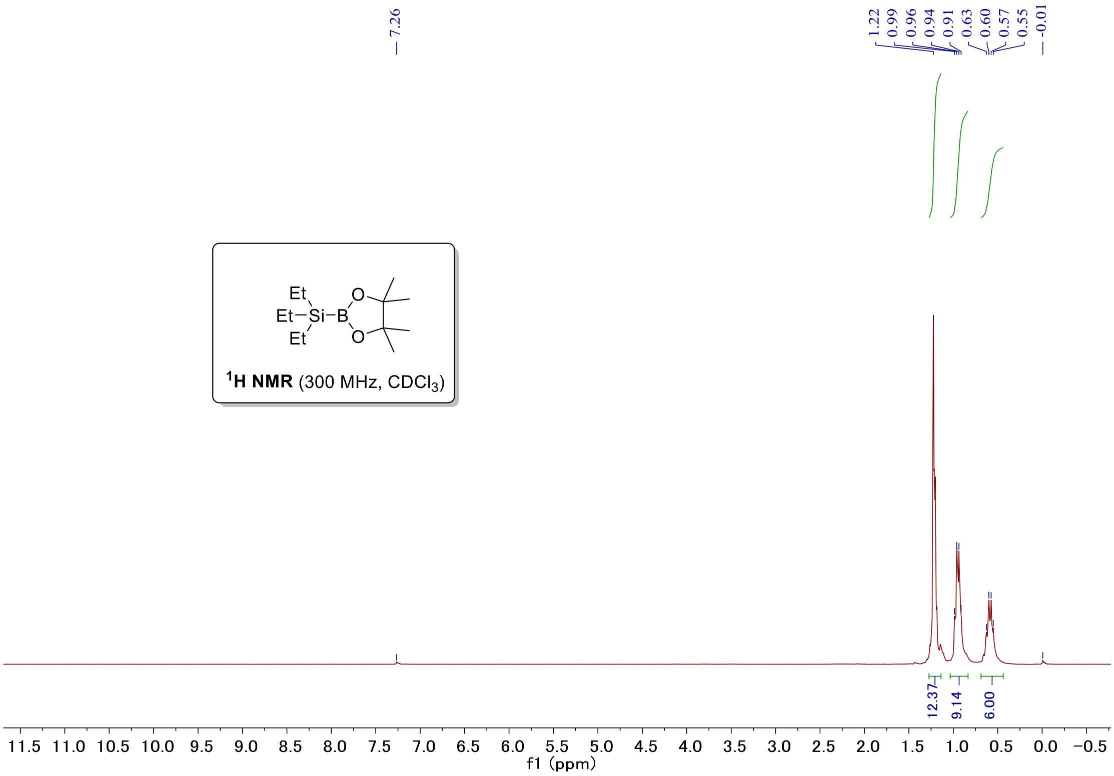

Supplementary Figure 95. ^{1}H NMR (300 MHz, $CDCl_3$, 25 ^oC) of compound **$Et_3SiBpin$**

Tripropyl(4,4,5,5-tetramethyl-1,3,2-dioxaborolan-2-yl)silane (*ⁿ*Pr3SiBpin)

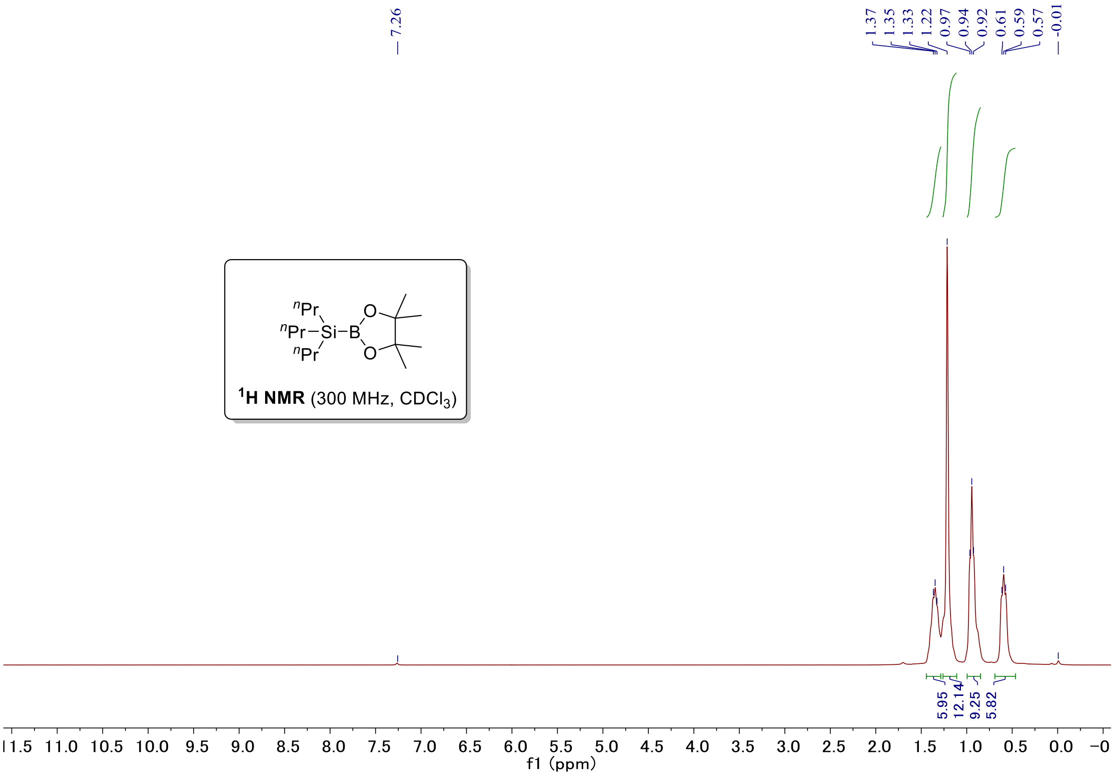

Supplementary Figure 96. ^{1}H NMR (300 MHz, $CDCl_3$, 25 °C) of compound ***ⁿ*Pr3SiBpin**

***tert*-Butyldimethyl(4,4,5,5-tetramethyl-1,3,2-dioxaborolan-2-yl)silane (*ᵗ*BuMe2SiBpin)**

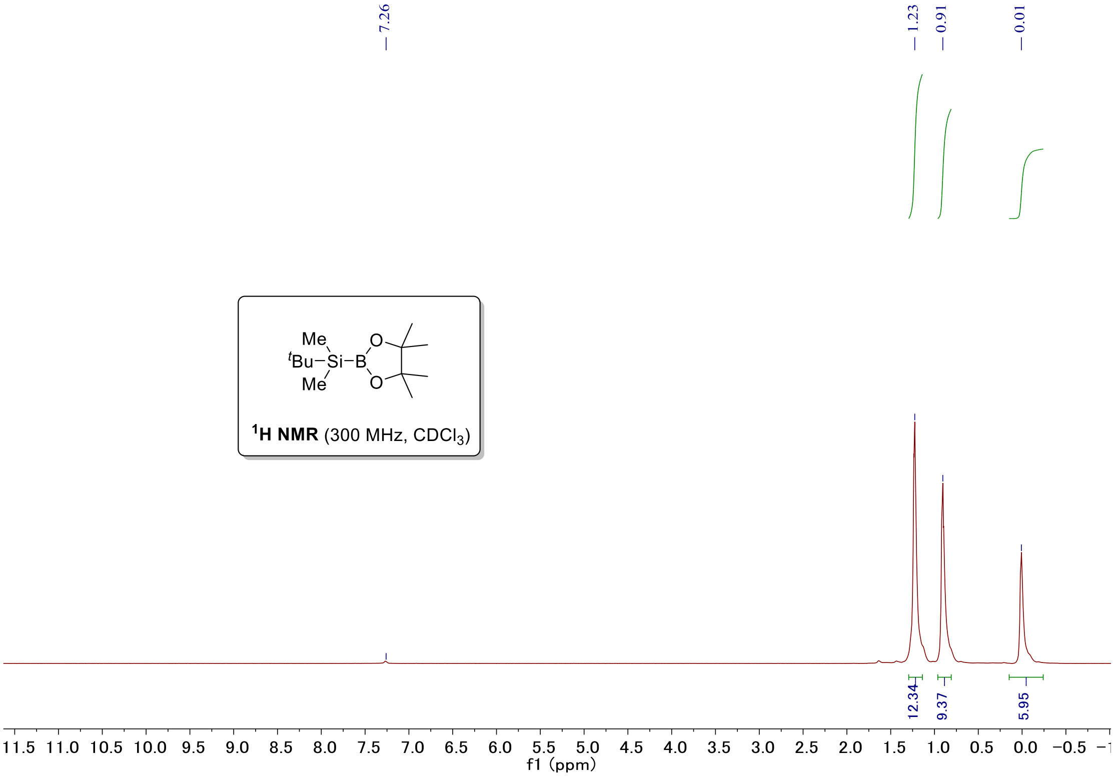

Supplementary Figure 97. ^{1}H NMR (300 MHz, $CDCl_3$, 25 °C) of compound ***ᵗ*BuMe2SiBpin**

1,1,1,3,3,3-Hexamethyl-2-(4,4,5,5-tetramethyl-1,3,2-dioxaborolan-2-yl)-2-(trimethylsilyl)trisilane ($TMS_3SiBpin$)

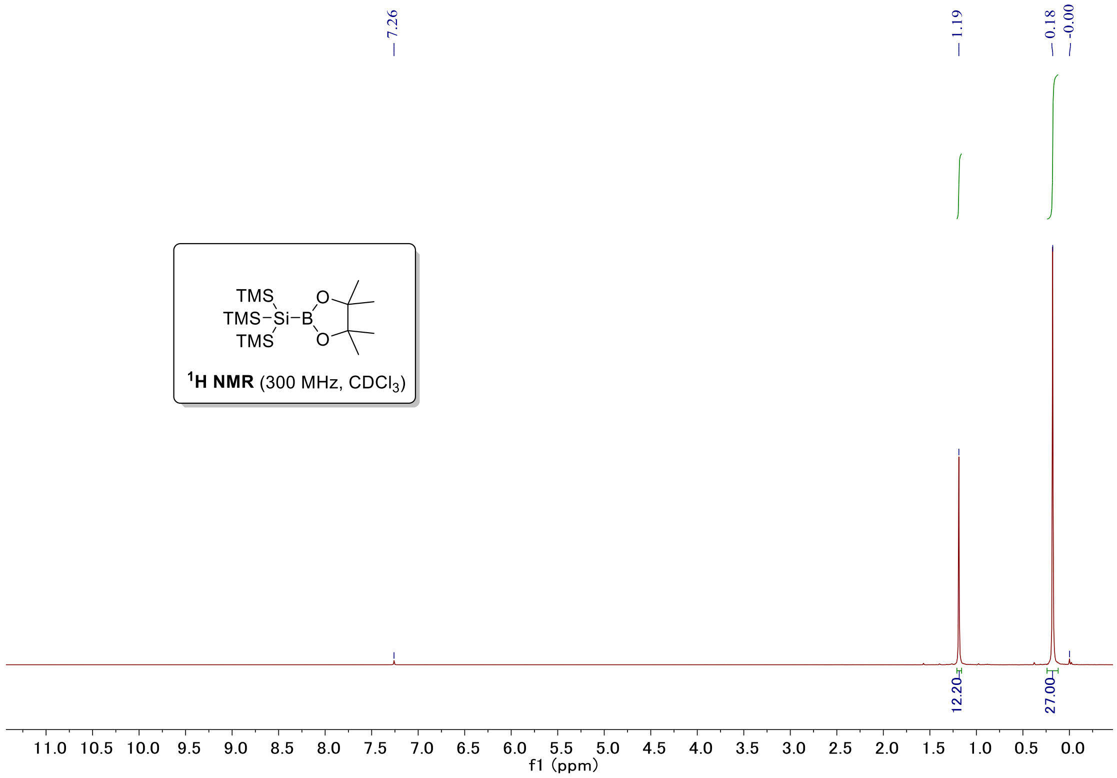

Supplementary Figure 98. ^1H NMR (300 MHz, $CDCl_3$, 25 °C) of compound **$TMS_3SiBpin$**

***N*-Methyl-*N*-phenyl-[1,1'-biphenyl]-4-amine (4aa)**

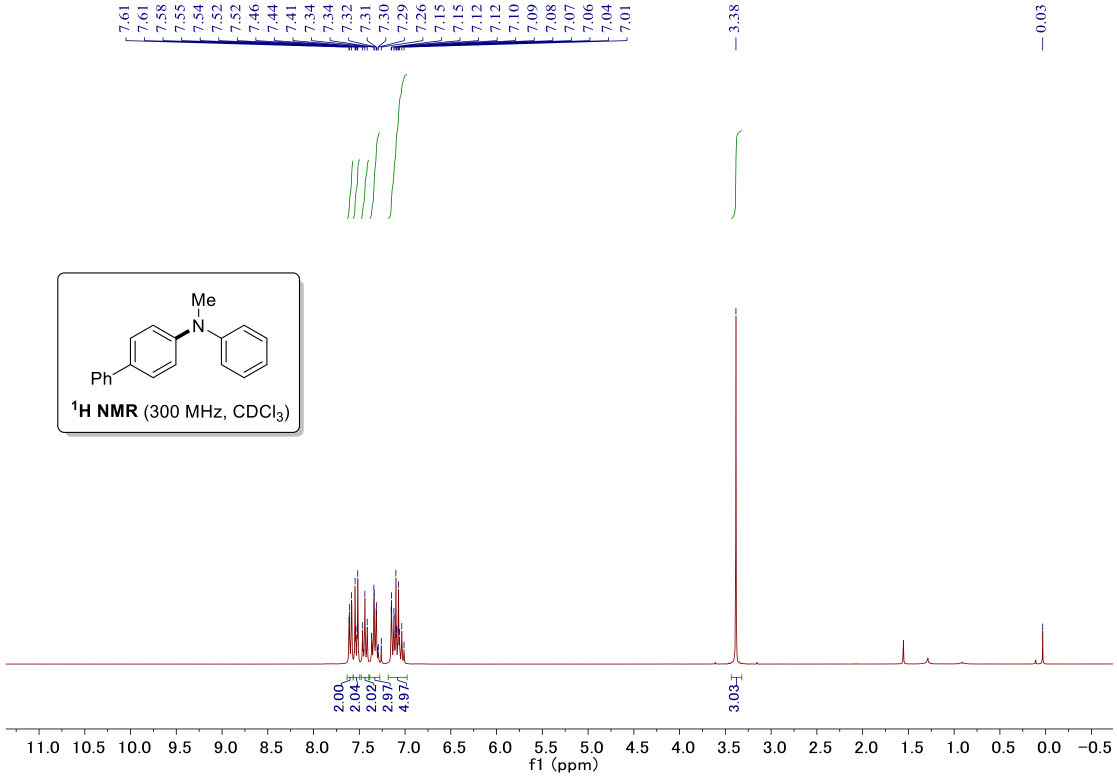

Supplementary Figure 99. ^1H NMR (300 MHz, $CDCl_3$, 25 °C) of compound **4aa**

N-Methyl-*N*-phenyl-[1,1'-biphenyl]-3-amine (4ba)

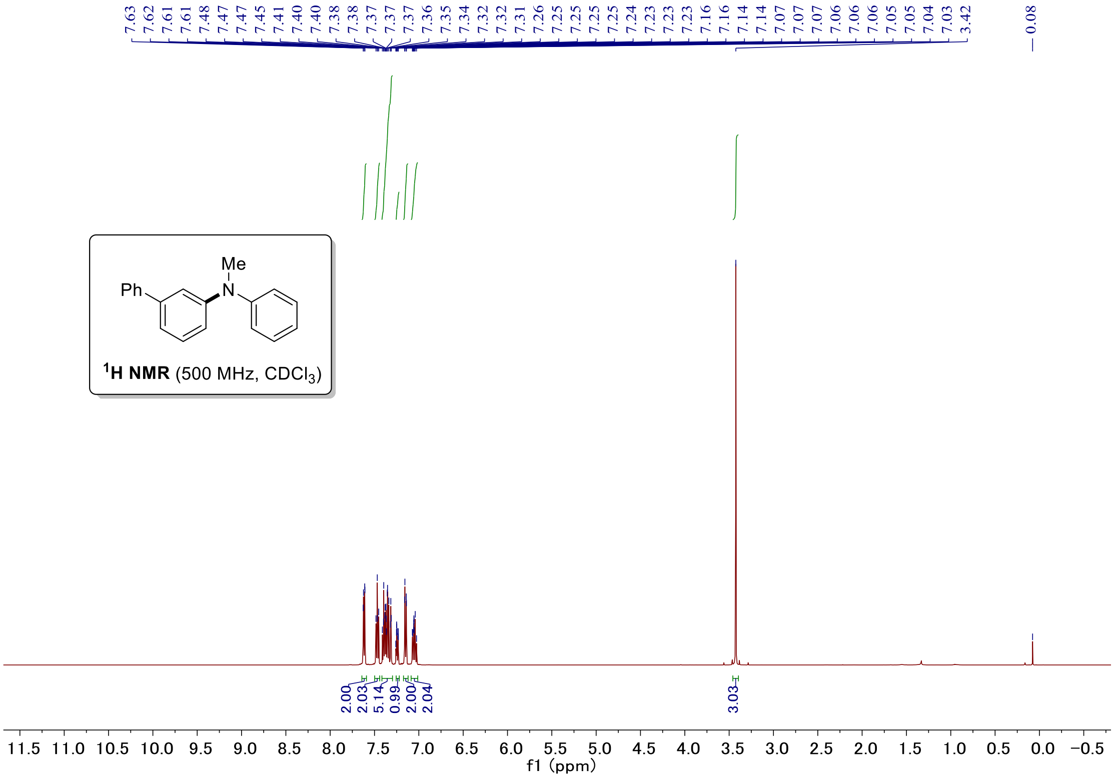

Supplementary Figure 100. ^{1}H NMR (500 MHz, $CDCl_3$, 25 °C) of compound **4ba**

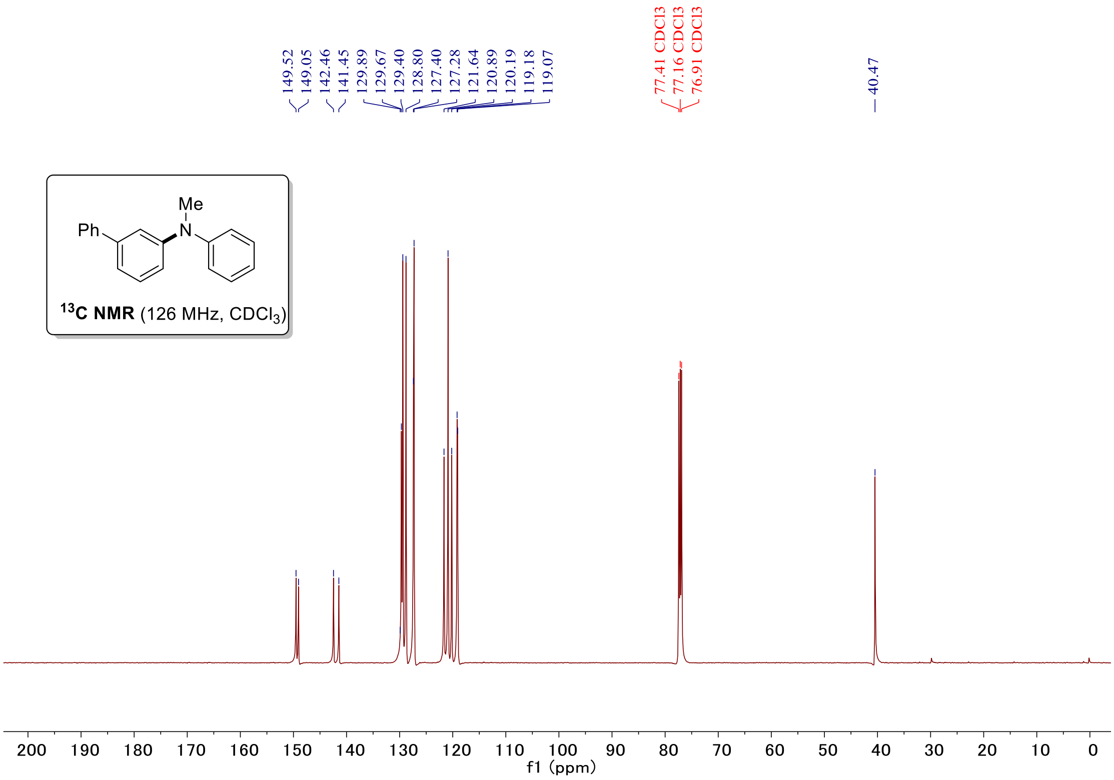

Supplementary Figure 101. ^{13}C NMR (126 MHz, $CDCl_3$, 25 °C) of compound **4ba**

N-Methyl-*N*-phenyl-[1,1'-biphenyl]-2-amine (4ca)

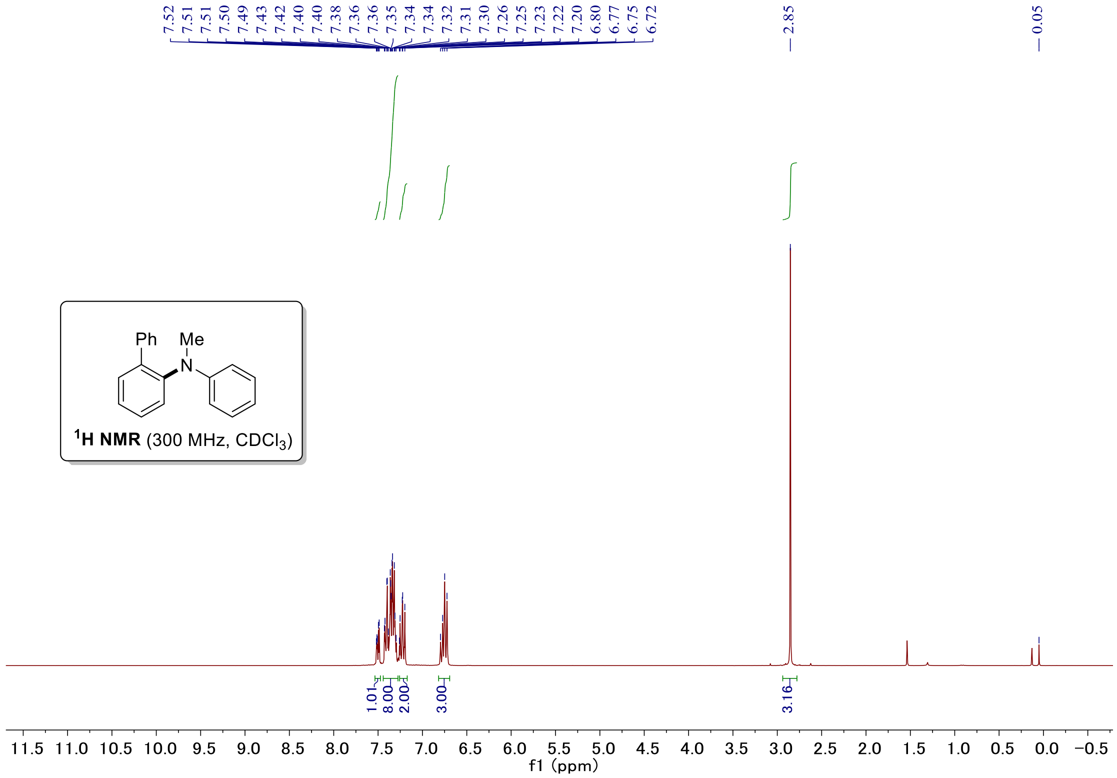

Supplementary Figure 102. ^{1}H NMR (300 MHz, $CDCl_3$, 25 °C) of compound **4ca**

N-Methyl-*N*-phenylnaphthalen-1-amine (4da)

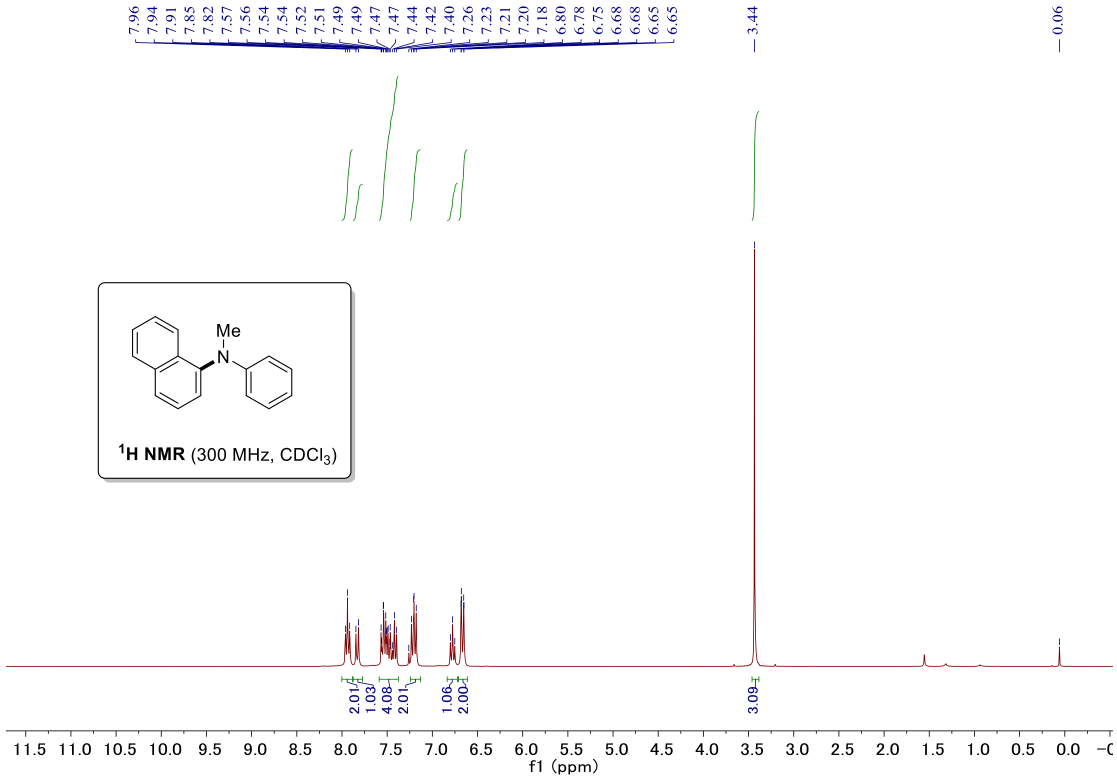

Supplementary Figure 103. ^{1}H NMR (300 MHz, $CDCl_3$, 25 °C) of compound **4da**

N-methyl-*N*-phenylaniline (4ea)

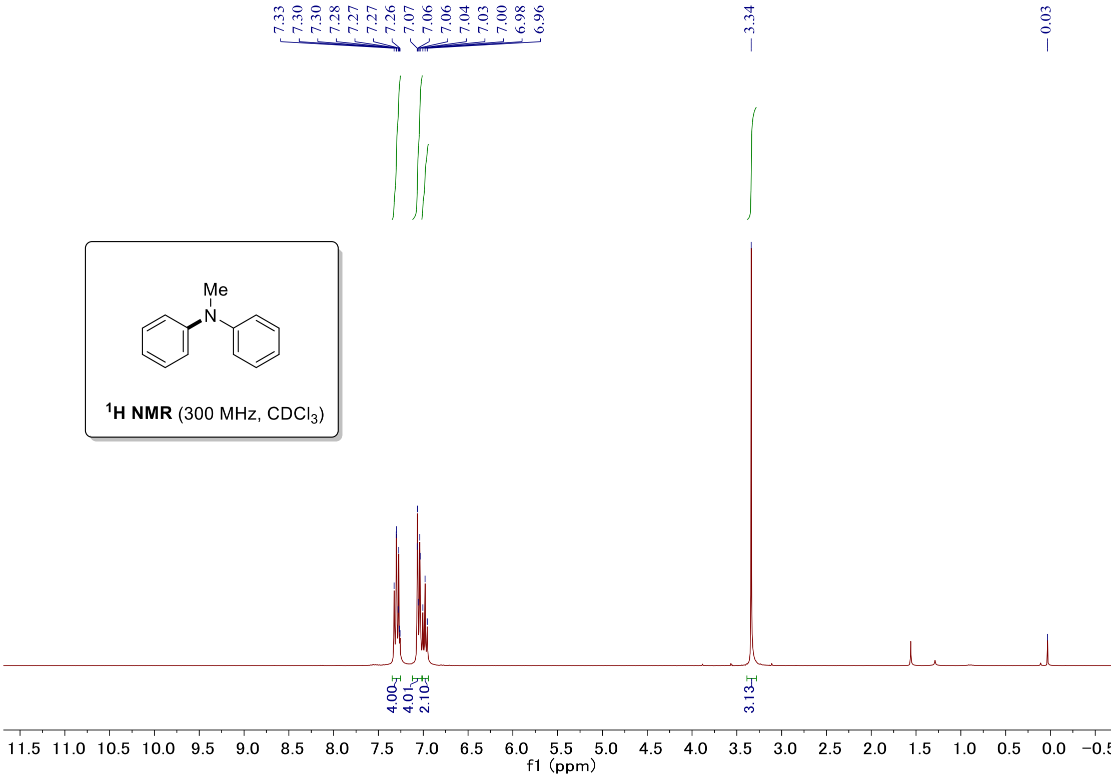

Supplementary Figure 104. ^{1}H NMR (300 MHz, $CDCl_3$, 25 °C) of compound **4ea**

N,4-Dimethyl-*N*-phenylaniline (4fa)

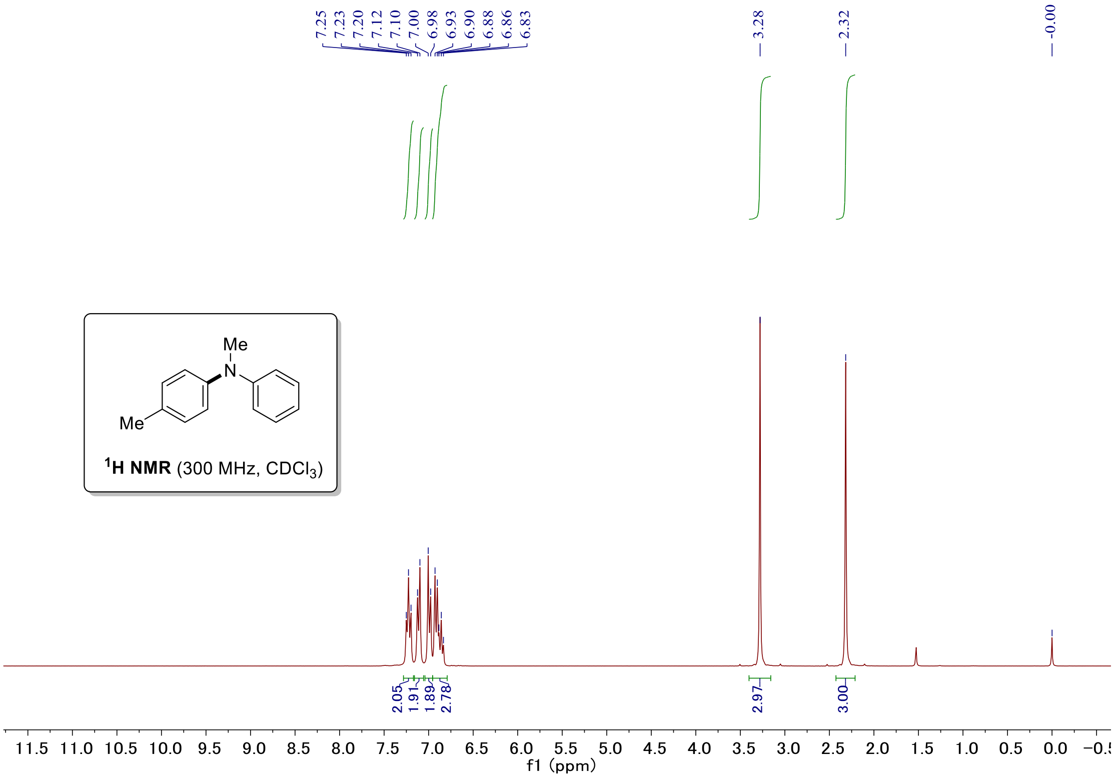

Supplementary Figure 105. ^{1}H NMR (300 MHz, $CDCl_3$, 25 °C) of compound **4fa**

4-Methoxy-*N*-methyl-*N*-phenylaniline (4ga)

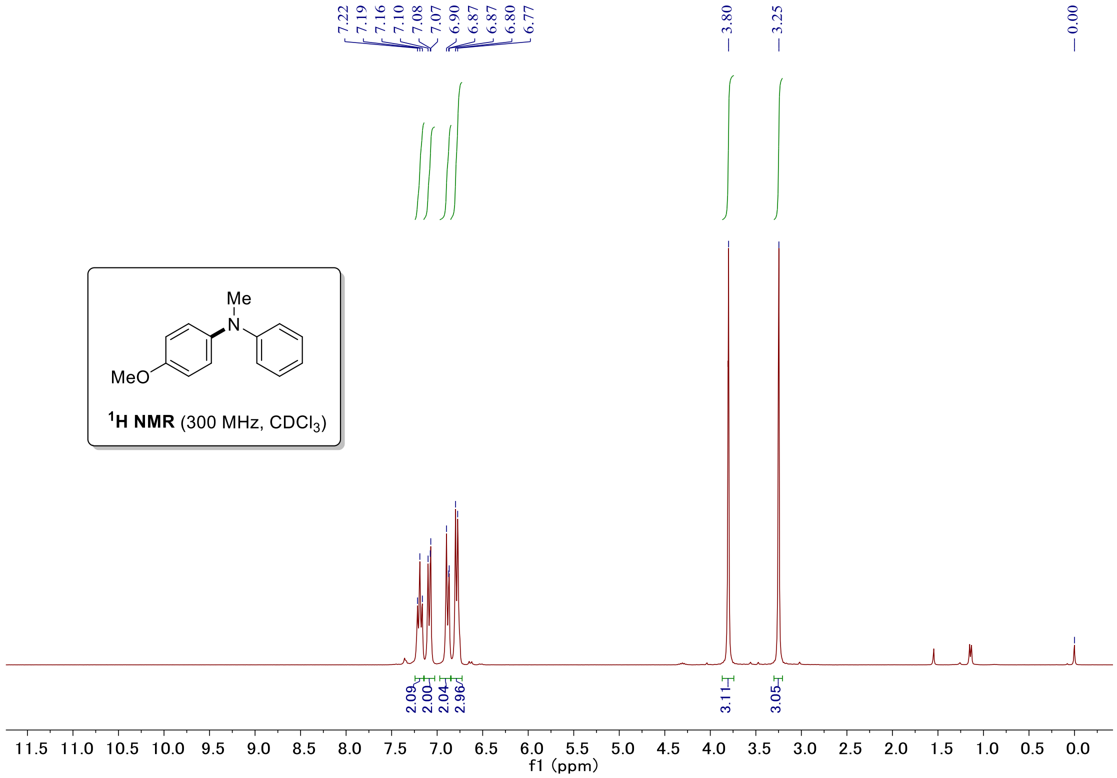

Supplementary Figure 106. ^{1}H NMR (300 MHz, $CDCl_3$, 25 °C) of compound **4ga**

***N*-Methyl-*N*-phenyl-4-(trifluoromethyl)aniline (4ha)**

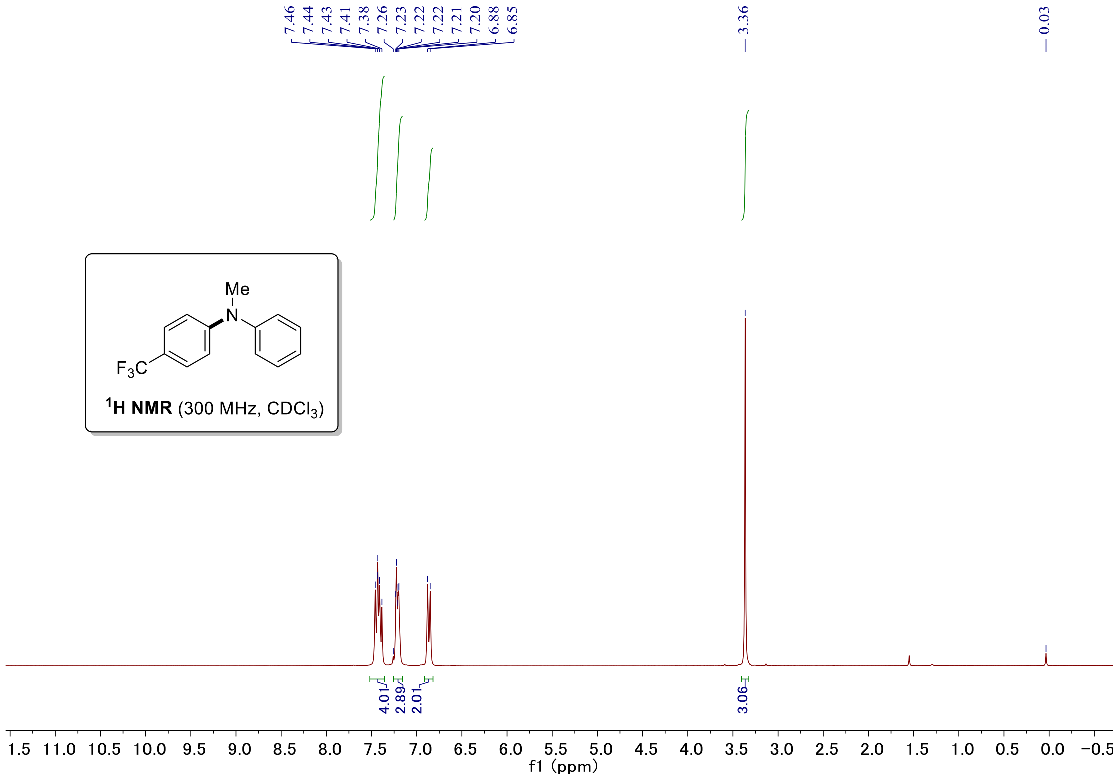

Supplementary Figure 107. ^{1}H NMR (300 MHz, $CDCl_3$, 25 °C) of compound **4ha**

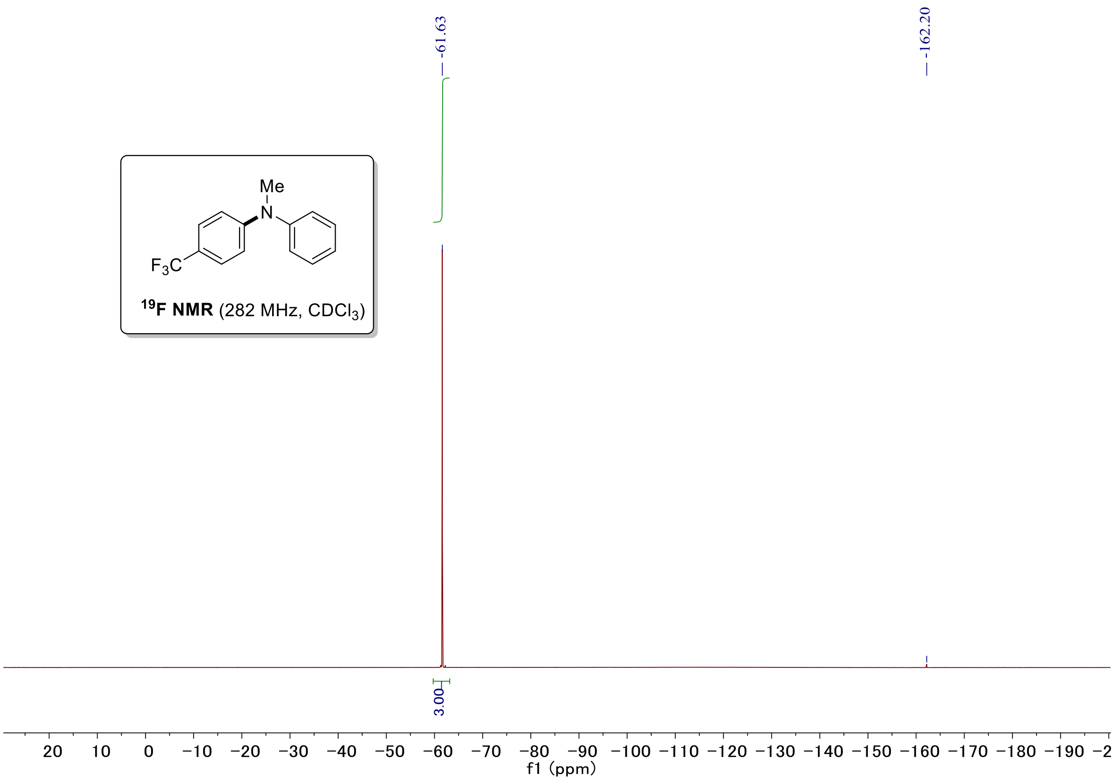

Supplementary Figure 108. ^{19}F NMR (282 MHz, $CDCl_3$, 25 °C) of compound **4ha**

4-Chloro-*N*-methyl-*N*-phenylaniline (4ia)

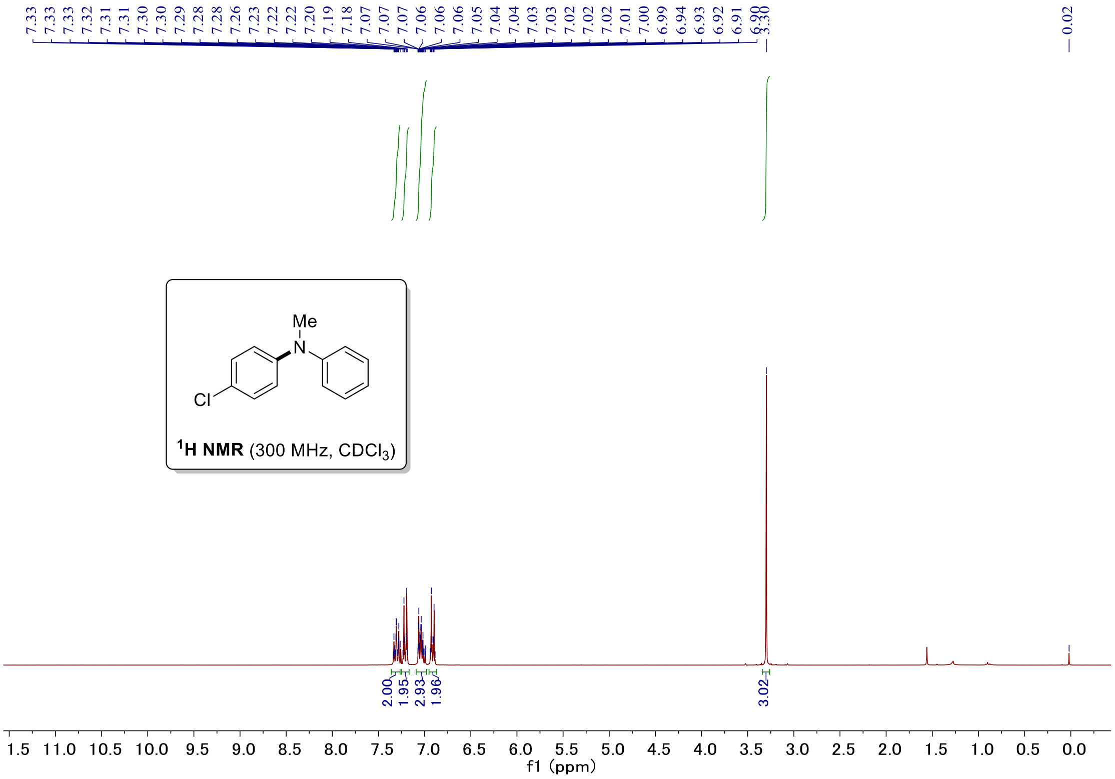

Supplementary Figure 109. ^{1}H NMR (300 MHz, $CDCl_3$, 25 °C) of compound **4ia**

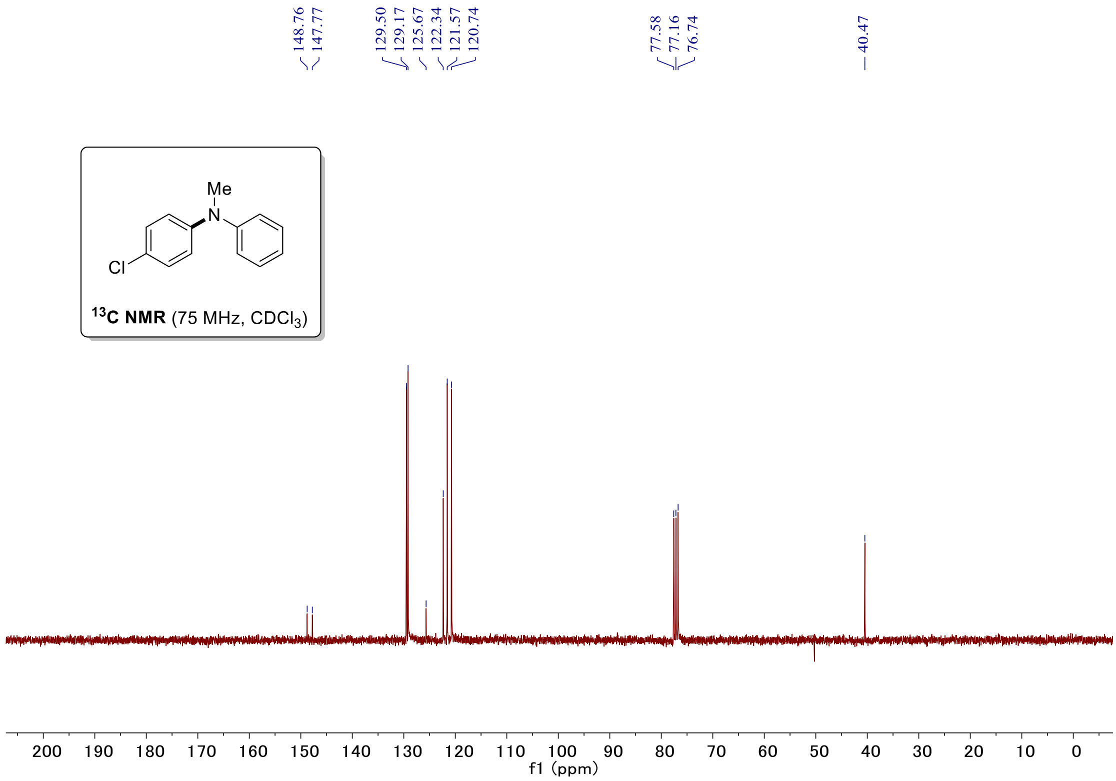

Supplementary Figure 110. ^{13}C NMR (75 MHz, $CDCl_3$, 25 °C) of compound **4ia**

3-Bromo-*N*-methyl-*N*-phenylaniline (4ja)

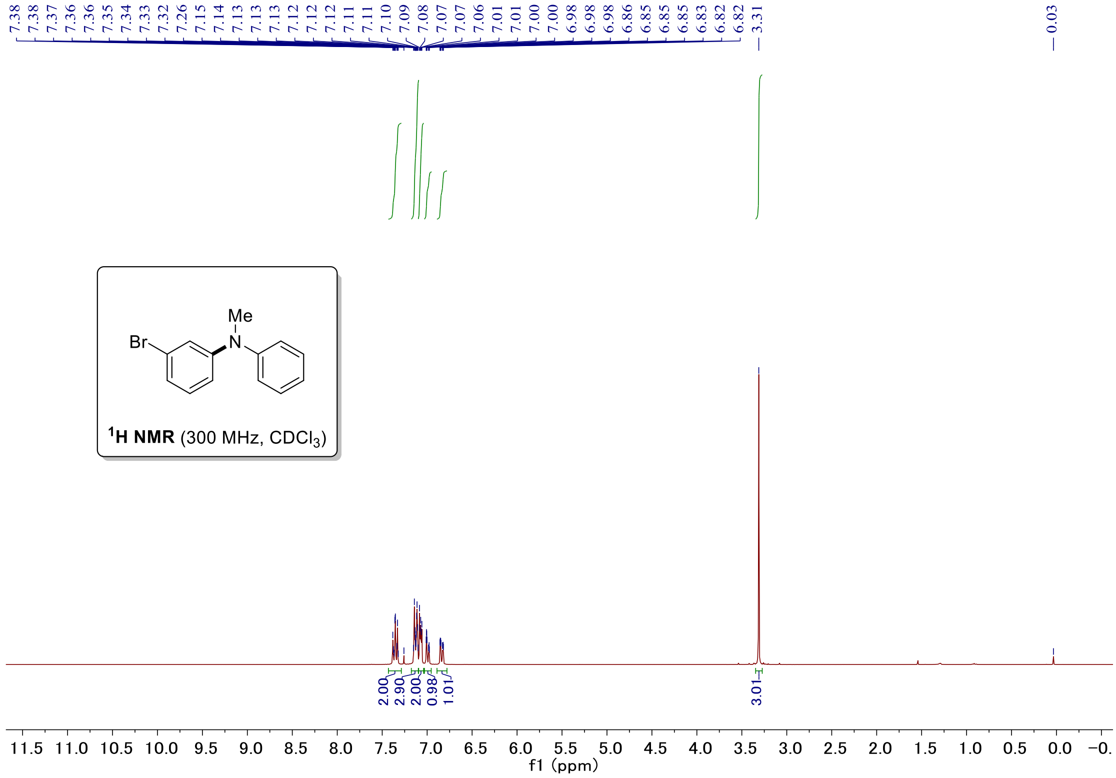

Supplementary Figure 111. ^{1}H NMR (300 MHz, $CDCl_3$, 25 °C) of compound **4ja**

N-Methyl-4-(naphthalen-1-yl)-*N*-phenylaniline (4ka)

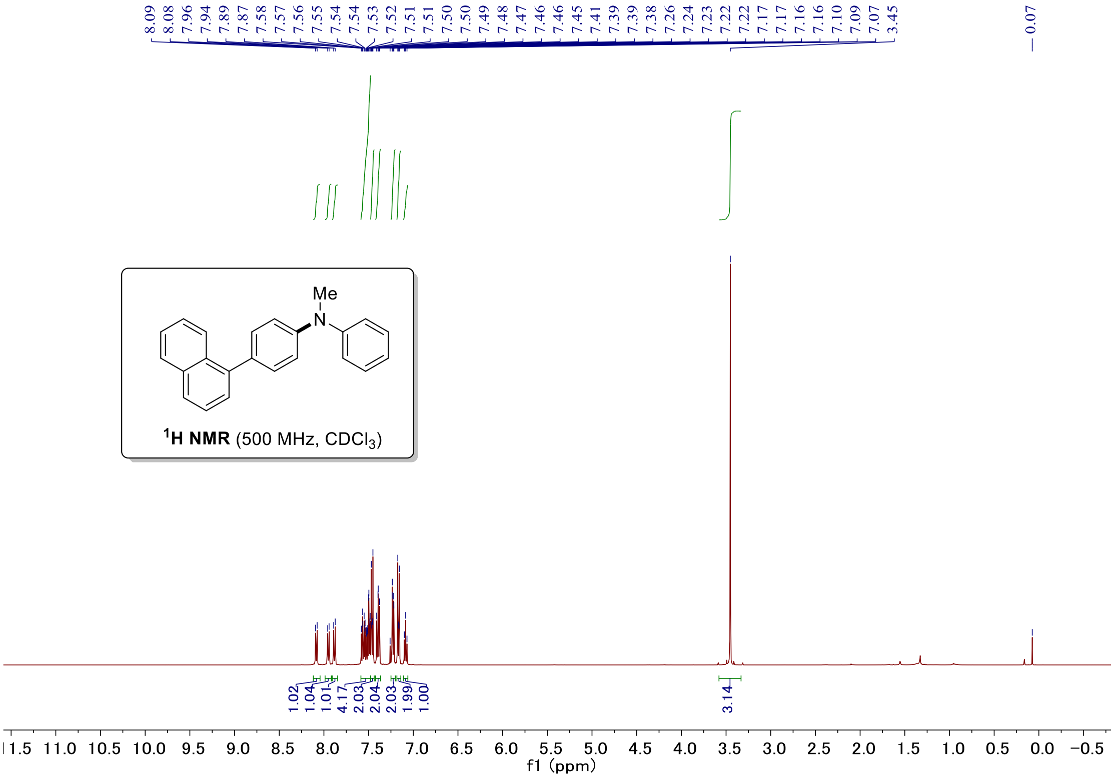

Supplementary Figure 112. ^{1}H NMR (500 MHz, $CDCl_3$, 25 °C) of compound **4ka**

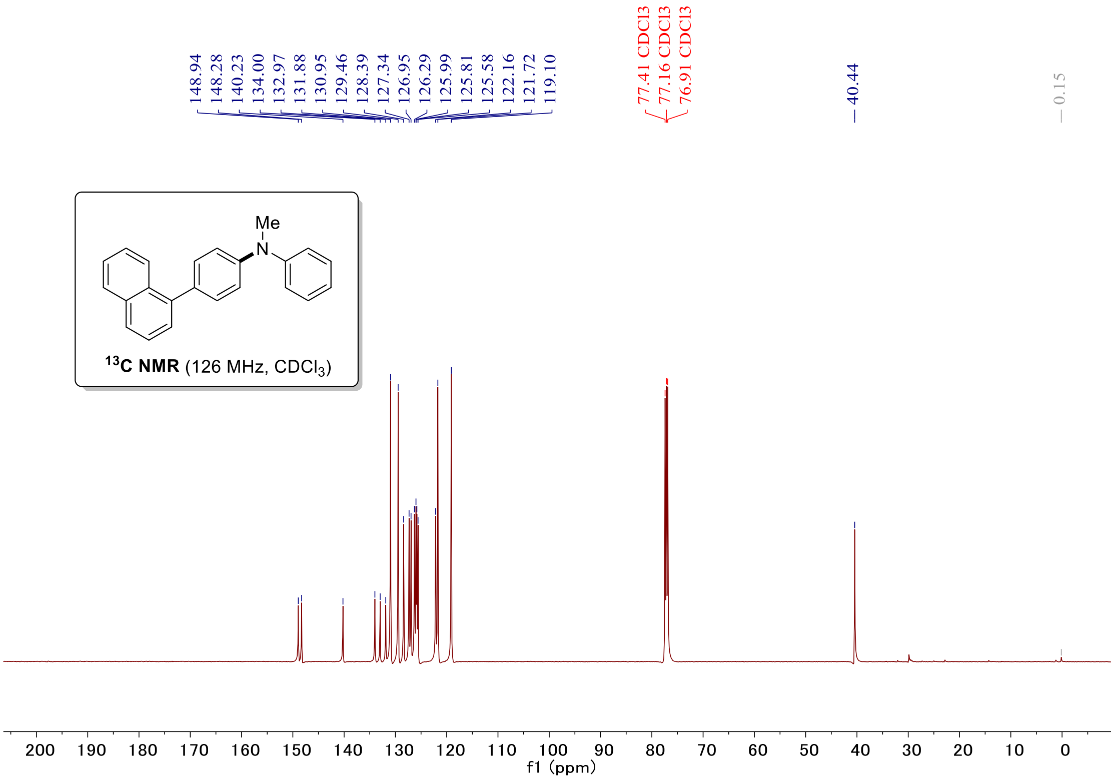

Supplementary Figure 113. ^{13}C NMR (126 MHz, $CDCl_3$, 25 °C) of compound **4ka**

***N*,4′-Dimethyl-*N*-phenyl-[1,1'-biphenyl]-4-amine (4la)**

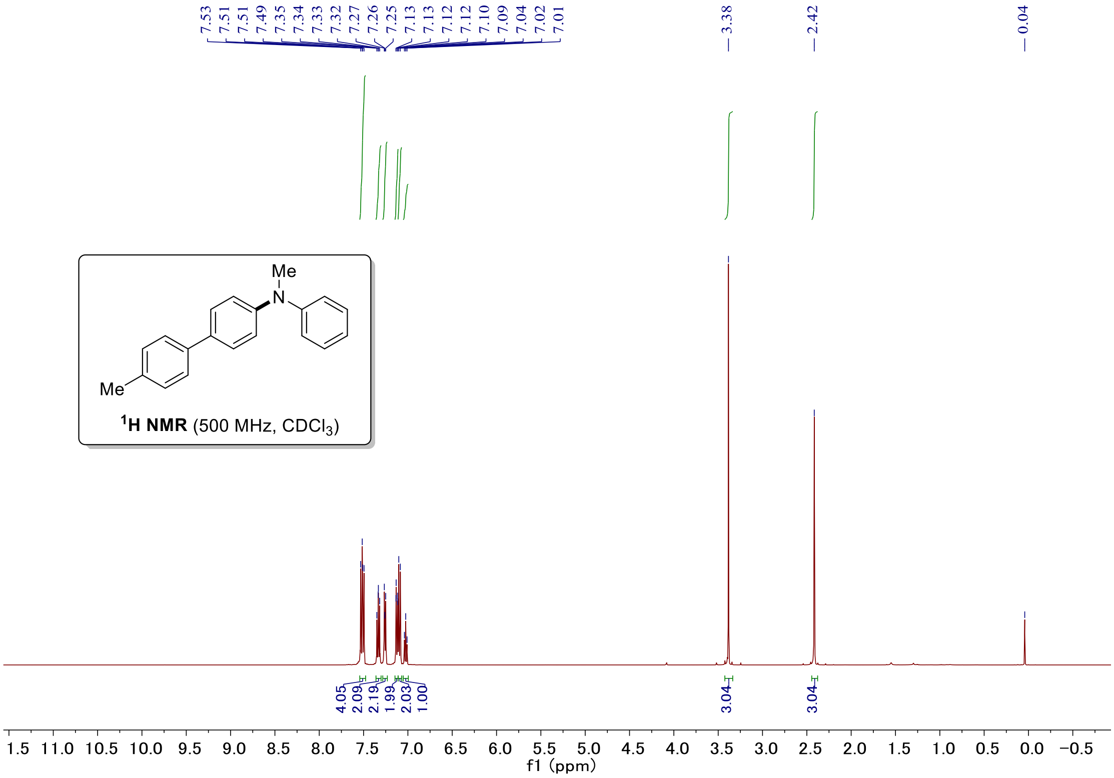

Supplementary Figure 114. ^{1}H NMR (500 MHz, $CDCl_3$, 25 °C) of compound **4la**

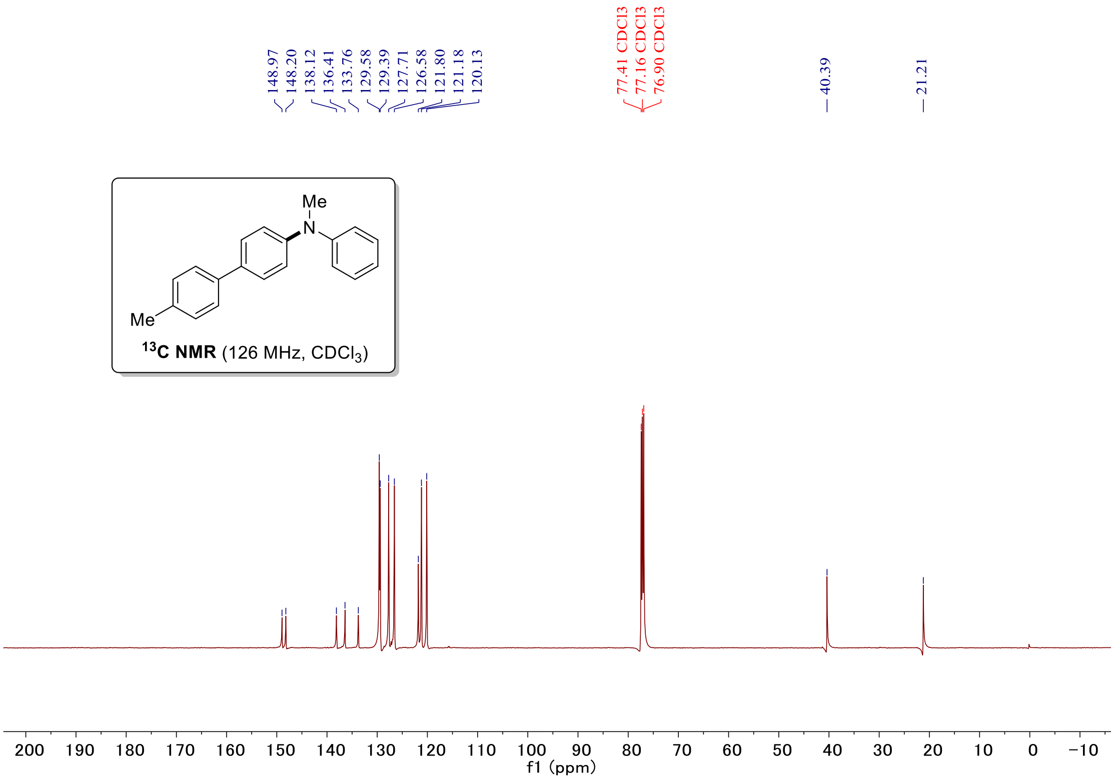

Supplementary Figure 115. ^{13}C NMR (126 MHz, $CDCl_3$, 25 °C) of compound **4la**

4'-Methoxy-*N*-methyl-*N*-phenyl-[1,1'-biphenyl]-4-amine (4ma)

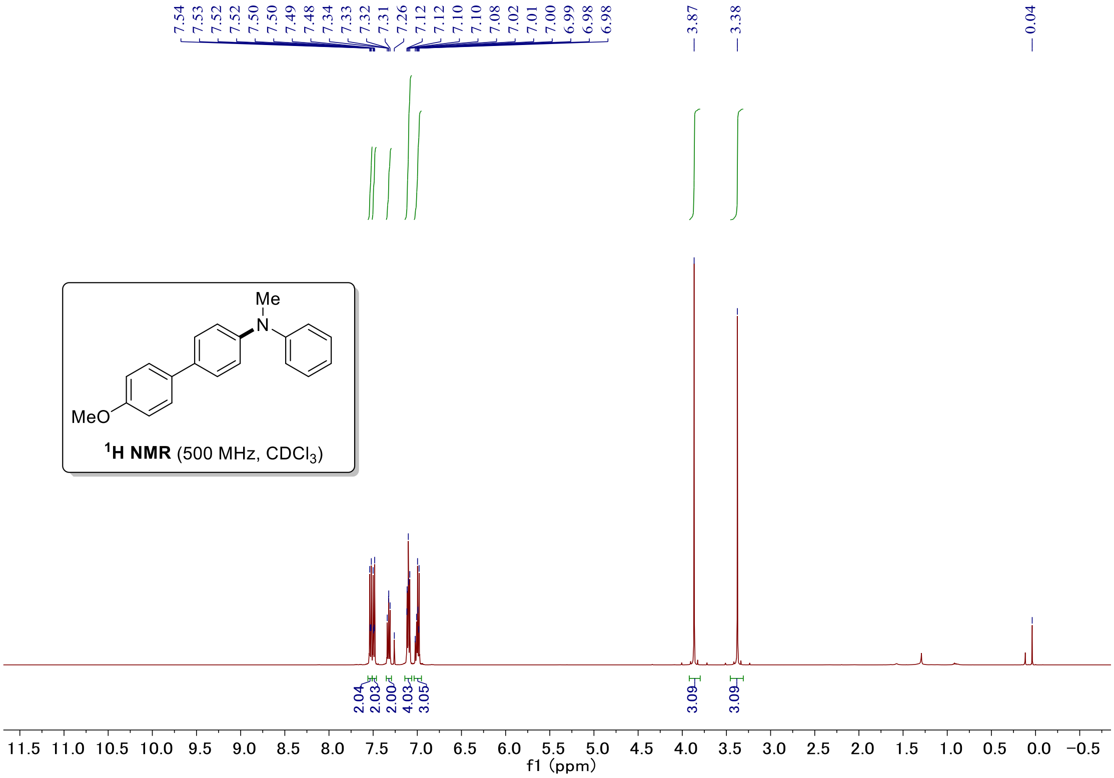

Supplementary Figure 116. ^{1}H NMR (500 MHz, $CDCl_3$, 25 °C) of compound **4ma**

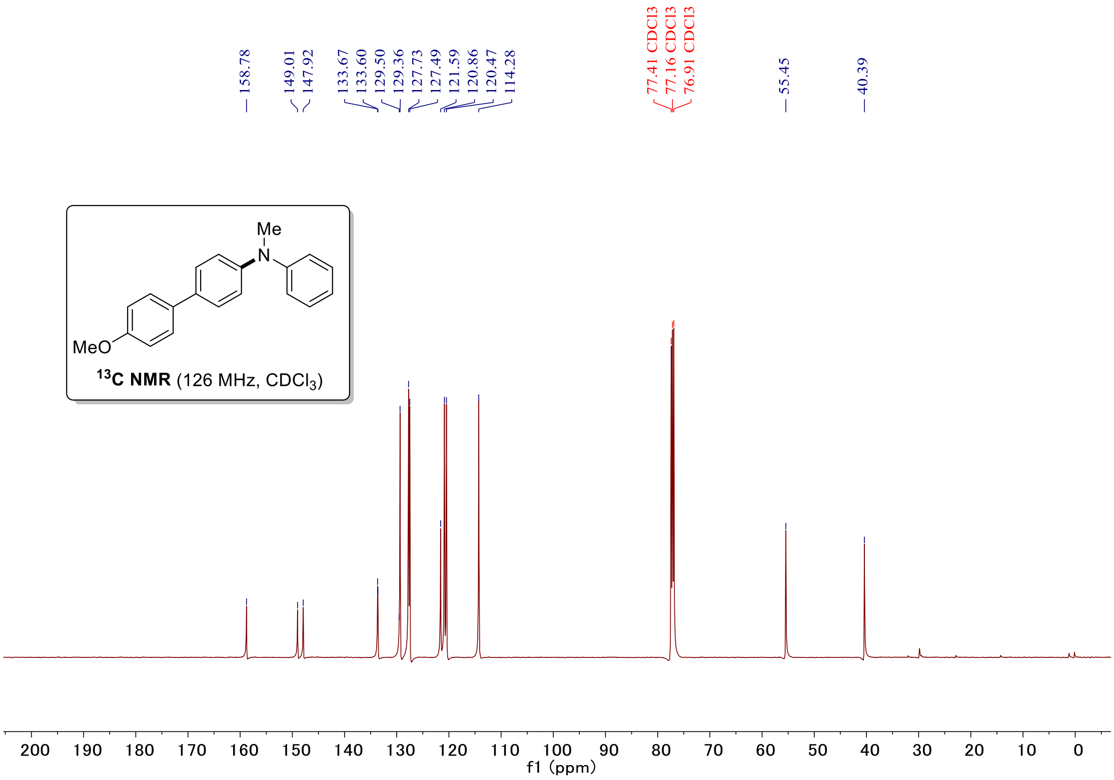

Supplementary Figure 117. ^{13}C NMR (126 MHz, $CDCl_3$, 25 °C) of compound **4ma**

4'-(Benzyloxy)-*N*-methyl-*N*-phenyl-[1,1'-biphenyl]-4-amine (4na)

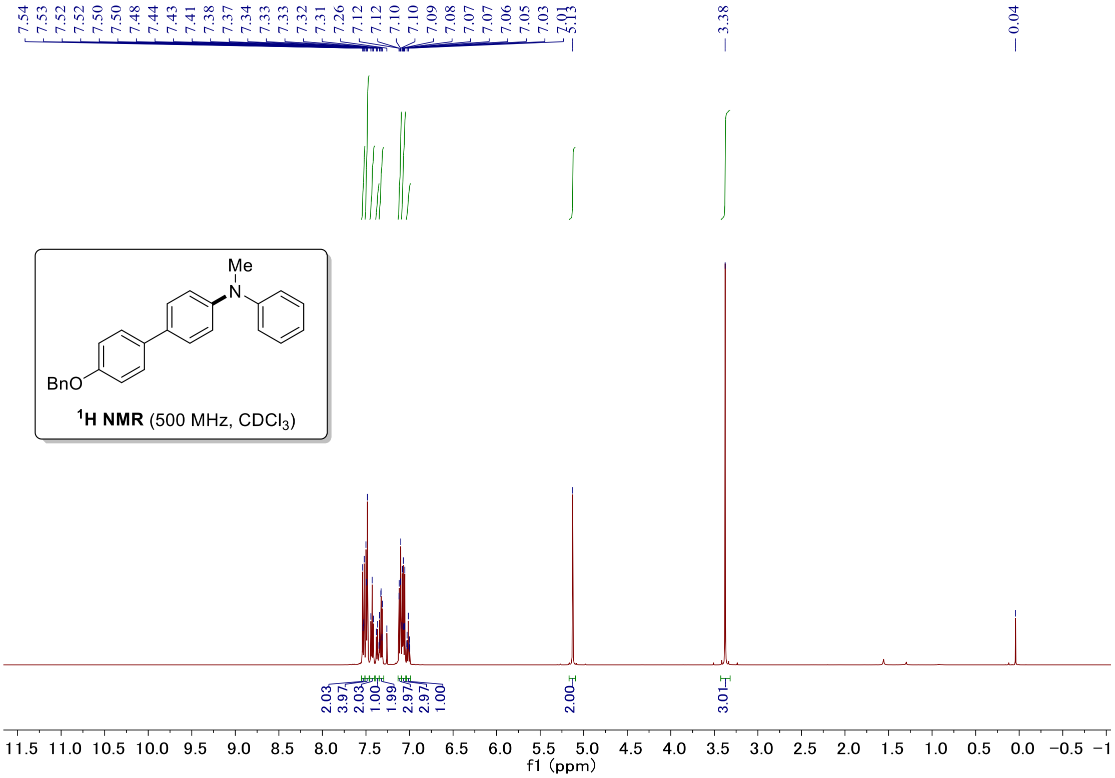

Supplementary Figure 118. ^{1}H NMR (500 MHz, $CDCl_3$, 25 °C) of compound **4na**

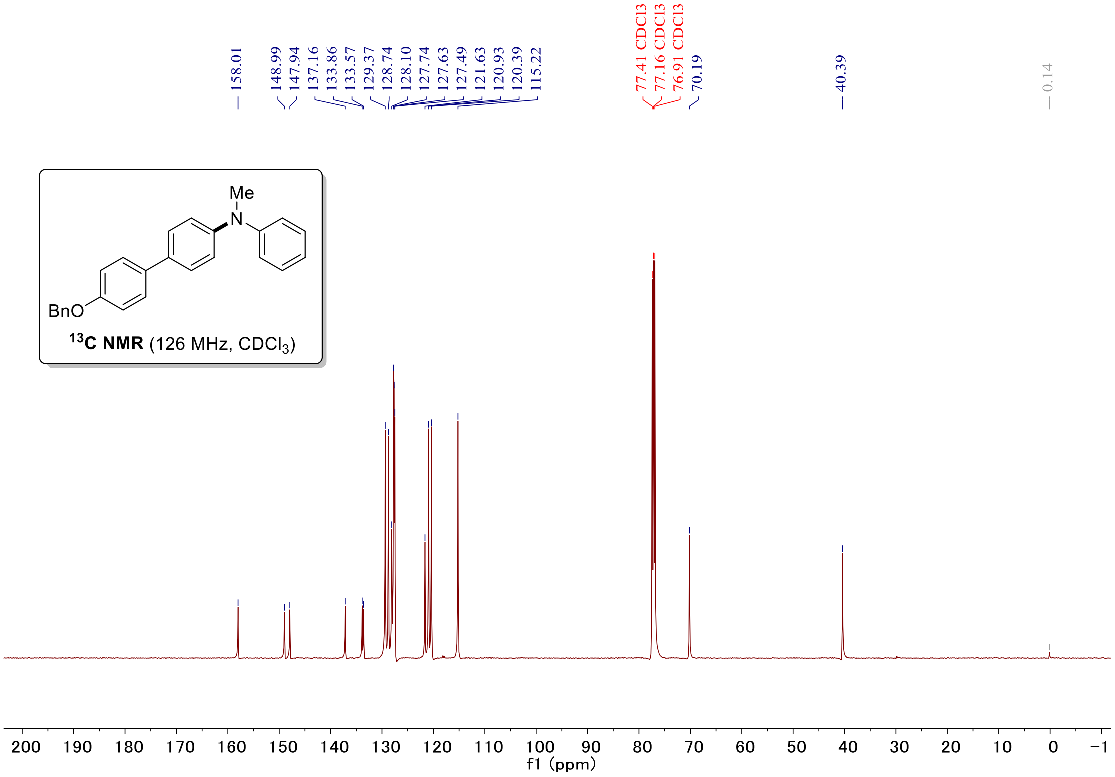

Supplementary Figure 119. ^{13}C NMR (126 MHz, $CDCl_3$, 25 °C) of compound **4na**

4'-Chloro-*N*-methyl-*N*-phenyl-[1,1'-biphenyl]-4-amine (4oa)

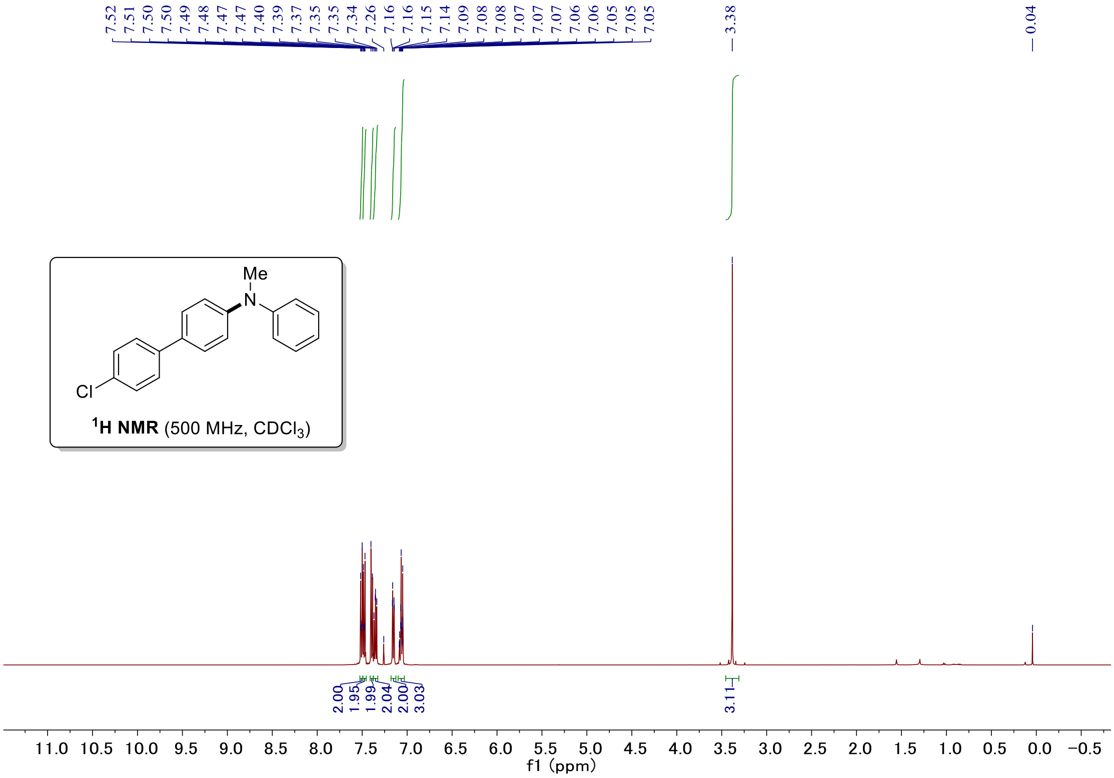

Supplementary Figure 120. ^{1}H NMR (500 MHz, $CDCl_3$, 25 °C) of compound **4oa**

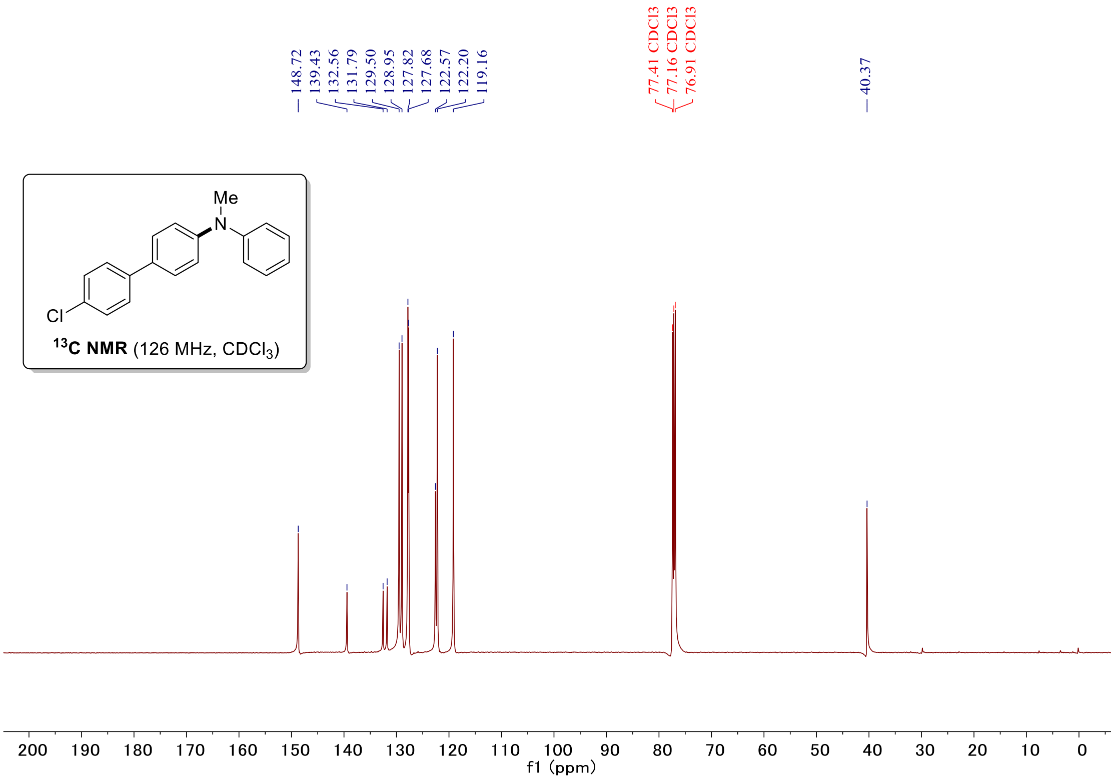

Supplementary Figure 121. ^{13}C NMR (126 MHz, $CDCl_3$, 25 °C) of compound **4oa**

4'-Bromo-*N*-methyl-*N*-phenyl-[1,1'-biphenyl]-4-amine (4pa)

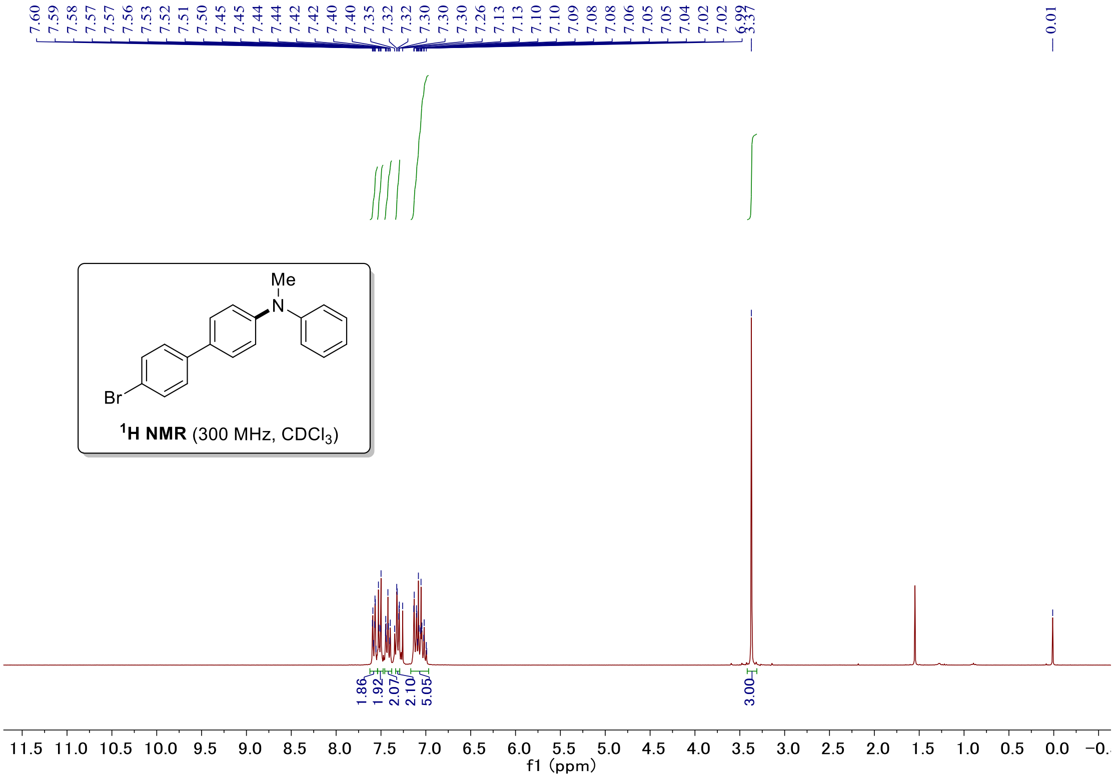

Supplementary Figure 122. ^{1}H NMR (300 MHz, $CDCl_3$, 25 °C) of compound **4pa**

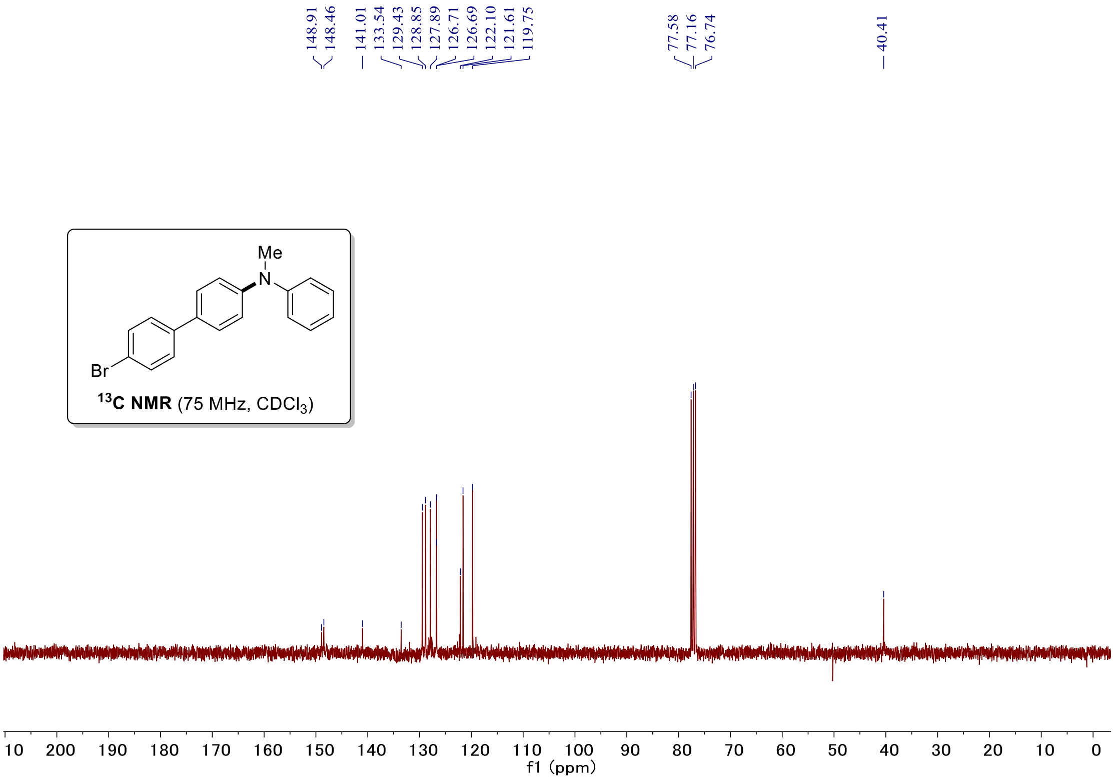

Supplementary Figure 123. ^{13}C NMR (75 MHz, $CDCl_3$, 25 °C) of compound **4pa**

4'-(Methyl(phenyl)amino)-[1,1'-biphenyl]-4-carbonitrile (4qa)

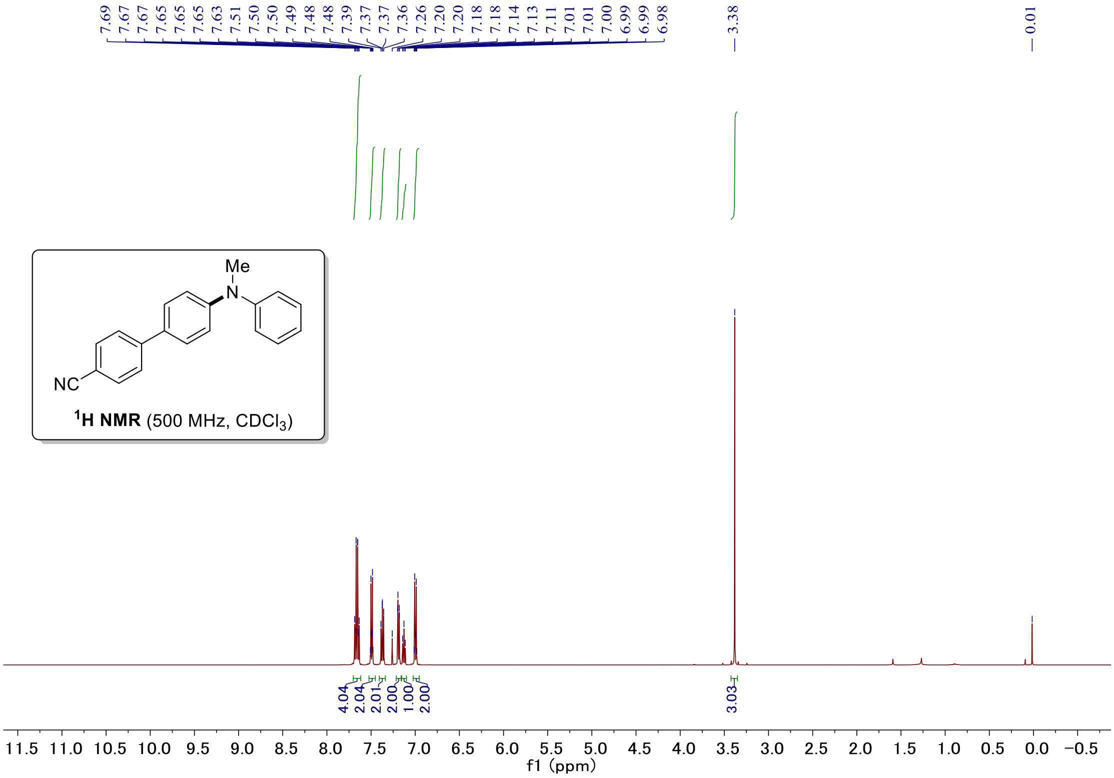

Supplementary Figure 124. ^{1}H NMR (500 MHz, $CDCl_3$, 25 °C) of compound **4qa**

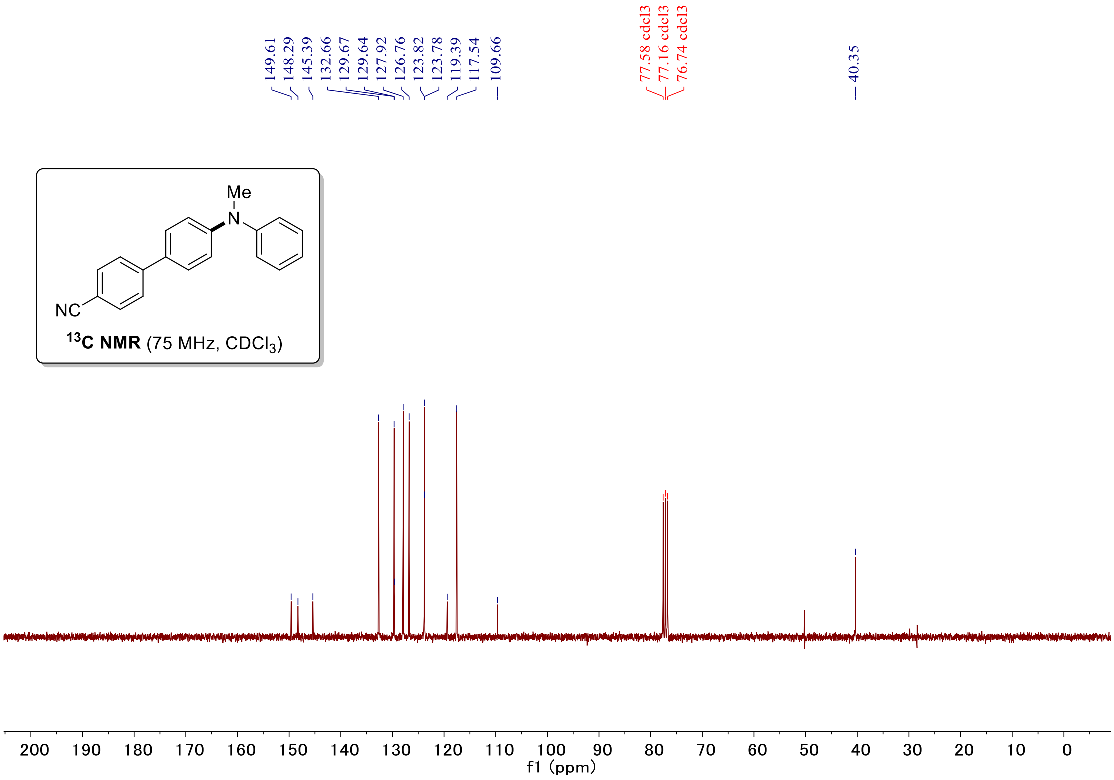

Supplementary Figure 125. ^{13}C NMR (75 MHz, $CDCl_3$, 25 °C) of compound **4qa**

N-Methyl-N-phenyl-4'-(trifluoromethyl)-[1,1'-biphenyl]-4-amine (4ra)

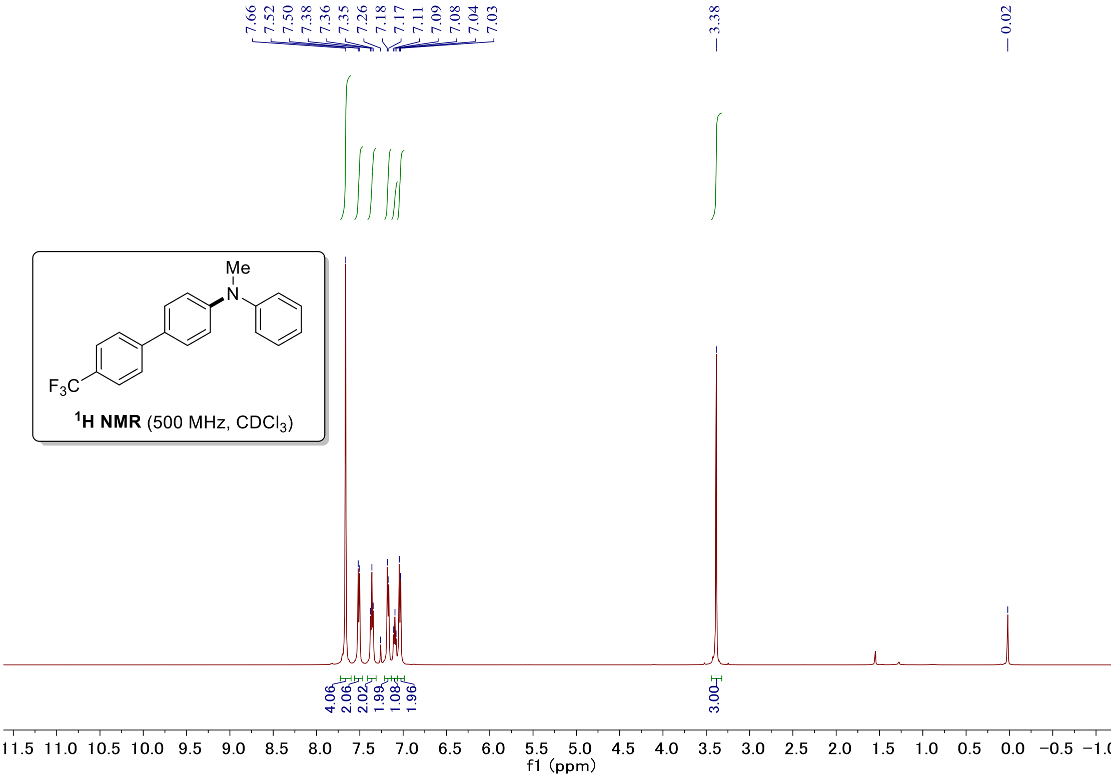

Supplementary Figure 126. ^{1}H NMR (500 MHz, $CDCl_3$, 25 °C) of compound **4ra**

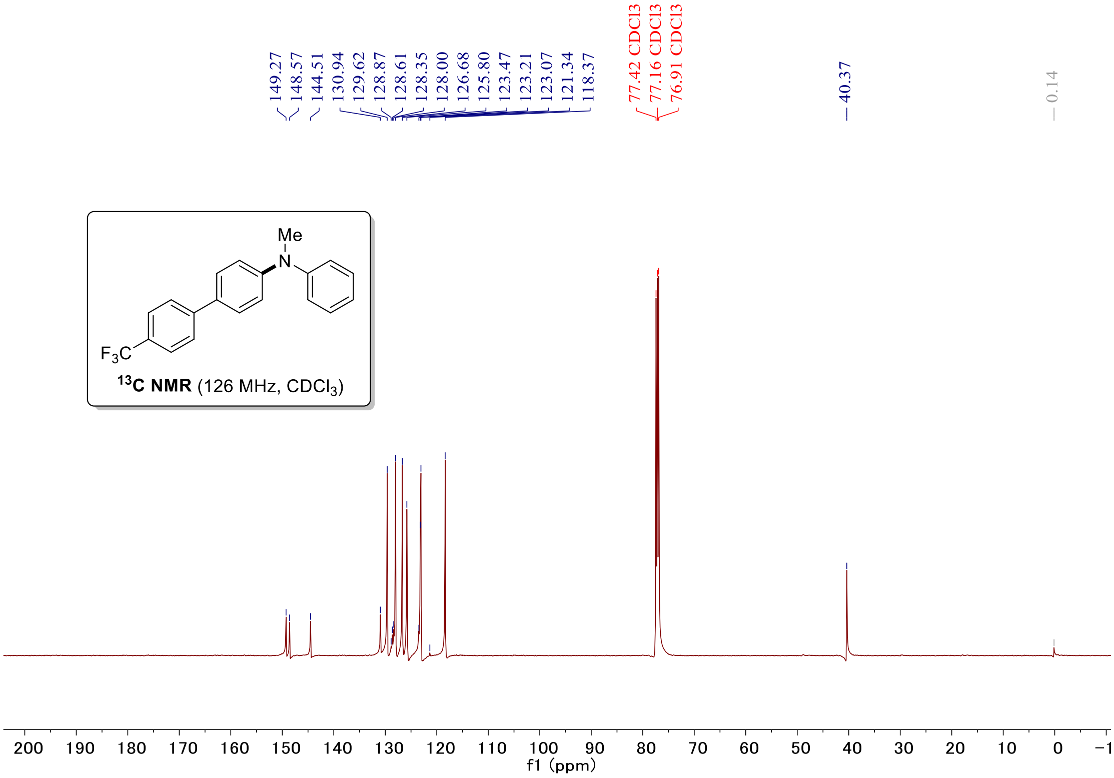

Supplementary Figure 127. ^{13}C NMR (126 MHz, $CDCl_3$, 25 °C) of compound **4ra**

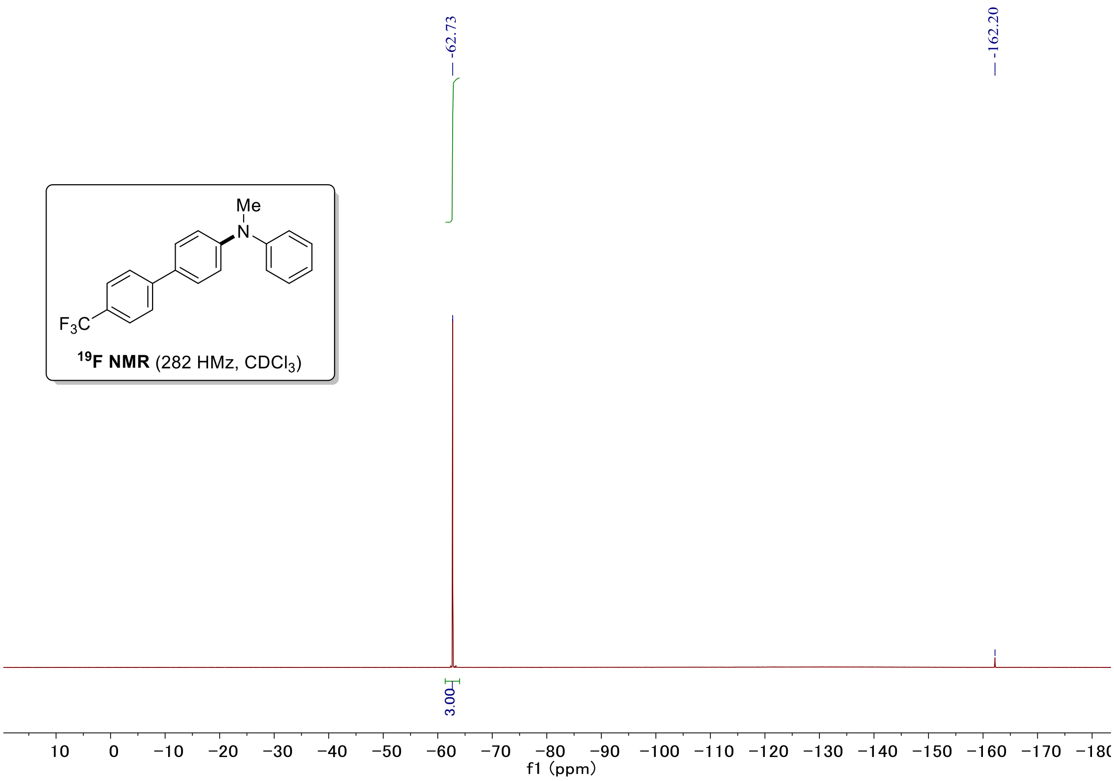

Supplementary Figure 128. ^{19}F NMR (282 MHz, $CDCl_3$, 25 °C) of compound **4ra**

N-Methyl-*N*-phenyl-3'-(trifluoromethyl)-[1,1'-biphenyl]-4-amine (4sa)

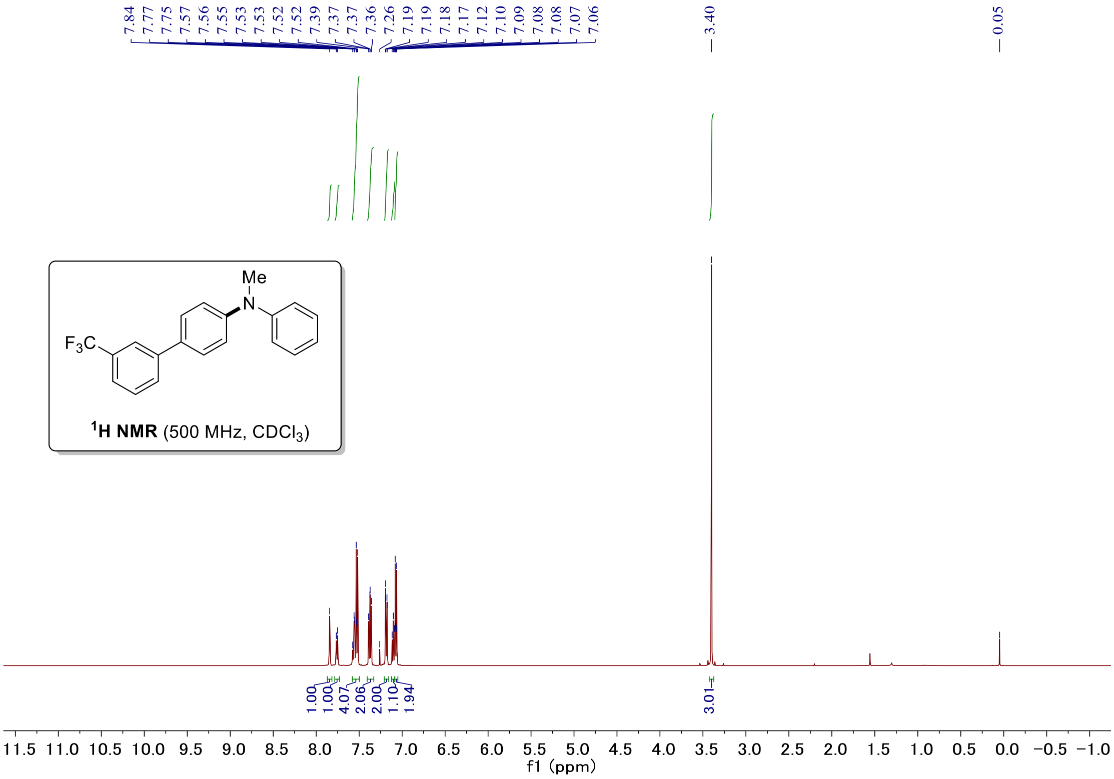

Supplementary Figure 129. ^{1}H NMR (500 MHz, $CDCl_3$, 25 °C) of compound **4sa**

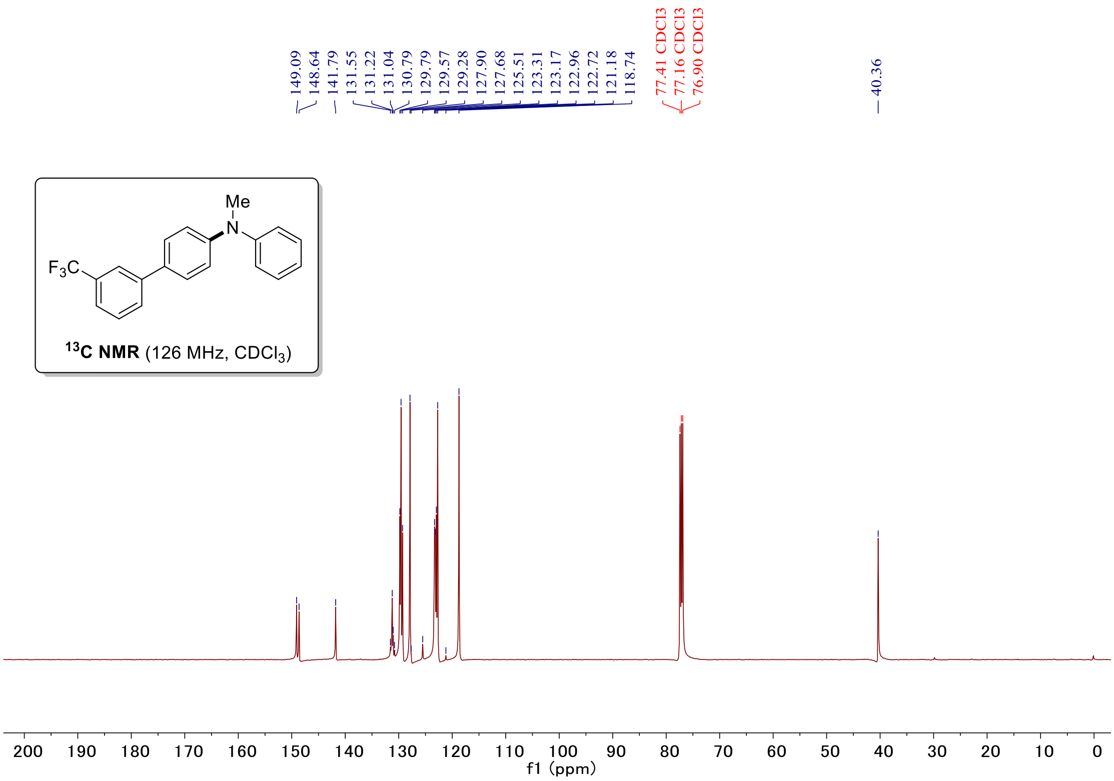

Supplementary Figure 130. ^{13}C NMR (126 MHz, $CDCl_3$, 25 °C) of compound **4sa**

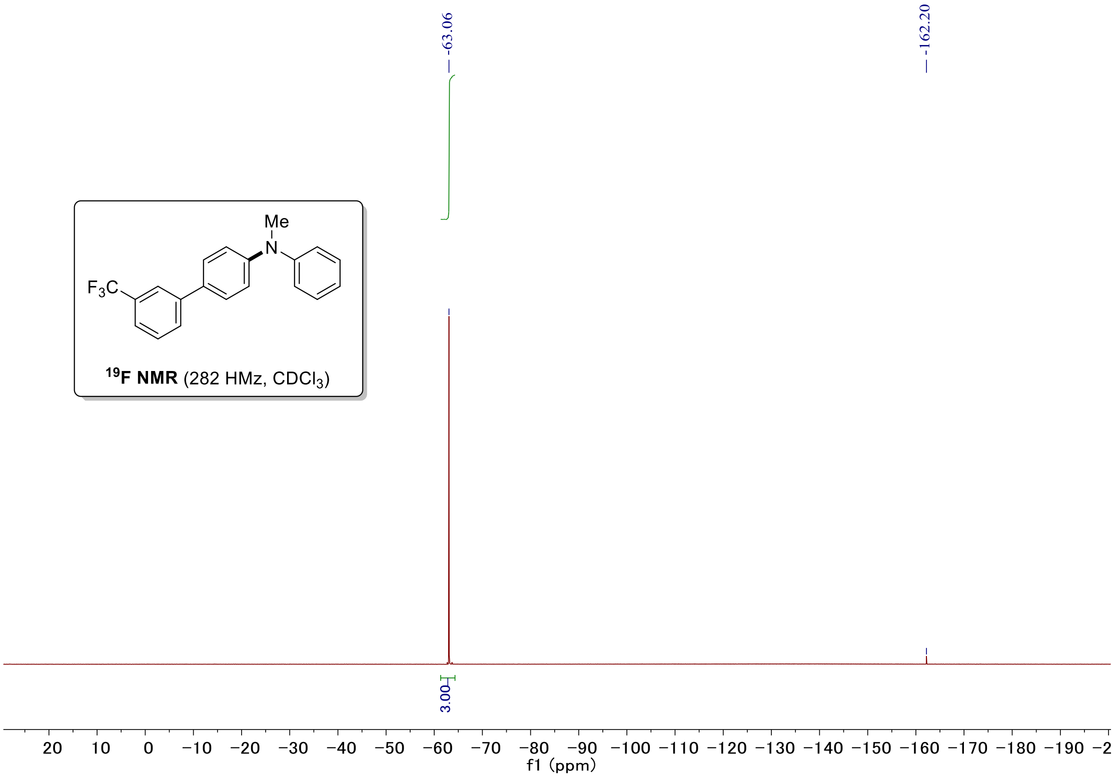

Supplementary Figure 131. ^{19}F NMR (282 MHz, $CDCl_3$, 25 °C) of compound **4sa**

***N*-Methyl-*N*-phenyl-3',5'-bis(trifluoromethyl)-[1,1'-biphenyl]-4-amine (4ta)**

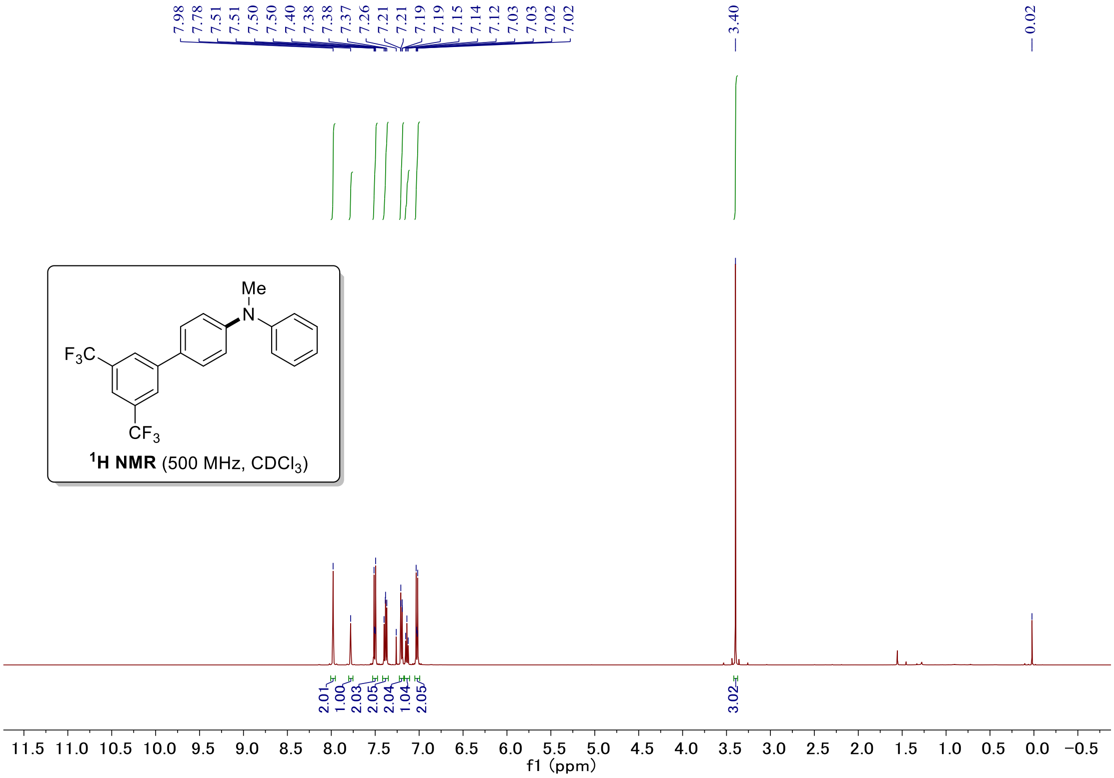

Supplementary Figure 132. ^{1}H NMR (500 MHz, $CDCl_3$, 25 °C) of compound **4ta**

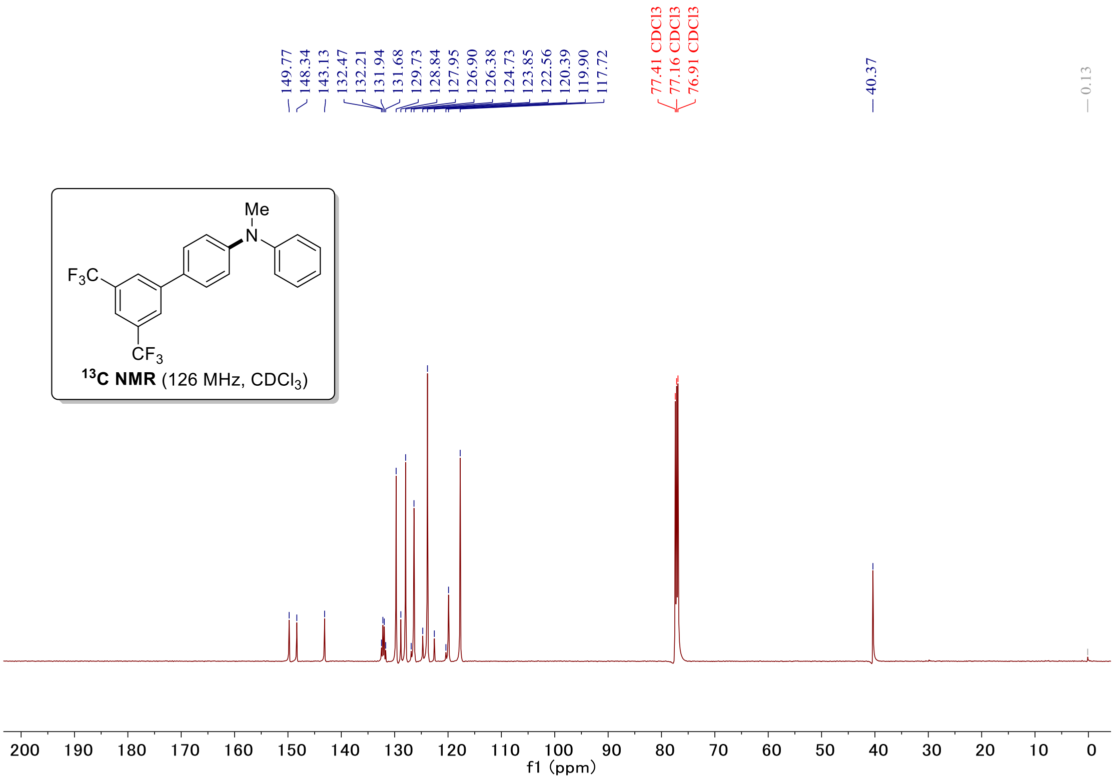

Supplementary Figure 133. ^{13}C NMR (126 MHz, $CDCl_3$, 25 °C) of compound **4ta**

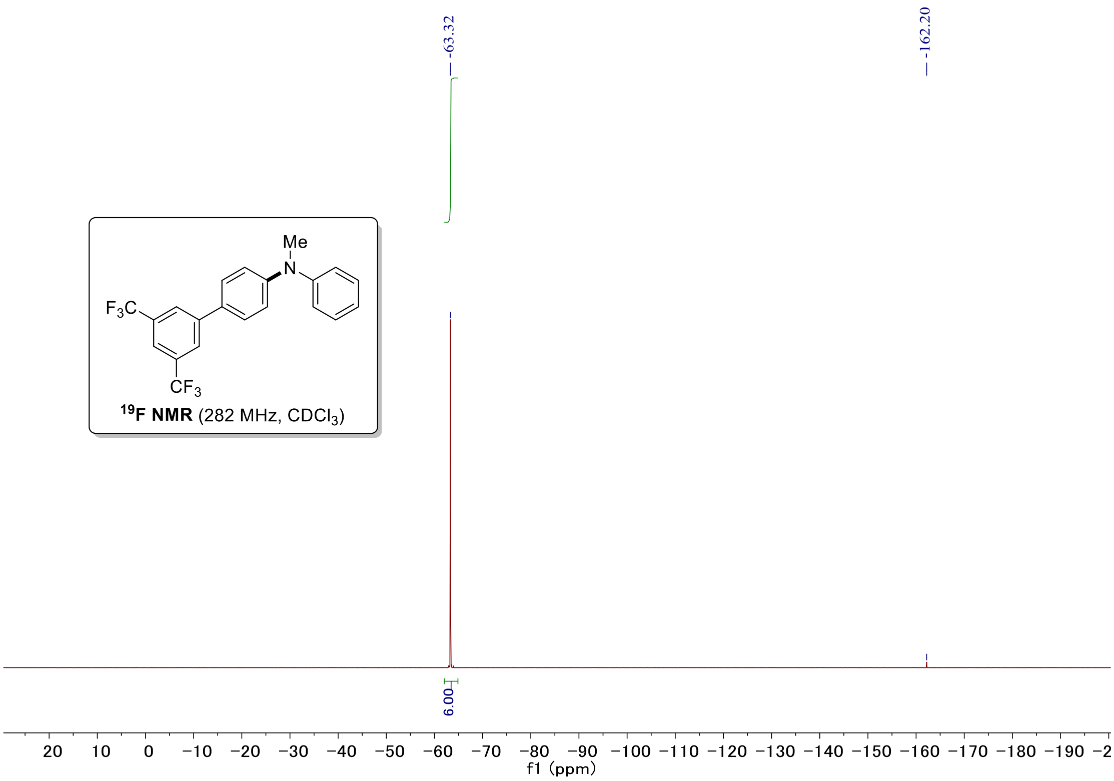

Supplementary Figure 134. ^{19}F NMR (282 MHz, $CDCl_3$, 25 °C) of compound **4ta**

4-(Benzo[*d*][1,3]dioxol-5-yl)-*N*-methyl-*N*-phenylaniline (4ua)

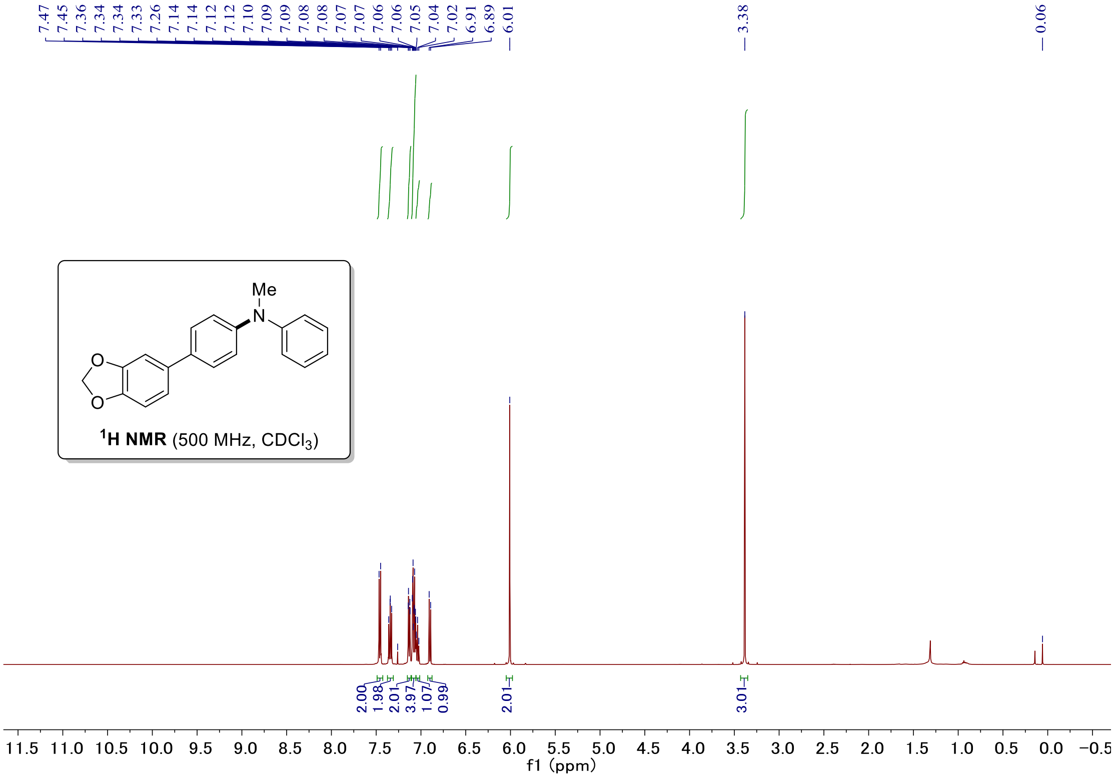

Supplementary Figure 135. ^{1}H NMR (500 MHz, $CDCl_3$, 25 °C) of compound **4ua**

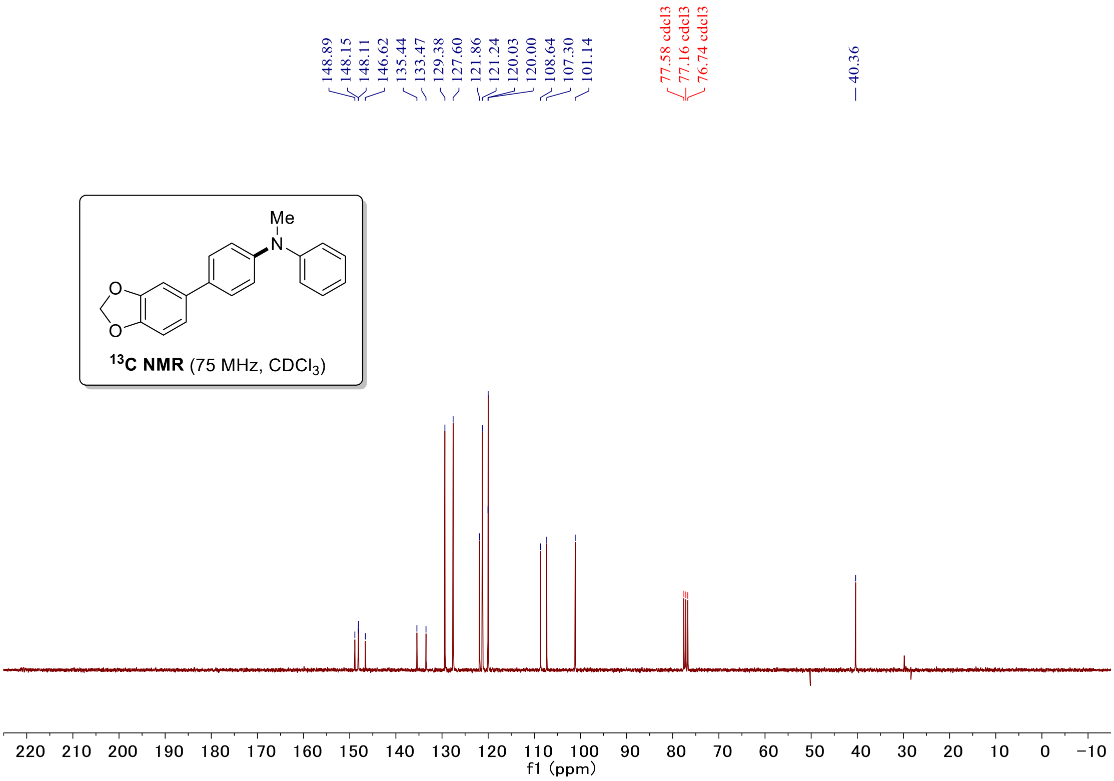

Supplementary Figure 136. ^{13}C NMR (75 MHz, $CDCl_3$, 25 °C) of compound **4ua**

***N*-Methyl-*N*,*6*-diphenylpyridin-3-amine (4va)**

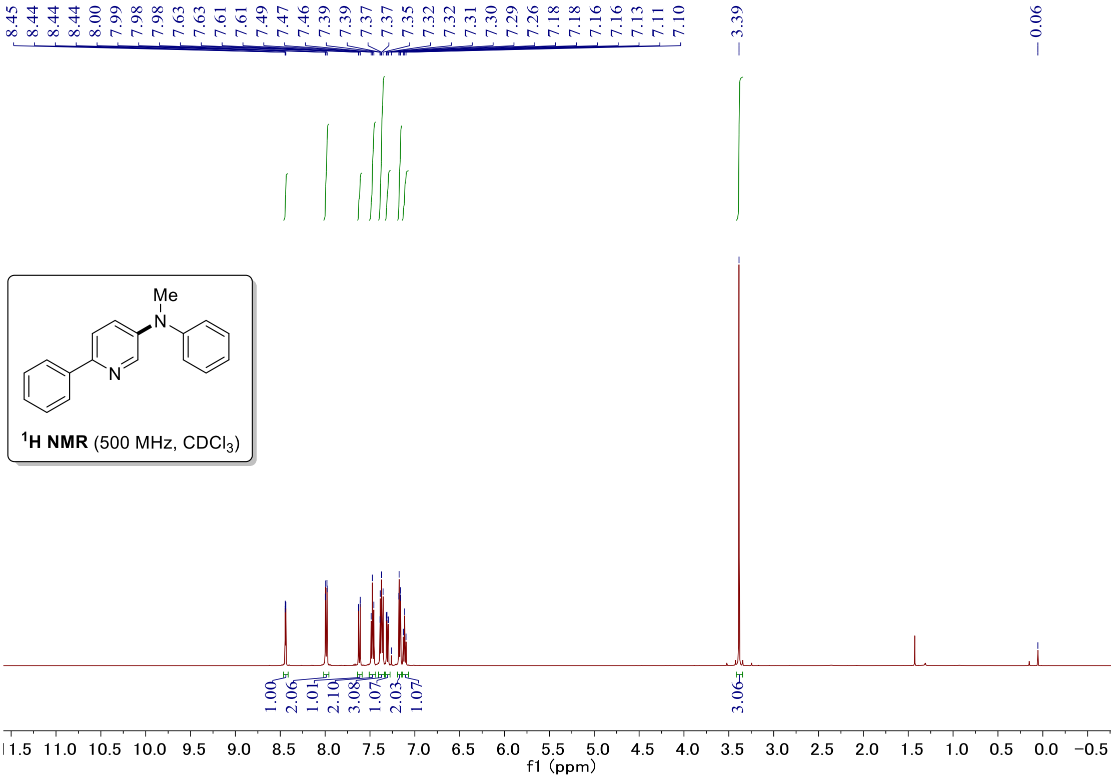

Supplementary Figure 137. ^{1}H NMR (500 MHz, $CDCl_3$, 25 °C) of compound **4va**

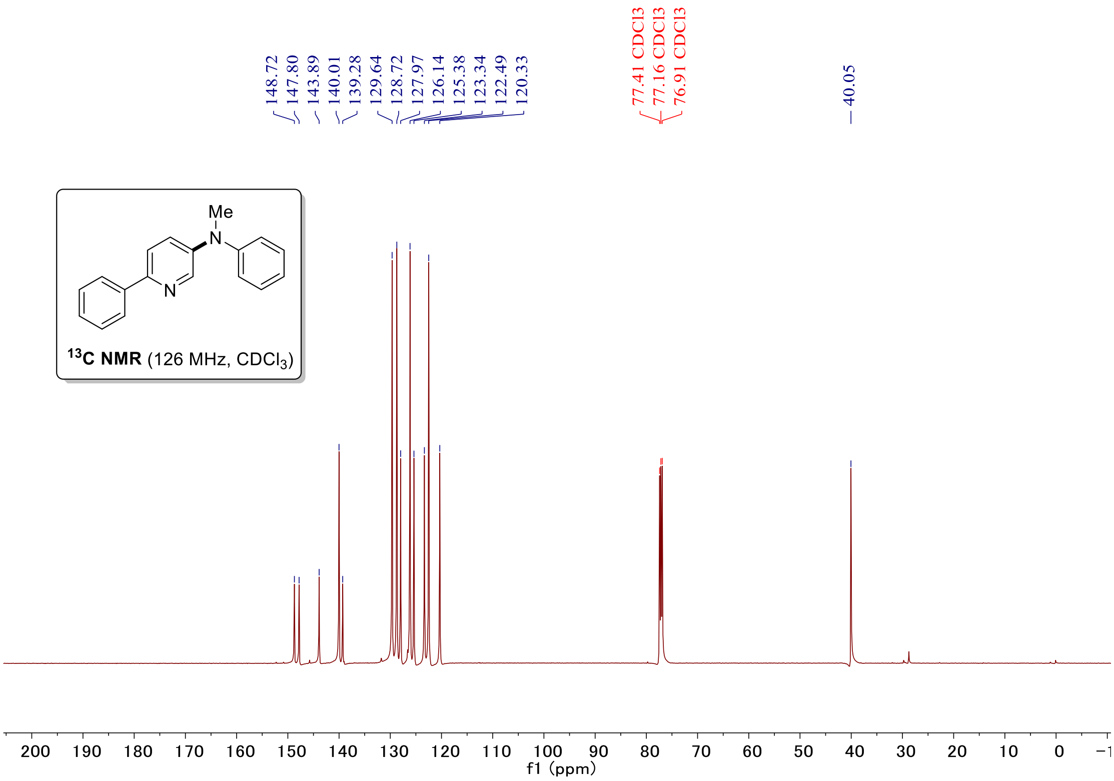

Supplementary Figure 138. ^{13}C NMR (126 MHz, $CDCl_3$, 25 °C) of compound **4va**

***N*-Methyl-*N*-phenyl-4-(pyridin-4-yl)aniline (4wa)**

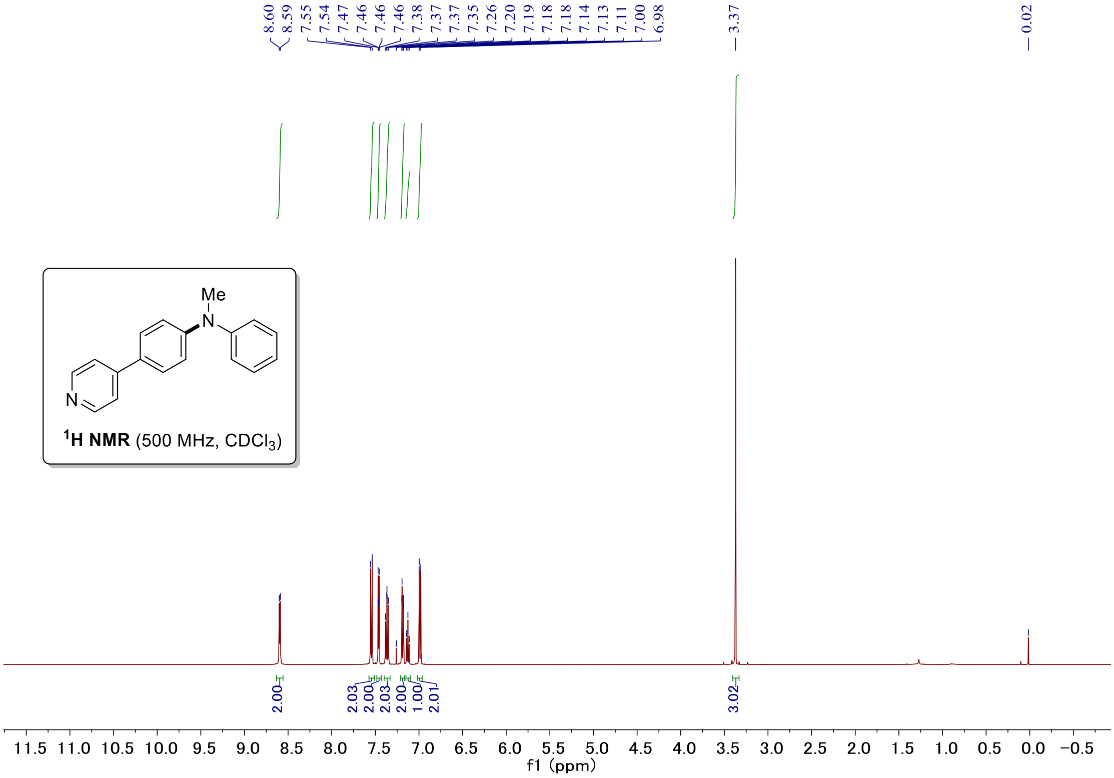

Supplementary Figure 139. ^{1}H NMR (500 MHz, $CDCl_3$, 25 °C) of compound **4wa**

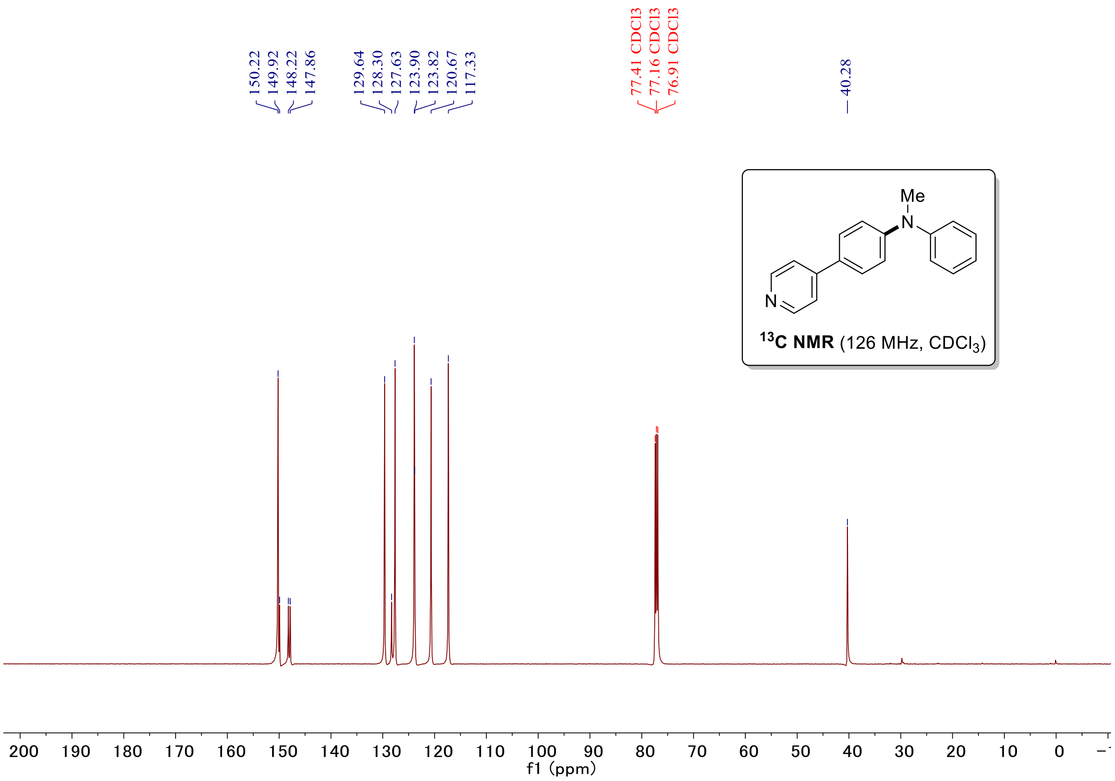

Supplementary Figure 140. ^{13}C NMR (126 MHz, $CDCl_3$, 25 °C) of compound **4wa**

N-Methyl-*N*,2-diphenylpyridin-4-amine (4xa)

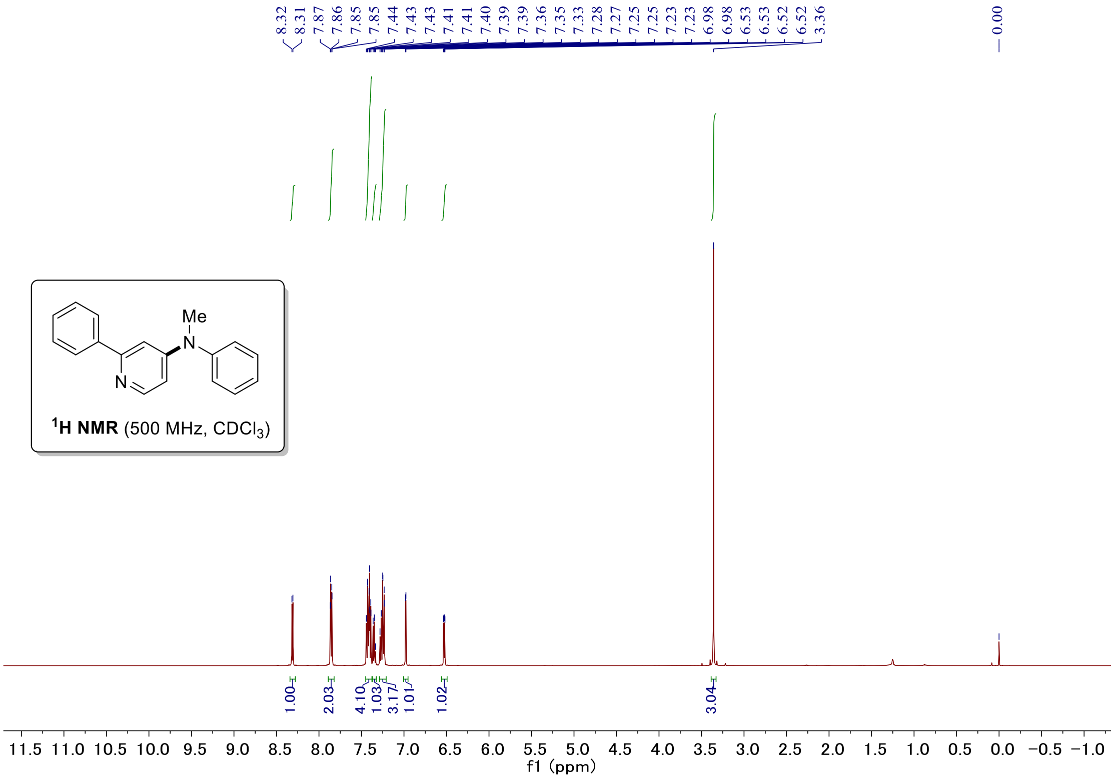

Supplementary Figure 141. ^{1}H NMR (500 MHz, $CDCl_3$, 25 °C) of compound **4xa**

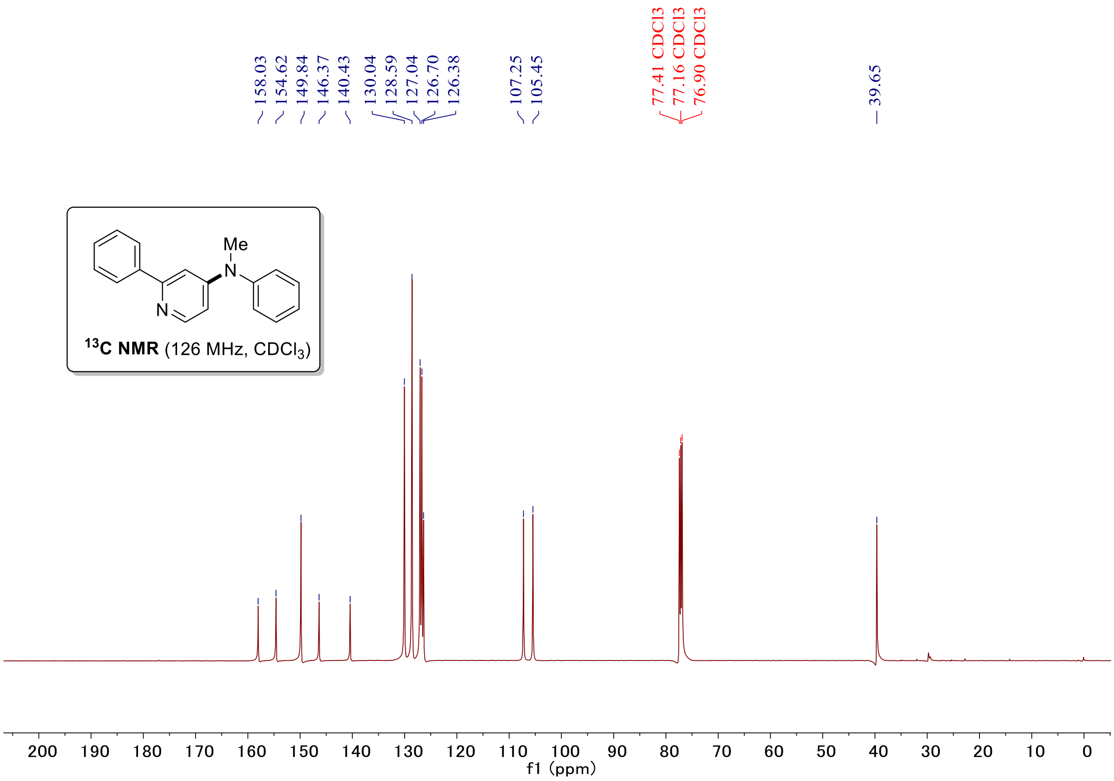

Supplementary Figure 142. ^{13}C NMR (126 MHz, $CDCl_3$, 25 °C) of compound **4xa**

***N*-Methyl-*N*,5-diphenylpyridin-2-amine (4ya)**

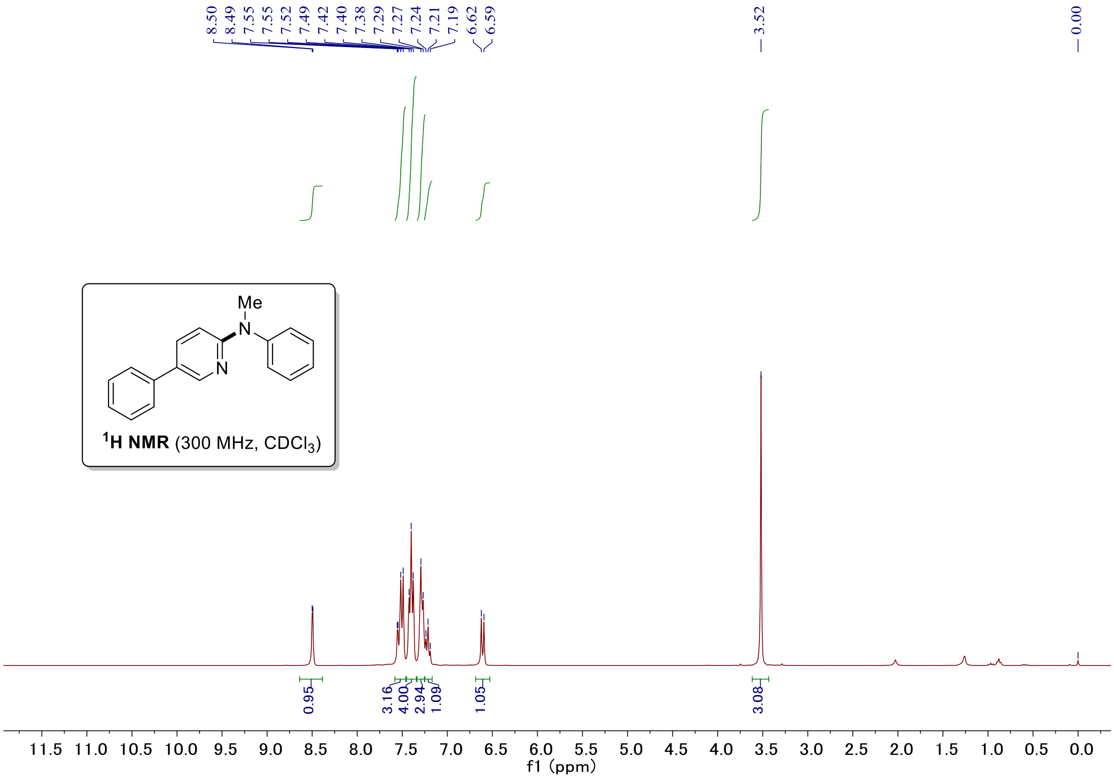

Supplementary Figure 143. ^{1}H NMR (300 MHz, $CDCl_3$, 25 °C) of compound **4ya**

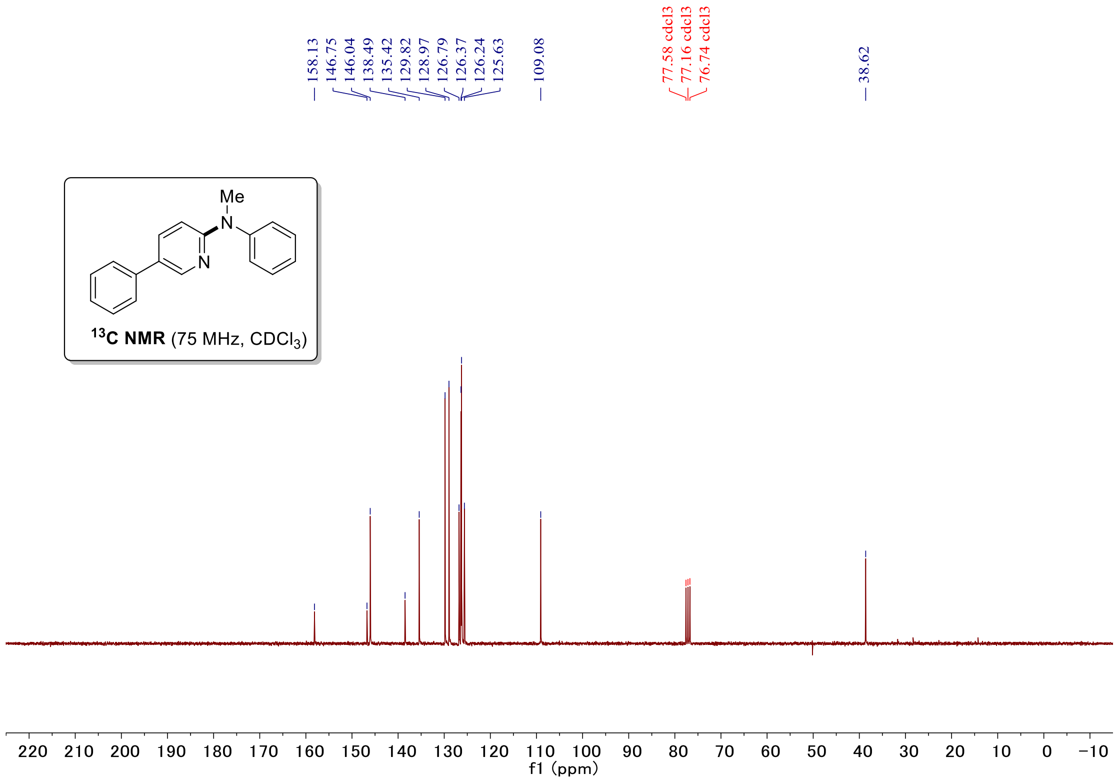

Supplementary Figure 144. ^{13}C NMR (75 MHz, $CDCl_3$, 25 °C) of compound **4ya**

N-Methyl-*N*-phenyl-4-(1*H*-pyrrol-1-yl)aniline (4za)

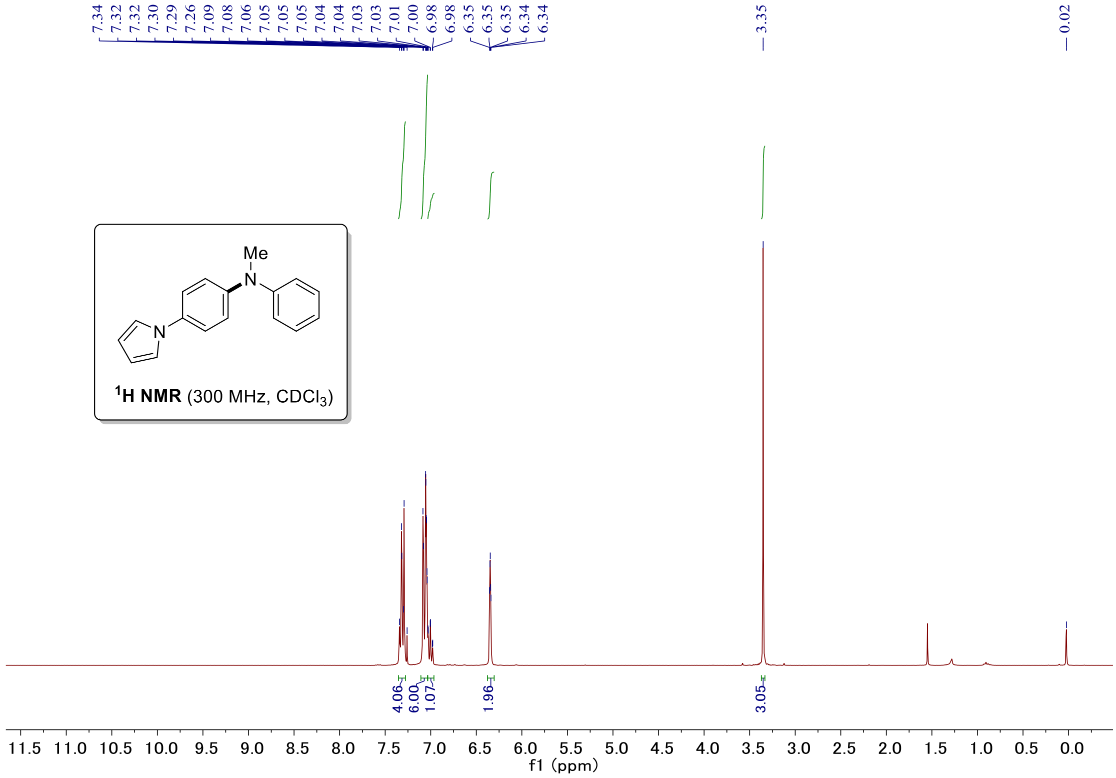

Supplementary Figure 145. ^{1}H NMR (300 MHz, $CDCl_3$, 25 °C) of compound **4za**

N,*1*-Dimethyl-*N*-phenyl-1*H*-indol-6-amine (4aaa)

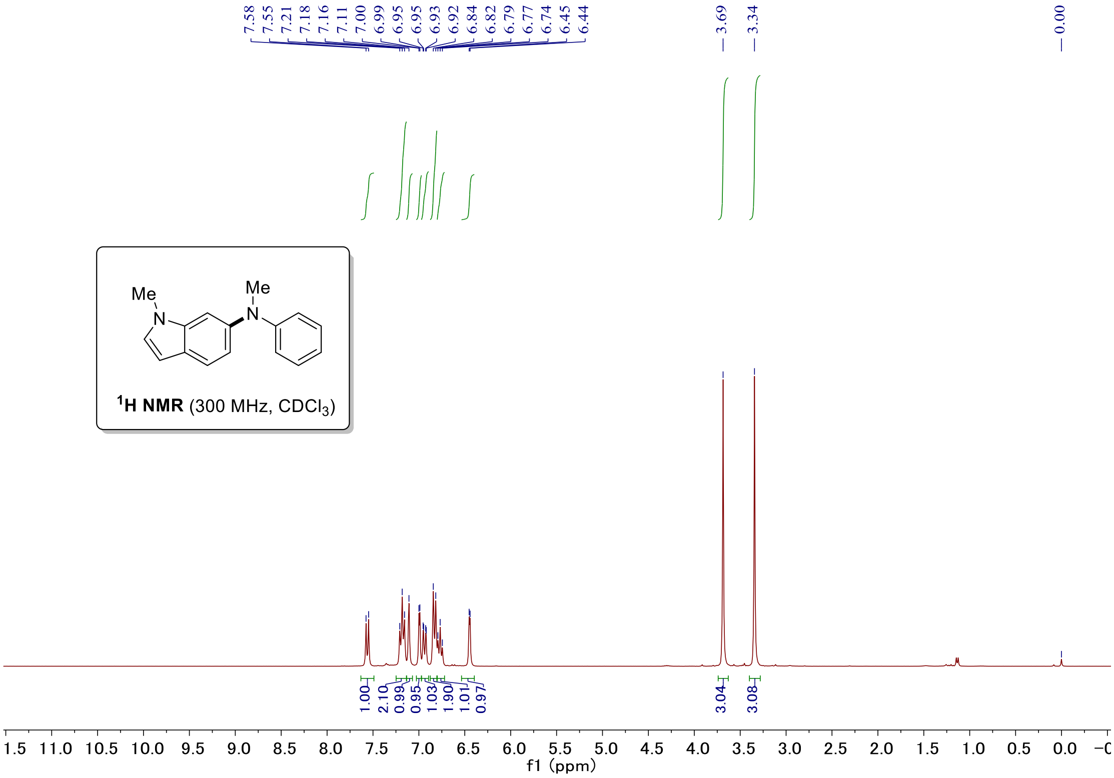

Supplementary Figure 146. ^{1}H NMR (300 MHz, $CDCl_3$, 25 °C) of compound **4aaa**

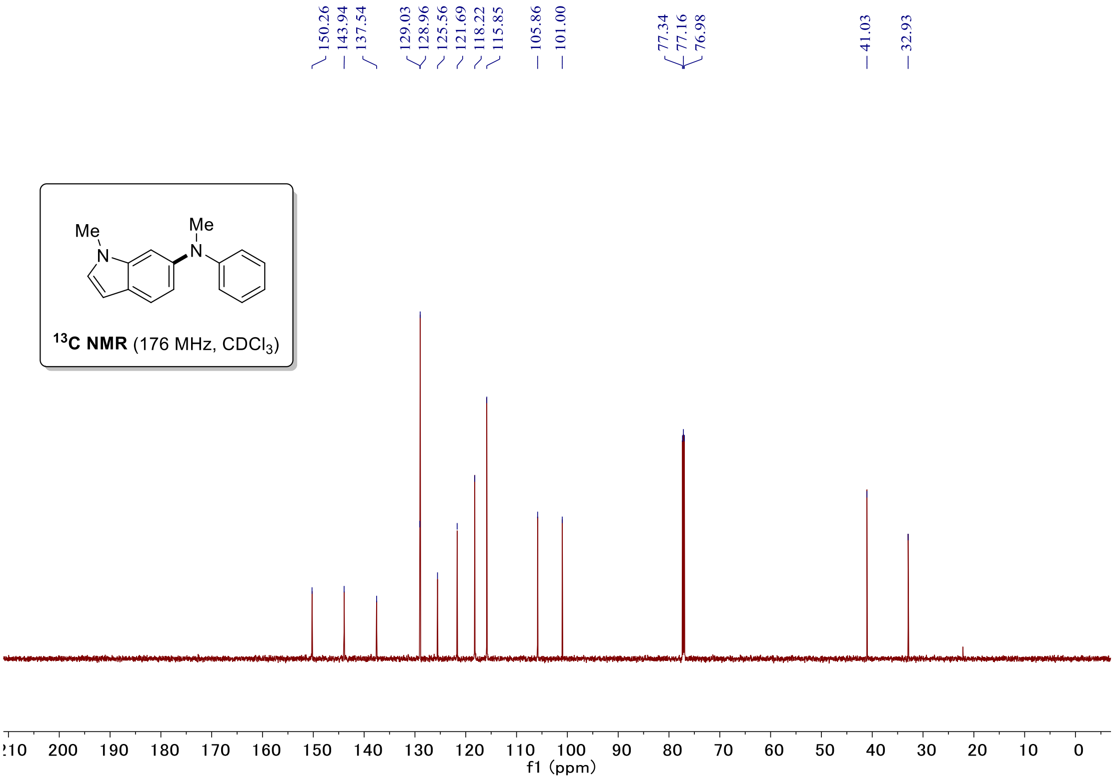

Supplementary Figure 147. ^{13}C NMR (176 MHz, $CDCl_3$, 25 °C) of compound **4aaa**

***N*-Methyl-4-(1-methyl-1*H*-indol-2-yl)-*N*-phenylaniline (4aba)**

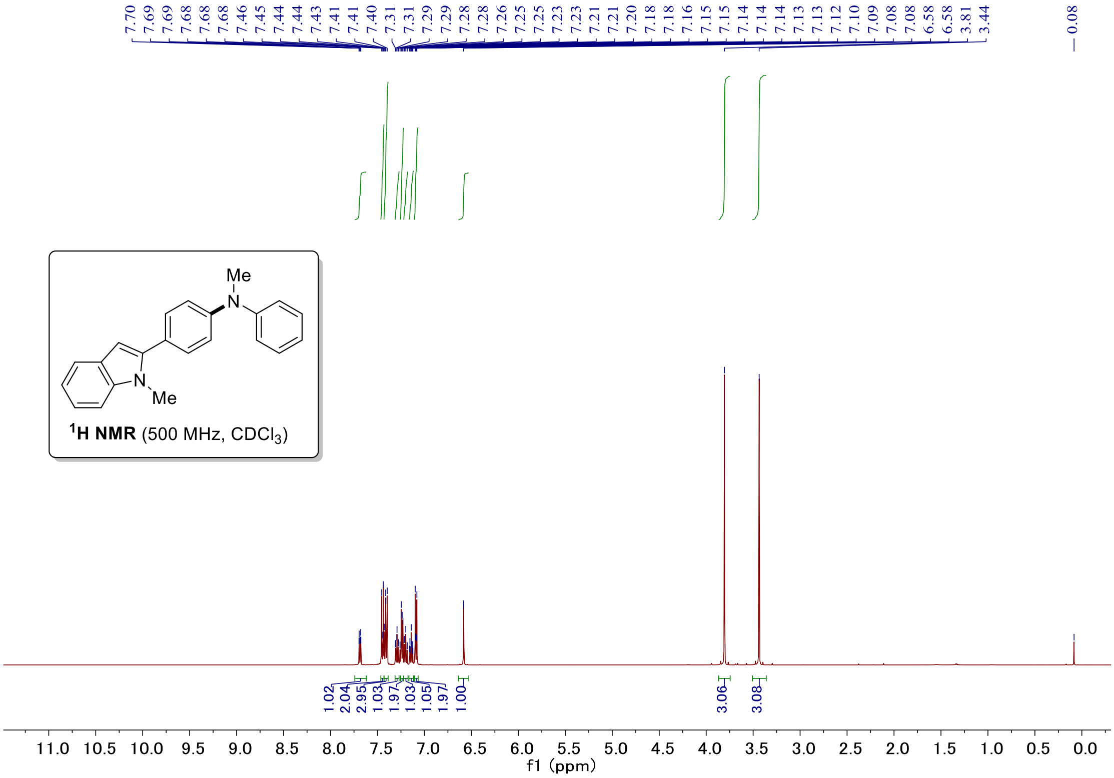

Supplementary Figure 148. ^{1}H NMR (500 MHz, CDCl$_3$, 25 °C) of compound **4aba**

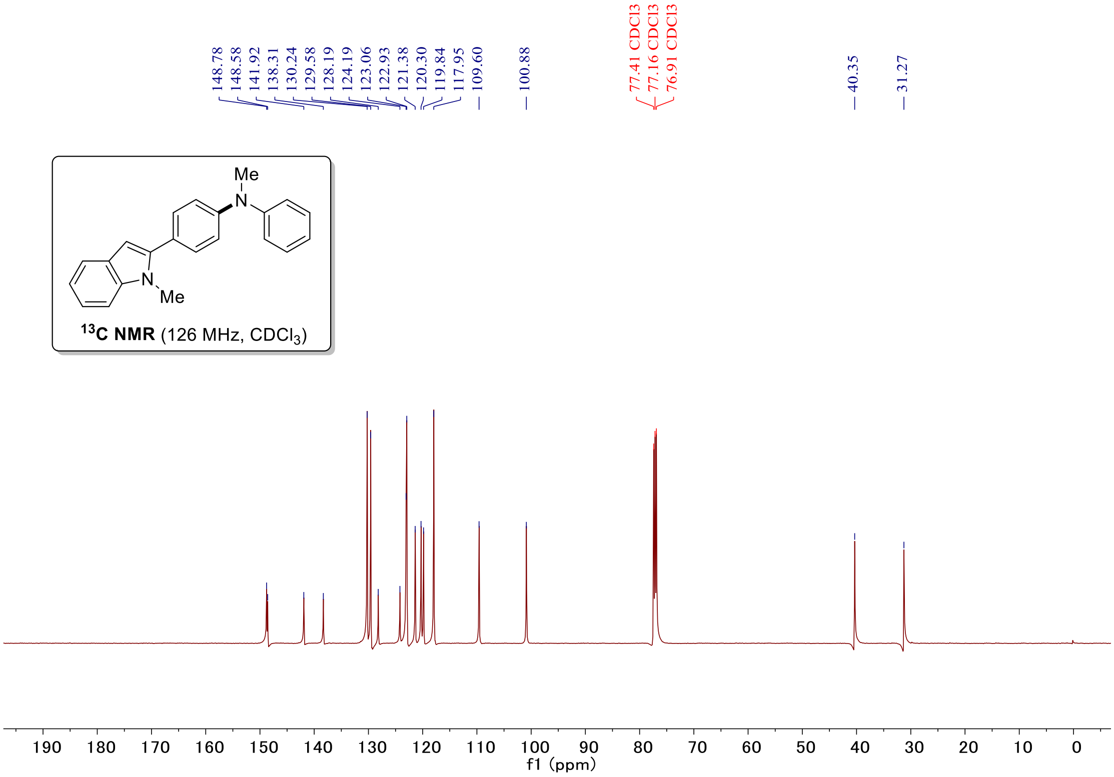

Supplementary Figure 149. ^{13}C NMR (126 MHz, CDCl$_3$, 25 °C) of compound **4aba**

4-(Benzofuran-2-yl)-*N*-methyl-*N*-phenylaniline (4aca)

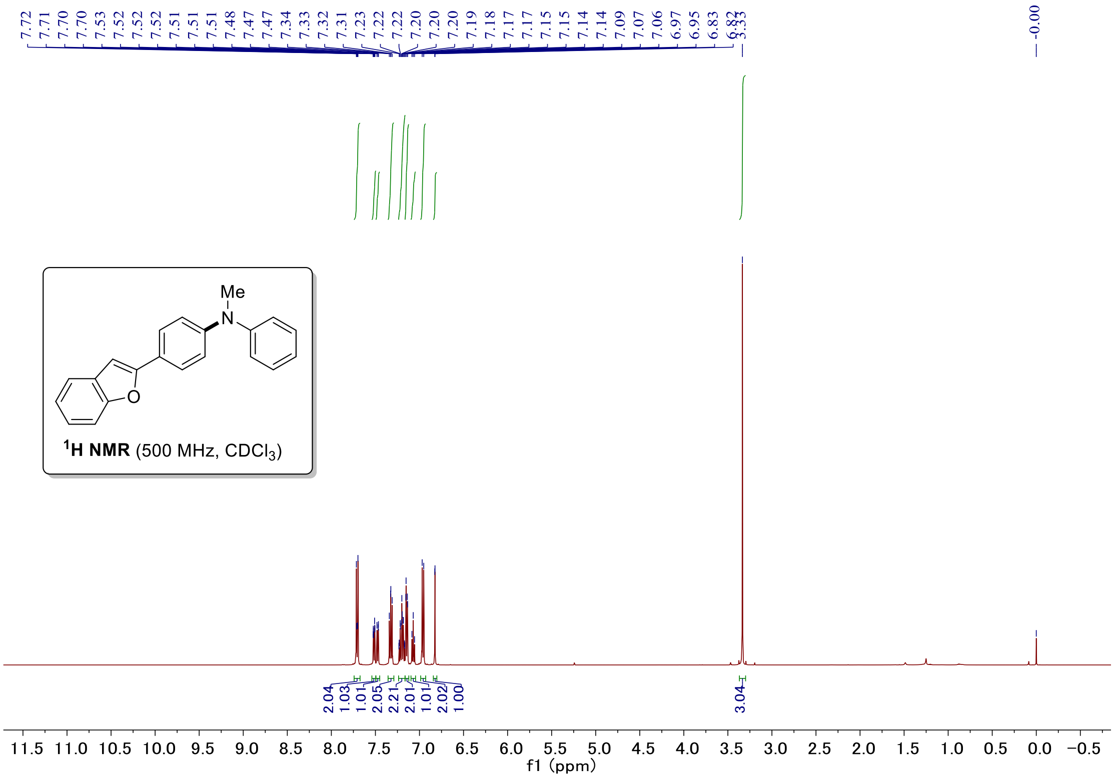

Supplementary Figure 150. ^{1}H NMR (500 MHz, $CDCl_3$, 25 °C) of compound **4aca**

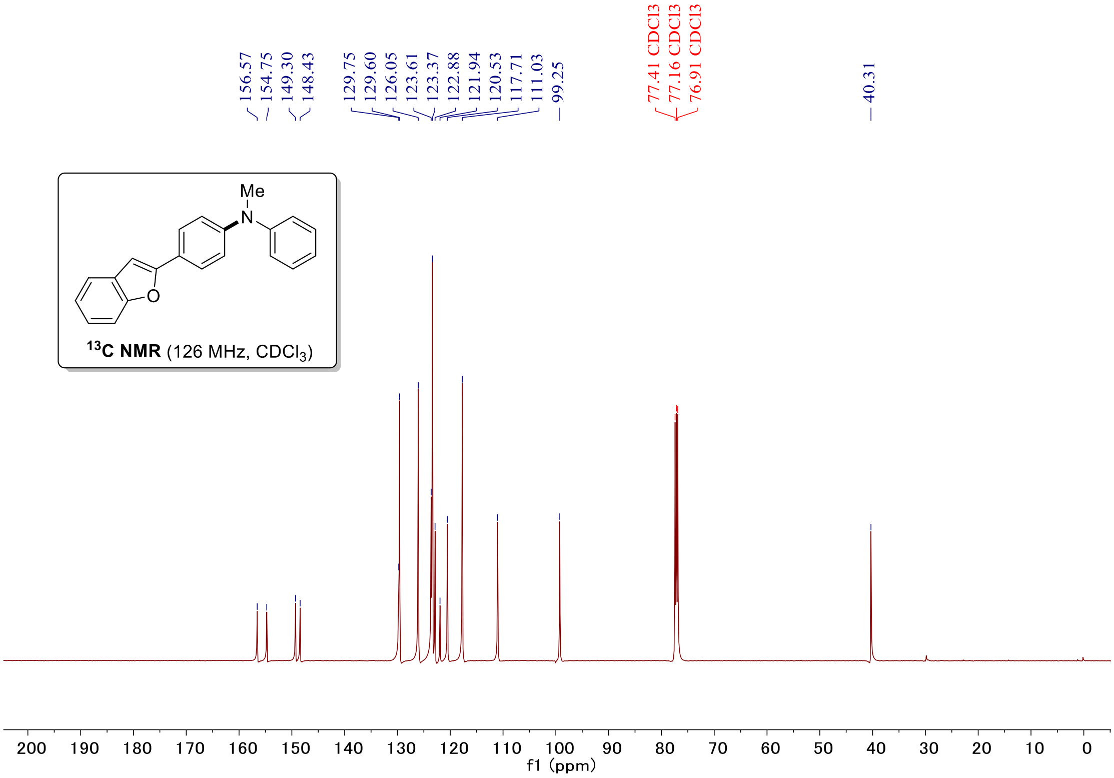

Supplementary Figure 151. ^{13}C NMR (126 MHz, $CDCl_3$, 25 °C) of compound **4aca**

N-Methyl-*N*-(*p*-tolyl)-[1,1'-biphenyl]-4-amine (4ab)

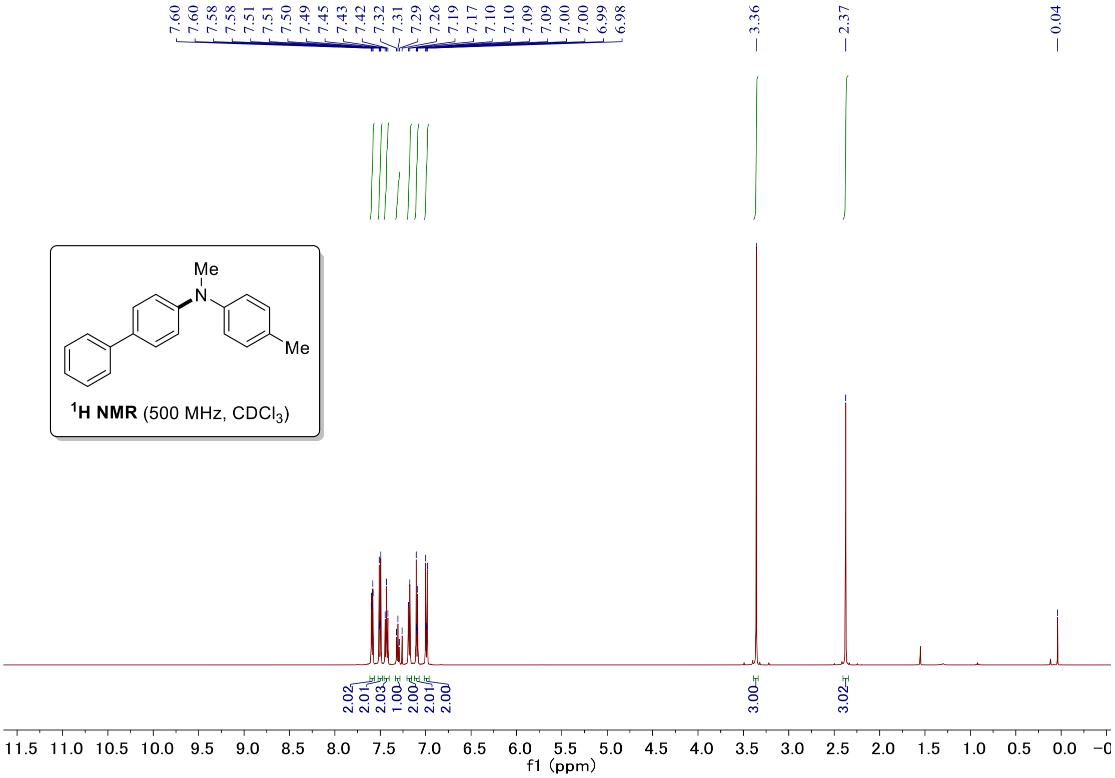

Supplementary Figure 152. ^{1}H NMR (500 MHz, $CDCl_3$, 25 °C) of compound **4ab**

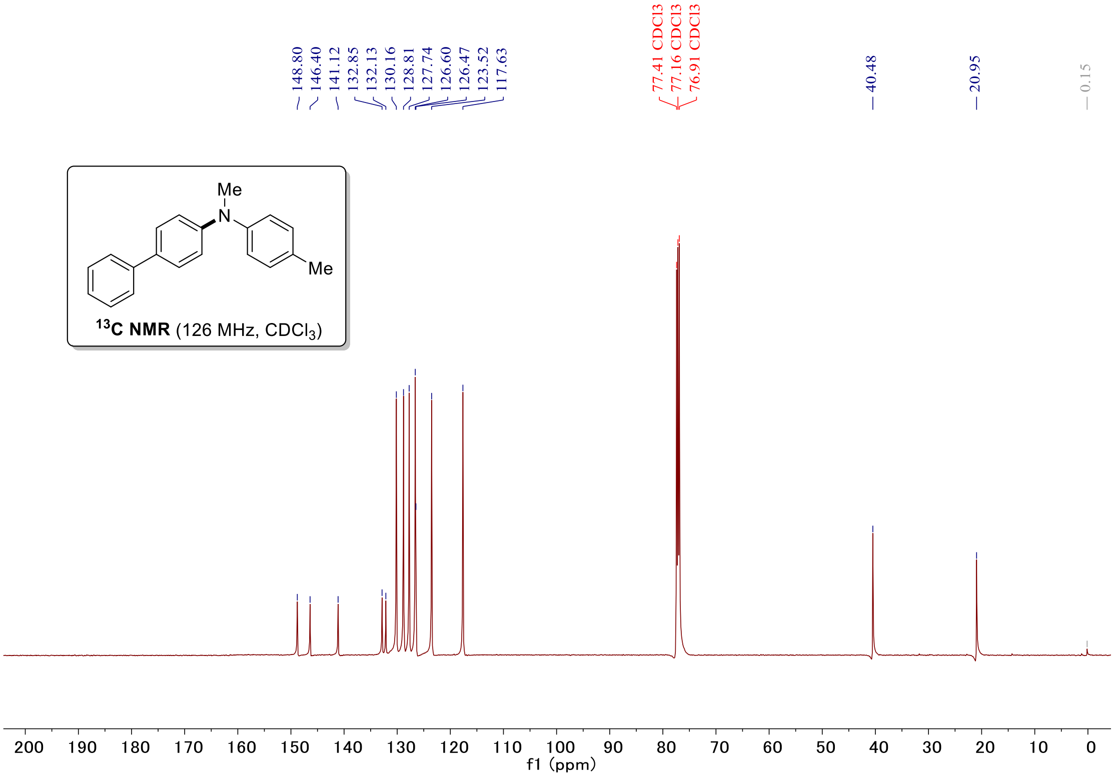

Supplementary Figure 153. ^{13}C NMR (126 MHz, $CDCl_3$, 25 °C) of compound **4ab**

***N*-(3,5-Dimethylphenyl)-*N*-methyl-[1,1'-biphenyl]-4-amine (4ac)**

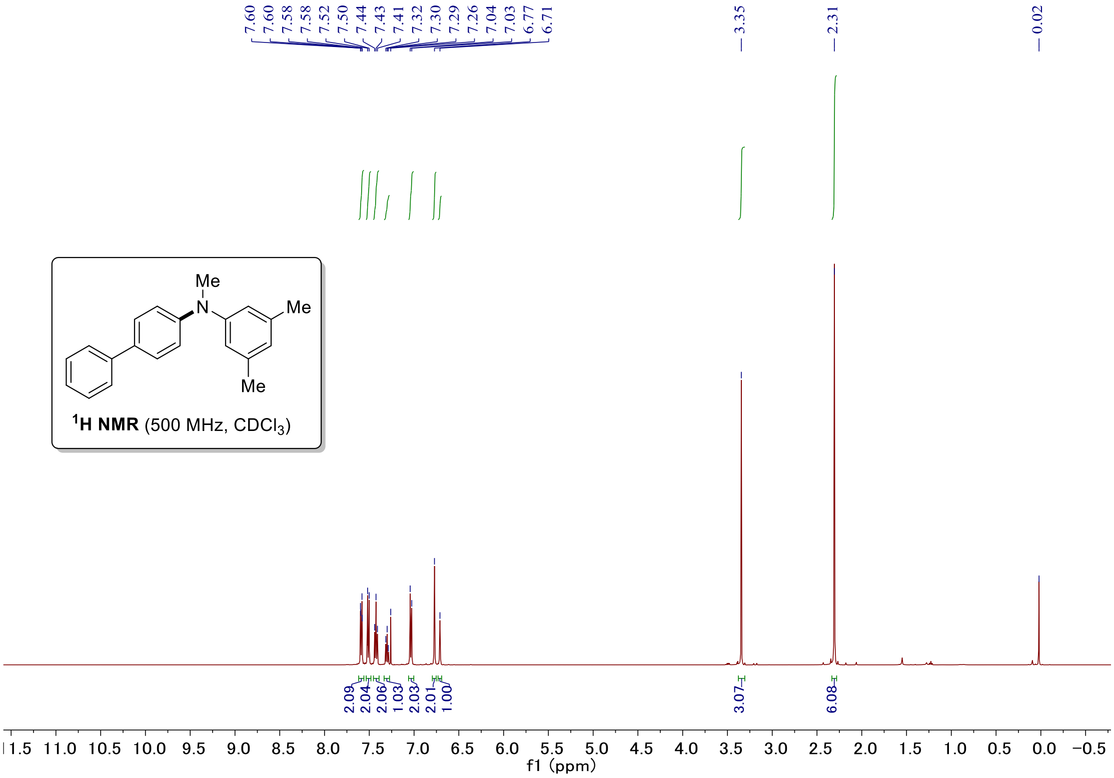

Supplementary Figure 154. ^{1}H NMR (500 MHz, $CDCl_3$, 25 °C) of compound **4ac**

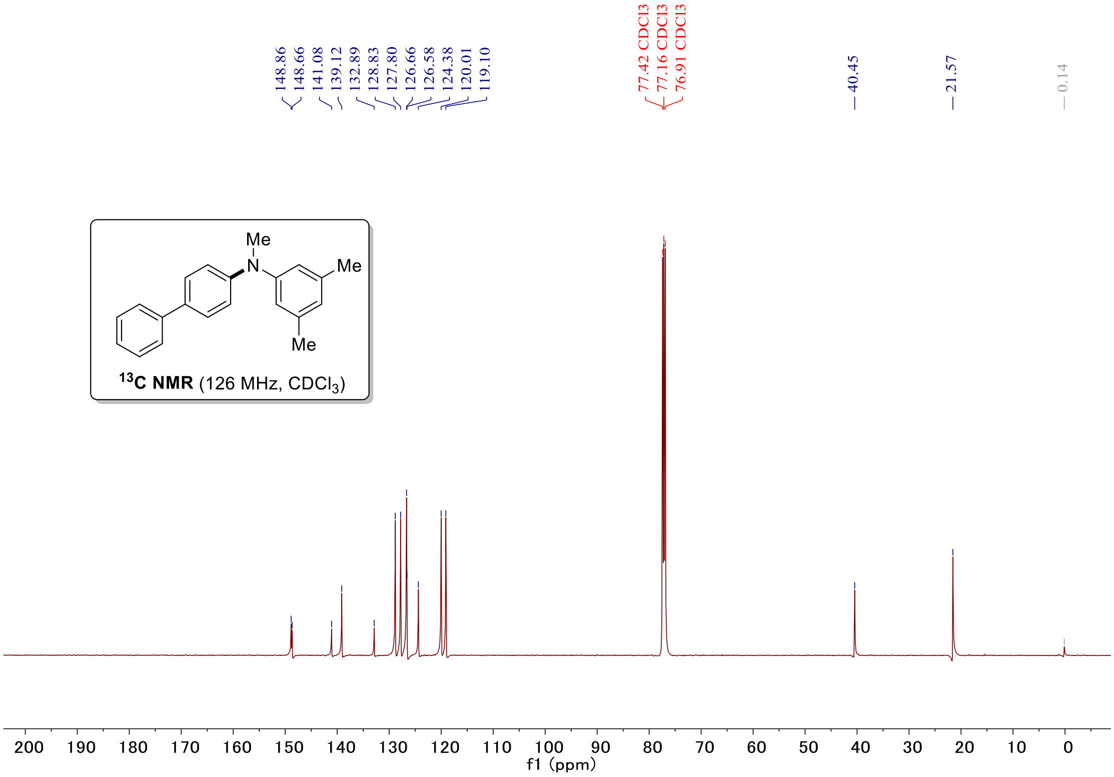

Supplementary Figure 155. ^{13}C NMR (126 MHz, $CDCl_3$, 25 °C) of compound **4ac**

N-Methyl-*N*-(*o*-tolyl)-[1,1'-biphenyl]-4-amine (4ad)

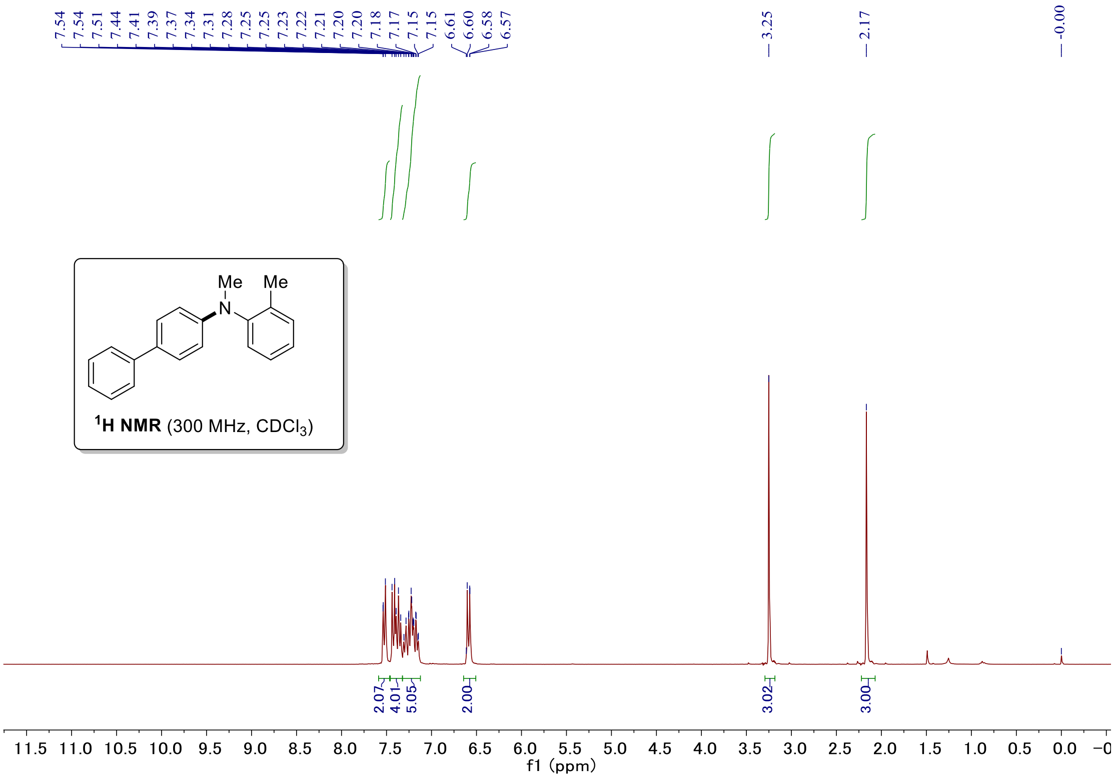

Supplementary Figure 156. ^{1}H NMR (300 MHz, $CDCl_3$, 25 ^{o}C) of compound **4ad**

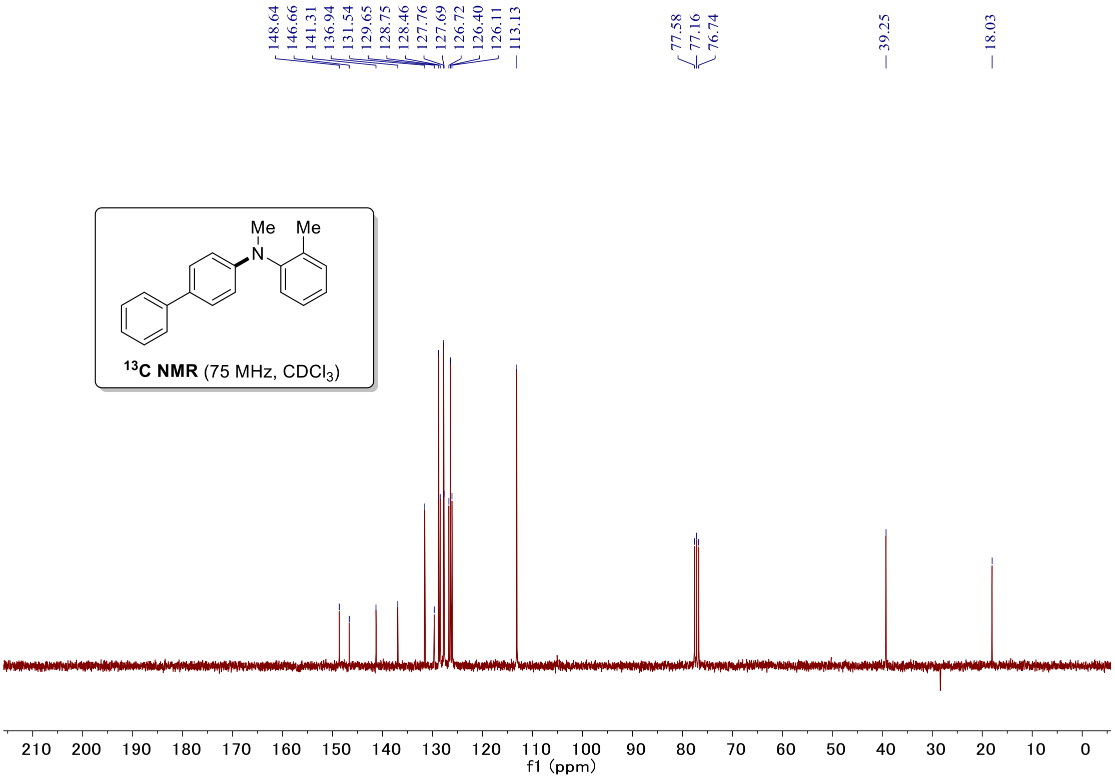

Supplementary Figure 157. ^{13}C NMR (75 MHz, $CDCl_3$, 25 ^{o}C) of compound **4ad**

N-Mesityl-N-methyl-[1,1'-biphenyl]-4-amine (4ae)

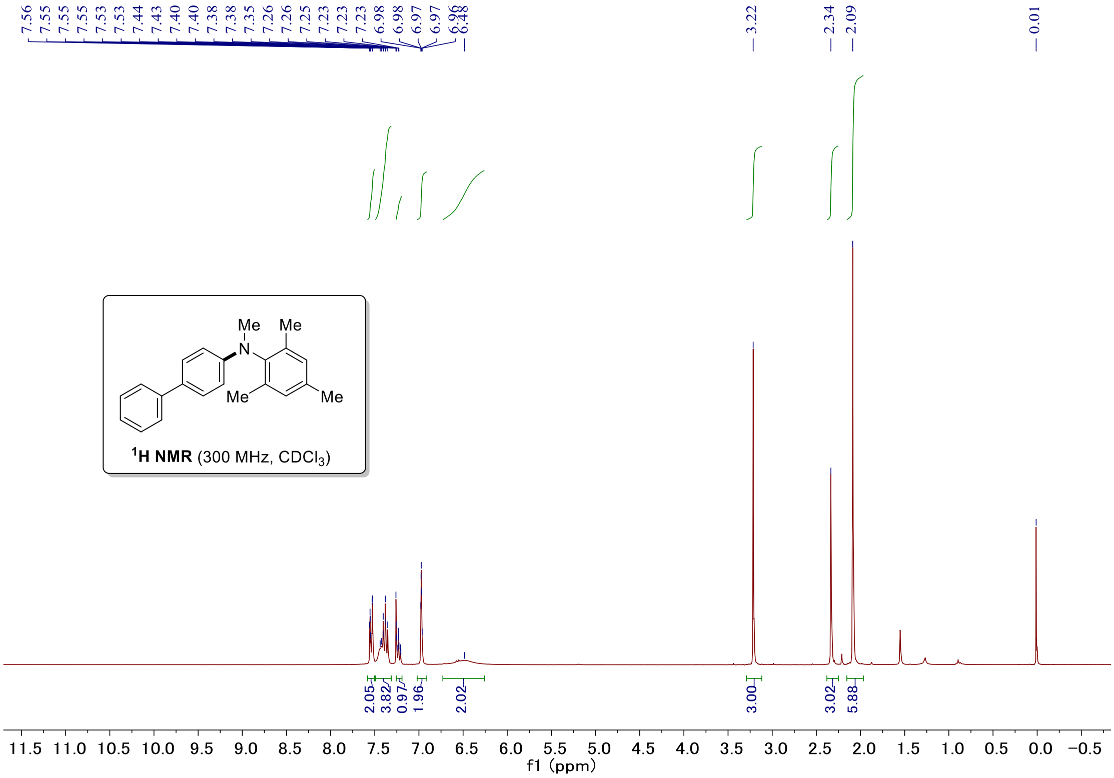

Supplementary Figure 158. ^{1}H NMR (300 MHz, $CDCl_3$, 25 °C) of compound **4ae**

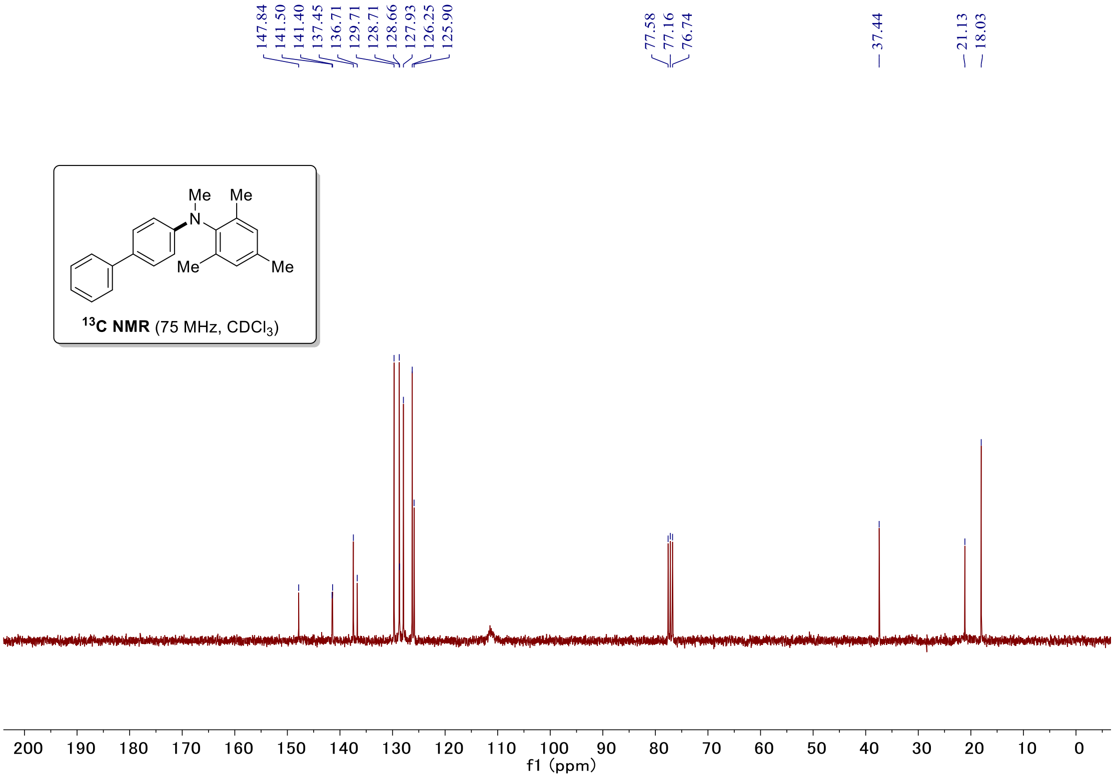

Supplementary Figure 159. ^{13}C NMR (75 MHz, $CDCl_3$, 25 °C) of compound **4ae**

N-(4-Methoxyphenyl)-N-methyl-[1,1'-biphenyl]-4-amine (4af)

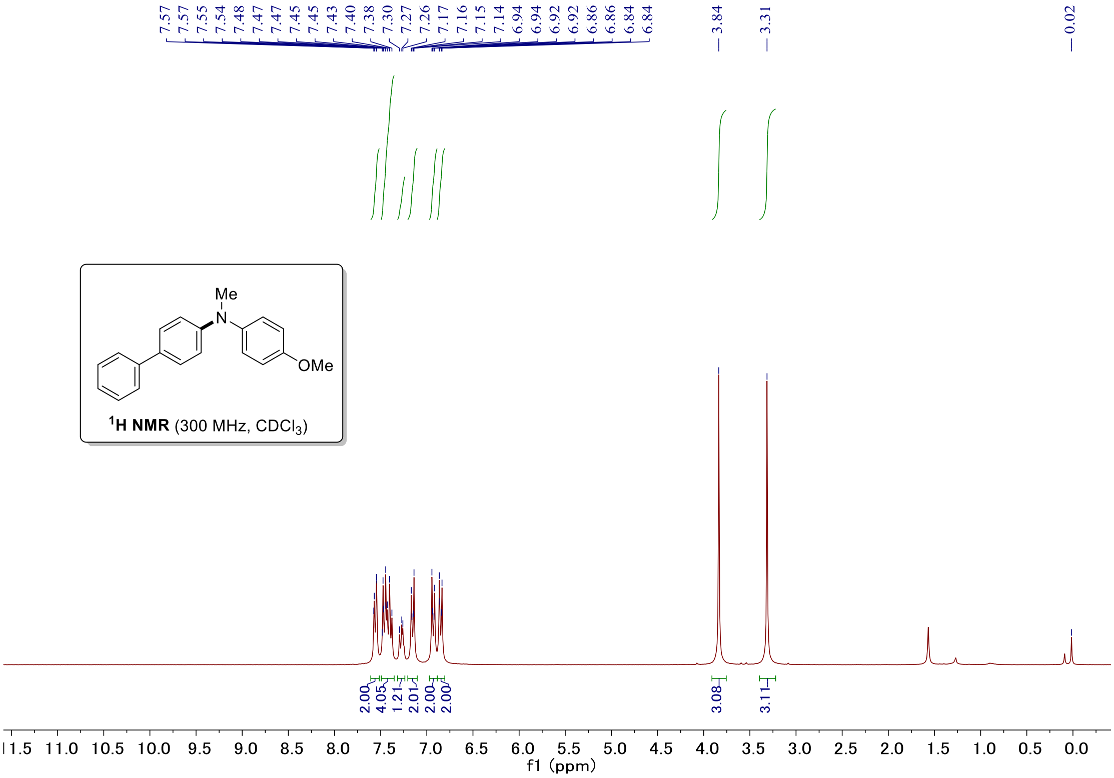

Supplementary Figure 160. ^{1}H NMR (300 MHz, $CDCl_3$, 25 °C) of compound **4af**

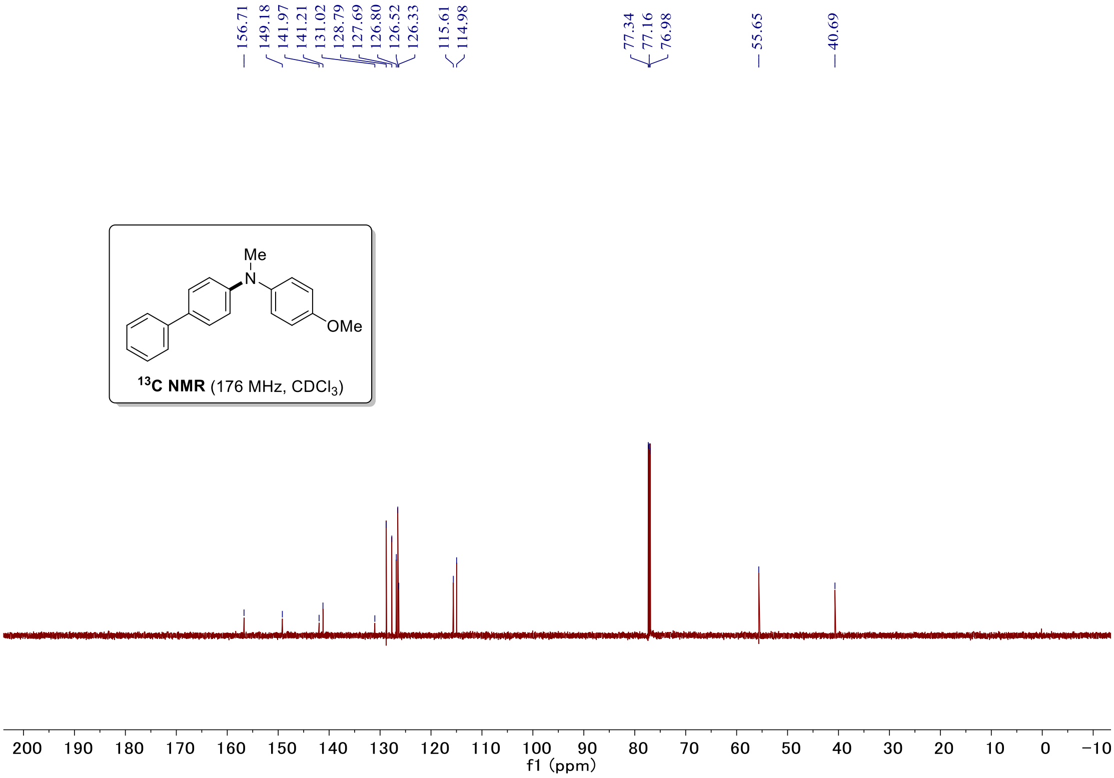

Supplementary Figure 161. ^{13}C NMR (176 MHz, $CDCl_3$, 25 °C) of compound **4af**

N-([1,1'-Biphenyl]-4-yl)-*N*-methylbenzo[*d*][1,3]dioxol-5-amine (4ag)

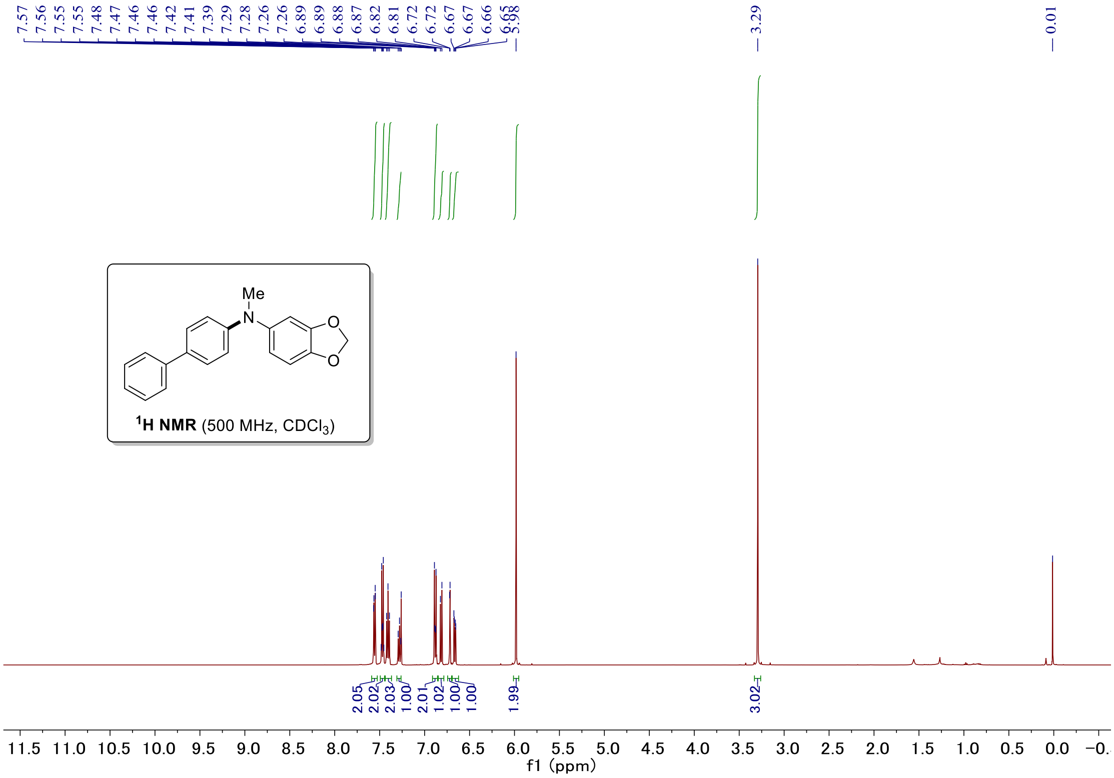

Supplementary Figure 162. ^{1}H NMR (500 MHz, $CDCl_3$, 25 °C) of compound **4ag**

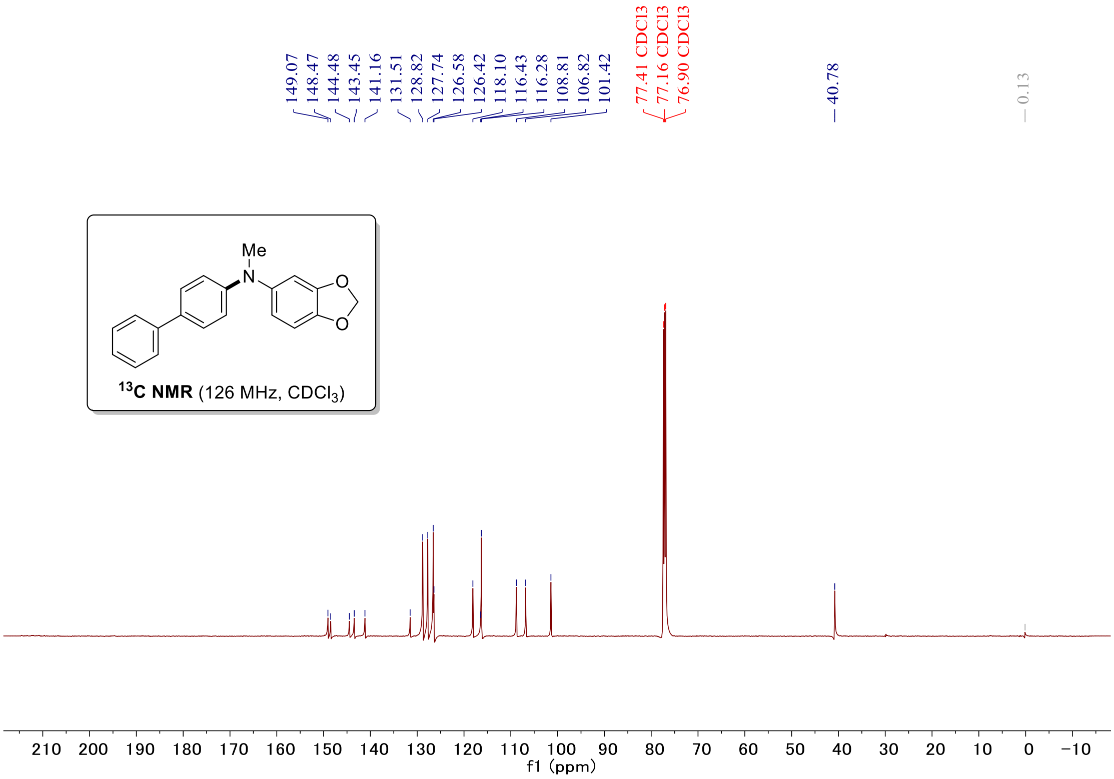

Supplementary Figure 163. ^{13}C NMR (126 MHz, $CDCl_3$, 25 °C) of compound **4ag**

N-Methyl-*N*-(4-(trifluoromethoxy)phenyl)-[1,1'-biphenyl]-4-amine (4ah)

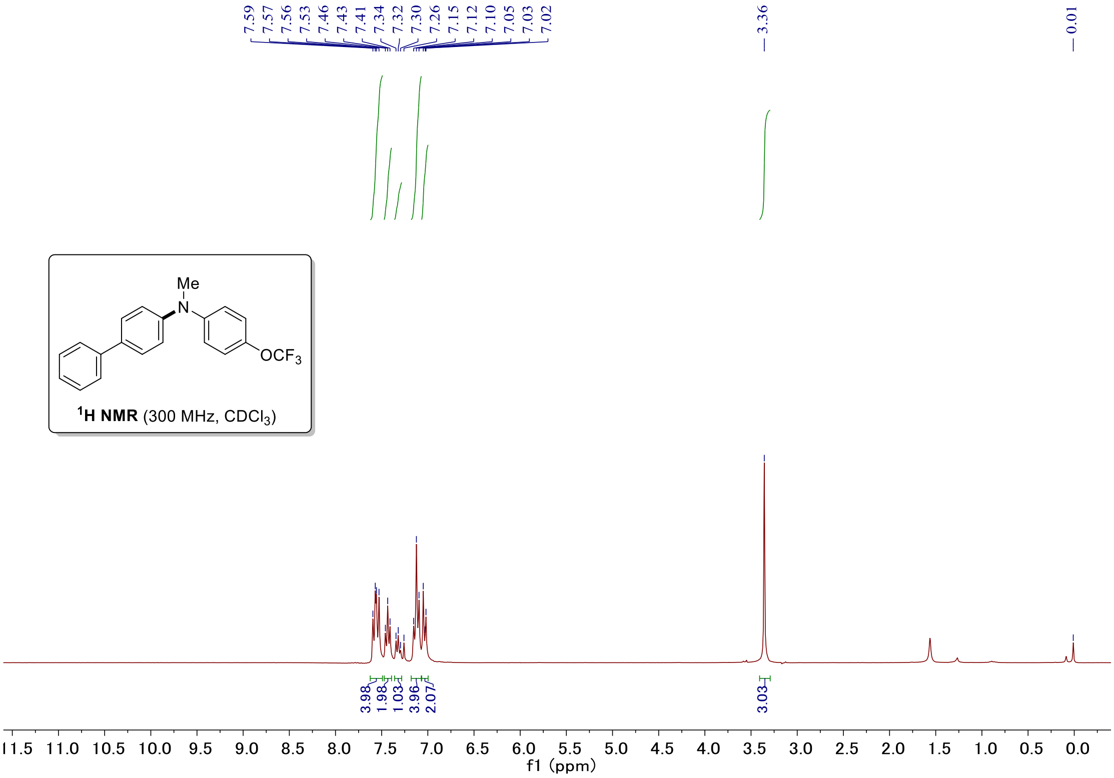

Supplementary Figure 164. ^{1}H NMR (300 MHz, $CDCl_3$, 25 °C) of compound **4ah**

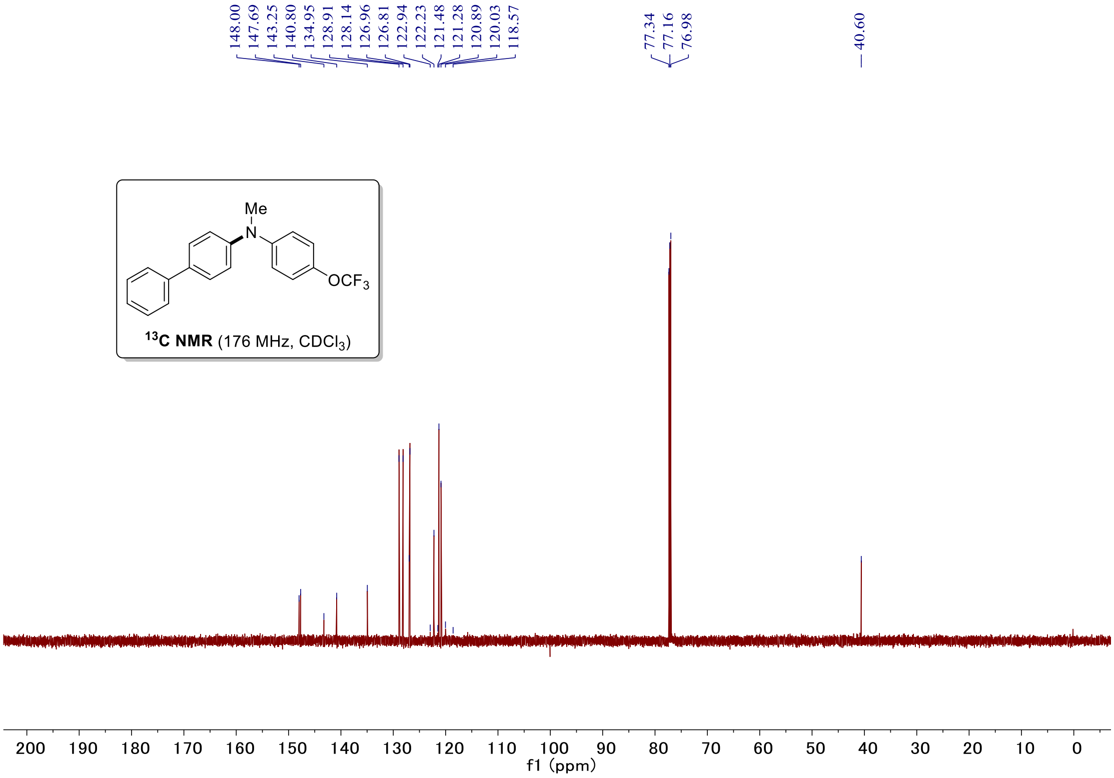

Supplementary Figure 165. ^{13}C NMR (176 MHz, $CDCl_3$, 25 °C) of compound **4ah**

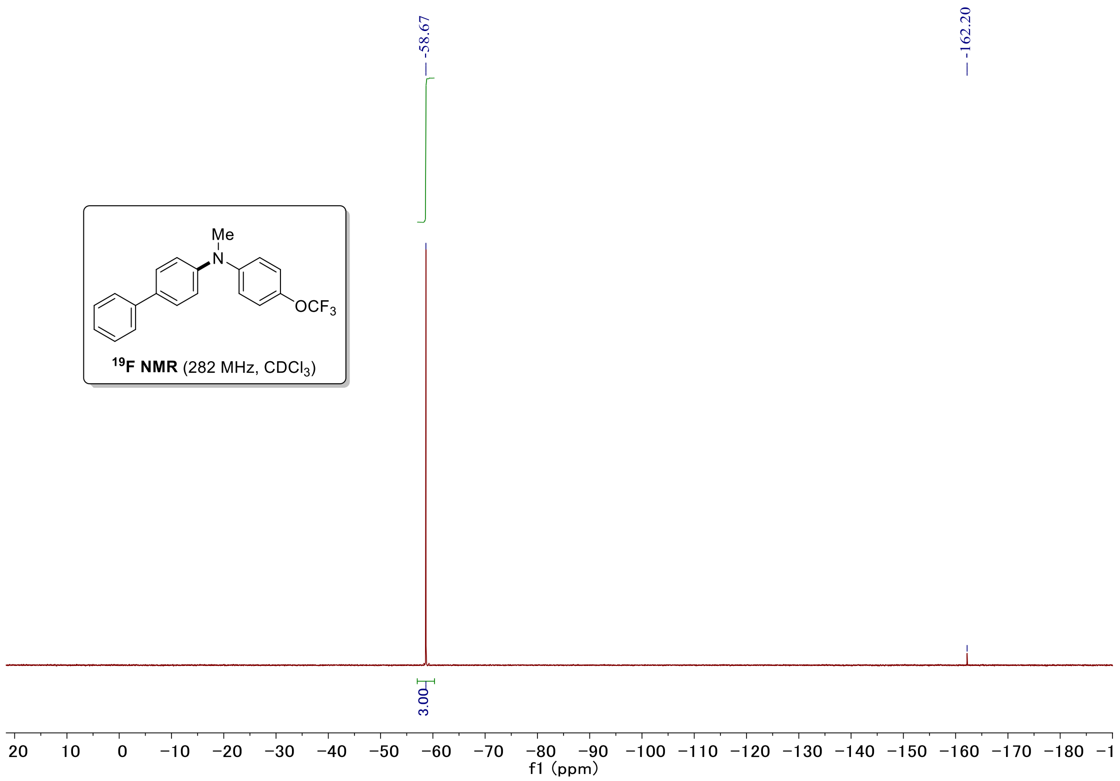

Supplementary Figure 166. ^{19}F NMR (282 MHz, $CDCl_3$, 25 °C) of compound **4ah**

N-(4-Chlorophenyl)-*N*-methyl-[1,1'-biphenyl]-4-amine (4ai)

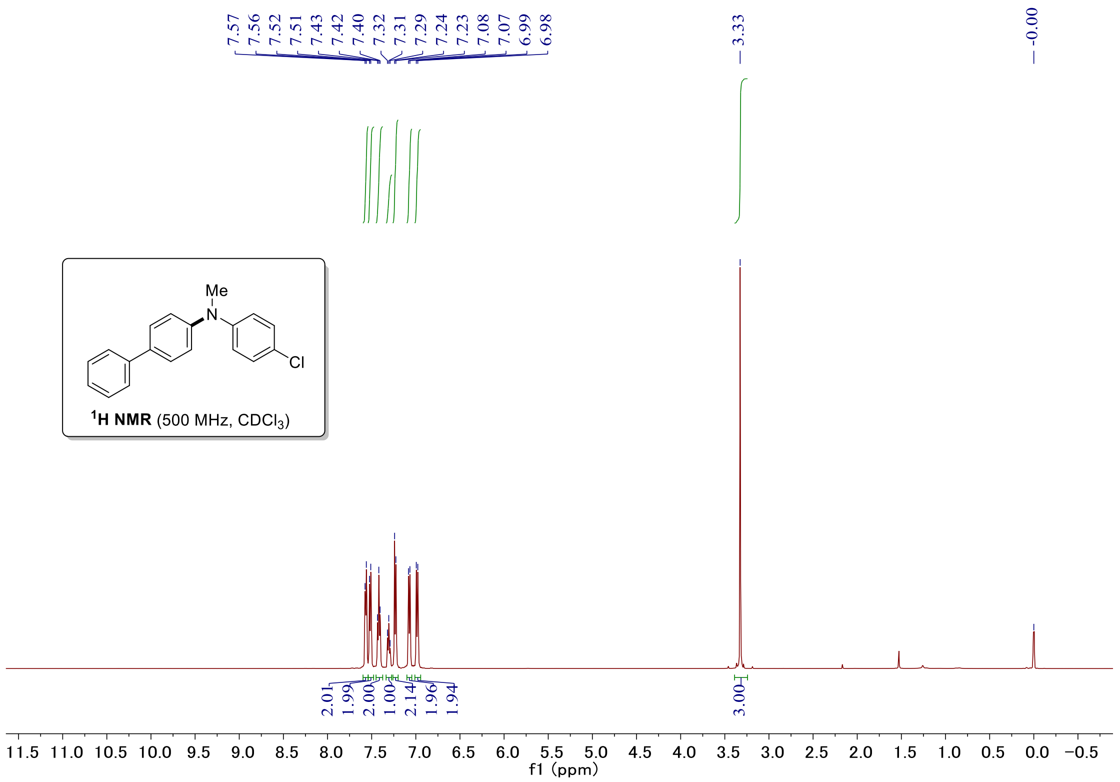

Supplementary Figure 167. ^{1}H NMR (500 MHz, $CDCl_3$, 25 °C) of compound **4ai**

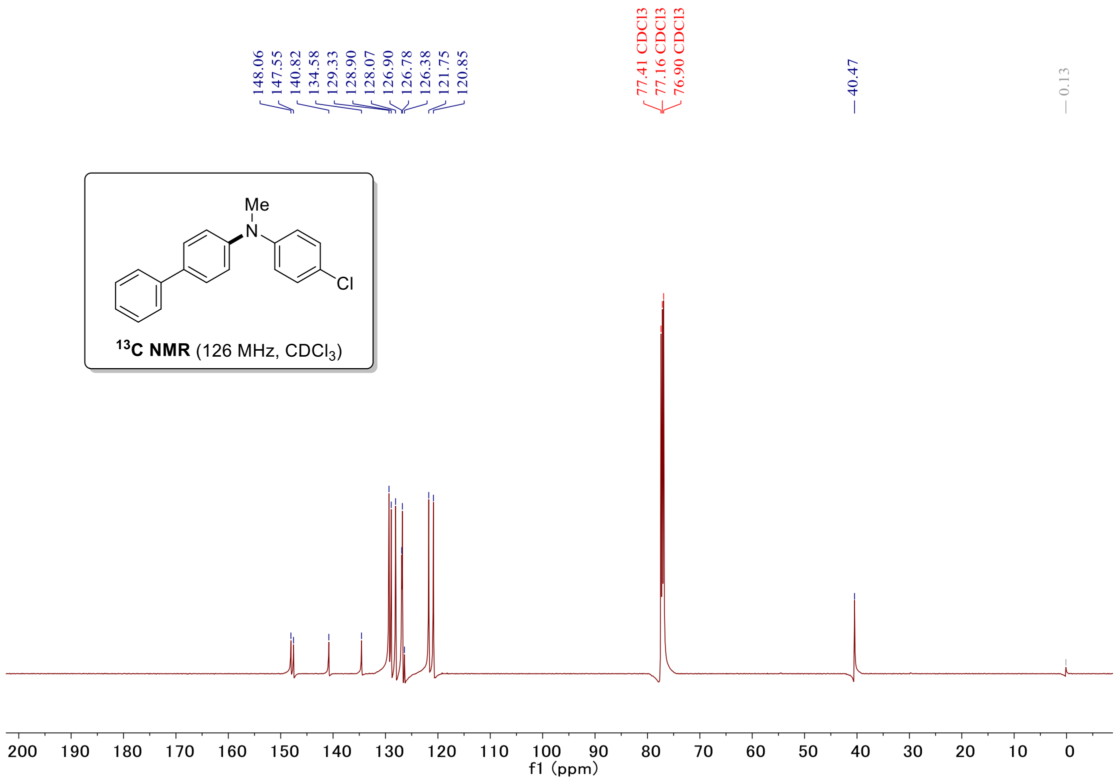

Supplementary Figure 168. ^{13}C NMR (126 MHz, $CDCl_3$, 25 °C) of compound **4ai**

N-(3-Chlorophenyl)-*N*-methyl-[1,1'-biphenyl]-4-amine (4aj)

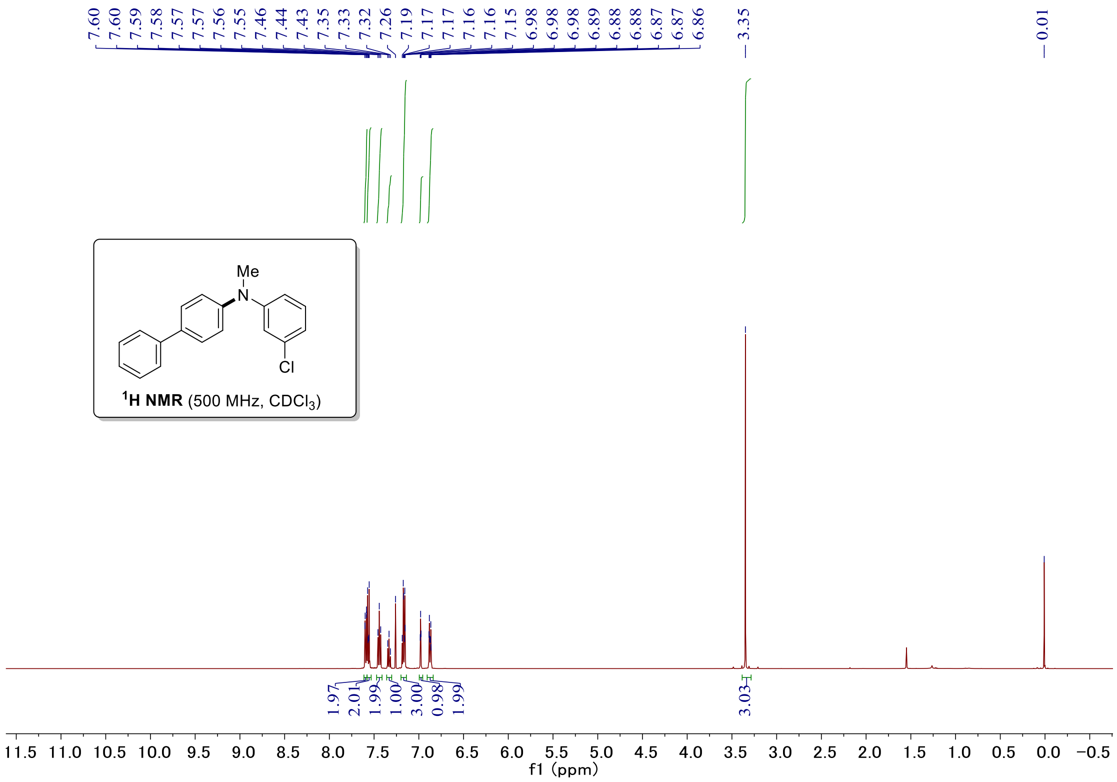

Supplementary Figure 169. ^{1}H NMR (500 MHz, $CDCl_3$, 25 °C) of compound **4aj**

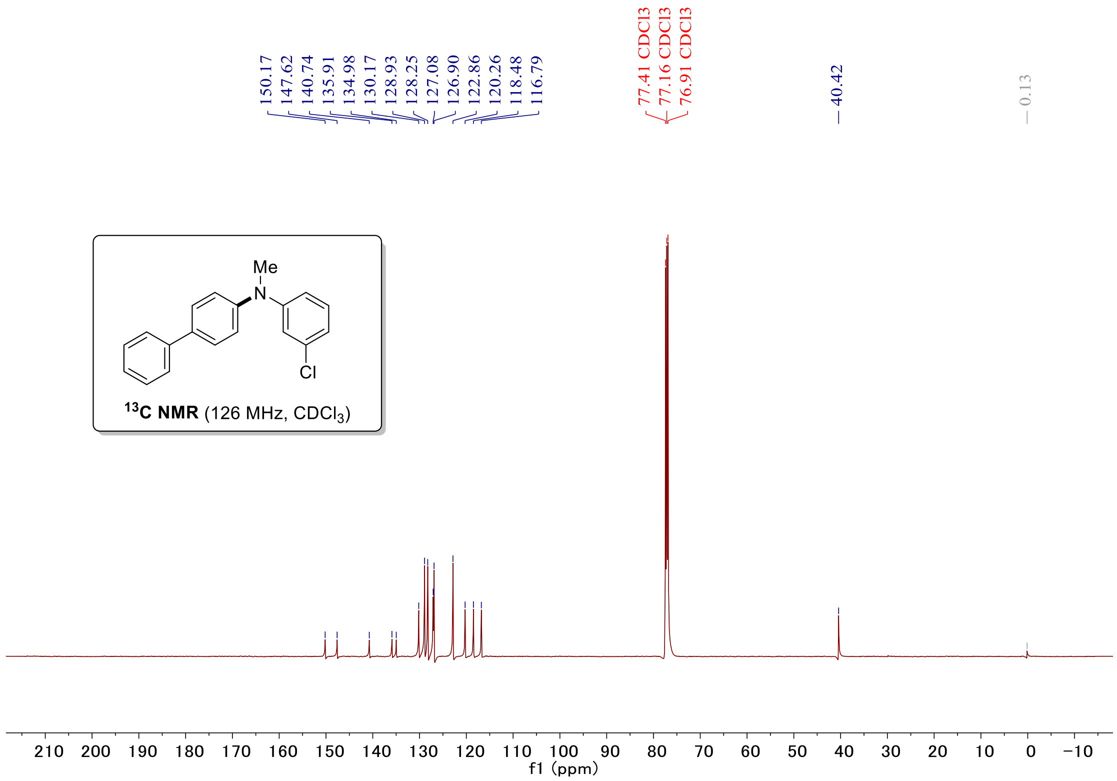

Supplementary Figure 170. ^{13}C NMR (126 MHz, $CDCl_3$, 25 °C) of compound **4aj**

***N*-(4-Bromophenyl)-*N*-methyl-[1,1'-biphenyl]-4-amine (4ak)**

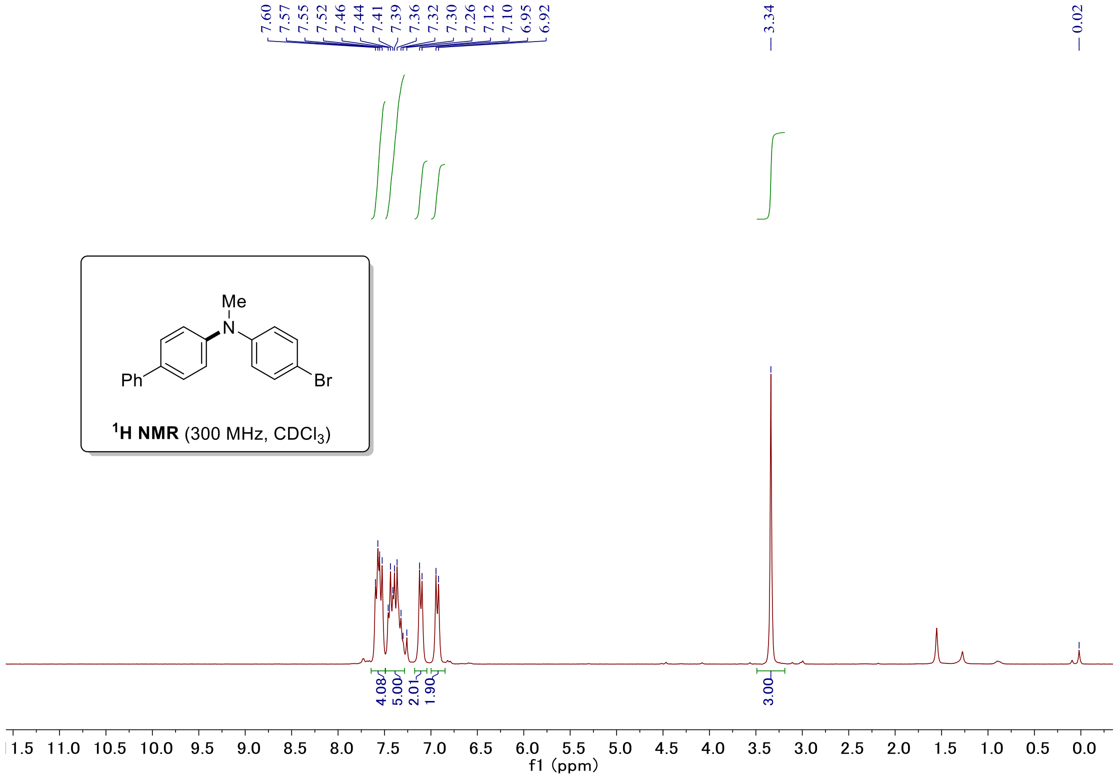

Supplementary Figure 171. ^{1}H NMR (300 MHz, $CDCl_3$, 25 °C) of compound **4ak**

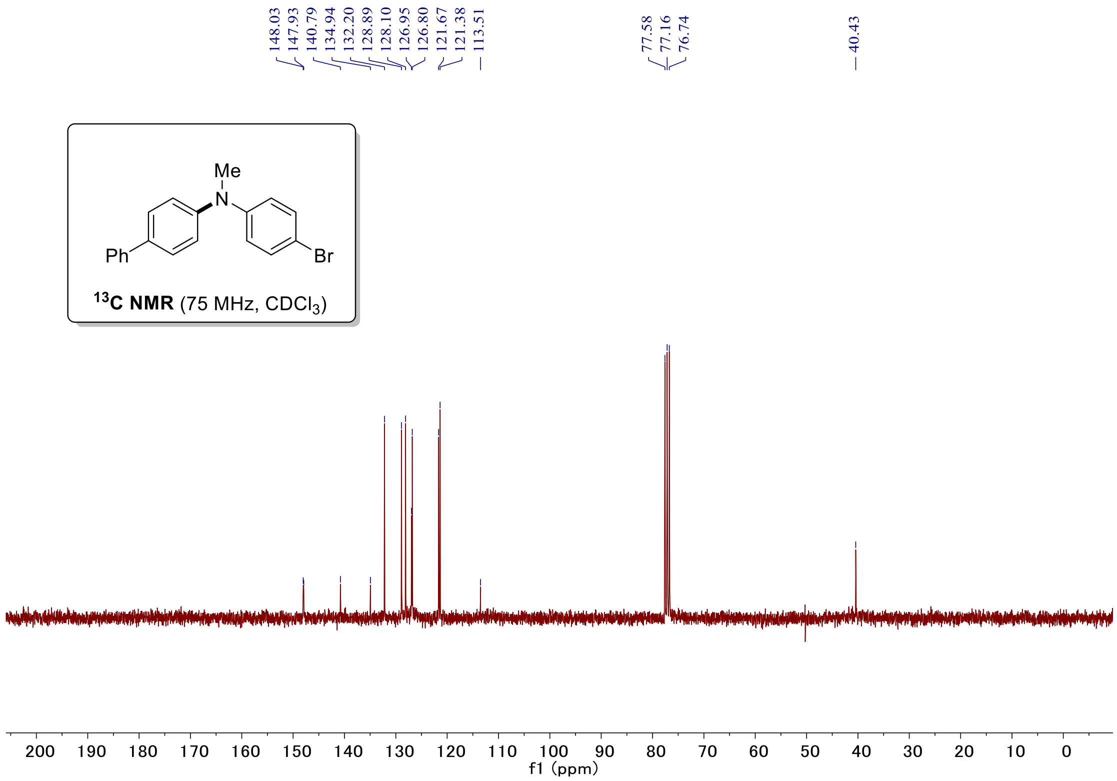

Supplementary Figure 172. ^{13}C NMR (75 MHz, $CDCl_3$, 25 °C) of compound **4ak**

N-(3,5-Dimethylphenyl)-*N*-methyl-[1,1'-biphenyl]-3-amine (4bc)

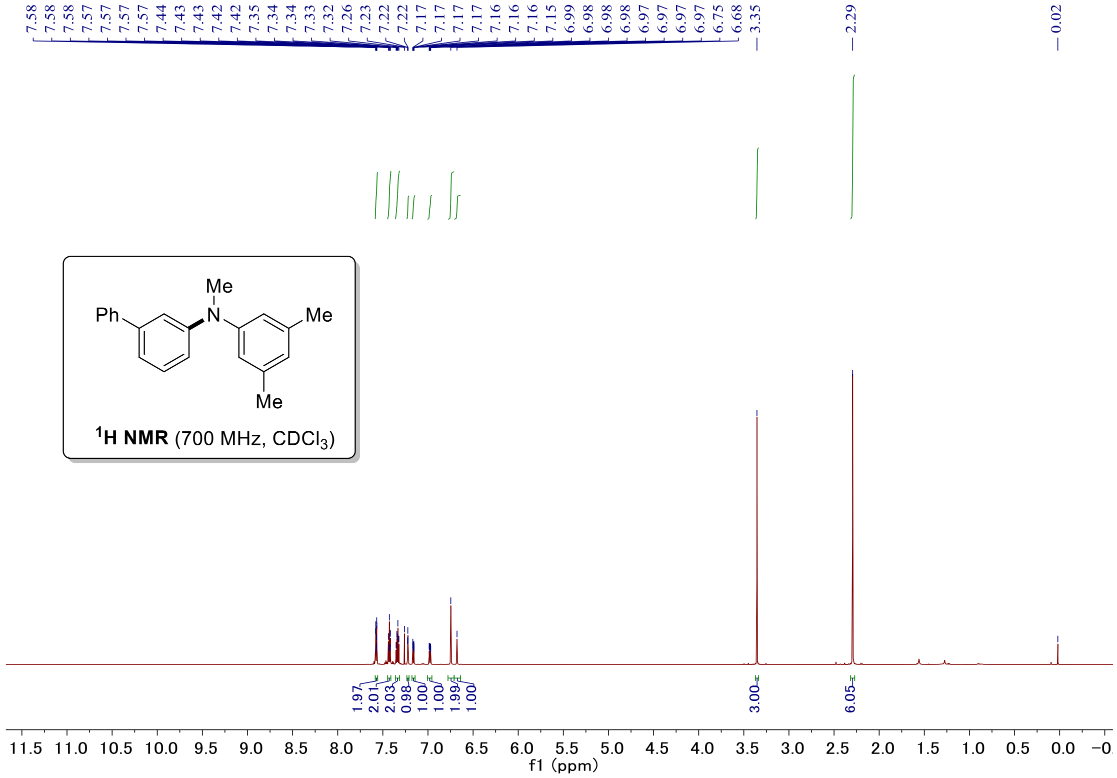

Supplementary Figure 173. ^{1}H NMR (700 MHz, $CDCl_3$, 25 °C) of compound **4bc**

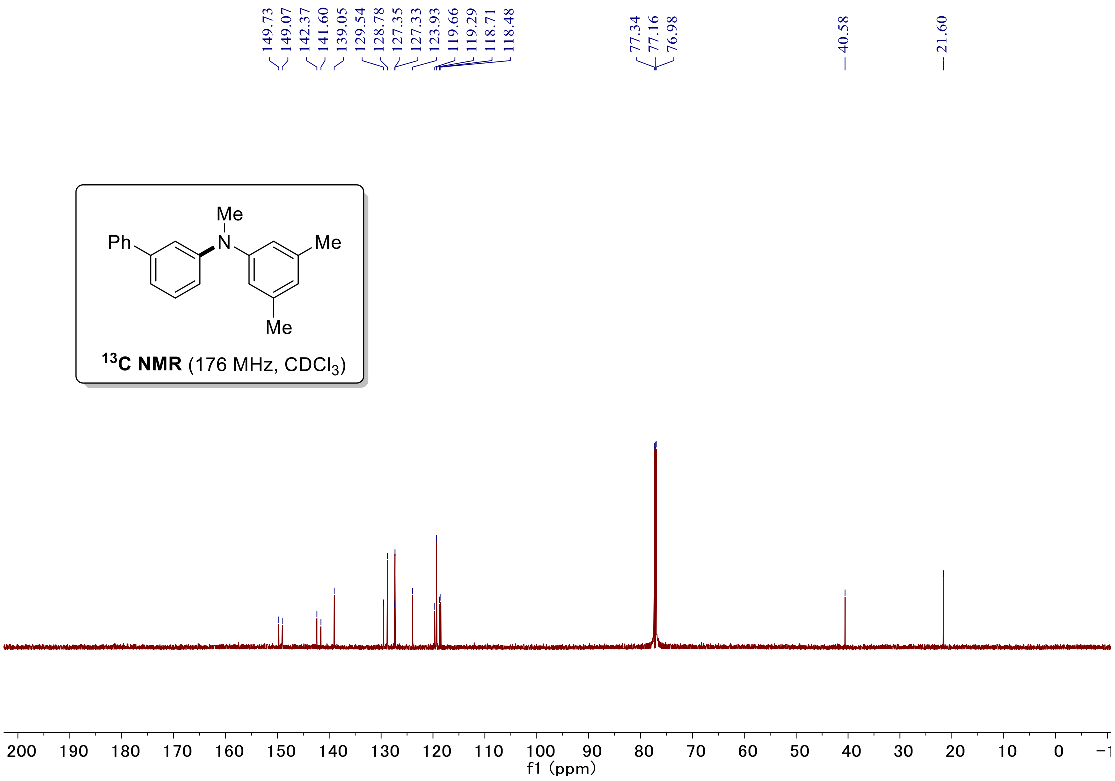

Supplementary Figure 174. ^{13}C NMR (126 MHz, $CDCl_3$, 25 °C) of compound **4bc**

N-Methyl-*N*-(*o*-tolyl)-[1,1'-biphenyl]-2-amine (4cd)

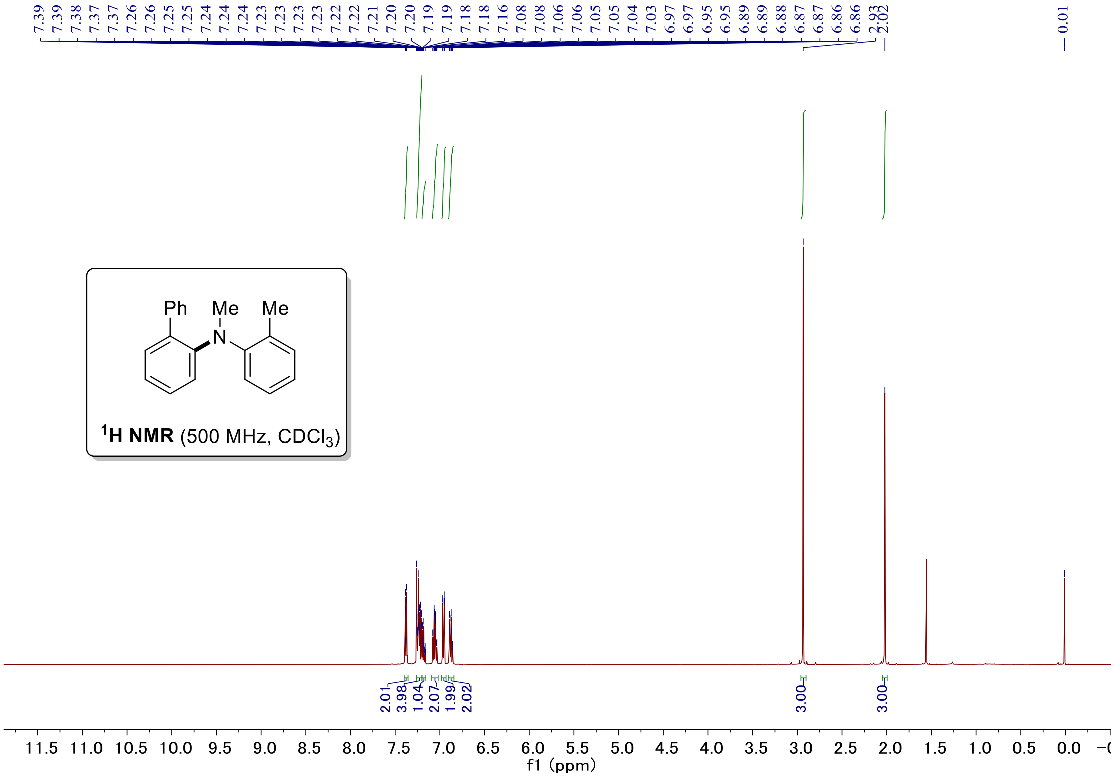

Supplementary Figure 175. ^{1}H NMR (500 MHz, $CDCl_3$, 25 °C) of compound **4cd**

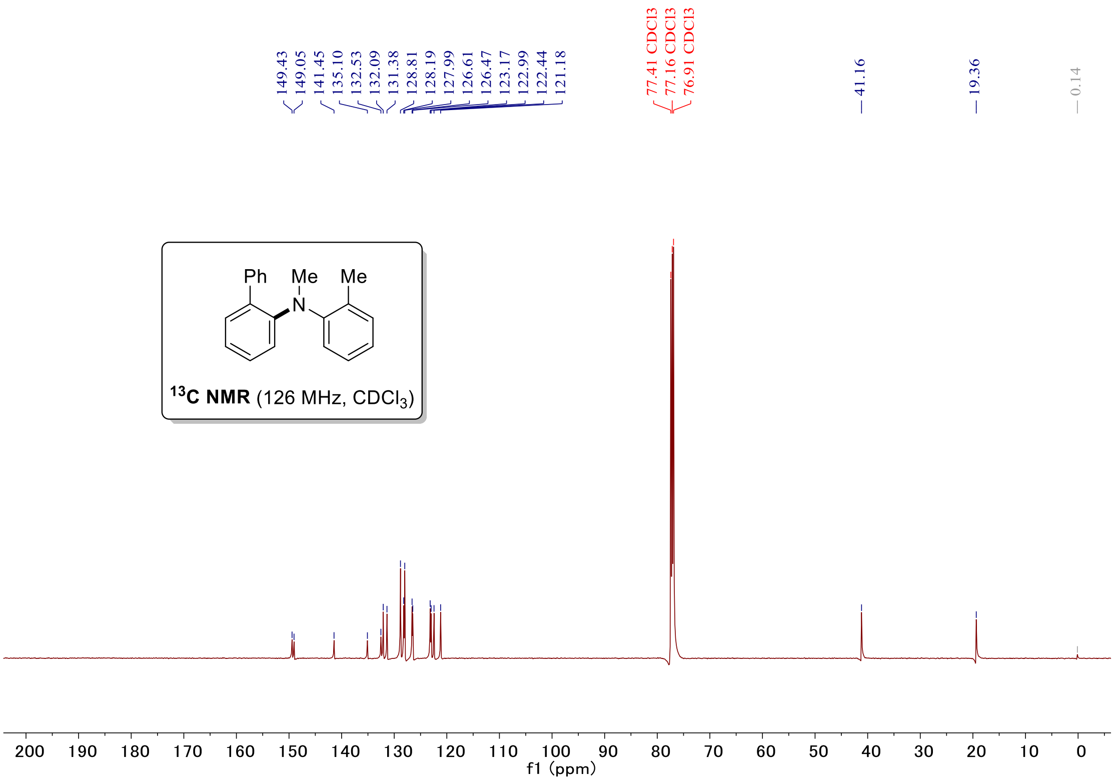

Supplementary Figure 176. ^{13}C NMR (126 MHz, $CDCl_3$, 25 °C) of compound **4cd**

***N*-Methyl-*N*-(4-(trifluoromethoxy)phenyl)naphthalen-1-amine (4dh)**

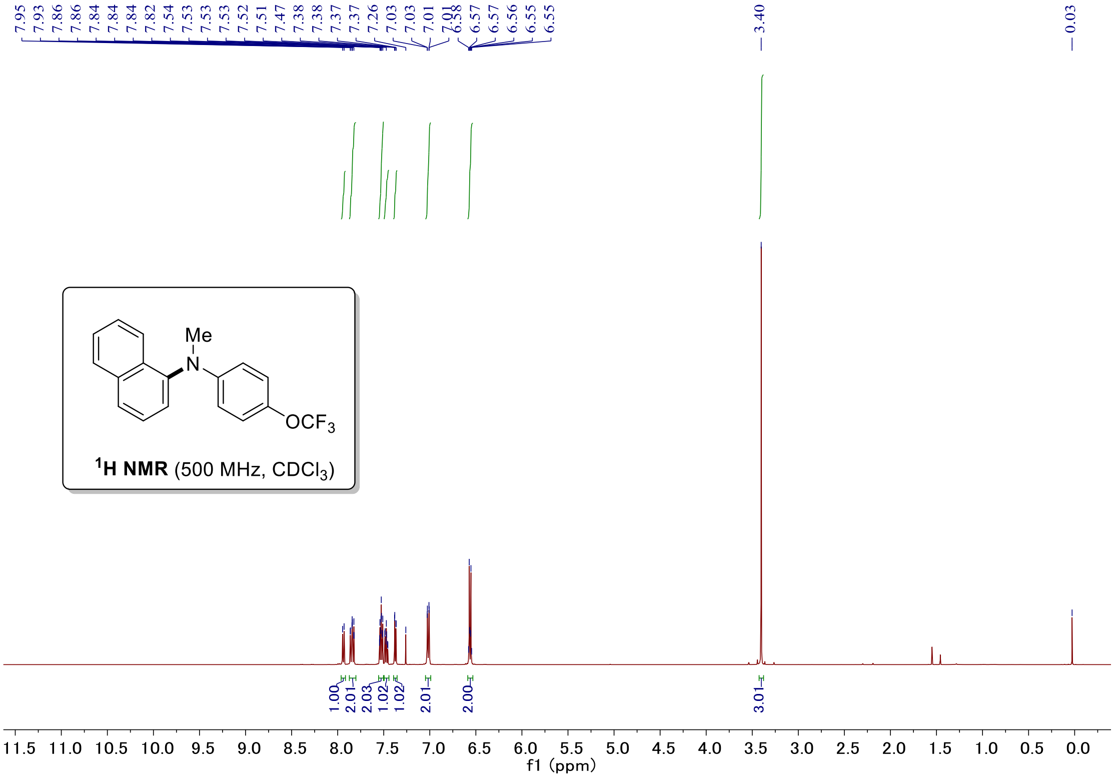

Supplementary Figure 177. ^{1}H NMR (500 MHz, $CDCl_3$, 25 °C) of compound **4dh**

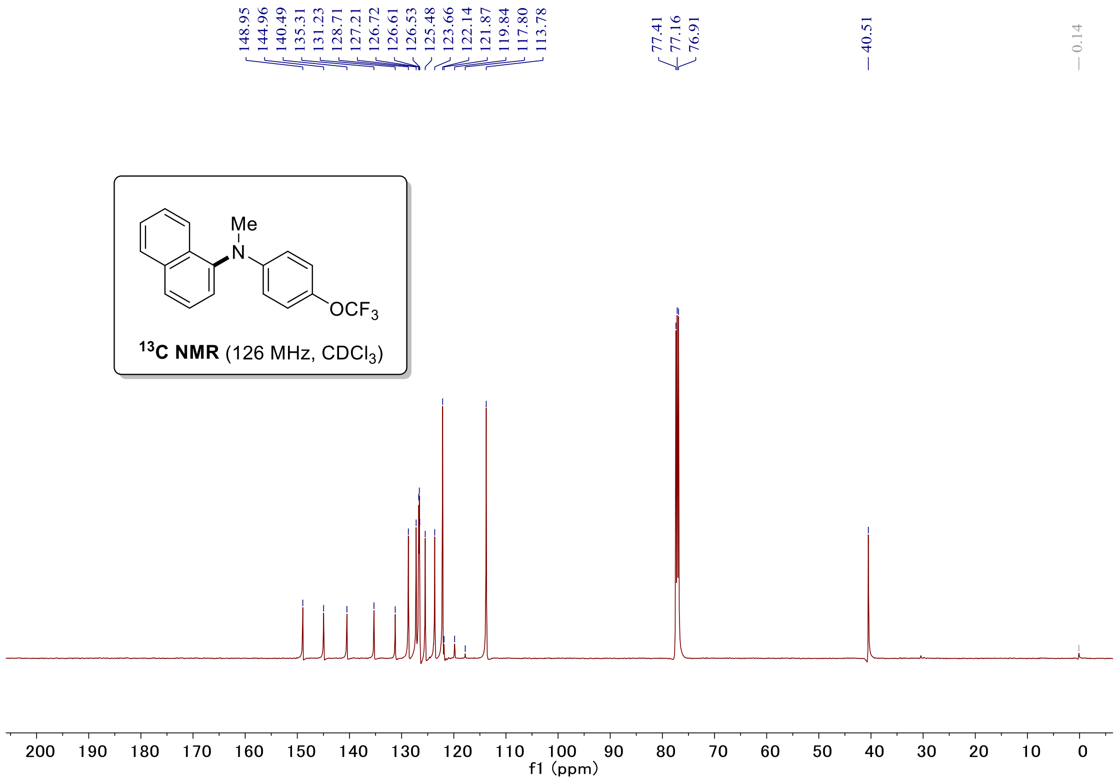

Supplementary Figure 178. ^{13}C NMR (126 MHz, $CDCl_3$, 25 °C) of compound **4dh**

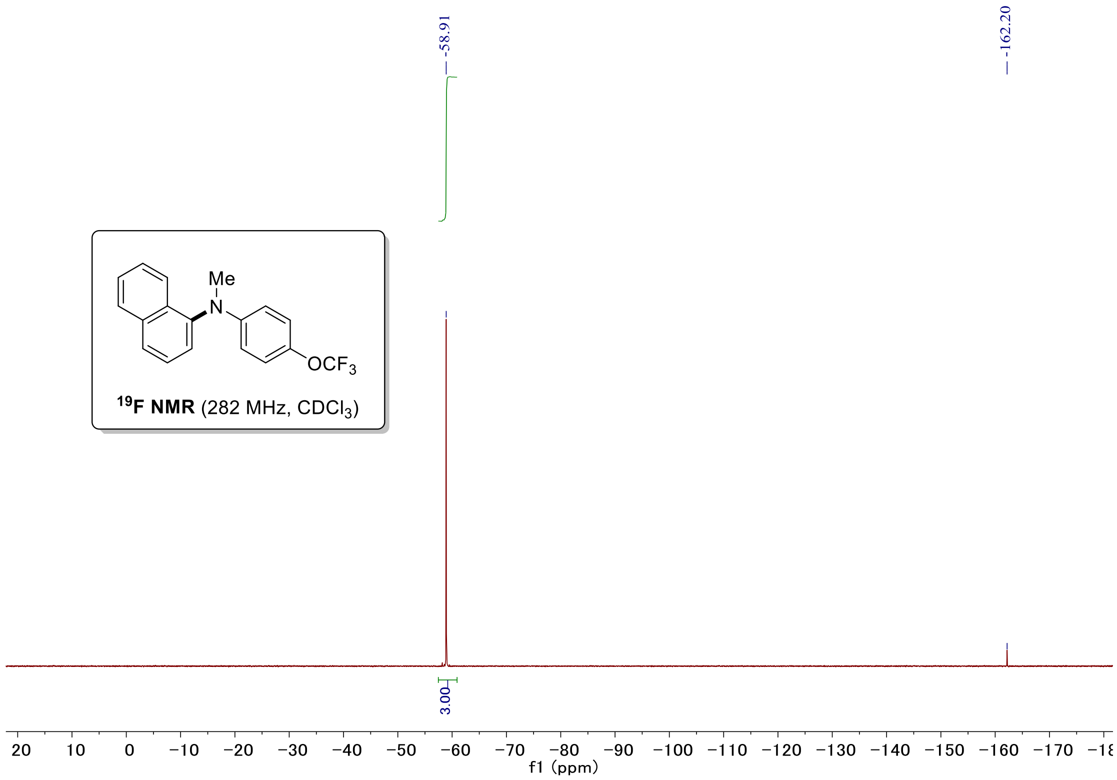

Supplementary Figure 179. ^{19}F NMR (282 MHz, $CDCl_3$, 25 °C) of compound **4dh**

N-(4-methoxyphenyl)-N,3,5-trimethylaniline (4gc)

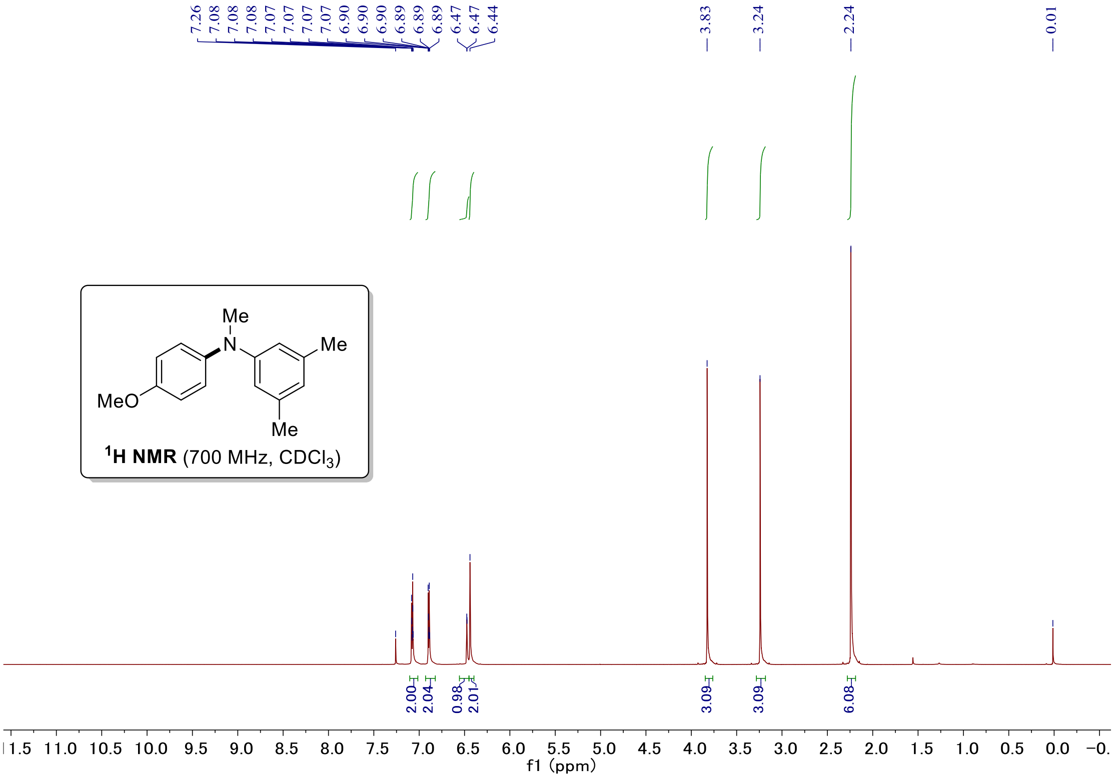

Supplementary Figure 180. ^{1}H NMR (700 MHz, $CDCl_3$, 25 °C) of compound **4gc**

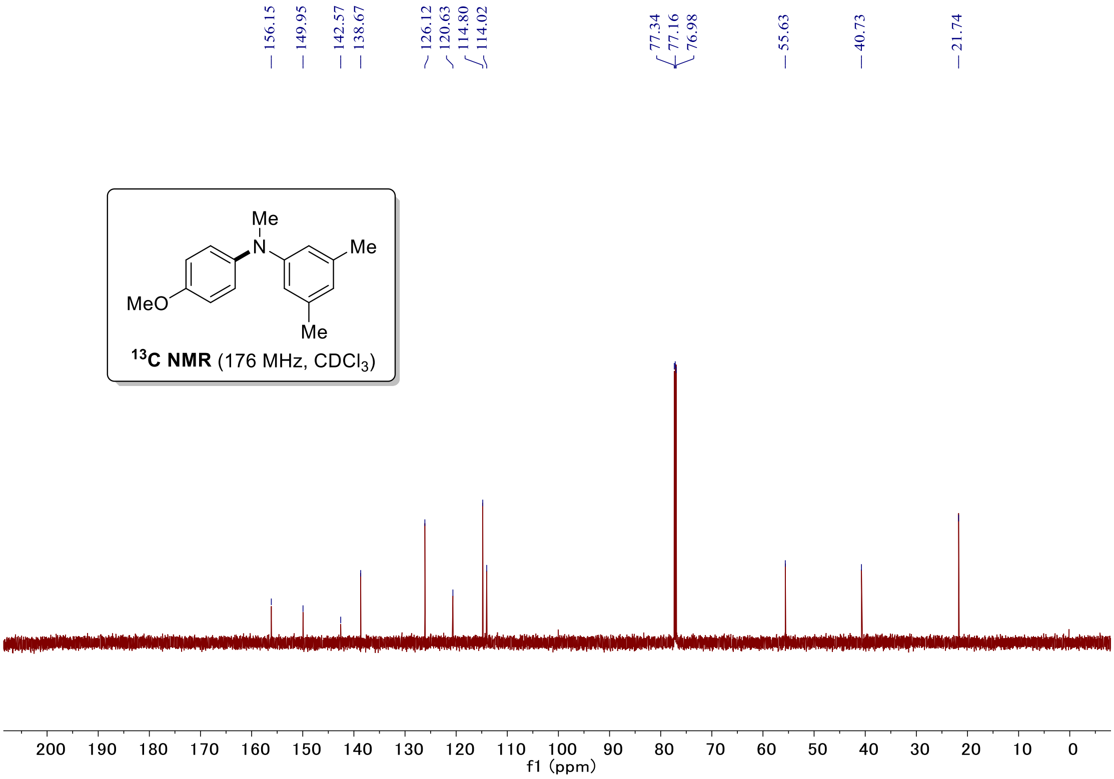

Supplementary Figure 181. ^{13}C NMR (176 MHz, $CDCl_3$, 25 °C) of compound **4gc**

N-Methyl-4-(trifluoromethoxy)-*N*-(4-(trifluoromethyl)phenyl)aniline (4hh)

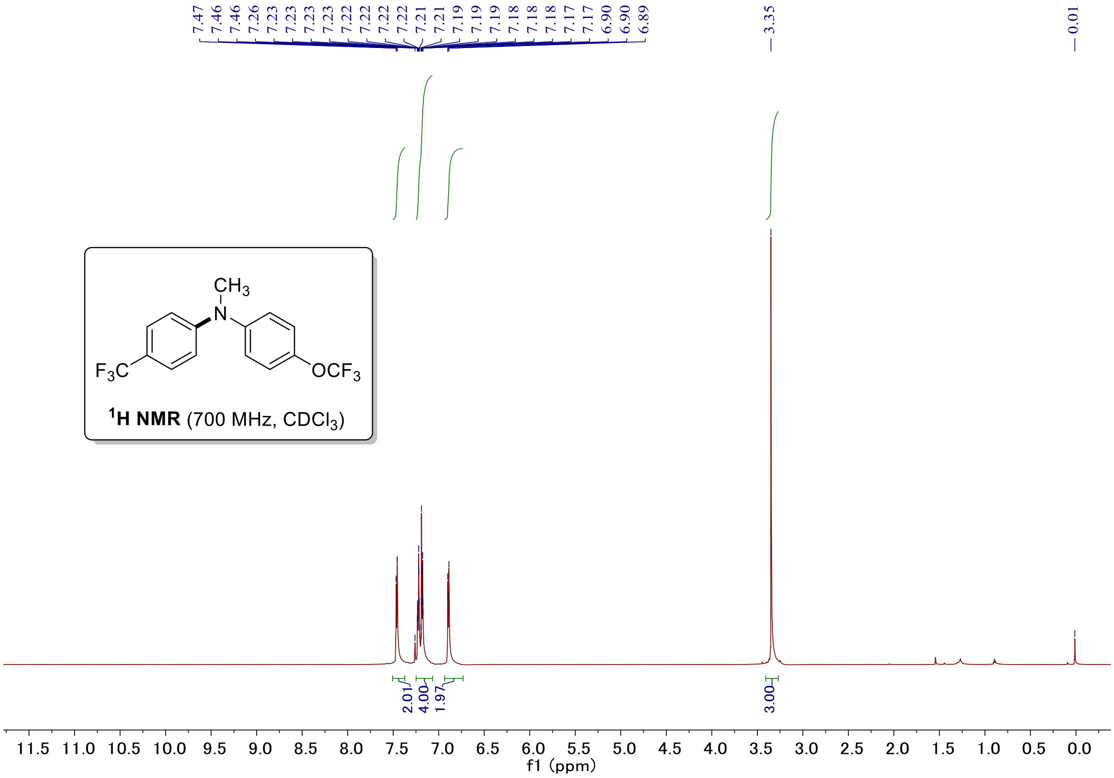

Supplementary Figure 182. ^{1}H NMR (700 MHz, $CDCl_3$, 25 °C) of compound **4hh**

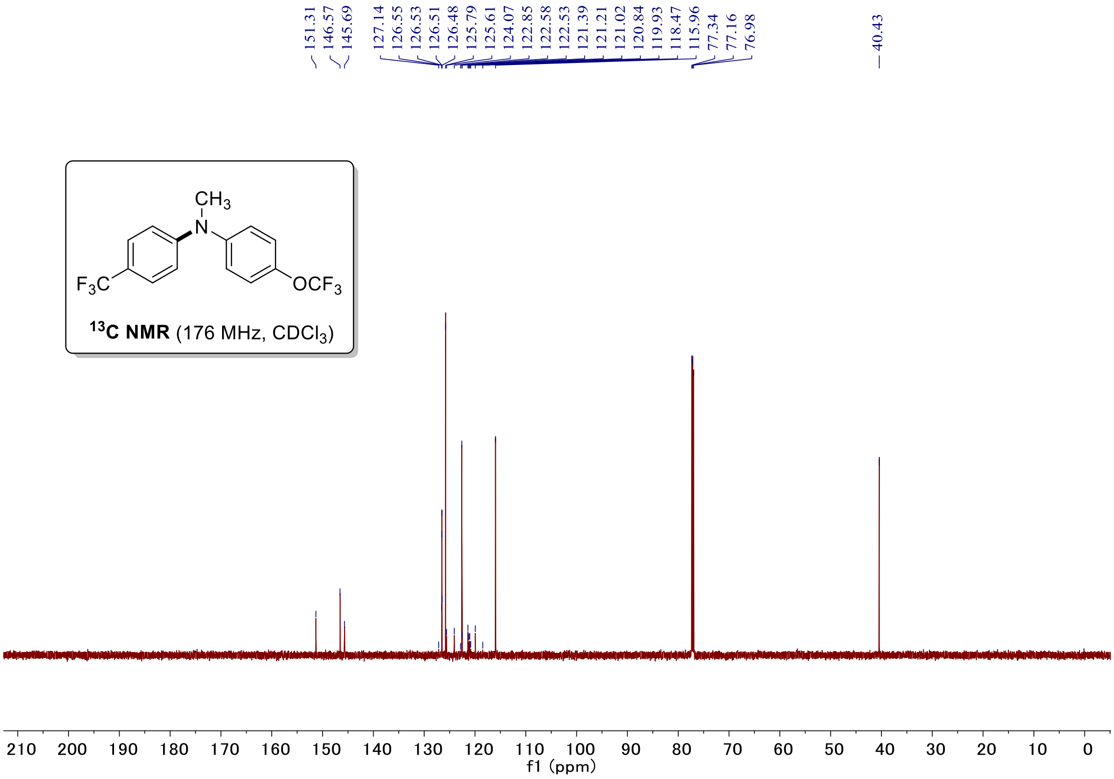

Supplementary Figure 183. ^{13}C NMR (176 MHz, $CDCl_3$, 25 °C) of compound **4hh**

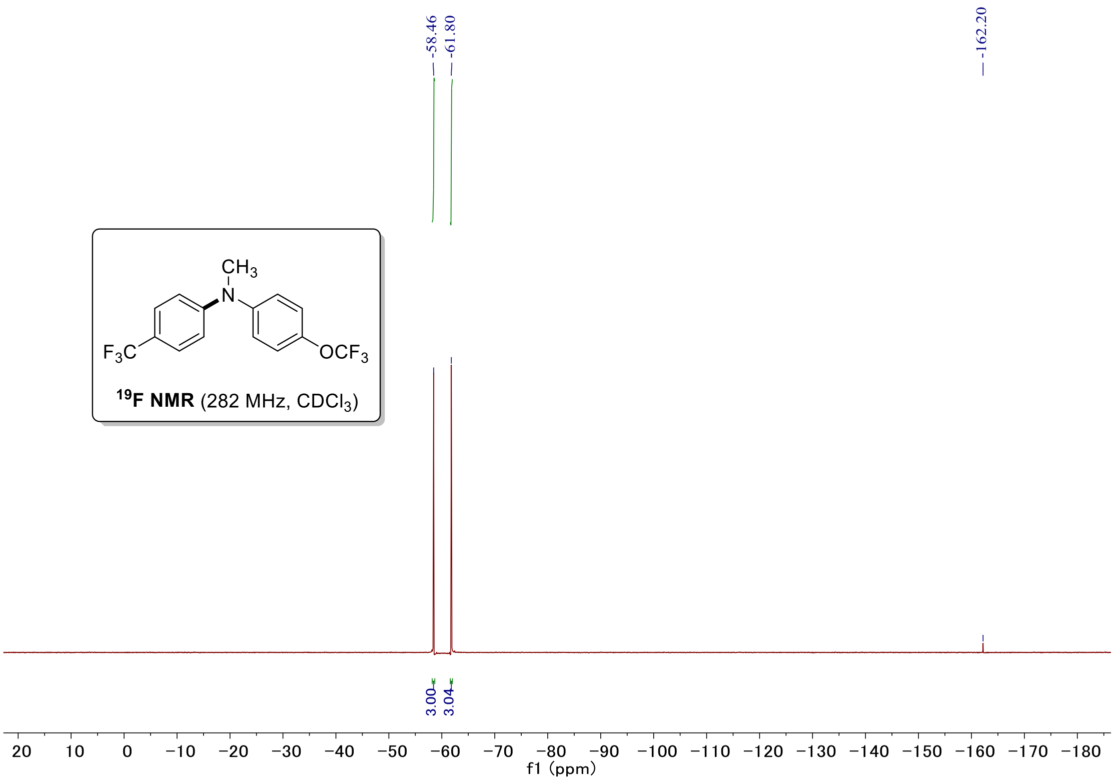

Supplementary Figure 184. ^{19}F NMR (282 MHz, $CDCl_3$, 25 °C) of compound **4hh**

***N*-(4-Chlorophenyl)-*N*,4'-dimethyl-[1,1'-biphenyl]-4-amine (4li)**

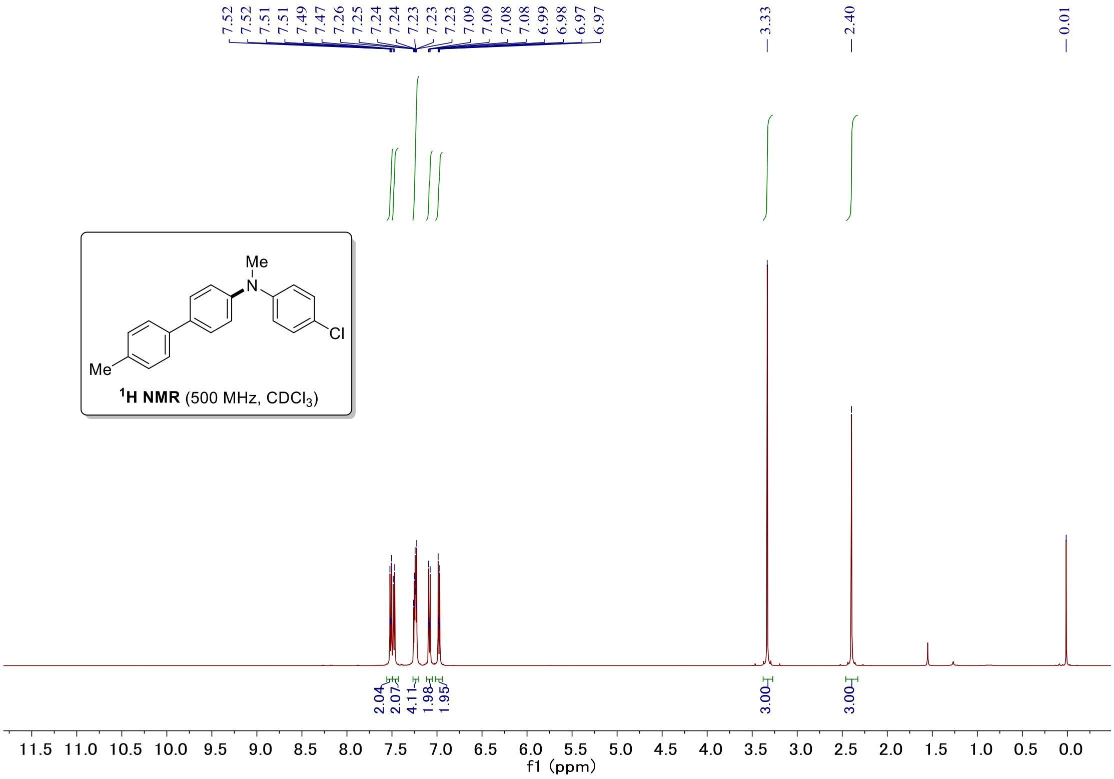

Supplementary Figure 185. ^{1}H NMR (500 MHz, $CDCl_3$, 25 °C) of compound **4li**

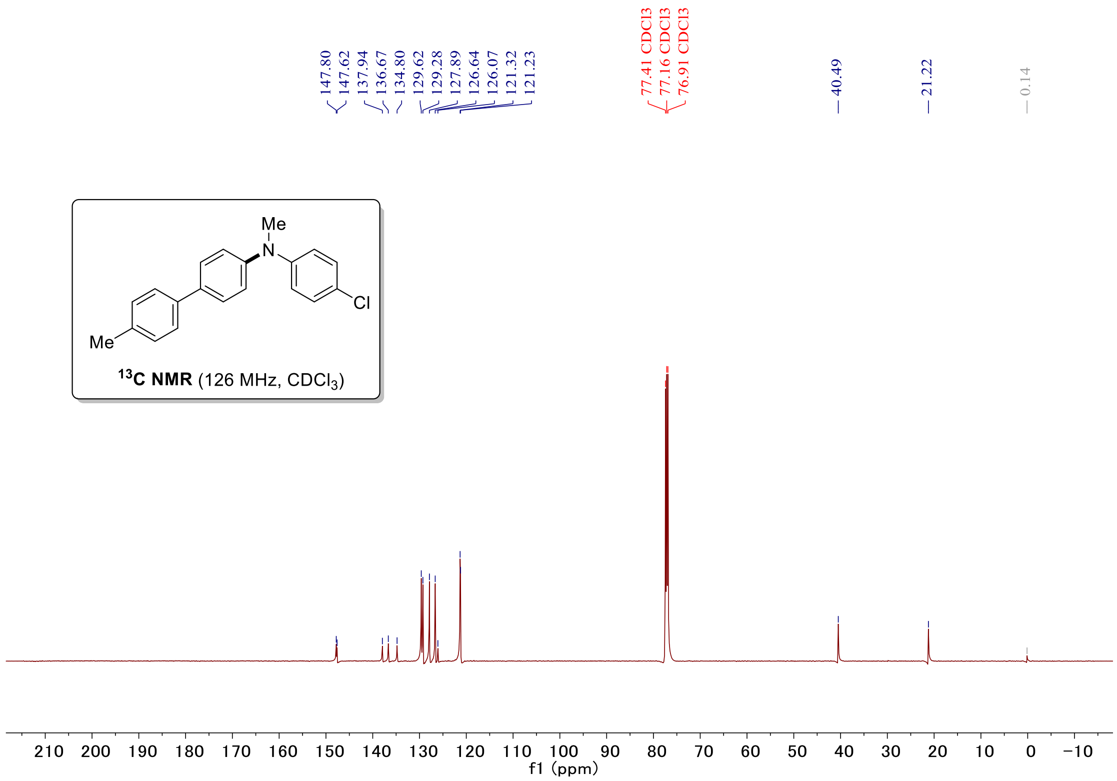

Supplementary Figure 186. ^{13}C NMR (126 MHz, $CDCl_3$, 25 °C) of compound **4li**

_N_,4'-Dimethyl-_N_-(4-(trifluoromethoxy)phenyl)-[1,1'-biphenyl]-4-amine (4lh)

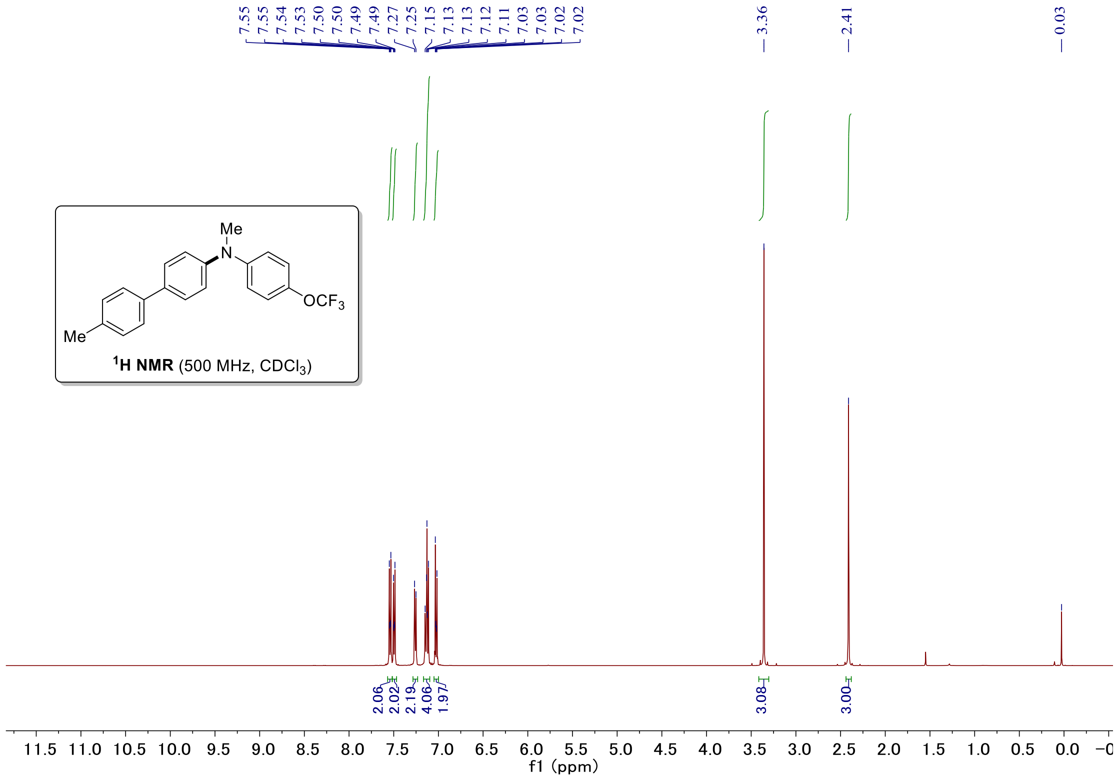

Supplementary Figure 187. ^{1}H NMR (500 MHz, $CDCl_3$, 25 °C) of compound **4lh**

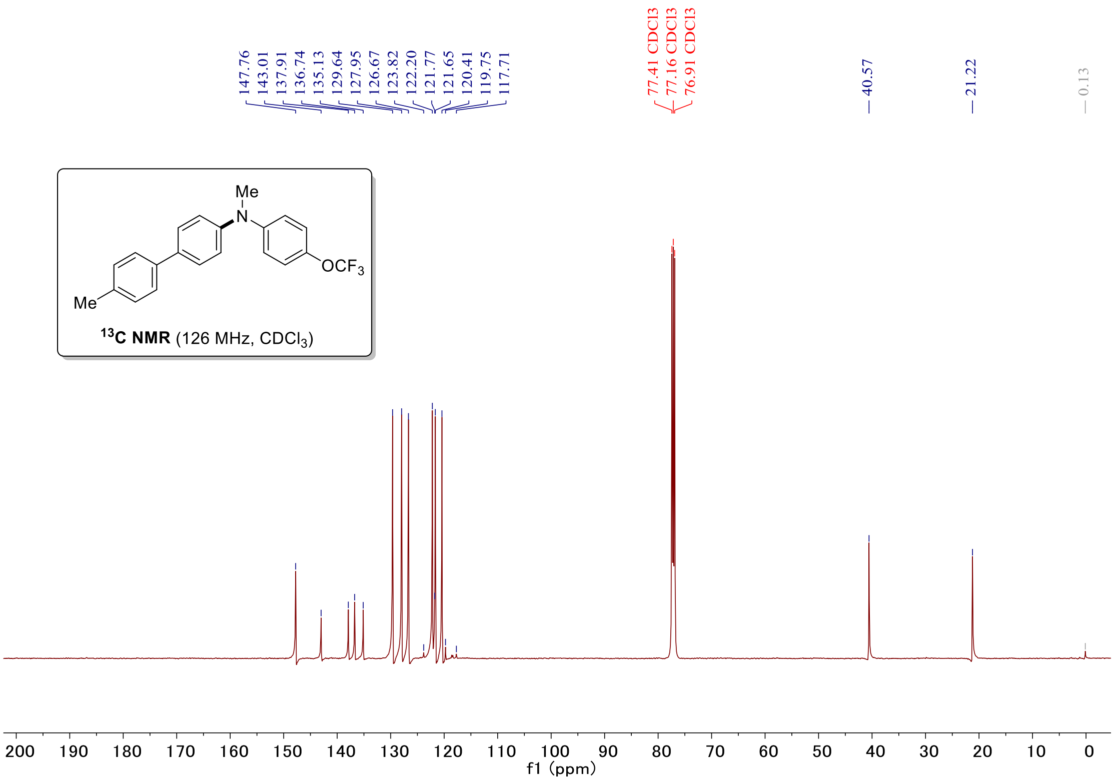

Supplementary Figure 188. ^{13}C NMR (126 MHz, $CDCl_3$, 25 °C) of compound **4lh**

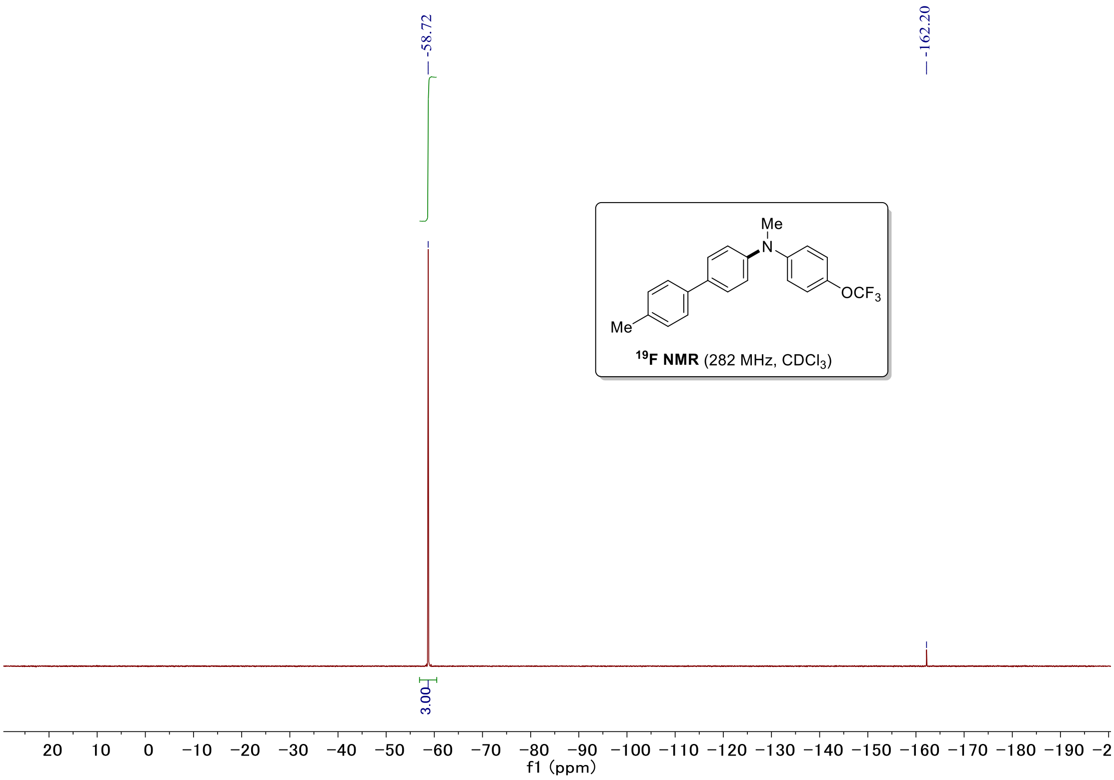

Supplementary Figure 189. ^{19}F NMR (282 MHz, $CDCl_3$, 25 °C) of compound **4lh**

4'-Methoxy-*N*-(4-methoxyphenyl)-*N*-methyl-[1,1'-biphenyl]-4-amine (4mf)

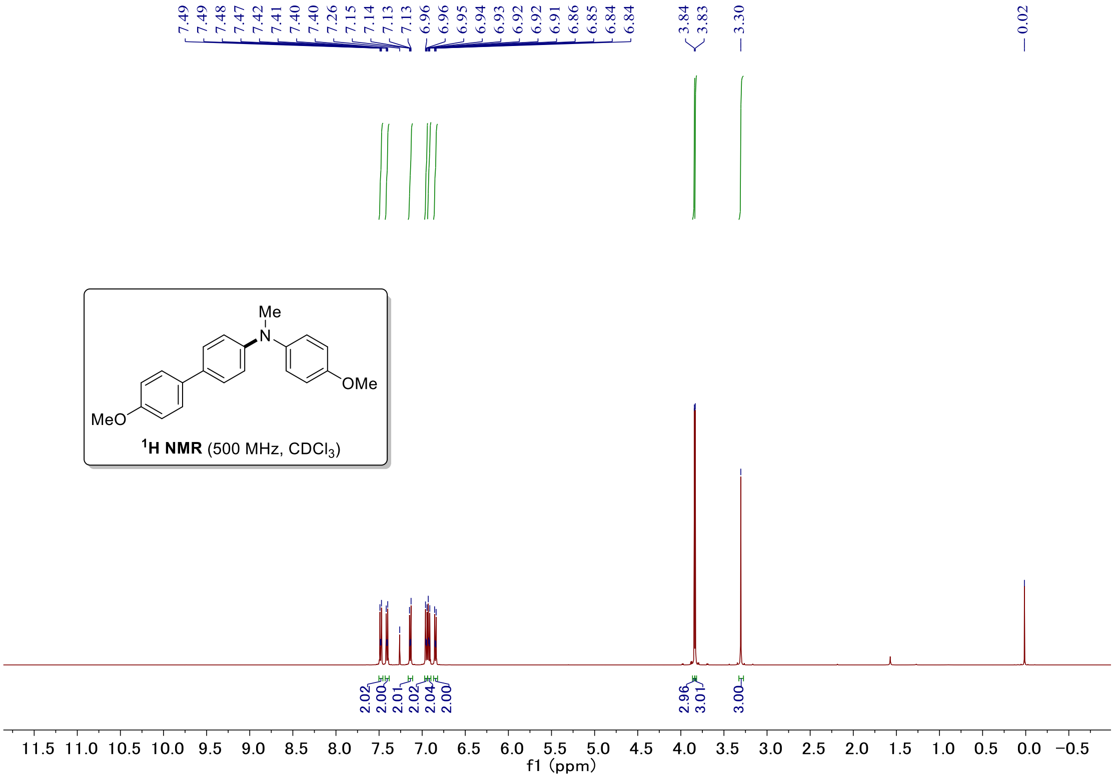

Supplementary Figure 190. ^{1}H NMR (500 MHz, $CDCl_3$, 25 °C) of compound **4mf**

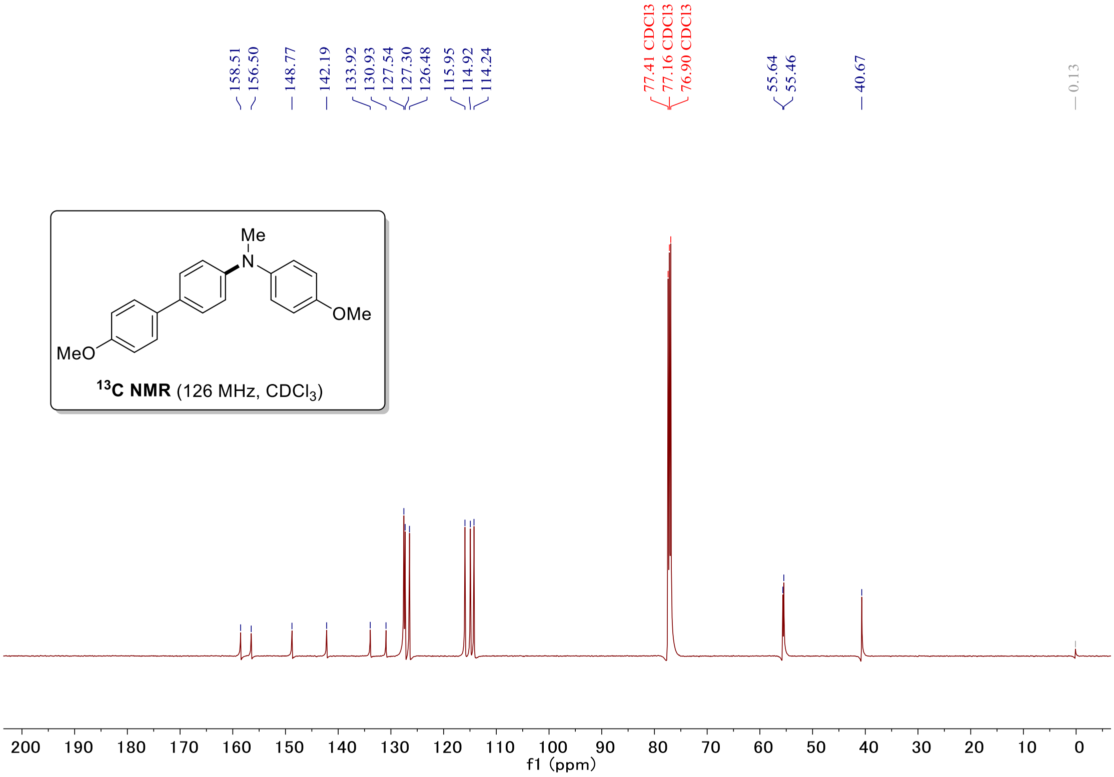

Supplementary Figure 191. ^{13}C NMR (126 MHz, $CDCl_3$, 25 °C) of compound **4mf**

4'-Methoxy-*N*-methyl-*N*-(4-(trifluoromethoxy)phenyl)-[1,1'-biphenyl]-4-amine (4mh)

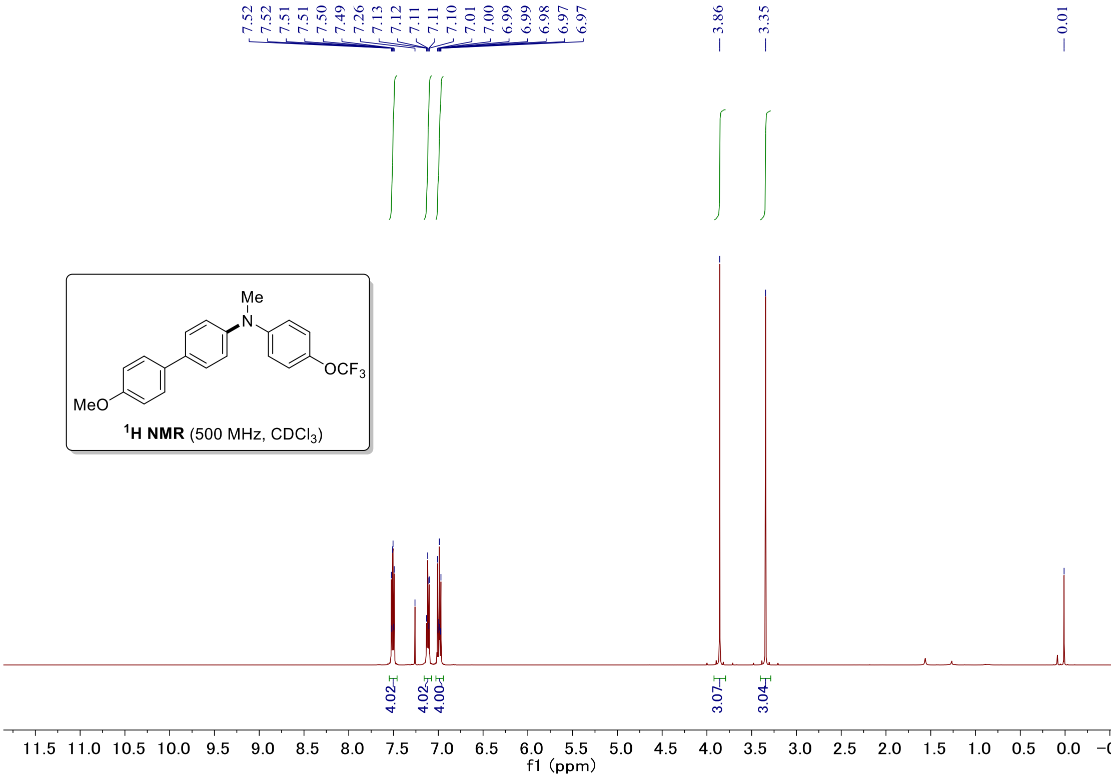

Supplementary Figure 192. ^{1}H NMR (500 MHz, $CDCl_3$, 25 °C) of compound **4mh**

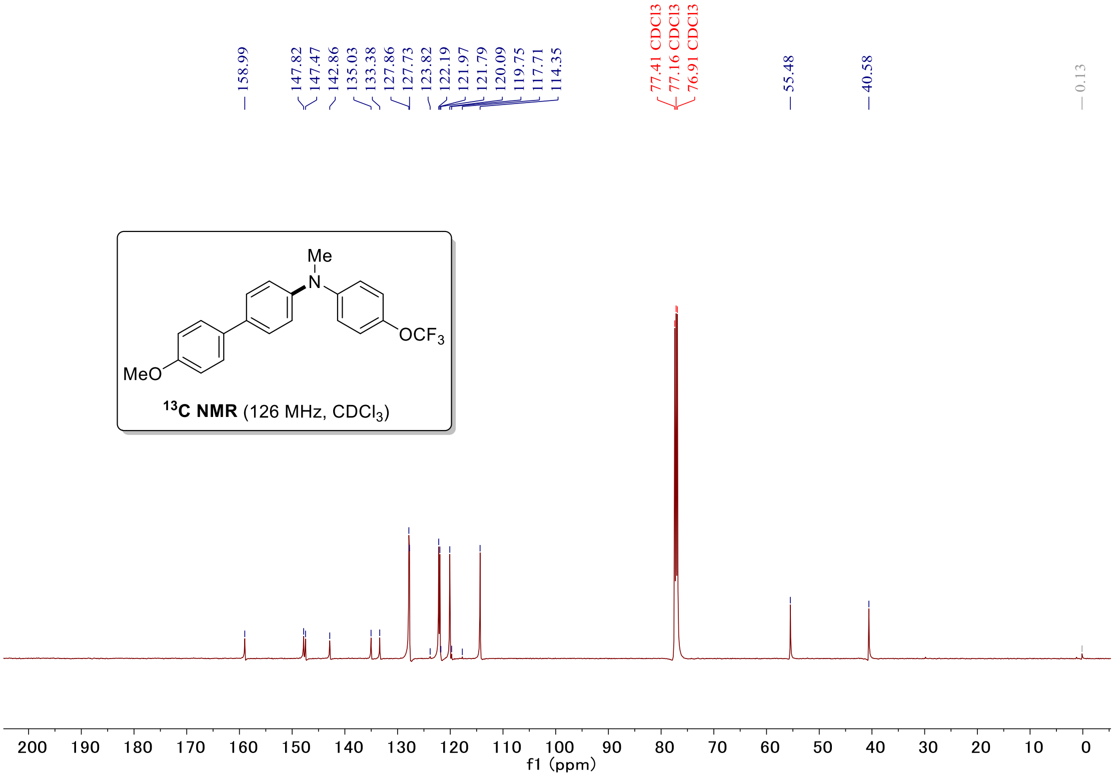

Supplementary Figure 193. ^{13}C NMR (126 MHz, $CDCl_3$, 25 °C) of compound **4mh**

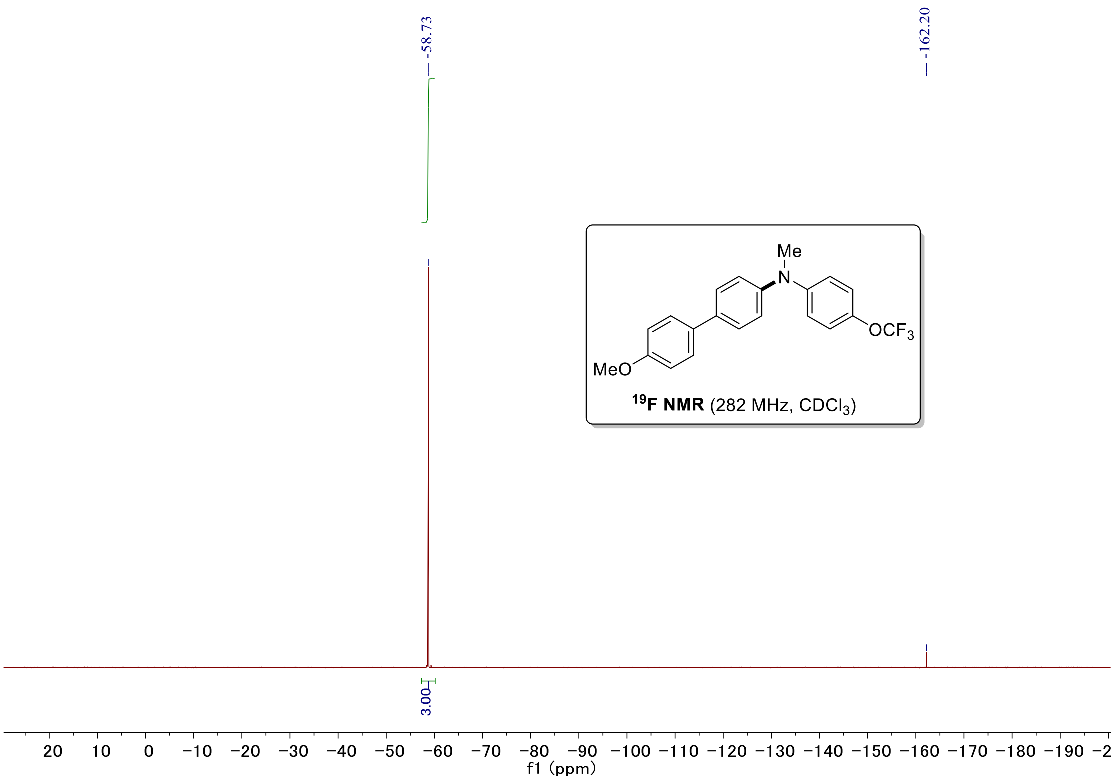

Supplementary Figure 194. ^{19}F NMR (282 MHz, $CDCl_3$, 25 °C) of compound **4mh**

4'-Chloro-*N*-(4-chlorophenyl)-*N*-methyl-[1,1'-biphenyl]-4-amine (4oi)

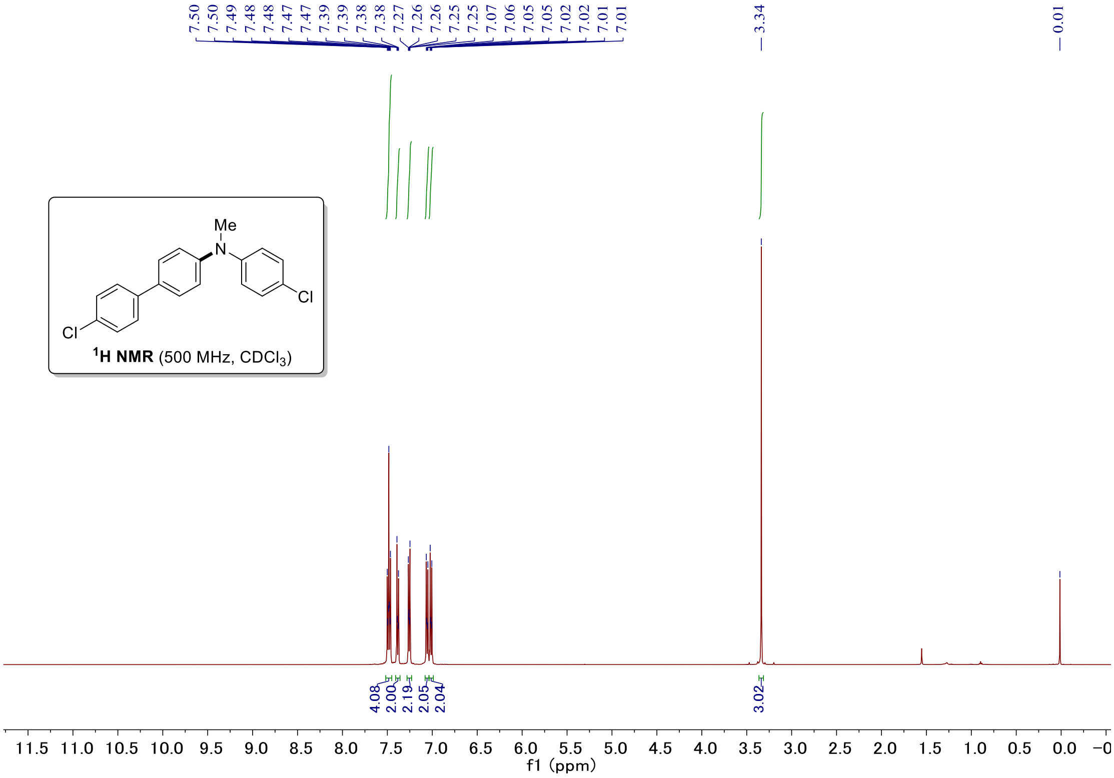

Supplementary Figure 195. ^{1}H NMR (500 MHz, $CDCl_3$, 25 °C) of compound **4oi**

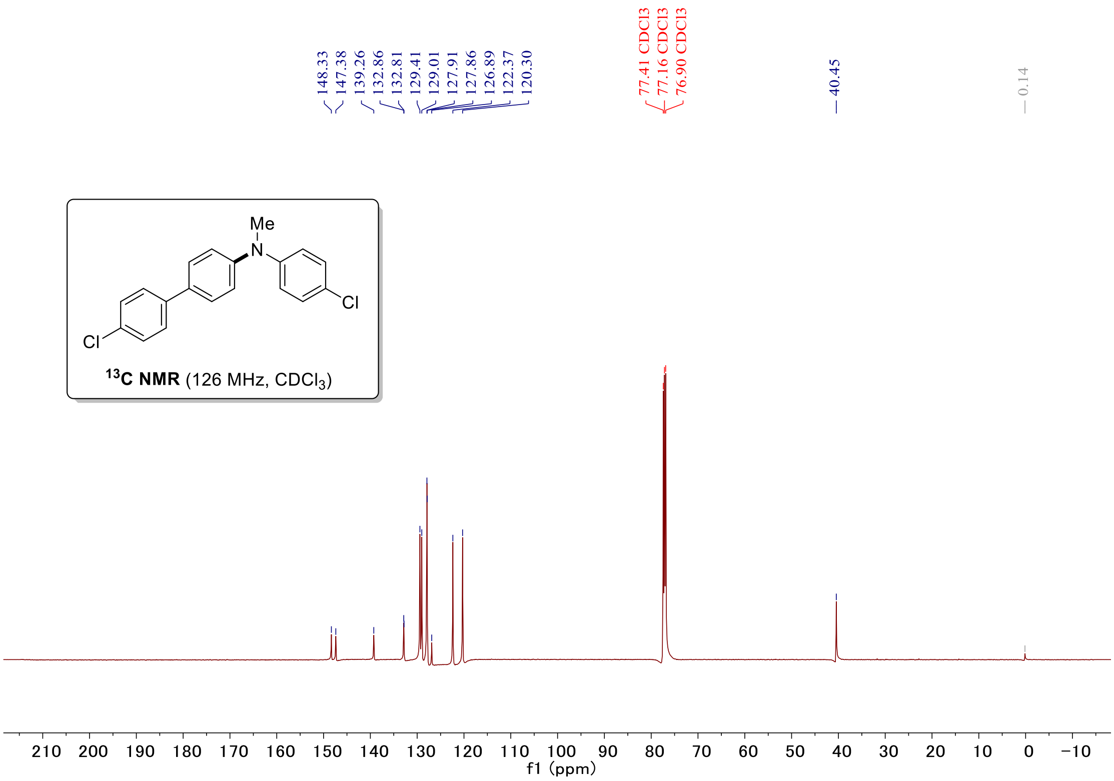

Supplementary Figure 196. ^{13}C NMR (126 MHz, $CDCl_3$, 25 ^{o}C) of compound **4oi**

4'-((4-Chlorophenyl)(methyl)amino)-[1,1'-biphenyl]-4-carbonitrile (4qi)

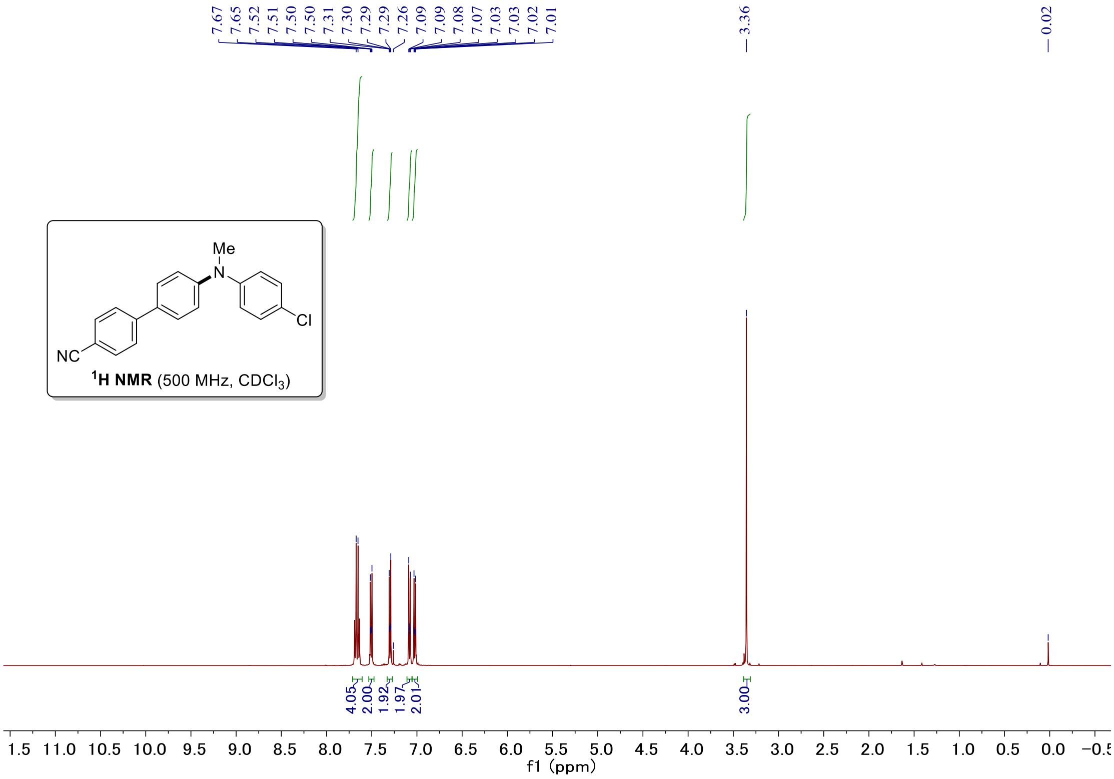

Supplementary Figure 197. ^{1}H NMR (500 MHz, $CDCl_3$, 25 ^{o}C) of compound **4qi**

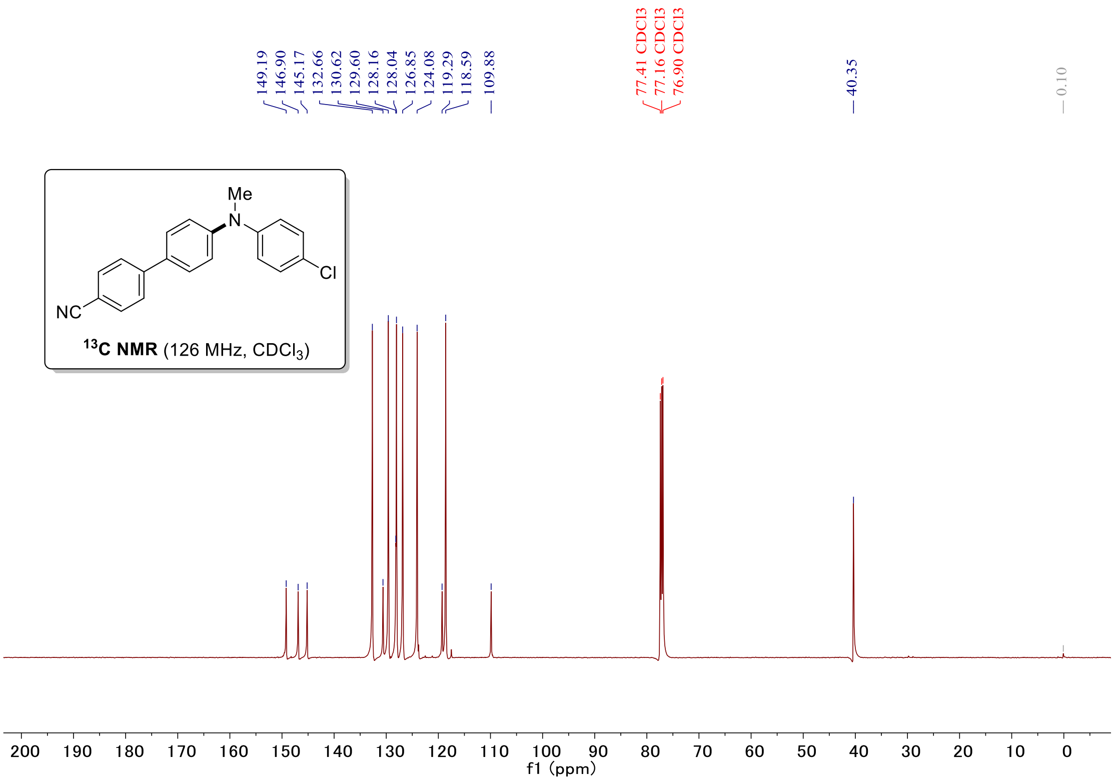

Supplementary Figure 198. ^{13}C NMR (126 MHz, $CDCl_3$, 25 °C) of compound **4qi**

***N*-(4-Methoxyphenyl)-*N*-methyl-4'-(trifluoromethyl)-[1,1'-biphenyl]-4-amine (4rf)**

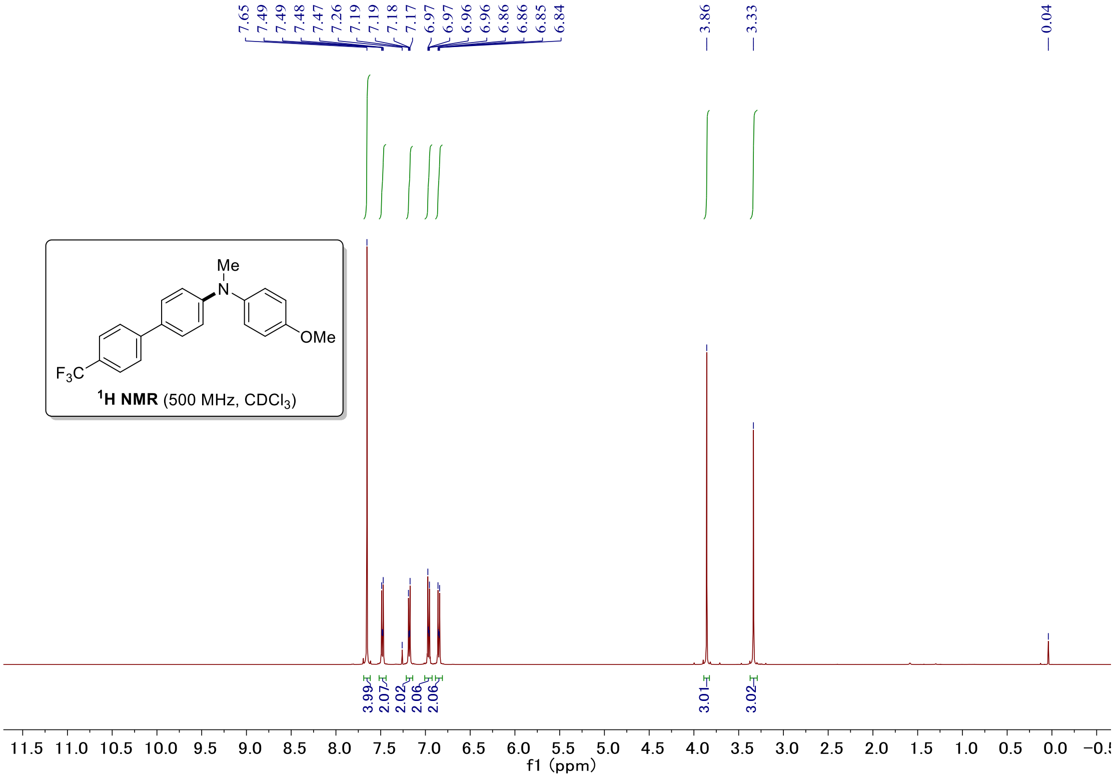

Supplementary Figure 199. ^{1}H NMR (500 MHz, $CDCl_3$, 25 °C) of compound **4rf**

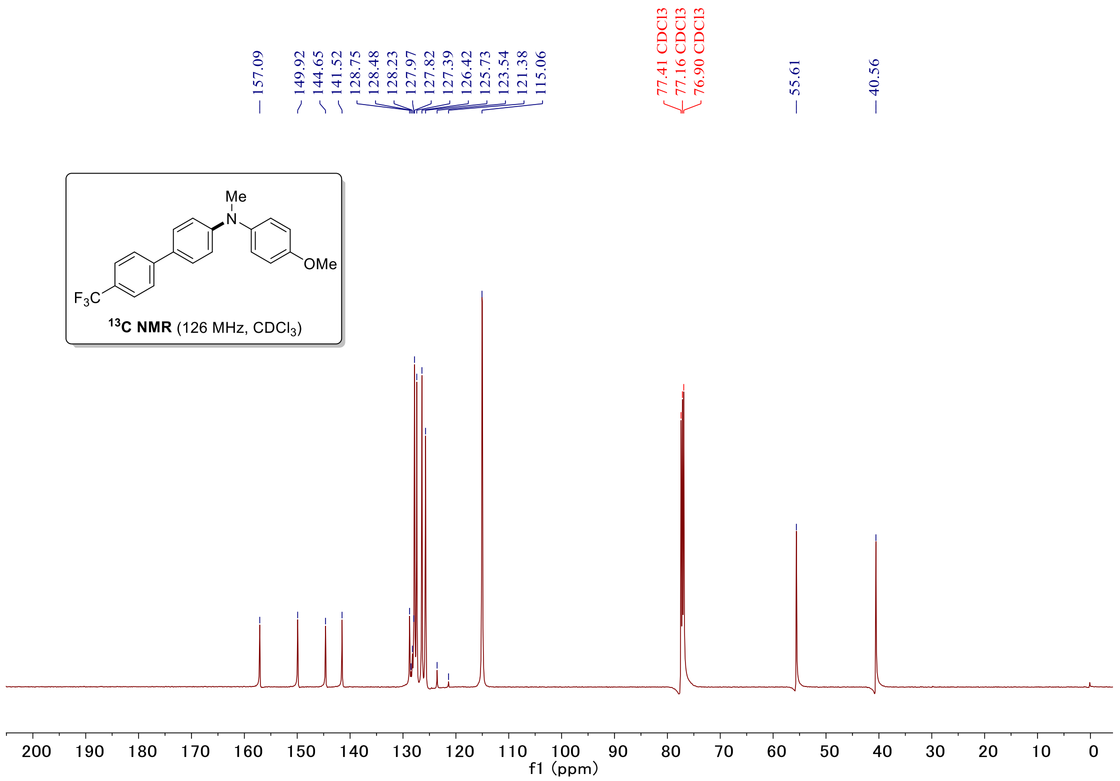

Supplementary Figure 200. ^{13}C NMR (126 MHz, $CDCl_3$, 25 ^{o}C) of compound **4rf**

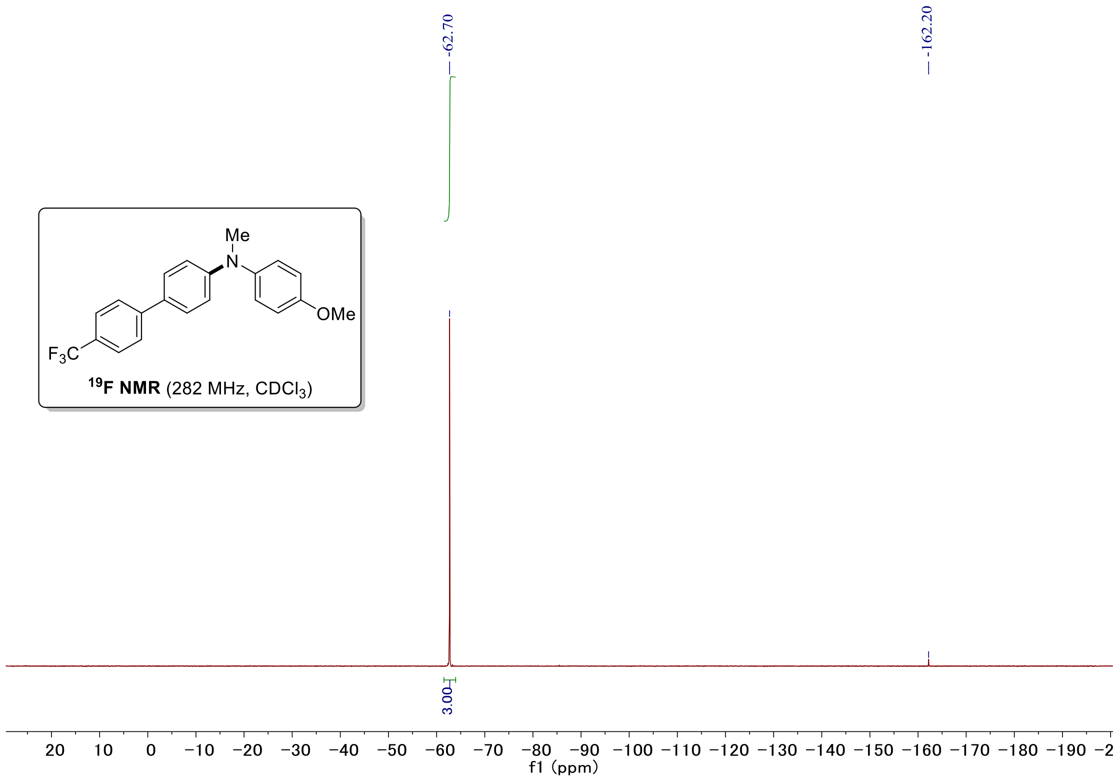

Supplementary Figure 201. ^{19}F NMR (282 MHz, $CDCl_3$, 25 ^{o}C) of compound **4rf**

N-Methyl-*N*-(4-(trifluoromethoxy)phenyl)-4'-(trifluoromethyl)-[1,1'-biphenyl]-4-amine (4rh)

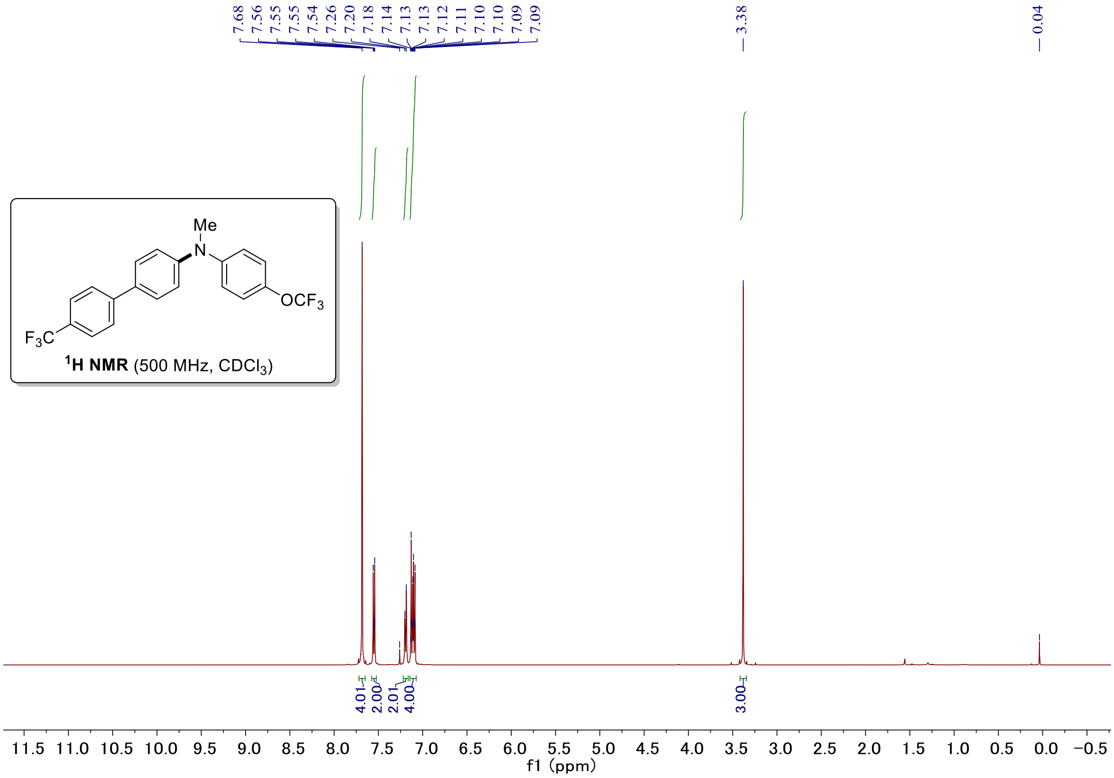

Supplementary Figure 202. ^{1}H NMR (500 MHz, $CDCl_3$, 25 °C) of compound **4rh**

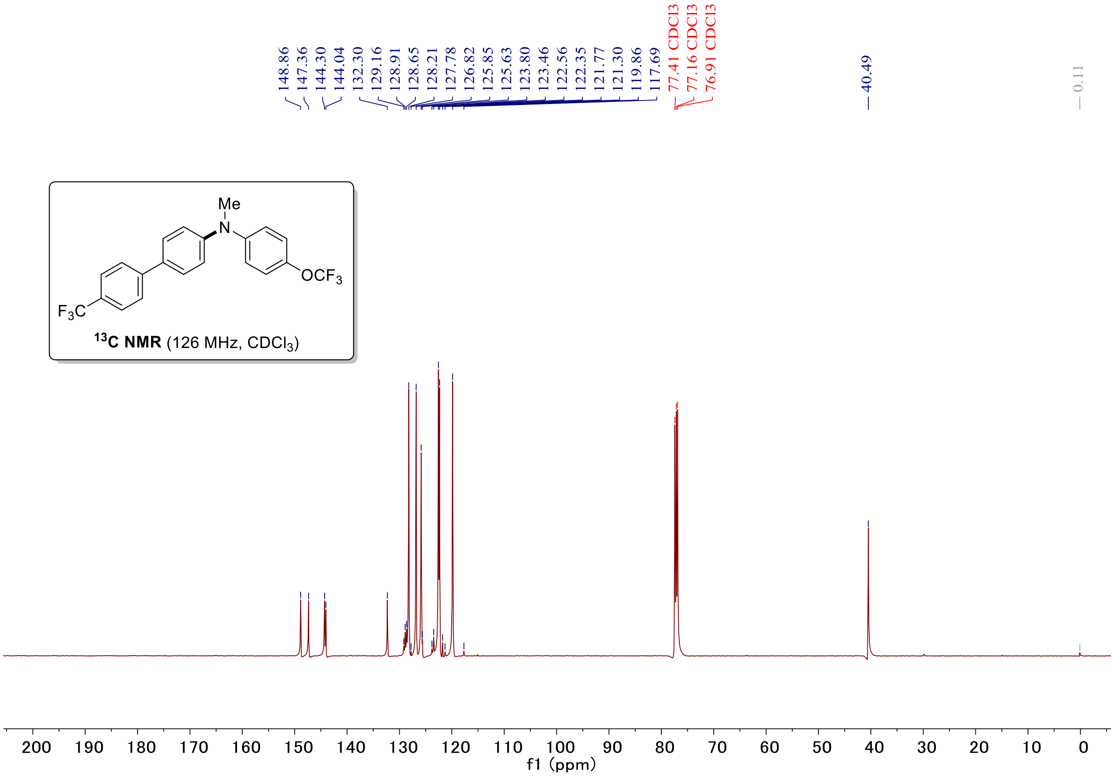

Supplementary Figure 203. ^{13}C NMR (126 MHz, $CDCl_3$, 25 °C) of compound **4rh**

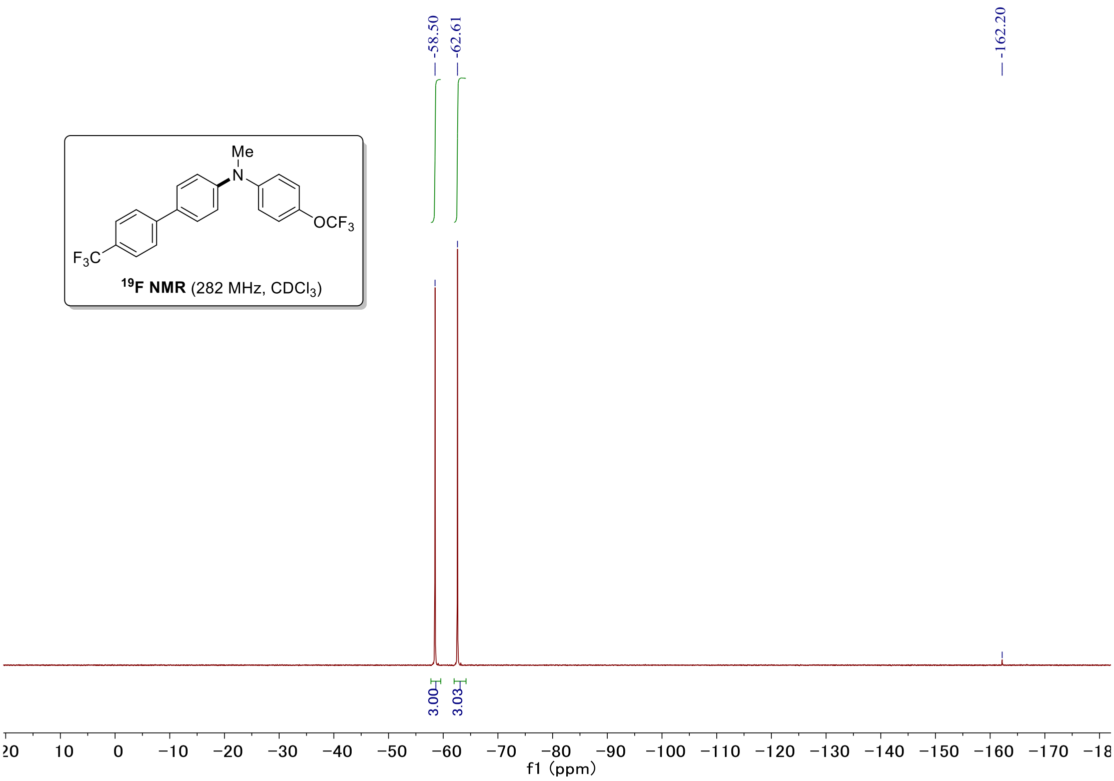

Supplementary Figure 204. ^{19}F NMR (282 MHz, $CDCl_3$, 25 ^{o}C) of compound **4rh**

N-(4-Chlorophenyl)-*N*-methyl-3'-(trifluoromethyl)-[1,1'-biphenyl]-4-amine (4si)

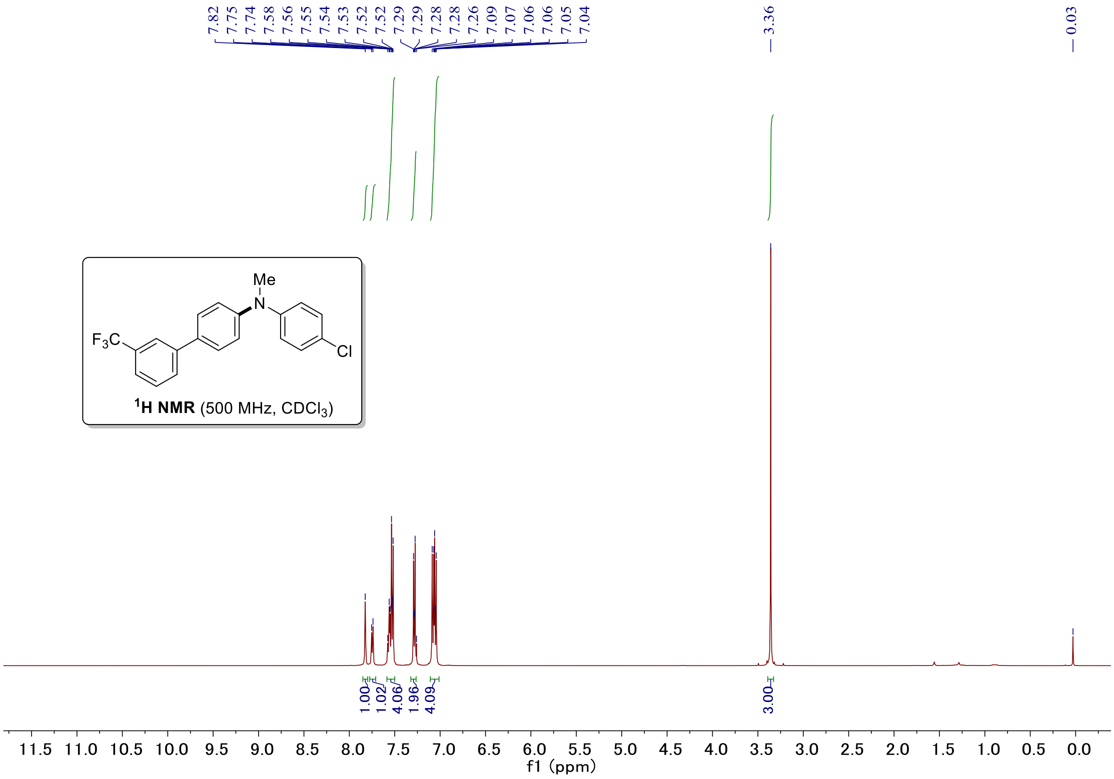

Supplementary Figure 205. ^{1}H NMR (500 MHz, $CDCl_3$, 25 ^{o}C) of compound **4si**

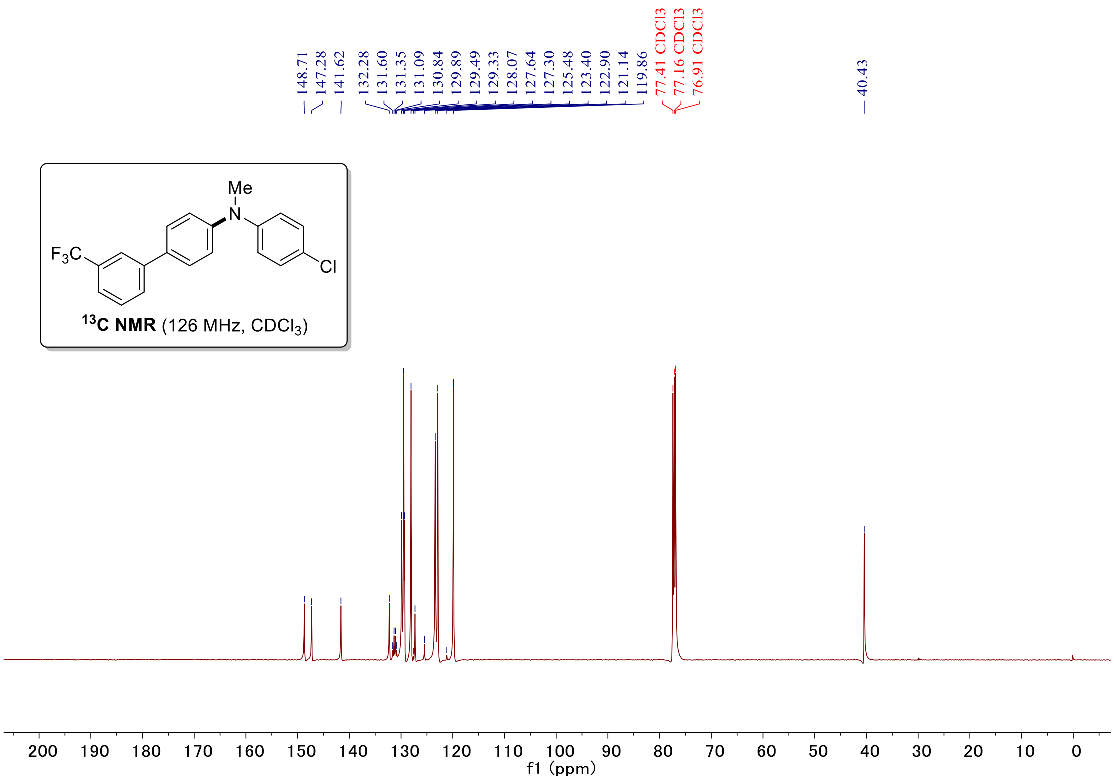

Supplementary Figure 206. ^{13}C NMR (126 MHz, $CDCl_3$, 25 °C) of compound **4si**

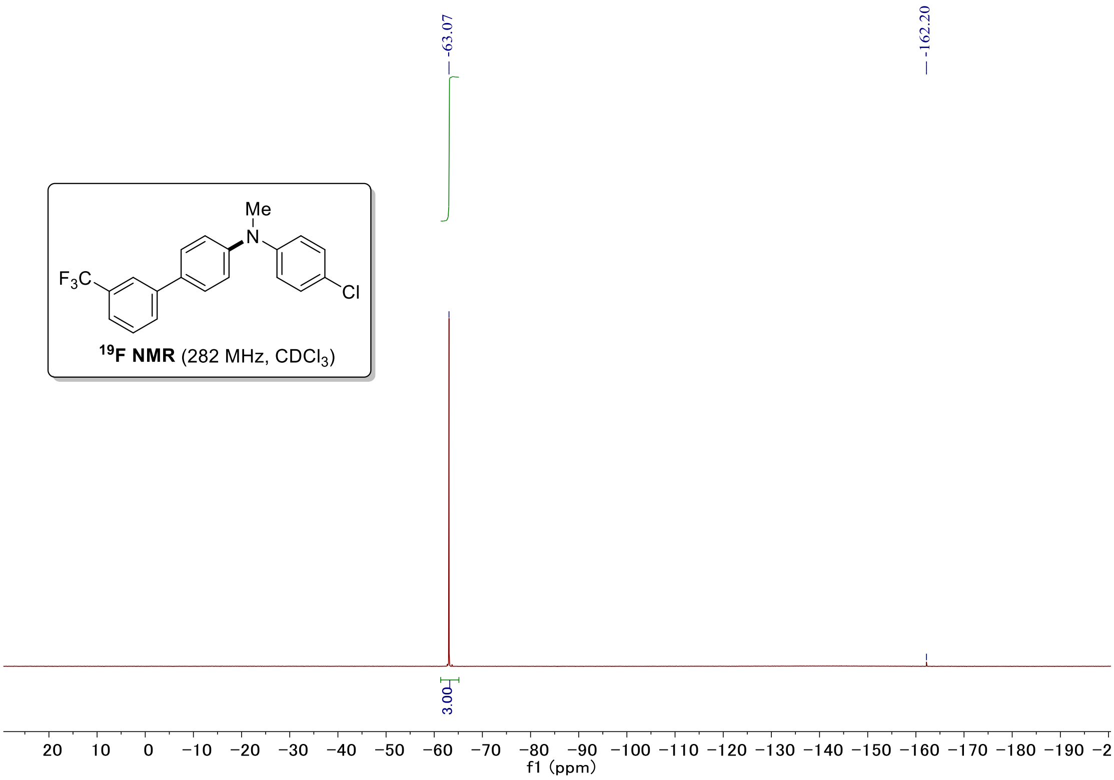

Supplementary Figure 207. ^{19}F NMR (282 MHz, $CDCl_3$, 25 °C) of compound **4si**

_N_-(3-Chlorophenyl)-_N_-methyl-3',5'-bis(trifluoromethyl)-[1,1'-biphenyl]-4-amine (4tj)

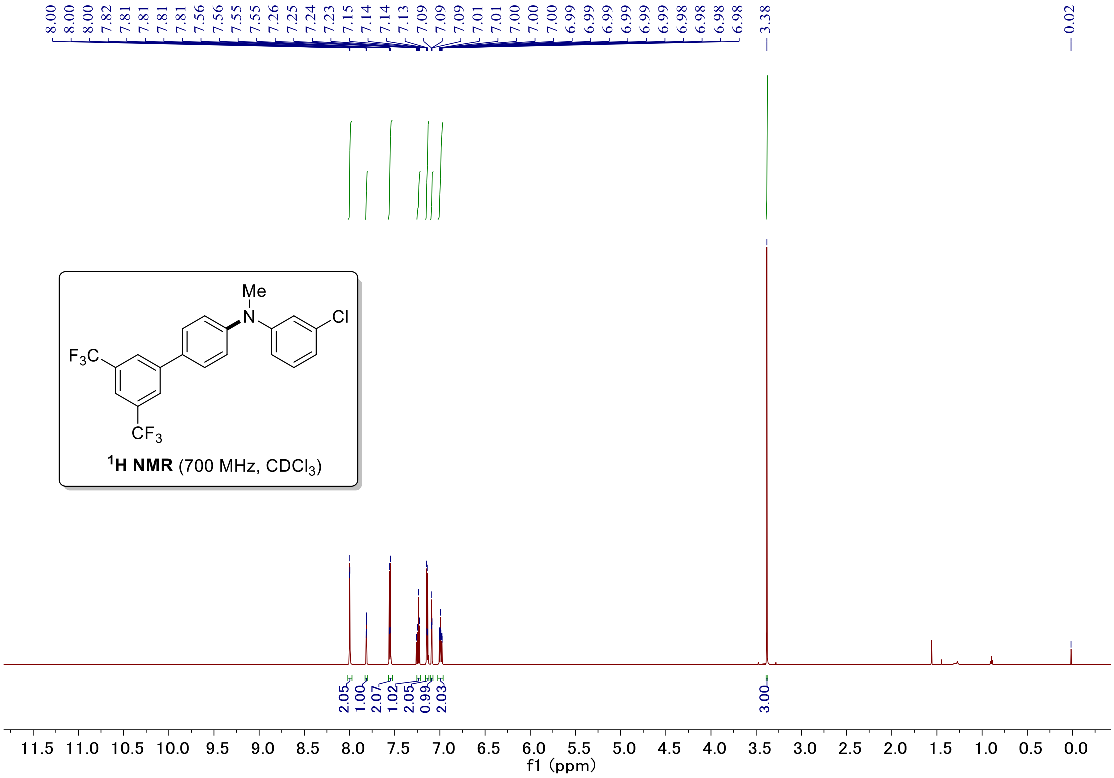

Supplementary Figure 208. ^{1}H NMR (700 MHz, $CDCl_3$, 25 °C) of compound **4tj**

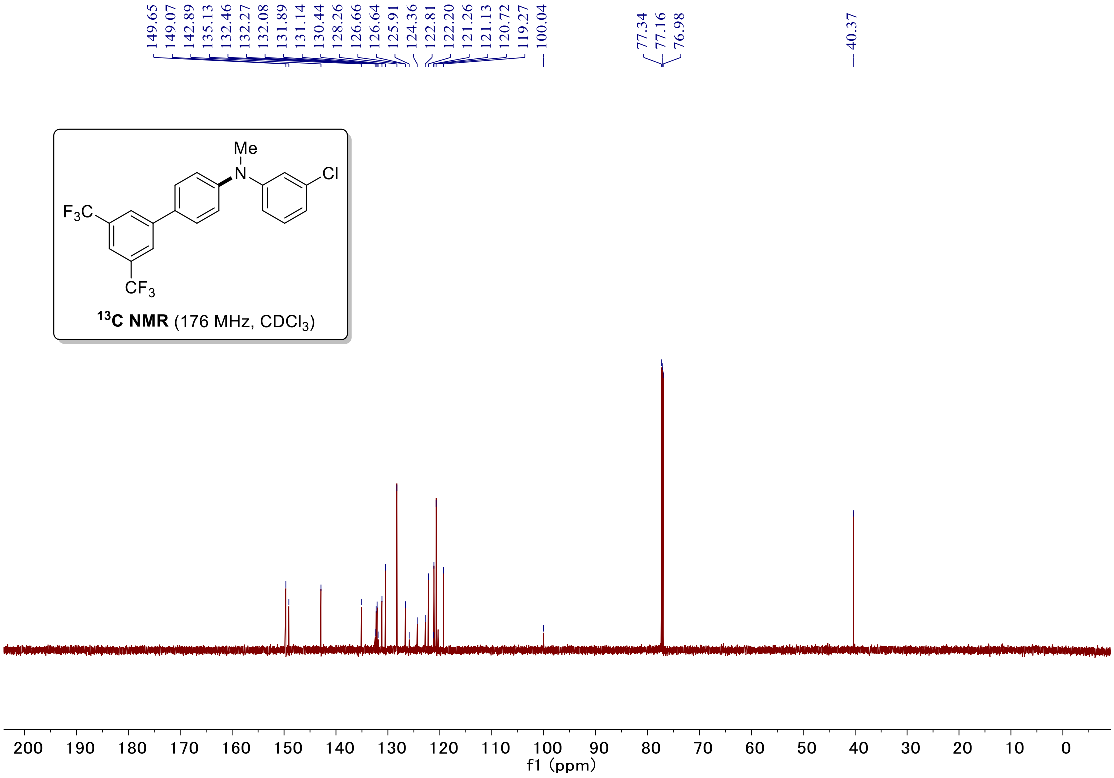

Supplementary Figure 209. ^{13}C NMR (176 MHz, $CDCl_3$, 25 °C) of compound **4tj**

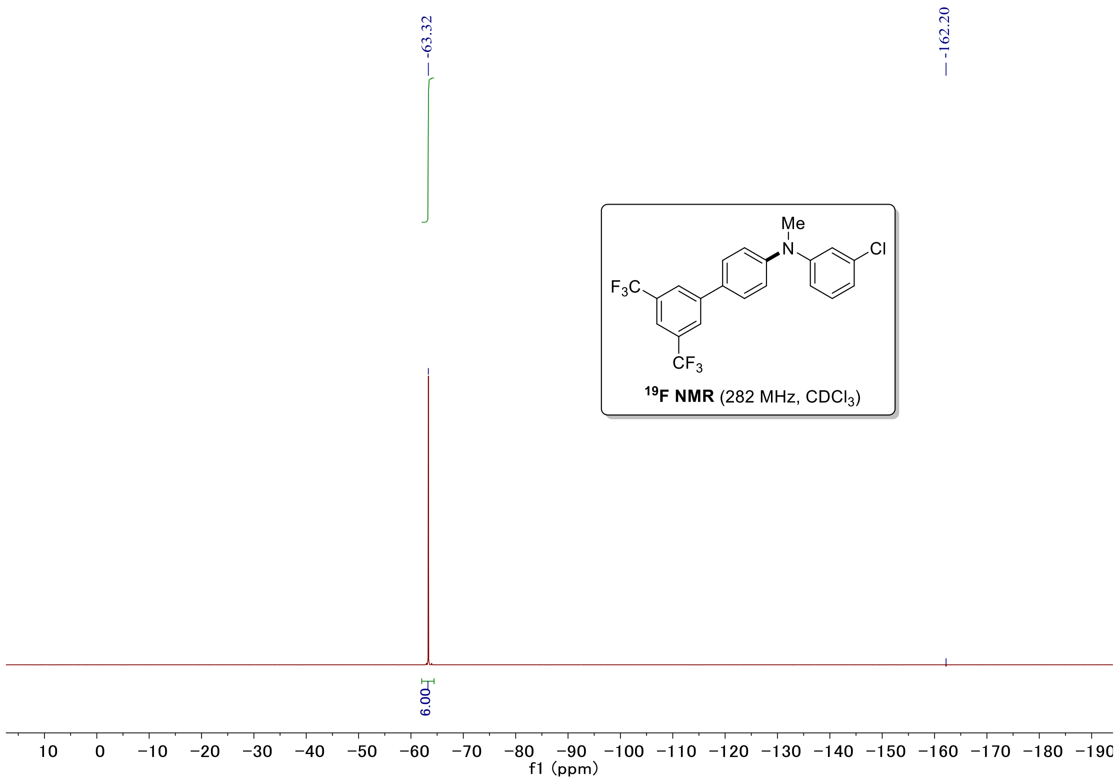

Supplementary Figure 210. ^{19}F NMR (282 MHz, $CDCl_3$, 25 °C) of compound **4tj**

_N_-(4-Chlorophenyl)-_N_-methyl-6-phenylpyridin-3-amine (4vi)

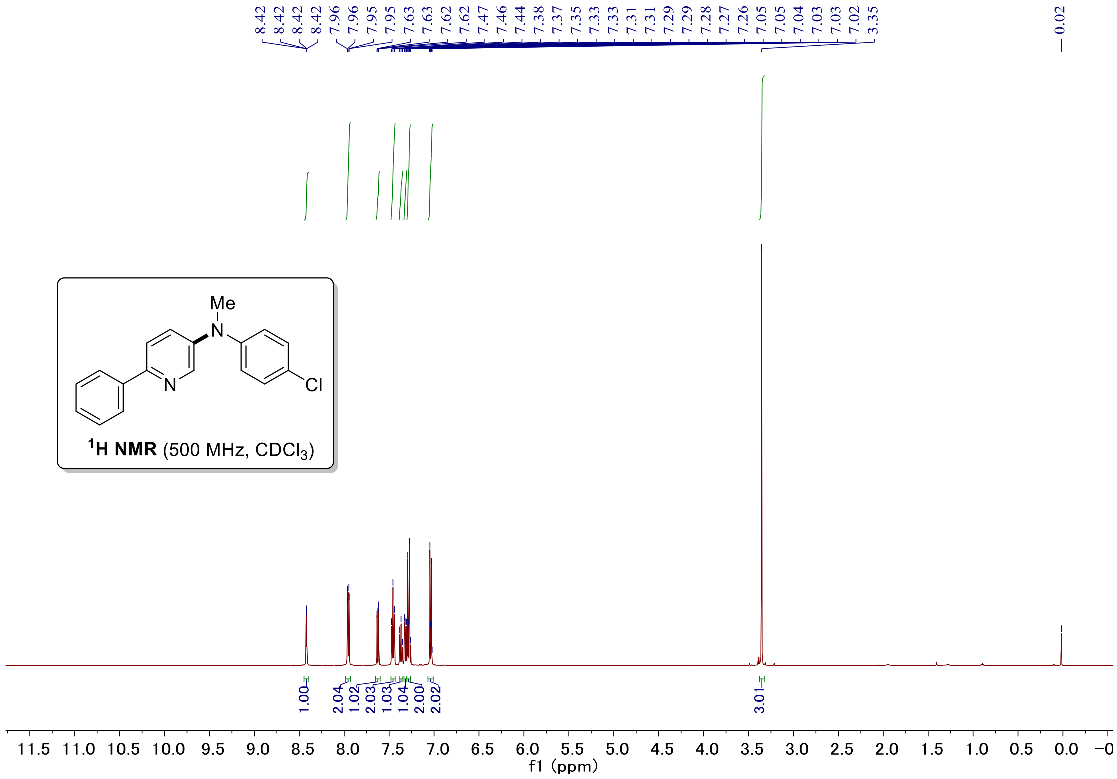

Supplementary Figure 211. ^{1}H NMR (500 MHz, $CDCl_3$, 25 °C) of compound **4vi**

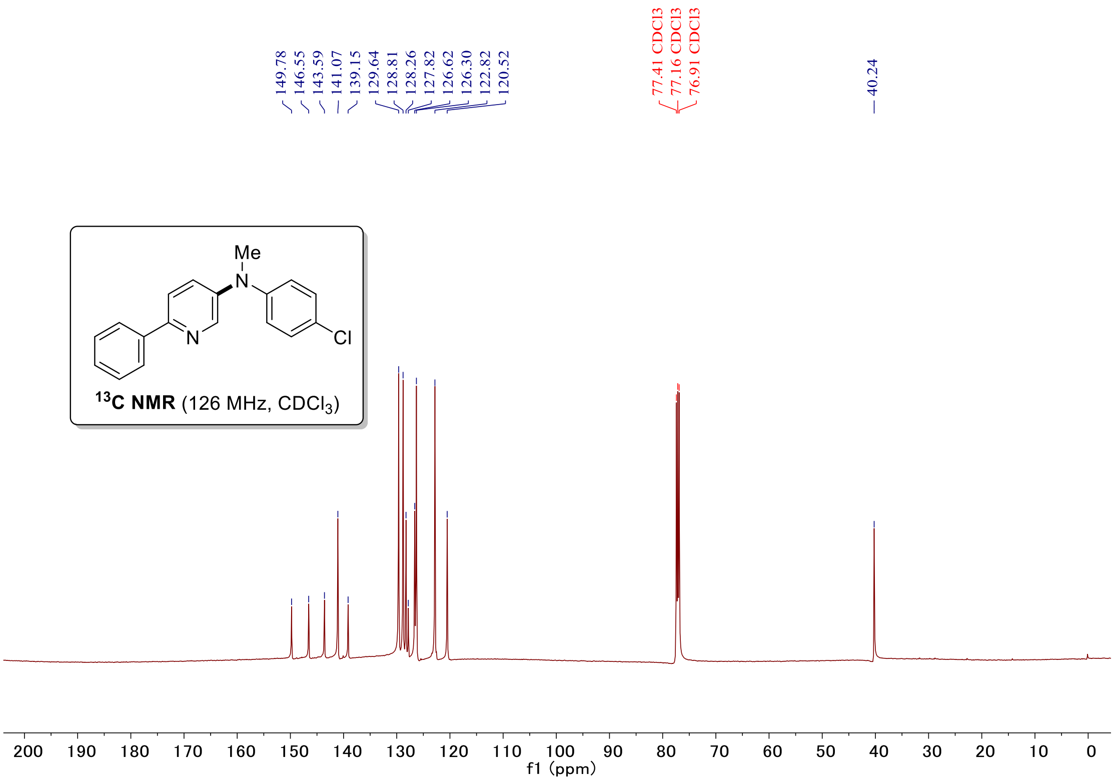

Supplementary Figure 212. ^{13}C NMR (126 MHz, $CDCl_3$, 25 °C) of compound **4vi**

3-Chloro-*N*-methyl-*N*-(4-(pyridin-4-yl)phenyl)aniline (4wj)

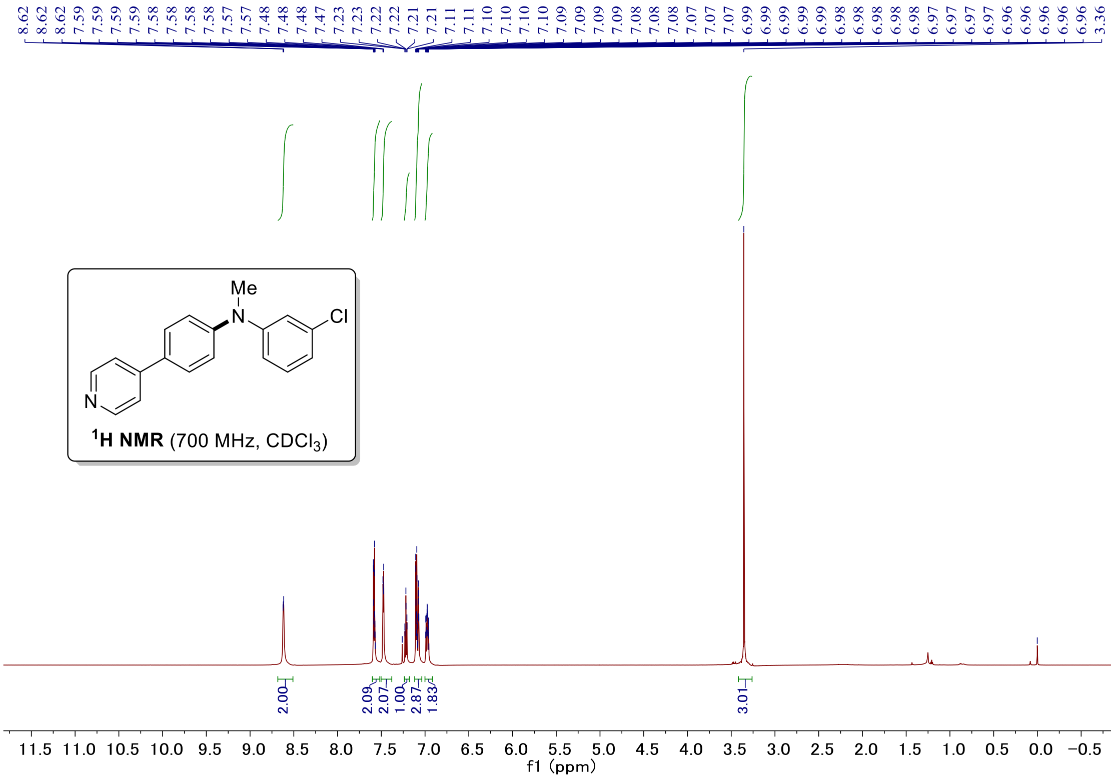

Supplementary Figure 213. ^{1}H NMR (700 MHz, $CDCl_3$, 25 °C) of compound **4wj**

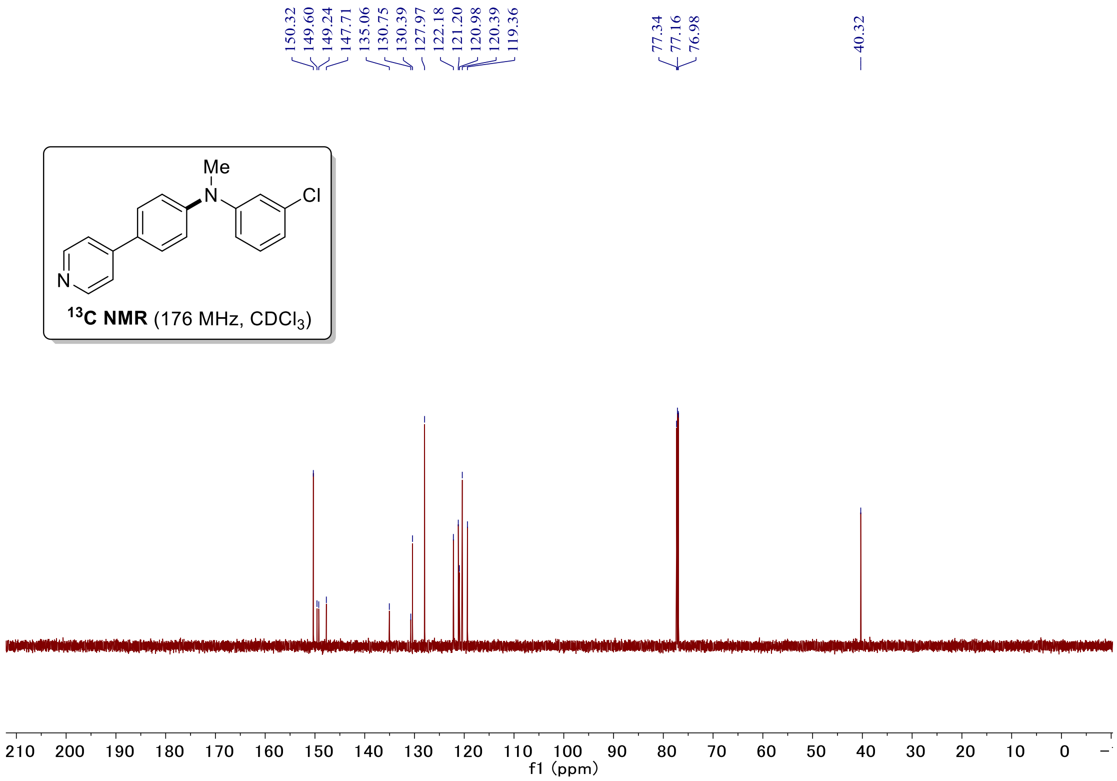

Supplementary Figure 214. ^{13}C NMR (176 MHz, $CDCl_3$, 25 °C) of compound **4wj**

***N*-(4-(1*H*-Pyrrol-1-yl)phenyl)-*N*-methylbenzo[*d*][1,3]dioxol-5-amine (4zg)**

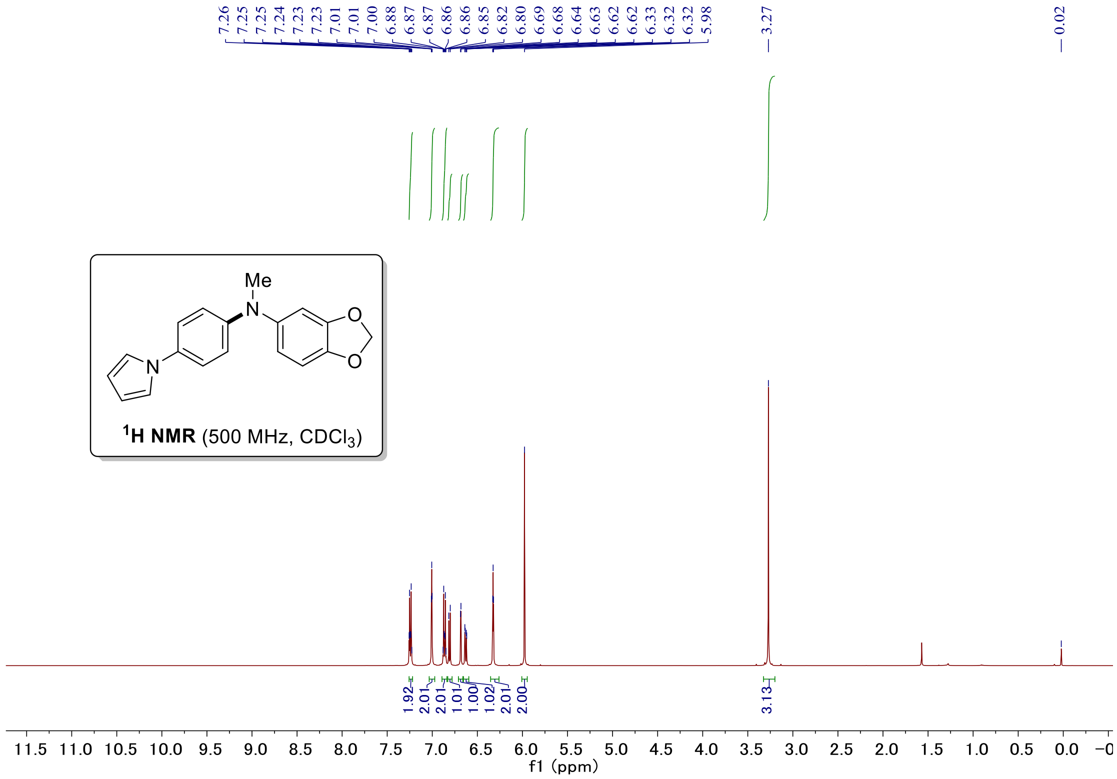

Supplementary Figure 215. ^{1}H NMR (500 MHz, $CDCl_3$, 25 °C) of compound **4zg**

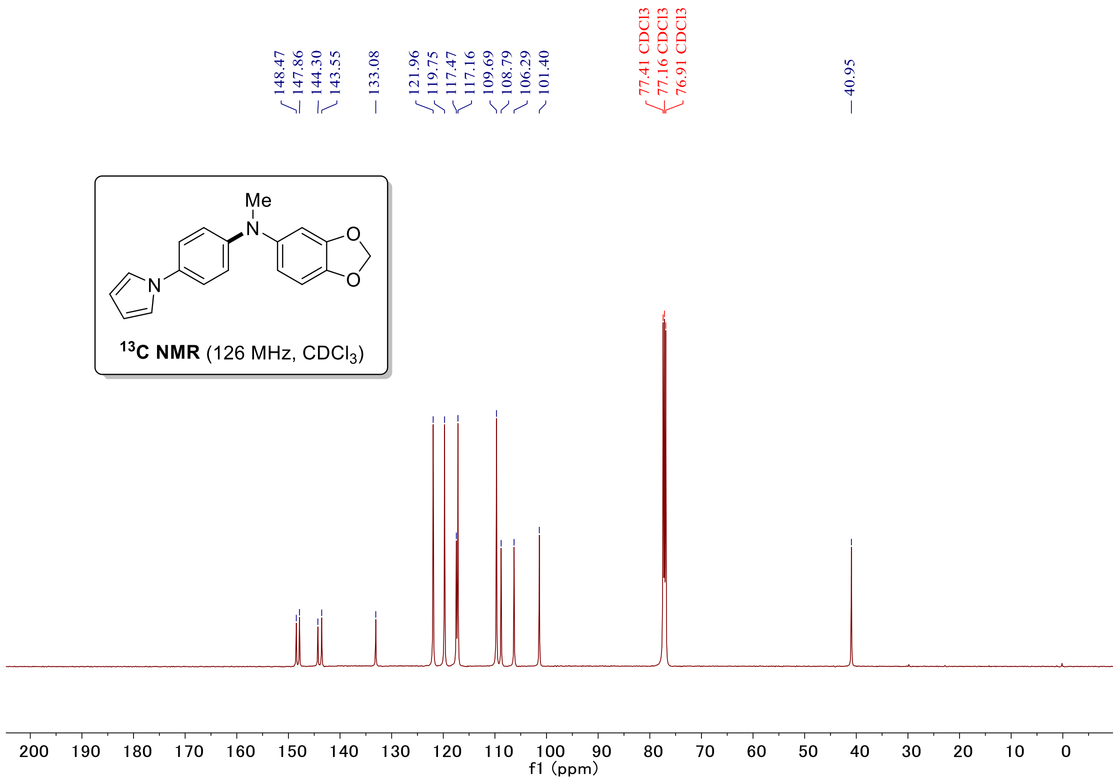

Supplementary Figure 216. ^{13}C NMR (126 MHz, $CDCl_3$, 25 °C) of compound **4zg**

***N*-Methyl-*N*-(4-(1-methyl-1*H*-indol-2-yl)phenyl)benzo[*d*][1,3]dioxol-5-amine (4abg)**

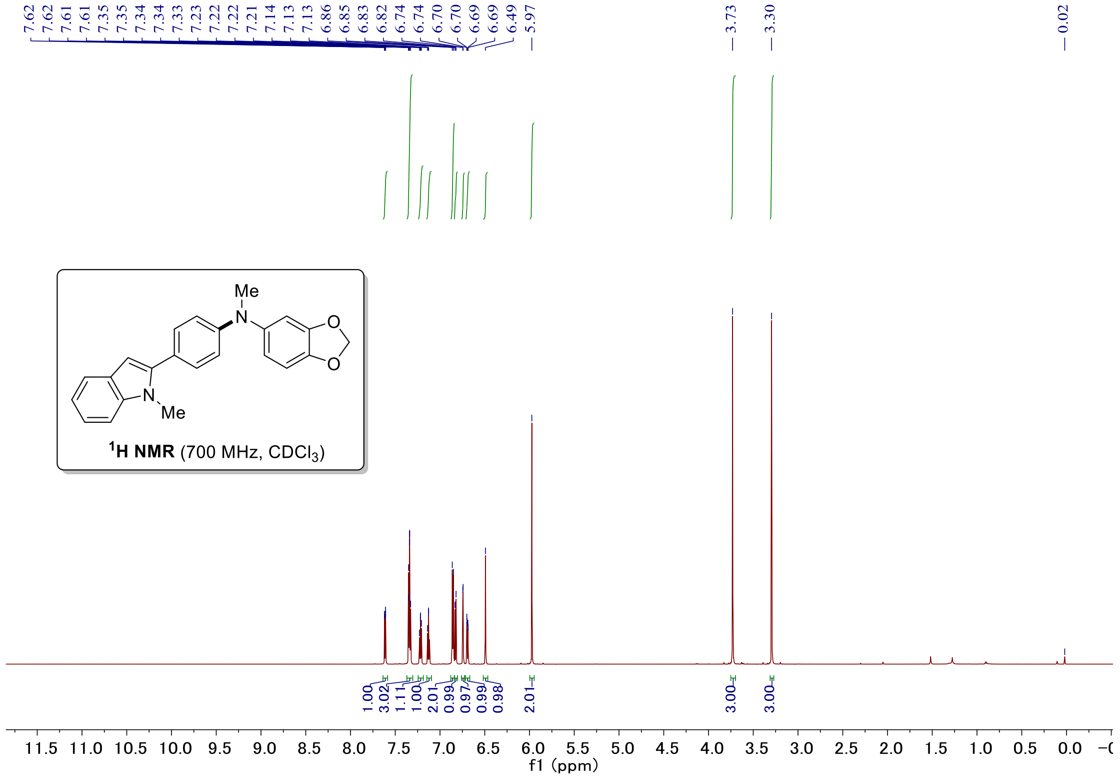

Supplementary Figure 217. ^{1}H NMR (700 MHz, $CDCl_3$, 25 °C) of compound **4abg**

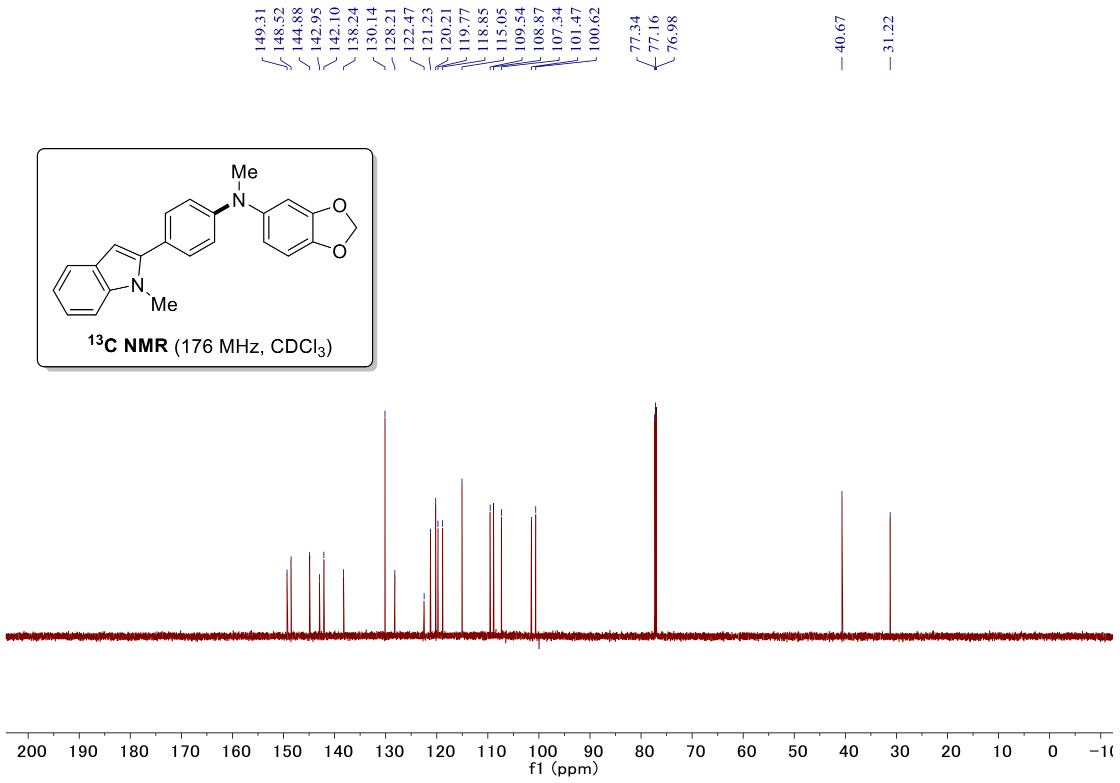

Supplementary Figure 218. ^{13}C NMR (176 MHz, $CDCl_3$, 25 °C) of compound **4abg**

_N_-Ethyl-_N_-phenyl-[1,1'-biphenyl]-4-amine (4al)

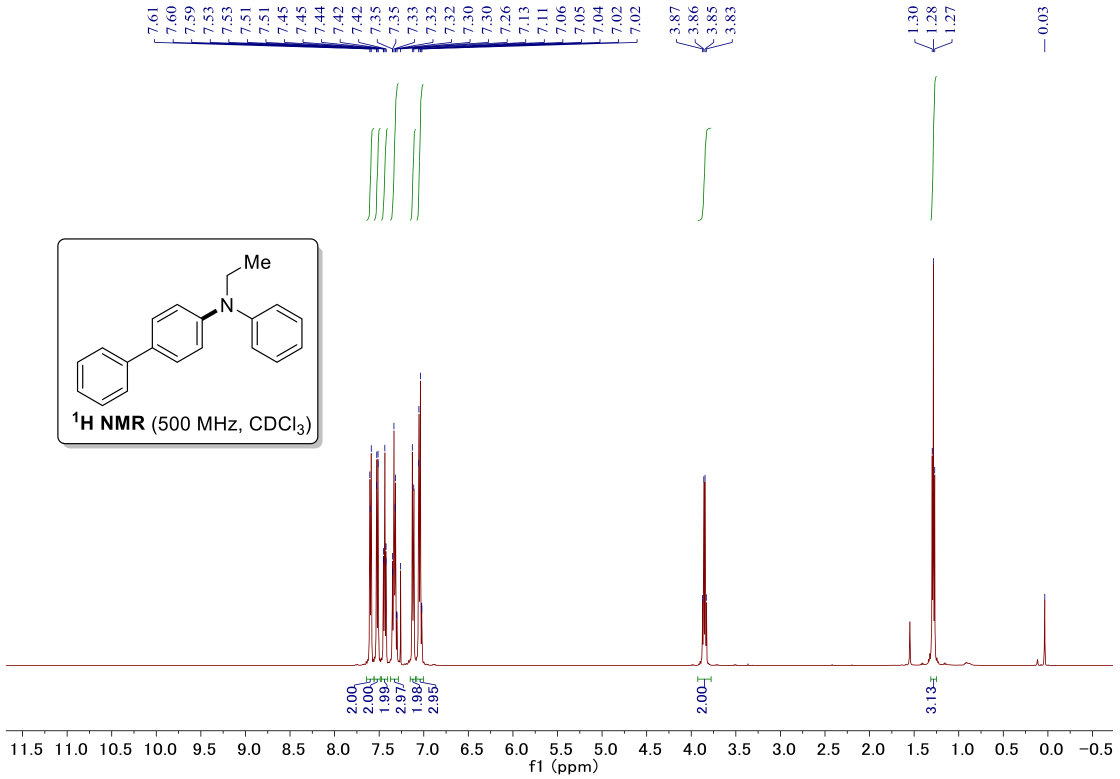

Supplementary Figure 219. ^{1}H NMR (500 MHz, $CDCl_3$, 25 °C) of compound **4al**

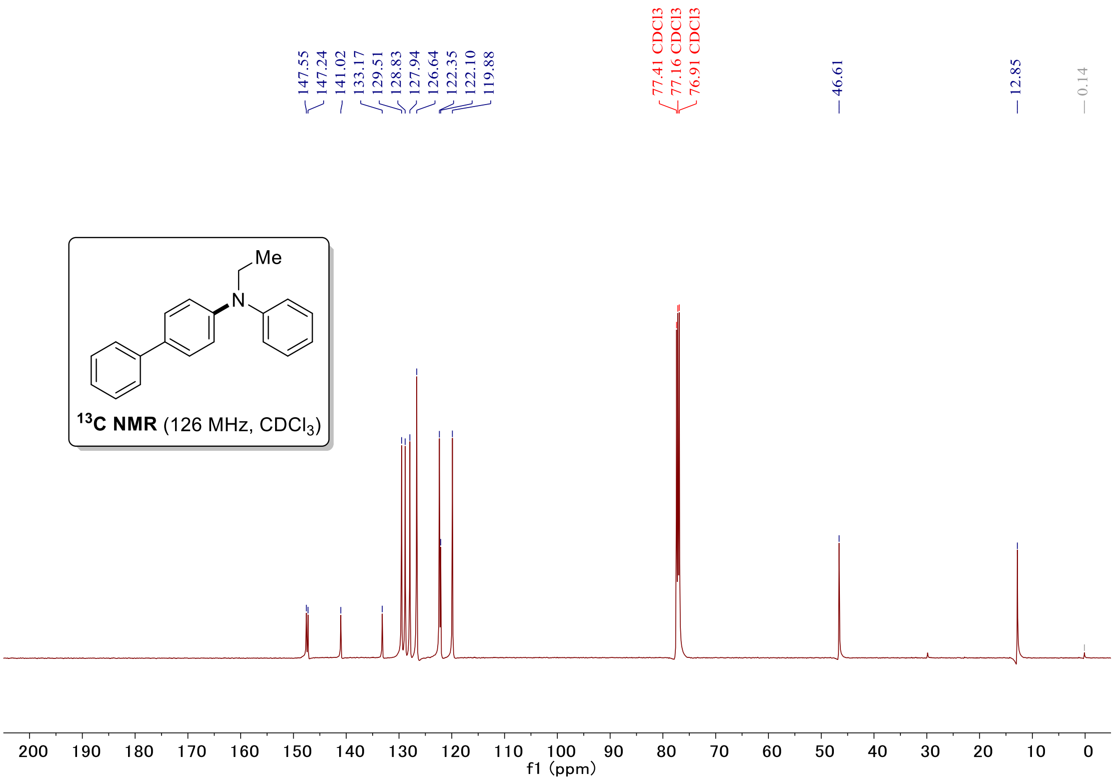

Supplementary Figure 220. ^{13}C NMR (126 MHz, $CDCl_3$, 25 °C) of compound **4al**

***N*-Butyl-*N*-phenyl-[1,1'-biphenyl]-4-amine (4am)**

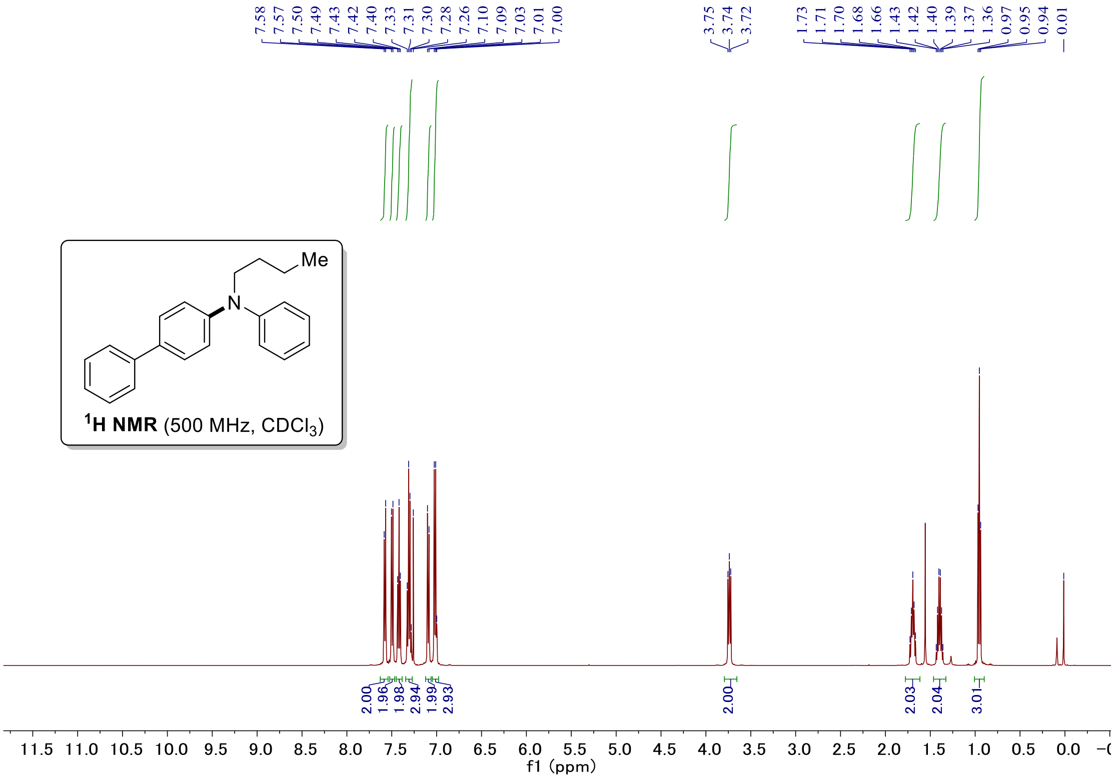

Supplementary Figure 221. ^{1}H NMR (500 MHz, $CDCl_3$, 25 °C) of compound **4am**

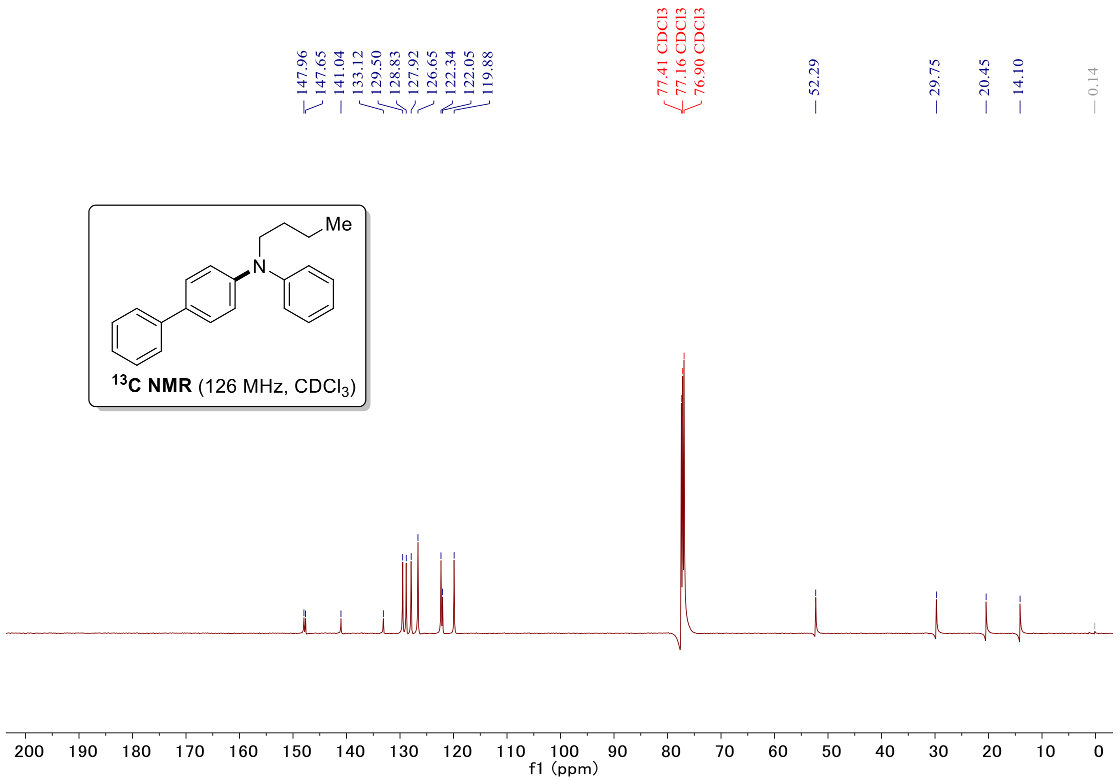

Supplementary Figure 222. ^{13}C NMR (126 MHz, $CDCl_3$, 25 °C) of compound **4am**

1-([1,1'-Biphenyl]-4-yl)indoline (4an)

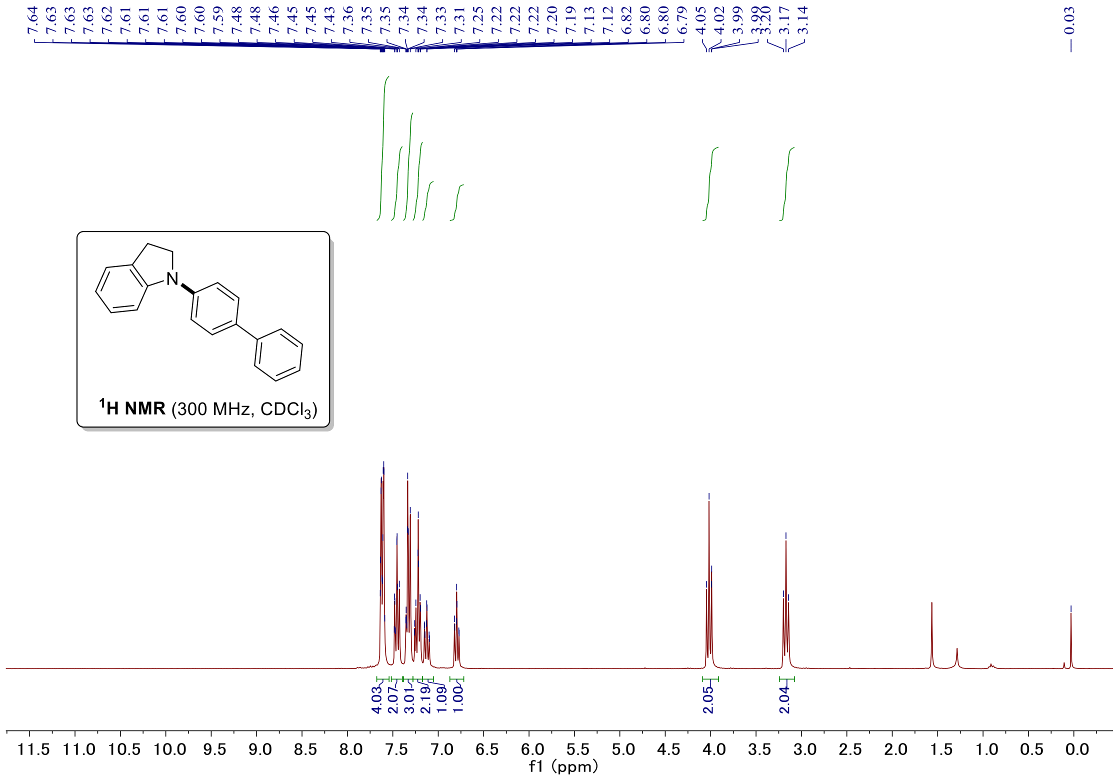

Supplementary Figure 223. ^{1}H NMR (300 MHz, $CDCl_3$, 25 °C) of compound **4an**

1-([1,1'-Biphenyl]-4-yl)-2-methylindoline (4ao)

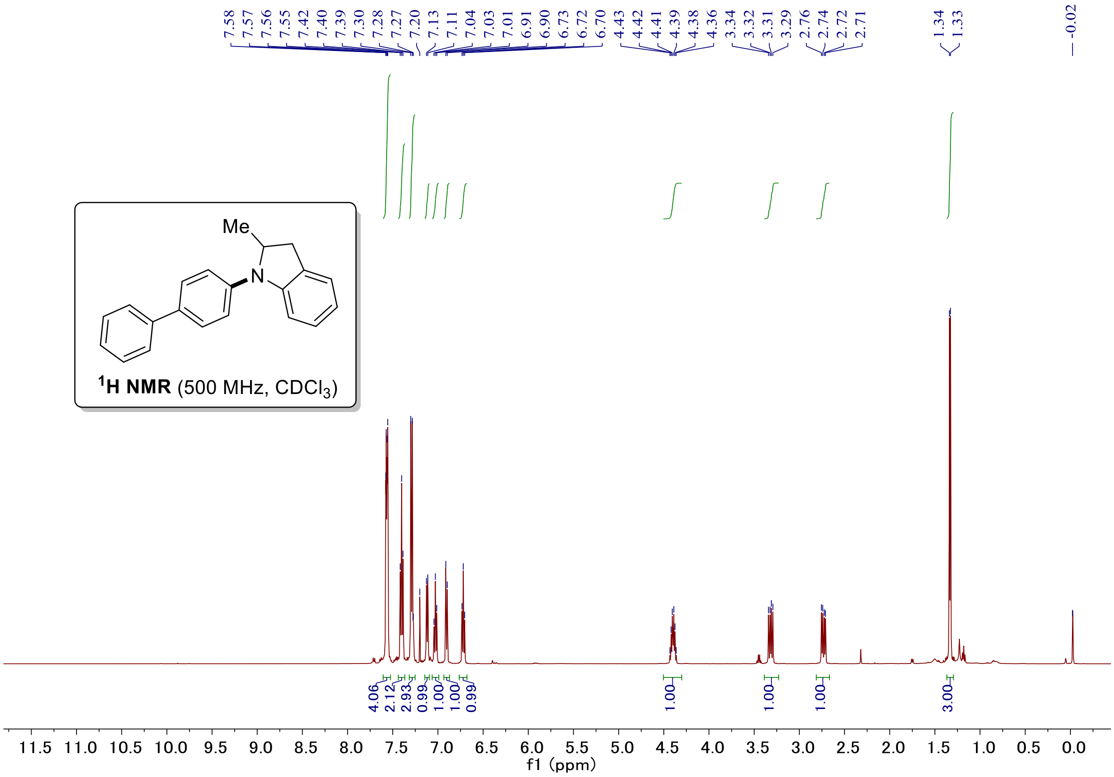

Supplementary Figure 224. ^{1}H NMR (500 MHz, $CDCl_3$, 25 °C) of compound **4ao**

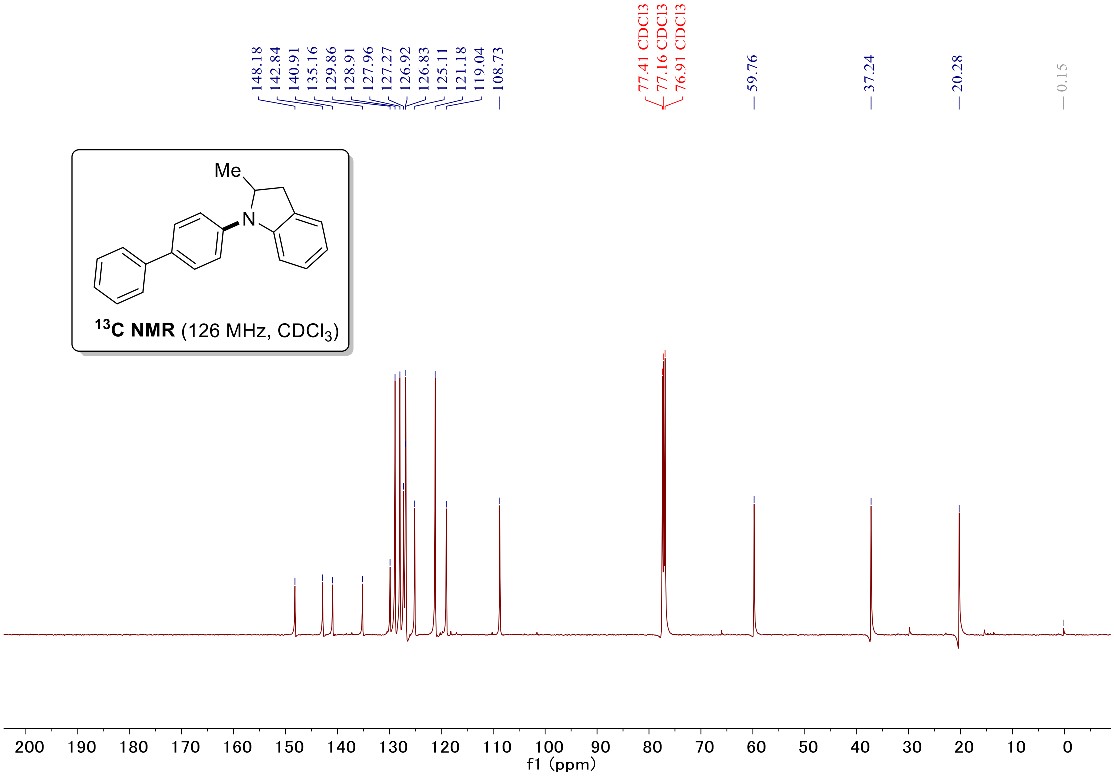

Supplementary Figure 225. ^{13}C NMR (126 MHz, $CDCl_3$, 25 °C) of compound **4ao**

1-([1,1'-Biphenyl]-4-yl)-1,2,3,4-tetrahydroquinoline (4ap)

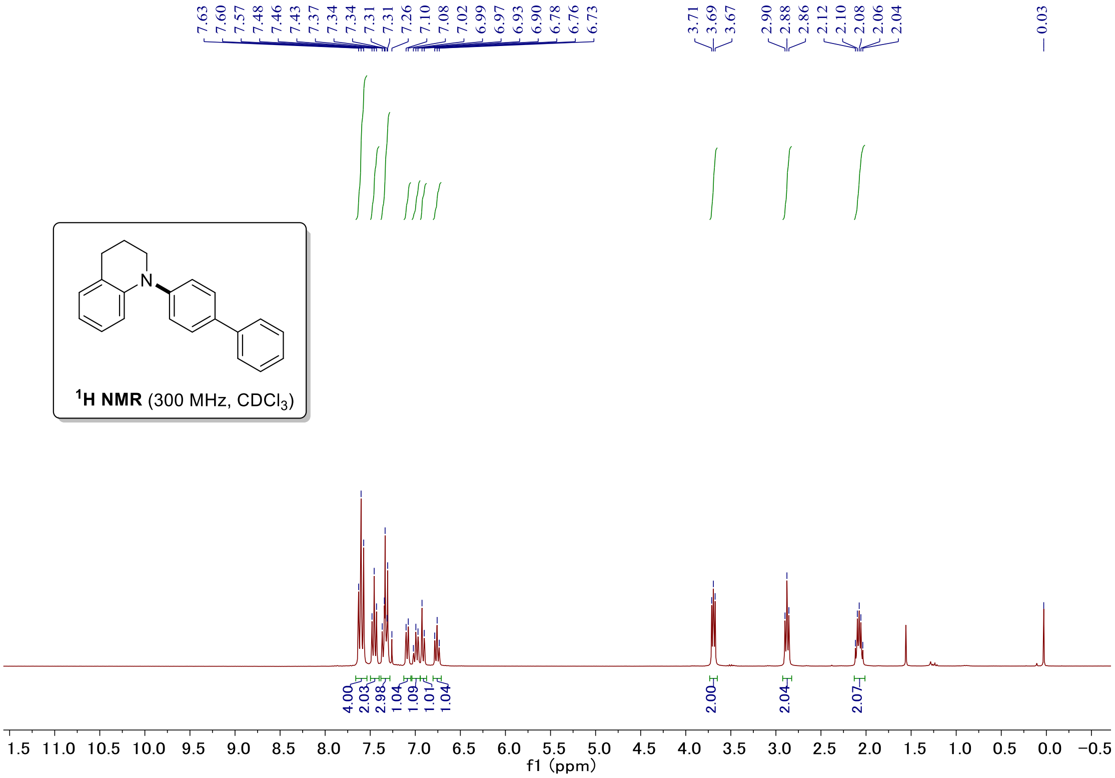

Supplementary Figure 226. ^{1}H NMR (300 MHz, $CDCl_3$, 25 °C) of compound **4ap**

4-([1,1'-Biphenyl]-4-yl)-3,4-dihydro-2*H*-benzo[*b*][1,4]oxazine (4aq)

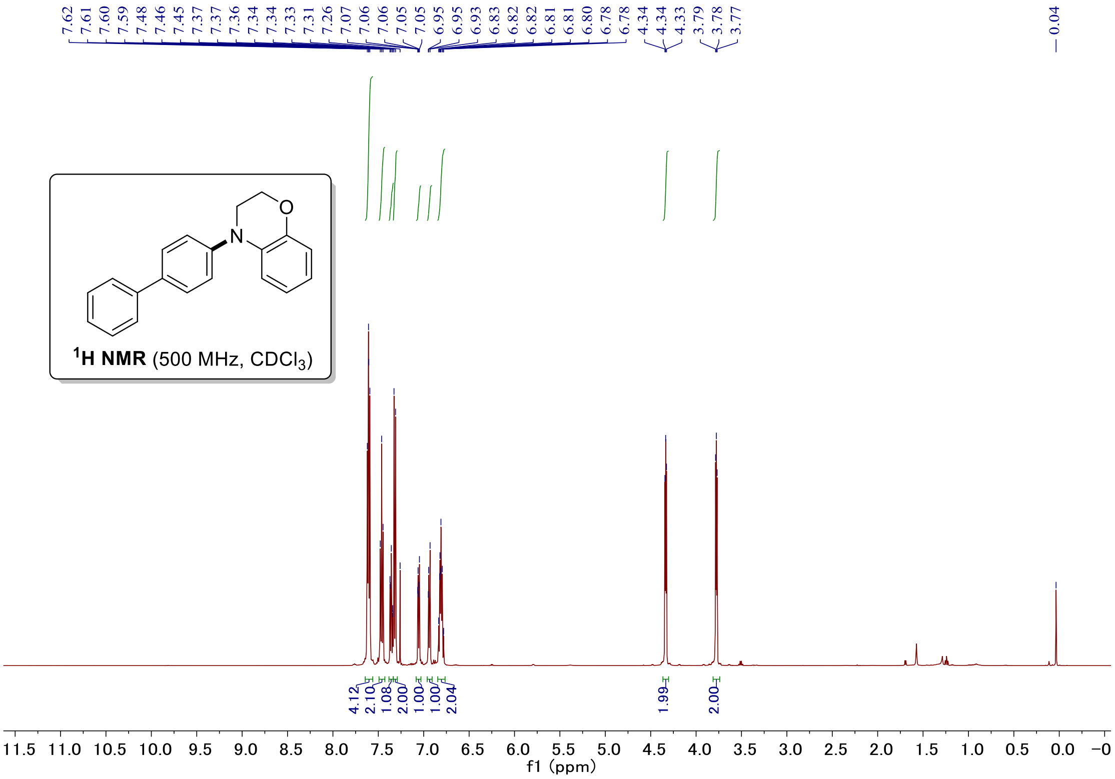

Supplementary Figure 227. ^{1}H NMR (500 MHz, $CDCl_3$, 25 °C) of compound **4aq**

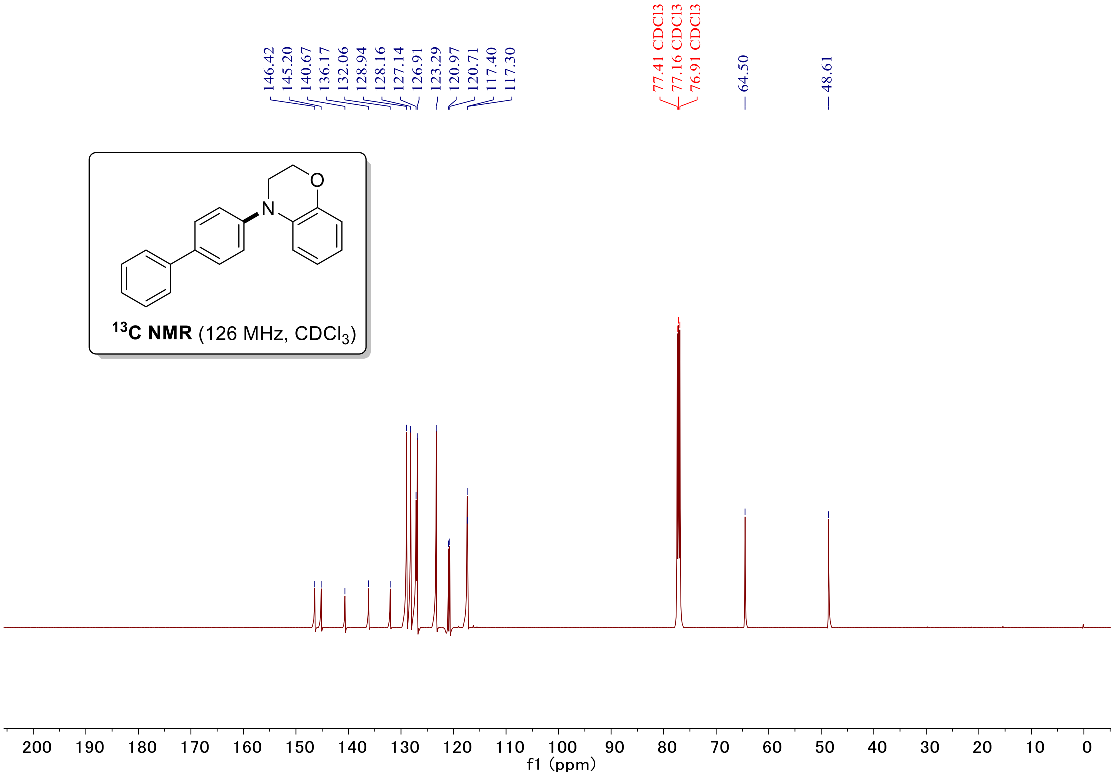

Supplementary Figure 228. ^{13}C NMR (126 MHz, $CDCl_3$, 25 °C) of compound **4aq**

4-([1,1'-Biphenyl]-4-yl)morpholine (4ar)

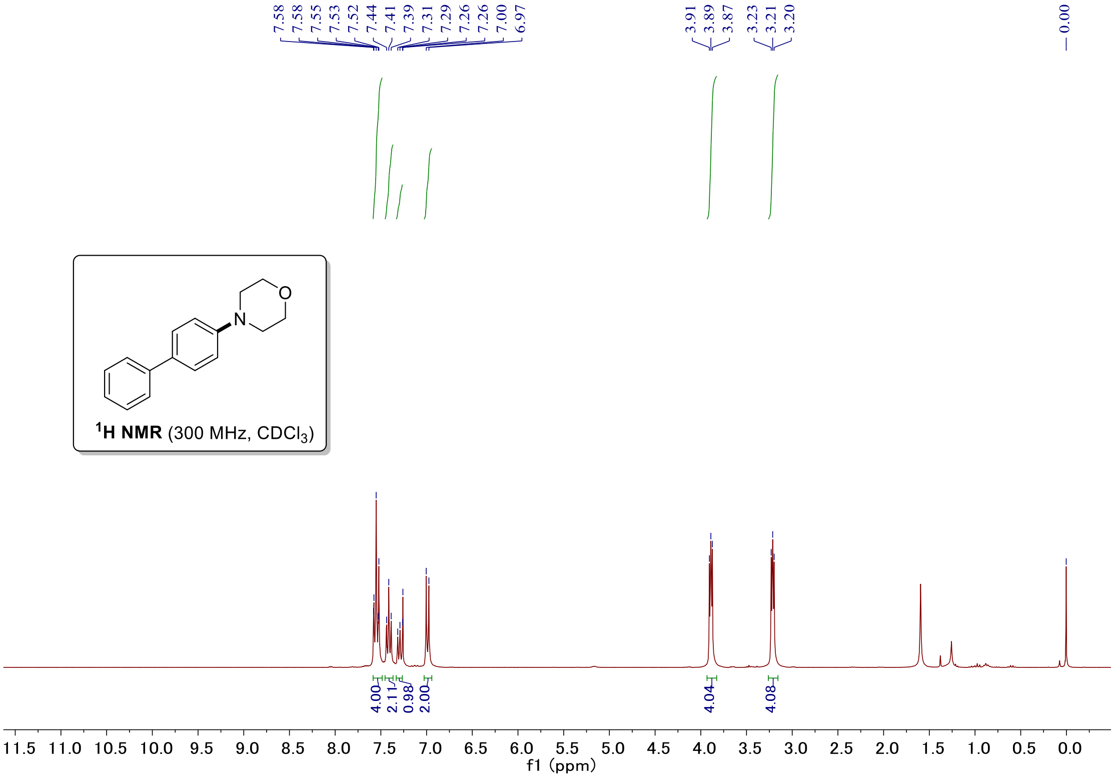

Supplementary Figure 229. ^{1}H NMR (300 MHz, $CDCl_3$, 25 °C) of compound **4ar**

1-([1,1'-Biphenyl]-4-yl)pyrrolidine (4as)

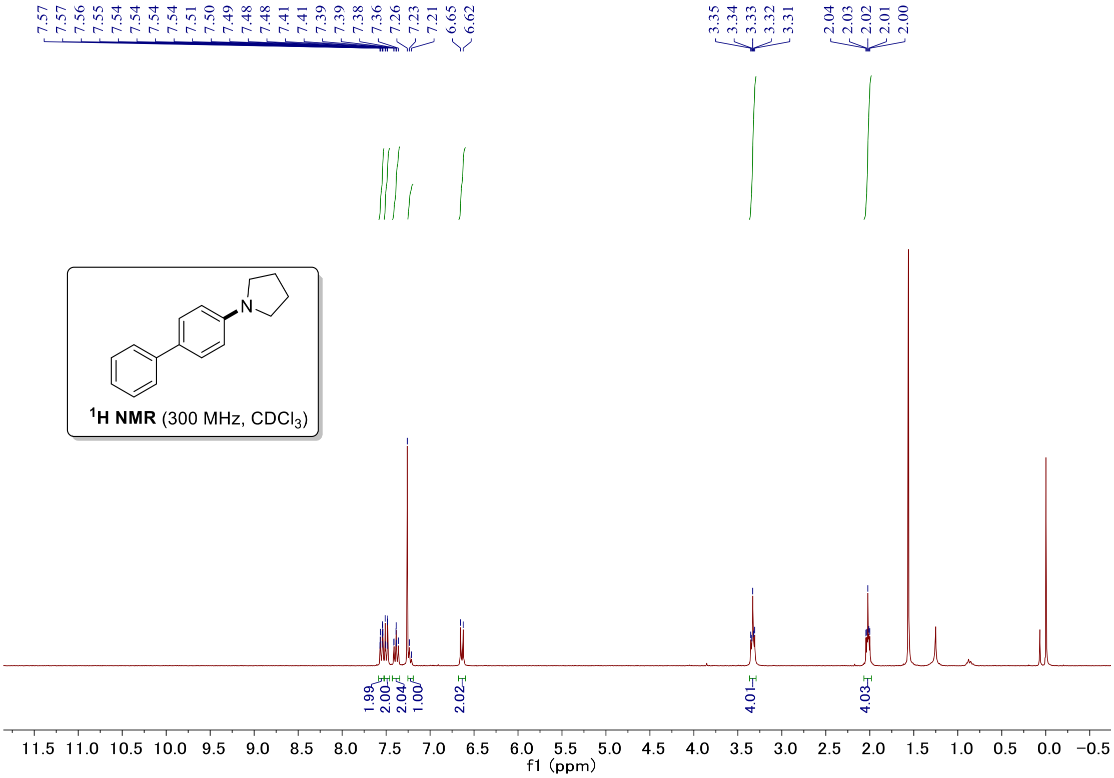

Supplementary Figure 230. ^{1}H NMR (300 MHz, $CDCl_3$, 25 °C) of compound **4as**

2-([1,1'-Biphenyl]-4-yl)-1,2,3,4-tetrahydroisoquinoline (4at)

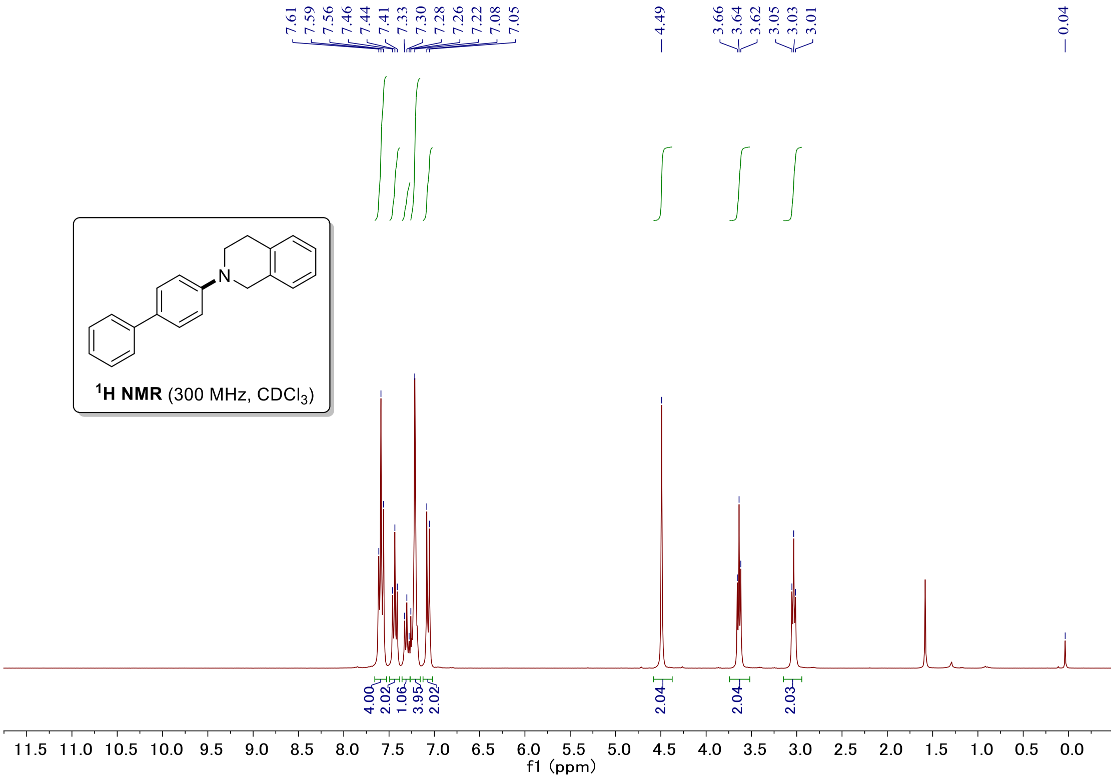

Supplementary Figure 231. ^{1}H NMR (300 MHz, $CDCl_3$, 25 °C) of compound **4at**

N,*N*-Diethyl-[1,1'-biphenyl]-4-amine (4au)

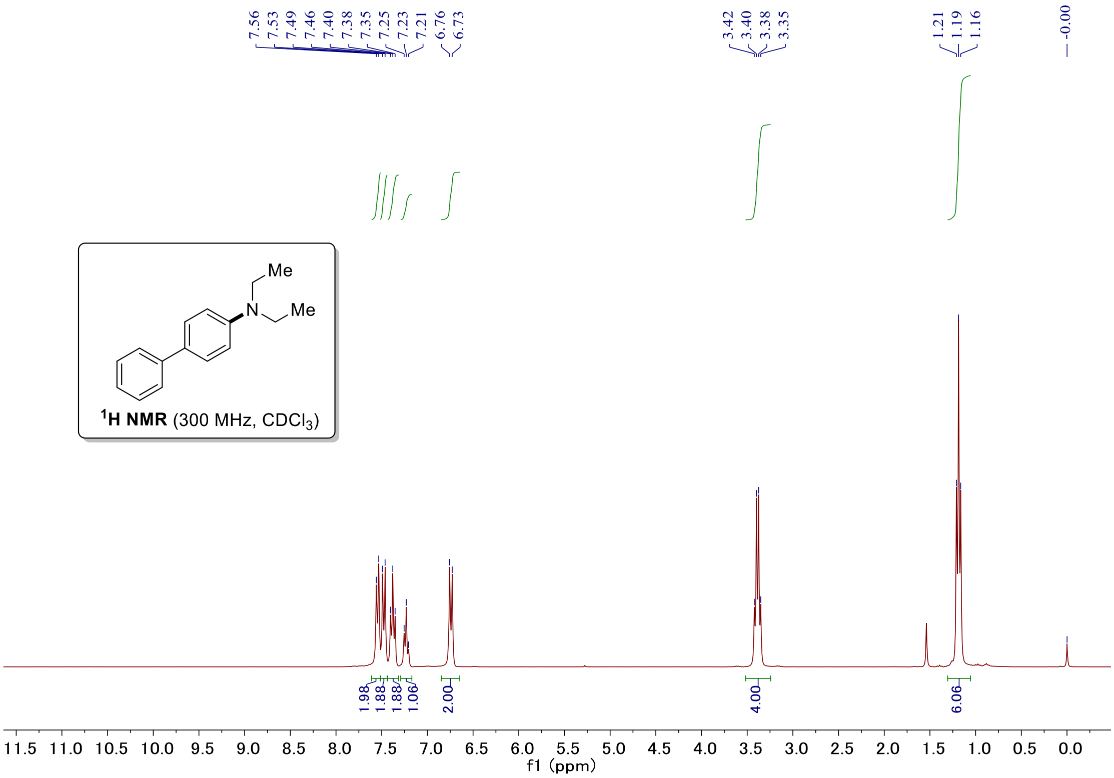

Supplementary Figure 232. ^{1}H NMR (300 MHz, $CDCl_3$, 25 °C) of compound **4au**

N-(Biphenyl-4-ylmethyl)-*N*-methylaniline (5aa)

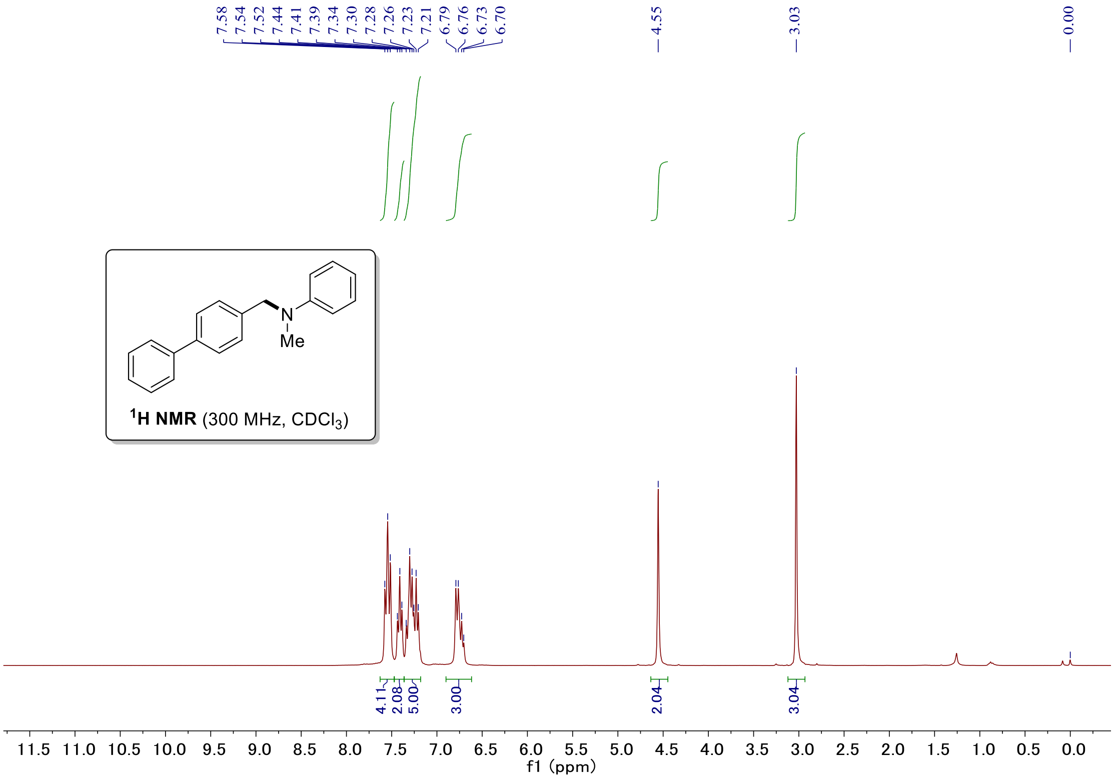

Supplementary Figure 233. ^{1}H NMR (300 MHz, $CDCl_3$, 25 °C) of compound **5aa**

N-Methyl-*N*-(4-(trifluoromethoxy)benzyl)aniline (5ba)

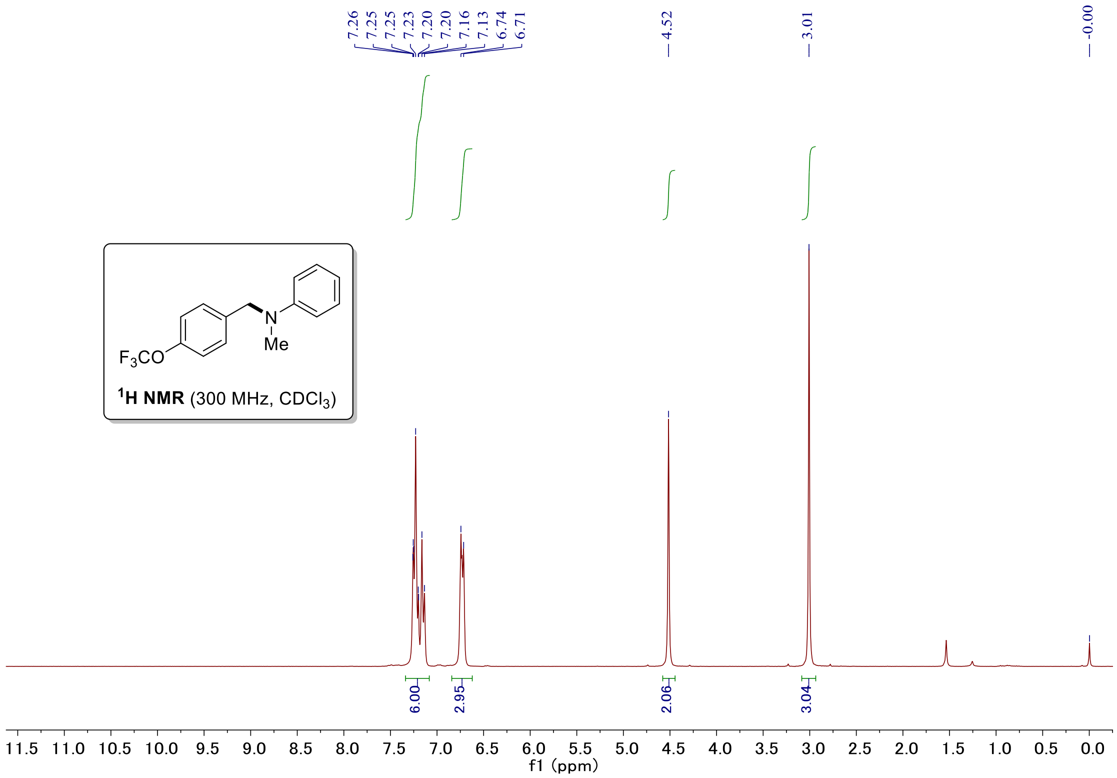

Supplementary Figure 234. ^{1}H NMR (300 MHz, $CDCl_3$, 25 °C) of compound **5ba**

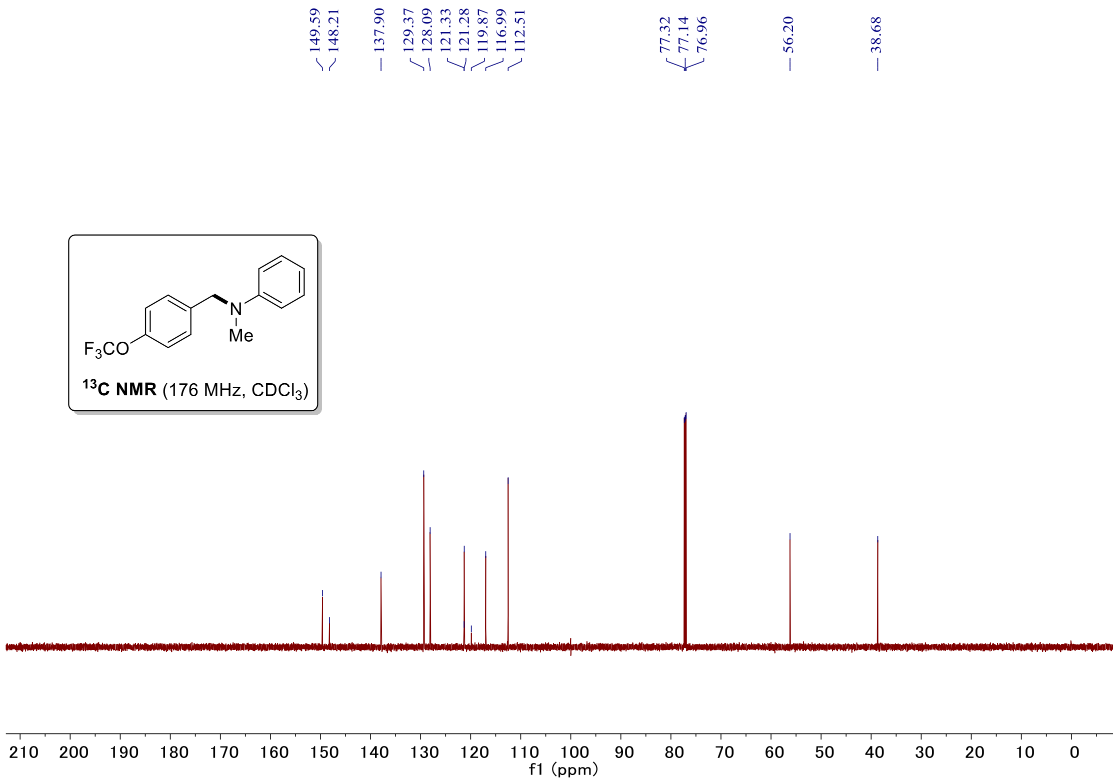

Supplementary Figure 235. ^{13}C NMR (176 MHz, $CDCl_3$, 25 °C) of compound **5ba**

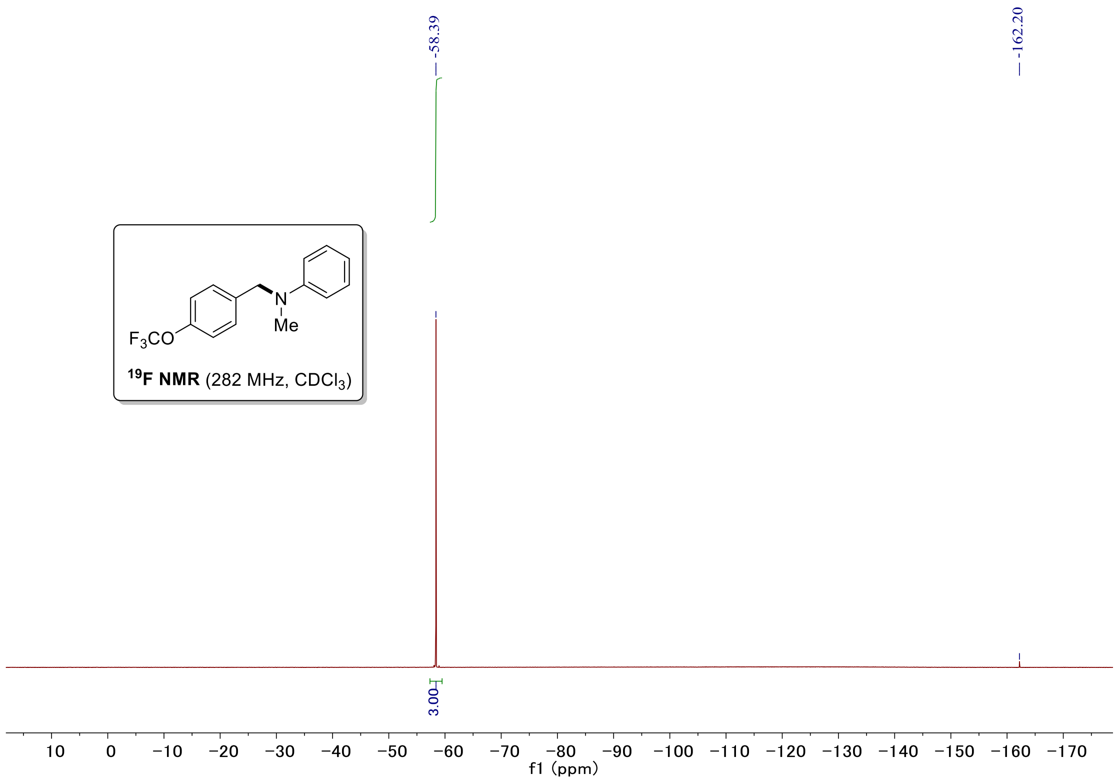

Supplementary Figure 236. ^{19}F NMR (282 MHz, $CDCl_3$, 25 °C) of compound **5ba**

N-Methyl-*N*-(naphthalen-1-ylmethyl)aniline (5ca)

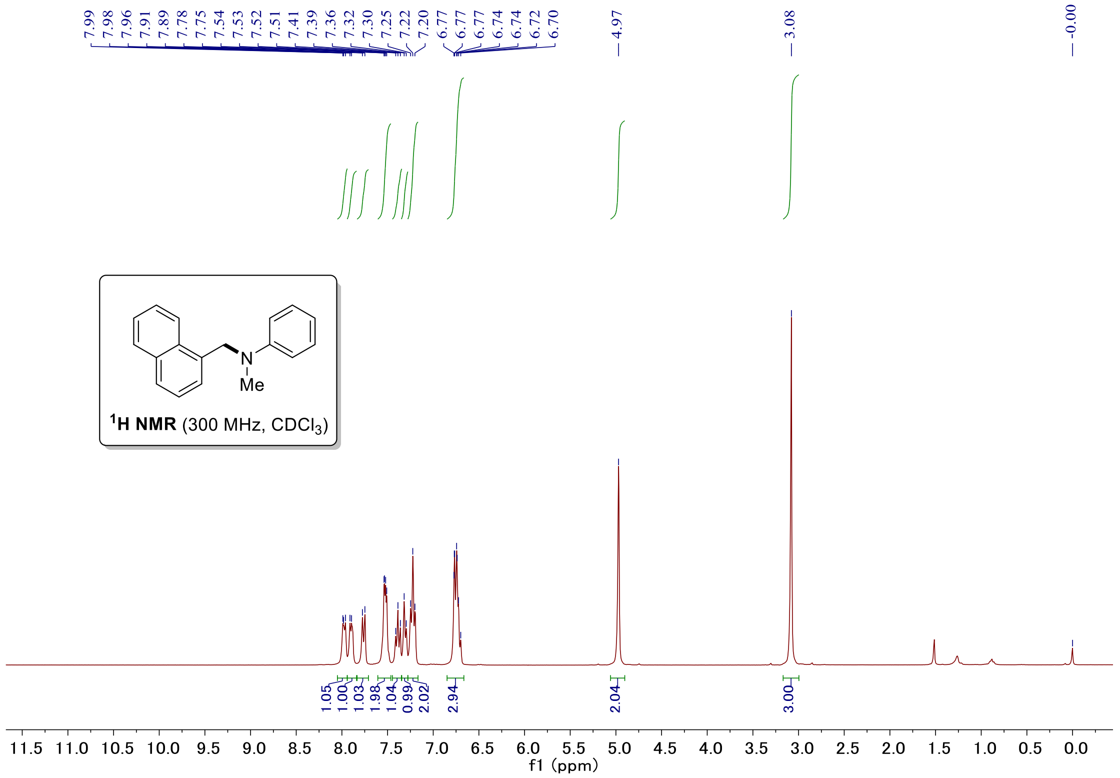

Supplementary Figure 237. ^{1}H NMR (300 MHz, $CDCl_3$, 25 °C) of compound **5ca**

N-Methyl-*N*-(1-(naphthalen-2-yl)ethyl)aniline (5da)

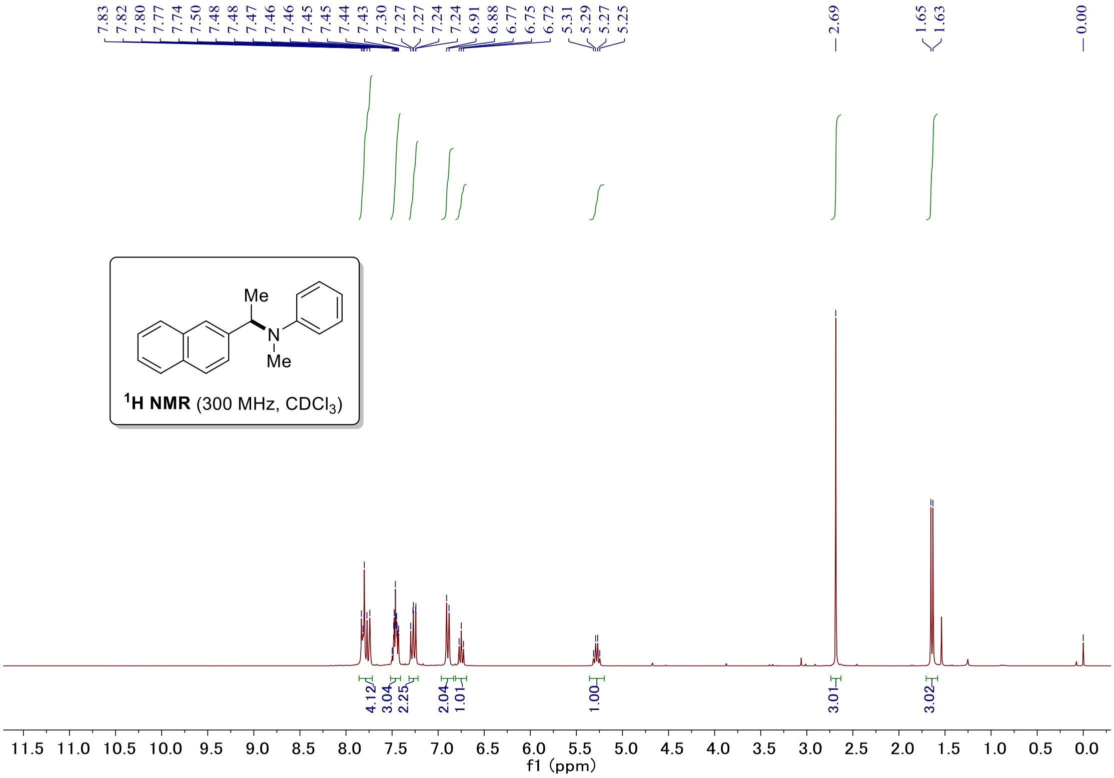

Supplementary Figure 238. ^{1}H NMR (300 MHz, $CDCl_3$, 25 °C) of compound **5da**

N-Decyl-*N*-methylaniline (5ea)

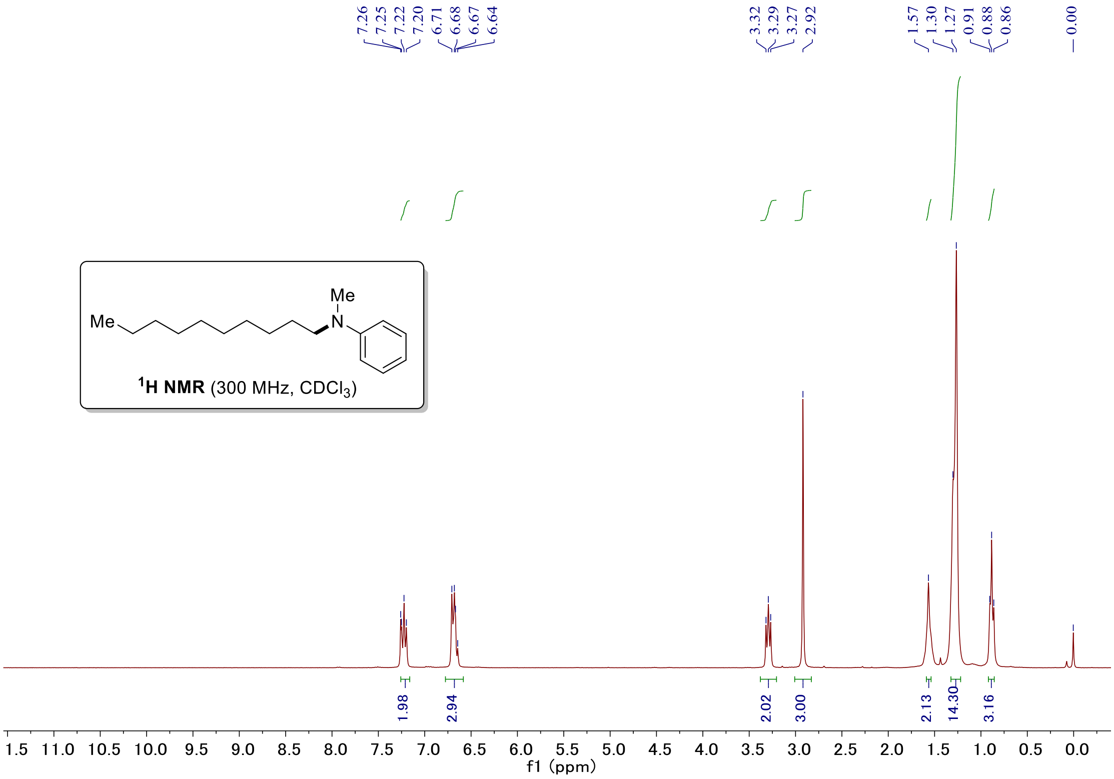

Supplementary Figure 239. ^{1}H NMR (300 MHz, $CDCl_3$, 25 °C) of compound **5ea**

***N*-(Methyl-*d*[3])-*N*-phenylbiphenyl-4-amine (*d*[3]-4aa)**

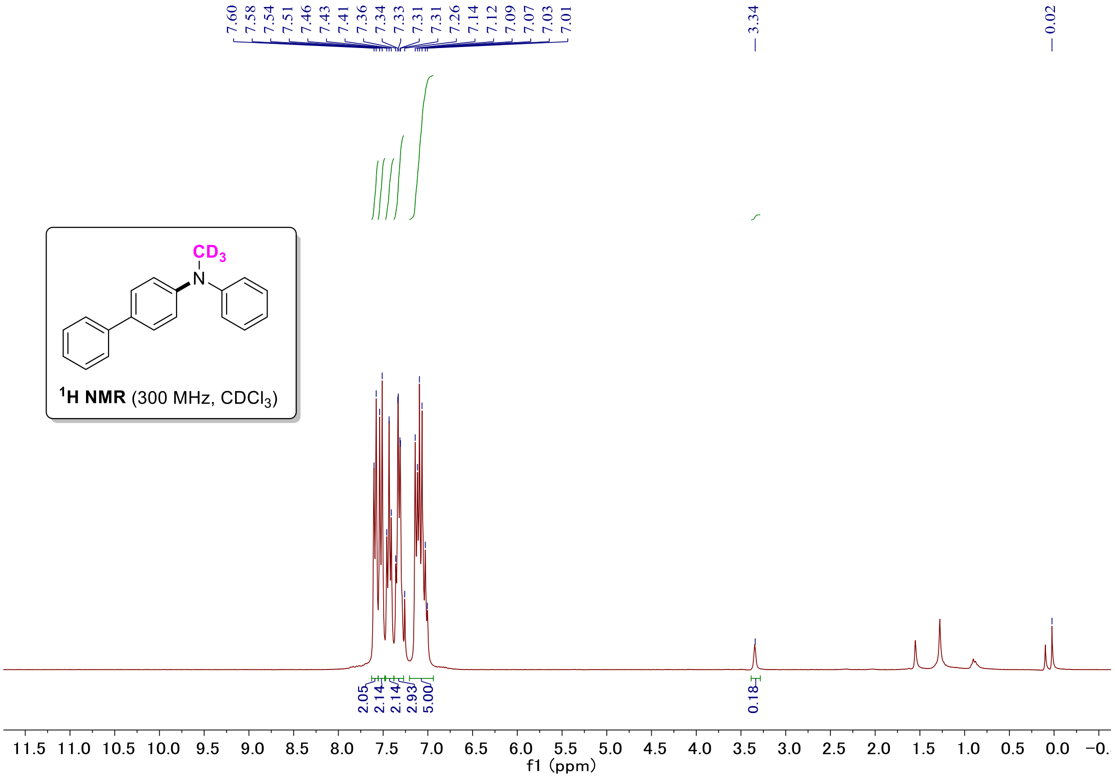

Supplementary Figure 240. ^{1}H NMR (300 MHz, $CDCl_3$, 25 °C) of compound ***d*[3]-4aa**

***N*-Methyl-*N*-phenyl-4-((((*R*)-2,5,7,8-tetramethyl-2-((4*R*,8*R*)-4,8,12-trimethyltridecyl)chroman-6-yl)oxy)methyl)aniline (4ada)**

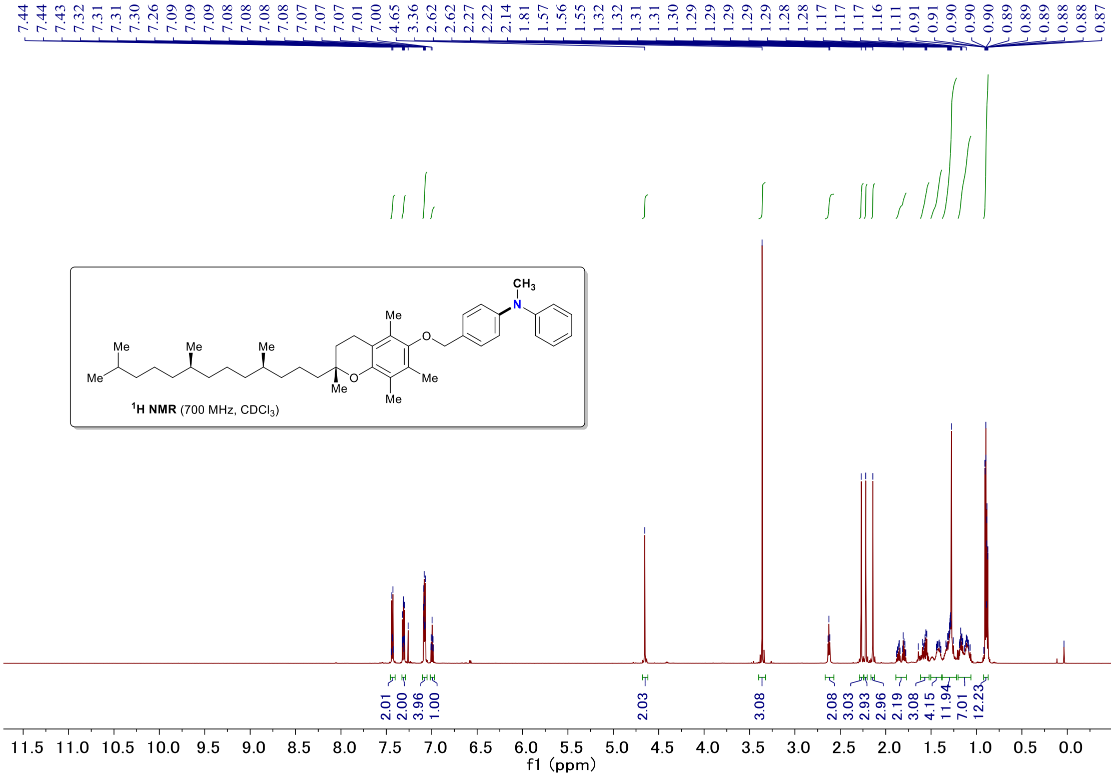

Supplementary Figure 241. ^{1}H NMR (700 MHz, $CDCl_3$, 25 °C) of compound **4ada**

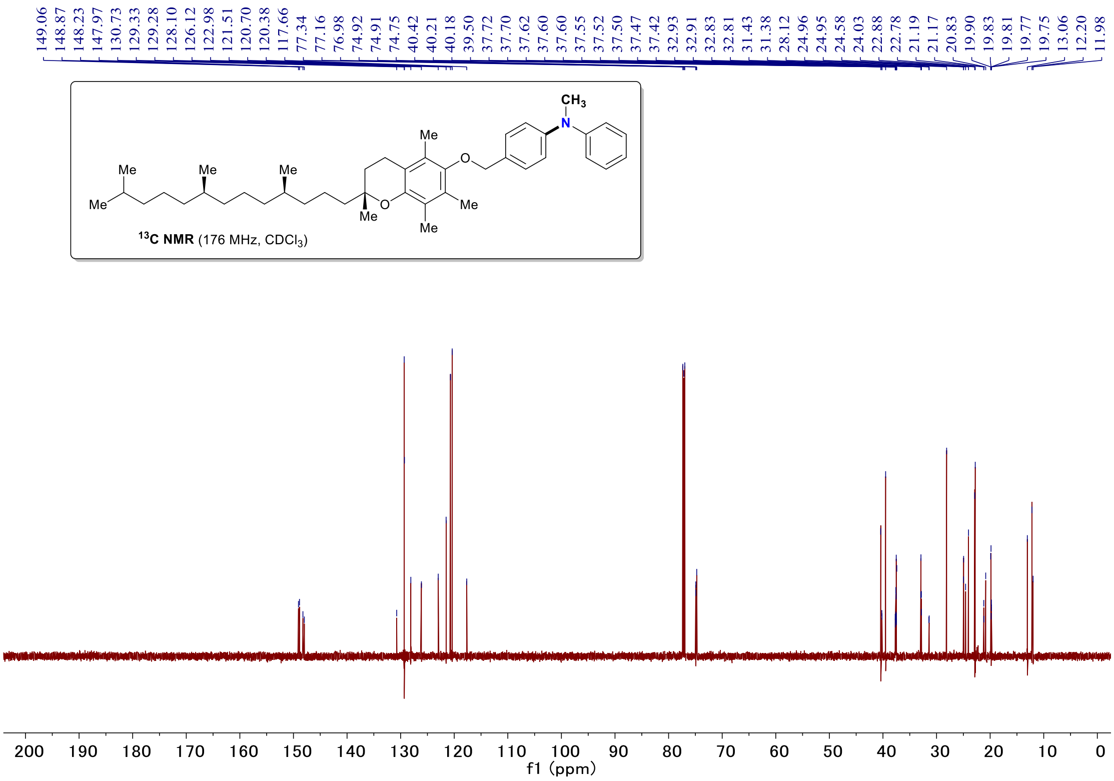

Supplementary Figure 242. ^{13}C NMR (176 MHz, $CDCl_3$, 25 °C) of compound **4ada**

(2*S*,5*R*)-2-*iso*-Propyl-5-methylcyclohexyl 4-([1,1'-biphenyl]-4-yl(methyl)amino)benzoate (4av)

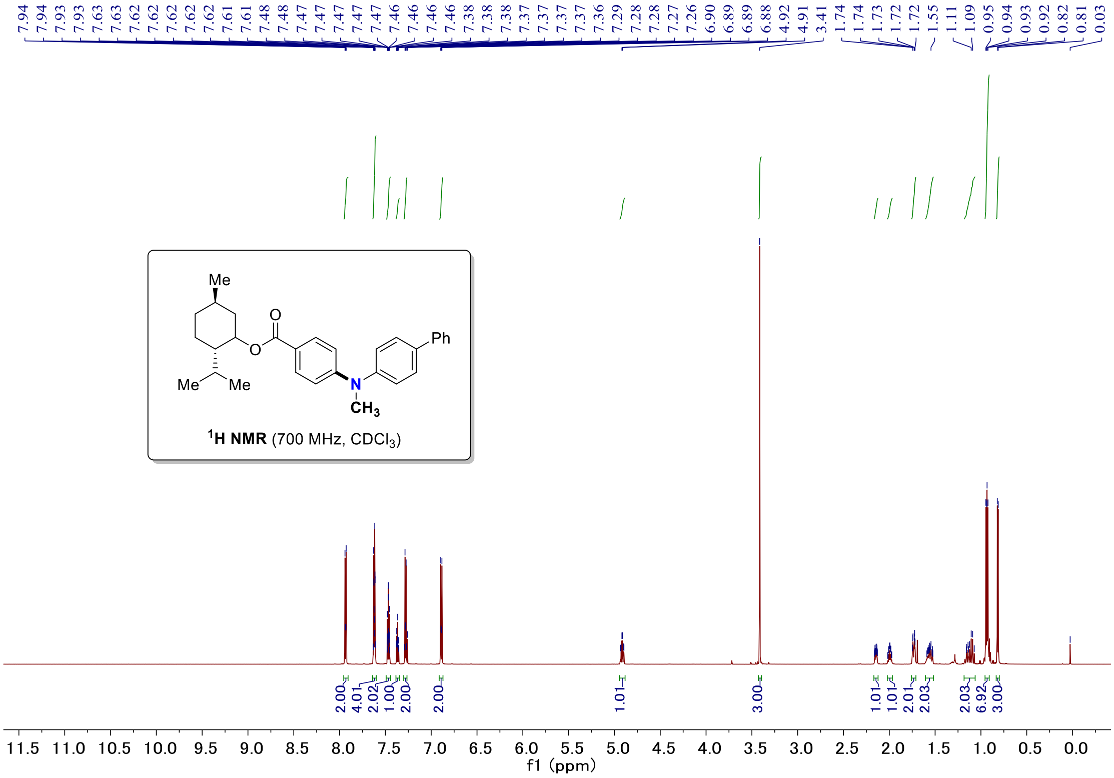

Supplementary Figure 243. ^{1}H NMR (700 MHz, $CDCl_3$, 25 °C) of compound **4av**

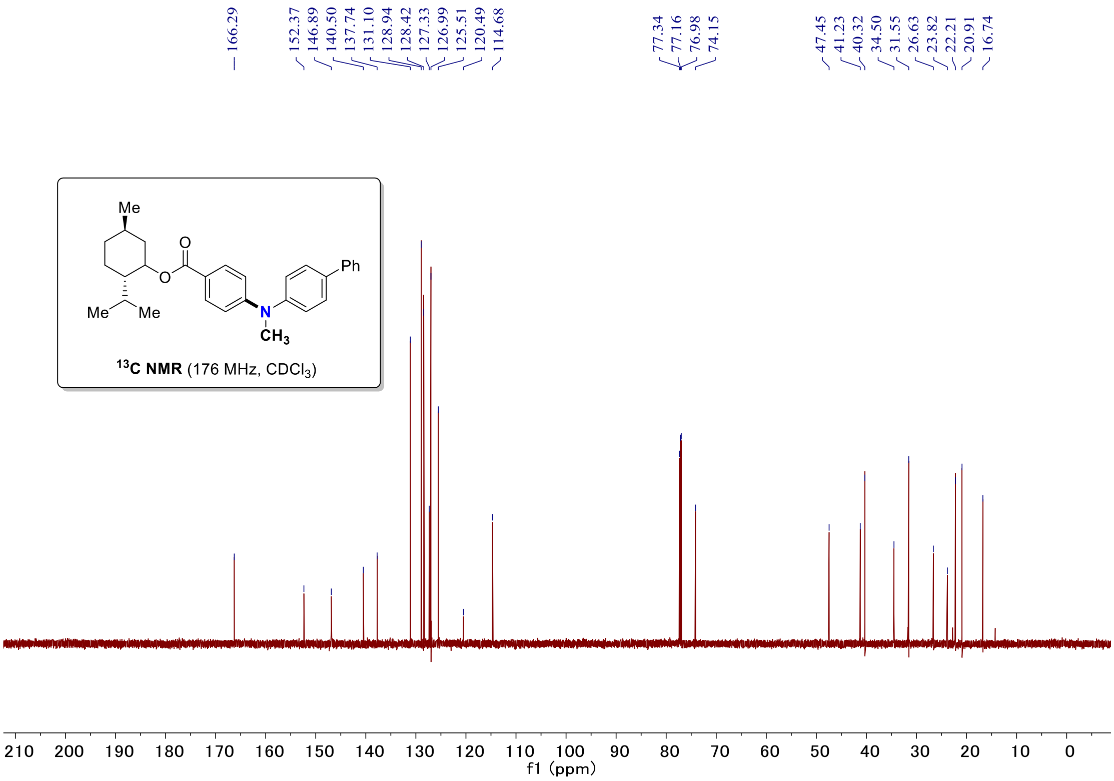

Supplementary Figure 244. ^{13}C NMR (176 MHz, $CDCl_3$, 25 °C) of compound **4av**

(2*S*,5*R*)-2-*iso*-Propyl-5-methylcyclohexyl 4-(((8*R*,9*S*,13*S*,14*S*)-17-methoxy-13-methyl-7,8,9,11,12,13,14,15,16,17-decahydro-6*H*-cyclopenta[*a*]phenanthren-3-yl)(methyl)amino)benzoate (4aev)

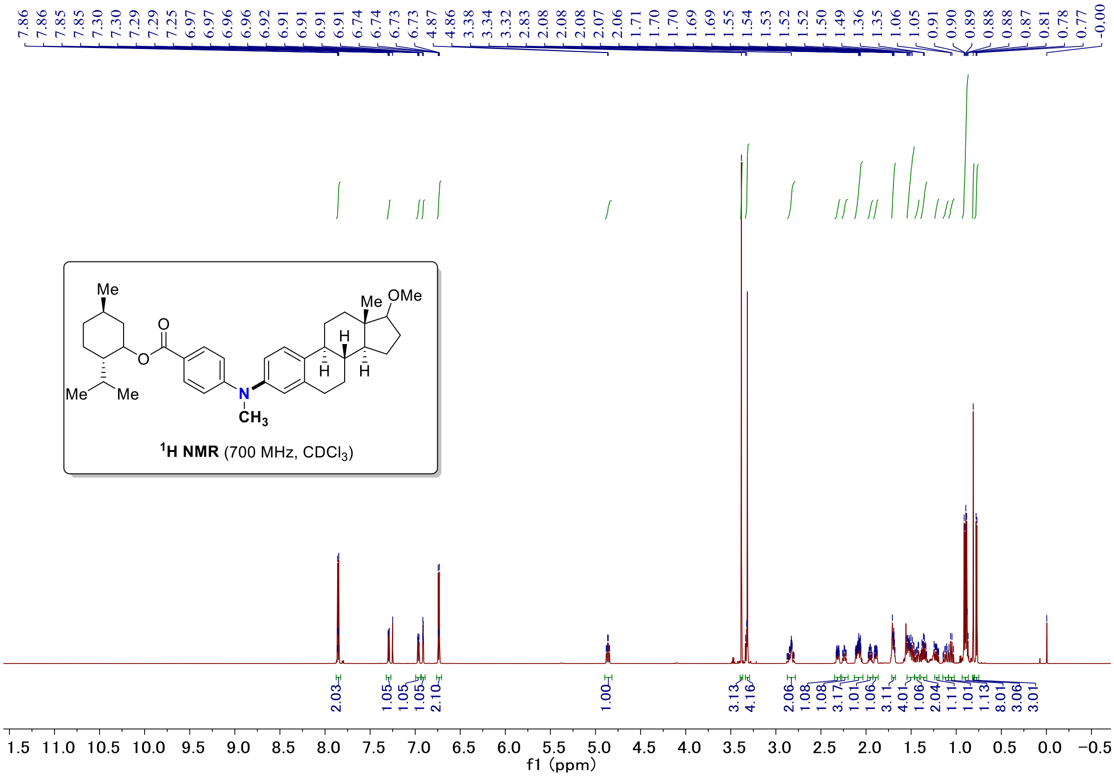

Supplementary Figure 245. ^{1}H NMR (700 MHz, $CDCl_3$, 25 °C) of compound **4aev**

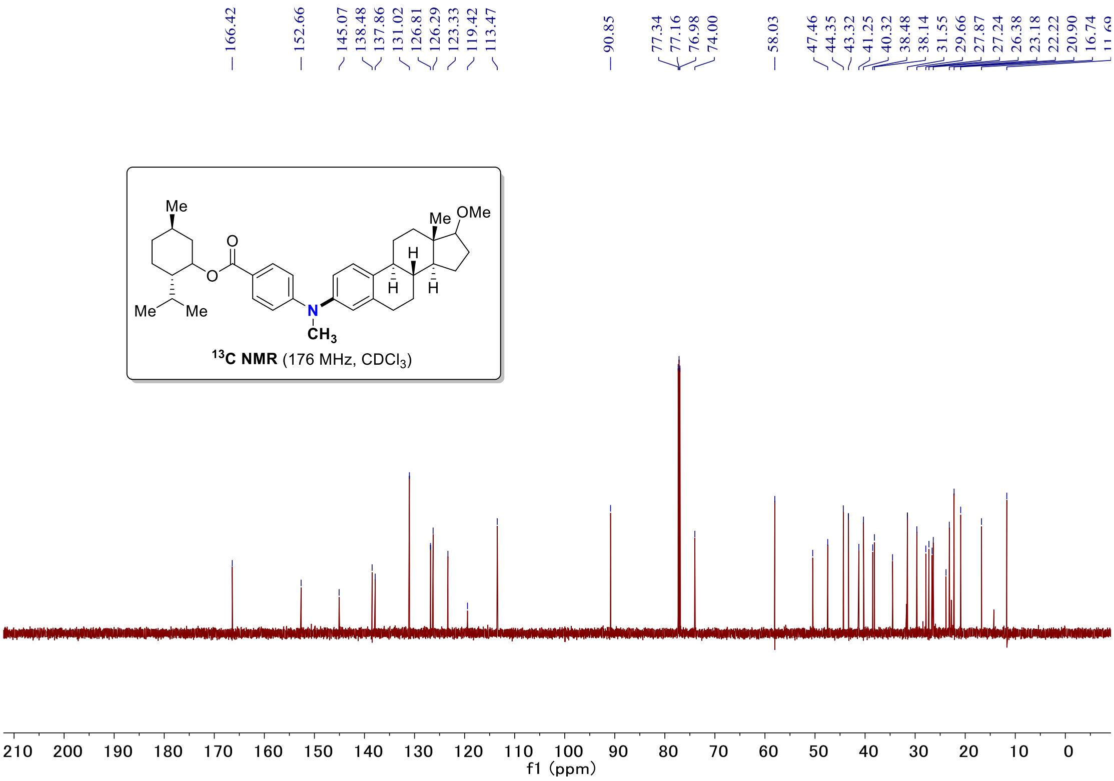

Supplementary Figure 246. ^{13}C NMR (176 MHz, $CDCl_3$, 25 °C) of compound **4aev**

(8*R*,9*S*,13*S*,14*S*)-17-Methoxy-13-methyl-*N*-(methyl-*d*^{3})-*N*-phenyl-7,8,9,11,12,13,14,15,16,17-decahydro-6*H*-cyclopenta[*a*]phenanthren-3-amine (*d*^{3}-4aea)

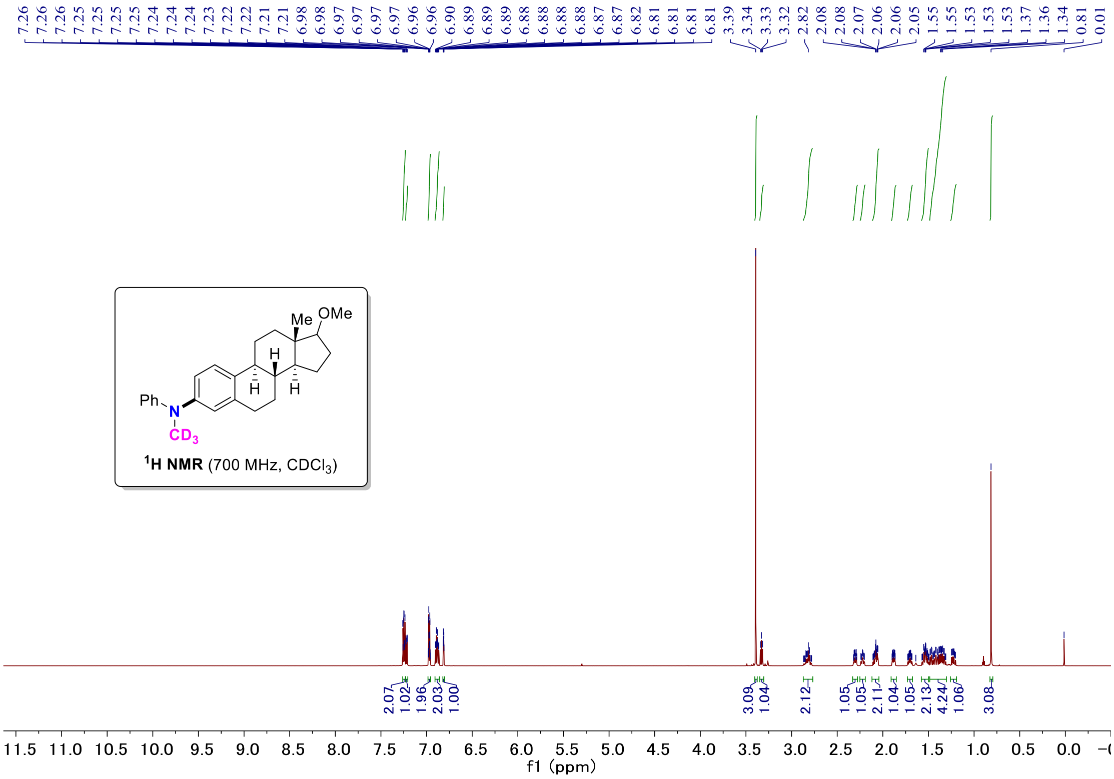

Supplementary Figure 247. ^1H NMR (700 MHz, $CDCl_3$, 25 °C) of compound ***d*^{3}-4aea**

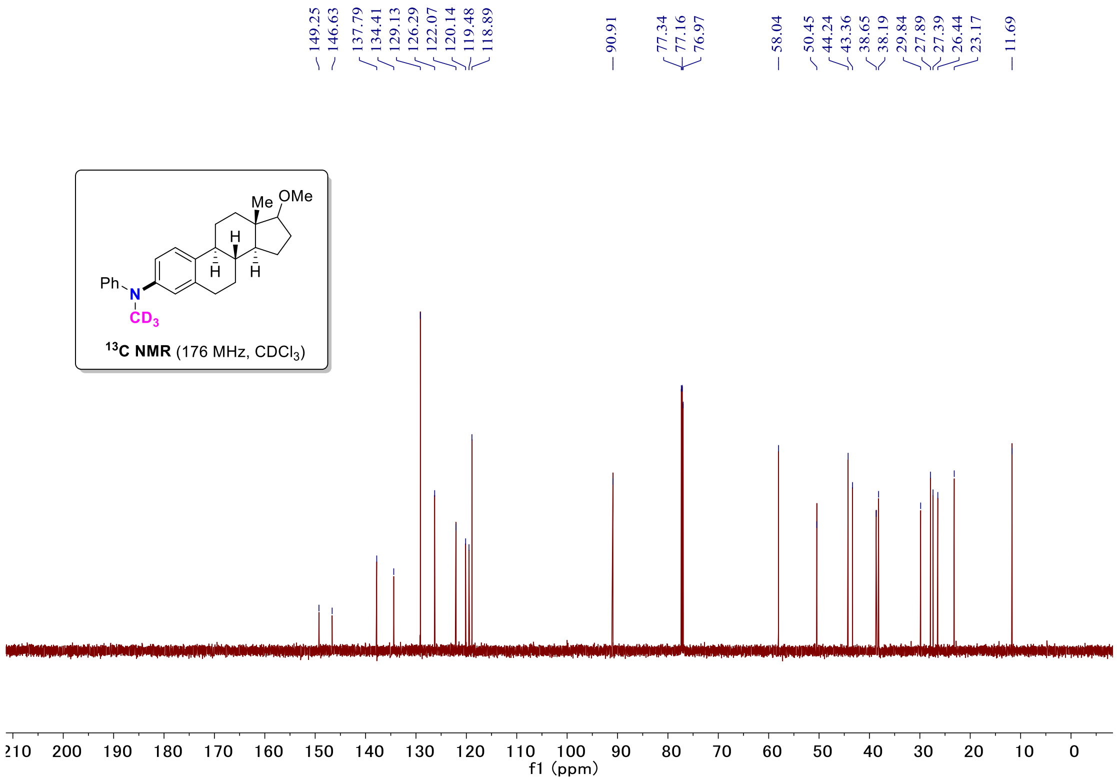

Supplementary Figure 248. ^{13}C NMR (176 MHz, $CDCl_3$, 25 °C) of compound ***d*^{3}-4aea**

4-((((1*R*,2*S*,5*R*)-2-Isopropyl-5-methylcyclohexyl)oxy)methyl)-*N*-(methyl-*d*^{3})-*N*-phenylaniline (*d*^{3}-4afa)

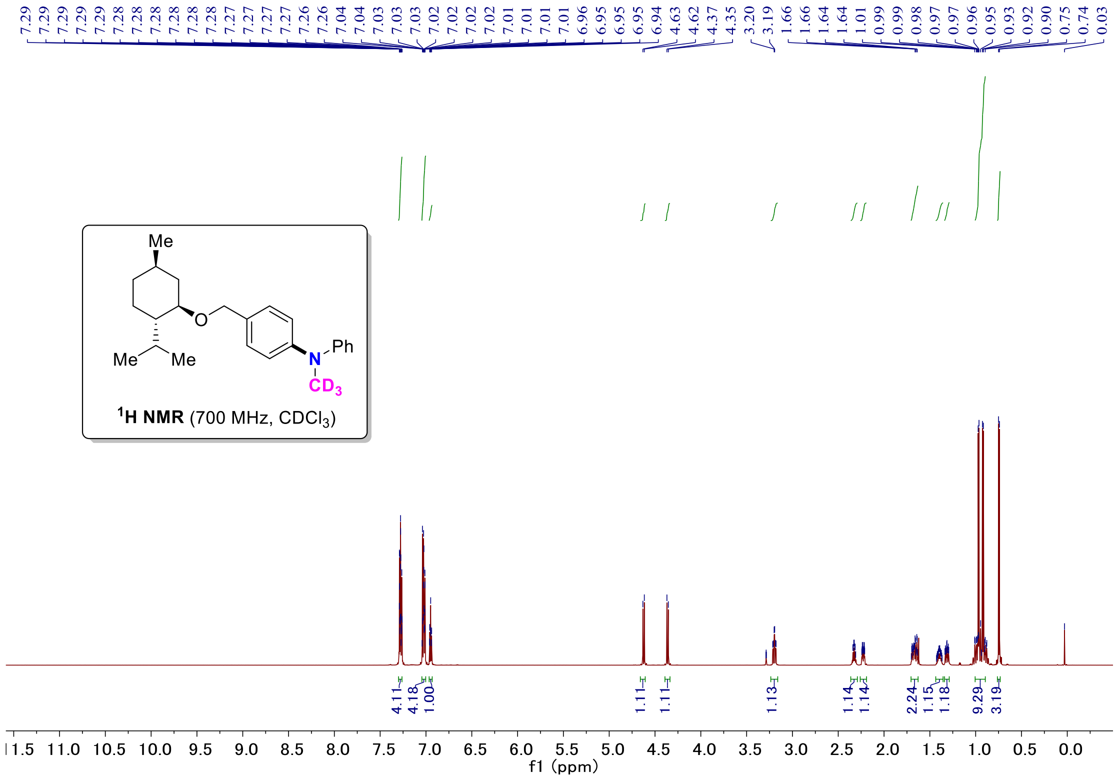

Supplementary Figure 249. ^{1}H NMR (700 MHz, $CDCl_3$, 25 °C) of compound ***d*^{3}-4afa**

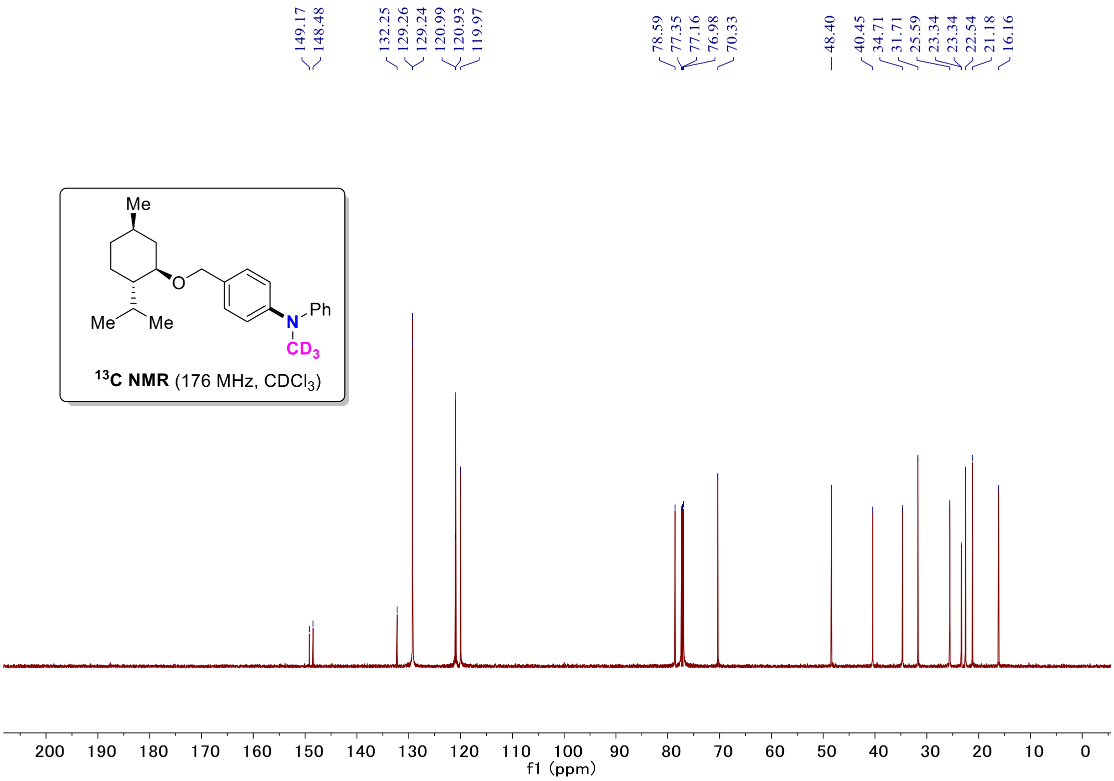

Supplementary Figure 250. ^{13}C NMR (176 MHz, $CDCl_3$, 25 °C) of compound ***d*³-4afa**

4-((Methyl-*d*³)(phenyl)amino)phenyl (*R*)-2-(6-methoxynaphthalen-2-yl)propanoate (*d*³-4aga)

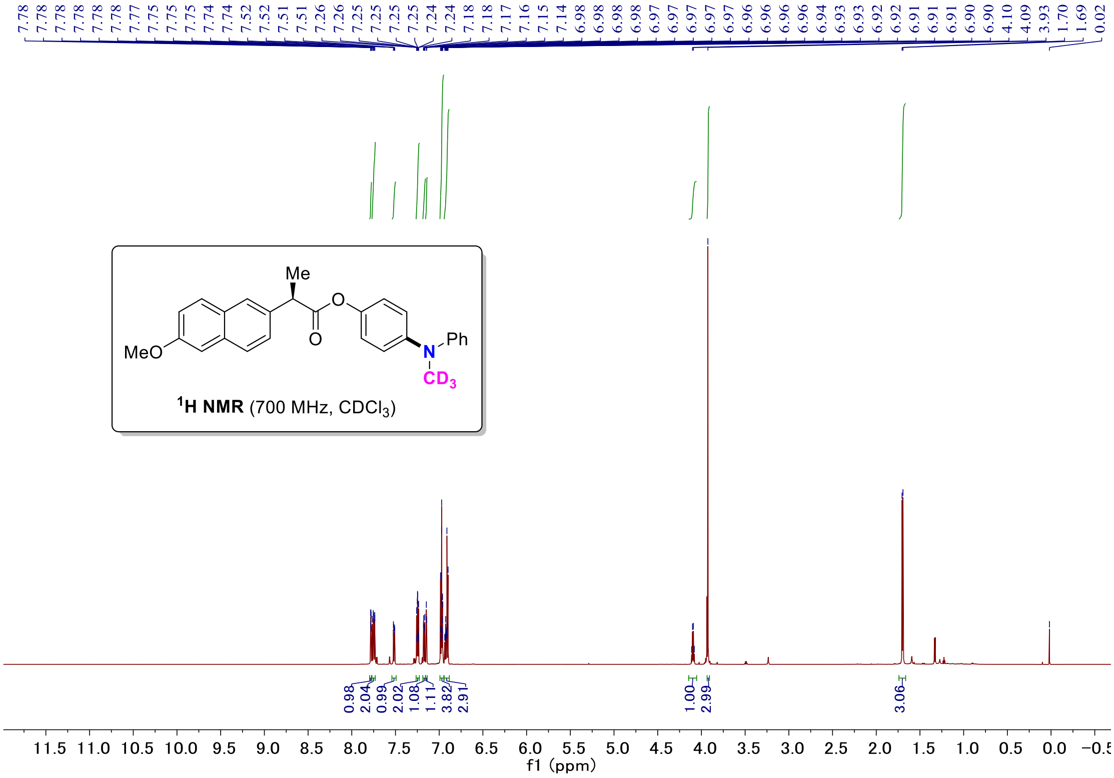

Supplementary Figure 251. ^{1}H NMR (700 MHz, $CDCl_3$, 25 °C) of compound ***d*³-4aga**

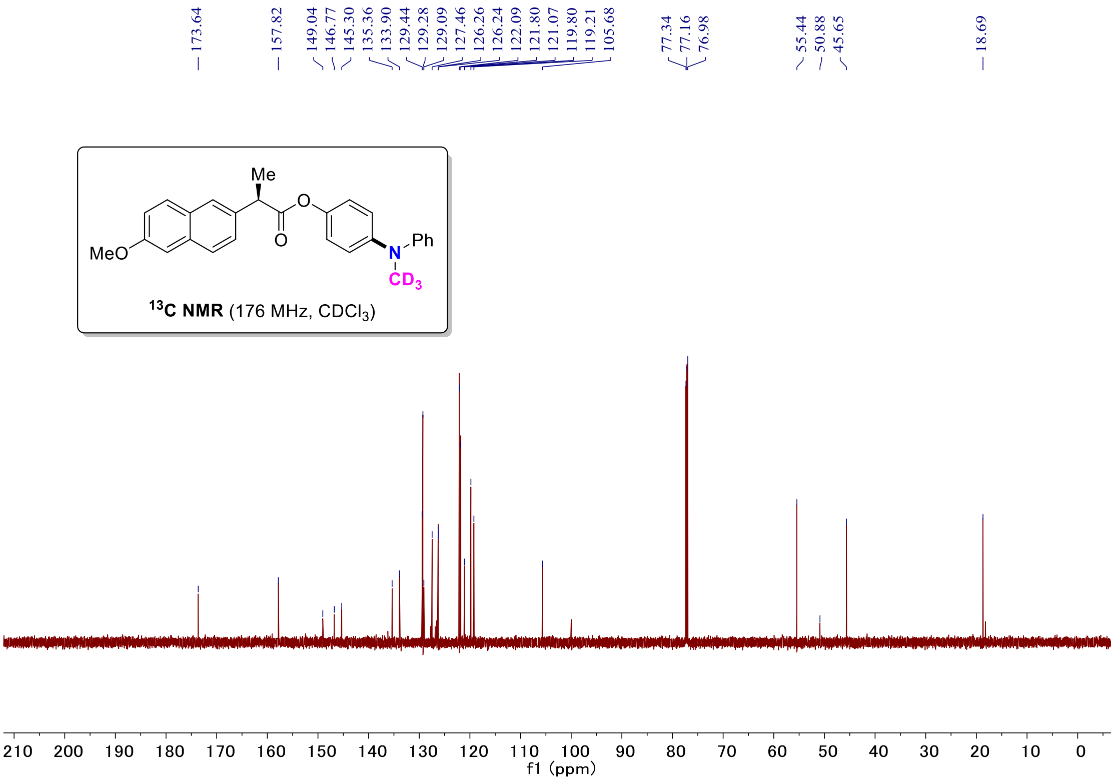

Supplementary Figure 252. ^{13}C NMR (176 MHz, $CDCl_3$, 25 °C) of compound ***d*^{3}-4aga**

***N*-(Methyl-*d*^{3})-*N*-phenyl-4-((((*R*)-2,5,7,8-tetramethyl-2-((4*R*,8*R*)-4,8,12-trimethyltridecyl)chroman-6-yl)oxy)methyl)aniline (*d*^{3}-4ada)**

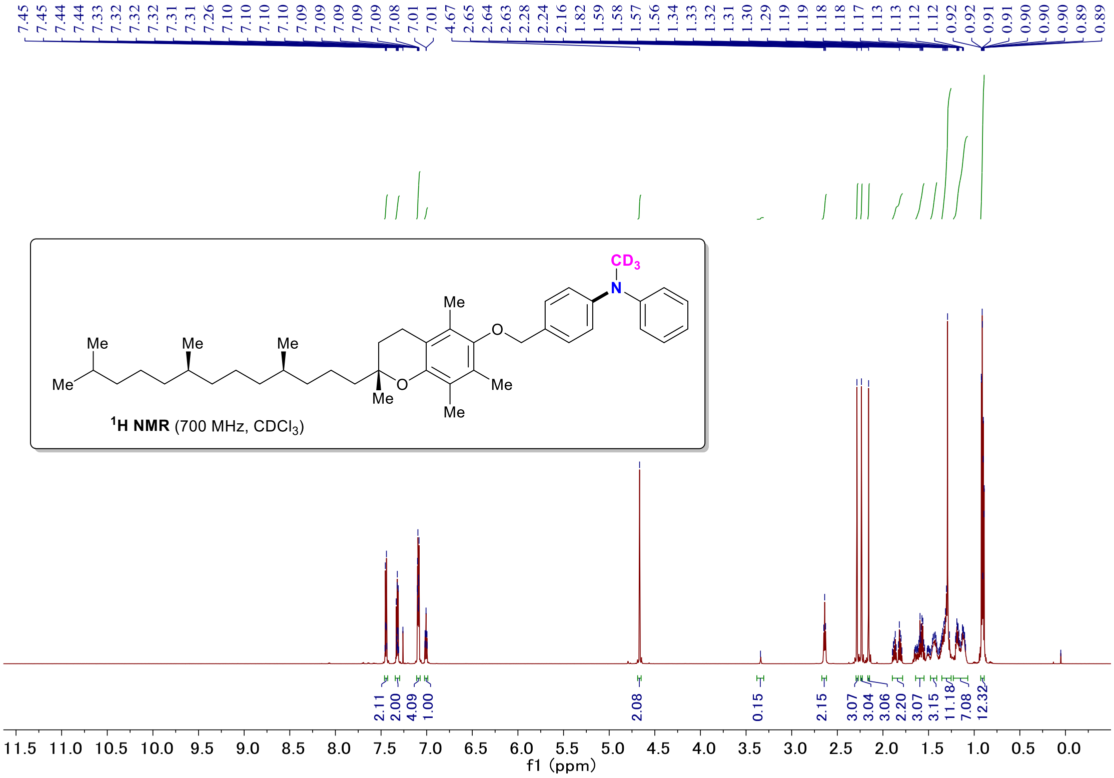

Supplementary Figure 253. ^{1}H NMR (700 MHz, $CDCl_3$, 25 °C) of compound ***d*^{3}-4ada**

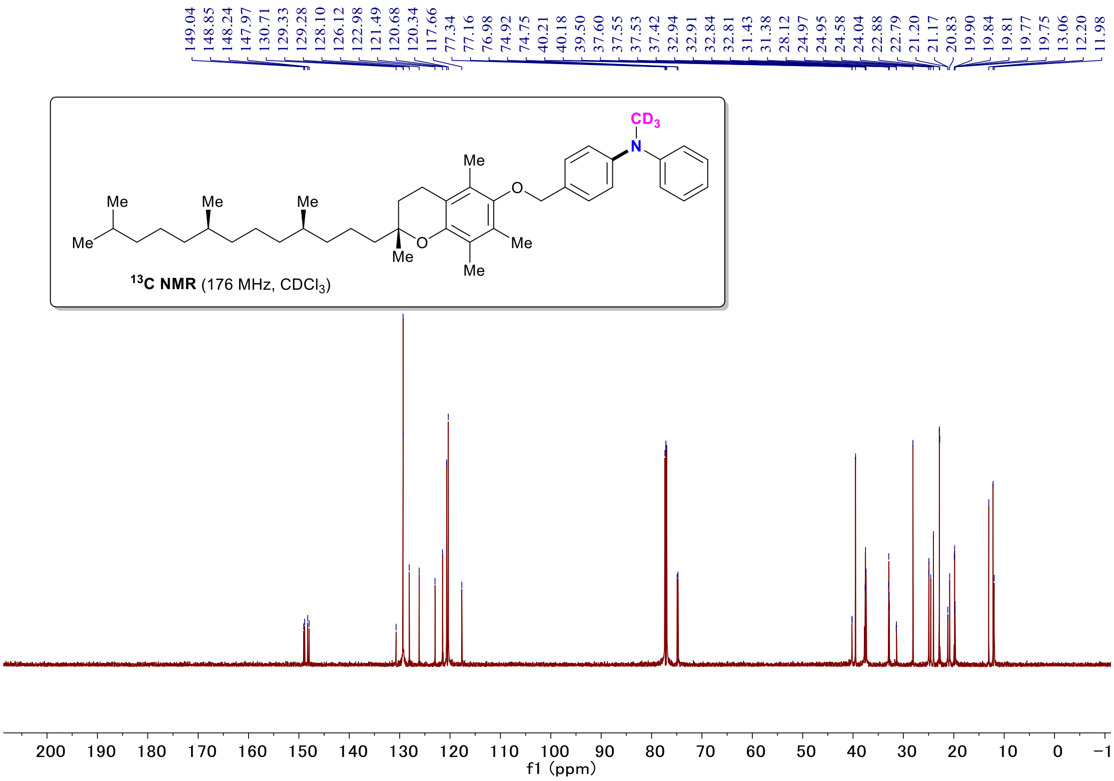

Supplementary Figure 254. ^{13}C NMR (176 MHz, $CDCl_3$, 25 °C) of compound ***d*^{3}-4ada**

1,2-Dimethyl-1,2-diphenylhydrazine (7)

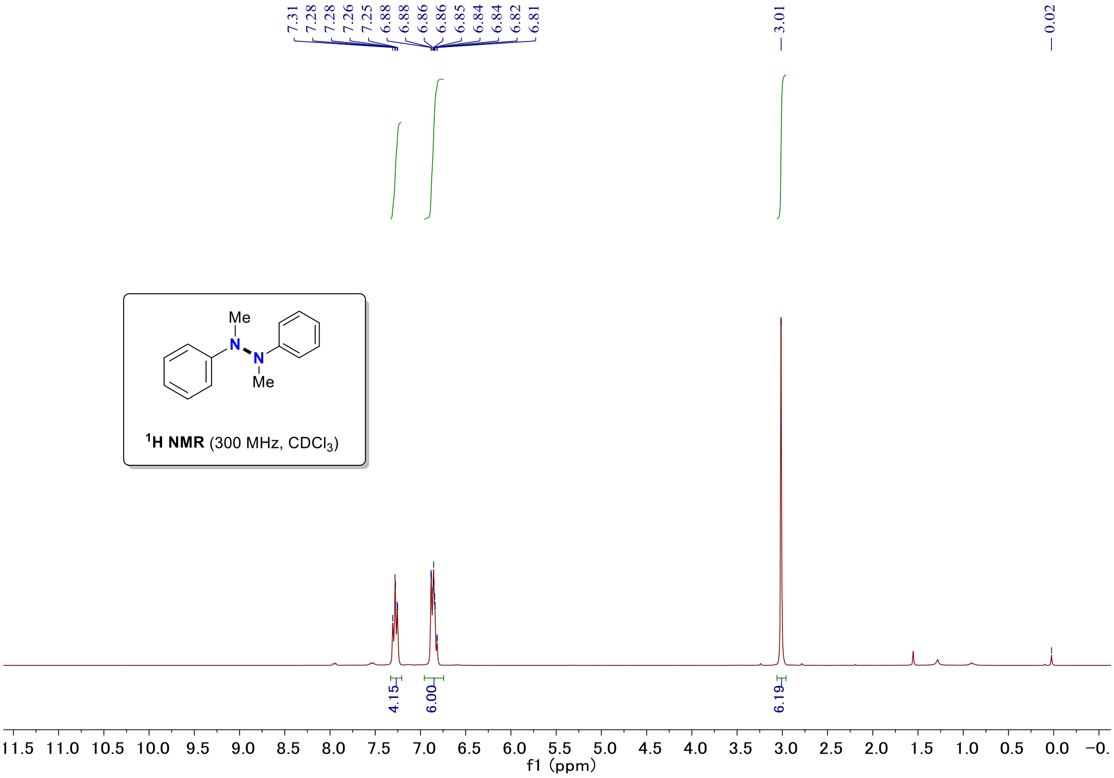

Supplementary Figure 255. ^{1}H NMR (300 MHz, $CDCl_3$, 25 °C) of compound **7**

5. Supplementary References

1. Cui, B., Jia, S., Tokunaga, E. & Shibata, N. Defluorosilylation of fluoroarenes and fluoroalkanes. *Nat. Commun.* **9**, 4393 (2018).
2. Quasdorf, K. W. et al. Suzuki−Miyaura cross-coupling of aryl carbamates and sulfamates: experimental and computational studies. *J. Am. Chem. Soc.* **133**, 6352–6363 (2011).
3. Zhou, J. et al. Catalyst-free carbosilylation of alkenes using silyl boronates and organic fluorides via selective C-F bond activation. *Nat. Commun.* **12**, 3749 (2021).
4. Qiao, X. et al. Weak epitaxy growth of phthalocyanine on inducing layers of fluorinated 5,5″-bis(biphenyl-4-yl)-2,2′:5′,2″-terthiophene. *J. Phys. Chem. B* **116**, 1812–1818 (2012).
5. Hua, X. et al. Inherent vs apparent chemoselectivity in the Kumada–Corriu cross-coupling reaction. *Org. Lett.* **18**, 5312–5315 (2016).
6. Gurung, S. K. et al. Copper-catalyzed Suzuki–Miyaura coupling of arylboronate esters: transmetalation with (PN)CuF and identification of intermediates. *Org. Lett.* **16**, 1264–1267 (2014).
7. Niwa, T., Ochiai, H., Watanabe, Y. & Hosoya, T. Ni/Cu-catalyzed defluoroborylation of fluoroarenes for diverse C–F bond functionalizations. *J. Am. Chem. Soc.* **137**, 14313–14318 (2015).
8. Music, A. et al. Photocatalyzed transition-metal-free oxidative cross-coupling reactions of tetraorganoborates. *Chem. Eur. J.* **27**, 4322–4326 (2021).
9. Chen, X. et al. Rhodium-catalyzed site-selective coupling of indoles with diazo esters: C4-alkylation versus C2-annulation. *Org. Lett.* **19**, 6184-6187 (2017).
10. Furuya, T., Strom, A. E. & Ritter, T. Silver-mediated fluorination of functionalized aryl stannanes. *J. Am. Chem. Soc.* **131**, 1662–1663 (2009).
11. Blessley, G. et al. Palladium-catalyzed substitution and cross-coupling of benzylic fluorides. *Org. Lett.* **14**, 2754–2757 (2012).
12. Hirano, K. et al. Boron trifluoride-mediated alkylation of diphenylphosphine with *tert*-alkyl fluoride. *Org. Lett.* **6**, 4873–4875 (2004).
13. Umemoto, T. et al. Discovery of 4-*tert*-butyl-2,6-dimethylphenylsulfur trifluoride as a deoxofluorinating agent with high thermal stability as well as unusual resistance to aqueous hydrolysis, and its diverse fluorination capabilities including deoxofluoro-arylsulfinylation with

high stereoselectivity. *J. Am. Chem. Soc.* **132**, 18199–18205 (2010).

14. Cheng, H. et al. Photoinduced hydroarylation and cyclization of alkenes with luminescent platinum(II) complexes. *Angew. Chem. Int. Ed.* **60**, 1383–1389 (2021).
15. Lu, Z. K. et al. Copper-catalyzed amination of aromatic halides with 2-*N*, *N*-dimethylaminoethanol as solvent. *Tetrahedron Lett.* 44, 6289–6292 (2003).
16. Wang, L., Neumann, H. & Beller, M. Palladium-catalyzed methylation of nitroarenes with methanol. *Angew. Chem. Int. Ed.* **58**, 5417–5421 (2019).
17. Falk, E., Gasser, V. C. M. & Morandi, B. Synthesis of *N*-alkyl anilines from arenes via iron-promoted aromatic C–H amination. *Org. Lett.* **23**, 1422–1426 (2021).
18. Li, F., Xie, J., Shan, H., Sun, C. & Chen, L. General and efficient method for direct *N*-monomethylation of aromatic primary amines with methanol. *RSC Adv.* **2**, 8645–8652 (2012).
19. Choi, S., Park, J., Yu, E., Sim, J. & Park, C. Electrosynthesis of dihydropyrano[4,3-*b*]indoles based on a double oxidative [3+3] cycloaddition. *Angew. Chem. Int. Ed.* **132**, 11984–11989 (2020).
20. Pflug, N. C., Schmitt, M. & Mcneill, K. Development of *N*-cyclopropylanilines to probe the oxidative properties of triplet-state photosensitizers. *Environ. Sci. Technol.* **53**, 4813–4822 (2019).
21. Nykaza, T. V., Yang, J. & Radosevich, A. T. PEt_3-mediated deoxygenative C–N coupling of nitroarenes and boronic acids. *Tetrahedron* **75**, 3248–3252 (2019).
22. Sarki, N. et al. Simple $RuCl_3$-catalyzed *N*-methylation of amines and transfer hydrogenation of nitroarenes using methanol. *ChemCatChem* **13**, 1722–1729 (2021).
23. Ke, L. et al. Catalytic selective oxidative coupling of secondary *N*-alkylanilines: an approach to azoxyarene. *Org. Lett.* **21**, 4008–4013 (2019).
24. Boebel, T. A. & Hartwig, J. F. Iridium-catalyzed preparation of silylboranes by silane borylation and their use in the catalytic borylation of arenes. *Organometallics* **27**, 6013–6019 (2008).
25. Yamamoto, E. et al. Tris(trimethylsilyl)silylboronate esters: novel bulky, air- and moisture-stable silylboronate ester reagents for boryl substitution and silaboration reactions. *Organometallics* **36**, 3019–3022 (2017).
26. Shao, Q. L. et al. Solvent-free mechanochemical Buchwald-Hartwig amination of aryl chlorides without inert gas protection. *Tetrahedron Lett.* **59**, 2277–2280 (2018).
27. Cho, S. & Wang, Q. 1,2-Difunctionalization of aryl triflates: a direct and modular access to diversely functionalized anilines. *Org. Lett.* **22**, 1670–1674 (2020).

28. Chen, Z., Chen, X. & So, C. M. Palladium-catalyzed C(sp^2)–N bond cross-coupling with triaryl phosphates. *J. Org. Chem.* **84**, 6366–6376 (2019).

29. Ali, M. H. & Buchwald, S. L. An improved method for the palladium-catalyzed amination of aryl iodides. *J. Org. Chem.* **66**, 2560–2565 (2001).

30. Bhojgude, S. S. et al. Employing arynes in transition-metal-free monoarylation of aromatic tertiary amines. *Org. Lett.* **15**, 5452–5455 (2013).

31. Weber, P. et al. A highly active ylide-functionalized phosphine for palladium-catalyzed aminations of aryl chlorides. *Angew. Chem. Int. Ed.* **58**, 3203–3207 (2019).

32. Lipshutz, B. H. et al. Copper + Nickel-in-Charcoal (Cu−Ni/C): a bimetallic, heterogeneous catalyst for cross-couplings. *Org. Lett.* **10**, 4279–4282 (2008).

33. Barros, M. T., Dey, S. S. & Maycock, C. D. Metal-free synthesis of secondary arylamines: an aliphatic-to-aromatic transformation. *Eur. J. Org. Chem.* **2013**, 742–747 (2013).

34. Matsumoto, K. et al. A highly selective palladium-catalyzed aerobic oxidative aniline–aniline cross-coupling reaction. *Org. Lett.* **21**, 7279–7283 (2019).

35. Tian, H. et al. Radical alkylation of C(sp3)–H bonds with diacyl peroxides under catalyst-free conditions. *Chem. Commun.* **55**, 14813–14816 (2019).

36. Zhang, Y. et al. CuI/DMPAO-catalyzed *N*-arylation of acyclic secondary amines. *Org. Lett.***14**, 3056–3059 (2012).

37. Houle, C. et al. Thiourea-catalyzed C−F bond activation: amination of benzylic fluorides. *Chem. Eur. J.* **26**, 10620–10625 (2020).

38. Gui, Y. Y. et al. Coupling of C(sp3)–H bonds with C(sp2)–O electrophiles: mild, general and selective. *Chem. Commun.* **53,** 1192–1195 (2017).

39. Wang, Z. et al. A facile one-pot process for the formation of hindered tertiary amines. *Molecules* **17**, 5151–5163 (2012).

40. Chung, H. & Chung, Y. K. Cobalt–rhodium heterobimetallic nanoparticle-catalyzed *N*-alkylation of amines with alcohols to secondary and tertiary amines. *J. Org. Chem.* **83**, 8533–8542 (2018).

41. Bredihhin, A. & Uno M. Effective strategy for the systematic synthesis of hydrazine derivatives. *Tetrahedron* **64**, 6788–6793 (2008).

42. O'Hagan, D. Understanding organofluorine chemistry. an introduction to the C–F bond. *Chem. Soc. Rev.* **37**, 308–319 (2008).

43. Glock, C., Görls, H. & Westerhausen, M. Electronic, steric, and ligand influence on the solid-state structures of substituted sodium and potassium anilides. *Eur. J. Inorg. Chem.* **2011**, 5288–5298 (2011).
